# B(C_6_F_5_)_3_‐Catalyzed Regiodivergent Thioetherifications of Alkenes *via* Thiiranium Intermediates: Experimental and Computational Insights

**DOI:** 10.1002/chem.202404236

**Published:** 2024-12-23

**Authors:** Nusaybah Alotaibi, Rasool Babaahmadi, Sampurna Das, Emma Richards, Thomas Wirth, Milan Pramanik, Rebecca L. Melen

**Affiliations:** ^1^ Cardiff Catalysis Institute School of Chemistry Cardiff University Translational Research Hub, Maindy Road, Cathays Cardiff, Cymru/Wales CF24 4HQ UK; ^2^ Department of Chemistry King Faisal University College of Science, P.O. Box 400 Al-Ahsa 31982 Saudi Arabia; ^3^ School of Chemistry Cardiff University Main Building, Park Place Cardiff, Cymru/Wales CF10 3AT UK

**Keywords:** Vinyl sulfides, Allylic thioethers, B(C_6_F_5_)_3_ catalysis, Csp^3^−S sulfenylation, Csp^2^−S sulfenylation

## Abstract

Precise control of selective alkene functionalization is a continuing challenge in the chemical community. In this study, we develop a substitution‐controlled regiodivergent thioetherification of di‐ or trisubstituted alkenes using 10 mol % tris(pentafluorophenyl)borane [B(C_6_F_5_)_3_] as a catalyst and *N*‐thiosuccinimide as a sulfenylating reagent. This metal‐free borane catalyzed C−S bond forming method is utilized for a Csp^2^−H sulfenylation reaction to synthesize an array of diphenylvinylsulfide derivatives with good to excellent yields (25 examples, up to 91 % yield). Some of the products exhibit aggregation‐induced emission luminogen properties used in organic light‐emitting diodes (OLEDs), chemical sensors, and biological imaging units. Depending upon the starting alkene, Csp^3^−S sulfenylation products could also be generated regioselectively. A variety of allylic thioethers from α‐alkyl substituted styrenes were isolable in good yields and selectivities (14 examples, up to 67 % yield). The DFT‐supported mechanistic study confirms that the reaction proceeds *via* a thiiranium ion intermediate, where the regioselectivity and product formation is determined by the alkene substituents which influence the activation barriers and energy profiles. Diphenylvinylsulfide derivatives can also be efficiently transformed into a range of synthetically valuable compounds, including vinyl sulfoxides, vinyl sulfones, and vinyl sulfoximines.

## Graphical abstract

This study investigates a substitution‐controlled regiodivergent thioetherification of di‐ and trisubstituted alkenes, using 10 mol % B(C_6_F_5_)_3_ as a metal‐free catalyst and *N*‐thiosuccinimide as a sulfenylating agent. The method provides efficient Csp^2^−H and Csp^3^−S sulfenylation products with high yields, along with diphenylvinylsulfides with AIE properties used for OLEDs, sensors, and imaging, and allylic thioethers. DFT analysis reveals a thiiranium‐ion‐mediated mechanism.

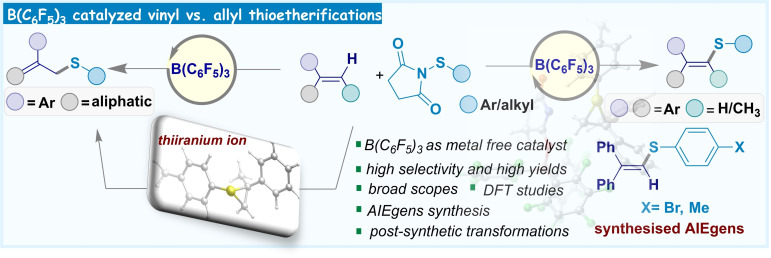

## Introduction

**Figure 1:**
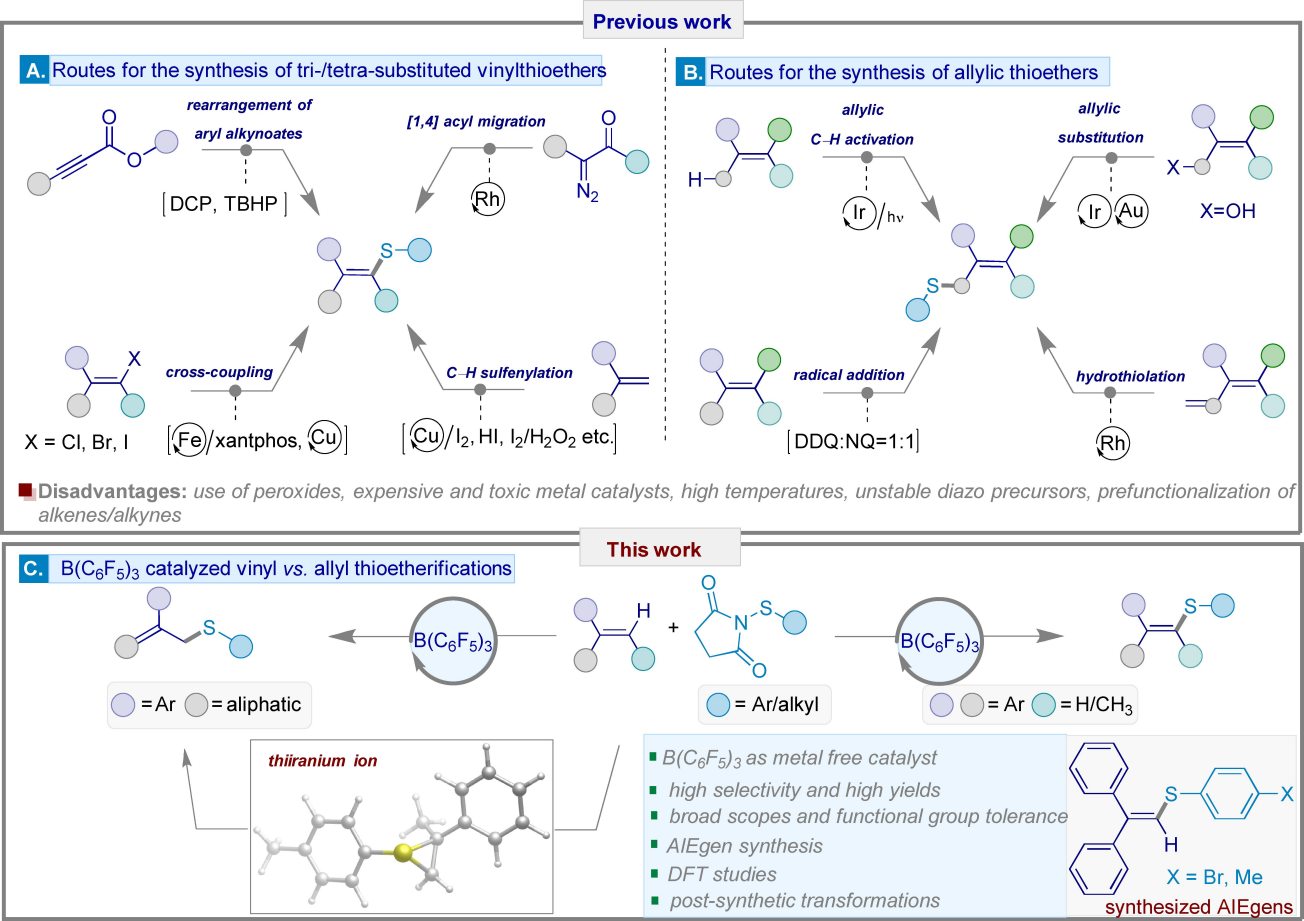
Previous synthetic routes for synthesizing tri‐ or tetrasubstituted vinyl sulfides [A] and allyl thioethers [B]. Current work on B(C_6_F_5_)_3_ catalysis for the substrate‐controlled synthesis of either vinylic or allylic thioethers [C].

The synthesis and study of organosulfur compounds containing C−S bonds is an intense research area due to their prevalence in biologically active molecules, pharmaceuticals (20 % of FDA‐approved drugs are organosulfur compounds), agrochemicals, materials science, flavors, fragrances, and food ingredients.[^1^, ^2^, ^3^, ^4^, ^5^, ^6^, ^7^, ^8^, ^9^] Indeed, due to its high polarizability, the installation of sulfur in organic molecules can improve photophysical and biological properties in organic photoelectric materials and pharmaceuticals. Vinyl sulfides exhibit large Stokes shifts significantly contributing to the frontier of research on aggregation–induced emission (AIE) and expanded the scope of new AIE luminogens (AIEgens) widely used in organic light‐emitting diodes (OLEDs), chemical sensors, biological imaging agents, and therapeutic units.^[6]^ Vinyl sulfides are also useful Michael acceptors,^[10]^ enol substitutes,^[11]^ and can be used in olefin metathesis.[^12^, ^13^] They can also act as a dienophile in Diels‐Alder reactions, and as dipolarophiles in 1,3‐dipolar cycloadditions.[^14^, ^15^] Similarly, allylic thioethers, another class of biologically relevant organosulfur compounds, are extensively exploited as sulfoxide synthons for diene syntheses in the Julia olefination reaction.^[16]^ Due to these applications, in recent decades, a plethora of synthetic strategies have been developed to synthesize substituted vinyl sulfides. Such methods include Fe‐ or Cu‐catalyzed cross‐coupling reactions of thiols and vinyl halides,[^17^, ^18^] Cu/I_2_, I_2_/H_2_O_2_, and HI‐mediated C−H sulfinylations of disubstituted alkenes,[^6^, ^19^, ^20^] rearrangements of alkynoates using *tert*‐butyl hydroperoxide (TBHP) and dicumyl peroxide (DCP),[^21^, ^22^] or Rh‐catalyzed 1,4‐acyl migrations with diazo compounds and thioesters as coupling precursors (Figure 1A).^[23]^ Early examples of allylic thioether synthesis relied on Rh‐catalyzed hydrothiolation of 1,3‐dienes^[24]^ or allenes,^[25]^ Ir‐ or Au‐catalyzed substitutions with allyl alcohols,[^26^, ^27^] allylic C−H activations by Ir photocatalysis,^[28]^ and radical addition–dehydrogenation reactions by using a 1 : 1 ratio of 2,3‐dichloro‐5,6‐dicyano‐1,4‐benzoquinone (DDQ) and 1,4‐naphthoquinone (NQ) as oxidants (Figure 1B).^[29]^ However, the existing methods have hindered the widespread implementation of these strategies in academic and industrial sectors due to several issues including the use of expensive/toxic metal salts or harmful peroxides, the requirement of prefunctionalization of alkenes/alkynes, the instability and scarcity of diazo and sulfur precursors, prolonged reaction times, high reaction temperatures, stoichiometric iodine reagents as oxidants leading to significant waste, and significantly, selectivity issues. Additionally, most of the literature reports propose reaction mechanisms without identifying specific intermediates or reaction pathways which has impeded future applications and the further development of protocols in the synthetic community. Thus, the investigation of intermediates and the development of novel methods for the stereo‐ and regiocontrolled synthesis of both vinyl sulfides and allyl sulfides in a unified reaction sequence using a sustainable reaction strategy is highly desirable.

In recent years, organocatalysts have been used as a sustainable strategy for obtaining organic scaffolds in a highly selective manner.^[30]^ Relating to this, borane catalysis has also advanced the field of metal‐free catalysis owing to its relatively high abundance, low toxicity, and potential for alternate or excellent selectivity.[^31^, ^32^, ^33^, ^34^] We and other groups have utilized tris(pentafluorophenyl)borane [B(C_6_F_5_)_3_] as a metal‐free catalyst and a Lewis acidic component of frustrated Lewis pair (FLP) chemistry for addressing a diverse range of C−C and C−X bond‐forming transformations.[^35^, ^36^, ^37^, ^38^]

Recently, we described the B(C_6_F_5_)_3_‐catalyzed chemo‐ and regioselective Csp^2^−H thioetherifications of various commercially‐available arenes and heteroarenes with thiosuccinimides. They proceeded through elongation of the thiosuccinimide N−S bond assisted by boron adduct formation. This forms a stable ion pair leading to thioarylated products with high chemo‐ and regioselectivity.^[39]^ Inspired by this we postulated that B(C_6_F_5_)_3_ can catalyze the substrate‐controlled thioetherification of di‐ or trisubstituted alkenes using thiosuccinimide as a sulfenylating agent. This would enable a metal‐free access to tri‐ or tetra‐substituted vinyl sulfides or allyl thioethers (Figure 1C).^[40]^

## Results and Discussion

### Reaction Development

**Figure 2:**
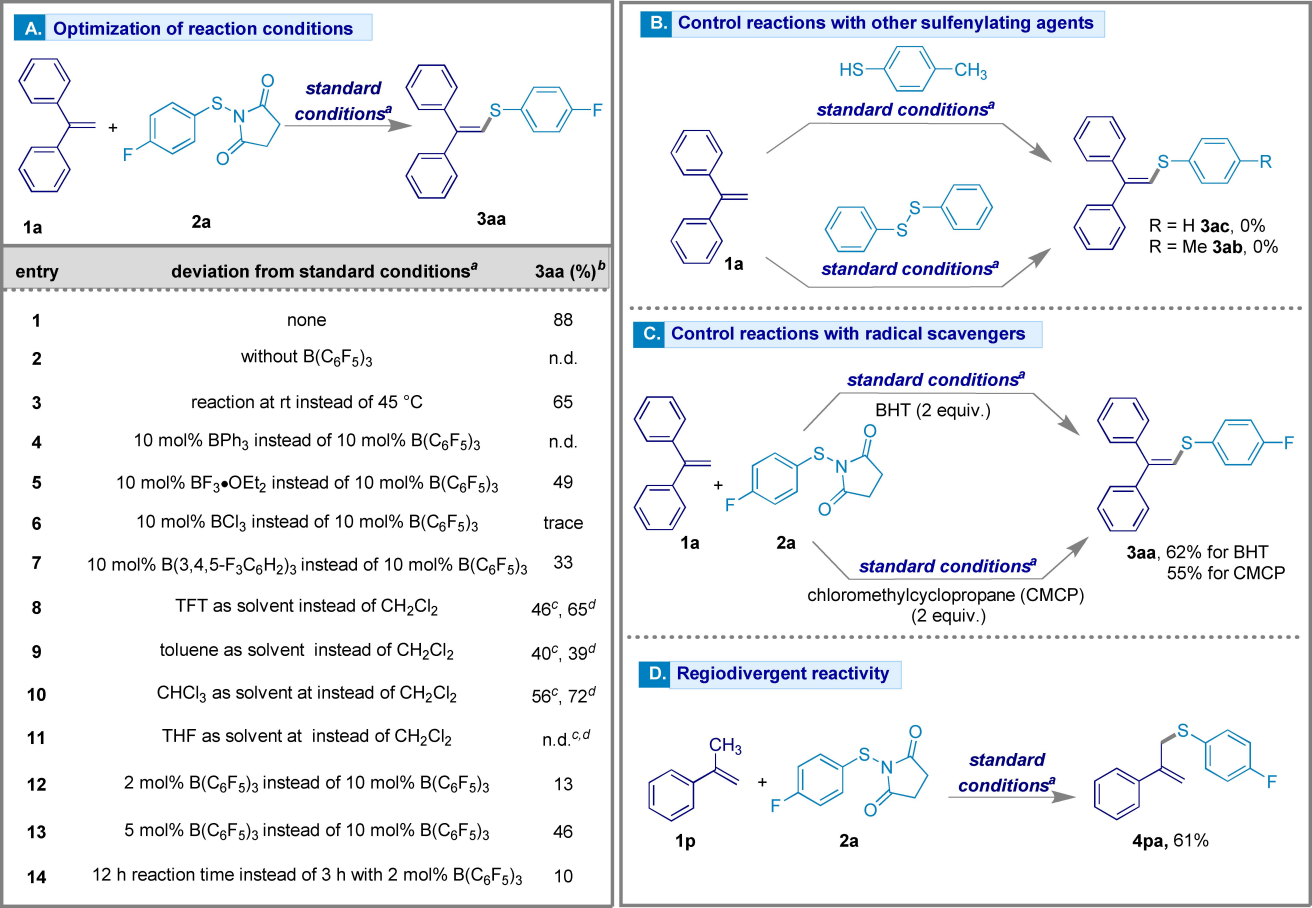
Reaction development showing the optimization of the reaction conditions [A], control experiments with other sulfenylating agents [B] and radical scavengers [C], and regiodivergent reactivity with alkene **1 p** (14 mg, 0.12 mmol, 1.2 equiv.) [D]. ^*a*^ Standard conditions: **1 a** (22 mg, 0.12 mmol, 1.2 equiv.), **2 a** (23 mg, 0.1 mmol, 1.0 equiv.) in 0.1 M CH_2_Cl_2_ solvent at 45 °C for 3 h; ^*b*^ Isolated yields; ^*c*^ Reaction performed at room temperature; ^*d*^ Reaction performed under reflux.

Initially we investigated the synthesis of the trisubstituted vinyl sulfide **3 aa** from commercially available 1,1‐diphenylethylene (**1 a**) as the Csp^2^−H coupling partner and thiosuccinimide **2 a** (Figure 2A). By applying our previous hypothesis for the activation of thiosuccinimides catalyzed by B(C_6_F_5_)_3_,^[39]^ we obtained sulfenylated product **3 aa** in 88 % yield after 3 h at 45 °C in dichloromethane when using 10 mol % B(C_6_F_5_)_3_ as a catalyst (Figure 2A, entry 1). To ensure that B(C_6_F_5_)_3_ is essential to generate the electrophilic sulfur center from the thiosuccinimide through N−S bond activation, we heated a mixture of **1 a** and **2 a** in dichloromethane without catalyst. No product was observed (Figure 2A, entry 2). Lowering the temperature to room temperature in a reaction with 10 mol % B(C_6_F_5_)_3_ reduced the yield of the product **3 aa** to 65 % (Figure 2A, entry 3). Screening other borane Lewis acids including BPh_3_, BF_3_⋅OEt_2_, BCl_3_ and B(3,4,5‐F_3_C_6_H_2_)_3_ gave lower yields than B(C_6_F_5_)_3_ (Figure 2A, entries 4–7)_._ The examination of solvents such as trifluorotoluene (TFT), toluene, and CHCl_3_ at room temperature and reflux showed that the solvents TFT and CHCl_3_ yielded **3 aa** with 46 % and 56 % at room temperature, improving to 65 % and 72 % at reflux. On the other hand, when using toluene as the solvent, little change in the yield was observed giving 40 % and 39 % yield of **3 aa** respectively. THF failed to produce **3 aa** at either room temperature or under reflux (Figure 2A, entries 8–11). The efficacy of the catalyst was also evaluated with loadings of 2 mol % and 5 mol %, resulting in yields of 13 % and 46 % of **3 aa**, respectively. Even with an extended reaction time there was little improvement in the yield when employing 2 mol % B(C_6_F_5_)_3_ (Figure 2A, entries 12–14). Other commercially available sulfenylating precursors were also considered (Figure 2B). The employment of 4‐methylbenzene thiol instead of **2 a** as a dehydrogenating C−S coupling partner with **1 a** under the standard reaction conditions did not yield the desired product. This could indicate that coordination of the succinimide containing sulfur precursor with boron through a dative adduct is a requirement for this C−S bond forming reaction. Likewise, attempts using diphenyl disulfide as a sulfenyl source also failed to facilitate the sulfenylation reaction, thus ruling out the possibility of a borane‐assisted S−S bond cleavage. We also investigated if this reaction was proceeding through a radical pathway by using butylated hydroxytoluene (BHT) and chloromethylcyclopropane (CMCP) as radical scavengers under the standard conditions. Addition of these scavengers did not affect the yields (62 % and 55 % yields, respectively) indicating a diamagnetic pathway was operative (Figure 2C). Interestingly, when using α‐methylstyrene (**1 p**) as a starting material, the formation of a Csp^3^−S sulfenylation product (**4 pa**, 61 % yield) was observed rather than the Csp^2^−S product (Figure 2D).

### DFT Studies

**Figure 3:**
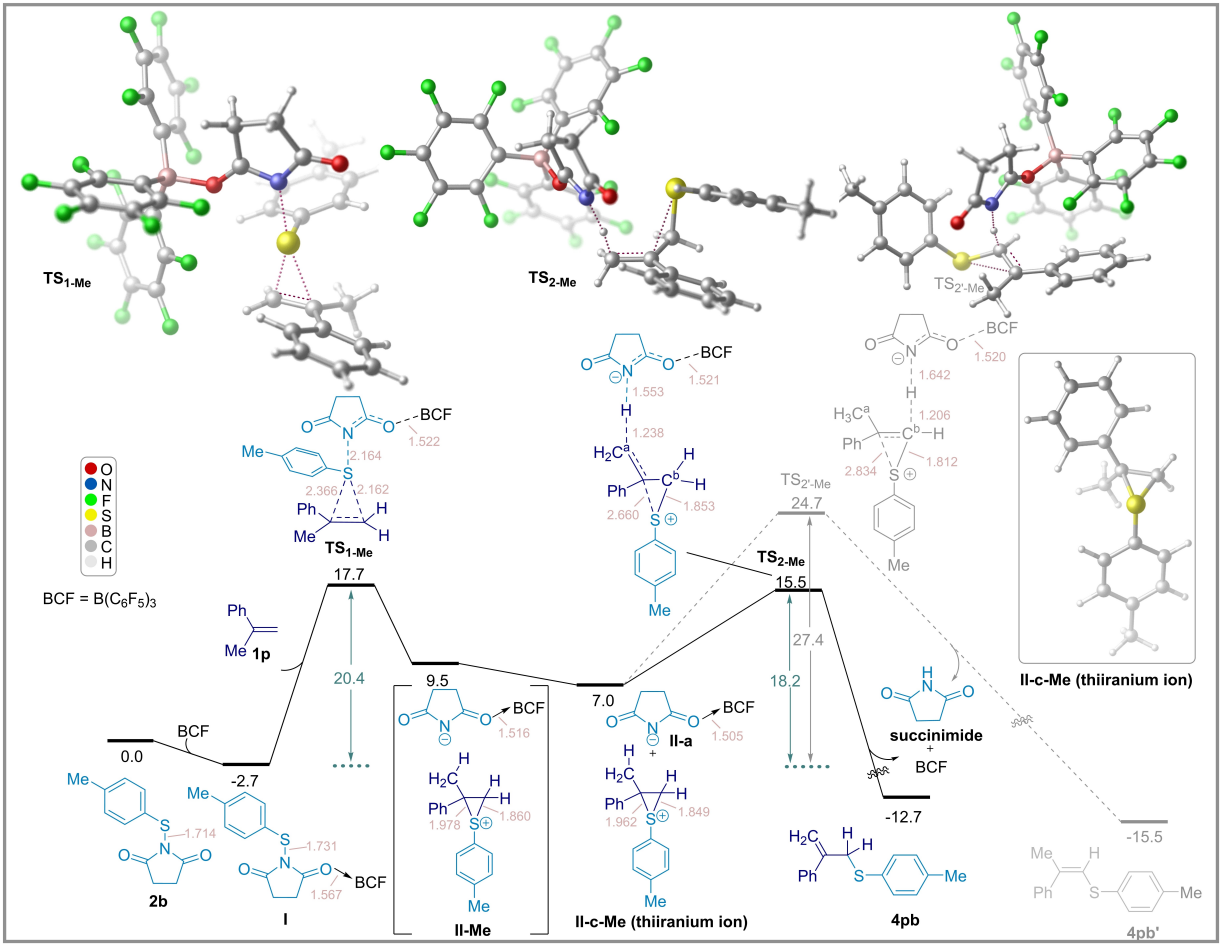
Energy profile for the formation of Csp^2^−S and Csp^3^−S products (**4 pb** and **4 pb′)** at the SMD/M06‐2X/def2‐TZVP//SMD/M06‐2X/6‐31G(d) level of theory using *α*‐methylstyrene **1 p**. Bond lengths (light pink color) shown in Å. Relative energies are given in kcal mol^−1^.

To investigate the mechanism of the thioetherification reaction, we performed DFT calculations at the SMD/M06‐2X/def2‐TZVP//SMD/M06‐2X/6‐31G(d) level of theory in dichloromethane as the solvent. Firstly, we explored the mechanism for the reaction between α‐methylstyrene (**1 p**) and thiosuccinimide **2 b** as shown in Figure 3 to further understand the differing reaction pathways to generate the Csp^2^−S or Csp^3^−S sulfenylation products. The DFT calculations indicate that the initial step of the reaction is the coordination of B(C_6_F_5_)_3_ to one of the carbonyl oxygen atoms of the thiosuccinimide, leading to an elongation of the N−S bond forming adduct **I**. The subsequent reaction of the π‐bond of alkene **1 p** with adduct **I** occurs through a 3‐membered transition state (**TS_1‐Me_**), which leads to the ion pair **II–Me** consisting of a thiiranium cation^[40]^ and succinimide anion. The separated succinimide anion and thiiranium cation were found to be slightly more stable by 2.5 kcal mol^−1^ (**II‐a** and **II‐c‐Me**). From this point, there are two possible pathways for deprotonation, namely from either C^a^ or C^b^, along with ring opening of the thiiranium species with the aid of succinimide anion **II‐a**. The calculations indicate that deprotonation from the C^a^ *via* the transition state **TS_2‐Me_** requires an activation barrier of 18.2 kcal mol^−1^. On the other hand, deprotonation from C^b^ through **TS_2′‐Me_** requires a higher activation energy of 27.4 kcal mol^−1^, which is kinetically not feasible at the reaction temperature of 45 °C. This implies that the Csp^3^−S sulfenylation product **4 pb** is more favorably formed than the Csp^2^−S sulfenylation product **4 pb’** when using *α*‐methylstyrene (**1 p**). As illustrated in Figure 3, the formation of **4 pb**, the release of succinimide, and the regeneration of the B(C_6_F_5_)_3_ catalyst occurs exothermically with a reaction energy of −12.7 kcal mol^−1^.

**Figure 4:**
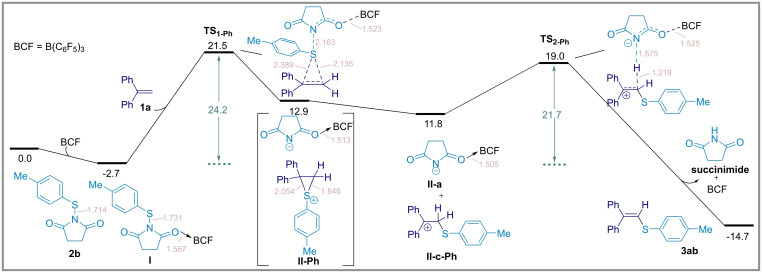
Energy profile for the formation of the Csp^2^−S product **3 ab** at the SMD/M06‐2X/def2‐TZVP//SMD/M06‐2X/6‐31G(d) level of theory using 1,1‐diphenylethylene (**1 a**) as the alkene coupling partner. Bond lengths (light pink color) are shown in Å. Relative energies are given in kcal mol^−1^.

Next, the methyl group was exchanged with a phenyl group on the alkene coupling partner and the pathway for the formation of product **3ab** from 1,1‐diphenylethylene (**1 a**) and thiosuccinimide **2 b** was calculated. As indicated in Figure 4, a similar reactivity for the formation of thiiranium cation *via* **TS_1‐ph_** with an activation barrier of 24.2 kcal mol^−1^ was found. This slightly higher activation barrier is justified with the reflux temperature (45 °C for dichloromethane) required for the reaction. Comparing the energies of ion pairs **II–Me** (9.5 kcal mol^−1^) and **II‐Ph** (12.9 kcal mol^−1^) (as shown in Figures 3 and 4), the higher reactivity of **II‐Ph** results in direct ring opening and the formation of the separated cation **II‐c‐Ph** after optimization. Deprotonation then takes place with an activation barrier of 21.7 kcal mol^−1^ (**TS_2−Ph_**), leading to the final product **3 ab**, which is produced with a reaction energy of −14.7 kcal mol^−1^. Of note, we have isolated only succinimide as the by‐product at the end of the reaction, indicating that B(C₆F₅)₃ is likely regenerated. This conclusion is supported by our calculations as indicated in the last step of Figures 3 and 4.

### Reaction Scope

**Figure 5:**
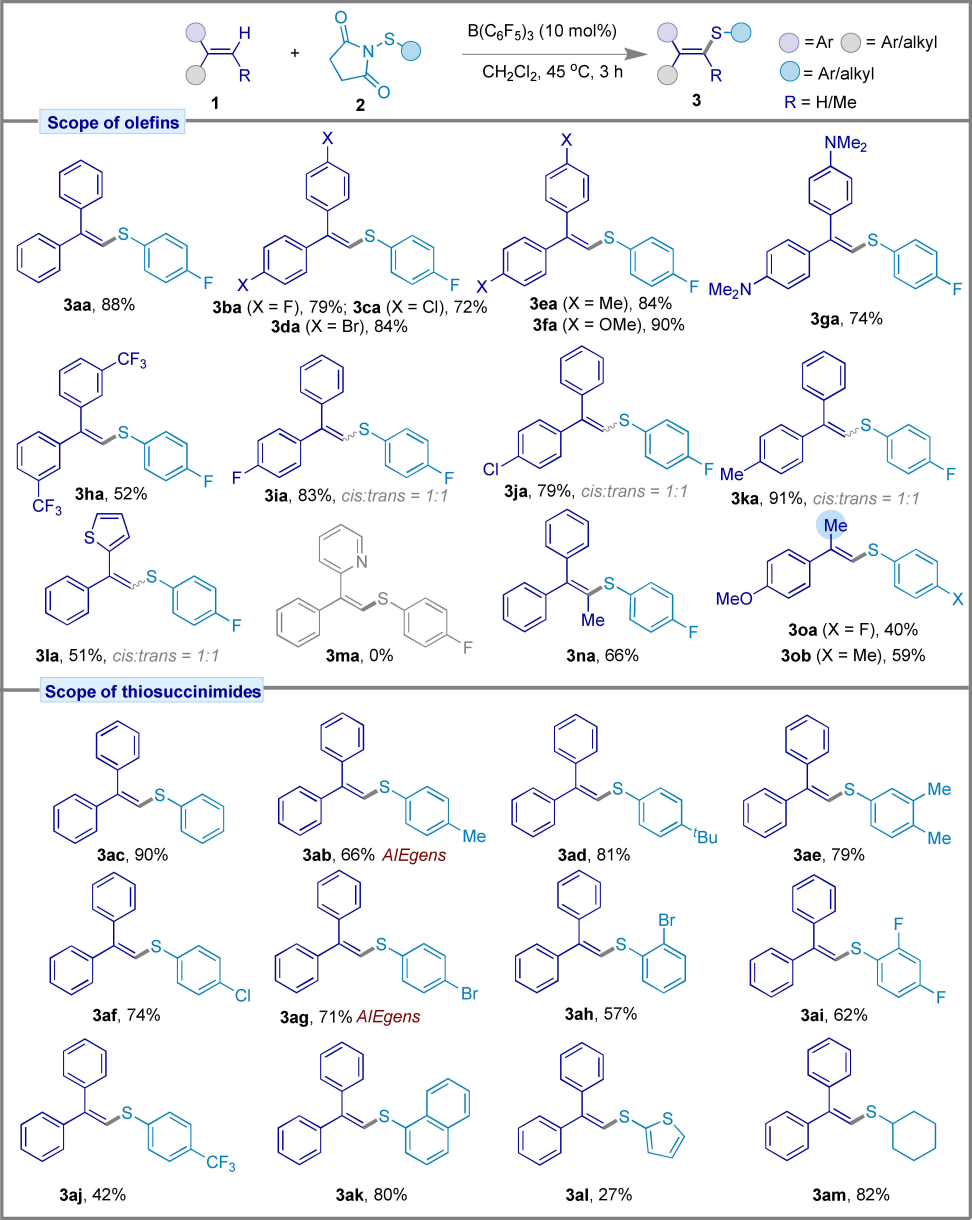
Reaction scope for the formation of vinyl sulfides. All reactions were performed on a 0.1 mmol scale using the optimized reaction conditions.

Equipped with the optimized reaction conditions and detailed mechanistic study, we sought to generalize the protocol for various di‐ and trisubstituted ethylene derivatives with **2 a** as the sulfenyl source (Figure 5). Symmetrical 1,1‐disubstituted alkenes with electron‐withdrawing or electron‐donating groups (−F, −Cl, −Br, −Me, −OMe, −NMe_2_) attached to the aryl rings were converted to the corresponding vinyl sulfides in good to excellent yields (**3 aa**–**3 ga**, 72–90 %). Compound **3 ha** was isolated in 52 % yield when 3,3′‐(ethene‐1,1‐diyl)bis((trifluoromethyl)benzene) was used as an alkene partner. However, when using unsymmetrical 1,1‐disubstituted alkenes, a 1 : 1 ratio of *cis* and *trans* isomers of the vinyl sulfide products **3 ia**–**3 la** was formed in 51–91 % yields. However, as anticipated with the limitation of borane catalysis, the C−S coupling product **3 ma** could not be obtained due to the coordination of borane with the pyridyl nitrogen which prevented the B(C₆F₅)₃‐succinimide adduct process required for the reaction initiation. Furthermore, the trisubstituted alkene **1 n** was transformed selectively into vinyl sulfide **3 na**, a tetrasubstituted alkene, under the optimized reaction conditions. In contrast to our observation of the Csp^3^−S sulfenylation product (**4 pb**) generated with *α*‐methylstyrene (**1 p**), 4‐isopropenylanisole **1 o** having a *para*‐OMe functionality on the phenyl group, selectively delivered the Csp^2^−S sulfenylation products **3 oa** (40 %) and **3 ob** (59 %). We performed the calculations to understand the reverse selectivity on 4‐isopropenylanisole. The transition state energies for the deprotonation step, identified as the selectivity‐determining step, were found to be closely comparable, with only a 0.1 kcal/mol difference between **TS_2−Me−OMe−F_** and **TS_2′−Me−OMe−F_**. However, the stability of the product **3 oa** is notably greater, attributed to the enhanced resonance contribution of the OMe group as compared to **3oa′**, providing a 7.0 kcal energy advantage over **3oa′**. This indicates that while the transition state energies are kinetically comparable, **3 oa** is thermodynamically the more favorable product (see SI, Scheme S1). We also investigated the scope of thiosuccinimides **2** to include aromatic, heteroaromatic, and aliphatic thiosuccinimides, including those which might have potential applications as AIEgens. The vinyl sulfides (**3 ac–3 ae**) formed from electron‐rich thiosuccinimides produced higher yields (66–90 %) relative to vinyl sulfides **3 af**–**3 aj** (42–74 % yield) formed from electron‐withdrawing halogen‐substituted thiosuccinimides. Interestingly, diphenyl vinyl sulfide derivatives **3 ab** and **3 ag** are AIEgens used in biological imaging, and various luminogenic smart materials such as OLEDs.^[6]^ The polyaromatic sulfenyl partner 1‐(naphthalen‐1‐ylthio)pyrrolidine‐2,5‐dione (**2 k**) gave 80 % of the Csp^2^−S coupled product (**3 ak**). However, the regioselective sulfenylation reaction proceeded sluggishly with the heteroaromatic sulfenyl partner 1‐(cyclohexylthio)pyrrolidine‐2,5‐dione (**2 l**), yielding only 27 % of **3 al** as the C−S coupled product. To our delight, the aliphatic sulfenyl partner 1‐(thiophen‐2‐ylthio)pyrrolidine‐2,5‐dione (**2 m**) demonstrated significant reactivity, yielding 82 % of vinyl sulfide **3 am**.

**Figure 6:**
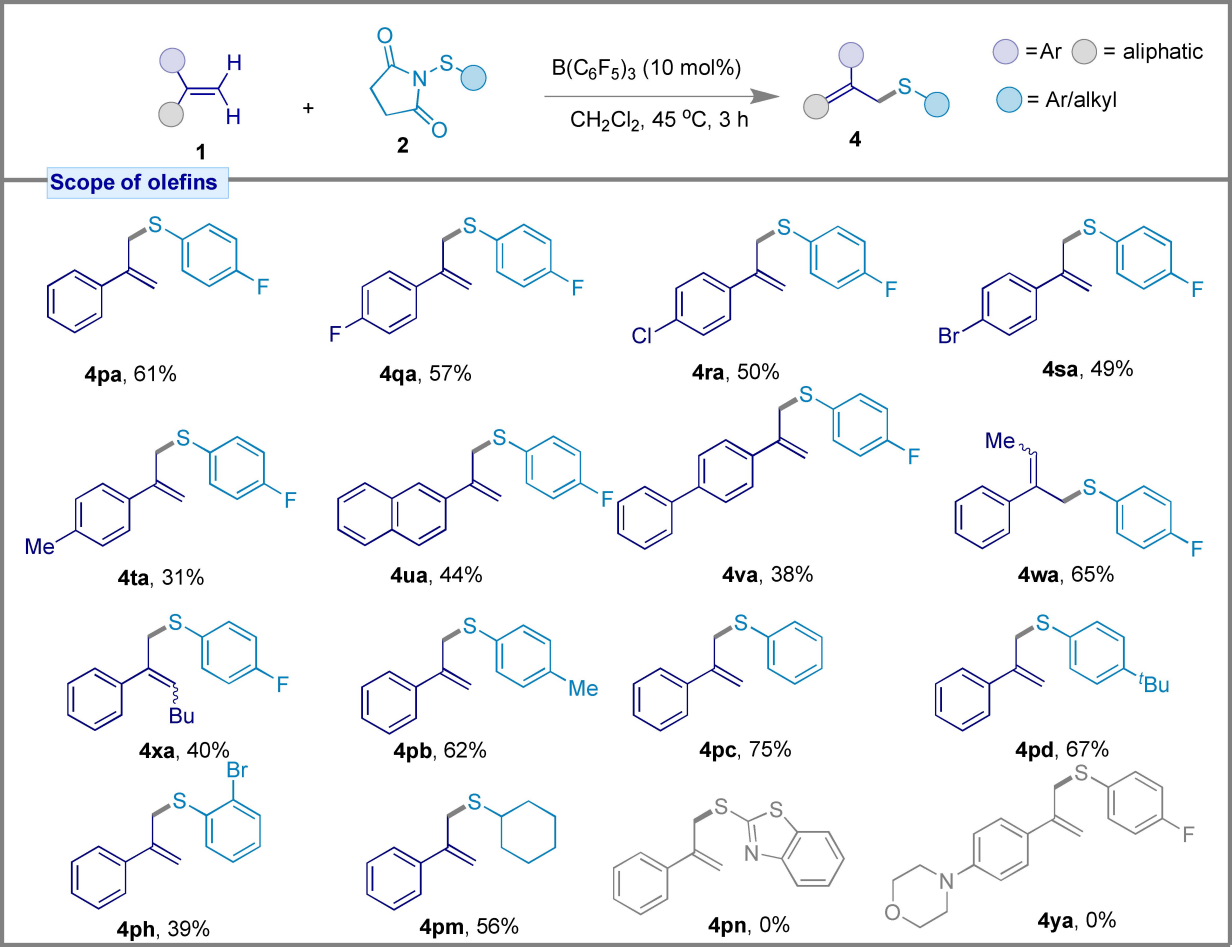
Reaction scope for the formation of allylic thioethers. All reactions were performed on a 0.1 mmol scale using the optimized reaction conditions.

Using our optimized reaction conditions, we could also generate allylic thioethers selectivity when using alkyl substituted styrenes. As shown in Figure 6, *α‐*methylstyrenes bearing *para*‐substituted halogens, alkyl, or aryl groups attached to the phenyl ring (**1 p**–**t**, **1 v**), as well as 2‐(prop‐2‐yn‐1‐yl)naphthalene (**1 u**) were amenable to the reaction producing the corresponding allylic thioethers (**4 pa**–**4 va**) selectively. Importantly no Csp^2^−S sulfenylation product was formed during these reactions. Notably the lower reaction yields were likely attributable to the use of an electron‐withdrawing sulfenyl partner *p*‐fluorophenyl thiosuccinimide (**2 a**), which destabilizes the thiiranium cation intermediate. Other α‐alkylstyrene derivatives were also tolerant towards the sulfenylation reaction to afford allyl sulfides **4 wa** and **4 xa** with 65 % and 40 % yields, respectively. We also examined the effect of electron‐donating sulfenyl partners **2** which demonstrated better yields for the formation of **4 pc**–**4 pd** (62–75 % yield), and also the *ortho*‐bromo substituted thiosuccinimide (**2 h**) which yielded **4 ph** in 39 % yield. Allylic thioether **4 pm** was also generated in 56 % yield for the aliphatic sulfenyl coupling partner **2 m**. Limitations of the reaction were observed with benzo[*d*]thiazole‐2‐thio succinimide (**1 q**) with *α*‐methylstyrene (**2 m**), and also for 4‐(4‐(prop‐1‐en‐2‐yl)phenyl)morpholine (**1 y**) with thiosuccinimide (**2 a**).

### Post Synthetic Modification

**Figure 7:**
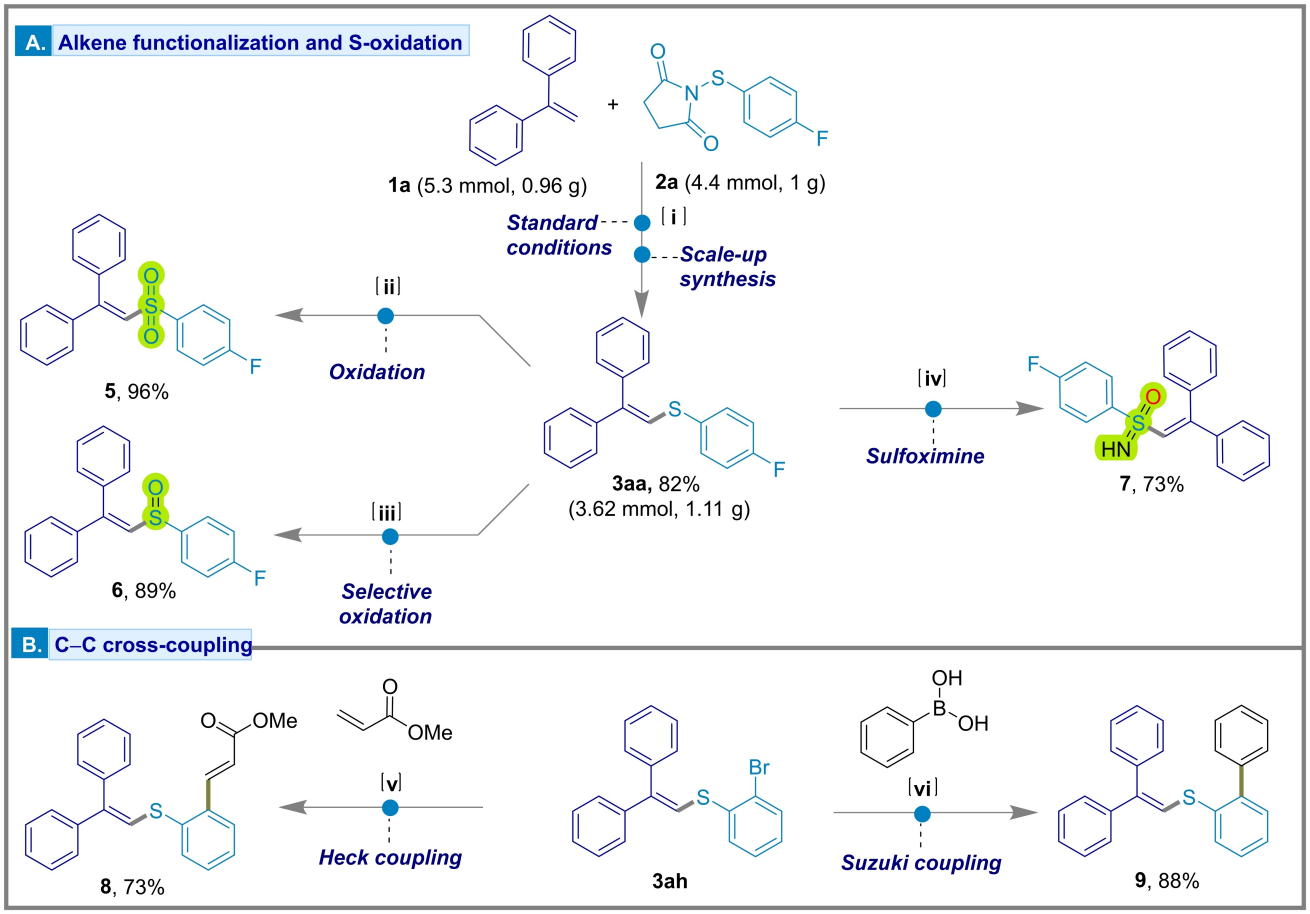
Post synthetic modifications of diphenyl vinyl sulfides: [A] alkene functionalization and *S*‐centered oxidations, [i] scale‐up synthesis of **3 aa**; [ii] synthesis of vinyl sulfone **5** using 3.1 equiv. *m*CPBA in CH_2_Cl_2_ at −78 °C then 40 °C for 16 h; [iii] synthesis of vinyl sulfoxide **6** using 1.1 equiv. *m*CPBA in CH_2_Cl_2_ at −78 °C then rt for 3 h; [iv] synthesis of sulfoximine **7** using 2.5 equiv. PhI(OAc)_2_ and 2 equiv. NH_2_COONH_4_ in MeOH at rt for 3 h; [B] C−C cross‐coupling, [v] synthesis of compound **8** using 5 mol % Pd(PPh_3_)_2_Cl_2_, 3 equiv. DIPEA and 1.2 equiv. methyl acrylate in DMF at 120 °C for 48 h; [vi] synthesis of compound **9** using 5 mol % Pd(PPh_3_)_2_Cl_2_, 3 equiv. K_2_CO_3_ and 1.2 equiv. of phenylboronic acid in 3 : 1 dioxane and water at 100 °C for 24 h.

The C−S coupled products obtained with the metal‐free Csp^2^−H sulfenylation protocol were further utilized for various post‐synthetic transformations (Figure 7). Prior to this, the synthesis of **3 aa** was scaled up to give the product in 82 % yield (3.62 mmol, 1.11 g). Oxidation of sulfur was carried out using *m*CPBA as the oxidant, yielding the synthetically valuable vinyl sulfone **5** in high yields (96 %). By limiting the amount of *m*CPBA used, overoxidation of sulfur could be prevented leading selectively to the vinyl sulfoxide **6** with 89 % yield. Furthermore, diphenyl vinyl sulfide **3 aa** was converted to the sulfoximine (**7**, 73 %) by reaction with (diacetoxyiodo)benzene and ammonium carbamate (H_2_NCO_2_NH_4_). This sulfoximine with a free −NH group allows potential for access to other synthetically useful products by *N*‐functionalization.^[41]^ The implementation of Heck and Suzuki reactions with compound **3 ah** provided access to additional C−C coupling reactions on the sulfenyl backbone of diphenyl vinyl sulfide, forming compound **8** with 88 % and compound **9** with 73 % yield.

## Conclusions

In summary, we have developed a metal‐free borane‐catalyzed regiodivergent synthesis of either vinyl thioethers or allyl thioethers depending on the substituents on the alkene starting material. This protocol offers the synthesis of the vinyl sulfides derivatives including those having AIEgen properties allowing a new method for the synthesis of aggregation induced emission based materials. DFT studies into the reaction mechanism explained the selectivity in forming the Csp^2^−S or Csp^3^−S sulfenylation products. A thiiranium intermediate was proposed to be key in the reaction mechanism. We believe that this regio‐divergent thioethification reaction catalyzed by a borane will further stimulate the research area of metal‐free main group catalysis in designing stereo‐ and regioselective organic transformations.

## Conflict of Interests

The authors declare no conflict of interest.

1

## Supporting information

As a service to our authors and readers, this journal provides supporting information supplied by the authors. Such materials are peer reviewed and may be re‐organized for online delivery, but are not copy‐edited or typeset. Technical support issues arising from supporting information (other than missing files) should be addressed to the authors.

Supporting Information

## Data Availability

Information about the data that underpins the results presented in this article, including how to access them, can be found at https://doi.org/10.17035/cardiff.28016297.
