# Supplementary material for: B(C6F5)3‐Catalyzed Regiodivergent Thioetherifications of Alkenes via Thiiranium Intermediates: Experimental and Computational Insights
Source: Chemistry. 2024 Dec 23;31(10):e202404236. doi: 10.1002/chem.202404236 (PMC11833220; doi:10.1002/chem.202404236)
Supplement: Supplementary file 1 — Supporting Information [file CHEM-31-e202404236-s001.pdf]

Chemistry–A European Journal

Supporting Information

$B(C_6F_5)_3$-Catalyzed Regiodivergent Thioetherifications of Alkenes *via* Thiiranium Intermediates: Experimental and Computational Insights

Nusaybah Alotaibi, Rasool Babaahmadi, Sampurna Das, Emma Richards, Thomas Wirth, Milan Pramanik,* and Rebecca L. Melen*

Supporting Information for

B(C_6F_5)$_3$-Catalyzed Regiodivergent Thioetherifications of Alkenes *via* Thiiranium Intermediates: Experimental and Computational Insights

Nusaybah Alotaibi,†[a,b] Rasool Babaahmadi, †[a] Sampurna Das,[a] Emma Richards,[a,c] Thomas Wirth,[c] Milan Pramanik,[a] and Rebecca L. Melen*[a]*

[a] Ms. Nusaybah Alotaibi, Dr. Rasool Babaahmadi, Dr. Milan Pramanik, Ms. Sampurna Das, Prof. Dr. Rebecca L. Melen

Cardiff Catalysis Institute, School of Chemistry, Cardiff University, Translational Research Hub, Maindy Road, Cathays, Cardiff, CF24 4HQ, Cymru/Wales, UK. Email: pramanikm@cardiff.ac.uk, MelenR@cardiff.ac.uk.

[b] Ms. Nusaybah Alotaibi

Department of Chemistry, King Faisal University, College of Science, P.O. Box 400, AlAhsa 31982, Saudi Arabia.

[c] Dr. Emma Richards, Prof. Dr. Thomas Wirth

School of Chemistry, Cardiff University, Main Building, Park Place, Cardiff, CF10 3AT, Cymru/Wales, UK.

† Equal contribution

Table of contents

1. Experimental details	S3
2. Synthesis and spectral characterization	S3–S35
3. Control experiments	S35–S36
4. Computational details	S36–S82
5. NMR spectra	S83–S247
6. References	S248–S250

1. Experimental

1.1 General experimental

All reactions and manipulations were carried out under an atmosphere of dry, O_2-free nitrogen using standard double-manifold techniques with a rotary oil pump. A nitrogen-filled glove box (MBraun) was used to manipulate solids including the storage of starting materials, ambient temperature reactions, product recovery and sample preparation for analysis. Solvents were dried by employing a solvent purification system MB SPS-800 and stored under a nitrogen atmosphere. Anhydrous (with Sure/Seal^{TM}) 1,2-dichloroethane (1,2-$C_2H_4Cl_2$) was purchased from Merck and dried over molecular sieves before use. Chemicals were purchased from commercial suppliers and used as received. All the triarylfluoroboranes were prepared as per the standard literature report.[1] Thin-layer chromatography (TLC) was performed on pre-coated aluminum sheets of Merck silica gel 60 F254 (0.20 mm). ^{1}H, ^{13}C and ^{19}F NMR spectra were recorded on a Bruker Avance II 400 or Bruker Avance 500 spectrometer. All coupling constants are absolute values and are expressed in Hertz (Hz). ^{13}C NMR spectra were measured as ^{1}H decoupled. Yields are given as isolated yields. Chemical shifts are expressed as parts per million (ppm, δ) downfield of tetramethylsilane (TMS) and are referenced to $CDCl_3$ (7.26/77.16 ppm) as internal standard. The description of signals includes s = singlet, d = doublet, t = triplet, q = quartet, p = pentet and m = multiplet. All coupling constants are absolute values and are expressed in Hertz (Hz). All spectra were analysed assuming a first order approximation. IR-spectra were measured on a Shimadzu IRAffinity-1 photo-spectrometer. Mass spectra were measured on a Waters LCT Premier/XE or a Waters GCT Premier spectrometer. Ions were generated by the Atmospheric Solids, Analysis Probe (ASAP), Electrospray (ES) or Electron Ionization (EI). The molecular ion peaks values quoted for either molecular ion (M^+), molecular ion plus or minus hydrogen ($M+H^+$, $M-H^-$), molecular ion plus sodium ($M+Na^+$).

2. Synthesis and characterization

2.1 Synthesis of alkenes

General procedure a: All the alkenes **1b–y**, except **1a**, **1p** and **1q** which were purchased from commercial suppliers and used as received, were prepared using literature procedures.[4] To a stirred suspension of alkyltriphenylphoshonium bromide (9.6 mmol, 2 equiv.) in dry THF (25

mL), *t*-BuOK (0.97 g, 8.64 mmol, 1.8 equiv.) was added under a nitrogen atmosphere. After 30 min, the corresponding ketone (4.8 mmol, 1 equiv.) was added into the flask and the mixture was stirred overnight at room temperature. The resulting reaction mixture was poured into 50 mL of water and subsequently extracted three times with diethyl ether (30 mL × 3). The combined organic phases were dried over anhydrous $MgSO_4$ and the solvent evaporated under reduced pressure, giving the crude product, which was purified via column chromatography using silica gel and hexane/ethyl acetate (30:1) as eluent.

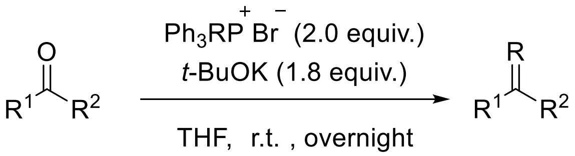

2.2 Synthesis of *N*-thiosuccinimides

General procedure b: All the *N*-thiosuccinimide compounds **2a–o** were prepared using literature procedures.[2] To a stirred solution of the corresponding thiol (7.53 mmol, 1 equiv.) in 20 mL of toluene, *N*-chlorosuccinimide (1.01 g, 7.53 mmol, 1 equiv.) was added to the reaction mixture at room temperature under a nitrogen atmosphere. After one hour, triethylamine (1.05 mL, 7.53 mmol, 1 equiv.) in dry toluene (10 mL) was added dropwise over a period of 30 minutes to the reaction mixture. The mixture was allowed to stir at 40 °C overnight. The reaction was diluted by addition of 60 mL of diethyl ether. The resulting white precipitate was filtered. The filtrate was concentrated to afford the crude product. The pure compound was obtained after column chromatography using ethyl acetate and hexane (30:70) as eluent. The NMR spectroscopic data are in agreements with the prepared compounds.[3]

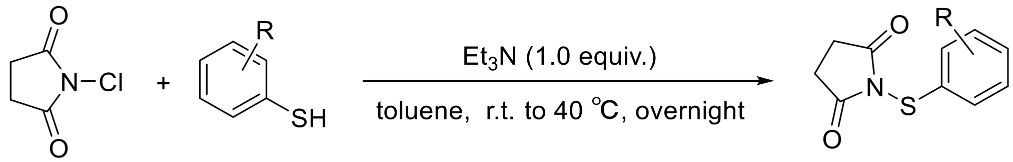

2.3 Synthesis of thiolated alkenes

General procedure c: Inside the glovebox, three glass microwave vials were charged separately with the alkene **1** (1.2 equiv.), thiosuccinimide **2** (1.0 equiv.) and $B(C_6F_5)_3$ (10 mol%) and then capped with a septum. The three vials were brought outside the glovebox and 0.3 mL of dry CH_2Cl_2 was added to each vial using a syringe. The thiosuccinimide solution was added to the $B(C_6F_5)_3$ solution. The mixture was added to the alkene solution dropwise under vigorous stirring

at room temperature. The reaction mixture was left at an optimum temperature 45 °C under continuous stirring. After 3 h, all volatiles were removed in vacuo and the crude compound was purified via preparative thin layer chromatography using hexane/ethyl acetate as eluent.

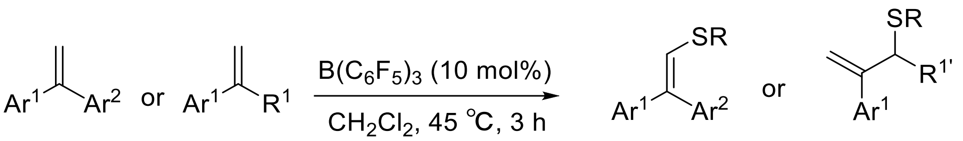

2.4 Synthesis and spectral characterization of alkenes starting materials

Synthesis of 4,4'-(ethene-1,1-diyl)bis(fluorobenzene) **1b**[5]

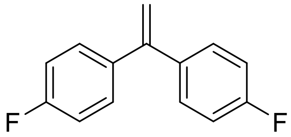

Synthesized in accordance with General Procedure a using 4,4′-difluorobenzophenone (1.05 g, 4.8 mmol, 1 equiv.) and methyltriphenylphoshonium bromide (3.43 g, 9.6 mmol, 2 equiv.). The crude product was purified via column chromatography using hexane/ethyl acetate (30:1 v/v) as eluent. The desired compound **1b** was obtained as a colorless liquid. Yield: 0.905 g, 4.2 mmol, 87%. ^{1}H NMR (400 MHz, $CDCl_3$, 298 K) δ: 7.34–7.27 (m, 4H, Ar–CH), 7.07–7.00 (m, 4H, Ar–CH), 5.41 (s, 2H, vinylic); ^{13}C NMR (101 MHz, $CDCl_3$, 298 K) δ: 162.7 (d, $J_{C–F}$ = 247.0 Hz), 148.2, 137.5 (d, $J_{C–F}$ = 3.3 Hz), 130.0 (d, $J_{C–F}$ = 8.0 Hz), 115.3 (d, $J_{C–F}$ = 21.4 Hz), 114.3 (CH_2); ^{19}F NMR (376 MHz, $CDCl_3$, 298 K) δ: -114.4. Data agrees with literature values.[5]

Synthesis of 4,4'-(ethene-1,1-diyl)bis(chlorobenzene) **1c**[5]

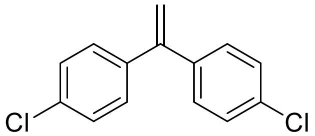

Synthesized in accordance with General Procedure a using 4,4’-dichlorobenzophenone (1.21 g, 4.8 mmol, 1 equiv.) and methyltriphenylphoshonium bromide (3.43 g, 9.6 mmol, 2 equiv.). The crude product was purified via column chromatography using hexane/ethyl acetate (30:1 v/v) as eluent. The desired compound **1c** was obtained as a white solid. Yield: 0.832 g, 3.2 mmol, 67%. ^{1}H NMR (400 MHz, $CDCl_3$, 298 K) δ: 7.36–7.31 (m, 4H, Ar–CH), 7.29–7.24 (m, 4H, Ar–CH), 5.48 (s, 2H, vinylic); ^{13}C NMR (101 MHz, $CDCl_3$, 298 K) δ: 148.1, 139.6, 134.0, 129.6, 128.6, 115.2 (CH_2); Melting point: 80–82 °C. NMR data agrees with literature values.[5]

Synthesis of 4,4'-(ethene-1,1-diyl)bis(bromobenzene) **1d**[5]

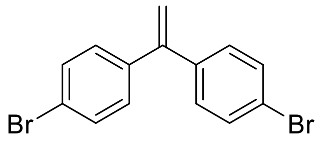

Synthesized in accordance with General Procedure a using 4,4′-dibromobenzophenone (1.63 g, 4.8 mmol, 1 equiv.) and methyltriphenylphoshonium bromide (3.43 g, 9.6 mmol, 2 equiv.). The crude product was purified via column chromatography using hexane/ethyl acetate (30:1 v/v) as eluent. The desired compound **1d** was obtained as a white solid. Yield: 0.998 g, 3 mmol, 62%. ^{1}H NMR (400 MHz, $CDCl_3$, 298 K) δ: 7.50–7.42 (m, 4H, Ar–CH), 7.22–7.14 (m, 4H, Ar–CH), 5.46 (s, 2H, vinylic); ^{13}C NMR (101 MHz, $CDCl_3$, 298 K) δ: 148.2, 140.0, 131.6, 130.0, 122.2, 115.3 (CH_2); Melting point: 70–73 °C. NMR data agrees with literature values.[5]

Synthesis of 4,4'-(ethene-1,1-diyl)bis(methylbenzene) **1e**[5]

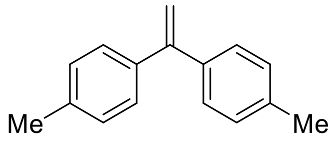

Synthesized in accordance with General Procedure a using 4,4′-dimethylbenzophenone (1 g, 4.8 mmol, 1 equiv.) and methyltriphenylphoshonium bromide (3.43 g, 9.6 mmol, 2 equiv.). The crude product was purified via column chromatography using hexane/ethyl acetate (30:1 v/v) as eluent. The desired compound **1e** was obtained as a colorless liquid. Yield: 0.602 g, 2.9 mmol, 60%. ^{1}H NMR (400 MHz, $CDCl_3$, 298 K) δ: 7.33–7.27 (m, 4H, Ar–CH), 7.22–7.17 (m, 4H, Ar–CH), 5.45 (s, 1H, vinylic), 5.44 (s, 1H, vinylic), 2.43 (s, 6H, Me); ^{13}C NMR (101 MHz, $CDCl_3$, 298 K) δ: 149.9, 139.0, 137.5, 129.0, 128.3, 113.1 (CH_2), 21.3 (Me). Data agrees with literature values.[5]

Synthesis of 4,4'-(ethene-1,1-diyl)bis(methoxybenzene) **1f**[6]

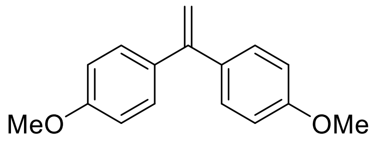

Synthesized in accordance with General Procedure a using 4,4′-dimethoxybenzophenone (1.16 g, 4.8 mmol, 1 equiv.) and methyltriphenylphoshonium bromide (3.43 g, 9.6 mmol, 2 equiv.). The crude product was purified via column chromatography using hexane/ethyl acetate (30:1 v/v) as eluent. The desired compound **1f** was obtained as a white solid. Yield: 0.498 g, 2.1 mmol, 43%. ^{1}H NMR (400 MHz, $CDCl_3$, 298 K) δ: 7.33–7.26 (m, 4H, Ar–CH), 6.92–6.83 (m, 4H, Ar–CH), 5.30 (s, 2H, vinylic), 3.83 (s, 6H, Me); ^{13}C NMR (101 MHz, $CDCl_3$, 298 K) δ: 159.4, 149.1, 134.5, 129.6, 113.6 (CH_2), 111.8, 55.4 (OMe); Melting point: 130–134 °C. NMR data agrees with literature values.[6]

Synthesis of 4,4'-(ethene-1,1-diyl)bis(N,N-dimethylaniline) **1g**[7]

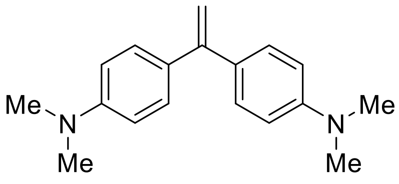

Synthesized in accordance with General Procedure b using bis(4-(dimethylamino)phenyl)methanone (1.29 g, 4.8 mmol, 1 equiv.) and methyltriphenylphoshonium bromide (3.43 g, 9.6 mmol, 2 equiv.). The crude product was purified via column chromatography using hexane/ethyl acetate (80:20 v/v) as eluent. The desired compound **1g** was obtained as a white solid. Yield: 0.828 g, 3.1 mmol, 65%. ^{1}H NMR (400 MHz, $CDCl_3$, 298 K) δ: 7.27 (d, *J* = 8.8 Hz, 4H, Ar–CH), 6.67 (d, *J* = 8.9 Hz, 4H, Ar–CH), 5.20 (s, 2H, vinylic), 2.93 (s, 12H, Me); ^{13}C NMR (101 MHz, $CDCl_3$, 298 K) δ: 150.2, 149.7, 130.3, 129.3, 112.0, 109.0, 40.6. Data agrees with literature values.[7]

Synthesis of 3,3'-(ethene-1,1-diyl)bis((trifluoromethyl)benzene) **1h**

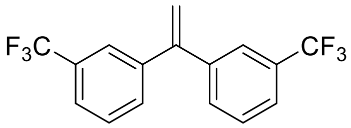

Synthesized in accordance with General Procedure b using bis(3-(trifluoromethyl)phenyl)methanone (1.53 g, 4.8 mmol, 1 equiv.) and methyltriphenylphoshonium bromide (3.43 g, 9.6 mmol, 2 equiv.). The crude product was purified via column chromatography using hexane/ethyl acetate (30:1 v/v) as eluent. The desired compound **1h** was obtained as a colorless liquid. Yield: 0.912 g, 2.9 mmol, 60%. ^{1}H NMR (400 MHz, $CDCl_3$, 298 K) δ: 7.70 – 7.59 (m, 4H, Ar–CH), 7.55 – 7.44 (m, 4H, Ar–CH), 5.63 (s, 2H, vinylic); ^{13}C NMR (101 MHz, $CDCl_3$, 298 K) δ: 148.0, 141.7, 131.62, 131.60, 131.2 (q, $J_{C\text{-}F}$ = 32.3 Hz), 129.1, 125.0 (p, $J_{C\text{-}F}$ = 3.6 Hz), 124.2 (q, $J_{C\text{-}F}$ = 272.5 Hz), 117.2; ^{19}F NMR (376 MHz, $CDCl_3$, 298 K) δ: -62.71. IR ν_{max} (cm^{-1}): 3050, 3029, 1855, 1598, 1500, 1480, 1210, 1100, 814, 741; HRMS (EI) [M] [$C_{16}H_{10}F_6$]: calculated 316.0681, found 316.0672.

Synthesis of 1-fluoro-4-(1-phenylvinyl)benzene **1i**[5]

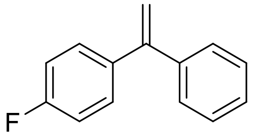

Synthesized in accordance with General Procedure a using 4-fluorobenzophenone (0.96 g, 4.8 mmol, 1 equiv.) and methyltriphenylphoshonium bromide (3.43 g, 9.6 mmol, 2 equiv.). The crude product was purified via column chromatography using hexane/ethyl acetate (30:1 v/v) as eluent. The desired compound **1i** was obtained as a colorless liquid. Yield: 0.550 g, 2.8 mmol, 58%. ^{1}H NMR (400 MHz, $CDCl_3$, 298 K) δ: 7.40–7.29 (m, 7H, Ar–CH), 7.09–7.01 (m, 2H, Ar–

CH), 5.48 (d, J = 1.3 Hz, 1H, vinylic), 5.45 (d, J = 1.3 Hz, 1H, vinylic); ^{13}C NMR (101 MHz, $CDCl_3$, 298 K) δ:162.7 (d, $J_{C–F}$ = 246.7 Hz), 149.2, 141.5, 137.7 (d, $J_{C–F}$ = 3.6 Hz), 130.0 (d, $J_{C–F}$ = 8.0 Hz), 128.4, 128.3, 128.0, 115.2 (d, $J_{C–F}$ = 21.4 Hz), 114.3 (CH_2); ^{19}F NMR (376 MHz, $CDCl_3$, 298 K) δ: -114.6. Data agrees with literature values.[5]

Synthesis of 1-chloro-4-(1-phenylvinyl)benzene **1j**[5]

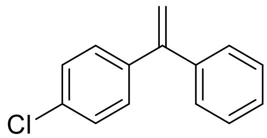

Synthesized in accordance with General Procedure a using 4-chlorobenzophenone (1.04 g, 4.8 mmol, 1 equiv.) and methyltriphenylphoshonium bromide (3.43 g, 9.6 mmol, 2 equiv.). The crude product was purified via column chromatography using hexane/ethyl acetate (30:1 v/v) as eluent. The desired compound **1j** was obtained as a colorless liquid. Yield: 0.600 g, 2.8 mmol, 58%. ^{1}H NMR (400 MHz, $CDCl_3$, 298 K) δ: 7.43–7.28 (m, 9H, Ar–CH), 5.51 (s, 1H, vinylic), 5.49 (s, 1H, vinylic); ^{13}C NMR (101 MHz, $CDCl_3$, 298 K) δ: 149.1, 141.1, 140.1, 133.7, 129.7, 128.5, 128.4, 128.3, 128.1, 114.8 (CH_2). Data agrees with literature values.[5]

Synthesis of 1-methyl-4-(1-phenylvinyl)benzene **1k**[5]

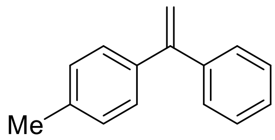

Synthesized in accordance with General Procedure a using 4-methylbenzophenone (0.94 g, 4.8 mmol, 1 equiv.) and methyltriphenylphoshonium bromide (3.43 g, 9.6 mmol, 2 equiv.). The crude product was purified via column chromatography using hexane/ethyl acetate (30:1 v/v) as eluent. The desired compound **1k** was obtained as a colorless liquid. Yield: 0.597 g, 3 mmol, 64%. ^{1}H NMR (400 MHz, $CDCl_3$, 298 K) δ: 7.41–7.32 (m, 5H, Ar–CH), 7.30–7.26 (m, 2H, Ar–CH), 7.20–7.15 (m, 2H, Ar–CH), 5.47 (t, J = 1.5 Hz, 1H, vinylic), 5.44 (t, J = 1.5 Hz, 1H, vinylic), 2.40 (s, 3H, Me); ^{13}C NMR (101 MHz, $CDCl_3$, 298 K) δ: 150.1, 141.8, 138.8, 137.6, 129.0, 128.4, 128.29, 128.25, 127.8, 113.8 (CH_2), 21.3 (Me). Data agrees with literature values.[5]

Synthesis of 2-(1-phenylvinyl)thiophene **1l**[8]

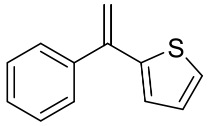

Synthesized in accordance with General Procedure a using 2-benzoylthiophene (0.90 g, 4.8 mmol, 1 equiv.) and methyltriphenylphoshonium bromide (3.43 g, 9.6 mmol, 2 equiv.). The crude product was purified via column

chromatography using hexane/ethyl acetate (30:1 v/v) as eluent. The desired compound **1l** was obtained as a colorless liquid. Yield: 0.479 g, 2.3 mmol, 54%. ^{1}H NMR (400 MHz, $CDCl_3$, 298 K) δ: 7.55–7.47 (m, 2H, Ar–CH), 7.47–7.37 (m, 3H, Ar–CH), 7.28 (dd, J = 5.1, 1.3 Hz, 1H, thiophene–CH), 7.03 (dd, J = 5.1, 3.6 Hz, 1H, thiophene–CH), 6.98 (dd, J = 3.6, 1.3 Hz, 1H, thiophene–CH), 5.66 (d, J = 1.0 Hz, 1H, vinylic), 5.31 (d, J = 1.0 Hz, 1H, vinylic); ^{13}C NMR (101 MHz, $CDCl_3$, 298 K) δ: 144.9, 143.5, 141.2, 128.4, 128.3, 128.2, 127.4, 126.6, 125.1, 113.7 (CH_2). Data agrees with literature values.[8]

Synthesis of 2-(1-phenylvinyl)pyridine **1m**[9]

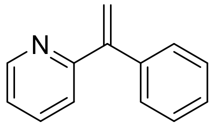

Synthesized in accordance with General Procedure b using phenyl(pyridin-2-yl)methanone (0.88 g, 4.8 mmol, 1 equiv.) and methyltriphenylphoshonium bromide (3.43 g, 9.6 mmol, 2 equiv.). The crude product was purified via column chromatography using hexane/ethyl acetate (80:20 v/v) as eluent. The desired compound **1m** was obtained as a colorless liquid. Yield: 0.275 g, 1.5 mmol, 32%. ^{1}H NMR (400 MHz, $CDCl_3$, 298 K) δ: 8.55 (ddd, J = 4.9, 1.9, 0.9 Hz, 1H), 7.52 (td, J = 7.7, 1.9 Hz, 1H), 7.28 – 7.24 (m, 5H), 7.17 (dt, J = 7.9, 1.1 Hz, 1H), 7.10 (ddd, J = 7.5, 4.8, 1.2 Hz, 1H), 5.91 (d, J = 1.6 Hz, 1H), 5.51 (d, J = 1.6 Hz, 1H); ^{13}C NMR (101 MHz, $CDCl_3$, 298 K) δ: 158.6, 149.5, 149.3, 140.5, 136.4, 128.5, 128.4, 127.9, 122.9, 122.5, 117.8. Data agrees with literature values.[9]

Synthesis of prop-1-ene-1,1-diyldibenzene **1n**[10]

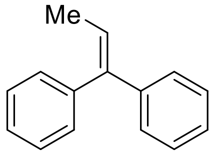

Synthesized in accordance with General Procedure a using benzophenone (0.87 g, 4.8 mmol, 1 equiv.) and ethyltriphenylphoshonium bromide (3.56 g, 9.6 mmol, 2 equiv.). The crude product was purified via column chromatography using hexane/ethyl acetate (30:1 v/v) as eluent. The desired compound **1n** was obtained as a white solid. Yield: 0.595 g, 3.1 mmol, 64%. ^{1}H NMR (400 MHz, $CDCl_3$, 298 K) δ: 7.45–7.37 (m, 2H, Ar–CH), 7.37–7.33 (m, 1H, Ar–CH), 7.32–7.19 (m, 7H, Ar–CH), 6.22 (q, J = 7.1 Hz, 1H, vinylic), 1.80 (d, J = 7.0 Hz, 3H, Me); ^{13}C NMR (101 MHz, $CDCl_3$, 298 K) δ: 143.1, 142.6, 140.2, 130.2, 128.3, 128.2, 127.3, 127.0, 126.9, 124.3, 15.9 (Me); Melting point: 50–55 °C. NMR data agrees with literature values.[10]

Synthesis of 1-methoxy-4-(prop-1-en-2-yl)benzene **1o**[11]

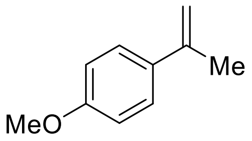

Synthesized in accordance with General Procedure a using 4′-methoxyacetophenone (0.72 g, 4.8 mmol, 1 equiv.) and methyltriphenylphoshonium bromide (3.43 g, 9.6 mmol, 2 equiv.). The crude product was purified via column chromatography using hexane/ethyl acetate (30:1 v/v) as eluent. The desired compound **1o** was obtained as a white solid. Yield: 0.621 g, 4.2 mmol, 87%. ^{1}H NMR (400 MHz, $CDCl_3$, 298 K) δ: 1H NMR (400 MHz, $CDCl_3$, 298 K) δ 7.49–7.36 (m, 2H, Ar–CH), 6.96–6.81 (m, 2H, Ar–CH), 5.30 (dd, *J* = 1.6, 0.8 Hz, 1H, vinylic), 5.03–4.96 (m, 1H, vinylic), 3.82 (s, 3H, OMe), 2.14 (dd, *J* = 1.5, 0.8 Hz, 3H, Me); ^{13}C NMR (101 MHz, $CDCl_3$, 298 K) δ: 159.2, 142.7, 133.9, 126.7, 113.7, 110.8 (CH_2), 55.4 (OMe), 22.1 (Me); Melting point: 40–41 °C. NMR data agrees with literature values.[11]

Synthesis of 1-chloro-4-(prop-1-en-2-yl)benzene **1r**[12]

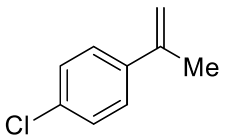

Synthesized in accordance with General Procedure a using *p*-chloroacetophenone (0.74 g, 4.8 mmol, 1 equiv.) and methyltriphenylphoshonium bromide (3.43 g, 9.6 mmol, 2 equiv.). The crude product was purified via column chromatography using hexane/ethyl acetate (30:1 v/v) as eluent. The desired compound **1r** was obtained as a colorless liquid. Yield: 0.601 g, 3.9 mmol, 82%. ^{1}H NMR (400 MHz, $CDCl_3$, 298 K) δ: 7.47–7.38 (m, 2H, Ar–CH), 7.37–7.28 (m, 2H, Ar–CH), 5.39 (s, 1H, vinylic), 5.14 (q, *J* = 1.4 Hz, 1H, vinylic), 2.17 (d, *J* = 1.4 Hz, 3H, Me); ^{13}C NMR (101 MHz, $CDCl_3$, 298 K) δ: 142.3, 139.8, 133.3, 128.4, 126.9, 113.1 (CH_2), 21.8 (Me). Data agrees with literature values.[12]

Synthesis of 1-bromo-4-(prop-1-en-2-yl)benzene **1s**[11]

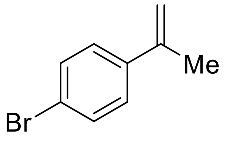

Synthesized in accordance with General Procedure a using *p*-bromoacetophenone (0.96 g, 4.8 mmol, 1 equiv.) and methyltriphenylphoshonium bromide (3.43 g, 9.6 mmol, 2 equiv.). The crude product was purified via column chromatography using hexane/ethyl acetate (30:1 v/v) as eluent. The desired compound **1s** was obtained as a sticky liquid. Yield: 0.699 g, 3.6 mmol, 74%. ^{1}H NMR (400 MHz, $CDCl_3$, 298 K) δ: 7.49–7.43 (m, 2H, Ar–CH), 7.36–7.31 (m, 2H, Ar–CH), 5.37 (s, 1H,

vinylic), 5.12 (q, J = 1.4 Hz, 1H, vinylic), 2.14 (d, J = 1.3 Hz, 3H, Me); ^{13}C NMR (101 MHz, $CDCl_3$, 298 K) δ: 142.3, 140.2, 131.4, 127.3, 121.5, 113.2 (CH_2), 21.8 (Me). Data agrees with literature values.[11]

Synthesis of 1-methyl-4-(prop-1-en-2-yl)benzene **1t**[13]

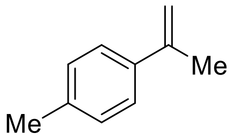

Synthesized in accordance with General Procedure a using *p*-methylacetophenone (6.44 g, 4.8 mmol, 1 equiv.) and methyltriphenylphoshonium bromide (3.43 g, 9.6 mmol, 2 equiv.). The crude product was purified via column chromatography using hexane/ethyl acetate (30:1 v/v) as eluent. The desired compound **1t** was obtained as a colorless liquid. Yield: 0.401 g, 3.0 mmol, 63%. ^{1}H NMR (400 MHz, $CDCl_3$, 298 K) δ: 7.43–7.35 (m, 2H, Ar–CH), 7.19–7.12 (m, 2H, Ar–CH), 5.40–5.30 (m, 1H, vinylic), 5.09–5.01 (m, 1H, vinylic), 2.37 (s, 3H, Me), 2.16 (dd, J = 1.6, 0.8 Hz, 3H, Me); ^{13}C NMR (101 MHz, $CDCl_3$, 298 K) δ: 143.2, 138.5, 137.3, 129.0, 125.5, 111.7 (CH_2), 22.0 (Me), 21.2 (Me). Data agrees with literature values.[13]

Synthesis of 2-(prop-1-en-2-yl)naphthalene **1u**[14]

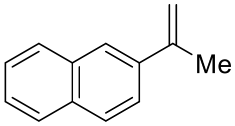

Synthesized in accordance with General Procedure a using 2-acetylnaphthalene (0.81 g, 4.8 mmol, 1 equiv.) and methyltriphenylphoshonium bromide (3.43 g, 9.6 mmol, 2 equiv.). The crude product was purified via column chromatography using hexane/ethyl acetate (30:1 v/v) as eluent. The desired compound **1u** was obtained as a white solid. Yield: 0.640 g, 3.8 mmol, 78%. ^{1}H NMR (400 MHz, $CDCl_3$, 298 K) δ: 7.98–7.80 (m, 4H, Ar–CH), 7.75 (dd, J = 8.6, 1.9 Hz, 1H, Ar–CH), 7.59–7.45 (m, 2H, Ar–CH), 5.62 (s, 1H, vinylic), 5.28 (p, J = 1.5 Hz, 1H, vinylic), 2.39–2.30 (m, 3H, Me); ^{13}C NMR (101 MHz, $CDCl_3$, 298 K) δ: 143.1, 138.5, 133.5, 132.9, 128.4, 127.8, 127.6, 126.2, 125.9, 124.4, 124.0, 113.1 (CH_2), 22.0 (Me); Melting point: 45–47 °C. NMR data agrees with literature values.[14]

Synthesis of 4-(prop-1-en-2-yl)-1,1'-biphenyl **1v**[15]

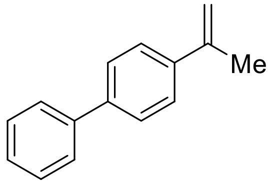

Synthesized in accordance with General Procedure a using 1-([1,1'-biphenyl]-4-yl)ethan-1-one (0.94 g, 4.8 mmol, 1 equiv.) and methyltriphenylphoshonium bromide (3.43 g, 9.6 mmol, 2 equiv.). The crude product was purified via column chromatography using hexane/ethyl acetate (30:1 v/v) as eluent. The desired compound **1v** was obtained as a colorless liquid. Yield: 0.83 g, 4.3 mmol, 89%. ^{1}H NMR (400 MHz, $CDCl_3$, 298 K) δ: 7.64–7.52 (m, 6H, Ar–CH), 7.48–7.40 (m, 2H, Ar–CH), 7.37–7.31 (m, 1H, Ar–CH), 5.44 (dd, J = 1.5, 0.8 Hz, 1H, vinylic), 5.12 (p, J = 1.5 Hz, 1H, vinylic), 2.20 (dd, J = 1.6, 0.8 Hz, 3H, Me); ^{13}C NMR (101 MHz, $CDCl_3$, 298 K) δ: 142.9, 140.9, 140.4, 140.3, 128.9, 127.4, 127.13, 127.07, 126.0, 112.6 (CH_2), 22.0 (Me). Data agrees with literature values.[15]

Synthesis of but-1-en-2-ylbenzene **1w**[16]

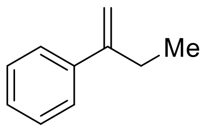

Synthesized in accordance with General Procedure a using propiophenone (0.64 g, 4.8 mmol, 1 equiv.) and methyltriphenylphoshonium bromide (3.43 g, 9.6 mmol, 2 equiv.). The crude product was purified via column chromatography using hexane/ethyl acetate (30:1 v/v) as eluent. The desired compound **1w** was obtained as a colorless liquid. Yield: 0.503 g, 3.8 mmol, 79%. ^{1}H NMR (400 MHz, $CDCl_3$, 298 K) δ: 7.50–7.44 (m, 2H, Ar–CH), 7.41–7.34 (m, 2H, Ar–CH), 7.34–7.28 (m, 1H, Ar–CH), 5.34 (dd, J = 1.5, 0.9 Hz, 1H, vinylic), 5.12 (q, J = 1.5 Hz, 1H, vinylic), 2.58 (qdd, J = 7.4, 1.6, 0.9 Hz, 2H, -CH_2-), 1.17 (t, J = 7.4 Hz, 3H, Me); ^{13}C NMR (101 MHz, $CDCl_3$, 298 K) δ: 150.2, 141.7, 128.4, 127.4, 126.2, 111.1 (CH_2), 28.2 (-CH_2-), 13.1 (Me). Data agrees with literature values.[16]

Synthesis of hept-1-en-2-ylbenzene **1x**[17]

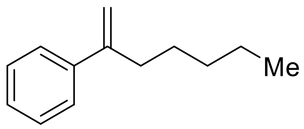

Synthesized in accordance with General Procedure a using hexanophenone (0.85 g, 4.8 mmol, 1 equiv.) and methyltriphenylphoshonium bromide (3.43 g, 9.6 mmol, 2 equiv.). The crude product was purified via column chromatography using hexane/ethyl acetate (30:1 v/v) as eluent. The desired compound **1x** was obtained as a colorless liquid. Yield: 0.371 g, 2.12 mmol, 44%. ^{1}H NMR (400 MHz, $CDCl_3$, 298 K) δ: 7.50–7.42 (m, 2H, Ar–CH), 7.41–7.33 (m, 2H, Ar–CH), 7.33–7.27 (m, 1H, Ar–CH), 5.31 (q, J = 1.7 Hz, 1H, vinylic), 5.21–4.99 (m, 1H, vinylic),

2.63–2.48 (m, 2H, CH_2), 1.59–1.43 (m, 2H, CH_2), 1.44–1.28 (m, 4H, CH_2), 1.02–0.85 (m, 3H, Me); ^{13}C NMR (101 MHz, $CDCl_3$, 298 K) δ: 148.9, 141.6, 128.4, 127.4, 126.3, 112.1 (CH_2 vinylic), 35.5 (CH_2), 31.7 (CH_2), 28.1 (CH_2), 22.7 (CH_2), 14.2 (Me). Data agrees with literature values.[17]

2.5 Synthesis and spectral characterization of products

Synthesis of (2,2-diphenylvinyl)(4-fluorophenyl)sulfane **3aa**[18]

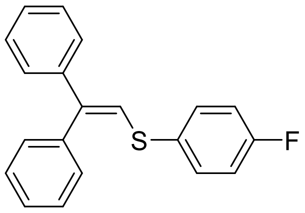

Synthesized in accordance with General Procedure c using 1,1-diphenylethylene **1a** (22 mg, 0.12 mmol, 1.2 equiv.) and *N*-[(4-fluorophenyl)thio]succinimide **2a** (23 mg, 0.1 mmol, 1.0 equiv.). The crude product was purified via preparative thin layer chromatography using hexane as eluent. The desired compound **3aa** was obtained as a colorless oil. Yield: 27 mg, 0.09 mmol, 88%. ^{1}H NMR (400 MHz, $CDCl_3$, 298 K) δ: 7.46–7.39 (m, 4H, Ar–CH), 7.38–7.31 (m, 3H, Ar–CH), 7.31–7.20 (m, 5H, Ar–CH), 7.07–6.98 (m, 2H, Ar–CH), 6.75 (s, 1H, CH); ^{13}C NMR (101 MHz, $CDCl_3$, 298 K) δ: 162.26 (d, $J_{C–F}$ = 247.0 Hz), 141.47, 141.01, 139.20, 132.20 (d, $J_{C–F}$ = 8.0 Hz), 131.66 (d, $J_{C–F}$ = 3.3 Hz), 129.86, 128.56, 128.47, 128.01, 127.49, 127.32, 124.76, 116.40 (d, $J_{C–F}$ = 22.2 Hz); ^{19}F NMR (376 MHz, $CDCl_3$, 298 K) δ: -114.6. Data agrees with literature values.[18]

Synthesis of (2,2-bis(4-fluorophenyl)vinyl)(4-fluorophenyl)sulfane **3ba**[19]

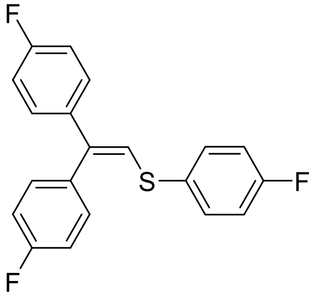

Synthesized in accordance with General Procedure c using 1,1-bis(4-fluorophenyl)ethylene **1b** (26 mg, 0.12 mmol, 1.2 equiv.) and *N*-[(4-fluorophenyl)thio]succinimide **2a** (23 mg, 0.1 mmol, 1.0 equiv.). The crude product was purified via preparative thin layer chromatography using hexane as eluent. The desired compound **3ba** was obtained as a colorless oil. Yield: 27 mg, 0.08 mmol, 79%. ^{1}H NMR (400 MHz, $CDCl_3$, 298 K) δ: 7.46–7.39 (m, 2H, Ar–CH), 7.35–7.28 (m, 2H, Ar–CH), 7.21–7.15 (m, 2H, Ar–CH), 7.15–7.09 (m, 2H, Ar–CH), 7.09–7.02 (m, 2H, Ar–CH), 7.02–6.94 (m, 2H, Ar–CH), 6.68 (s, 1H, CH); ^{13}C NMR (101 MHz, $CDCl_3$, 298 K) δ: 162.46 (d, $J_{C–F}$ = 247.8 Hz), 162.44 (d, $J_{C–F}$ = 247.4 Hz), 162.39 (d, $J_{C–F}$ = 247.8 Hz), 138.9, 137.6 (d, $J_{C–F}$ = 3.3 Hz), 134.9 (d, $J_{C–F}$ = 3.3 Hz),

132.4 (d, $J_{C–F}$ = 8.0 Hz), 131.6 (d, $J_{C–F}$ = 8.0 Hz), 131.2 (d, $J_{C–F}$ = 3.6 Hz), 128.9 (d, $J_{C–F}$ = 8.0 Hz), 125.0, 116.5 (d, $J_{C–F}$ = 21.8 Hz), 115.7 (d, $J_{C–F}$ = 21.1 Hz), 115.4 (d, $J_{C–F}$ = 21.4 Hz); ^{19}F NMR (376 MHz, $CDCl_3$, 298 K) δ: -113.3, -114.2, -114.6. Data agrees with literature values.[19]

Synthesis of (2,2-bis(4-chlorophenyl)vinyl)(4-fluorophenyl)sulfane **3ca**

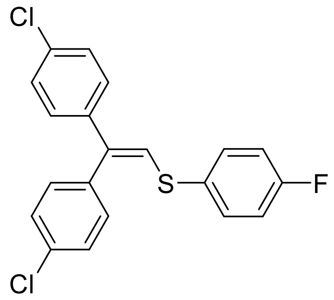

Synthesized in accordance with General Procedure c using 1,1-bis(4-chlorophenyl)ethylene **1c** (30 mg, 0.12 mmol, 1.2 equiv.) and *N*-[(4-fluorophenyl)thio]succinimide **2a** (23 mg, 0.1 mmol, 1.0 equiv.). The crude product was purified via preparative thin-layer chromatography using hexane as eluent. The desired compound **3ca** was obtained as a colorless oil. Yield: 25 mg, 0.07 mmol, 72%. ^{1}H NMR (400 MHz, $CDCl_3$, 298 K) δ: 7.51–7.35 (m, 4H, Ar–CH), 7.34–7.21 (m, 4H, Ar–CH), 7.19–7.11 (m, 2H, Ar–CH), 7.11–7.01 (m, 2H, Ar–CH), 6.76 (s, 1H, CH); ^{13}C NMR (101 MHz, $CDCl_3$, 298 K) δ: 162.49 (d, $J_{C–F}$ = 248.1 Hz), 139.58, 138.19, 137.14, 134.06, 133.54, 132.57 (d, $J_{C–F}$ = 8.4 Hz), 131.21, 130.92 (d, $J_{C–F}$ = 3.3 Hz), 128.98, 128.73, 128.48, 126.37, 116.58 (d, $J_{C–F}$ = 21.8 Hz); ^{19}F NMR (376 MHz, $CDCl_3$, 298 K) δ: -113.9; IR ν_{max} (cm^{-1}): 3032, 2924, 2349, 1591, 1489, 1396, 1229, 1090, 1013, 829, 806; HRMS (APCI) [M] [$C_{20}H_{13}FSCl_2$]: calculated 374.0099, found 374.0097.

Synthesis of (2,2-bis(4-bromophenyl)vinyl)(4-fluorophenyl)sulfane **3da**

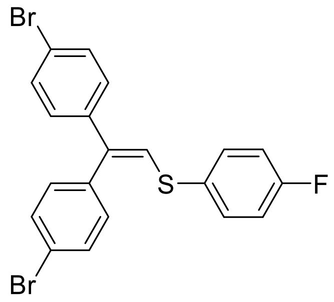

Synthesized in accordance with General Procedure c using 1,1-bis(4-bromophenyl)ethylene **1d** (41 mg, 0.12 mmol, 1.2 equiv.) and *N*-[(4-fluorophenyl)thio]succinimide **2a** (23 mg, 0.1 mmol, 1.0 equiv.). The crude product was purified via preparative thin layer chromatography using hexane as eluent. The desired compound **3da** was obtained as a white solid. Yield: 39 mg, 0.08 mmol, 84%. ^{1}H NMR (400 MHz, $CDCl_3$, 298 K) δ: 7.59–7.53 (m, 2H, Ar–CH), 7.46–7.37 (m, 4H, Ar–CH), 7.25–7.17 (m, 2H, Ar–CH), 7.10–7.02 (m, 4H, Ar–CH), 6.76 (s, 1H, CH); ^{13}C NMR (101 MHz, $CDCl_3$, 298 K) δ: 162.5 (d, J_{C-F} = 247.8 Hz), 139.9, 138.2, 137.5, 132.6 (d, $J_{C–F}$ = 8.4 Hz), 131.9, 131.7, 131.5, 130.84 (d, $J_{C–F}$ = 3.3 Hz), 128.79, 126.5, 122.3, 121.7, 116.6 (d, $J_{C–F}$ = 22.2 Hz); ^{19}F NMR (376 MHz, $CDCl_3$, 298 K) δ: -113.8; IR ν_{max} (cm^{-1}): 3026, 2924, 2852, 1901, 1749, 1589, 1487, 1393, 1227, 1069,

1009, 800, 629; HRMS (APCI) [M] [$C_{20}H_{13}FSBr_2$]: calculated 463.9068, found 463.9068; Melting point: 105–108 °C.

Synthesis of (2,2-di-p-tolylvinyl)(4-fluorophenyl)sulfane **3ea**

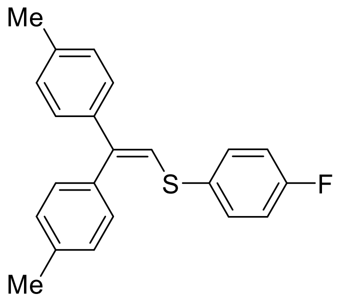

Synthesized in accordance with General Procedure c using 1,1-bis(4-methylphenyl)ethylene **1e** (25 mg, 0.12 mmol, 1.2 equiv.) and *N*-[(4-fluorophenyl)thio]succinimide **2a** (23 mg, 0.1 mmol, 1.0 equiv.). The crude product was purified via preparative thin layer chromatography using hexane as eluent. The desired compound **3ea** was obtained as a colorless oil. Yield: 28 mg, 0.08 mmol, 84%. ^{1}H NMR (400 MHz, $CDCl_3$, 298 K) δ: 7.49–7.38 (m, 2H, Ar–CH), 7.30–7.23 (m, 4H, Ar–CH), 7.19–7.09 (m, 4H, Ar–CH), 7.09–7.01 (m, 2H, Ar–CH), 6.71 (s, 1H, CH), 2.43 (s, 3H, Me), 2.36 (s, 3H, Me); ^{13}C NMR (101 MHz, $CDCl_3$, 298 K) δ: 162.1 (d, $J_{C–F}$ = 246.7 Hz), 141.3, 138.9, 137.7, 137.3, 136.4, 131.94 (d, $J_{C–F}$ = 8.0 Hz), 131.94, 129.73, 129.21, 129.13, 127.29, 123.06, 116.33 (d, $J_{C–F}$ = 22.2 Hz), 21.50 (Me), 21.24 (Me); ^{19}F NMR (376 MHz, $CDCl_3$, 298 K) δ: -115.0; IR ν_{max} (cm^{-1}): 3022, 2920, 2862, 1589, 1512, 1489, 1227, 1155, 821, 800, 771; HRMS (APCI) [M] [$C_{22}H_{19}FS$]: calculated 334.1191, found 334.1189.

Synthesis of (2,2-bis(4-methoxyphenyl)vinyl)(4-fluorophenyl)sulfane **3fa**

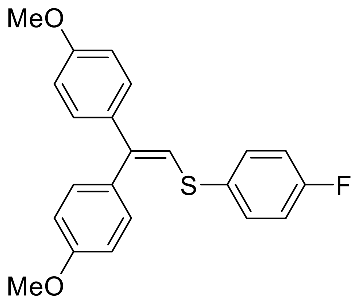

Synthesized in accordance with General Procedure c using 1,1-bis(4-methoxyphenyl)ethylene **1f** (29 mg, 0.12 mmol, 1.2 equiv.) and *N*-[(4-fluorophenyl)thio]succinimide **2a** (23 mg, 0.1 mmol, 1.0 equiv.). The crude product was purified via preparative thin layer chromatography using hexane as eluent. The desired compound **3fa** was obtained as a yellow oil. Yield: 33 mg, 0.09 mmol, 90%. ^{1}H NMR (400 MHz, $CDCl_3$, 298 K) δ: 7.45–7.38 (m, 2H, Ar–CH), 7.31–7.27 (m, 2H, Ar–CH), 7.22–7.17 (m, 2H, Ar–CH), 7.07–7.00 (m, 2H, Ar–CH), 6.99–6.94 (m, 2H, Ar–CH), 6.86–6.80 (m, 2H, Ar–CH), 6.59 (s, 1H, CH), 3.86 (s, 3H, OMe), 3.81 (s, 3H, OMe); ^{13}C NMR (101 MHz, $CDCl_3$, 298 K) δ: 162.1 (d, $J_{C–F}$ = 247.0 Hz), 159.3, 159.2, 141.0, 134.6, 132.1 (d, $J_{C–F}$ = 3.3 Hz), 131.8 (d, $J_{C–F}$ = 8.0 Hz), 131.7, 131.1, 128.7, 121.5, 116.3 (d, $J_{C–F}$ = 22.2 Hz), 113.81, 113.80, 55.42

(OMe), 55.36 (OMe); ^{19}F NMR (376 MHz, $CDCl_3$, 298 K) δ: -115.1; IR ν_{max} (cm^{-1}): 2953, 2835, 1743, 1605, 1508, 1487, 1283, 1244, 1224, 1173, 1032, 816; HRMS (APCI) [M] [$C_{22}H_{19}O_2FS$]: calculated 366.1090, found 366.1092.

Synthesis of 4,4'-(2-((4-fluorophenyl)thio)ethene-1,1-diyl)bis(N,N-dimethylaniline) **3ga**

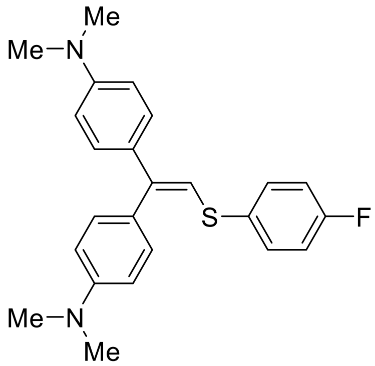

Synthesized in accordance with General Procedure c using 1,1-bis(4-dimethylaminophenyl)ethylene **1g** (32 mg, 0.12 mmol, 1.2 equiv.) and *N*-[(4-fluorophenyl)thio]succinimide **2a** (23 mg, 0.1 mmol, 1.0 equiv.). The crude product was purified via preparative thin layer chromatography using hexane/ethyl acetate (80:20 v/v) as eluent. The desired compound **3ga** was obtained as a colorless oil. Yield: 29 mg, 0.07 mmol, 74%. ^{1}H NMR (400 MHz, $CDCl_3$, 298 K) δ: 7.45–7.37 (m, 2H, Ar–CH), 7.28–7.22 (m, 2H, Ar–CH), 7.22–7.17 (m, 2H, Ar–CH), 7.07–6.98 (m, 2H, Ar–CH), 6.79–6.73 (m, 2H, Ar–CH), 6.70–6.62 (m, 2H, Ar–CH), 6.47 (s, 1H, CH), 3.01 (s, 6H, Me), 2.97 (s, 6H, Me); ^{13}C NMR (101 MHz, $CDCl_3$, 298 K) δ: 161.78 (d, $J_{C\text{-}F}$ = 246.0 Hz), 150.04, 150.00, 143.18, 133.12 (d, $J_{C\text{–}F}$ = 3.3 Hz), 131.10 (d, $J_{C\text{–}F}$ = 8.0 Hz), 130.91, 130.64, 128.62, 127.44, 117.67, 116.12 (d, $J_{C\text{–}F}$ = 22.2 Hz), 112.14, 111.82, 40.63 (Me), 40.54 (Me); ^{19}F NMR (376 MHz, $CDCl_3$, 298 K) δ: -116.1. IR ν_{max} (cm^{-1}): 3020, 3029, 1825, 1588, 1511, 1420, 1206, 1190, 814, 741, 694; HRMS (ES+) $[M+H]^+$ $[C_{24}H_{26}N_2FS]^+$: calculated 393.1801, found 393.1800.

Synthesis of (2,2-bis(3-(trifluoromethyl)phenyl)vinyl)(4-fluorophenyl)sulfane **3ha**

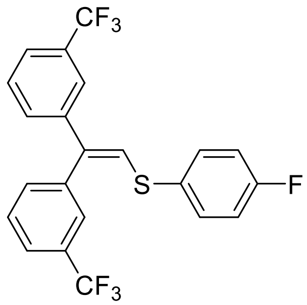

Synthesized in accordance with General Procedure c using 1,1-bis(*m*-trifluoromethylphenyl)ethylene **1h** (38 mg, 0.12 mmol, 1.2 equiv.) and *N*-[(4-fluorophenyl)thio]succinimide **2a** (23 mg, 0.1 mmol, 1.0 equiv.). The crude product was purified via preparative thin layer chromatography using hexane as eluent. The desired compound **3ha** was obtained as a colorless oil. Yield: 23 mg, 0.05 mmol, 52%. ^{1}H NMR (400 MHz, $CDCl_3$, 298 K) δ: 7.72–7.50 (m, 5H, Ar–CH), 7.50–7.36 (m, 4H, Ar–CH), 7.33 (d, *J* = 8.0 Hz, 1H, Ar–CH), 7.16–7.00 (m, 2H, Ar–CH), 6.89 (s, 1H, CH); ^{13}C NMR (101 MHz, $CDCl_3$, 298 K) δ: 162.7 (d, $J_{C\text{–}F}$ = 248.5 Hz), 141.6, 139.2, 137.2, 133.2 (d, $J_{C\text{–}F}$ = 2.1 Hz), 133.0 (d, $J_{C\text{–}F}$

= 8.4 Hz), 131.8 (d, $J_{C–F}$ = 13.4 Hz), 131.4 (d, $J_{C–F}$ = 13.1 Hz), 131.1 (d, $J_{C–F}$ = 12.8 Hz), 130.8 (d, $J_{C–F}$ = 12.8 Hz), 130.5, 130.3 (d, $J_{C–F}$ = 3.5 Hz), 129.1 (d, $J_{C–F}$ = 21.1 Hz), 129.1, 126.6 (q, $J_{C–F}$ = 3.8 Hz), 125.5 (d, $J_{C–F}$ = 2.9 Hz), 125.1 (d, $J_{C–F}$ = 3.7 Hz), 124.4 (d, $J_{C–F}$ = 3.7 Hz), 123.7 (q, $J_{C–F}$ = 3.8 Hz), 122.8 (d, $J_{C–F}$ = 3.0 Hz), 116.7 (d, $J_{C–F}$ = 22.0 Hz); ^{19}F NMR (376 MHz, $CDCl_3$, 298 K) δ: -62.6, -62.7, -113.3. IR ν_{max} (cm^{-1}): 3042, 3092, 1851, 1545, 1511, 1488, 1211, 1106, 841, 744, 689; HRMS (ES+) [M]$^+$ [$C_{22}H_{13}F_7S$]$^+$: calculated 442.0626, found 442.0618.

Synthesis of (4-fluorophenyl)(2-(4-fluorophenyl)-2-phenylvinyl)sulfane **3ia**

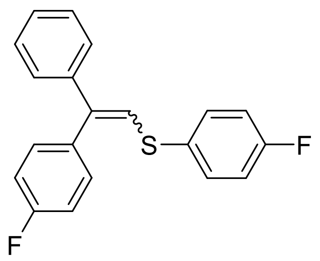

Synthesized in accordance with General Procedure c using 1-(4-fluorophenyl)-1-phenylethene **1i** (24 mg, 0.12 mmol, 1.2 equiv.) and *N*-[(4-fluorophenyl)thio]succinimide **2a** (23 mg, 0.1 mmol, 1.0 equiv.). The crude product was purified via preparative thin layer chromatography using hexane as eluent. The desired compound **3ia** was obtained as a colorless oil with an *E* and *Z* ratio = 1:1. Combined yield (*E* and *Z*): 27 mg, 0.08 mmol, 83%. ^{1}H NMR (400 MHz, $CDCl_3$, 298 K) δ: 7.49–7.31 (m, 12H, Ar–CH), 7.31–7.18 (m, 6H, Ar–CH), 7.17–7.10 (m, 2H, Ar–CH), 7.10–7.03 (m, 4H, Ar–CH), 7.03–6.95 (m, 2H, Ar–CH), 6.77 (s, 1H, CH), 6.71 (s, 1H, CH); ^{13}C NMR (101 MHz, $CDCl_3$, 298 K) δ: 162.40 (d, $J_{C–F}$ = 247.4 Hz), 162.37 (d, $J_{C–F}$ = 247.4 Hz), 162.33 (d, $J_{C–F}$ = 247.4 Hz), 162.31 (d, $J_{C–F}$ = 247.4 Hz), 141.4, 139.94, 139.93, 139.0, 137.7 (d, $J_{C–F}$ = 3.3 Hz), 135.1 (d, $J_{C–F}$ = 3.3 Hz), 132.29 (d, $J_{C–F}$ = 8.0 Hz), 132.27 (d, $J_{C–F}$ = 8.0 Hz), 131.7 (d, $J_{C–F}$ = 8.0 Hz), 131.5 (d, $J_{C–F}$ = 3.3 Hz), 131.4 (d, $J_{C–F}$ = 3.3 Hz), 129.8, 128.9 (d, $J_{C–F}$ = 8.0 Hz), 128.6, 128.5, 128.2, 127.6, 127.3, 125.1, 124.6, 116.47 (d, $J_{C–F}$ = 22.0 Hz), 116.45 (d, $J_{C–F}$ = 22.1 Hz), 115.6 (d, $J_{C–F}$ = 21.4 Hz), 115.4 (d, $J_{C–F}$ = 21.4 Hz) (*all the J coupling of C–F could not be determined due to the overlap of multiple peaks*); ^{19}F NMR (376 MHz, $CDCl_3$, 298 K) δ: -113.6, -114.4, -114.5, -114.9; IR ν_{max} (cm^{-1}): 3055, 3030, 1884, 1589, 1504, 1489, 1223, 1155, 814, 754, 696; HRMS (APCI) [M] [$C_{20}H_{14}F_2S$]: calculated 324.0784, found 324.0780.

Synthesis of (2-(4-chlorophenyl)-2-phenylvinyl)(4-fluorophenyl)sulfane **3ja**

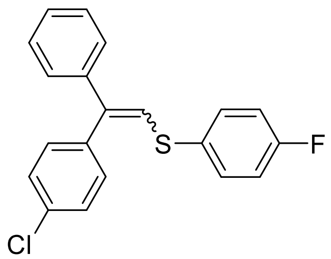

Synthesized in accordance with General Procedure c using 1-phenyl-1-(*p*-chlorophenyl)ethylene **1j** (26 mg, 0.12 mmol, 1.2 equiv.) and *N*-[(4-fluorophenyl)thio]succinimide **2a** (23 mg, 0.1 mmol, 1.0 equiv.). The crude product was purified via preparative thin layer chromatography using hexane as eluent. The desired compound **3ja** was obtained as a colorless oil with an *E* and *Z* ratio = 1:1. Combined yield (*E* and *Z*): 27 mg, 0.08 mmol, 79%. ^{1}H NMR (400 MHz, $CDCl_3$, 298 K) δ: 7.52–7.37 (m, 9H, Ar–CH), 7.37–7.27 (m, 8H, Ar–CH), 7.27–7.13 (m, 5H, Ar–CH), 7.13–7.01 (m, 4H, Ar–CH), 6.78 (s, 2H, CH); ^{13}C NMR (101 MHz, $CDCl_3$, 298 K) δ: 162.37 (d, $J_{C–F}$ = 247.8 Hz), 141.13, 139.91, 139.70, 139.48, 138.75, 137.59, 133.83, 133.31, 132.44 (d, $J_{C–F}$ = 8.0 Hz), 132.33 (d, $J_{C–F}$ = 8.4 Hz), 131.33 (d, $J_{C–F}$ = 3.3 Hz), 131.29, 131.24 (d, $J_{C–F}$ = 3.3 Hz), 129.78, 128.84, 128.69, 128.62, 128.57, 128.51, 128.21, 127.69, 127.30, 125.66, 125.45, 116.50 (d, $J_{C–F}$ = 21.8 Hz), 116.48 (d, $J_{C–F}$ = 22.2 Hz); ^{19}F NMR (376 MHz, $CDCl_3$, 298 K) δ: -114.2; IR ν_{max} (cm^{-1}): 3057, 3028, 2349, 1744, 1589, 1487, 1396, 1229, 1090, 1013, 814, 754, 698; HRMS (APCI) [M] [$C_{20}H_{14}FSCl$]: calculated 340.0489, found 340.0483.

Synthesis of (4-fluorophenyl)(2-phenyl-2-(p-tolyl)vinyl)sulfane **3ka**

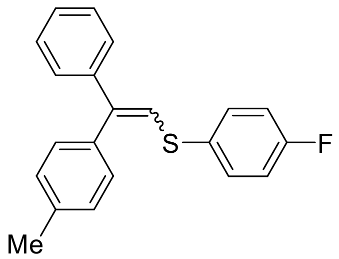

Synthesized in accordance with General Procedure c using 1-phenyl-1-*p*-tolylethylene **1k** (23 mg, 0.12 mmol, 1.2 equiv.) and *N*-[(4-fluorophenyl)thio]succinimide **2a** (23 mg, 0.1 mmol, 1.0 equiv.). The crude product was purified via preparative thin layer chromatography using hexane as eluent. The desired compound **3ka** was obtained as a white solid with an *E* and *Z* ratio = 1:1. Combined yield (*E* and *Z*): 29 mg, 0.09 mmol, 91%. ^{1}H NMR (400 MHz, $CDCl_3$, 298 K) δ: 7.47–7.41 (m, 6H, Ar–CH), 7.40–7.22 (m, 11H, Ar–CH), 7.22–6.98 (m, 9H, Ar–CH), 6.74 (s, 2H, CH), 2.39 (s, 6H, Me); ^{13}C NMR (101 MHz, $CDCl_3$, 298 K) δ: 162.22 (d, $J_{C–F}$ = 247.0 Hz), 162.18 (d, $J_{C–F}$ = 247.0 Hz), 141.6, 141.2, 141.1, 139.4, 138.7, 137.8, 137.4, 136.2, 132.1 (d, $J_{C–F}$ = 8.0 Hz), 132.0 (d, $J_{C–F}$ = 8.0 Hz), 131.83 (d, $J_{C–F}$ = 3.3 Hz), 131.78 (d, $J_{C–F}$ = 3.4 Hz), 129.8, 129.7, 129.3, 129.2, 128.5, 128.4, 127.9, 127.44, 127.37, 127.2, 124.3, 123.6, 116.37 (d, $J_{C–F}$ = 22.2 Hz), 116.36 (d, $J_{C–F}$ = 22.1 Hz), 21.5 (Me), 21.2 (Me); ^{19}F NMR (376

MHz, $CDCl_3$, 298 K) δ: -114.7, -114.8; IR ν_{max} (cm^{-1}): 3022, 2920, 2326, 1747, 1589, 1508, 1489, 1441, 1227,1155, 802, 696; HRMS (APCI) [M] [$C_{21}H_{17}FS$]: calculated 320.1035, found 320.1035.

Synthesis of 2-(2-((4-fluorophenyl)thio)-1-phenylvinyl)thiophene **3la**

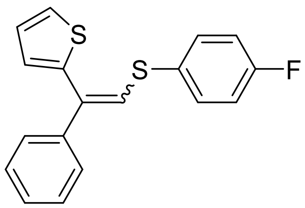

Synthesized in accordance with General Procedure c using 1-phenyl-1-(2-thienyl)ethylene **1l** (22 mg, 0.12 mmol, 1.2 equiv.) and *N*-[(4-fluorophenyl)thio]succinimide **2a** (23 mg, 0.1 mmol, 1.0 equiv.). The crude product was purified via preparative thin layer chromatography using hexane as eluent. The desired compound **3la** was obtained as a colorless oil with an *E* and *Z* ratio = 1:1. Combined yield (*E* and *Z*): 16 mg, 0.05 mmol, 51%. ^{1}H NMR (400 MHz, $CDCl_3$, 298 K) δ: 7.51–7.43 (m, 4H, Ar–CH), 7.43–7.30 (m, 11H, Ar–CH), 7.18 (dd, J = 5.1, 1.2 Hz, 1H, Ar–CH), 7.14 (dd, J = 3.6, 1.3 Hz, 1H), 7.11–7.01 (m, 5H), 6.93 (dd, J = 5.1, 3.6 Hz, 1H), 6.80 (s, 1H, CH), 6.70 (dd, J = 3.7, 1.2 Hz, 1H), 6.55 (s, 1H, CH); ^{13}C NMR (101 MHz, $CDCl_3$, 298 K) δ: 162.5 (d, $J_{C–F}$ = 247.8 Hz), 162.3 (d, $J_{C–F}$ = 247.0 Hz), 145.5, 142.2, 141.2, 138.4, 135.6, 133.8, 132.7 (d, $J_{C–F}$ = 8.0 Hz), 132.1 (d, $J_{C–F}$ = 8.4 Hz), 131.31 (d, $J_{C–F}$ = 2.6 Hz), 131.28 (d, $J_{C–F}$ = 2.9 Hz), 129.5, 128.9, 128.6, 128.4, 128.2, 127.9, 127.6, 126.9, 126.5, 125.9, 125.3, 124.6, 123.2, 116.6 (d, $J_{C–F}$ = 8.0 Hz), 116.4 (d, $J_{C–F}$ = 8.0 Hz); ^{19}F NMR (376 MHz, $CDCl_3$, 298 K) δ: -114.0, -114.6; IR ν_{max} (cm^{-1}): 3067, 2347, 1747, 1589, 1489, 1228, 1155, 1089, 826, 696; HRMS (APCI) [M] [$C_{18}H_{13}FS_2$]: calculated 312.0443, found 312.0443.

Synthesis of (1,1-diphenylprop-1-en-2-yl)(4-fluorophenyl)sulfane **3na**

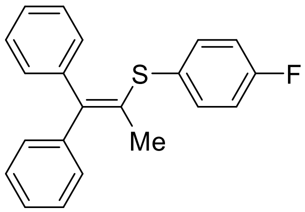

Synthesized in accordance with General Procedure c using 1,1-diphenyl-1-propene **1n** (23 mg, 0.12 mmol, 1.2 equiv.) and *N*-[(4-fluorophenyl)thio]succinimide **2a** (23 mg, 0.1 mmol, 1.0 equiv.). The crude product was purified via preparative thin layer chromatography using hexane as eluent. The desired compound **3na** was obtained as a colorless oil. Yield: 21 mg, 0.07 mmol, 66%. ^{1}H NMR (400 MHz, $CDCl_3$, 298 K) δ: 7.39–7.14 (m, 12H, Ar–CH), 7.04–6.94 (m, 2H, Ar–CH), 1.93 (s, 3H, Me); ^{13}C NMR (101 MHz, $CDCl_3$, 298 K) δ: 162.3 (d, $J_{C–F}$ = 247.0 Hz), 143.7, 142.5, 142.1, 134.0 (d, $J_{C–F}$ = 8.0 Hz), 130.1 (d, $J_{C–F}$ = 3.3 Hz), 129.70, 129.68, 129.6, 128.3, 128.1, 127.3, 127.1, 116.1 (d, $J_{C–F}$ = 21.8 Hz), 21.9 (Me); ^{19}F

NMR (376 MHz, $CDCl_3$, 298 K) δ: -114.7; IR ν_{max} (cm^{-1}): 3055, 2918, 2308, 1587, 1487, 1441, 1220, 1155, 827, 698; HRMS (APCI) [M] [$C_{21}H_{17}FS$]: calculated 320.1035, found 320.1042.

Synthesis of (4-fluorophenyl)(2-(4-methoxyphenyl)prop-1-en-1-yl)sulfane **3oa**

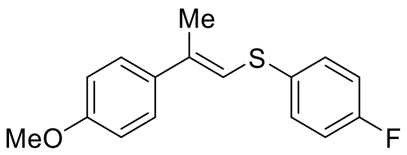

Synthesized in accordance with General Procedure c using 4-methoxy-α-methylstyrene **1o** (18 mg, 0.12 mmol, 1.2 equiv.) and *N*-[(4-fluorophenyl)thio]succinimide **2a** (23 mg, 0.1 mmol, 1.0 equiv.). The crude product was purified via preparative thin layer chromatography using hexane/ethyl acetate as eluent (90:10). The desired compound **3oa** was obtained as a colorless oil. Yield: 11 mg, 0.04 mmol, 40%. ^{1}H NMR (400 MHz, $CDCl_3$, 298 K) δ: 7.41–7.31 (m, 4H, Ar–CH), 7.07–6.98 (m, 2H, Ar–CH), 6.91–6.83 (m, 2H, Ar–CH), 6.39 (q, J = 1.1 Hz, 1H, CH), 3.82 (s, 3H, OMe), 2.23 (d, J = 1.0 Hz, 3H, Me); ^{13}C NMR (101 MHz, $CDCl_3$, 298 K) δ: 162.0 (d, $J_{C–F}$ = 246.3 Hz), 159.2, 137.4, 134.4, 131.8 (d, $J_{C–F}$ = 3.3 Hz), 131.4 (d, $J_{C–F}$ = 8.0 Hz), 126.7, 119.7, 116.3 (d, $J_{C–F}$ = 21.8 Hz), 113.9, 55.5 (OMe), 17.9 (Me); ^{19}F NMR (376 MHz, $CDCl_3$, 298 K) δ: -115.7; IR ν_{max} (cm^{-1}): 2933, 2835, 2349, 2326, 1747, 1606, 1510, 1489, 1250, 1227, 1180, 1033, 815; HRMS (APCI) [M] [$C_{16}H_{15}OFS$]: calculated 274.0828, found 274.0826.

Synthesis of (2-(4-methoxyphenyl)prop-1-en-1-yl)(p-tolyl)sulfane **3ob**

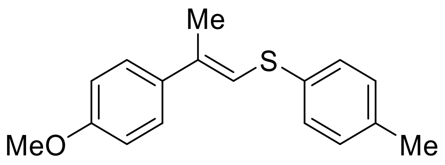

Synthesized in accordance with General Procedure c using 4-methoxy-α-methylstyrene **1o** (18 mg, 0.12 mmol, 1.2 equiv.) and *N*-[(4-methylphenyl)thio]succinimide **2b** (22 mg, 0.1 mmol, 1.0 equiv.). The crude product was purified via preparative thin layer chromatography using hexane as eluent. The desired compound **3ob** was obtained as a white solid. Yield: 16 mg, 0.06 mmol, 59%. ^{1}H NMR (400 MHz, $CDCl_3$, 298 K) δ: 7.38–7.28 (m, 4H, Ar–CH), 7.19–7.08 (m, 2H, Ar–CH), 6.90–6.82 (m, 2H, Ar–CH), 6.45 (q, J = 1.0 Hz, 1H, CH), 3.82 (s, 3H, OMe), 2.34 (s, 3H, Me), 2.23 (d, J = 1.1 Hz, 3H, Me); ^{13}C NMR (101 MHz, $CDCl_3$, 298 K) δ: 159.0, 136.53, 136.46, 134.6, 133.1, 129.9, 129.5, 126.6, 120.2, 113.9, 55.5 (OMe), 21.2 (Me), 17.9 (Me); IR ν_{max} (cm^{-1}): 2361, 1603, 1558, 1508, 1489, 1252,

1182, 1092, 1028, 816, 799; HRMS (EI) [M] [$C_{17}H_{18}OS$]: calculated 270.10729, found 270.1071); Melting point: 105–109 °C.

Synthesis of (2,2-diphenylvinyl)(phenyl)sulfane **3ac**[18]

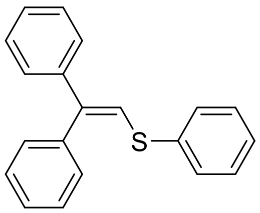

Synthesized in accordance with General Procedure c using 1,1-diphenylethylene **1a** (22 mg, 0.12 mmol, 1.2 equiv.) and *N*-[(phenyl)thio]succinimide **2c** (21 mg, 0.1 mmol, 1.0 equiv.). The crude product was purified via preparative thin layer chromatography using hexane as eluent. The desired compound **3ac** was obtained as a colorless oil. Yield: 26 mg, 0.09 mmol, 90%. ^{1}H NMR (400 MHz, $CDCl_3$, 298 K) δ: 7.45–7.38 (m, 4H, Ar–CH), 7.38–7.20 (m, 11H, Ar–CH), 6.86 (s, 1H, CH); ^{13}C NMR (101 MHz, $CDCl_3$, 298 K) δ: 141.6, 141.2, 139.3, 136.6, 129.9, 129.7, 129.3, 128.53, 128.45, 128.0, 127.4, 127.3, 126.9, 124.2. Data agrees with literature values.[18]

Synthesis of (2,2-diphenylvinyl)(p-tolyl)sulfane **3ab**[18]

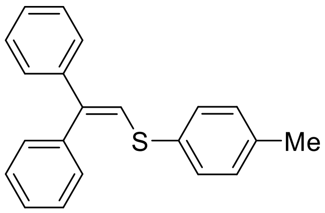

Synthesized in accordance with General Procedure c using 1,1-diphenylethylene **1a** (22 mg, 0.12 mmol, 1.2 equiv.) and *N*-[(4-methylphenyl)thio]succinimide **2b** (22 mg, 0.1 mmol, 1.0 equiv.). The crude product was purified via preparative thin layer chromatography using hexane as eluent. The desired compound **3ab** was obtained as a white solid. Yield: 20 mg, 0.07 mmol, 66%. ^{1}H NMR (400 MHz, $CDCl_3$, 298 K) δ: 7.50–7.43 (m, 2H, Ar–CH), 7.43–7.36 (m, 5H, Ar–CH), 7.36–7.23 (m, 5H, Ar–CH), 7.20–7.15 (m, 2H, Ar–CH), 6.87 (s, 1H, CH), 2.38 (s, 3H, Me); ^{13}C NMR (101 MHz, $CDCl_3$, 298 K) δ: 141.7, 140.3, 139.4, 137.1, 133.0, 130.2, 130.0, 129.9, 128.5, 128.4, 127.9, 127.3, 125.4, 21.2 (Me); Melting point: 90–92 °C. NMR data agrees with literature values.[18]

Synthesis of (4-(tert-butyl)phenyl)(2,2-diphenylvinyl)sulfane **3ad**[18]

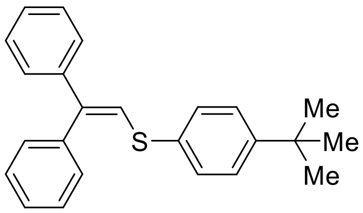

Synthesized in accordance with General Procedure c using 1,1-diphenylethylene **1a** (22 mg, 0.12 mmol, 1.2 equiv.) and *N*-[((4-(*tert*-butyl)phenyl)thio]succinimide **2d** (26 mg, 0.1 mmol, 1.0 equiv.). The crude product was purified via preparative thin layer chromatography using hexane as eluent. The desired compound **3ad** was obtained as a colorless oil. Yield: 28 mg, 0.08 mmol, 81%. ^{1}H NMR (400 MHz, $CDCl_3$, 298 K) δ: 7.45–7.32 (m, 9H, Ar–CH), 7.31–7.21 (m, 5H, Ar–CH), 6.86 (s, 1H, CH), 1.32 (s, 9H, Me); ^{13}C NMR (101 MHz, $CDCl_3$, 298 K) δ: 150.3, 141.7, 140.4, 139.4, 133.0, 129.93, 129.89, 128.5, 128.4, 127.9, 127.3, 126.3, 125.3, 34.7 (Me), 31.4 (Me). Data agrees with literature values.[18]

Synthesis of (3,4-dimethylphenyl)(2,2-diphenylvinyl)sulfane **3ae**

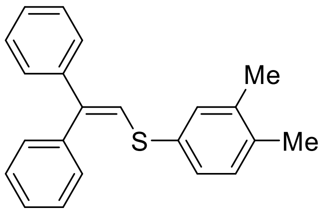

Synthesized in accordance with General Procedure c using 1,1-diphenylethylene **1a** (22 mg, 0.12 mmol, 1.2 equiv.) and *N*-[(3,4-dimethylphenyl)thio]succinimide **2e** (24 mg, 0.1 mmol, 1.0 equiv.). The crude product was purified via preparative thin layer chromatography using hexane as eluent. The desired compound **3ae** was obtained as a colorless oil. Yield: 25 mg, 0.08 mmol, 79%. ^{1}H NMR (400 MHz, $CDCl_3$, 298 K) δ: 7.35–7.30 (m, 2H, Ar–CH), 7.29–7.23 (m, 3H, Ar–CH), 7.22–7.12 (m, 5H, Ar–CH), 7.00–6.94 (m, 2H, Ar–CH), 6.80–6.78 (m, 1H, Ar–CH), 6.78 (s, 1H, CH), 2.21 (s, 6H, Me); ^{13}C NMR (101 MHz, $CDCl_3$, 298 K) δ: 141.7, 140.6, 139.4, 138.9, 136.0, 129.9, 129.2, 128.9, 128.5, 128.4, 128.2, 127.8, 127.5, 127.32, 127.30, 127.0, 124.8, 21.36 (Me); IR ν_{max} (cm^{-1}): 3026, 2916, 2857, 1580, 1492, 1441, 833, 754, 696; HRMS (APCI) [M] [$C_{22}H_{20}S$]: calculated 316.1286, found 316.1286.

Synthesis of (4-chlorophenyl)(2,2-diphenylvinyl)sulfane **3af**[18]

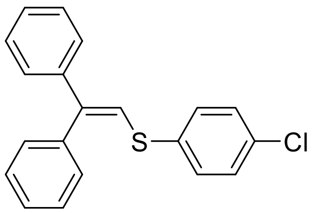

Synthesized in accordance with General Procedure c using 1,1-diphenylethylene **1a** (22 mg, 0.12 mmol, 1.2 equiv.) and *N*-[(4-chlorophenyl)thio]succinimide **2f** (24 mg, 0.1 mmol, 1.0 equiv.). The crude product was purified via preparative thin layer chromatography using hexane as eluent. The desired compound **3af** was obtained as a

colorless oil. Yield: 24 mg, 0.07 mmol, 74%. ^{1}H NMR (400 MHz, $CDCl_3$, 298 K) δ: 7.45–7.39 (m, 2H, Ar–CH), 7.39–7.29 (m, 7H, Ar–CH), 7.29–7.23 (m, 5H, Ar–CH), 6.78 (s, 1H, CH); ^{13}C NMR (101 MHz, $CDCl_3$, 298 K) δ: 142.1, 141.4, 139.1, 135.2, 132.9, 130.8, 129.8, 129.4, 128.6, 128.5, 128.1, 127.6, 127.4, 123.3. Data agrees with literature values.[18]

Synthesis of (4-bromophenyl)(2,2-diphenylvinyl)sulfane **3ag**[18]

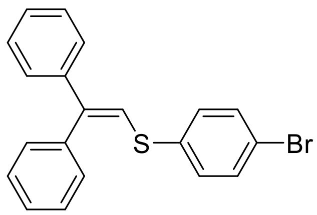

Synthesized in accordance with General Procedure c using 1,1-diphenylethylene **1a** (22 mg, 0.12 mmol, 1.2 equiv.) and *N*-[(4-bromophenyl)thio]succinimide **2g** (29 mg, 0.1 mmol, 1.0 equiv.). The crude product was purified via preparative thin layer chromatography using hexane as eluent. The desired compound **3ag** was obtained as a white solid. Yield: 26 mg, 0.07 mmol, 71%. ^{1}H NMR (400 MHz, $CDCl_3$, 298 K) δ: 7.45–7.38 (m, 4H, Ar–CH), 7.38–7.21 (m, 10H, Ar–CH), 6.76 (s, 1H, CH); ^{13}C NMR (101 MHz, $CDCl_3$, 298 K) δ: 142.3, 141.4, 139.1, 135.9, 132.3, 131.0, 129.8, 128.6, 128.5, 128.1, 127.7, 127.4, 123.1, 120.8; Melting point: 100–104 °C. NMR data agrees with literature values.[18]

Synthesis of (2-bromophenyl)(2,2-diphenylvinyl)sulfane **3ah**

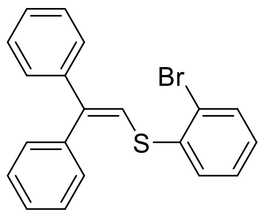

Synthesized in accordance with General Procedure c using 1,1-diphenylethylene **1a** (22 mg, 0.12 mmol, 1.2 equiv.) and *N*-[(2-bromophenyl)thio]succinimide **2h** (29 mg, 0.1 mmol, 1.0 equiv.). The crude product was purified via preparative thin layer chromatography using hexane as eluent. The desired compound **3ah** was obtained as a colorless oil. Yield: 21 mg, 0.06 mmol, 57%. ^{1}H NMR (400 MHz, $CDCl_3$, 298 K) δ: 7.52 (dd, J = 8.0, 1.4 Hz, 1H, Ar–CH), 7.45 (dd, J = 7.9, 1.6 Hz, 1H, Ar–CH), 7.43–7.26 (m, 10H, Ar–CH), 7.25–7.19 (m, 1H, Ar–CH), 7.05 (td, J = 7.8, 1.6 Hz, 1H, Ar–CH), 6.72 (s, 1H, CH); ^{13}C NMR (101 MHz, $CDCl_3$, 298 K) δ: 144.2, 141.6, 139.0, 138.2, 133.2, 129.92, 129.87, 128.54, 128.50, 128.13, 128.10, 127.83, 127.75, 127.5, 123.9, 121.9; IR ν_{max} (cm^{-1}): 3055, 3026, 2349, 1576, 1493, 1445, 1020, 748, 696; HRMS (APCI) [M] [$C_{20}H_{15}SBr$]: calculated 366.0078, found 366.0071.

Synthesis of (2,4-difluorophenyl)(2,2-diphenylvinyl)sulfane **3ai**

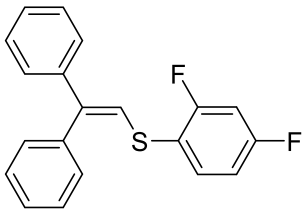

Synthesized in accordance with General Procedure c using 1,1-diphenylethylene **1a** (22 mg, 0.12 mmol, 1.2 equiv.) and *N*-[(2,4-difluorophenyl)thio]succinimide **2i** (24 mg, 0.1 mmol, 1.0 equiv.). The crude product was purified via preparative thin layer chromatography using hexane as eluent. The desired compound **3ai** was obtained as a colorless oil. Yield: 20 mg, 0.06 mmol, 62%. ^{1}H NMR (400 MHz, $CDCl_3$, 298 K) δ: 7.53–7.43 (m, 3H, Ar–CH), 7.43–7.36 (m, 3H, Ar–CH), 7.36–7.21 (m, 5H, Ar–CH), 6.96–6.85 (m, 2H, Ar–CH), 6.67 (d, *J* = 0.9 Hz, 1H, CH); ^{13}C NMR (101 MHz, $CDCl_3$, 298 K) δ: 162.8 (dd, $J_{C–F}$ = 250.3, 11.3 Hz), 161.5 (dd, $J_{C–F}$ = 249.6, 12.0 Hz) 141.8, 141.3, 138.9, 134.0 (dd, $J_{C–F}$ = 9.6, 2.7 Hz), 129.9, 128.6, 128.5, 128.1, 127.6, 127.3, 123.2, 118.7 (dd, $J_{C–F}$ = 18.2, 4.0 Hz), 112.2 (dd, $J_{C–F}$ = 21.6, 3.8 Hz), 104.8 (t, $J_{C–F}$ = 26.0 Hz); ^{19}F NMR (376 MHz, $CDCl_3$, 298 K) δ: -104.1, -109.7; IR ν_{max} (cm^{-1}): 3057, 2028, 1597, 1587, 1442, 1421, 1265, 1140, 964, 851, 771, 754, 696; HRMS (APCI) [M] [$C_{20}H_{14}F_2$ S]: calculated 324.0784, found 324.0782.

Synthesis of (2,2-diphenylvinyl)(4-(trifluoromethyl)phenyl)sulfane **3aj**[18]

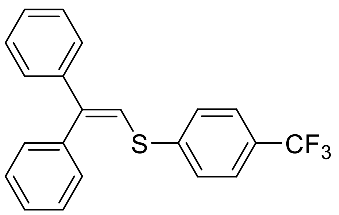

Synthesized in accordance with General Procedure c using 1,1-diphenylethylene **1a** (22 mg, 0.12 mmol, 1.2 equiv.) and *N*-[(4-trifluoromethylphenyl)thio]succinimide **2j** (28 mg, 0.1 mmol, 1.0 equiv.). The crude product was purified via preparative thin layer chromatography using hexane as eluent. The desired compound **3aj** was obtained as a colorless oil. Yield: 15 mg, 0.04 mmol, 42%. ^{1}H NMR (400 MHz, $CDCl_3$, 298 K) δ: 7.57 (d, *J* = 8.1 Hz, 2H, Ar–CH), 7.50 (d, *J* = 8.3 Hz, 2H, Ar–CH), 7.47–7.26 (m, 10H, Ar–CH), 6.85 (s, 1H, CH); ^{13}C NMR (101 MHz, $CDCl_3$, 298 K) δ: 144.1, 142.0, 141.3, 139.0, 129.8, 128.60, 128.57, 128.55 (q, $J_{C–F}$ = 32.7 Hz), 128.5, 128.3, 128.0, 127.5, 126.0 (q, $J_{C–F}$ = 4.0 Hz), 124.2 (q, $J_{C–F}$ = 271.8 Hz, CF_3), 121.1; ^{19}F NMR (376 MHz, $CDCl_3$, 298 K) δ: -62.5. Data agrees with literature values.[18]

Synthesis of (2,2-diphenylvinyl)(naphthalen-1-yl)sulfane **3ak**[20]

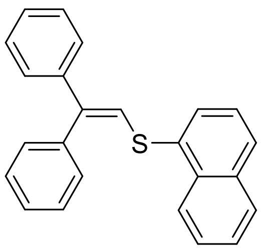

Synthesized in accordance with General Procedure c using 1,1-diphenylethylene **1a** (22 mg, 0.12 mmol, 1.2 equiv.) and *N*-[(naphthyl)thio)]succinimide **2k** (26 mg, 0.1 mmol, 1.0 equiv.). The crude product was purified via preparative thin layer chromatography using hexane as eluent. The desired compound **3ak** was obtained as a white solid. Yield: 27 mg, 0.08 mmol, 80%. ^{1}H NMR (400 MHz, $CDCl_3$, 298 K) δ: 8.44–8.35 (m, 1H, Ar–CH), 7.96–7.77 (m, 3H, Ar–CH), 7.64–7.40 (m, 8H, Ar–CH), 7.34–7.20 (m, 5H, Ar–CH), 6.86 (s, 1H, CH); ^{13}C NMR (101 MHz, $CDCl_3$, 298 K) δ: 141.6, 141.0, 139.4, 134.2, 133.6, 133.1, 130.0, 129.8, 128.7, 128.6, 128.5, 128.4, 128.0, 127.4, 127.3, 126.8, 126.5, 125.8, 125.5, 125.3; Melting point: 100–103 °C. NMR data agrees with literature values.[20]

Synthesis of 2-((2,2-diphenylvinyl)thio)thiophene **3al**[18]

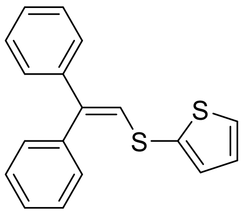

Synthesized in accordance with General Procedure c using 1,1-diphenylethylene **1a** (22 mg, 0.12 mmol, 1.2 equiv.) and *N*-[(thiophenyl)thio]succinimide **2l** (21 mg, 0.1 mmol, 1.0 equiv.). The crude product was purified via preparative thin layer chromatography using hexane as eluent. The desired compound **3al** was obtained as a yellow solid. Yield: 8 mg, 0.03 mmol, 27%. ^{1}H NMR (400 MHz, $CDCl_3$, 298 K) δ: 7.47–7.40 (m, 2H, Ar–CH), 7.39–7.29 (m, 4H, Ar–CH), 7.29–7.10 (m, 6H, Ar–CH), 7.00 (dd, J = 5.3, 3.6 Hz, 1H, Ar–CH), 6.71 (s, 1H, CH); ^{13}C NMR (101 MHz, $CDCl_3$, 298 K) δ: 141.2, 139.4, 138.9, 134.1, 132.9, 129.9, 129.5, 128.6, 128.4, 128.1, 127.8, 127.4, 127.31, 127.29; Melting point: 98–100 °C. NMR data agrees with literature values.[18]

Synthesis of cyclohexyl(2,2-diphenylvinyl)sulfane **3am**[21]

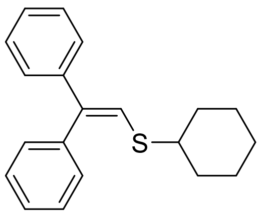

Synthesized in accordance with General Procedure c using 1,1-diphenylethylene **1a** (22 mg, 0.12 mmol, 1.2 equiv.) and *N*-[(cyclohexyl)thio]succinimide **2m** (21 mg, 0.1 mmol, 1.0 equiv.). The crude product was purified via preparative thin layer chromatography using hexane as eluent. The desired compound **3am** was obtained as a colorless oil. Yield: 24 mg, 0.08 mmol, 82%. ^{1}H NMR (400 MHz, $CDCl_3$, 298 K) δ: 7.34–7.26 (m, 2H, Ar–

CH), 7.26–7.16 (m, 5H, Ar–CH), 7.16–7.09 (m, 3H, Ar–CH), 6.58 (s, 1H, CH), 2.93–2.75 (m, 1H), 2.03–1.90 (m, 2H), 1.79–1.63 (m, 2H), 1.61–1.49 (m, 1H), 1.40–1.21 (m, 4H), 1.20–1.10 (m, 1H); ^{13}C NMR (101 MHz, $CDCl_3$, 298 K) δ: 142.4, 139.9, 138.3, 129.9, 128.4, 128.3, 127.5, 127.2, 126.9, 124.9, 46.9, 34.0, 26.2, 25.8. Data agrees with literature values.[21]

Synthesis of (4-fluorophenyl)(2-phenylallyl)sulfane **4pa**[22]

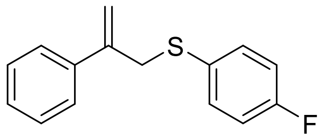

Synthesized in accordance with General Procedure c using α-methylstyrene **1p** (14 mg, 0.12 mmol, 1.2 equiv.) and *N*-[(4-fluorophenyl)thio]succinimide **2a** (23 mg, 0.1 mmol, 1.0 equiv.). The crude product was purified via preparative thin layer chromatography using hexane as eluent. The desired compound **4pa** was obtained as a colorless oil. Yield: 15 mg, 0.06 mmol, 61%. ^{1}H NMR (400 MHz, $CDCl_3$, 298 K) δ: 7.47–7.41 (m, 2H, Ar–CH), 7.40–7.28 (m, 5H, Ar–CH), 7.04–6.92 (m, 2H, Ar–CH), 5.35 (d, J = 1.1 Hz, 1H, vinylic), 5.04 (q, *J* = 1.1 Hz, 1H, vinylic), 3.91 (d, *J* = 1.1 Hz, 2H, allylic CH_2); ^{13}C NMR (101 MHz, $CDCl_3$, 298 K) δ: 162.3 (d, $J_{C–F}$ = 247.0 Hz), 143.3, 139.5, 134.1 (d, $J_{C–F}$ = 8.0 Hz), 130.8 (d, $J_{C–F}$ = 3.3 Hz), 128.6, 128.1, 126.4, 116.0 (d, $J_{C–F}$ = 21.8 Hz), 115.8 (vinylic CH_2), 41.0 (allylic CH_2); ^{19}F NMR (376 MHz, $CDCl_3$, 298 K) δ: -114.8. Data agrees with literature values.[22]

Synthesis of (4-fluorophenyl)(2-(4-fluorophenyl)allyl)sulfane **4qa**

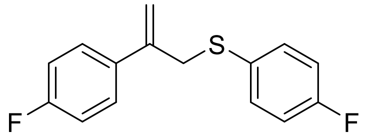

Synthesized in accordance with General Procedure c using 4-fluoro-α-methylstyrene **1q** (16 mg, 0.12 mmol, 1.2 equiv.) and *N*-[(4-fluorophenyl)thio]succinimide **2a** (23 mg, 0.1 mmol, 1.0 equiv.). The crude product was purified via preparative thin layer chromatography using hexane as eluent. The desired compound **4qa** was obtained as a colorless oil. Yield: 15 mg, 0.06 mmol, 57%. ^{1}H NMR (400 MHz, $CDCl_3$, 298 K) δ: 7.43–7.36 (m, 2H, Ar–CH), 7.35–7.28 (m, 2H, Ar–CH), 7.08–6.94 (m, 4H, Ar–CH), 5.28 (d, *J* = 1.0 Hz, 1H, vinylic), 5.01 (d, *J* = 1.1 Hz, 1H, vinylic), 3.87 (d, *J* = 1.0 Hz, 2H, allylic CH_2); ^{13}C NMR (101 MHz, $CDCl_3$, 298 K) δ: 162.7 (d, $J_{C–F}$ = 247.0 Hz), 162.4 (d, *J* = 247.0 Hz), 142.3, 135.5 (d, $J_{C–F}$ = 3.3 Hz), 134.2 (d, $J_{C–F}$ = 8.0 Hz), 130.6 (d, $J_{C–F}$ = 3.3 Hz), 128.1 (d, $J_{C–F}$ = 8.0 Hz), 116.1 (d, $J_{C–F}$ = 21.8 Hz), 115.8, 115.4 (d, $J_{C–F}$ = 21.4 Hz), 41.2 (allylic CH_2); ^{19}F NMR (376 MHz, $CDCl_3$, 298 K) δ: -114.3, -114.5; IR ν_{max} (cm^{-1}): 2926,

2347, 1750, 1601, 1589, 1508, 1489, 1221, 1155, 1090, 1013, 904, 827; HRMS (EI) [M] [$C_{15}H_{12}F_2S$]: calculated 262.06223, found 262.0621.

Synthesis of (2-(4-chlorophenyl)allyl)(4-fluorophenyl)sulfane **4ra**

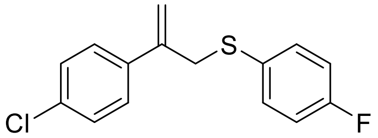

Synthesized in accordance with General Procedure c using 4-chloro-α-methylstyrene **1r** (18 mg, 0.12 mmol, 1.2 equiv.) and *N*-[(4-fluorophenyl)thio]succinimide **2a** (23 mg, 0.1 mmol, 1.0 equiv.). The crude product was purified via preparative thin layer chromatography using hexane as eluent. The desired compound **4ra** was obtained as a colorless oil. Yield: 14 mg, 0.05 mmol, 50%. ^{1}H NMR (400 MHz, $CDCl_3$, 298 K) δ: 7.41–7.26 (m, 6H, Ar–CH), 7.06–6.90 (m, 2H, Ar–CH), 5.31 (d, J = 0.9 Hz, 1H, vinylic), 5.02 (q, J = 1.0 Hz, 1H, vinylic), 3.86 (d, J = 1.0 Hz, 2H, allylic CH_2); ^{13}C NMR (101 MHz, $CDCl_3$, 298 K) δ: 162.4 (d, $J_{C\text{-}F}$ = 247.4 Hz), 142.3, 137.8, 134.3 (d, $J_{C–F}$ = 8.4 Hz), 134.0, 130.4 (d, $J_{C–F}$ = 3.3 Hz), 128.7, 127.8, 116.3, 116.1 (d, $J_{C–F}$ = 21.8 Hz), 41.0 (allylic CH_2); ^{19}F NMR (376 MHz, $CDCl_3$, 298 K) δ: -114.4; IR ν_{max} (cm^{-1}): 2922, 2850, 2324, 1736, 1589, 1489, 1396, 1225, 1155, 1091, 1011, 906, 827; HRMS (EI) [M] [$C_{15}H_{12}ClFS$]: calculated 278.03268, found 278.0328.

Synthesis of (2-(4-bromophenyl)allyl)(4-fluorophenyl)sulfane **4sa**

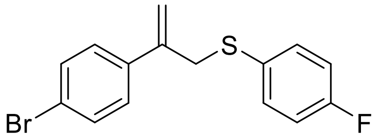

Synthesized in accordance with General Procedure c using 4-bromo-α-methylstyrene **1s** (24 mg, 0.12 mmol, 1.2 equiv.) and *N*-[(4-fluorophenyl)thio]succinimide **2a** (23 mg, 0.1 mmol, 1.0 equiv.). The crude product was purified via preparative thin layer chromatography using hexane as eluent. The desired compound **4sa** was obtained as a colorless oil. Yield: 16 mg, 0.05 mmol, 50%. ^{1}H NMR (400 MHz, $CDCl_3$, 298 K) δ: 7.50–7.45 (m, 2H, Ar–CH), 7.35–7.27 (m, 4H, Ar–CH), 7.02–6.95 (m, 2H, Ar–CH), 5.32 (s, 1H, vinylic), 5.03 (d, J = 1.0 Hz, 1H, vinylic), 3.86 (d, J = 1.1 Hz, 2H, allylic CH_2); ^{13}C NMR (101 MHz, $CDCl_3$, 298 K) δ: 162.4 (d, $J_{C–F}$ = 247.0 Hz), 142.3, 138.3, 134.3 (d, $J_{C–F}$ = 8.4 Hz), 131.7, 130.4 (d, $J_{C–F}$ = 3.3 Hz), 128.1, 122.1, 116.4, 116.1 (d, $J_{C–F}$ = 22.2 Hz), 40.9 (allylic CH_2); ^{19}F NMR (376 MHz, $CDCl_3$, 298 K) δ: -114.3; IR ν_{max} (cm^{-1}): 3090, 2922, 2309, 1587, 1489, 1225, 1009, 827; HRMS (EI) [M] [$C_{15}H_{12}BrFS$]: calculated 323.98012, found 323.9799.

Synthesis of (4-fluorophenyl)(2-(p-tolyl)allyl)sulfane **4ta**

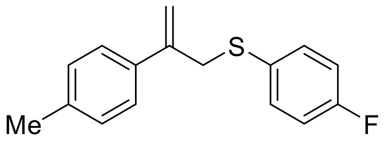

Synthesized in accordance with General Procedure c using 4-methyl-α-methylstyrene **1t** (16 mg, 0.12 mmol, 1.2 equiv.) and *N*-[(4-fluorophenyl)thio]succinimide **2a** (23 mg, 0.1 mmol, 1.0 equiv.). The crude product was purified via preparative thin layer chromatography using hexane as eluent. The desired compound **4ta** was obtained as a colorless oil. Yield: 8 mg, 0.03 mmol, 31%. ^{1}H NMR (400 MHz, $CDCl_3$, 298 K) δ: 7.37–7.29 (m, 4H, Ar–CH), 7.17 (d, *J* = 7.9 Hz, 2H, Ar–CH), 7.02–6.94 (m, 2H, Ar–CH), 5.32 (d, *J* = 1.1 Hz, 1H, vinylic), 5.00 (q, *J* = 1.2 Hz, 1H, vinylic), 3.89 (d, *J* = 1.0 Hz, 2H, allylic CH_2), 2.37 (s, 3H, Me); ^{13}C NMR (101 MHz, $CDCl_3$, 298 K) δ: 162.3 (d, $J_{C–F}$ = 247.0 Hz), 143.1, 137.9, 136.5, 134.0 (d, $J_{C–F}$ = 8.0 Hz), 130.9 (d, $J_{C–F}$ = 3.3 Hz), 129.3, 126.3, 116.0 (d, $J_{C–F}$ = 21.8 Hz), 115.0, 41.0 (allylic CH_2), 21.3 (Me); ^{19}F NMR (376 MHz, $CDCl_3$, 298 K) δ: -114.9; IR ν_{max} (cm^{-1}): 3026, 2922, 2311, 1744, 1589, 1514, 1489, 1225, 1155, 1090, 822; HRMS (EI) [M] [$C_{16}H_{15}FS$]: calculated 258.08730, found 258.0872.

Synthesis of (4-fluorophenyl)(2-(naphthalen-2-yl)allyl)sulfane **4ua**

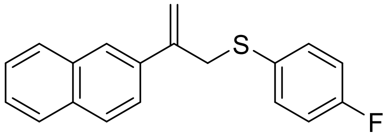

Synthesized in accordance with General Procedure c using 2-(prop-1-en-2-yl)naphthalene **1u** (20 mg, 0.12 mmol, 1.2 equiv.) and *N*-[(4-fluorophenyl)thio]succinimide **2a** (23 mg, 0.1 mmol, 1.0 equiv.). The crude product was purified via preparative thin layer chromatography using hexane as eluent. The desired compound **4ua** was obtained as colorless oil. Yield: 13 mg, 0.04 mmol, 44%. ^{1}H NMR (400 MHz, $CDCl_3$, 298 K) δ: 7.89 (d, *J* = 2.1 Hz, 1H, Ar–CH), 7.87–7.79 (m, 3H, Ar–CH), 7.59 (dd, *J* = 8.5, 1.9 Hz, 1H, Ar–CH), 7.53–7.45 (m, 2H, Ar–CH), 7.41–7.29 (m, 2H, Ar–CH), 7.05–6.93 (m, 2H, Ar–CH), 5.51 (d, *J* = 1.1 Hz, 1H, vinylic), 5.15 (d, *J* = 1.1 Hz, 1H, vinylic), 4.03 (d, *J* = 1.1 Hz, 2H, allylic CH_2); ^{13}C NMR (101 MHz, $CDCl_3$, 298 K) δ: 162.3 (d, $J_{C–F}$ = 246.7 Hz), 143.1, 136.6, 134.1 (d, $J_{C–F}$ = 8.0 Hz), 133.4, 133.1, 130.8 (d, $J_{C–F}$ = 3.3 Hz), 128.4, 128.1, 127.7, 126.4, 126.3, 125.3, 124.6, 116.3, 116.0 (d, $J_{C–F}$ = 21.8 Hz), 41.0 (allylic CH_2); ^{19}F NMR (376 MHz, $CDCl_3$, 298 K) δ: -114.6; IR ν_{max} (cm^{-1}): 2361, 2342, 1489, 1223, 1155, 1090, 1013, 893, 856, 818, 750; HRMS (EI) [M] [$C_{19}H_{15}FS$]: calculated 294.08730, found 294.0870.

Synthesis of (2-([1,1'-biphenyl]-4-yl)allyl)(4-fluorophenyl)sulfane **4va**

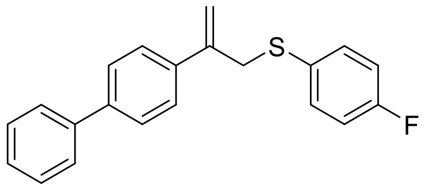

Synthesized in accordance with General Procedure c using 4-(prop-1-en-2-yl)-1,1'-biphenyl **1v** (23 mg, 0.12 mmol, 1.2 equiv.) and *N*-[(4-fluorophenyl)thio]succinimide **2a** (23 mg, 0.1 mmol, 1.0 equiv.). The crude product was purified via preparative thin layer chromatography using hexane as eluent. The desired compound **4va** was obtained as colorless oil. Yield: 12 mg, 0.04 mmol, 37%. ^{1}H NMR (400 MHz, $CDCl_3$, 298 K) δ: 7.65–7.57 (m, 4H, Ar–CH), 7.56–7.49 (m, 2H, Ar–CH), 7.49–7.42 (m, 2H, Ar–CH), 7.40–7.30 (m, 3H, Ar–CH), 7.05–6.93 (m, 2H, Ar–CH), 5.41 (d, *J* = 1.0 Hz, 1H, vinylic), 5.06 (q, *J* = 1.1 Hz, 1H, vinylic), 3.94 (d, *J* = 1.1 Hz, 2H, allylic CH_2); ^{13}C NMR (101 MHz, $CDCl_3$, 298 K) δ: 162.3 (d, $J_{C\text{–}F}$ = 247.0 Hz), 142.8, 140.9, 140.7, 138.2, 134.2 (d, $J_{C\text{–}F}$ = 8.0 Hz), 130.8 (d, $J_{C\text{–}F}$ = 3.3 Hz), 129.0, 127.5, 127.3, 127.2, 126.8, 116.1 (d, $J_{C\text{–}F}$ = 21.8 Hz), 115.8, 41.0 (allylic CH_2); ^{19}F NMR (376 MHz, $CDCl_3$, 298 K) δ: -114.6; IR ν_{max} (cm^{-1}): 2361, 2342, 1589, 1489, 1223, 1155, 1088, 1007, 905, 827, 770, 739, 696; HRMS (ES+) $[M+H]^+$ $[C_{21}H_{18}FS]^+$: calculated 321.1113, found 321.1115.

Synthesis of (4-fluorophenyl)(2-phenylbut-2-en-1-yl)sulfane **4wa**

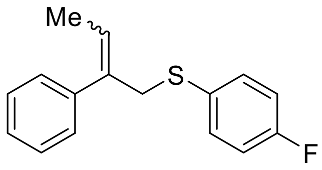

Synthesized in accordance with General Procedure c using α-ethylstyrene **1w** (16 mg, 0.12 mmol, 1.2 equiv.) and *N*-[(4-fluorophenyl)thio]succinimide **2a** (23 mg, 0.1 mmol, 1.0 equiv.). The crude product was purified via preparative thin layer chromatography using hexane as eluent. The desired compound **4wa** was obtained as a colorless oil. Yield: 17 mg, 0.07 mmol, 65%. ^{1}H NMR (400 MHz, $CDCl_3$, 298 K) δ: 7.44–7.30 (m, 6H, Ar–CH), 7.30–7.23 (m, 1H, Ar–CH), 7.02–6.94 (m, 2H, Ar–CH), 5.93 (q, *J* = 7.1 Hz, 1H, CH), 3.94 (s, 2H, CH_2), 1.64 (d, *J* = 7.0 Hz, 3H, Me); ^{13}C NMR (101 MHz, $CDCl_3$, 298 K) δ: 162.4 (d, $J_{C\text{–}F}$ = 247.0 Hz), 141.8, 135.6, 134.4 (d, $J_{C\text{–}F}$ = 8.4 Hz), 131.2 (d, $J_{C\text{–}F}$ = 3.3 Hz), 128.5, 127.3, 127.2, 126.3, 115.9 (d, $J_{C\text{–}F}$ = 21.8 Hz), 35.5 (CH_2), 14.4 (Me); ^{19}F NMR (376 MHz, $CDCl_3$, 298 K) δ: -114.7; IR ν_{max} (cm^{-1}): 3057, 3032, 2924, 2855, 2349, 1589, 1489, 1221, 1155, 1089, 827, 766, 696; HRMS (EI) [M] $[C_{16}H_{15}FS]$: calculated 258.08730, found 258.0872.

Synthesis of (4-fluorophenyl)(2-phenylhept-2-en-1-yl)sulfane **4xa**

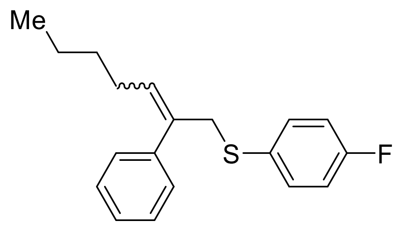

Synthesized in accordance with General Procedure c using hept-1-en-2-ylbenzene **1x** (21 mg, 0.12 mmol, 1.2 equiv.) and *N*-[(4-fluorophenyl)thio]succinimide **2a** (23 mg, 0.1 mmol, 1.0 equiv.). The crude product was purified via preparative thin layer chromatography using hexane as eluent. The desired compound **4xa** was obtained as a colorless oil. Yield: 12 mg, 0.04 mmol, 40%. ^{1}H NMR (400 MHz, $CDCl_3$, 298 K) δ: 7.36–7.29 (m, 2H, Ar–CH), 7.29–7.20 (m, 4H, Ar–CH), 7.20–7.14 (m, 1H, Ar–CH), 6.96–6.79 (m, 2H, Ar–CH), 5.74 (t, J = 7.3 Hz, 1H, CH), 3.84 (s, 2H, CH_2), 1.94 (q, J = 7.3 Hz, 2H, CH_2), 1.27–1.15 (m, 4H, CH_2), 0.84–0.76 (m, 3H, Me); ^{13}C NMR (101 MHz, $CDCl_3$, 298 K) δ: 162.3 (d, $J_{C–F}$ = 247.0 Hz), 141.9, 134.5, 134.2 (d, $J_{C–F}$ = 8.4 Hz), 133.6, 131.3 (d, $J_{C–F}$ = 3.3 Hz), 128.5, 127.2, 126.4, 115.9 (d, $J_{C–F}$ = 21.8 Hz), 35.8 (CH_2), 31.8 (CH_2), 28.6 (CH_2), 22.6 (CH_2), 14.1 (Me); ^{19}F NMR (376 MHz, $CDCl_3$, 298 K) δ: -114.8; IR ν_{max} (cm^{-1}): 2955, 2926, 2855, 2355, 2341, 1589, 1489, 1456, 1447, 1220, 1153, 1090, 1012, 827, 766, 694; HRMS (EI) [M] [$C_{19}H_{21}FS$]: calculated 300.13425, found 300.1339.

Synthesis of phenyl(2-phenylallyl)sulfane **4pc**

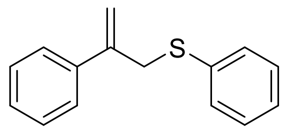

Synthesized in accordance with General Procedure c using α-methylstyrene **1p** (14 mg, 0.12 mmol, 1.2 equiv.) and *N*-[(phenyl)thio]succinimide **2c** (21 mg, 0.1 mmol, 1.0 equiv.). The crude product was purified via preparative thin layer chromatography using hexane as eluent. The desired compound **4pc** was obtained as colorless oil. Yield: 17 mg, 0.08 mmol, 75%. ^{1}H NMR (400 MHz, $CDCl_3$, 298 K) δ: 7.51–7.45 (m, 2H, Ar–CH), 7.40–7.26 (m, 7H, Ar–CH), 7.24–7.18 (m, 1H, Ar–CH), 5.41 (d, J = 1.1 Hz, 1H, vinylic), 5.20 (q, J = 1.1 Hz, 1H, vinylic), 3.99 (d, J = 1.1 Hz, 2H, allylic CH_2); ^{13}C NMR (101 MHz, $CDCl_3$, 298 K) δ: 143.3, 139.7, 136.3, 130.5, 128.9, 128.5, 128.0, 126.6, 126.4, 115.8, 39.6 (allylic CH_2); IR ν_{max} (cm^{-1}): 2359, 2344, 1229, 1088, 1024, 903, 775, 736, 691, 669; HRMS (EI) [M] [$C_{15}H_{14}S$]: calculated 226.08107, found 226.0803.

Synthesis of (2-phenylallyl)(p-tolyl)sulfane **4pb**[22]

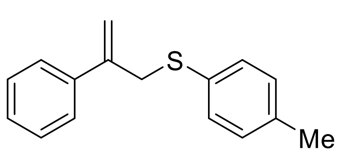

Synthesized in accordance with General Procedure c using α-methylstyrene **1p** (14 mg, 0.12 mmol, 1.2 equiv.) and *N*-[(4-methylphenyl)thio]succinimide **2b** (22 mg, 0.1 mmol, 1.0 equiv.). The crude product was purified via preparative thin layer chromatography using hexane as eluent. The desired compound **4pb** was obtained as colorless oil. Yield: 15 mg, 0.06 mmol, 62%. ^{1}H NMR (400 MHz, $CDCl_3$, 298 K) δ: 7.51–7.46 (m, 2H, Ar–CH), 7.40–7.30 (m, 3H, Ar–CH), 7.30–7.24 (m, 2H, Ar–CH), 7.14–7.08 (m, 2H, Ar–CH), 5.39 (d, *J* = 1.1 Hz, 1H, vinylic), 5.15 (q, *J* = 1.1 Hz, 1H, vinylic), 3.95 (d, *J* = 1.1 Hz, 2H, allylic CH_2), 2.34 (s, 3H, Me); ^{13}C NMR (101 MHz, $CDCl_3$, 298 K) δ: 143.4, 139.7, 136.8, 132.5, 131.4, 129.7, 128.5, 128.0, 126.4, 115.6 (vinylic CH_2), 40.4 (allylic CH_2), 21.2 (Me). Data agrees with literature values.[22]

Synthesis of (4-(tert-butyl)phenyl)(2-phenylallyl)sulfane **4pd**[22]

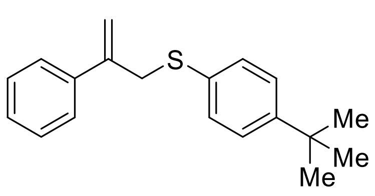

Synthesized in accordance with General Procedure c using α-methylstyrene **1p** (14 mg, 0.12 mmol, 1.2 equiv.) and 1-((4-(*tert*-butyl)phenyl)thio)pyrrolidine-2,5-dione **2d** (26 mg, 0.1 mmol, 1.0 equiv.). The crude product was purified via preparative thin layer chromatography using hexane as eluent. The desired compound **4pd** was obtained as colorless oil. Yield: 19 mg, 0.07 mmol, 67%. ^{1}H NMR (400 MHz, $CDCl_3$, 298 K) δ: 7.47–7.42 (m, 2H, Ar–CH), 7.35–7.21 (m, 7H, Ar–CH), 5.37 (d, *J* = 1.0 Hz, 1H, vinylic), 5.18 (q, *J* = 1.1 Hz, 1H, vinylic), 3.93 (d, *J* = 1.1 Hz, 2H, allylic CH_2), 1.28 (s, 9H, Me); ^{13}C NMR (101 MHz, $CDCl_3$, 298 K) δ: 149.8, 143.5, 139.8, 132.9, 130.5, 128.5, 128.0, 126.4, 126.0, 115.7, 40.0, 34.6, 31.4 (Me). Data agrees with literature values.[22]

Synthesis of (2-bromophenyl)(2-phenylallyl)sulfane **4ph**[22]

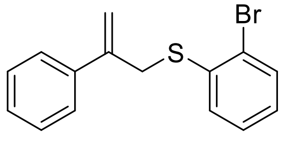

Synthesized in accordance with General Procedure c using α-methylstyrene **1p** (14 mg, 0.12 mmol, 1.2 equiv.) and *N*-[(2-bromophenyl)thio]succinimide **2h** (29 mg, 0.1 mmol, 1.0 equiv.). The crude product was purified via preparative thin layer chromatography using hexane as eluent. The desired compound **4ph** was obtained as colorless oil. Yield: 12 mg, 0.04 mmol, 39%. ^{1}H NMR (400 MHz, $CDCl_3$, 298 K) δ: 7.51–7.44 (m, 1H, Ar–CH), 7.43–7.36 (m, 2H, Ar–CH), 7.32–7.20

(m, 3H, Ar–CH), 7.19–7.12 (m, 2H, Ar–CH), 6.97 (m, 1H, Ar–CH), 5.38 (s, 1H, vinylic), 5.22 (d, *J* = 1.0 Hz, 1H, vinylic), 3.93 (d, *J* = 1.3 Hz, 2H, allylic CH_2); ^{13}C NMR (101 MHz, $CDCl_3$, 298 K) δ: 142.5, 139.6, 137.54, 133.1, 130.2, 128.6, 128.1, 127.8, 127.4, 126.3, 124.8, 116.3, 38.3 (allylic CH_2). Data agrees with literature values.[22]

Synthesis of cyclohexyl(2-phenylallyl)sulfane **4pm**[23]

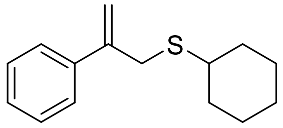

Synthesized in accordance with General Procedure c using α-methylstyrene **1p** (14 mg, 0.12 mmol, 1.2 equiv.) and *N*-(cyclohexylthio)succinimide **2m** (21 mg, 0.1 mmol, 1.0 equiv.). The crude product was purified via preparative thin layer chromatography using hexane as eluent. The desired compound **4pm** was obtained as colorless oil. Yield: 13 mg, 0.06 mmol, 56%. ^{1}H NMR (400 MHz, $CDCl_3$, 298 K) δ: 7.53–7.43 (m, 2H, Ar–CH), 7.38–7.26 (m, 3H, Ar–CH), 5.43 (d, *J* = 1.3 Hz, 1H, vinylic), 5.26 (q, *J* = 1.1 Hz, 1H, vinylic), 3.63 (d, *J* = 1.1 Hz, 2H, allylic CH_2), 2.71–2.59 (m, 1H), 2.00–1.91 (m, 2H), 1.81–1.70 (m, 2H), 1.64–1.57 (m, 1H), 1.39–1.24 (m, 5H); ^{13}C NMR (101 MHz, $CDCl_3$, 298 K) δ: 144.6, 139.9, 128.4, 127.9, 126.4, 114.7, 43.3, 35.1, 33.5, 26.2, 26.0. Data agrees with literature values.[23]

Synthesis of (2-((4-fluorophenyl)sulfonyl)ethene-1,1-diyl)dibenzene **5**[24,25]

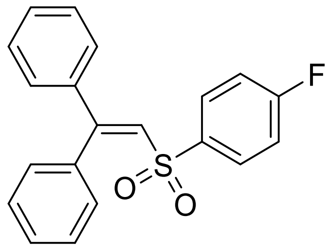

To a 20 mL round flask 2 mL of CH_2Cl_2 solution of (2,2-diphenylvinyl)(4-fluorophenyl)sulfane **3aa** (52.7 mg, 0.172 mmol) and *m*CPBA (88 mg, 0.516 mmol) was added under argon atmosphere at -78 °C. Then the reaction mixture was heated at 40 °C for 16 h. After the completion of the reaction, the solution was quenched with H_2O (5 mL) and then extracted with CH_2Cl_2 (5 mL × 3), dried over $MgSO_4$ and concentrated in rotary evaporator. The crude product was purified via preparative thin layer chromatography using hexane/ethylacetate 90:10 as eluent. The desired compound **5** was obtained as a white solid. Yield: 55.8 mg, 0.165 mmol, 96%. ^{1}H NMR (400 MHz, $CDCl_3$, 298 K) δ: 7.58–7.51 (m, 2H, Ar–CH), 7.42–7.35 (m, 2H, Ar–CH), 7.34–7.27 (m, 4H, Ar–CH), 7.24–7.18 (m, 2H, Ar–CH), 7.09–7.05 (m, 2H, Ar–CH), 7.04 (s, 1H, CH), 7.02–6.95 (m, 2H, Ar–CH); ^{13}C NMR (101 MHz, $CDCl_3$, 298 K) δ: 165.3 (d, $J_{C–F}$ = 255.4 Hz), 155.5, 139.0, 137.6 (d, $J_{C–F}$ = 3.3 Hz), 135.5, 130.6 (d, $J_{C–F}$ = 9.6

Hz), 130.6 (should be two peaks), 129.9, 129.1, 129.0, 128.8, 128.3, 128.1, 116.0 (d, $J_{C–F}$ = 22.5 Hz); ^{19}F NMR (376 MHz, $CDCl_3$, 298 K) δ: -104.74; Melting point: 111–112 °C. NMR data agrees with literature values.[24,25]

Synthesis of (2-((4-fluorophenyl)sulfinyl)ethene-1,1-diyl)dibenzene **6**

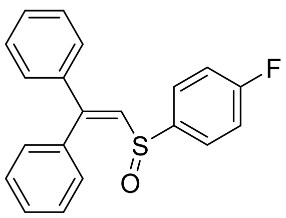

To a 20 mL round flask 2 mL of CH_2Cl_2 solution of (2,2-diphenylvinyl)(4-fluorophenyl)sulfane **3aa** (23.9 mg, 0.078 mmol) and *m*CPBA (15 mg, 0.086 mmol) was added under argon atmosphere at -78 °C then reaction mixture was allowed to stir at room temerature for 3 h. After the completion of the reaction, the solution was quenched with H_2O (5 mL) and then extracted with CH_2Cl_2 (5 mL × 3), dried over $MgSO_4$ and concentrated in rotary evaporator. The crude product was purified *via* preparative thin layer chromatography using hexane/ethylacetate 70:30 as eluent. The desired compound **6** was obtained as a sticky liquid. Yield: 23 mg, 0.069 mmol, 89%. ^{1}H NMR (400 MHz, $CDCl_3$, 298 K) δ: 7.65–7.51 (m, 2H, Ar–CH), 7.47–7.35 (m, 3H, Ar–CH), 7.34–7.22 (m, 5H, Ar–CH), 7.22–7.10 (m, 4H, Ar–CH), 6.71 (s, 1H, CH); ^{13}C NMR (101 MHz, $CDCl_3$, 298 K) δ: 164.3 (d, $J_{C–F}$ = 251.4 Hz), 153.0, 140.8 (d, $J_{C–F}$ = 3.3 Hz), 139.0, 137.0, 133.6, 130.2, 130.1, 129.4, 128.71, 128.66, 128.5, 126.9 (d, $J_{C–F}$ = 8.7 Hz), 116.8 (d, $J_{C–F}$ = 22.5 Hz); ^{19}F NMR (376 MHz, $CDCl_3$, 298 K) δ: -108.9; IR ν_{max} (cm^{-1}): 3057, 3032, 2924, 2347, 1749, 1717, 1587, 1489, 1445, 1227, 1152, 1080, 833, 756; HRMS (ES+) $[M+H]^+$ [$C_{20}H_{16}OFS$]: calculated 323.0906, found 323.0906.

Synthesis of (2,2-diphenylvinyl)(4-fluorophenyl)(imino)-l6-sulfanone **7**

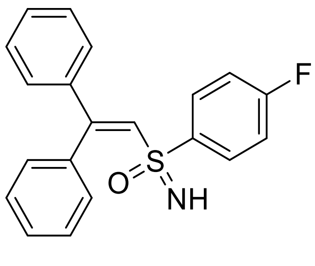

To a solution of (2,2-diphenylvinyl)(4-fluorophenyl)sulfane **3aa** (31.25 mg, 0.102 mmol) in 1 mL MeOH, PIDA (82 mg, 0.255 mmol) and NH_4COONH_2 (16 mg, 0.204 mmol) were added and stirred for 3 h at room temperature. After completion of the reaction, the solvent was removed and the crude product was purified via preparative thin layer chromatography using hexane/ethylacetate 80:20 as eluent. The desired compound **7** was obtained as a pale yellow sticky liquid. Yield: 25 mg, 0.07 mmol, 73%. ^{1}H NMR (400 MHz, $CDCl_3$, 298 K) δ: 7.66–7.59 (m, 2H, Ar–CH), 7.38–7.33 (m, 2H, Ar–CH), 7.32–7.26 (m, 4H, Ar–CH), 7.24–

7.18 (m, 2H, Ar–CH), 7.14 (s, 1H, CH), 7.08–7.03 (m, 2H), 7.00–6.93 (m, 2H), 2.80 (s, 1H, NH); ^{13}C NMR (101 MHz, $CDCl_3$, 298 K) δ: 165.1 (d, $J_{C–F}$ = 254.3 Hz), 153.1, 139.6 (d, $J_{C–F}$ = 3.2 Hz), 139.3, 135.4, 131.3, 131.1 (d, $J_{C–F}$ = 9.4 Hz), 130.3, 129.9, 128.9, 128.7, 128.2, 128.1, 115.8 (d, $J_{C–F}$ = 22.5 Hz); ^{19}F NMR (376 MHz, $CDCl_3$, 298 K) δ: -106.3; IR ν_{max} (cm^{-1}): 2922, 2851, 2378, 2347, 2322, 1744, 1717, 1587, 1543, 1491, 1231, 1217, 1092, 986, 835, 754; HRMS (ES+) $[M+H]^+$ [$C_{20}H_{17}NOFS$]: calculated 338.1015, found 338.1012.

Synthesis of methyl (E)-3-(2-((2,2-diphenylvinyl)thio)phenyl)acrylate **8**

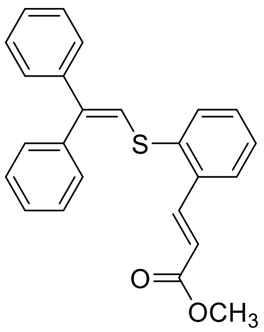

In a 20 mL Schlenk tube (2,2-diphenylvinyl)(4-fluorophenyl)sulfane **3ah** (40.14 mg, 0.131 mmol), methyl acrylate (33 mg, 0.393 mmol), DIPEA (50 mg, 0.393 mmol), and $Pd(PPh_3)_2Cl_2$ (10 mg, 0.013 mmol) in DMF (1.5 mL) were added under inert atmosphere. Then the reaction mixture was stirred in a preheated oil bath at 120 °C for 48 h. After the reaction time, the crude reaction mixture was filtered through Celite and concentrated in rotary evaporator. The crude product was purified via preparative thin layer chromatography using hexane/ethylacetate 95:5 as eluent. The desired compound **8** was obtained as a sticky liquid. Yield: 35 mg, 0.095 mmol, 73%. ^{1}H NMR (400 MHz, $CDCl_3$, 298 K) δ: 8.09 (d, *J* = 15.9 Hz, 1H, vinylic), 7.53–7.45 (m, 2H, Ar–CH), 7.40–7.33 (m, 2H, Ar–CH), 7.33–7.26 (m, 4H, Ar–CH), 7.26–7.12 (m, 6H, Ar–CH), 6.60 (s, 1H, CH), 6.30 (d, *J* = 15.9 Hz, 1H, vinylic), 3.71 (s, 3H, OMe); ^{13}C NMR (101 MHz, $CDCl_3$, 298 K) δ: 167.2, 142.7, 142.2, 141.4, 139.1, 137.5, 135.4, 131.9, 130.6, 129.9, 128.6, 128.5, 128.1, 127.8, 127.6, 127.44, 127.39, 123.8, 120.1, 51.9 (Me); IR ν_{max} (cm^{-1}): 3055, 3024, 2947, 2374, 2345, 1716, 1632, 1491, 1460, 1440, 1317, 1265, 1198, 1171, 1034, 978, 756, 698, 625; HRMS (ES+) $[M+H]^+$ [$C_{24}H_{21}O_2S$]: calculated 373.1262, found 373.1262.

Synthesis of [1,1'-biphenyl]-2-yl(2,2-diphenylvinyl)sulfane **9**

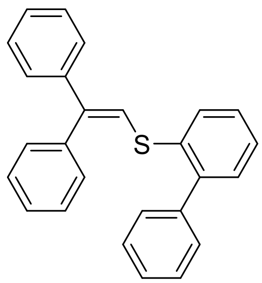

In a 20 mL Schlenk tube (2,2-diphenylvinyl)(4-fluorophenyl)sulfane **3ah** (40.14 mg, 0.131 mmol), phenyl boronic acid (19 mg, 0.157 mmol), K_2CO_3 (54 mg, 0.393 mmol), and $Pd(PPh_3)_2Cl_2$ (5 mg, 0.006 mmol) in dioxane/H_2O (1.5 mL/0.5 mL) were added under inert atmosphere. Then the reaction mixture was placed into a preheated oil bath at 100 °C for 24 h. After the reaction time, the crude reaction mixture was filtered through Celite and concentrated in a rotary evaporator. The crude product was purified via preparative thin layer chromatography using hexane as eluent. The desired compound **9** was obtained as a sticky liquid. Yield: 42 mg, 0.115mmol, 88%. ^{1}H NMR (400 MHz, $CDCl_3$, 298 K) δ: 7.67–7.59 (m, 1H, Ar–CH), 7.44–7.31 (m, 11H, Ar–CH), 7.30–7.22 (m, 5H, Ar–CH), 7.20–7.13 (m, 2H, Ar–CH), 6.70 (d, *J* = 1.5 Hz, 1H, CH); ^{13}C NMR (101 MHz, $CDCl_3$, 298 K) δ: 142.8, 142.0, 141.9, 140.6, 139.3, 135.4, 130.8, 130.7, 130.0, 129.6, 128.4, 128.3, 128.2 (x2), 127.8, 127.6, 127.4 (x2), 127.0, 124.6; IR ν_{max} (cm^{-1}): 3055, 3026, 2378, 2347, 1744, 1584, 1495, 1462, 1441, 1074, 1009, 939, 908, 772, 748, 696; HRMS (ES+) $[M+H]^+$ [$C_{26}H_{21}S$]: calculated 365.1364, found 365.1367.

3. Control Experiments

***Radical trapping experiment with radical scavengers*:** In the glovebox, a glass microwave vial was charged with 1,1-diphenylethylene **1a** (22 mg, 0.12 mmol, 1.2 equiv.) and dry CH_2Cl_2 (0.5 mL). This was then added to a separate vial containing *N*-[(4-fluorophenyl)thio]succinimide **2a** (23 mg, 0.1 mmol, 1.0 equiv.), butylated hydroxytoluene (BHT) (66 mg, 0.3 mmol, 3.0 equiv.) and $B(C_6F_5)_3$ (0.01 mmol, 10 mol%) in dry CH_2Cl_2 (0.5 mL). The reaction was stirred at 45 °C for 3 h. After the reaction time, the desired product was isolated in 62% (19 mg, 0.06 mmol). When chloromethylcyclopropane (27mg, 0.3 mmol, 3.0 equiv.) was used instead of BHT, the desired product was isolated in 55% (17 mg, 0.06 mmol).

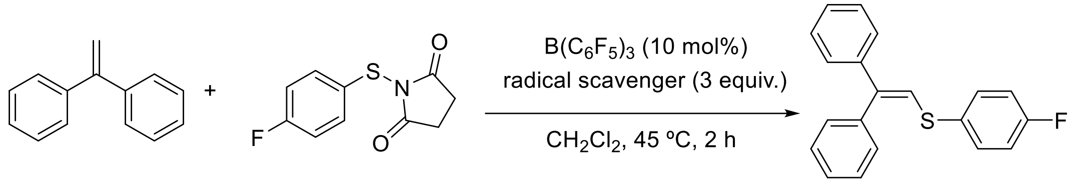

1a   2a   3aa
BHT (62%)
chloromethylcyclopropane (55%)

Experiments with other sulfenylating agents**:** In the glovebox, a glass microwave vial was charged with 1,1-diphenylethylene **1a** (22 mg, 0.12 mmol, 1.2 equiv.) in dry CH_2Cl_2 (0.5 mL). This was then added to a separate vial containing *p*-tolyl disulfide (25 mg, 0.1 mmol, 1.0 equiv.) and $B(C_6F_5)_3$ (0.01 mmol, 10 mol%) in dry CH_2Cl_2 (0.5 mL). The reaction was then stirred for 3 h at 45 °C. After the reaction time, no desired product was observed. When the reaction was carried out with 4-methylthiophenol (12.4 mg, 0.1 mmol, 1.0 equiv.) instead of *p*-tolyl disulfide, no desired product was observed.

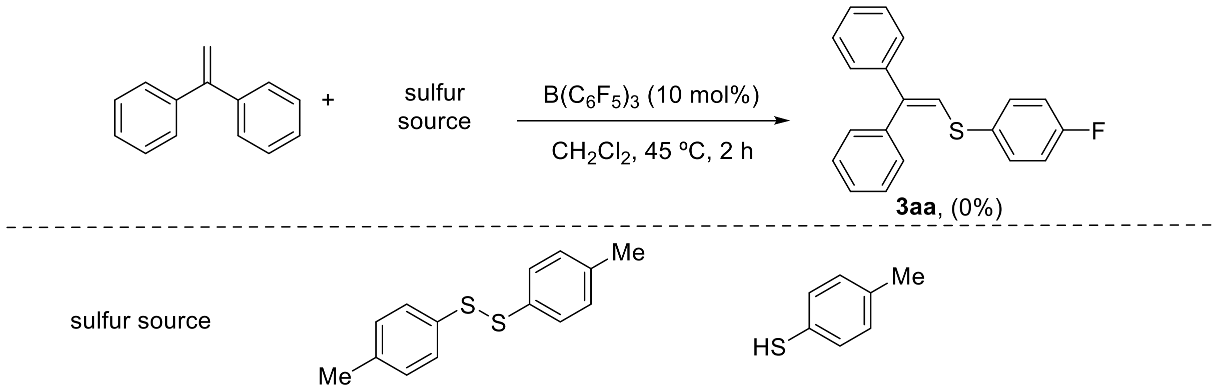

4. Computational Data

4.1 Computational Details

Density functional theory (DFT) calculations were performed using the Gaussian 09 software.[26] Geometry optimizations and frequency analyses were carried out with the M06-2X[27] functional and the 6-31G(d)[28] basis set for all atoms within the SMD[29] solvation model to simulate the solvation effects of dichloromethane. The optimized geometries were confirmed through frequency calculations, identifying them as either minima (no imaginary frequencies) or transition states (one imaginary frequency) at the same computational level. To refine electronic energies, single-point energy calculations were conducted at the SMD/M06-2X/def2-TZVP[30] level of

theory. Gibbs free energies were estimated by adding entropy corrections to these single-point energies. A correction factor of 1.89 kcal mol^{-1} was applied to adjust the relative free energies from the 1 atm to 1 M standard state.[31] All Gibbs energies in solution presented in the text are in kilocalories per mole, with bond lengths measured in angstroms. The 3D molecular structures were visualized using CYLview.[32]

Scheme S1. Calculated transition state and reaction energies for the formation of Csp^2–S and Csp^3–S products (**3oa** and **3oa'**) at the SMD/M06-2X/def2-TZVP//SMD/M06-2X/6-31G(d) level of theory using **2a** and 4-isopropenylanisole alkene **1o**.

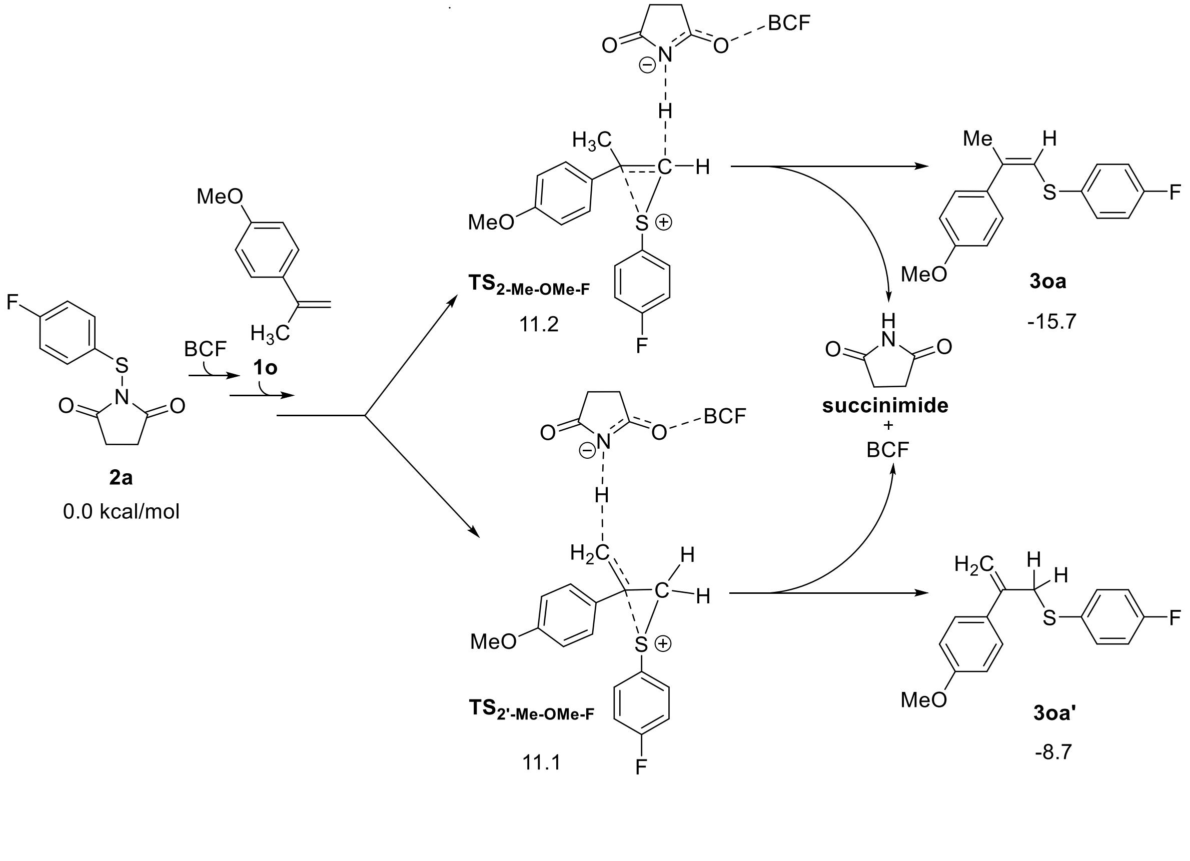

4.2 Cartesian coordinates and total energies for the calculated structures

4-Methylbenzenethiosuccinimide (2b)

E(SMD/M06-2X/6-31g(d)) = -1028.930918 au

H(SMD/M06-2X/6-31g(d)) = -1028.713619 au

G(SMD/M06-2X/6-31g(d)) = -1028.76961 au

E(SMD/M06-2X/def2-TZVP//SMD/M06-2X/6-31g(d)) = -1029.212294 au

C -2.20538300 -1.16899600 0.22902200

C	-3.16624500	-0.77273500	1.33249900
C	-3.12386500	0.75623600	1.38468200
C	-2.18027000	1.17603000	0.27498600
H	-4.15458200	-1.16570900	1.07941400
H	-2.73233800	1.14277700	2.32886100
O	-1.85590700	2.29491300	-0.03378200
O	-1.89284000	-2.28169500	-0.11281200
N	-1.71565600	0.01023500	-0.35531100
H	-4.09537600	1.22467900	1.20959700
H	-2.84454400	-1.24929200	2.26136200
S	-0.63584000	0.02440300	-1.68644100
C	0.92955800	0.00684500	-0.82787100
C	1.52184600	-1.20895000	-0.48261400
C	1.56782100	1.21095400	-0.51824600
C	2.74745400	-1.21443100	0.17747200
H	1.01879900	-2.14122700	-0.72179700
C	2.79273900	1.19035000	0.13890000
H	1.09944900	2.15373000	-0.78514800
C	3.40087600	-0.01960400	0.49316800
H	3.20506900	-2.16173600	0.45076900
H	3.28746100	2.12754100	0.38195100
C	4.73652100	-0.02671600	1.18854700
H	4.94704000	-1.00329800	1.63215600
H	5.54316000	0.20229200	0.48286700
H	4.77255100	0.72850500	1.97967800

$B(C_6F_5)_3$

E(SMD/M06-2X/6-31g(d)) = -2207.513247 au

H(SMD/M06-2X/6-31g(d)) = -2207.326569 au

G(SMD/M06-2X/6-31g(d)) = -2207.414573 au

E(SMD/M06-2X/def2-TZVP//SMD/M06-2X/6-31g(d)) = -2208.48246717 au

B	-0.00030800	0.00184700	-0.00087200

C	-1.37482600	0.75831800	0.00129600
C	-1.55085800	1.99041500	-0.63468900
C	-2.75939900	2.66707400	-0.65301700
C	-3.85391500	2.11914400	-0.00016300
C	-3.72904300	0.90219600	0.65403800
C	-2.50916500	0.24623200	0.63739100
C	1.34280600	0.81308600	-0.00270000
C	2.49686200	0.34706700	-0.63875500
C	3.68954400	1.05138900	-0.65466900
C	3.76556800	2.27188100	0.00034800
C	2.64999900	2.77508500	0.65357000
C	1.46942000	2.05070200	0.63435400
C	0.03128500	-1.56701600	-0.00054300
C	1.04118300	-2.29394800	0.63596900
C	1.08068800	-3.67846700	0.65475900
C	0.08862300	-4.39450600	0.00077800
C	-0.93177100	-3.71984800	-0.65362400
C	-0.94838000	-2.33484300	-0.63627900
F	-0.53554100	2.57064600	-1.27961200
F	-2.87802400	3.83308500	-1.28443500
F	-5.01597400	2.75672400	-0.00077100
F	-4.77666900	0.37712600	1.28581300
F	-2.45413800	-0.92166500	1.28281400
F	2.48908100	-0.82200300	-1.28434500
F	4.75764700	0.56890300	-1.28613000
F	4.90114200	2.95551000	0.00179300
F	0.43170400	2.58948400	1.27968400
F	-1.95839600	-1.74482400	-1.28072700
F	-1.88215500	-4.40480900	-1.28600800
F	0.11556000	-5.71976200	0.00086700

F	2.05872100	-4.32358600	1.28683900
F	2.02635200	-1.66295600	1.28009700
F	2.72178000	3.94445800	1.28589600

I

E(SMD/M06-2X/6-31g(d)) = -3236.479659 au

H(SMD/M06-2X/6-31g(d)) = -3236.072078 au

G(SMD/M06-2X/6-31g(d)) = -3236.193261 au

E(SMD/M06-2X/def2-TZVP//SMD/M06-2X/6-31g(d)) = -3237.722482 au

B	-0.86801200	0.13232400	-0.08081500
C	0.21562300	0.93234700	-1.00095700
C	0.40794400	2.30684400	-1.07272600
C	1.47255300	2.88914600	-1.75688800
C	2.39683100	2.08597700	-2.39913700
C	2.25115800	0.70695400	-2.35486900
C	1.17671800	0.17516200	-1.66661400
C	-1.87054100	1.07158100	0.78433200
C	-2.04730600	1.05229300	2.16148600
C	-2.99130000	1.84103300	2.81093500
C	-3.81144800	2.67831000	2.07504900
C	-3.68267300	2.71615900	0.69337800
C	-2.73313600	1.91215000	0.08783400
C	-1.78302500	-0.97022800	-0.85335300
C	-1.90523300	-1.11542100	-2.23101000
C	-2.77921800	-2.01972100	-2.82395100
C	-3.58440800	-2.81990000	-2.02999200
C	-3.50619000	-2.70606300	-0.64969200
C	-2.62876200	-1.78409800	-0.10372200
F	-0.41528000	3.16844700	-0.46127700
F	1.61107100	4.21499900	-1.78612800
F	3.44789400	2.62542100	-3.01552200
F	3.16395600	-0.07640700	-2.93012300

F	1.09548800	-1.16978500	-1.62166000
F	-1.32078000	0.24818800	2.95324600
F	-3.11972200	1.78432600	4.13885400
F	-4.71972600	3.43786100	2.68528600
F	-4.46986200	3.51744000	-0.02758900
F	-2.63995100	1.97028900	-1.25166300
F	-2.57573700	-1.72497800	1.24156800
F	-4.26721500	-3.47545000	0.12946000
F	-4.41980900	-3.69378900	-2.58492700
F	-2.85136300	-2.11891200	-4.15178000
F	-1.17387300	-0.37609800	-3.07530000
C	2.10714000	-3.12913600	2.10229700
C	1.23816100	-3.95999500	1.19079800
C	0.17392200	-2.99906600	0.65292000
C	0.59172300	-1.65111300	1.16972300
H	1.87233000	-4.36620800	0.39839000
H	-0.81647300	-3.21539100	1.06240400
O	0.10625800	-0.52403900	0.95645900
O	3.03886400	-3.45231200	2.78030900
N	1.64308500	-1.77541900	2.01008800
H	0.10426300	-2.97766600	-0.43551700
H	0.81895400	-4.79514300	1.75541300
S	2.39946300	-0.46034600	2.84437400
C	3.77118400	-0.16755500	1.74231900
C	3.65168300	0.76496500	0.70913200
C	4.96897700	-0.86226500	1.93218900
C	4.73323800	0.98863300	-0.13817600
H	2.72019500	1.30933400	0.56980700
C	6.03972800	-0.62618500	1.07786300
H	5.05001100	-1.58823600	2.73514700

C	5.93822200	0.30032700	0.03407500
H	4.64592600	1.70902400	-0.94809500
H	6.97055200	-1.16872300	1.22159600
C	7.11315200	0.57297600	-0.86544800
H	6.78698100	0.95160900	-1.83777900
H	7.77130700	1.32641800	-0.41729000
H	7.70792500	-0.33099600	-1.02351700

II-a

E(SMD/M06-2X/6-31g(d)) = -2567.620891 au

H(SMD/M06-2X/6-31g(d)) = -2567.344923 au

G(SMD/M06-2X/6-31g(d)) = -2567.448673 au

E(SMD/M06-2X/def2-TZVP//SMD/M06-2X/6-31g(d)) = -2568.742588 au

B	-0.04485300	-0.03277100	0.37420400
C	-1.43885700	-0.63055100	-0.25379600
C	-2.05496200	-1.70389800	0.38532700
C	-3.25879700	-2.26000700	-0.01805700
C	-3.91451100	-1.72777800	-1.11820400
C	-3.35149200	-0.65364000	-1.78413700
C	-2.14105900	-0.13068500	-1.34259800
C	1.11526200	-1.17875000	0.58620800
C	1.15460900	-2.44127900	0.00718900
C	2.19992900	-3.33763500	0.19539600
C	3.27898200	-2.97588200	0.98348500
C	3.29811300	-1.71923300	1.56681200
C	2.23510100	-0.85421200	1.34959200
C	0.68573300	1.10151200	-0.56491900
C	1.04507800	0.75252100	-1.86389700
C	1.75363200	1.57632800	-2.72107100
C	2.15707300	2.82668400	-2.27315100
C	1.83790300	3.21841100	-0.98541100
C	1.12370400	2.35479900	-0.16005200

F	-1.46368500	-2.27269900	1.44829000
F	-3.78743100	-3.30072900	0.63194700
F	-5.07431500	-2.24370300	-1.52526600
F	-3.97700700	-0.12212400	-2.83886200
F	-1.69648000	0.93771400	-2.02770800
F	0.16123900	-2.87521000	-0.78623300
F	2.17596900	-4.54253000	-0.38303600
F	4.29178500	-3.82338700	1.17084800
F	4.34174000	-1.34857100	2.31521300
F	2.34314900	0.36446400	1.90161100
F	0.85837200	2.82027200	1.07391400
F	2.22018500	4.41985700	-0.54244400
F	2.83795700	3.64264300	-3.07887700
F	2.05507700	1.18477700	-3.96254200
F	0.68302300	-0.45183200	-2.34131000
C	-2.08971000	2.94762900	3.32218900
C	-2.54759100	3.30589300	1.90623100
C	-2.07885600	2.10865400	1.08893500
C	-1.14383700	1.43079500	2.07506400
H	-3.62367900	3.48625100	1.88184700
H	-1.58837900	2.34992400	0.14320300
O	-0.33852800	0.46547700	1.76342700
O	-2.42026000	3.54428500	4.32666200
N	-1.21036300	1.86334900	3.30304200
H	-2.90173200	1.41982400	0.86835200
H	-2.03798300	4.22779100	1.60684400

II-c-Me (Thiiranium ion)

E(SMD/M06-2X/6-31g(d)) = -1017.642663 au

H(SMD/M06-2X/6-31g(d)) = -1017.338811 au

G(SMD/M06-2X/6-31g(d)) = -1017.399526 au

E(SMD/M06-2X/def2-TZVP//SMD/M06-2X/6-31g(d)) = -1017.902246 au

S	-0.00277400	-0.61059900	-1.03592500
C	1.66953700	-0.25038600	-0.55152800
C	2.57794900	-1.27906300	-0.30449500
C	2.05652700	1.08991200	-0.50751300
C	3.89090600	-0.94826700	0.00912600
H	2.27261000	-2.32018100	-0.36073300
C	3.37517900	1.39565300	-0.19316100
H	1.34094500	1.88149600	-0.71228900
C	4.30736900	0.38645600	0.07313100
H	4.60732900	-1.74179300	0.20093700
H	3.68583600	2.43581900	-0.15344100
C	5.72406500	0.73204600	0.44095300
H	6.40492700	-0.08997400	0.20676800
H	6.06020700	1.62891900	-0.08617000
H	5.79967500	0.93289800	1.51582600
C	-4.82045500	-0.56991600	0.06508500
C	-3.51087800	-0.97584500	0.31341900
C	-2.47175700	-0.04337800	0.26530100
C	-2.75037900	1.29543900	-0.03507400
C	-4.05587600	1.69233000	-0.29363900
C	-5.09329900	0.75977600	-0.24179900
H	-5.62523600	-1.29653100	0.11217500
H	-3.30811600	-2.01230000	0.56685600
H	-1.94102100	2.02063500	-0.08258700
H	-4.26588900	2.72891500	-0.53685300
H	-6.11357100	1.07235700	-0.44103600
C	-1.07507100	-0.45513600	0.60047600
C	-0.60559000	-1.79635400	0.24795700
C	-0.39247100	0.28223900	1.72032300
H	-0.97638000	0.07172200	2.62395500

H	0.63043100	-0.06173200	1.88186000
H	-0.40220000	1.36205800	1.55486300
H	-1.28874000	-2.51461400	-0.19719200
H	0.20903900	-2.22114600	0.83050000

II-c-Ph

E(SMD/M06-2X/6-31g(d)) = -1209.312396 au

H(SMD/M06-2X/6-31g(d)) = -1208.950718 au

G(SMD/M06-2X/6-31g(d)) = -1209.018017 au

E(SMD/M06-2X/def2-TZVP//SMD/M06-2X/6-31g(d)) = -1209.635252 au

S	-0.11517900	-2.33510000	-0.24479100
C	1.47401000	-1.54750700	-0.31396800
C	2.42707000	-1.76968400	0.68698100
C	1.77402700	-0.70508300	-1.38758000
C	3.65737600	-1.12922400	0.62025300
H	2.20213800	-2.43857200	1.51369500
C	3.01395400	-0.07687800	-1.44594300
H	1.03698100	-0.53940900	-2.16798400
C	3.96660700	-0.26728100	-0.44029000
H	4.39402900	-1.29971700	1.40105800
H	3.24194900	0.58078700	-2.28093600
C	5.28215800	0.46180300	-0.47743100
H	5.21376500	1.40041400	0.08585600
H	6.08026800	-0.13443800	-0.02670000
H	5.56813700	0.71222400	-1.50235800
C	-4.62857500	1.36708300	-0.12559200
C	-3.29836900	1.22629100	0.24256600
C	-2.68184900	-0.04545900	0.22298300
C	-3.44916300	-1.16725800	-0.16709600
C	-4.76620200	-1.01238300	-0.55816800
C	-5.35979800	0.25375500	-0.53441600
H	-5.09768000	2.34420600	-0.08245000

H	-2.74758800	2.08771500	0.60443900
H	-2.99946200	-2.15413800	-0.20845900
H	-5.33760100	-1.87628200	-0.88056500
H	-6.39906000	0.36683700	-0.82775300
C	-1.31926100	-0.19151200	0.68375400
C	-0.87470800	-1.49248600	1.22943700
H	-1.69469700	-2.12435300	1.56818100
H	-0.12403000	-1.42113100	2.01440600
C	-0.36184200	0.87490400	0.56287000
C	-0.44243600	1.78182500	-0.52452300
C	0.73256000	0.98003900	1.45531500
C	0.52558400	2.75616300	-0.69870600
H	-1.23031500	1.66796400	-1.26148500
C	1.68126000	1.97319000	1.28595900
H	0.81202800	0.31762800	2.31009400
C	1.58284800	2.85672400	0.20801700
H	0.46924700	3.42828700	-1.54813300
H	2.50323100	2.05867700	1.98903600
H	2.34002800	3.62275500	0.07018000

Succinimide

E(SMD/M06-2X/6-31g(d)) = -360.5336928 au

H(SMD/M06-2X/6-31g(d)) = -360.433872 au

G(SMD/M06-2X/6-31g(d)) = -360.471778 au

E(SMD/M06-2X/def2-TZVP//SMD/M06-2X/6-31g(d)) = -360.6868636 au

H	-0.00005500	-1.97517600	0.00004500
C	-1.16564600	-0.21121300	-0.00000700
C	-0.76574000	1.25525300	0.00003300
C	0.76573900	1.25524600	-0.00002600
C	1.16564800	-0.21122400	-0.00000500
H	-1.19894400	1.73238300	-0.88258600
H	1.19897900	1.73238000	0.88257100

O	2.28322100	-0.67272500	0.00002700
O	-2.28321600	-0.67272700	0.00000400
N	-0.00000100	-0.95986000	-0.00003400
H	1.19893000	1.73232900	-0.88267500
H	-1.19894300	1.73235400	0.88266200

II-Me

E(SMD/M06-2X/6-31g(d)) = -3585.29254 au

H(SMD/M06-2X/6-31g(d)) = -3584.710085 au

G(SMD/M06-2X/6-31g(d)) = -3584.851791 au

E(SMD/M06-2X/def2-TZVP//SMD/M06-2X/6-31g(d)) = -3586.663235 au

B	-1.67021600	-0.10415600	-0.05579100
C	-3.02236300	-0.74505500	0.61066400
C	-3.46757600	-1.98457500	0.15716600
C	-4.55979400	-2.65574500	0.68325200
C	-5.26616200	-2.08101800	1.72955200
C	-4.86311800	-0.85201000	2.22159600
C	-3.76060500	-0.21655100	1.66158300
C	-1.76655600	0.04088400	-1.69229500
C	-2.90785000	-0.06608800	-2.47919500
C	-2.90627400	0.10820600	-3.85855600
C	-1.72604600	0.42244900	-4.51052400
C	-0.56512400	0.56720800	-3.76853600
C	-0.61546200	0.38845500	-2.39428700
C	-1.30879400	1.40933500	0.46931500
C	-2.23285700	2.42239300	0.22549500
C	-2.00479400	3.76247400	0.48832800
C	-0.78155700	4.14364900	1.02249600
C	0.16928100	3.17586200	1.28949500
C	-0.10422200	1.84184000	1.00494400
F	-2.83476300	-2.58254500	-0.86480300
F	-4.93978800	-3.83885600	0.19408700

F	-6.31903200	-2.70586500	2.25480600
F	-5.52277000	-0.29592200	3.24142600
F	-3.41343900	0.94469900	2.24394000
F	-4.10443800	-0.34397900	-1.93871900
F	-4.03591400	-0.02204900	-4.55968100
F	-1.70847900	0.59342100	-5.83235600
F	0.58080100	0.89078900	-4.37553200
F	0.53481100	0.60107700	-1.73139800
F	0.88863900	0.97266600	1.29132600
F	1.34677800	3.53353700	1.81280800
F	-0.52471800	5.42678900	1.27775100
F	-2.93364200	4.68502400	0.22764100
F	-3.42768400	2.10070500	-0.29964400
C	1.24067500	-2.46469500	2.73998900
C	0.23125300	-1.75227800	3.63657800
C	-0.79839900	-1.23266200	2.64086400
C	-0.09829600	-1.49032200	1.31852800
H	-0.16872800	-2.43873000	4.38521300
H	-1.07489200	-0.18163500	2.76173400
O	-0.51879900	-1.06304300	0.17340000
O	2.17838700	-3.13025000	3.14476800
N	0.99130800	-2.21157900	1.39468700
H	-1.71986200	-1.82225600	2.66555500
H	0.74993900	-0.94276100	4.16054000
S	5.35181300	-0.49432800	0.67031900
C	4.90130800	1.22283300	0.53222100
C	4.56731500	1.94651400	1.67921900
C	4.93227400	1.82819100	-0.72173600
C	4.22840500	3.28498500	1.55076600
H	4.56633000	1.47137600	2.65623800

C	4.57774200	3.17080000	-0.82829000
H	5.20780700	1.25872700	-1.60501300
C	4.20930300	3.91111200	0.29763800
H	3.95640900	3.85347500	2.43585600
H	4.57703300	3.64461800	-1.80576900
C	3.77652500	5.34713900	0.18685900
H	4.40423500	5.99333800	0.80917000
H	3.82925500	5.70254600	-0.84475900
H	2.74493800	5.45734600	0.53949800
C	4.18321700	-5.24079800	-0.24770200
C	3.85951100	-3.98783500	0.26984900
C	4.17879700	-2.83880000	-0.45906700
C	4.81352600	-2.94637100	-1.70092200
C	5.14397200	-4.19831400	-2.20378400
C	4.82833000	-5.34732900	-1.47671100
H	3.92879200	-6.13409600	0.31412800
H	3.33460300	-3.90004300	1.21933200
H	5.06173200	-2.04828300	-2.26178100
H	5.64785100	-4.28015000	-3.16181900
H	5.08543600	-6.32549100	-1.87208100
C	3.76191600	-1.50336900	0.06371500
C	3.86367500	-1.24520200	1.49555900
C	2.80508600	-0.70477200	-0.76860300
H	1.88422500	-1.29625800	-0.80543500
H	2.56298500	0.25621700	-0.31422500
H	3.17535600	-0.56352700	-1.78761900
H	4.15968800	-2.04556100	2.16730100
H	3.21827200	-0.48008200	1.92198200

II-Ph

E(SMD/M06-2X/6-31g(d)) = -3776.962253 au

H(SMD/M06-2X/6-31g(d)) = -3776.324174 au

G(SMD/M06-2X/6-31g(d)) = -3776.471034 au

E(SMD/M06-2X/def2-TZVP//SMD/M06-2X/6-31g(d)) = -3778.397659 au

B	-1.95608100	-0.20789000	-0.05182700
C	-3.16850500	-0.92140100	-0.89195500
C	-3.16956500	-0.79975400	-2.27930500
C	-4.10197800	-1.40104400	-3.10907300
C	-5.10627400	-2.17554400	-2.54745500
C	-5.15157800	-2.32862800	-1.17259300
C	-4.19343600	-1.70578500	-0.38015100
C	-1.85774700	1.40558300	-0.36020400
C	-2.88993500	2.19141900	-0.86501600
C	-2.79628600	3.56697200	-1.03749700
C	-1.63249300	4.22365300	-0.67920800
C	-0.58173800	3.49162400	-0.15116600
C	-0.71228900	2.11704100	-0.01037700
C	-2.08676100	-0.33077100	1.58181900
C	-3.18121000	0.26578600	2.20364100
C	-3.35380200	0.33370300	3.57517200
C	-2.38602800	-0.21477000	4.40668700
C	-1.28129300	-0.82602100	3.84217700
C	-1.15753300	-0.87395000	2.45748900
F	-2.23349900	-0.04727000	-2.88173000
F	-4.04672900	-1.24050900	-4.43357100
F	-6.01194700	-2.76900400	-3.32338700
F	-6.10428000	-3.08315200	-0.61873100
F	-4.28871000	-1.95210400	0.93755200
F	-4.06870800	1.65429100	-1.21508800
F	-3.82335800	4.25951300	-1.53607000
F	-1.52685600	5.54394400	-0.82520600
F	0.52941800	4.12036600	0.23621600
F	0.34627400	1.48134100	0.53116200

F	-0.05918300	-1.51649500	2.00505700
F	-0.34201700	-1.36852900	4.62233000
F	-2.52703400	-0.15714200	5.73022700
F	-4.43107000	0.91652300	4.10600100
F	-4.15003400	0.80176600	1.44195500
C	0.98971700	-3.77585600	-0.69017700
C	-0.26995100	-4.32961300	-0.03094100
C	-1.25365800	-3.17129200	-0.15632000
C	-0.32296000	-2.03422400	-0.53423500
H	-0.58745600	-5.25447400	-0.51490300
H	-1.83534000	-2.95321200	0.74225300
O	-0.64410200	-0.78094600	-0.54208500
O	1.96958700	-4.42834100	-0.99045300
N	0.88385400	-2.39604900	-0.87749000
H	-1.95916500	-3.32764300	-0.97888800
H	-0.03469400	-4.55096400	1.01539500
S	4.06967800	0.48722300	-2.15980500
C	4.86583700	-0.99097100	-1.59008400
C	4.19621500	-2.19719400	-1.39163800
C	6.24256400	-0.88199100	-1.37440000
C	4.92460200	-3.29856300	-0.95725600
H	3.12802100	-2.29938000	-1.55935300
C	6.95069000	-2.00050700	-0.95340400
H	6.75558100	0.06332800	-1.53059500
C	6.30273700	-3.21961800	-0.72946000
H	4.39553500	-4.23334300	-0.79580300
H	8.02143000	-1.92143200	-0.78633400
C	7.06262500	-4.41617600	-0.22602700
H	6.97760100	-4.49047400	0.86440400
H	6.66451700	-5.34251700	-0.64920100

H	8.12530300	-4.34635800	-0.47285200
C	3.48489200	4.87623400	0.52091200
C	3.46895300	3.48528100	0.57534300
C	3.15334100	2.73660200	-0.56546800
C	2.84128000	3.41232600	-1.75632800
C	2.86049100	4.79880400	-1.80543500
C	3.18595200	5.53613100	-0.66634800
H	3.72603200	5.44179900	1.41543200
H	3.67960900	2.98674500	1.51440700
H	2.59580100	2.86454200	-2.66269100
H	2.62409000	5.30585900	-2.73555200
H	3.20035500	6.62100400	-0.70687600
C	3.12835900	1.25006500	-0.50100900
C	2.35462700	0.47388000	-1.47583000
H	1.67917600	0.99982600	-2.14760500
H	2.01743200	-0.53029700	-1.20708600
C	3.55259200	0.54036300	0.73102900
C	4.83345800	0.71446800	1.27198600
C	2.64188100	-0.31799400	1.35495900
C	5.19455600	0.03113300	2.42650700
H	5.54451700	1.37033200	0.77479100
C	2.99823100	-0.97502100	2.52920900
H	1.64693300	-0.44498800	0.94074900
C	4.27550600	-0.80844700	3.05855100
H	6.19235100	0.15302700	2.83590800
H	2.27379200	-1.61375600	3.02466300
H	4.55839000	-1.33456000	3.96528800

4pb

E(SMD/M06-2X/6-31g(d)) = -1017.223498 au

H(SMD/M06-2X/6-31g(d)) = -1016.931407 au

G(SMD/M06-2X/6-31g(d)) = -1016.993659 au

E(SMD/M06-2X/def2-TZVP//SMD/M06-2X/6-31g(d)) = -1017.484912 au

S	-0.13605800	-1.86562900	-0.96046000
C	-1.52924400	-0.79804100	-0.63428500
C	-1.43595300	0.57510900	-0.88131800
C	-2.71575700	-1.31987900	-0.11664500
C	-2.51796100	1.40757400	-0.61525100
H	-0.51313600	0.98560800	-1.28209500
C	-3.79201800	-0.47696600	0.15300500
H	-2.79810400	-2.38651400	0.07385700
C	-3.71081300	0.89591200	-0.09169400
H	-2.43610700	2.47370400	-0.81448600
H	-4.71155500	-0.89455400	0.55543500
C	-4.87032000	1.81277500	0.20007700
H	-4.61825300	2.52049300	0.99756300
H	-5.13789600	2.40206600	-0.68306700
H	-5.75271500	1.24944800	0.51466300
C	4.42142700	-0.06610700	-0.45495300
C	3.32013000	-0.67760400	0.13833500
C	2.21243400	0.07807500	0.54397400
C	2.23048800	1.46087800	0.31505200
C	3.33010200	2.07274500	-0.27949300
C	4.43188800	1.31116900	-0.66499600
H	5.27450900	-0.66884300	-0.75255800
H	3.33587800	-1.75147900	0.30089100
H	1.36361800	2.05545600	0.59042900
H	3.32115700	3.14506800	-0.45217200
H	5.28867700	1.78661700	-1.13301900
C	1.04013000	-0.55899700	1.20445100
C	0.59727100	-1.91407700	0.72959200
C	0.34820300	0.06990400	2.16138200
H	-0.53087400	-0.38496900	2.61045500

H	0.64565400	1.04562500	2.53577700
H	-0.14082200	-2.33980600	1.41379900
H	1.42685100	-2.62268300	0.64848900

4pb'

E(SMD/M06-2X/6-31g(d)) = -1017.227714 au

H(SMD/M06-2X/6-31g(d)) = -1016.935901 au

G(SMD/M06-2X/6-31g(d)) = -1016.997978 au

E(SMD/M06-2X/def2-TZVP//SMD/M06-2X/6-31g(d)) = -1017.489318 au

S	-0.80553700	1.87564700	-0.12147700
C	-2.18004600	0.74124300	-0.05463300
C	-2.13160900	-0.43222600	0.69997700
C	-3.34613900	1.06207500	-0.75450300
C	-3.23331100	-1.28338600	0.72996900
H	-1.23664800	-0.68200700	1.26363000
C	-4.44607900	0.21323500	-0.69748100
H	-3.38727600	1.96750300	-1.35365300
C	-4.40732300	-0.97630400	0.03773400
H	-3.18134600	-2.20085800	1.31108100
H	-5.34614300	0.47054000	-1.25092500
C	-5.60637000	-1.88656300	0.09568100
H	-5.32892700	-2.88270400	0.45097500
H	-6.36719600	-1.48963500	0.77753800
H	-6.07323100	-1.99044700	-0.88857500
C	5.21413500	-0.52109000	-0.32745900
C	4.19747200	0.41449000	-0.14864000
C	2.86834000	0.00773300	0.03223900
C	2.59421500	-1.36824000	0.05326100
C	3.60867900	-2.30211800	-0.12483000
C	4.92457000	-1.88315400	-0.31914500
H	6.23548500	-0.18158600	-0.47414600
H	4.43963200	1.47340300	-0.16418500
H	1.57925600	-1.71080400	0.23620800

H	3.37269900	-3.36206800	-0.09862500
H	5.71672700	-2.61350000	-0.45356900
C	1.79160600	1.01714400	0.21433500
C	0.54572600	0.74229100	-0.20587600
C	2.16651100	2.34084700	0.82664000
H	1.29587500	2.82423800	1.28016700
H	2.93216400	2.21483800	1.59770900
H	2.56728600	3.02963600	0.07286100
H	0.30798600	-0.19275700	-0.70647300

3ab

E(SMD/M06-2X/6-31g(d)) = -1208.897131 au

H(SMD/M06-2X/6-31g(d)) = -1208.548456 au

G(SMD/M06-2X/6-31g(d)) = -1208.618907 au

E(SMD/M06-2X/def2-TZVP//SMD/M06-2X/6-31g(d)) = -1209.225934 au

S	1.03867800	-1.40801000	-0.60802100
C	2.62906300	-0.67903800	-0.24893300
C	2.79096500	0.22667000	0.80405500
C	3.73658700	-1.05893700	-1.00666400
C	4.04716300	0.75470900	1.07672300
H	1.93498800	0.51378400	1.40944100
C	4.99380000	-0.53674300	-0.70974300
H	3.61728400	-1.75482300	-1.83233800
C	5.16985200	0.37946600	0.32909500
H	4.16190100	1.46522300	1.89208000
H	5.85049500	-0.83663800	-1.30804000
C	6.52979600	0.93594900	0.65970400
H	6.48778300	2.01901600	0.81213600
H	6.91818700	0.49289000	1.58399100
H	7.24772000	0.72850700	-0.13809300
C	-3.61645400	-2.26582000	1.53290100
C	-2.88122400	-1.13020900	1.21140100

C	-2.07083100	-1.10520500	0.06709900
C	-2.03920800	-2.23337800	-0.76014600
C	-2.78157400	-3.36947300	-0.44158900
C	-3.56873500	-3.38935200	0.70669000
H	-4.23019000	-2.27435800	2.42874000
H	-2.92293400	-0.25442300	1.85371800
H	-1.45070800	-2.21186300	-1.67404000
H	-2.75026300	-4.23470800	-1.09706600
H	-4.14811400	-4.27336000	0.95576200
C	-1.27039100	0.10605000	-0.25358600
C	0.03702900	0.04720700	-0.56883600
H	0.57406700	0.94908200	-0.85106600
C	-1.95680700	1.42840800	-0.22427200
C	-3.30545100	1.53900200	-0.59131800
C	-1.27644300	2.59080100	0.16640800
C	-3.94624800	2.77471700	-0.58750600
H	-3.85379000	0.65002400	-0.88991400
C	-1.91624000	3.82579500	0.16718300
H	-0.24254100	2.52326300	0.49410000
C	-3.25412200	3.92417100	-0.21216900
H	-4.98985000	2.83811300	-0.88190800
H	-1.37070400	4.71218700	0.47765900
H	-3.75534200	4.88749900	-0.20582000

1p

E(SMD/M06-2X/6-31g(d)) = -348.8088055 au

H(SMD/M06-2X/6-31g(d)) = -348.636275 au

G(SMD/M06-2X/6-31g(d)) = -348.67803 au

E(SMD/M06-2X/def2-TZVP//SMD/M06-2X/6-31g(d)) = -348.9396265 au

C	-1.96175700	1.14396300	-0.19893900
C	-0.57275800	1.20788600	-0.20059400
C	0.20392500	0.05878300	0.01002600

C	-0.46056000	-1.16115000	0.19442900
C	-1.85213900	-1.22651000	0.19779100
C	-2.60926100	-0.07437700	0.00407300
H	-2.54037600	2.04746300	-0.36931900
H	-0.08169500	2.15806100	-0.38991400
H	0.11049700	-2.07199400	0.34802200
H	-2.34395700	-2.18294000	0.35003000
H	-3.69405800	-0.12497000	0.00116800
C	1.69195600	0.12467600	0.02416100
C	2.34229500	1.24067600	0.37194300
H	1.82395400	2.13885100	0.69547600
H	3.42815400	1.27934800	0.36251800
C	2.44679000	-1.12681300	-0.34836400
H	3.51467300	-0.91704100	-0.44775800
H	2.32985800	-1.90431600	0.41552700
H	2.08199700	-1.54527100	-1.29291100

1a

E(SMD/M06-2X/6-31g(d)) = -540.479364 au

H(SMD/M06-2X/6-31g(d)) = -540.250379 au

G(SMD/M06-2X/6-31g(d)) = -540.300271 au

E(SMD/M06-2X/def2-TZVP//SMD/M06-2X/6-31g(d)) = -540.677368 au

C	2.58841000	-1.45995800	-0.89243800
C	1.38490500	-0.76563600	-0.81582900
C	1.27743100	0.39777600	-0.04155000
C	2.40059100	0.83210600	0.67303400
C	3.60362500	0.13449500	0.60107300
C	3.70242700	-1.01244300	-0.18391500
H	2.65479200	-2.35367300	-1.50603000
H	0.52050400	-1.12035800	-1.37084000
H	2.32270800	1.71443100	1.30236100
H	4.46235600	0.48201300	1.16812800

H	4.63958000	-1.55848400	-0.23792400
C	0.00001200	1.16445400	-0.00005800
C	-0.00002400	2.50471400	0.00004200
H	0.92405900	3.07123300	-0.07363300
H	-0.92417800	3.07111700	0.07374600
C	-1.27741800	0.39773600	0.04141600
C	-2.40068300	0.83211000	-0.67301700
C	-1.38482000	-0.76567100	0.81569400
C	-3.60370000	0.13450200	-0.60093600
H	-2.32287100	1.71442500	-1.30236600
C	-2.58832100	-1.46003600	0.89238400
H	-0.52042500	-1.12035900	1.37074800
C	-3.70240700	-1.01249500	0.18403300
H	-4.46250300	0.48203600	-1.16787800
H	-2.65462200	-2.35375300	1.50598100
H	-4.63955200	-1.55854900	0.23810900

TS1-Me

E(SMD/M06-2X/6-31g(d)) = -3585.27474 au

H(SMD/M06-2X/6-31g(d)) = -3584.694373 au

G(SMD/M06-2X/6-31g(d)) = -3584.838469 au

E(SMD/M06-2X/def2-TZVP//SMD/M06-2X/6-31g(d)) = -3586.645692 au

B	1.49008500	-0.31193600	0.01063100
C	2.64177900	0.77426000	-0.39027700
C	3.69999500	0.56191200	-1.26561300
C	4.60373600	1.55323200	-1.63107900
C	4.46043400	2.83067700	-1.11775800
C	3.41439500	3.09784200	-0.24691600
C	2.53821500	2.07850500	0.08792400
C	1.79869600	-1.85594900	-0.43362700
C	2.95402600	-2.45468900	0.06133000
C	3.27322800	-3.78880600	-0.12096000

C	2.39586800	-4.60146100	-0.82642100
C	1.22937100	-4.05768600	-1.33394500
C	0.95101600	-2.71082000	-1.12355700
C	1.14469500	-0.38854000	1.61312000
C	1.92887800	0.08222700	2.65878300
C	1.55997600	-0.01752300	3.99535600
C	0.35901400	-0.61964300	4.33225600
C	-0.44893200	-1.12422200	3.32551200
C	-0.03628900	-1.01724600	2.00570100
F	3.89045600	-0.63459800	-1.84569600
F	5.59577900	1.28559400	-2.48376100
F	5.31375700	3.79425400	-1.45868000
F	3.26262500	4.32644400	0.25336600
F	1.54851900	2.40178100	0.93767500
F	3.82433300	-1.70765400	0.76245100
F	4.39771300	-4.30386600	0.38086300
F	2.67796800	-5.89052800	-1.01122000
F	0.37398700	-4.82744800	-2.01139700
F	-0.20886300	-2.27006400	-1.64863200
F	-0.84325700	-1.58217000	1.09062600
F	-1.60688100	-1.72028300	3.62985600
F	-0.00918500	-0.72287600	5.60844600
F	2.35092800	0.46380300	4.95764600
F	3.10464000	0.68264300	2.42103300
C	-1.55239200	0.61437800	-3.50629900
C	-0.21476100	0.27768200	-4.16046000
C	0.77584000	0.25884200	-2.99415800
C	-0.14381700	0.32574300	-1.80067100
H	-0.31040100	-0.70460200	-4.63308000
H	1.41622100	1.14732400	-2.97860000

O	0.18123700	0.19836800	-0.57399700
O	-2.59601900	0.85643100	-4.06189800
N	-1.39573000	0.57265200	-2.11239700
H	1.42425800	-0.62002500	-2.95390100
H	0.01943600	1.00925300	-4.93538000
S	-2.83710500	0.46547400	-0.50181200
C	-2.50435500	2.20468000	-0.25059500
C	-1.53052100	2.60951500	0.66536000
C	-3.16484800	3.14584100	-1.03836700
C	-1.23729500	3.96126300	0.79784200
H	-0.99625600	1.86829200	1.25410200
C	-2.86371000	4.49758600	-0.89084100
H	-3.90103200	2.82104900	-1.76833400
C	-1.90090700	4.92437200	0.02718000
H	-0.47173400	4.27691500	1.50253500
H	-3.38234800	5.23077300	-1.50285800
C	-1.56630900	6.38370000	0.18609000
H	-1.74309500	6.71540300	1.21474500
H	-0.50983000	6.56797600	-0.03604600
H	-2.16941900	7.00504300	-0.48073000
C	-7.49073500	-1.81306500	-0.77306900
C	-6.82702000	-0.72586500	-0.21371100
C	-5.49558800	-0.84178400	0.20967200
C	-4.84207700	-2.07433600	0.04241400
C	-5.50393300	-3.15440300	-0.52765500
C	-6.83179400	-3.02908200	-0.93439000
H	-8.52439900	-1.70634300	-1.08744600
H	-7.35579500	0.21460200	-0.10029300
H	-3.80566500	-2.19862300	0.34402600
H	-4.98085300	-4.09693000	-0.65571700

H	-7.34772700	-3.87535400	-1.37771700
C	-4.80131000	0.32099600	0.81006800
C	-3.59141500	0.16186700	1.48030700
C	-5.49863900	1.64984000	0.84608700
H	-4.84359100	2.42574400	1.24614000
H	-5.85939600	1.95444100	-0.14055100
H	-6.36876000	1.56746600	1.50920400
H	-3.20368900	-0.81959300	1.73098100
H	-3.20208100	0.98804700	2.06769300

TS1-Ph

E(SMD/M06-2X/6-31g(d)) = -3776.941345 au

H(SMD/M06-2X/6-31g(d)) = -3776.304198 au

G(SMD/M06-2X/6-31g(d)) = -3776.454748 au

E(SMD/M06-2X/def2-TZVP//SMD/M06-2X/6-31g(d)) = -3778.379362 au

B	-2.02172000	0.12068600	0.06193500
C	-2.95460000	-1.09106600	-0.51275200
C	-4.11523400	-0.95272800	-1.26413600
C	-4.82471800	-2.02962500	-1.78431100
C	-4.36831900	-3.31765500	-1.56650000
C	-3.20573500	-3.51003300	-0.83497300
C	-2.53321600	-2.40721100	-0.33436400
C	-2.66358400	1.61883700	-0.07144300
C	-3.88288600	1.86086400	0.55504900
C	-4.49165300	3.10219500	0.61094200
C	-3.85589000	4.19061700	0.02865700
C	-2.63425600	4.00737200	-0.59455200
C	-2.06120200	2.74008300	-0.62473300
C	-1.57925400	-0.03961500	1.63334300
C	-2.13208700	-0.89284600	2.57944300
C	-1.65410900	-1.00348700	3.88043300
C	-0.57924200	-0.22917000	4.28440100

C	-0.00841700	0.65640800	3.38316300
C	-0.52858300	0.74917800	2.10091500
F	-4.60976200	0.25954700	-1.56371600
F	-5.93426300	-1.82855300	-2.49923900
F	-5.03322900	-4.36139500	-2.05803600
F	-2.75363600	-4.74744400	-0.61803800
F	-1.41693500	-2.66050900	0.36954900
F	-4.52744000	0.83549000	1.13787100
F	-5.66855000	3.26654700	1.21912200
F	-4.41966500	5.39715000	0.06719000
F	-2.01002900	5.04742700	-1.15319100
F	-0.86845200	2.65755500	-1.24528600
F	0.04268300	1.66400700	1.29988400
F	1.02754300	1.41600800	3.75493200
F	-0.10310700	-0.32973200	5.52483800
F	-2.22034200	-1.85234500	4.74164100
F	-3.16761200	-1.68713700	2.26976400
C	0.88933400	0.51630400	-3.66012800
C	-0.53248100	0.63617500	-4.20248600
C	-1.41860200	0.23559400	-3.02039400
C	-0.42329500	0.17383000	-1.88877400
H	-0.69157800	1.67687100	-4.50077500
H	-1.85614500	-0.76086200	-3.14500300
O	-0.67880900	0.01899700	-0.64959000
O	1.91925100	0.63219500	-4.27901000
N	0.82762700	0.27236800	-2.27997800
H	-2.23386300	0.92986900	-2.79881200
H	-0.65239100	0.00545000	-5.08495300
S	2.31270400	0.53362100	-0.72861800
C	2.36573500	-1.24462700	-0.59415700

C	1.61093000	-1.91367900	0.36583400
C	3.16459800	-1.96248700	-1.48860000
C	1.68235600	-3.30304700	0.44842900
H	0.96601900	-1.35711700	1.04132300
C	3.22652200	-3.34601900	-1.39525900
H	3.73706000	-1.43517500	-2.24693000
C	2.49524700	-4.03599800	-0.41902900
H	1.09015900	-3.82329500	1.19678800
H	3.85780600	-3.90285000	-2.08360200
C	2.60377400	-5.53328100	-0.30769600
H	1.87051800	-5.93453900	0.39636800
H	2.44915100	-6.01320700	-1.27924700
H	3.60195400	-5.82201700	0.04127100
C	6.89070200	-1.77424200	-0.49177200
C	6.18292800	-0.57926600	-0.41180700
C	5.12267200	-0.43396300	0.49600300
C	4.82799600	-1.50641600	1.35564000
C	5.53938000	-2.69615900	1.27772400
C	6.56529600	-2.84010600	0.34350500
H	7.69891100	-1.87003400	-1.21008400
H	6.45728000	0.24131700	-1.06495500
H	4.04318100	-1.41697700	2.09846600
H	5.29093000	-3.51385800	1.94764800
H	7.11678600	-3.77329200	0.27882200
C	4.32154200	0.80890900	0.53461100
C	3.10832500	0.89317300	1.22008100
H	2.68621600	1.87118500	1.43028900
H	2.73412800	0.08068400	1.83431100
C	4.88978000	2.08412800	-0.00400100
C	5.20451400	3.09485900	0.91120800

C	5.10573500	2.30715300	-1.36921300
C	5.74449000	4.30089900	0.47190800
H	5.03038100	2.92864800	1.97106400
C	5.63830000	3.51660700	-1.80535100
H	4.82926500	1.54382900	-2.09116500
C	5.96296300	4.51346700	-0.88700400
H	5.98702800	5.07561900	1.19290700
H	5.79067000	3.68252700	-2.86743900
H	6.37556000	5.45665400	-1.23212700

TS2-Me

E(SMD/M06-2X/6-31g(d)) = -3585.279052 au

H(SMD/M06-2X/6-31g(d)) = -3584.702452 au

G(SMD/M06-2X/6-31g(d)) = -3584.844401 au

E(SMD/M06-2X/def2-TZVP//SMD/M06-2X/6-31g(d)) = -3586.647539au

B	2.02399500	-0.07401500	0.00310100
C	3.18532400	-1.21820600	-0.11801600
C	3.45810900	-2.02894000	0.98110100
C	4.37571200	-3.06711700	0.96934800
C	5.07067200	-3.34180500	-0.19925400
C	4.82856500	-2.57467600	-1.32540300
C	3.90037300	-1.54102500	-1.26522200
C	2.15127100	0.90145900	1.31467700
C	3.27877300	1.10382300	2.09923600
C	3.30330300	1.96224100	3.19251200
C	2.16366900	2.67206400	3.53399700
C	1.01829500	2.51893900	2.76871900
C	1.04298300	1.66277100	1.67860400
C	1.94019700	0.95513000	-1.26863300
C	3.07220700	1.70938700	-1.56678000
C	3.10925100	2.70282000	-2.52931200
C	1.95433200	2.99333300	-3.24382100
C	0.79890500	2.27973200	-2.98011500

C	0.81290500	1.28679100	-2.00559000
F	2.82074600	-1.81113500	2.14276600
F	4.59632600	-3.79991800	2.06331900
F	5.95518100	-4.33672300	-0.23753900
F	5.47705100	-2.83878900	-2.46267500
F	3.70420700	-0.87617100	-2.41616600
F	4.42471000	0.45687500	1.83863400
F	4.41573500	2.10856100	3.91621300
F	2.17039400	3.49543500	4.58198600
F	-0.09363500	3.19513500	3.07775400
F	-0.09031500	1.60025900	0.95184900
F	-0.35316200	0.64073100	-1.81705900
F	-0.32078600	2.54823200	-3.65667100
F	1.96198400	3.94770300	-4.17343700
F	4.23135800	3.38377800	-2.77347100
F	4.21064400	1.47028800	-0.89261300
C	-1.67532800	-2.71291800	-1.26423000
C	-0.68261800	-2.74690300	-2.42355700
C	0.59055200	-2.14793500	-1.82617300
C	0.07438500	-1.57304900	-0.52871700
H	-0.57176100	-3.76411400	-2.80217000
H	1.08667500	-1.39517700	-2.44260000
O	0.71128600	-0.79751500	0.26384400
O	-2.77625800	-3.21845600	-1.24702600
N	-1.13805800	-1.96452500	-0.21311000
H	1.32979700	-2.91828300	-1.57930200
H	-1.08677500	-2.12260700	-3.22682200
S	-2.98893600	1.23394400	-0.16548000
C	-4.67368500	0.92750900	-0.65772600
C	-5.00914600	-0.30013500	-1.23752200
C	-5.65984300	1.89873800	-0.47277600
C	-6.32410600	-0.55168400	-1.61046300
H	-4.24749000	-1.06513200	-1.36718600

C	-6.97547500	1.63068400	-0.84096400
H	-5.39995200	2.85910200	-0.03445600
C	-7.32794500	0.40343400	-1.40954300
H	-6.58064700	-1.51364200	-2.04877500
H	-7.74010000	2.38786800	-0.68757500
C	-8.75233600	0.09848800	-1.79167800
H	-8.83402100	-0.12113300	-2.86145000
H	-9.11806700	-0.78192800	-1.25142100
H	-9.41377800	0.93811500	-1.56357500
C	-3.17640600	-0.27836000	2.01528500
C	-3.14567700	1.16921100	1.68002600
H	-2.25296000	1.65902400	2.07042100
H	-4.03485400	1.72367900	1.97849000
C	-4.42853500	-1.00940800	1.89046000
C	-4.40105700	-2.36194900	1.49588100
C	-5.67195000	-0.37454900	2.07707700
C	-5.58580600	-3.04936500	1.27397300
H	-3.45426500	-2.85357600	1.29744400
C	-6.85191200	-1.07957800	1.88937700
H	-5.71874100	0.66129600	2.39520300
C	-6.81009800	-2.41225300	1.47656400
H	-5.55428500	-4.07980000	0.93520200
H	-7.80579500	-0.58644700	2.04809800
H	-7.73626100	-2.95369700	1.30644000
C	-1.95551000	-0.94940600	2.24644900
H	-1.68143400	-1.37173000	1.11528500
H	-1.10786200	-0.31647300	2.50674500
H	-2.01630500	-1.88102200	2.80867900

TS2'-Me

E(SMD/M06-2X/6-31g(d)) = -3585.270768 au

H(SMD/M06-2X/6-31g(d)) = -3584.693181 au

G(SMD/M06-2X/6-31g(d)) = -3584.832271 au

E(SMD/M06-2X/def2-TZVP//SMD/M06-2X/6-31g(d)) = -3586.636735 au

B	-1.16159600	0.52164100	0.21772300
C	-1.53023300	2.13035100	0.29908800
C	-1.36111200	2.92445000	1.43351400
C	-1.67104200	4.27884200	1.48896700
C	-2.18668300	4.91330600	0.37389200
C	-2.38672900	4.17411200	-0.77930400
C	-2.06525000	2.82423100	-0.78847200
C	-1.06984100	-0.22353900	1.69297500
C	-1.97645900	-1.14576200	2.20516500
C	-1.76312600	-1.88730700	3.36452100
C	-0.60343700	-1.70682800	4.09418400
C	0.32588400	-0.77945400	3.64521600
C	0.07623200	-0.07060100	2.48008400
C	-2.19790800	-0.27779500	-0.77627500
C	-3.57231700	-0.07123800	-0.67533900
C	-4.51637200	-0.72504000	-1.44690200
C	-4.09726300	-1.65533900	-2.38844900
C	-2.74485100	-1.89979500	-2.53203900
C	-1.83259800	-1.21517200	-1.73355100
F	-0.88974700	2.42147400	2.58398900
F	-1.47742500	4.96816300	2.61552700
F	-2.48617800	6.20959800	0.40934600
F	-2.89030200	4.75998700	-1.86812400
F	-2.30922600	2.19819800	-1.95421900
F	-3.13577800	-1.42012000	1.58447500
F	-2.66990000	-2.78516800	3.75890100
F	-0.38499400	-2.39985300	5.20960100
F	1.45682100	-0.58541800	4.32766000
F	1.04599500	0.79049800	2.12956800
F	-0.54195400	-1.52723400	-1.95585000

F	-2.31946500	-2.80077300	-3.42361300
F	-4.98697700	-2.30301900	-3.13954600
F	-5.81992200	-0.48204300	-1.28848000
F	-4.04677400	0.78527300	0.24382000
C	2.62593700	0.38504100	-2.72480000
C	1.67309000	1.35059600	-3.42664200
C	0.46463200	1.42037200	-2.49004700
C	0.91462500	0.56126000	-1.33458800
H	2.17332200	2.31555900	-3.54663000
H	-0.44939200	1.00877400	-2.92563300
O	0.26698400	0.31618300	-0.25843300
O	3.67223300	-0.03818800	-3.16599900
N	2.10206100	0.02613600	-1.48180900
H	0.24053400	2.43019200	-2.13543000
H	1.43458500	0.96873800	-4.42176100
S	4.22012700	-1.20626300	1.59989300
C	4.89005600	0.09550000	0.56990800
C	4.22796600	1.32124200	0.45079100
C	6.11277100	-0.09585100	-0.07054900
C	4.78027800	2.33074600	-0.32730200
H	3.27873800	1.47230200	0.95815100
C	6.66771900	0.93306100	-0.83037700
H	6.63208000	-1.04560000	0.02324100
C	6.00837400	2.15390500	-0.97889000
H	4.25687300	3.27936000	-0.42451700
H	7.62327000	0.77666000	-1.32434400
C	6.57960500	3.25183800	-1.83699900
H	6.03296400	3.32405200	-2.78449600
H	6.50092500	4.22384900	-1.34038200
H	7.63124300	3.06708800	-2.07083300

C	-0.15240700	-4.54922700	-1.92741400
C	1.09653800	-4.03030000	-1.62853500
C	1.32204800	-3.36929300	-0.40064500
C	0.25737800	-3.27205200	0.52584300
C	-0.98332100	-3.81166300	0.22824800
C	-1.19077200	-4.44275400	-1.00058200
H	-0.32347100	-5.03231800	-2.88358600
H	1.88987000	-4.10142900	-2.36434000
H	0.41127600	-2.81576100	1.49809000
H	-1.79165600	-3.74266500	0.95062500
H	-2.16701800	-4.85814400	-1.23477700
C	2.60405000	-2.75820100	-0.13593600
C	2.68053300	-1.63421700	0.74630700
C	3.78844700	-3.06513000	-0.96987100
H	3.84042900	-2.27198200	-1.74111900
H	4.70525700	-2.98590200	-0.38050200
H	3.73776400	-4.03634800	-1.45814900
H	1.81538000	-1.47209000	1.38261700
H	2.52059600	-0.85908800	-0.16368200

TS2-Ph

E(SMD/M06-2X/6-31g(d)) = -3776.947496 au

H(SMD/M06-2X/6-31g(d)) = -3776.313496 au

G(SMD/M06-2X/6-31g(d)) = -3776.462067 au

E(SMD/M06-2X/def2-TZVP//SMD/M06-2X/6-31g(d)) = -3778.382186 au

B	-1.98435800	-0.07410800	0.00753100
C	-3.09146800	-1.07705400	-0.66658800
C	-4.30836400	-1.25026000	-0.01323400
C	-5.27766000	-2.15571200	-0.40953300
C	-5.03416900	-2.96550700	-1.51040300
C	-3.83402400	-2.84344100	-2.18776900
C	-2.89113200	-1.91795500	-1.75261800

C	-2.50245700	1.45771300	0.26517700
C	-1.68384800	2.33189800	0.97519100
C	-1.96562400	3.67271900	1.17841400
C	-3.13194800	4.20341700	0.64757000
C	-3.98312200	3.37964700	-0.06901200
C	-3.65541400	2.03987500	-0.24752700
C	-1.49408900	-0.85974300	1.36108100
C	-1.76980500	-0.50538800	2.67622200
C	-1.36225300	-1.25736200	3.77203900
C	-0.66410900	-2.43576100	3.57190500
C	-0.39004400	-2.84852700	2.27757400
C	-0.80665000	-2.06265500	1.21273900
F	-4.57993700	-0.50157700	1.06971900
F	-6.43023000	-2.26669300	0.25512000
F	-5.94789600	-3.84964800	-1.90895100
F	-3.58628800	-3.61686200	-3.24775500
F	-1.75417200	-1.86665900	-2.47243800
F	-0.54586100	1.87594100	1.52458600
F	-1.12936300	4.45132900	1.87086000
F	-3.42746100	5.49061700	0.81940200
F	-5.10019200	3.88192500	-0.60090400
F	-4.51674400	1.33062800	-0.99544900
F	-0.53744500	-2.53173800	-0.01808800
F	0.26086500	-3.99880500	2.07445600
F	-0.26778100	-3.17142600	4.61015100
F	-1.64029500	-0.85306200	5.01440300
F	-2.45533800	0.61188300	2.96411400
C	0.32789400	0.97912100	-3.96078100
C	-1.15500400	1.21443300	-4.21902400
C	-1.77020000	1.10675900	-2.82452800

C	-0.64011400	0.48512700	-2.03067000
H	-1.31329100	2.17458700	-4.71217700
H	-2.69357600	0.52839200	-2.76786800
O	-0.71588000	0.03428900	-0.83288300
O	1.24332000	1.17468700	-4.72873700
N	0.50545400	0.46768300	-2.67087400
H	-1.96931200	2.09490700	-2.39485700
H	-1.51423400	0.42123700	-4.88280700
S	4.02618700	-0.35419100	-2.84323800
C	5.39369100	-0.68828700	-1.74250300
C	6.24891900	0.33683300	-1.32488700
C	5.59347100	-1.98514800	-1.27519800
C	7.27545500	0.06142000	-0.43094300
H	6.10556800	1.34874500	-1.69620100
C	6.64138500	-2.25347700	-0.39356800
H	4.92048700	-2.78131000	-1.58094200
C	7.48933100	-1.23809600	0.04892000
H	7.93175400	0.86523900	-0.10506300
H	6.78577100	-3.26788400	-0.03038700
C	8.60005700	-1.51347300	1.02800300
H	8.41142500	-1.01121200	1.98355700
H	8.70087700	-2.58424600	1.22334700
H	9.55843600	-1.14236100	0.65054800
C	3.46560700	4.58991200	0.35166300
C	3.61546400	3.38504700	-0.31521900
C	3.05108000	2.20609900	0.21878800
C	2.33414800	2.27400900	1.43215500
C	2.15686300	3.49159900	2.07244900
C	2.72649300	4.64628500	1.53651000
H	3.91987800	5.48980400	-0.04951800

H	4.20523800	3.34342700	-1.22518600
H	1.87187400	1.37777500	1.83044300
H	1.56756700	3.54123300	2.98195900
H	2.59476800	5.59780700	2.04292800
C	3.13956900	0.94099100	-0.48300500
C	3.13946100	0.93110900	-1.91172400
H	1.96868200	0.66988500	-2.12424300
H	3.24800000	1.91627200	-2.36640300
C	3.12577100	-0.30145700	0.27848900
C	3.88873700	-0.39826100	1.45548600
C	2.39355500	-1.41338600	-0.17486600
C	3.93862400	-1.59889200	2.15394400
H	4.47351100	0.45391700	1.78855600
C	2.42116300	-2.59498100	0.55087200
H	1.77399500	-1.33586500	-1.06388500
C	3.19869600	-2.69230600	1.70770100
H	4.54891600	-1.67678300	3.04796100
H	1.82808100	-3.44047400	0.22108000
H	3.22032800	-3.62506200	2.26358800

4-Fluorobenzenethiosuccinimide (2a)

E(SMD/M06-2X/6-31g(d)) = -463.28796 au

H(SMD/M06-2X/6-31g(d)) = -463.079654 au

G(SMD/M06-2X/6-31g(d)) = -463.127138 au

E(SMD/M06-2X/def2-TZVP//SMD/M06-2X/6-31g(d)) = -463.466871 au

C	2.18745800	-1.16901300	-0.17864700
C	3.17777600	-0.79338100	-1.26289700
C	3.11930000	0.73305300	-1.36180500
C	2.15287300	1.17500200	-0.28091600
H	4.16187200	-1.16355600	-0.96276100
H	2.73775500	1.08608600	-2.32317300
O	1.81381000	2.29812000	-0.00598800

O	1.86477900	-2.27416300	0.17786900
N	1.68347200	0.02172000	0.36962200
H	4.08239200	1.21838100	-1.18649300
H	2.89607100	-1.30332100	-2.18678600
S	0.57985700	0.06224600	1.67887900
C	-0.96810500	0.02329600	0.79294500
C	-1.53121800	-1.20437600	0.43149300
C	-1.62061300	1.22046800	0.48568800
C	-2.74648500	-1.23918300	-0.24329700
H	-1.01337900	-2.12732100	0.67341600
C	-2.83908200	1.19574000	-0.18485900
H	-1.17273300	2.16823400	0.76791100
C	-3.37361900	-0.03578400	-0.53358700
H	-3.21044700	-2.17391700	-0.53949900
H	-3.37302900	2.10562200	-0.43688600
F	-4.54988300	-0.06497400	-1.17943300

1o

E(SMD/M06-2X/6-31g(d)) = -1088.838153 au

H(SMD/M06-2X/6-31g(d)) = -1088.656804 au

G(SMD/M06-2X/6-31g(d)) = -1088.71247 au

E(SMD/M06-2X/def2-TZVP//SMD/M06-2X/6-31g(d)) = -1089.153744 au

C	-1.15055300	0.98375700	-0.13274100
C	0.23353000	1.13499700	-0.14753700
C	1.10178600	0.05023300	0.01642600
C	0.52179400	-1.22004800	0.17107600
C	-0.85371700	-1.39001200	0.19057400
C	-1.70272200	-0.28823700	0.04215300
H	-1.78152600	1.85450600	-0.27065200
H	0.64150800	2.12791100	-0.31337100
H	1.15389800	-2.09525300	0.28776900
H	-1.29563400	-2.37366200	0.31700800

C	2.57973800	0.22319200	0.01233300
C	3.16233200	1.38678500	0.32534400
H	2.59554600	2.25885900	0.63861600
H	4.24302700	1.49443900	0.29367000
C	3.41599900	-0.98035100	-0.34578200
H	4.46823800	-0.70268600	-0.44519900
H	3.34752700	-1.75600100	0.42605400
H	3.08168000	-1.43192900	-1.28643000
O	-3.03243000	-0.55241100	0.06976600
C	-3.92254700	0.53730300	-0.09701800
H	-3.78699700	1.01633000	-1.07357700
H	-4.92733800	0.11762700	-0.03666500
H	-3.79432900	1.28343400	0.69568800

3oa

E(SMD/M06-2X/6-31g(d)) = -1191.614051 au

H(SMD/M06-2X/6-31g(d)) = -1191.323865 au

G(SMD/M06-2X/6-31g(d)) = -1191.390273 au

E(SMD/M06-2X/def2-TZVP//SMD/M06-2X/6-31g(d)) = -1191.958014 au

S	-1.87093900	1.89941600	-0.34059600
C	-3.05403600	0.57673800	-0.15973600
C	-2.79362400	-0.53429000	0.64725700
C	-4.28321200	0.68632900	-0.81597600
C	-3.74593000	-1.53952600	0.78583800
H	-1.84634500	-0.61556700	1.17276700
C	-5.25007300	-0.30303600	-0.66573800
H	-4.48771400	1.54303500	-1.45157300
C	-4.95803100	-1.40150900	0.12778300
H	-3.56135400	-2.41377000	1.40139300
H	-6.21076300	-0.23745200	-1.16536600
C	4.45100000	0.43144300	0.01149400
C	3.27901600	1.17870500	0.12899200

C	2.01591600	0.58128800	0.11022300
C	1.96345600	-0.81920900	-0.00732100
C	3.11565500	-1.57580900	-0.12337300
C	4.37231500	-0.95520400	-0.12069500
H	5.40809300	0.94008000	0.02280000
H	3.36498600	2.25746900	0.22356600
H	1.00396000	-1.32871000	0.01746100
H	3.07009500	-2.65750400	-0.20439600
C	0.78573700	1.40711700	0.22534600
C	-0.36095800	0.98139400	-0.33151300
C	0.90129300	2.72432500	0.94605900
H	-0.07774400	3.07866500	1.27911700
H	1.55570100	2.63382700	1.81817700
H	1.32698200	3.49695700	0.29452800
H	-0.40517400	0.05446800	-0.89745100
O	5.44291800	-1.77815000	-0.24091900
C	6.72869200	-1.18228800	-0.22968800
H	7.44188300	-2.00006500	-0.33764500
H	6.85053500	-0.48216600	-1.06423700
H	6.91633000	-0.66029600	0.71573500
F	-5.88456600	-2.36813800	0.26425500

3oa'

E(SMD/M06-2X/6-31g(d)) = -1191.605055 au

H(SMD/M06-2X/6-31g(d)) = -1191.313902 au

G(SMD/M06-2X/6-31g(d)) = -1191.378944 au

E(SMD/M06-2X/def2-TZVP//SMD/M06-2X/6-31g(d)) = -1191.949132 au

S	1.80474100	1.78347300	0.38006600
C	3.10250400	0.56603400	0.22056800
C	3.94591100	0.58685100	-0.89345800
C	3.29415400	-0.39835600	1.21551400
C	4.97382800	-0.34362000	-1.01892500

H	3.79906400	1.33503200	-1.66604000
C	4.31067900	-1.34073900	1.09815500
H	2.64763900	-0.40990000	2.08806400
C	5.13179700	-1.29018700	-0.01875200
H	5.64197100	-0.34447600	-1.87341200
H	4.47655200	-2.09846700	1.85649100
C	-4.50977100	0.37199700	0.35774200
C	-3.34993500	1.14341100	0.33947200
C	-2.14607000	0.65467300	-0.17680300
C	-2.13546000	-0.66310700	-0.66222800
C	-3.28022800	-1.44405100	-0.65371500
C	-4.47877500	-0.93152900	-0.14393500
H	-5.41868900	0.78885900	0.77604900
H	-3.38466600	2.14380800	0.76156000
H	-1.22082100	-1.08591600	-1.06822200
H	-3.26930100	-2.45970800	-1.03684300
C	-0.91656700	1.49388900	-0.19882500
C	0.38707200	0.73051300	-0.10825500
C	-0.97785400	2.82441900	-0.31595100
H	-0.08935500	3.44820300	-0.32148200
H	-1.92869300	3.33550000	-0.43480400
H	0.62653200	0.27348100	-1.07523900
H	0.29330200	-0.07805000	0.62341400
O	-5.54424400	-1.76907000	-0.16900400
C	-6.76825800	-1.28556100	0.35693300
H	-7.48801500	-2.09664800	0.24279100
H	-6.67171200	-1.03264500	1.41916800
H	-7.12010800	-0.40759700	-0.19667100
F	6.12069200	-2.19359000	-0.13434200

$TS_{2\text{-}Me\text{-}OMe\text{-}F}$

E(SMD/M06-2X/6-31g(d)) = -3759.673755 au

H(SMD/M06-2X/6-31g(d)) = -3759.09823 au

G(SMD/M06-2X/6-31g(d)) = -3759.24214 au

E(SMD/M06-2X/def2-TZVP//SMD/M06-2X/6-31g(d)) = -3761.125639 au

B	1.71903800	-0.50245400	0.11223500
C	1.88138300	-1.30592600	1.52826000
C	1.17815800	-0.83988900	2.63628000
C	1.18423700	-1.45687800	3.87613400
C	1.91963200	-2.62087300	4.04600500
C	2.62772900	-3.13531700	2.97416500
C	2.59203900	-2.47932700	1.74812400
C	2.09592200	1.08889900	0.20111500
C	2.72943900	1.74863600	1.24621900
C	2.96976000	3.11870100	1.25014900
C	2.60471400	3.87827900	0.15303500
C	2.00448100	3.25985500	-0.93276500
C	1.76111500	1.89664900	-0.88460000
C	2.60159100	-1.09248100	-1.13316600
C	3.98628900	-1.12127100	-0.99614100
C	4.85675200	-1.48605700	-2.00858000
C	4.33947200	-1.82799400	-3.25038500
C	2.96983500	-1.80170900	-3.44431700
C	2.13473400	-1.42982400	-2.39591000
F	0.44557500	0.28378700	2.53275700
F	0.49292400	-0.95082500	4.89991900
F	1.93854300	-3.23701300	5.22634800
F	3.32477300	-4.26480400	3.11930500
F	3.27125700	-3.07807600	0.75682200
F	3.13210700	1.08774500	2.34414100
F	3.53433600	3.70750100	2.30837500

F	2.77646900	5.20018100	0.15925500
F	1.63916300	3.98710200	-1.99223500
F	1.15619000	1.36148900	-1.95701900
F	0.81730800	-1.42515900	-2.67656200
F	2.46020100	-2.12451800	-4.63592900
F	5.15506200	-2.18209600	-4.24314800
F	6.17703100	-1.50616600	-1.80979800
F	4.53039000	-0.78548500	0.18572600
C	-2.35343900	-2.60859700	-0.96473400
C	-1.29094300	-3.69394700	-0.85340300
C	-0.09658100	-2.95445000	-0.25476700
C	-0.51933700	-1.50777600	-0.39676400
H	-1.65647000	-4.52677200	-0.25054000
H	0.86349600	-3.15298000	-0.73591800
O	0.22631700	-0.48693000	-0.21114900
O	-3.51898300	-2.76477200	-1.26290800
N	-1.78614500	-1.35801800	-0.71354400
H	0.01793000	-3.16481600	0.81437400
H	-1.07928600	-4.06652100	-1.86063500
S	-2.30233000	1.92086500	-1.99006000
C	-1.51843500	2.86044300	-0.69342700
C	-1.24852800	4.20748200	-0.95302600
C	-1.13251000	2.29253400	0.52469700
C	-0.56978800	4.98255000	-0.01741200
H	-1.55889700	4.65298700	-1.89360300
C	-0.47810500	3.06750000	1.47709700
H	-1.30230300	1.23917700	0.72473900
C	-0.19419900	4.39055200	1.17752300
H	-0.32020300	6.02052400	-0.21098500
H	-0.14841400	2.64738400	2.42112100

C	-4.41813400	0.13862900	-1.66067400
C	-3.42134000	0.90300000	-1.00511500
H	-2.73895100	-0.11382900	-0.76144600
H	-3.67616100	1.35489200	-0.04738300
C	-5.61519800	-0.24898500	-0.96497700
C	-6.31569000	-1.43647200	-1.29357200
C	-6.14300800	0.57012300	0.05623200
C	-7.46008800	-1.78810300	-0.61801500
H	-5.91975400	-2.11067000	-2.04345400
C	-7.30622800	0.23901900	0.72522300
H	-5.66313100	1.51278500	0.29680700
C	-7.96844700	-0.95497300	0.39770100
H	-7.98796500	-2.70858200	-0.84336500
H	-7.69874000	0.90742100	1.48165800
C	-4.12455200	-0.31689500	-3.04516700
H	-4.78608000	-1.10457800	-3.39978500
H	-4.24308500	0.55306800	-3.70912400
H	-3.07880000	-0.63756800	-3.12757400
O	-9.08759900	-1.37914900	0.98989400
C	-9.63289400	-0.59923000	2.05081100
H	-10.50634200	-1.14832600	2.40059800
H	-8.91127700	-0.49438100	2.86676600
H	-9.93982800	0.38777700	1.69083100
F	0.52005600	5.11179600	2.06245300

$TS_{2'\text{-}Me\text{-}OMe\text{-}F}$

E(SMD/M06-2X/6-31g(d)) = -3759.670804 au

H(SMD/M06-2X/6-31g(d)) = -3759.095523 au

G(SMD/M06-2X/6-31g(d)) = -3759.242559 au

E(SMD/M06-2X/def2-TZVP//SMD/M06-2X/6-31g(d)) = -3761.122362 au

B	-2.28757200	0.12051600	-0.00093900
C	-3.31146200	1.36807200	-0.25918100

C	-3.44432100	2.34869900	0.72162700
C	-4.22830800	3.48258600	0.58139000
C	-4.92470000	3.68083100	-0.60166400
C	-4.82132200	2.74090800	-1.61254100
C	-4.02432200	1.61706500	-1.42587200
C	-2.49761300	-0.64855800	1.43149300
C	-3.62142400	-0.60454600	2.24664600
C	-3.71439500	-1.28555700	3.45488800
C	-2.65378600	-2.06452400	3.88722400
C	-1.51877500	-2.15862600	3.09711400
C	-1.47437200	-1.47313200	1.89301000
C	-2.34121200	-1.05890600	-1.13432200
C	-3.55300000	-1.71919400	-1.32118000
C	-3.72140000	-2.80509600	-2.16219400
C	-2.62541200	-3.29205700	-2.86173100
C	-1.39618900	-2.67720100	-2.70612400
C	-1.27666800	-1.58728200	-1.84953600
F	-2.79601300	2.21363800	1.89003500
F	-4.31794800	4.38149600	1.56460800
F	-5.67812900	4.76653200	-0.76449700
F	-5.47481400	2.92682600	-2.76210900
F	-3.95221600	0.78177400	-2.47556000
F	-4.69310600	0.12666100	1.90471200
F	-4.81557800	-1.19213300	4.20446600
F	-2.72442100	-2.71524300	5.04786900
F	-0.48544600	-2.90872400	3.49444800
F	-0.35995900	-1.64604300	1.15696500
F	-0.04487100	-1.05076500	-1.76465900
F	-0.33365100	-3.13379200	-3.37355000
F	-2.75924900	-4.33689900	-3.67783000

F	-4.91296300	-3.39055100	-2.30251400
F	-4.63894300	-1.28281900	-0.65992400
C	1.62947800	2.22403500	-1.60188300
C	0.61071700	2.25842700	-2.73768200
C	-0.69558500	1.83055700	-2.06814900
C	-0.20530700	1.33118700	-0.73102800
H	0.58193100	3.24943900	-3.19337200
H	-1.26809800	1.07075500	-2.60523100
O	-0.89214400	0.71221900	0.14869500
O	2.77405300	2.61800700	-1.64815600
N	1.05206400	1.62023700	-0.48109500
H	-1.36206600	2.68044300	-1.88256500
H	0.93473100	1.53993400	-3.49719300
S	2.55093400	-1.77022400	0.02890600
C	4.21745700	-1.58149900	-0.57393800
C	4.56582400	-0.42991400	-1.28790600
C	5.17397300	-2.57612300	-0.34556400
C	5.86339700	-0.26222200	-1.76057600
H	3.82898200	0.35203400	-1.45104100
C	6.47692500	-2.41665500	-0.80561300
H	4.90078400	-3.47492900	0.19984700
C	6.79271600	-1.25903800	-1.50274700
H	6.16021100	0.62606200	-2.30873800
H	7.23915800	-3.17030200	-0.63967300
C	2.93441400	0.09686700	1.94419700
C	2.77180300	-1.38189400	1.82613600
H	1.85291400	-1.72488600	2.30438800
H	3.61672100	-1.95365800	2.21045600
C	4.23457400	0.67845200	1.70815500
C	4.33362200	1.97899000	1.17260000

C	5.43083700	-0.05354300	1.90146300
C	5.55502700	2.52567500	0.81990700
H	3.43530300	2.54857900	0.95778300
C	6.65362200	0.49360600	1.58979300
H	5.40057100	-1.05324500	2.32156500
C	6.72675700	1.78339400	1.03030700
H	5.58897600	3.51022200	0.36913500
H	7.57588200	-0.05583400	1.74836100
C	1.77513800	0.88726200	2.07106600
H	1.53299000	1.17668000	0.85893300
H	0.87479500	0.36733400	2.39568700
H	1.90041200	1.88533500	2.48988600
O	7.95591400	2.21414900	0.72382300
C	8.09380900	3.50725900	0.14259400
H	9.16128500	3.64416100	-0.02713200
H	7.55899200	3.56432400	-0.81096900
H	7.72698700	4.28157000	0.82388500
F	8.05078900	-1.09879300	-1.94580700

5. NMR Spectra

Figure S1: ^{1}**H NMR** (400 MHz, $CDCl_3$, 298 K) spectrum of **1b**.

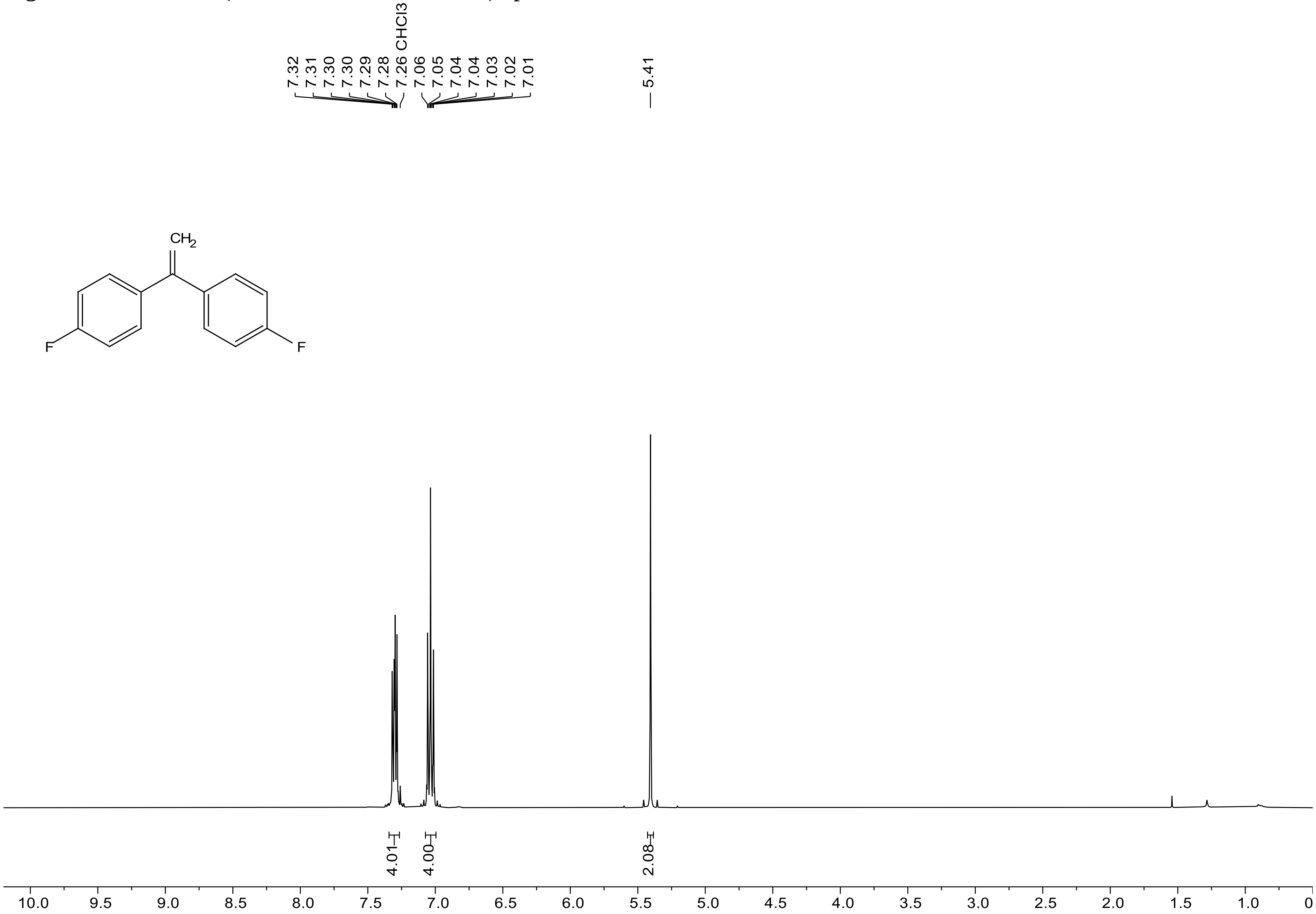

Figure S2: **^{13}C NMR** (101 MHz, $CDCl_3$, 298 K) spectrum of **1b**.

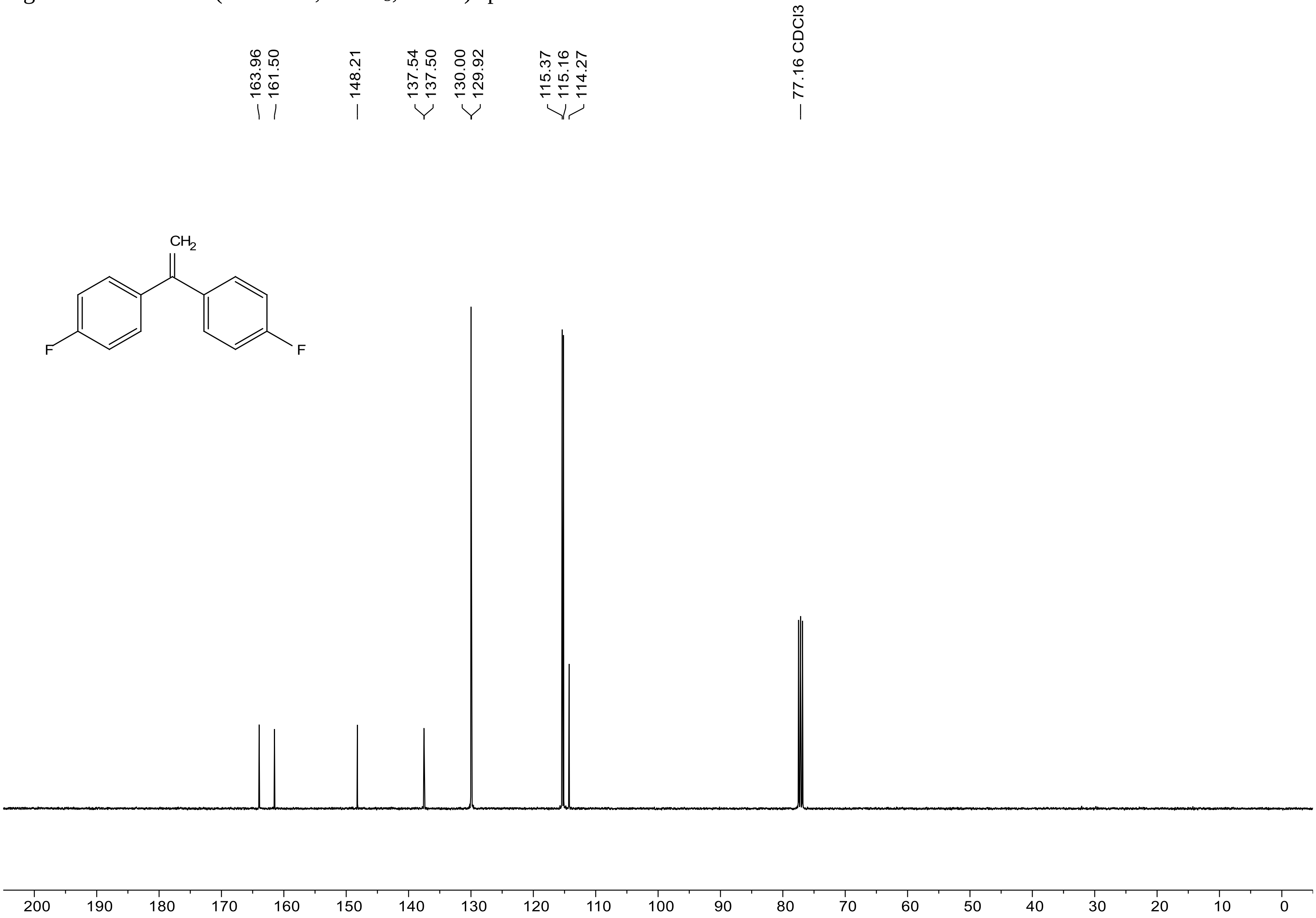

Figure S3: ^{19}**F NMR** (376 MHz, $CDCl_3$, 298 K) spectrum of **1b**.

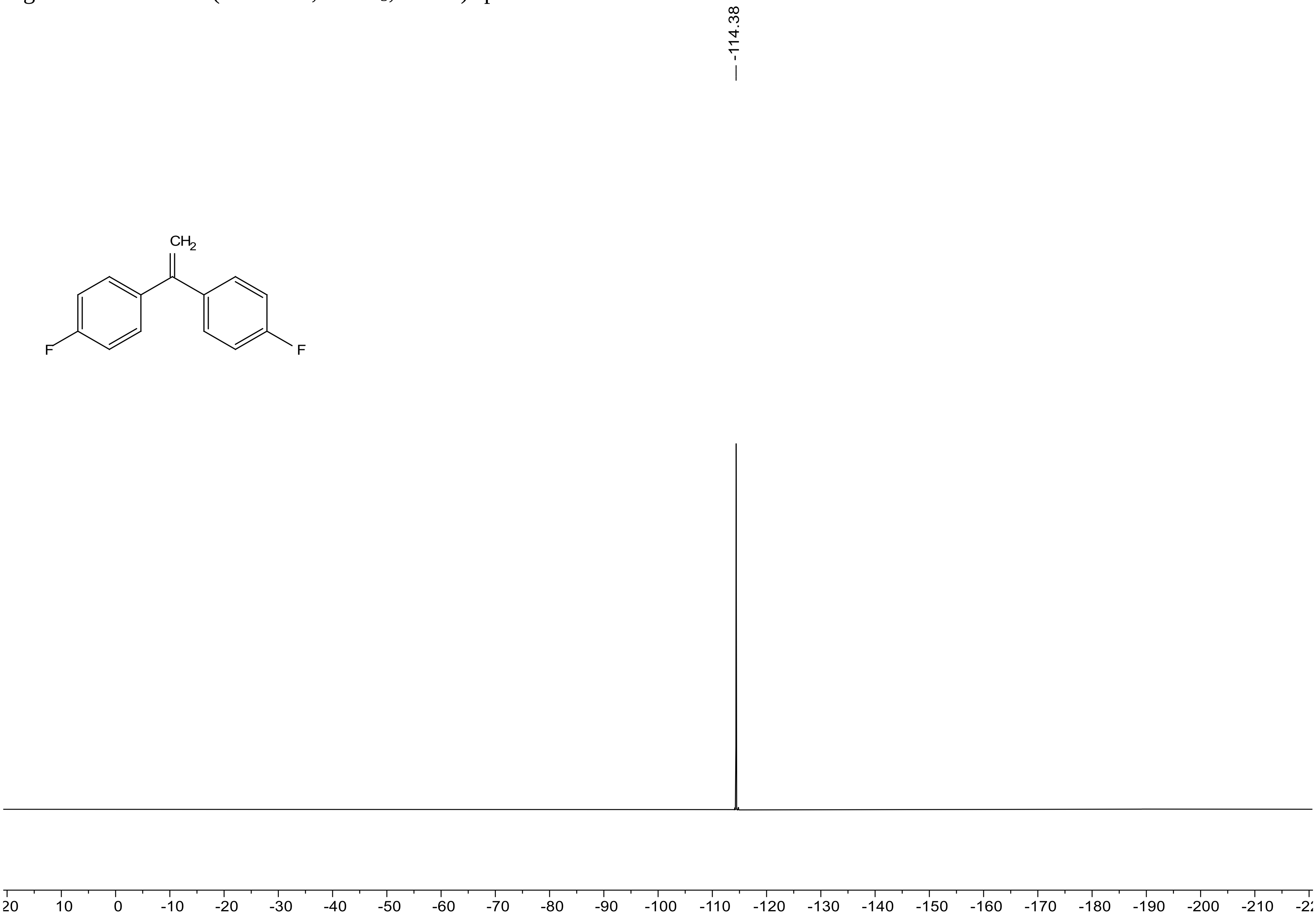

Figure S4: **^{1}H NMR** (400 MHz, $CDCl_3$, 298 K) spectrum of **1c**.

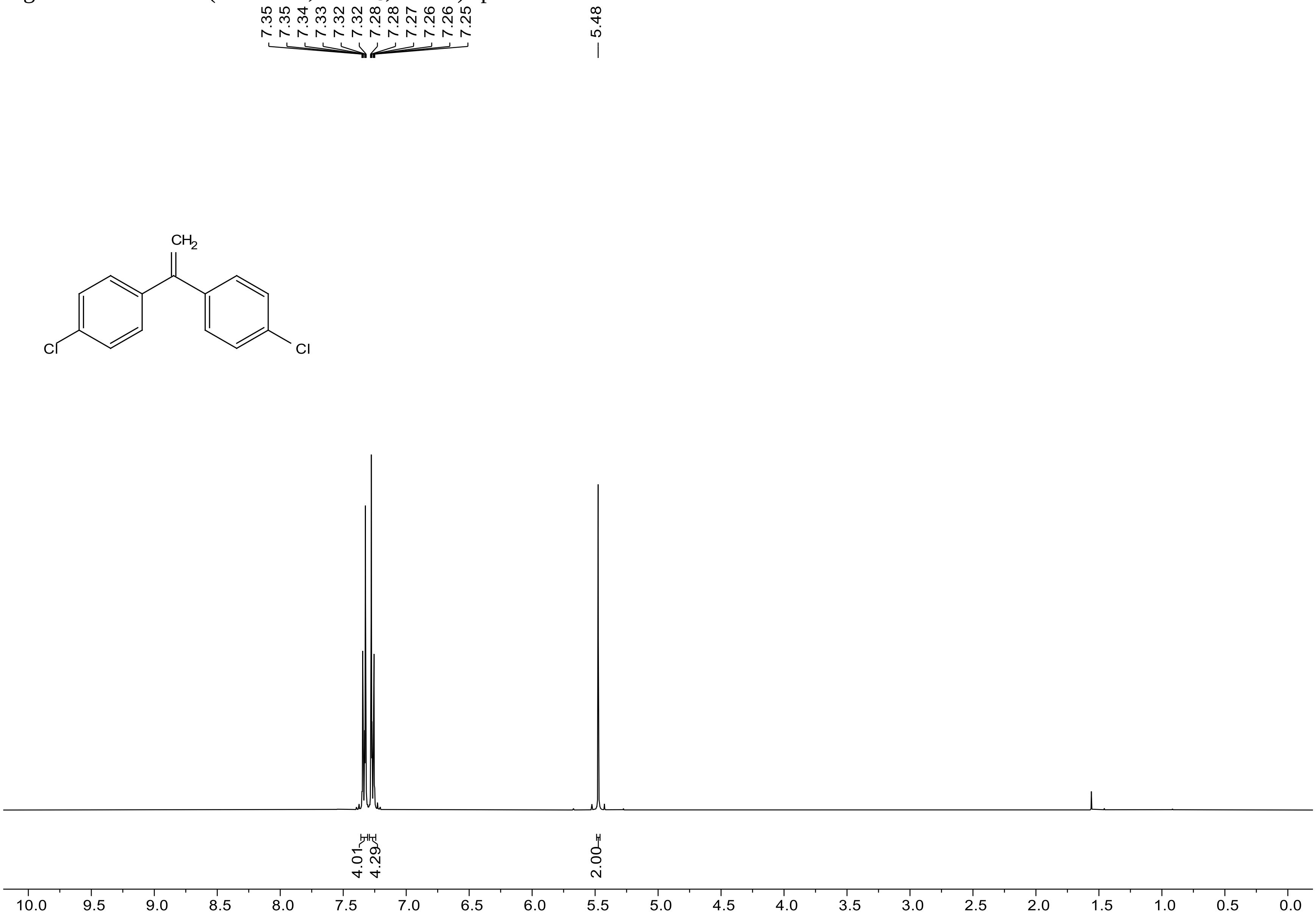

Figure S5: ^{13}**C NMR** (101 MHz, $CDCl_3$, 298 K) spectrum of **1c**.

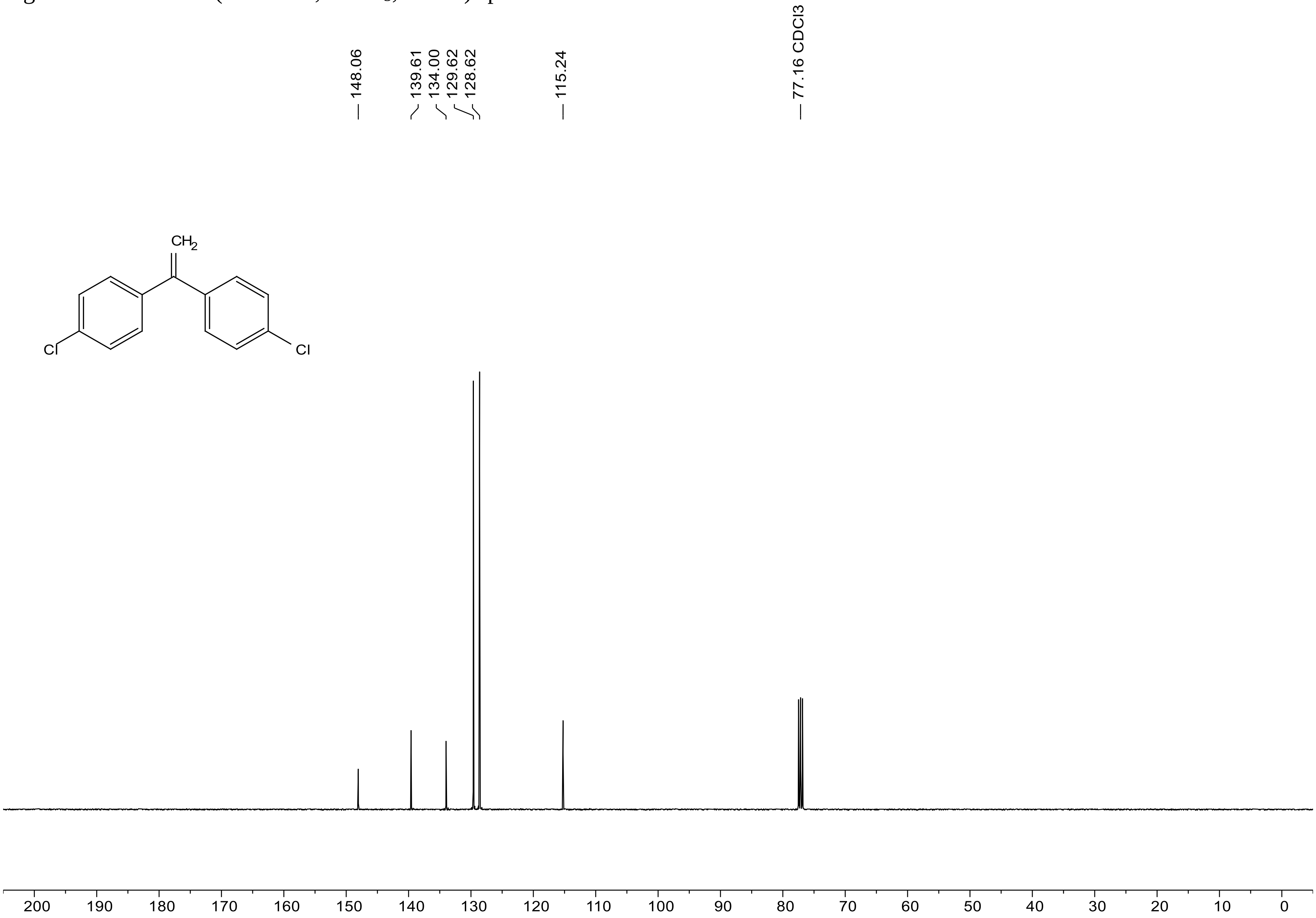

Figure S6: **^{1}H NMR** (400 MHz, $CDCl_3$, 298 K) spectrum of **1d**.

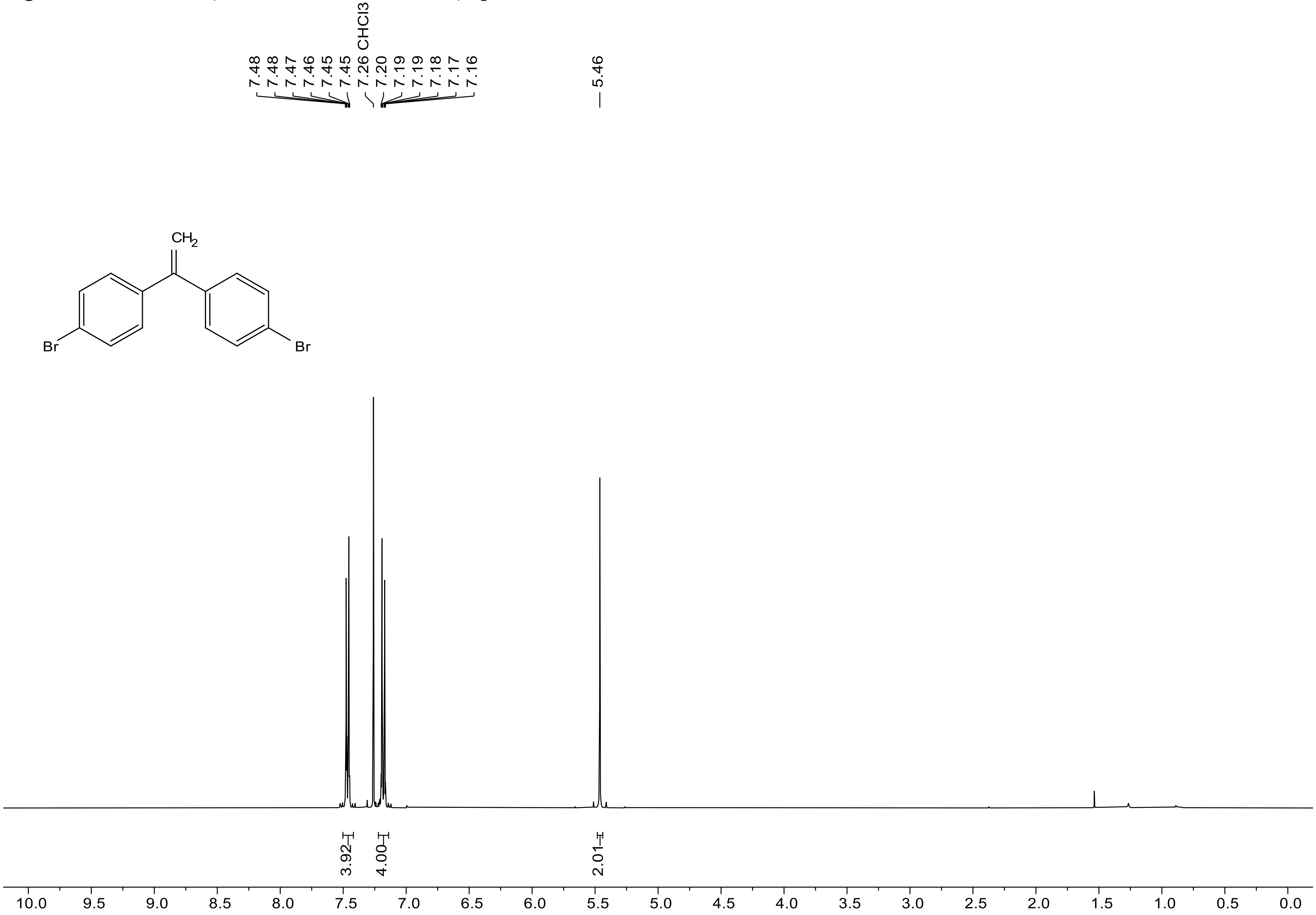

Figure S7: ^{13}**C NMR** (101 MHz, $CDCl_3$, 298 K) spectrum of **1d**.

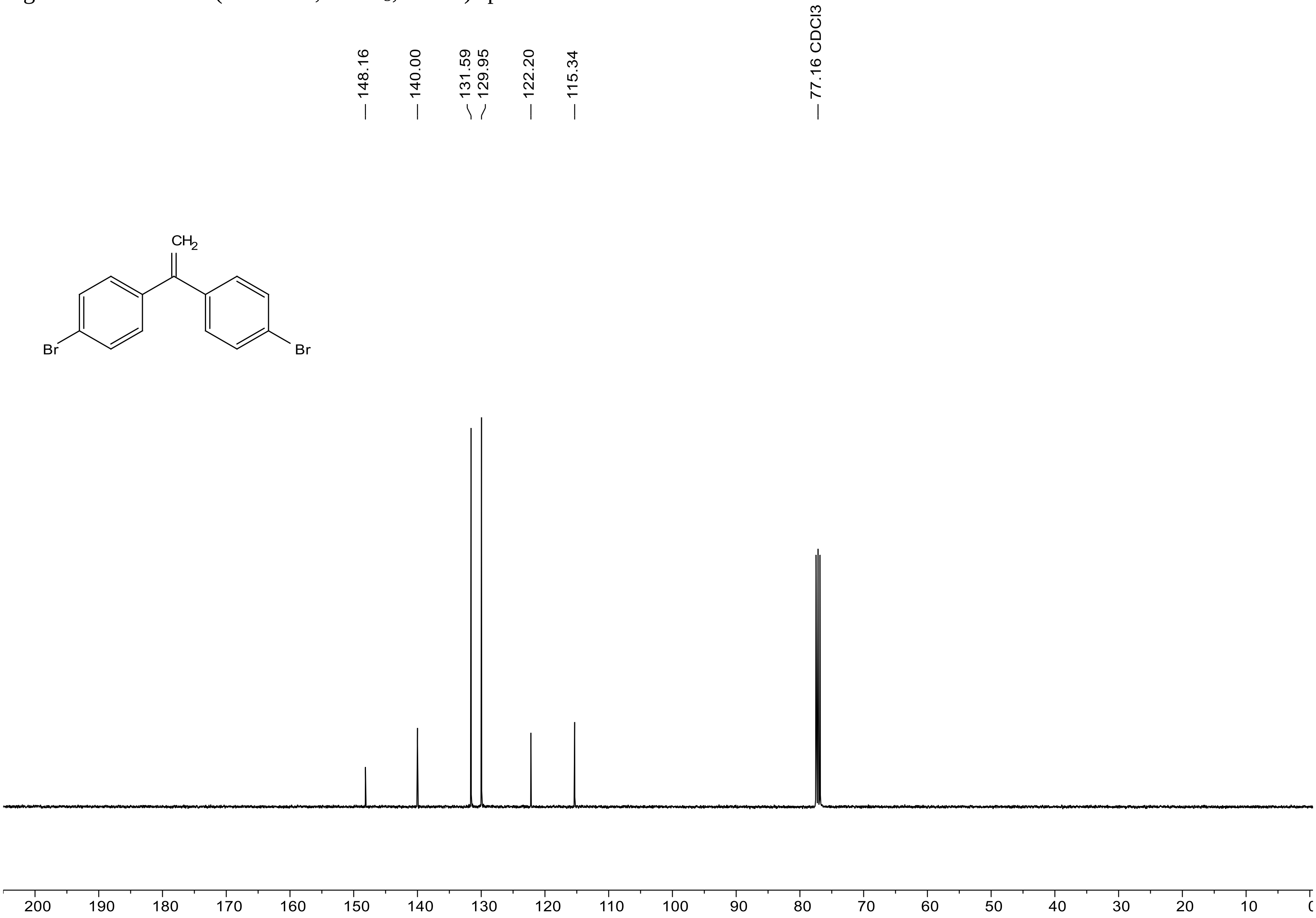

Figure S8: **^{1}H NMR** (400 MHz, $CDCl_3$, 298 K) spectrum of **1e**.

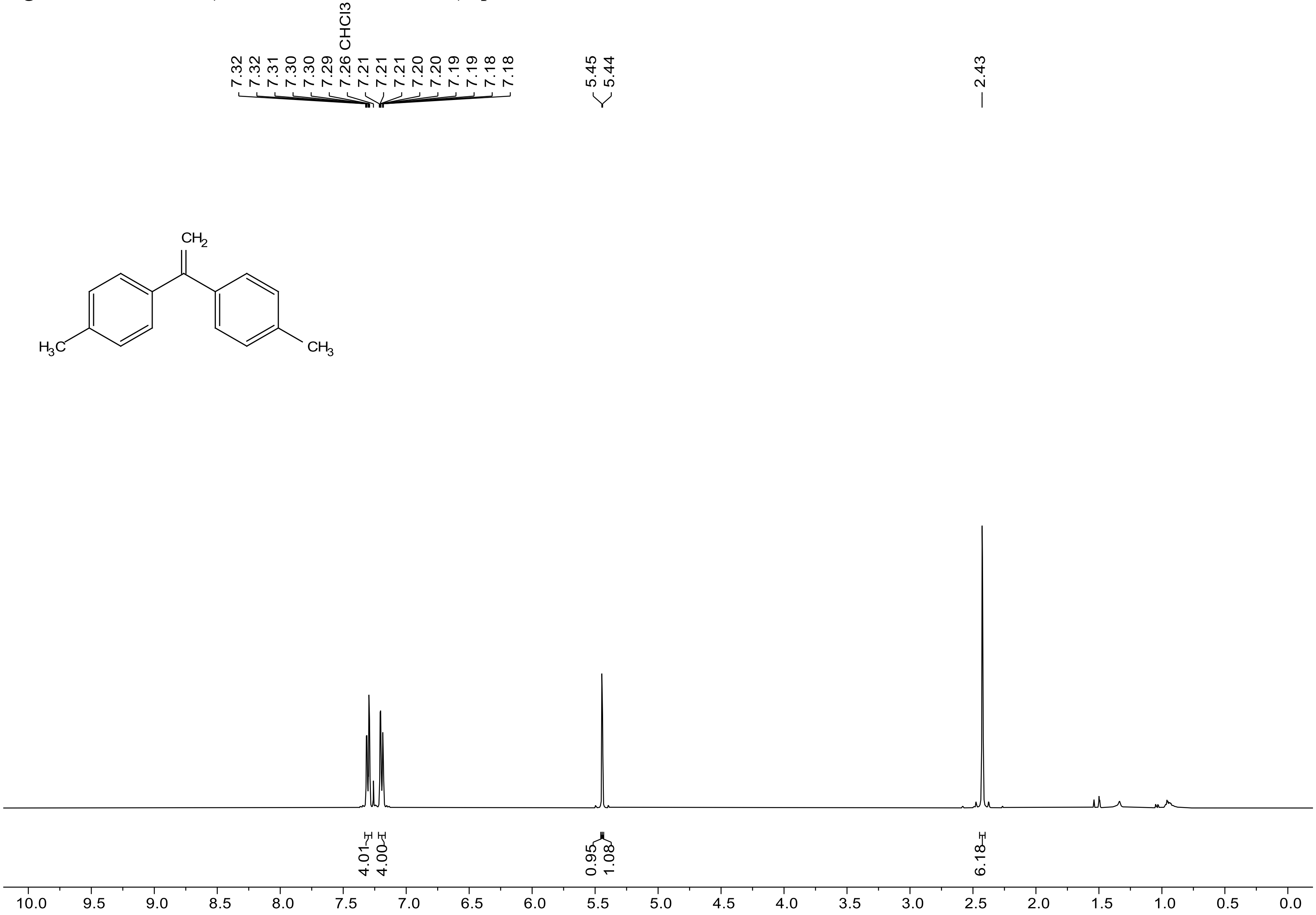

Figure S9: ^{13}C **NMR** (101 MHz, $CDCl_3$, 298 K) spectrum of **1e**.

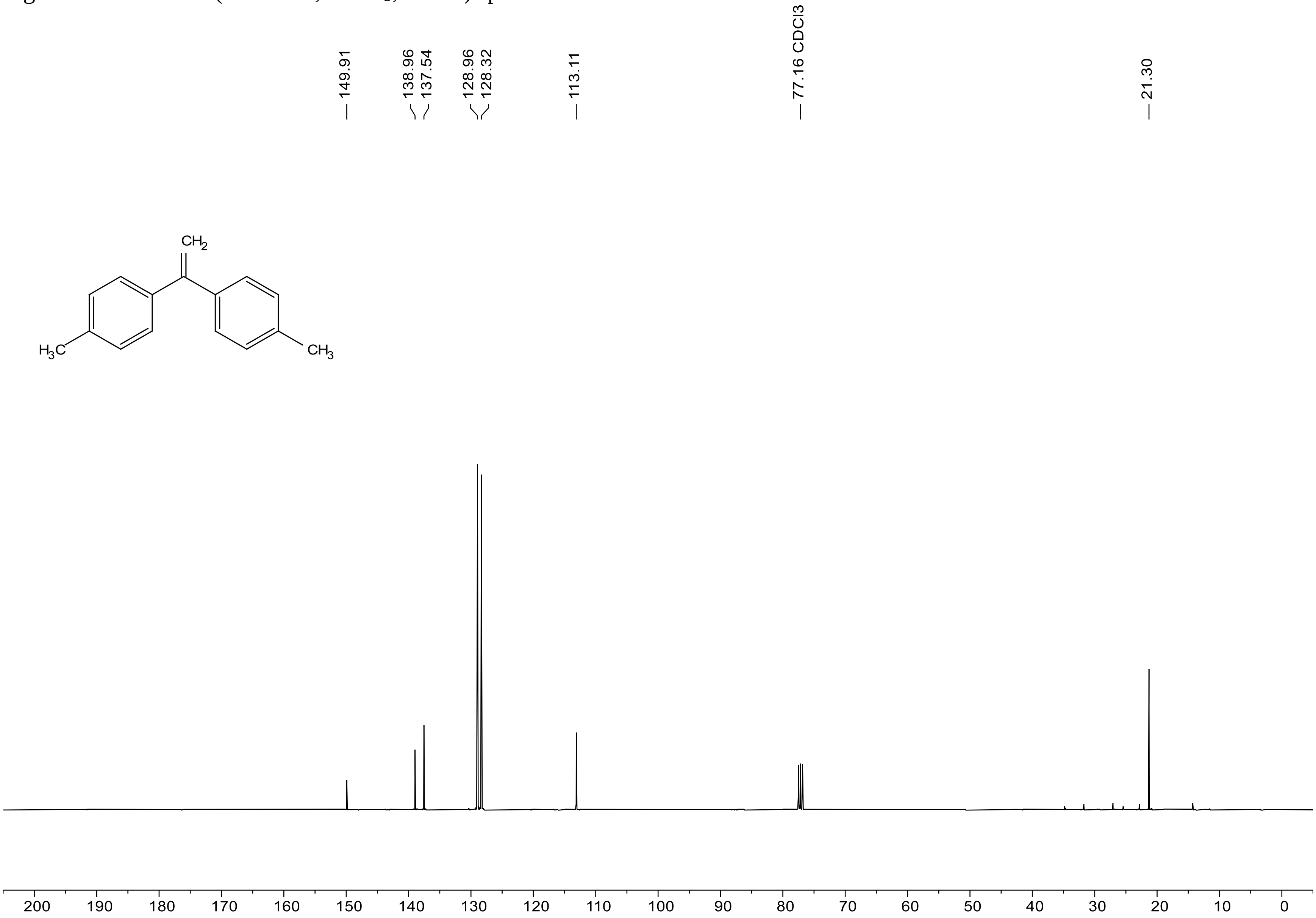

Figure S10: **^{1}H NMR** (400 MHz, $CDCl_3$, 298 K) spectrum of **1f**.

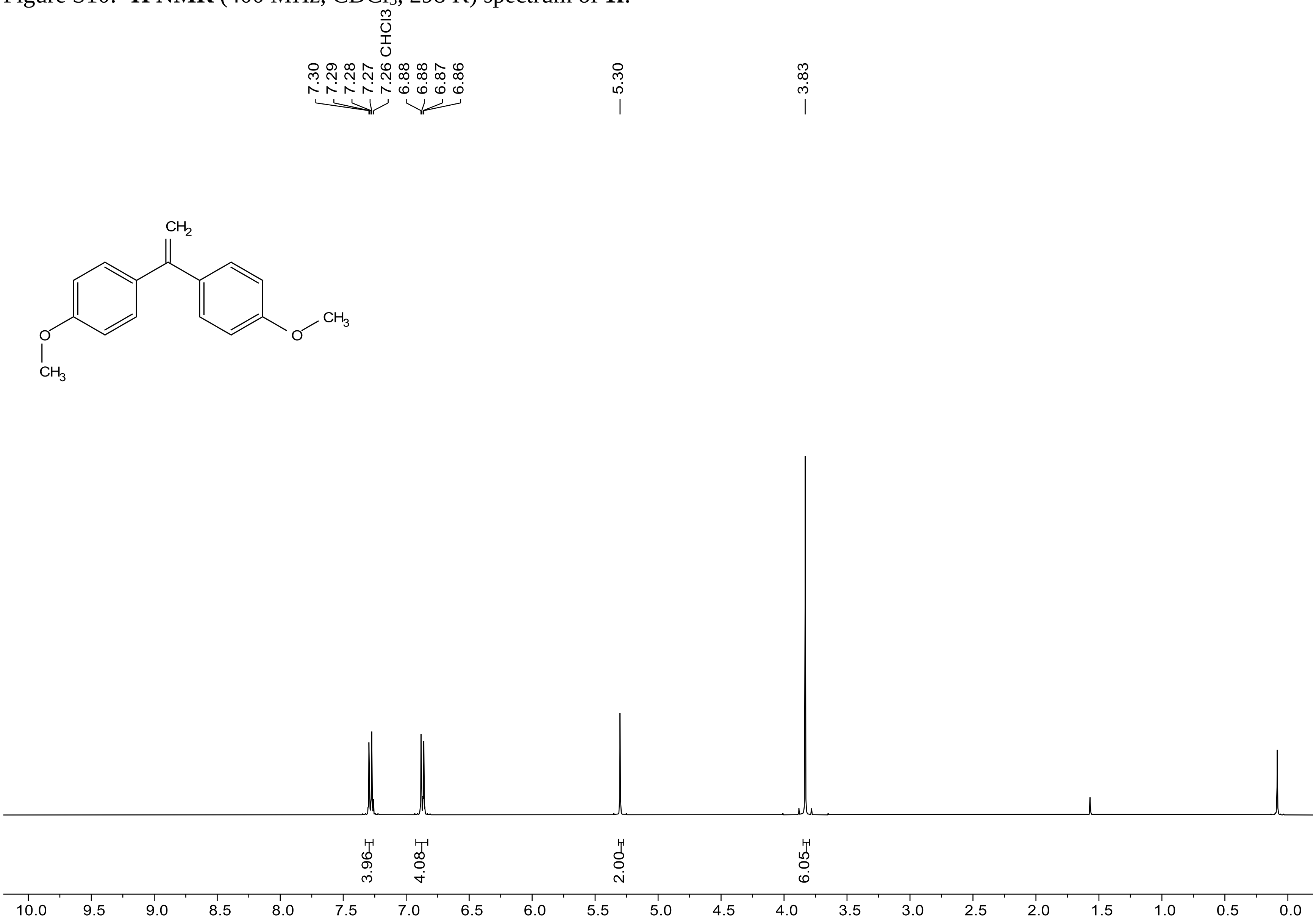

Figure S11: **^{13}C NMR** (101 MHz, $CDCl_3$, 298 K) spectrum of **1f**.

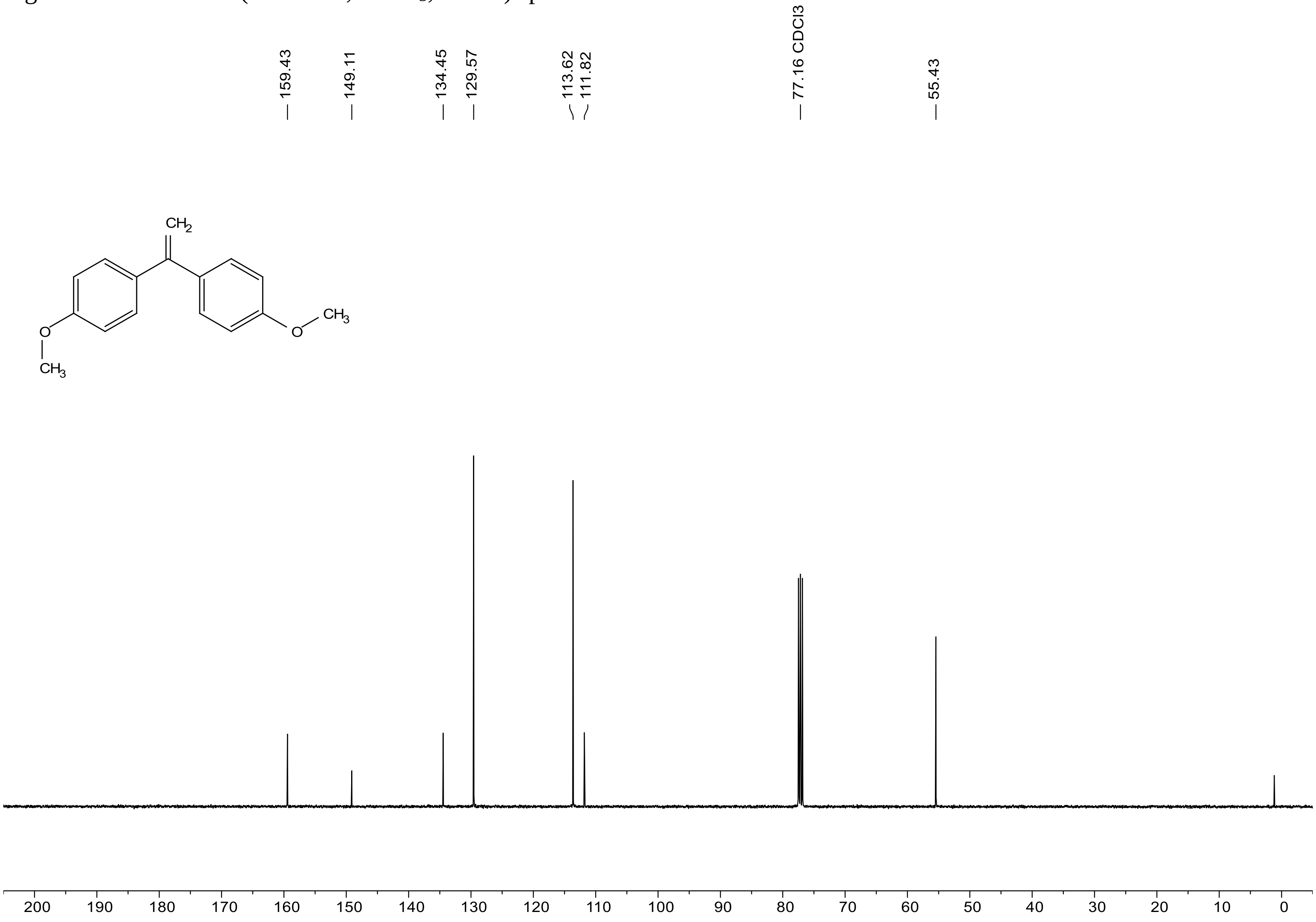

Figure S12: **^{1}H NMR** (400 MHz, $CDCl_3$, 298 K) spectrum of **1g**.

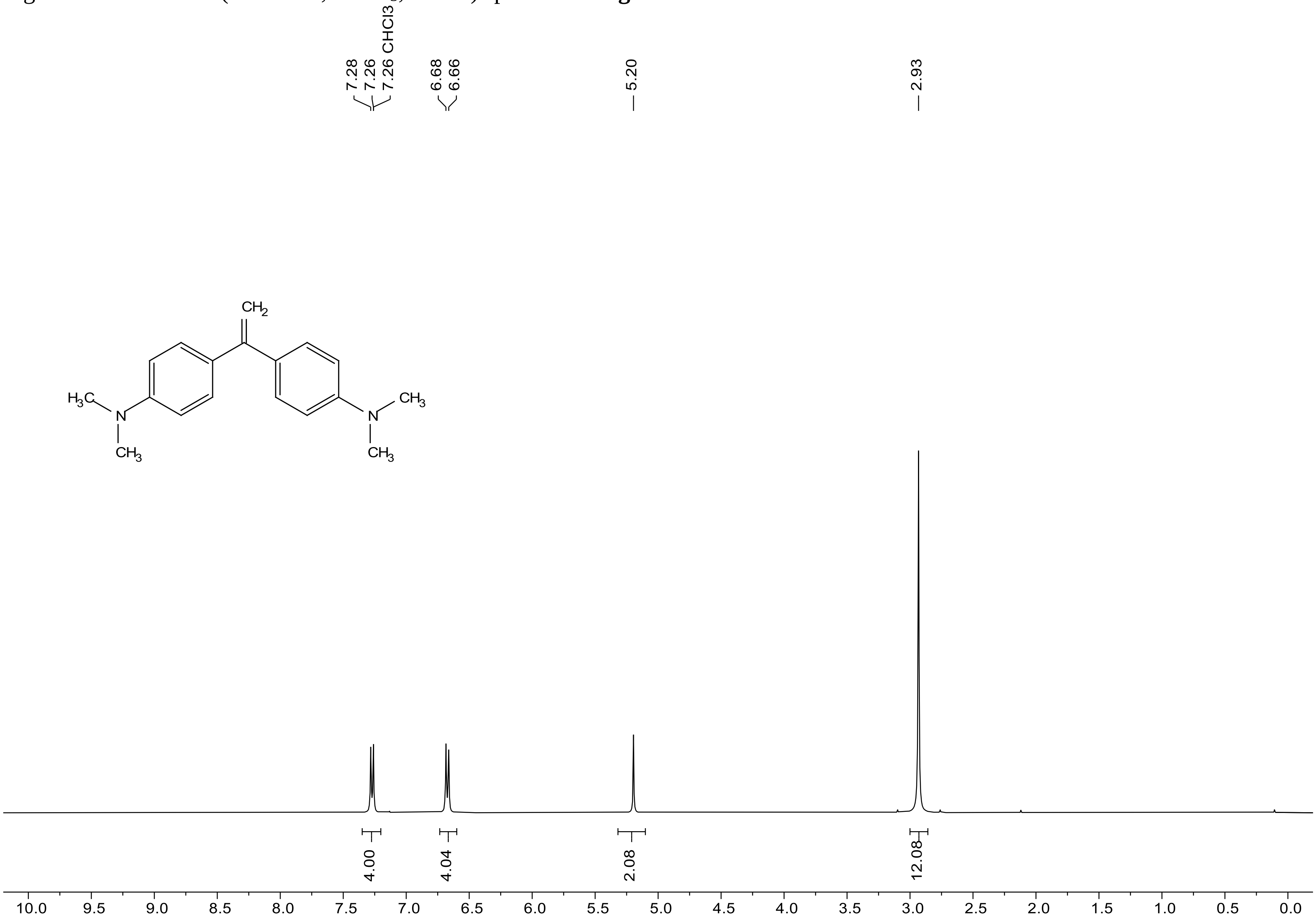

Figure S13: **^{13}C NMR** (101 MHz, $CDCl_3$, 298 K) spectrum of **1g**.

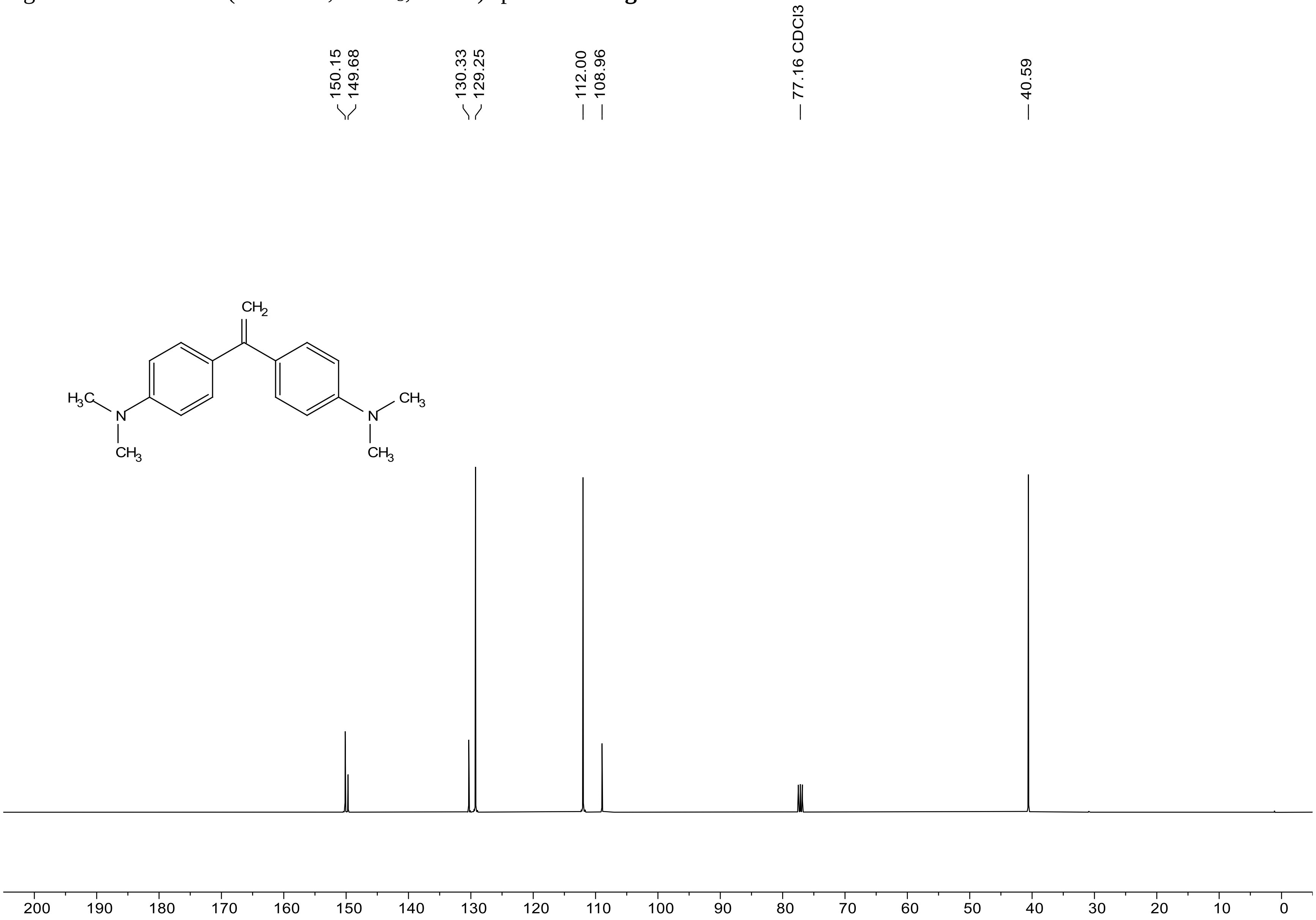

Figure S14: **^{1}H NMR** (400 MHz, $CDCl_3$, 298 K) spectrum of **1h**.

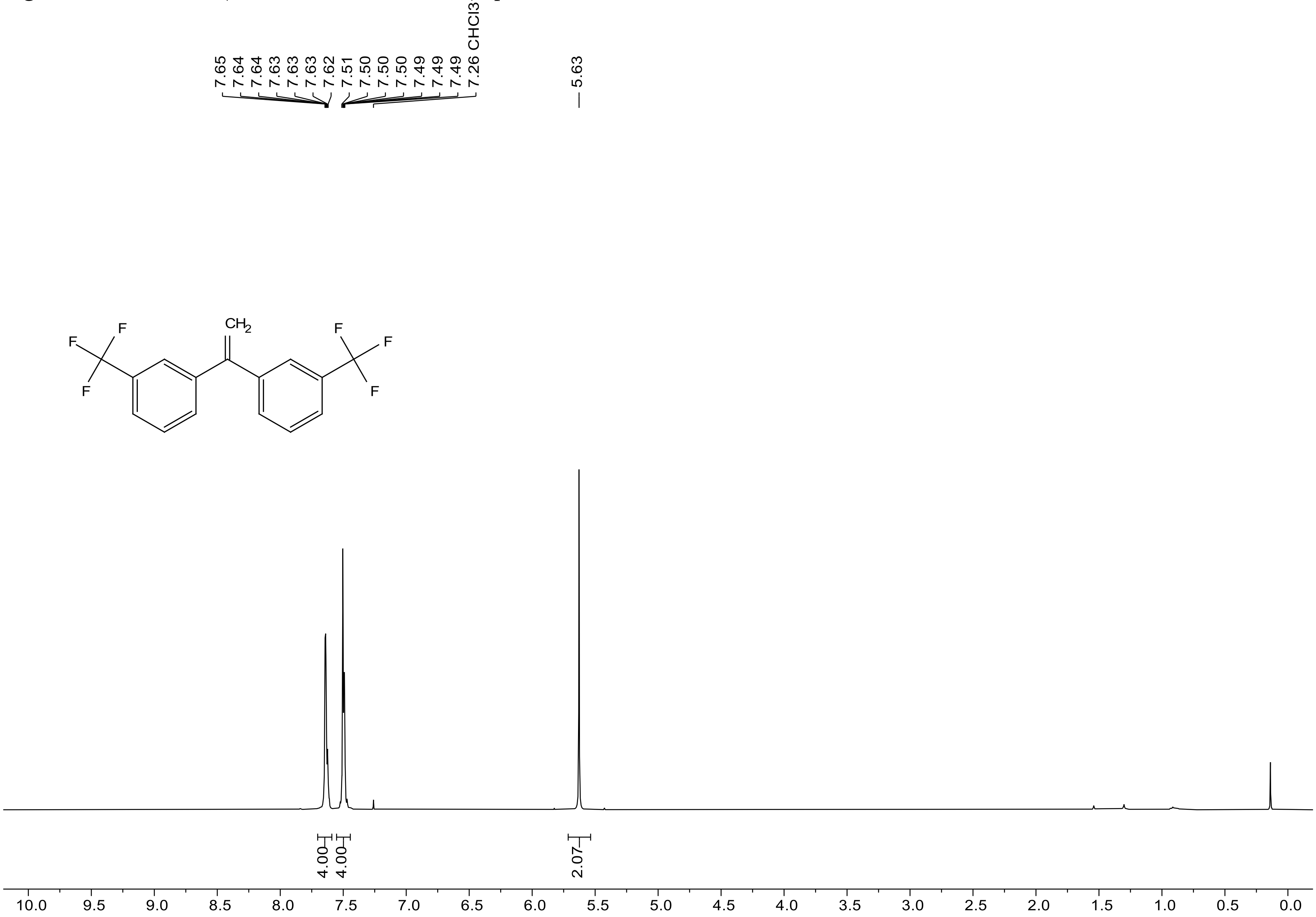

Figure S15: ^{13}**C NMR** (101 MHz, $CDCl_3$, 298 K) spectrum of **1h**.

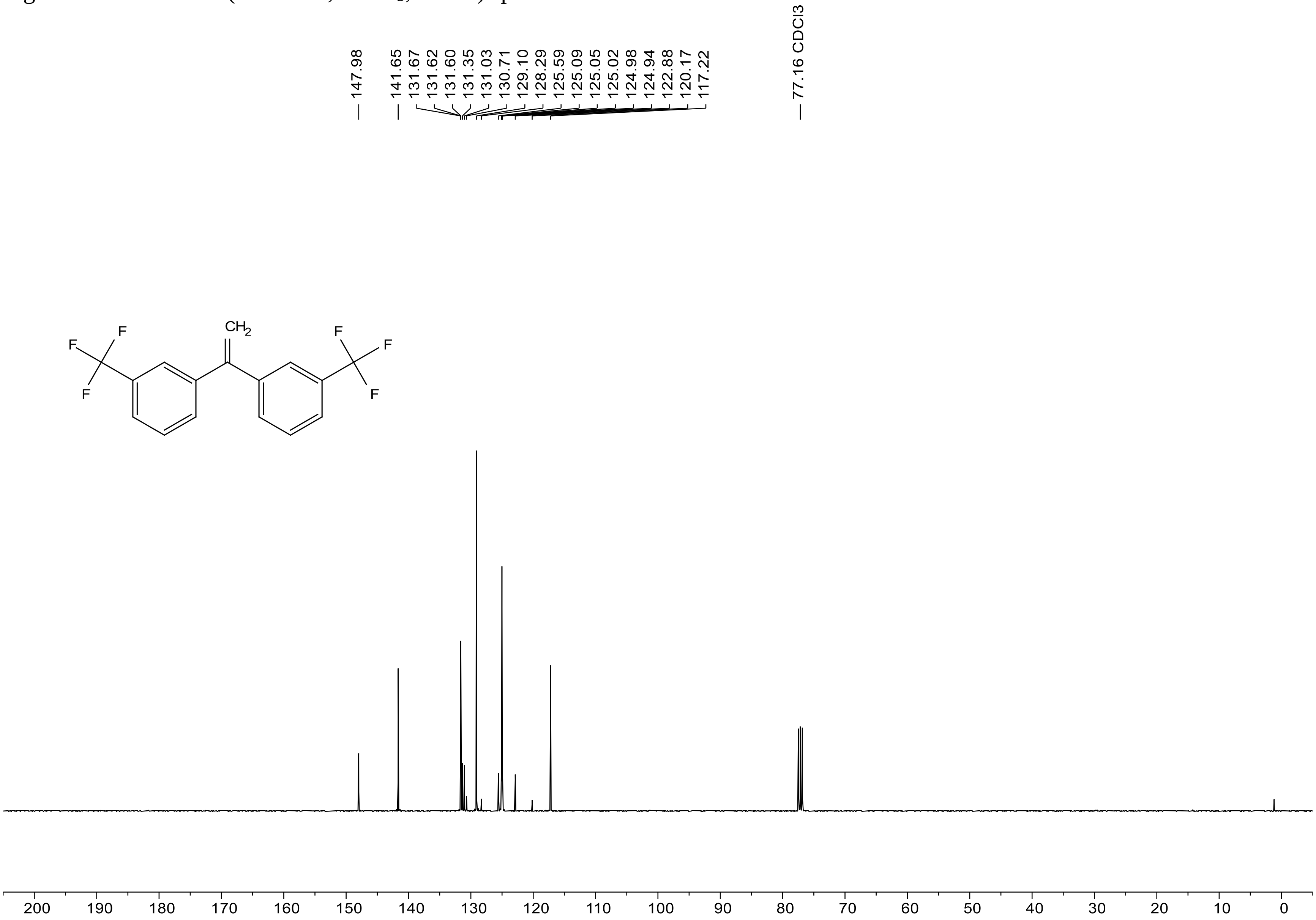

Figure S16: ^{19}**F NMR** (376 MHz, $CDCl_3$, 298 K) spectrum of **1h**.

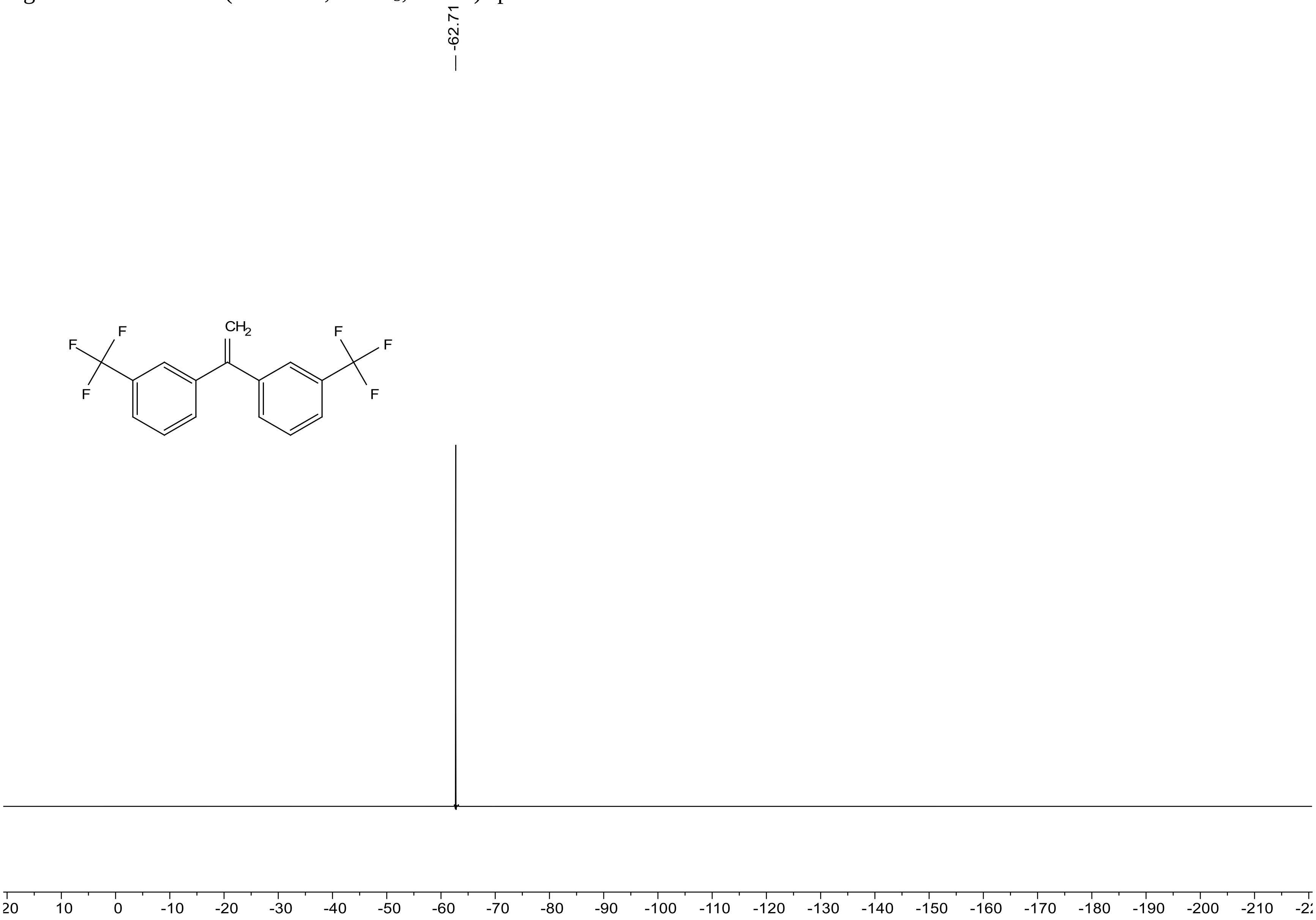

Figure S17: ^{1}**H NMR** (400 MHz, $CDCl_3$, 298 K) spectrum of **1i**.

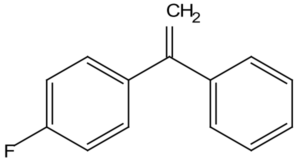

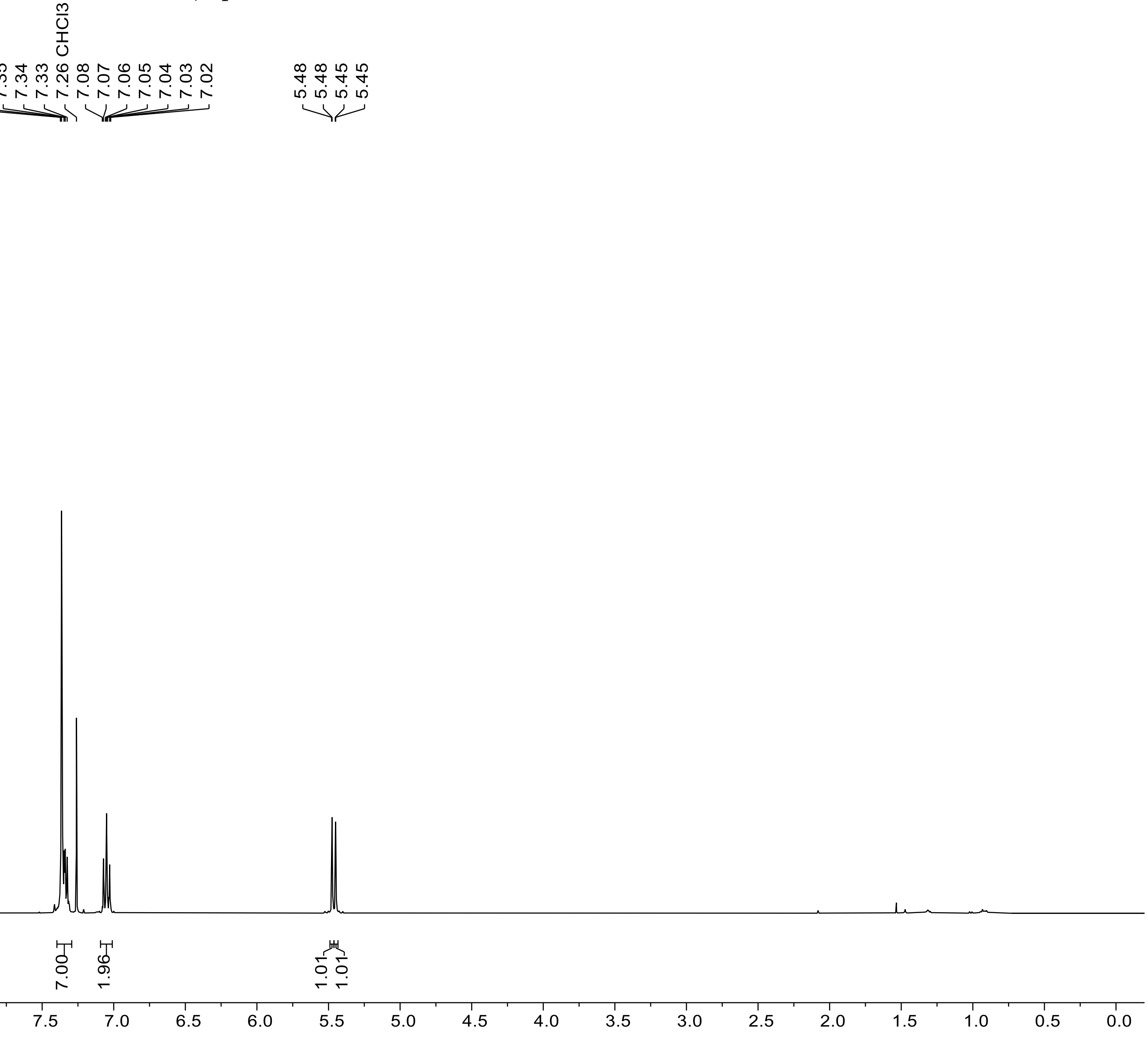

Figure S18: ^{13}**C NMR** (101 MHz, $CDCl_3$, 298 K) spectrum of **1i**.

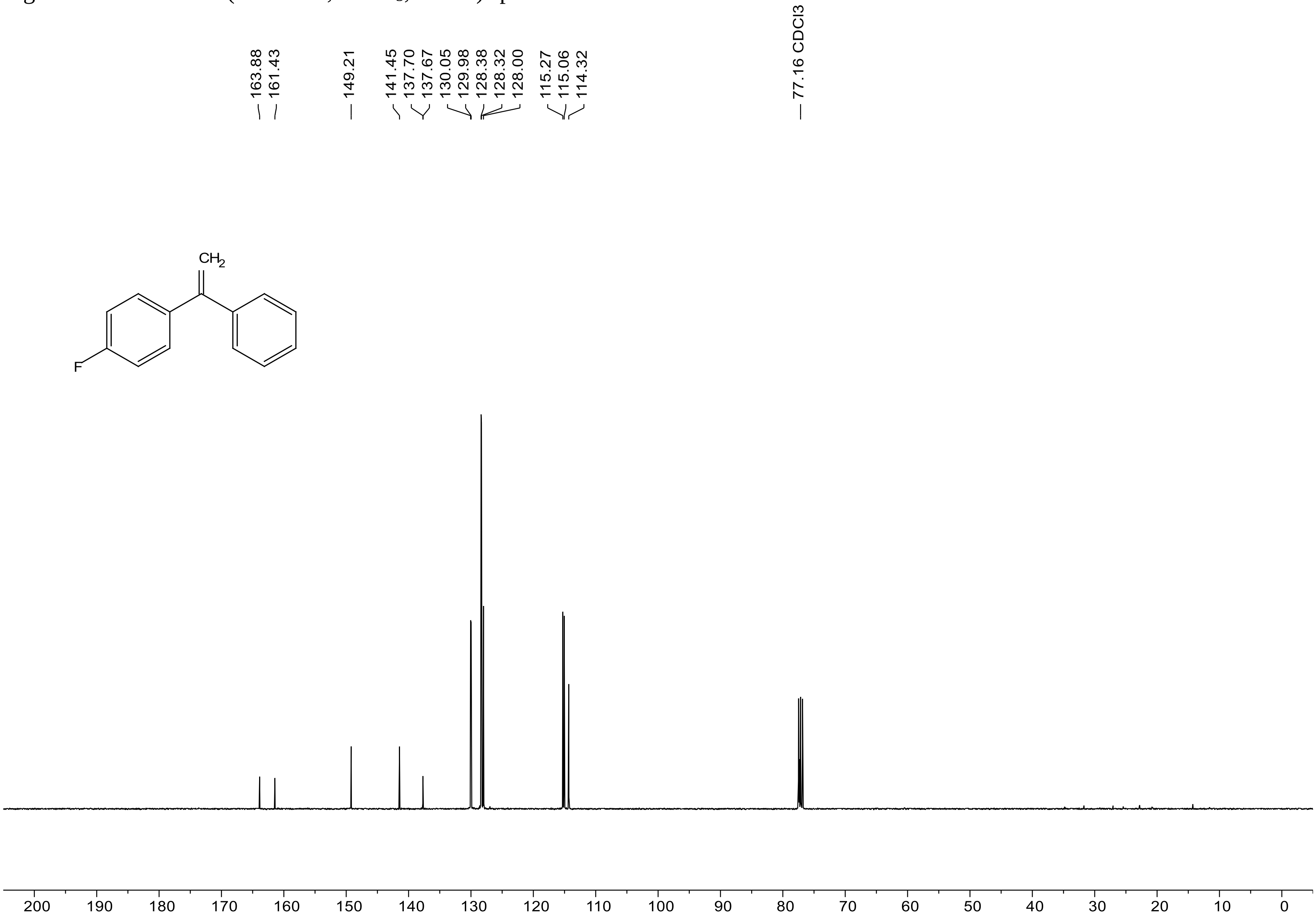

Figure S19: **^{19}F NMR** (376 MHz, $CDCl_3$, 298 K) spectrum of **1i**.

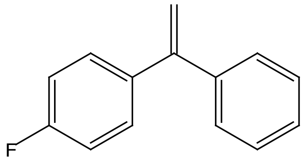

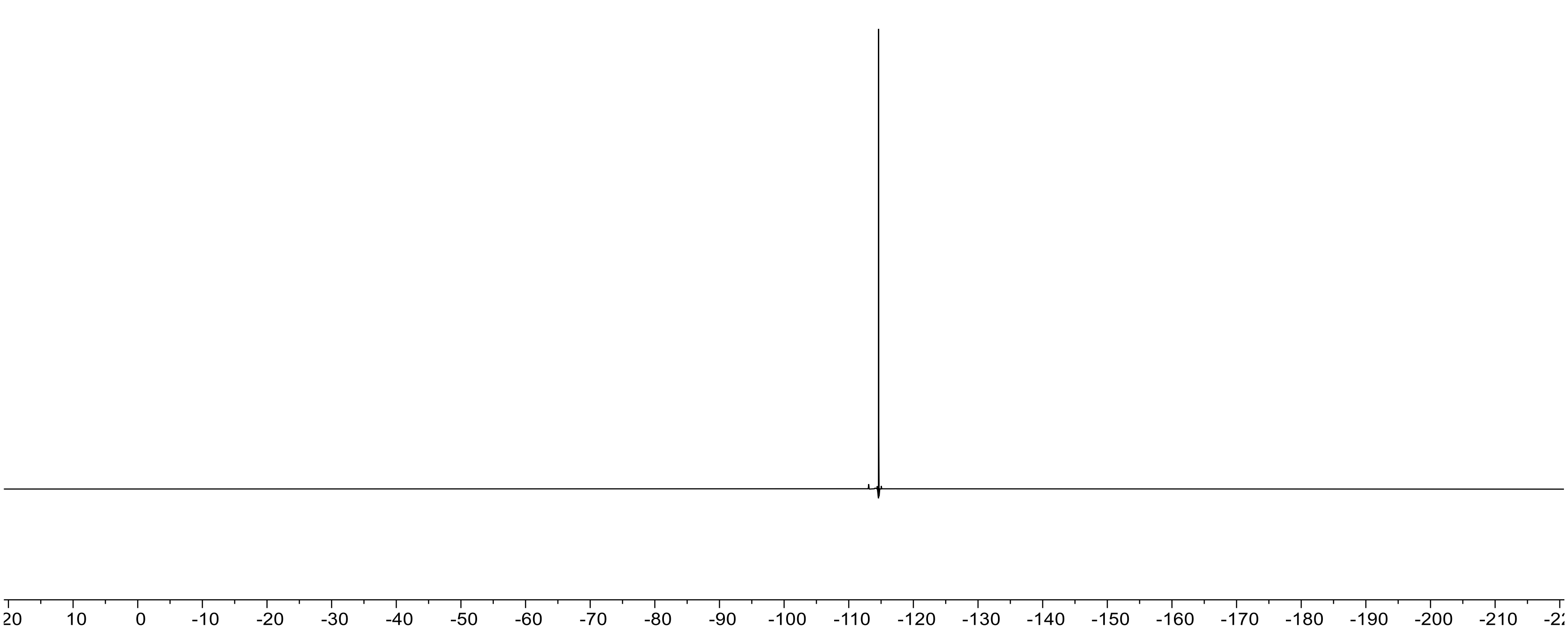

Figure S20: **^{1}H NMR** (400 MHz, $CDCl_3$, 298 K) spectrum of **1j**.

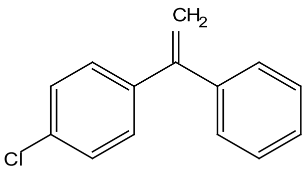

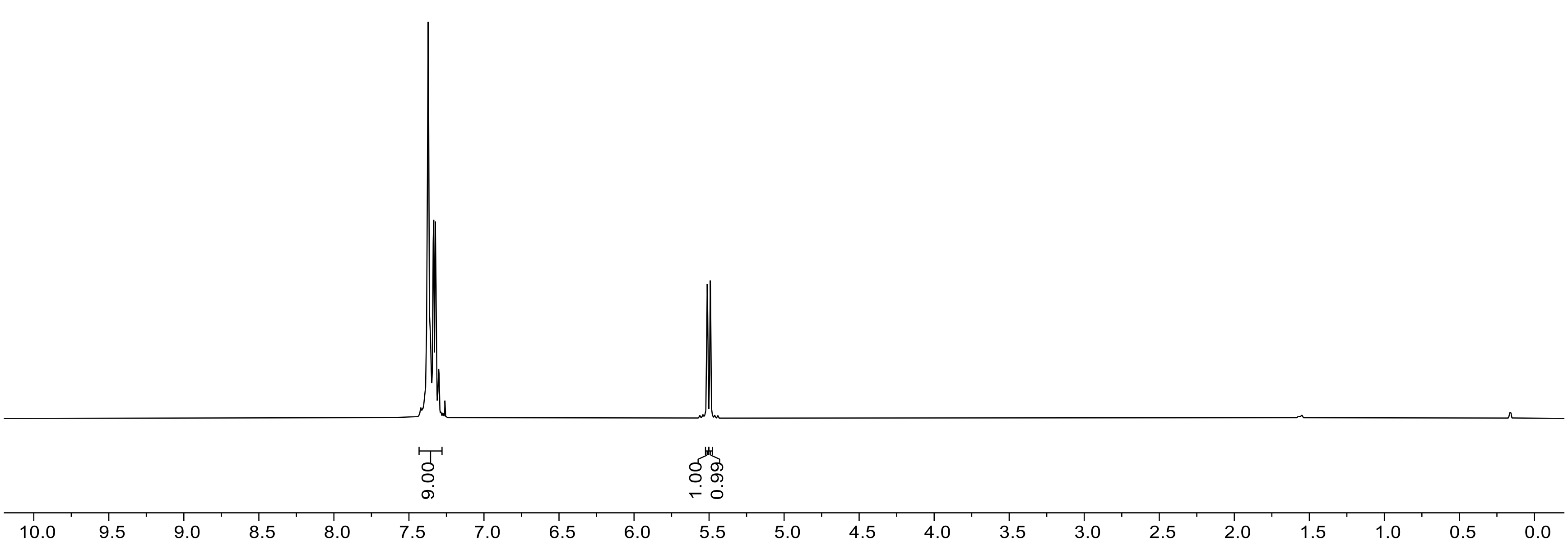

Figure S21: ^{13}**C NMR** (101 MHz, $CDCl_3$, 298 K) spectrum of **1j**.

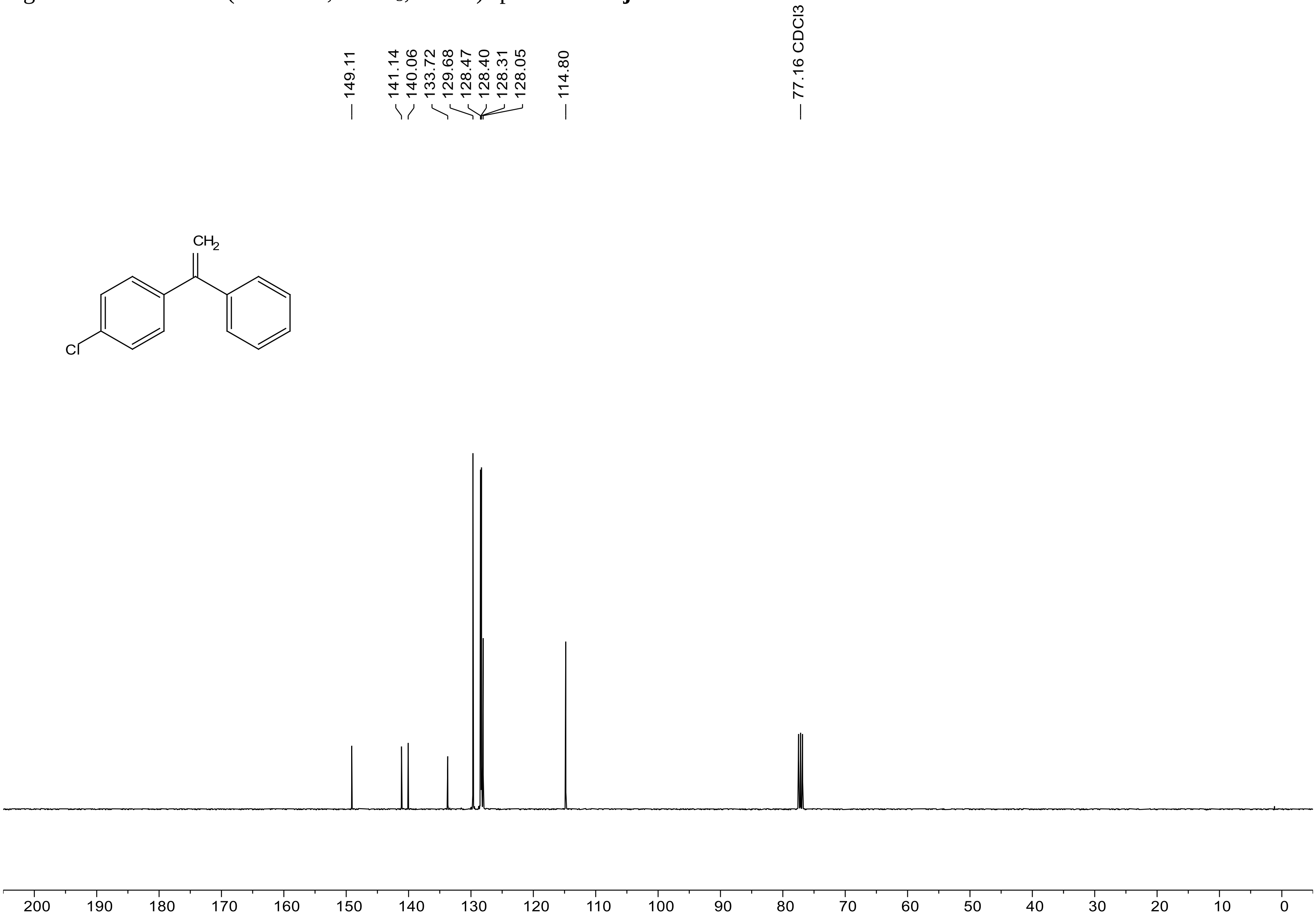

Figure S22: **^{1}H NMR** (400 MHz, $CDCl_3$, 298 K) spectrum of **1k**.

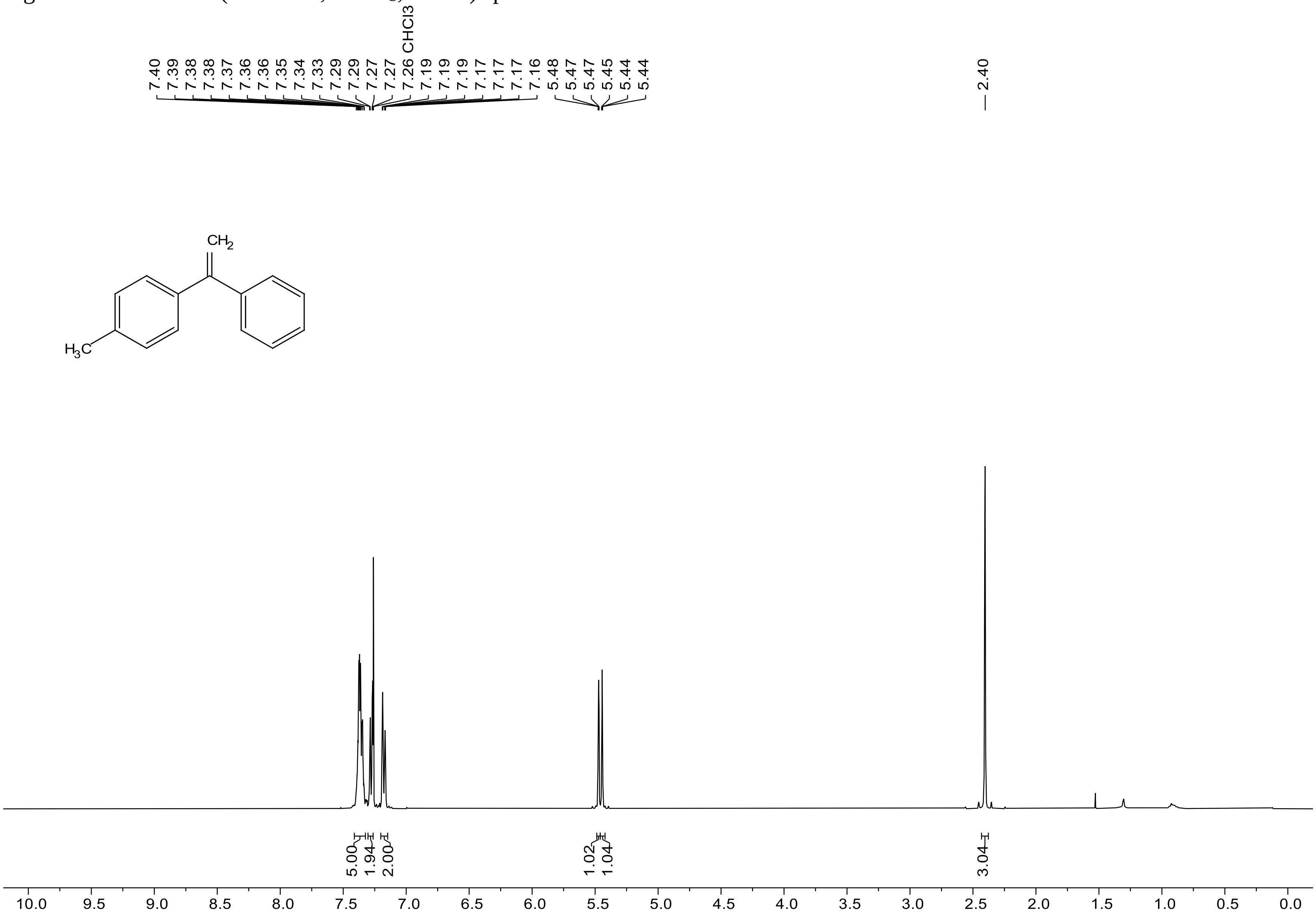

Figure S23: **^{13}C NMR** (101 MHz, $CDCl_3$, 298 K) spectrum of **1k**.

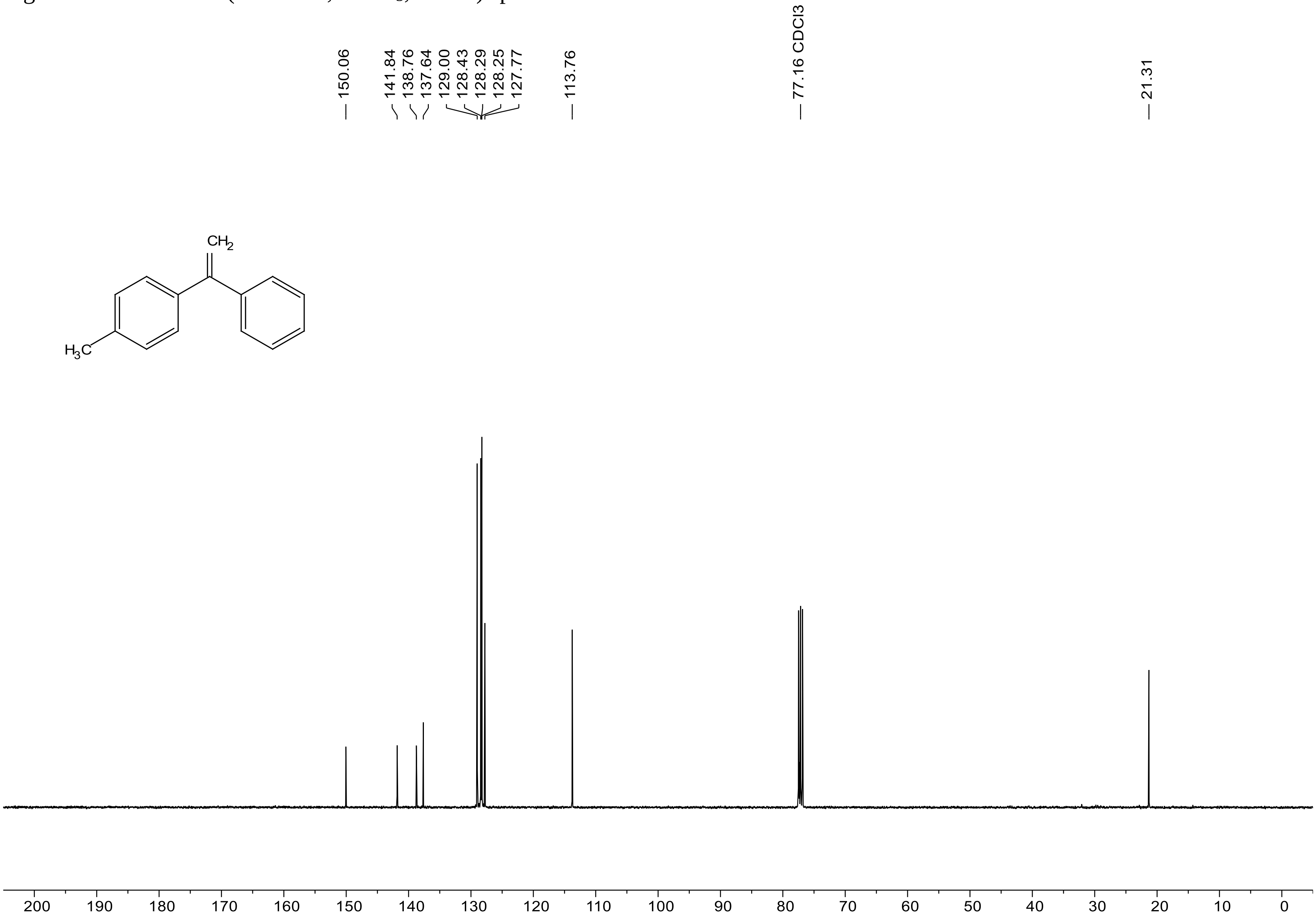

Figure S24: **^{1}H NMR** (400 MHz, $CDCl_3$, 298 K) spectrum of **1l**.

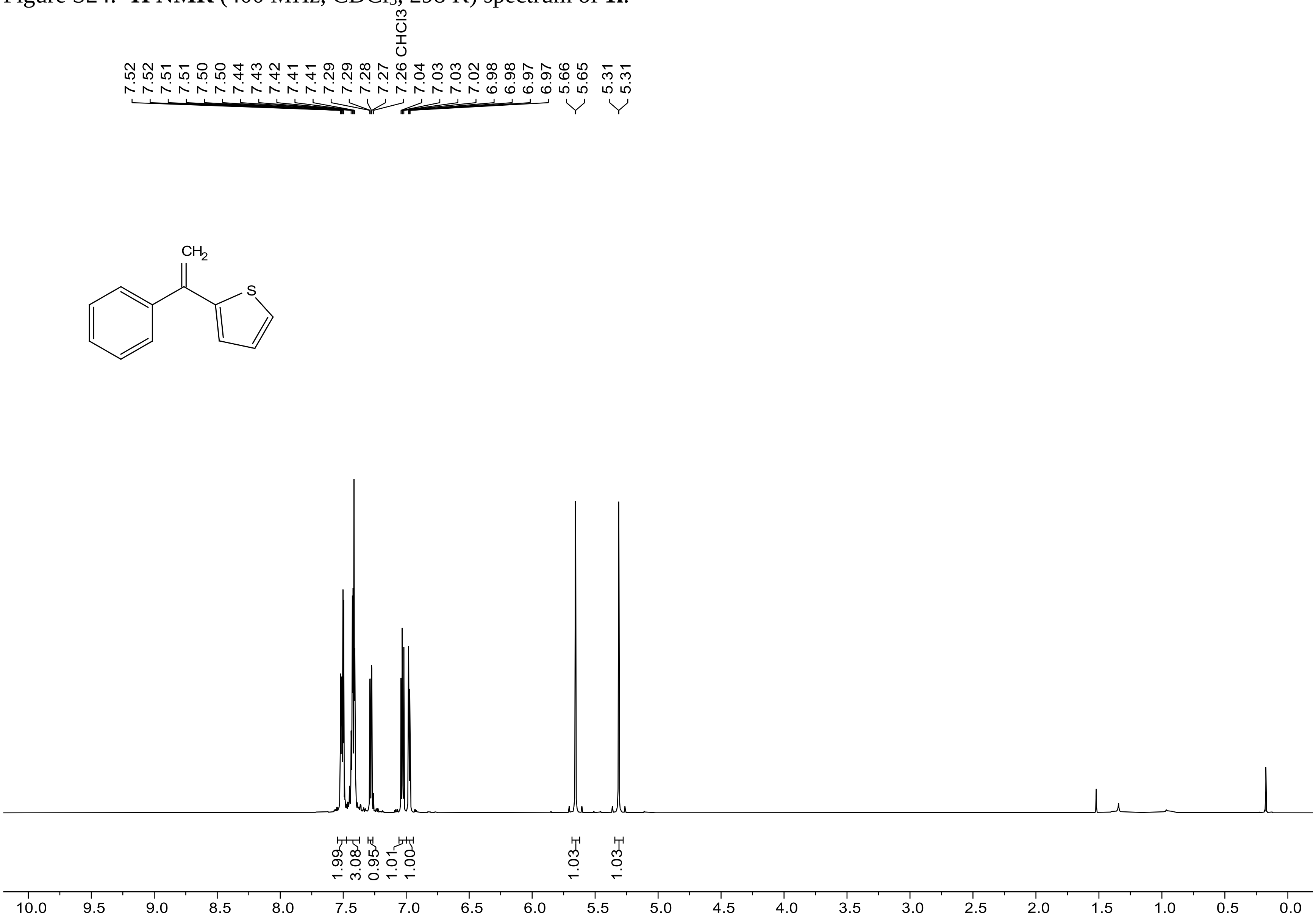

Figure S25: ^{13}**C NMR** (101 MHz, $CDCl_3$, 298 K) spectrum of **1l**.

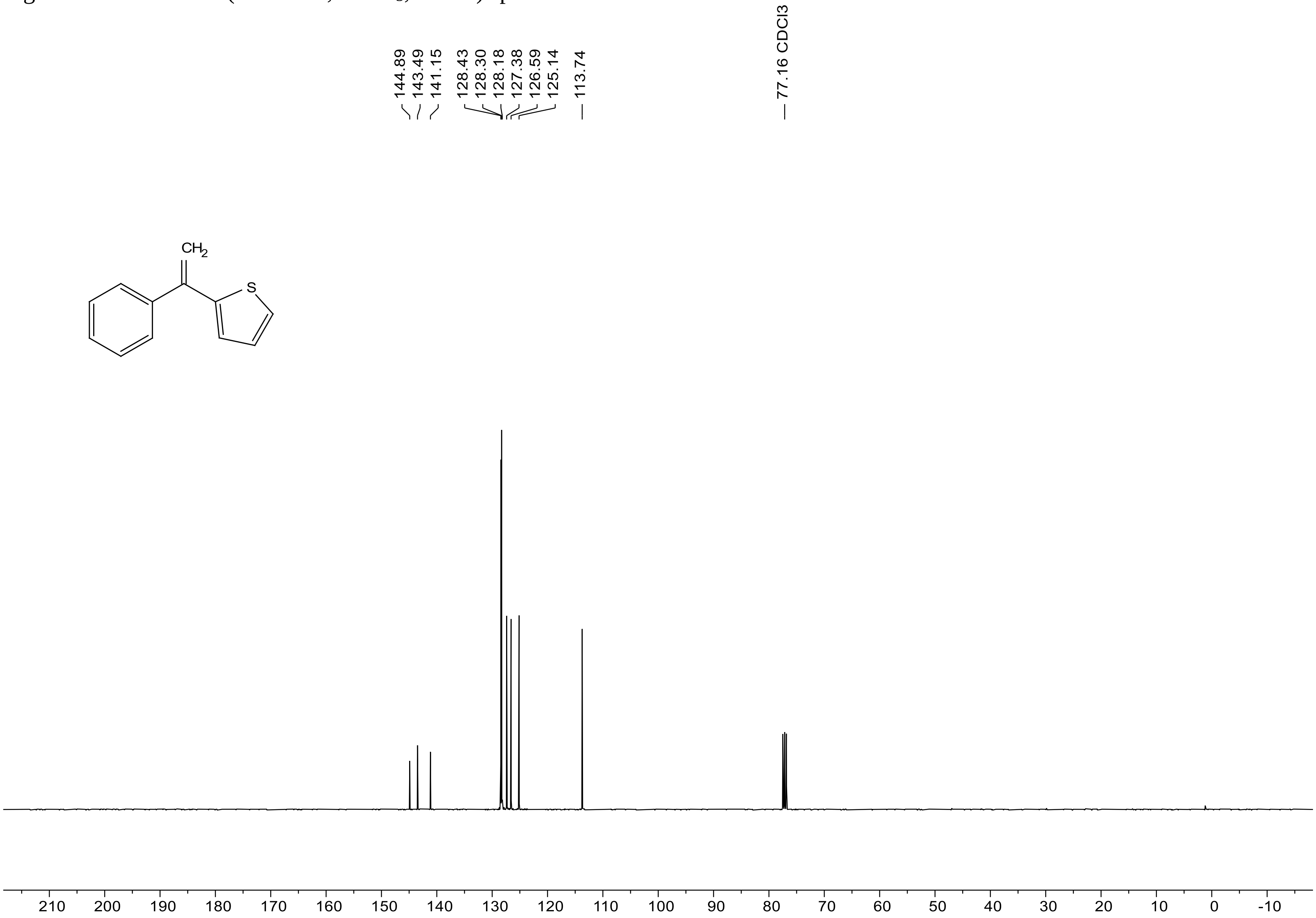

Figure S26: ^{1}**H NMR** (400 MHz, $CDCl_3$, 298 K) spectrum of **1m**.

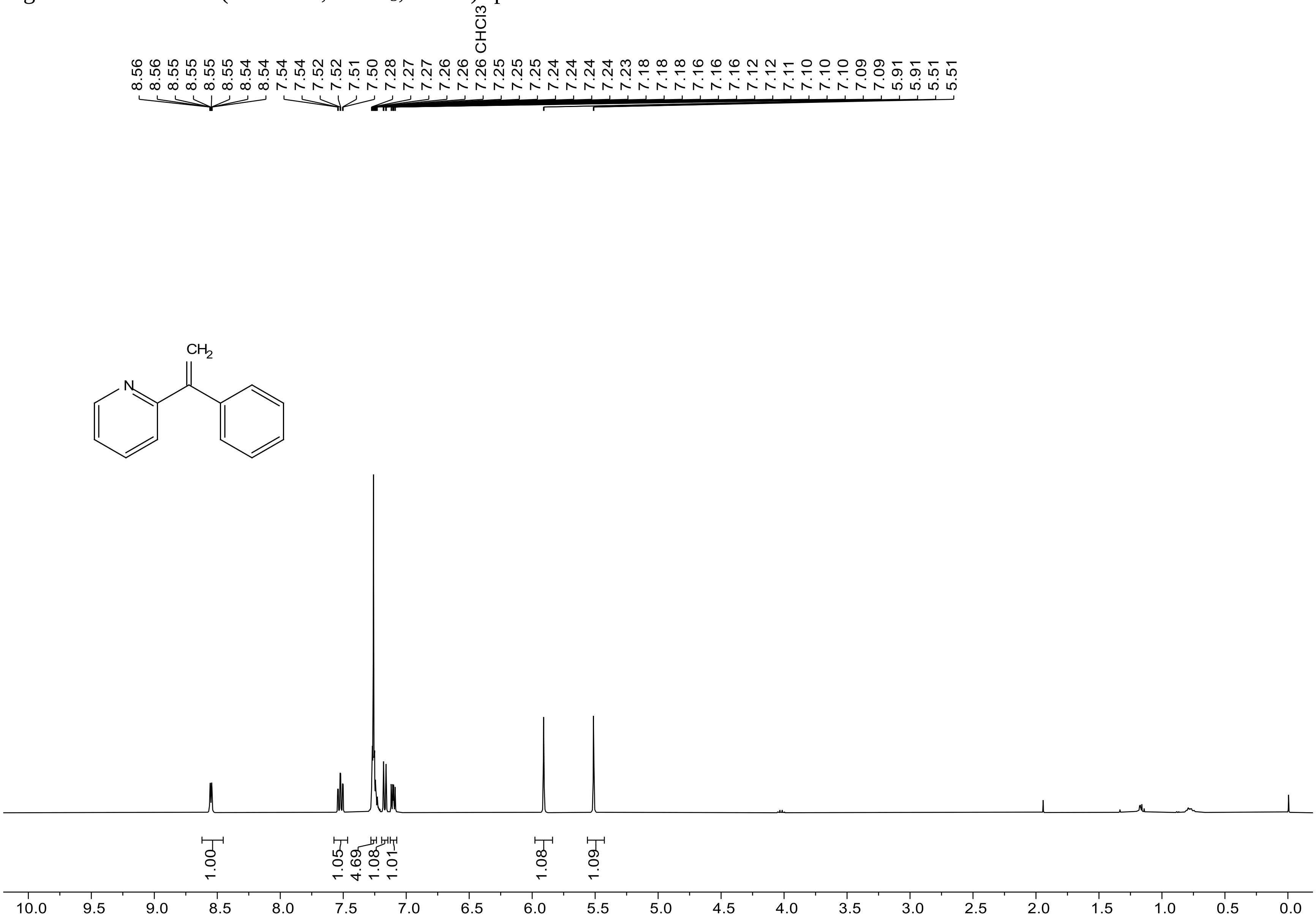

Figure S27: **^{13}C NMR** (101 MHz, $CDCl_3$, 298 K) spectrum of **1m**.

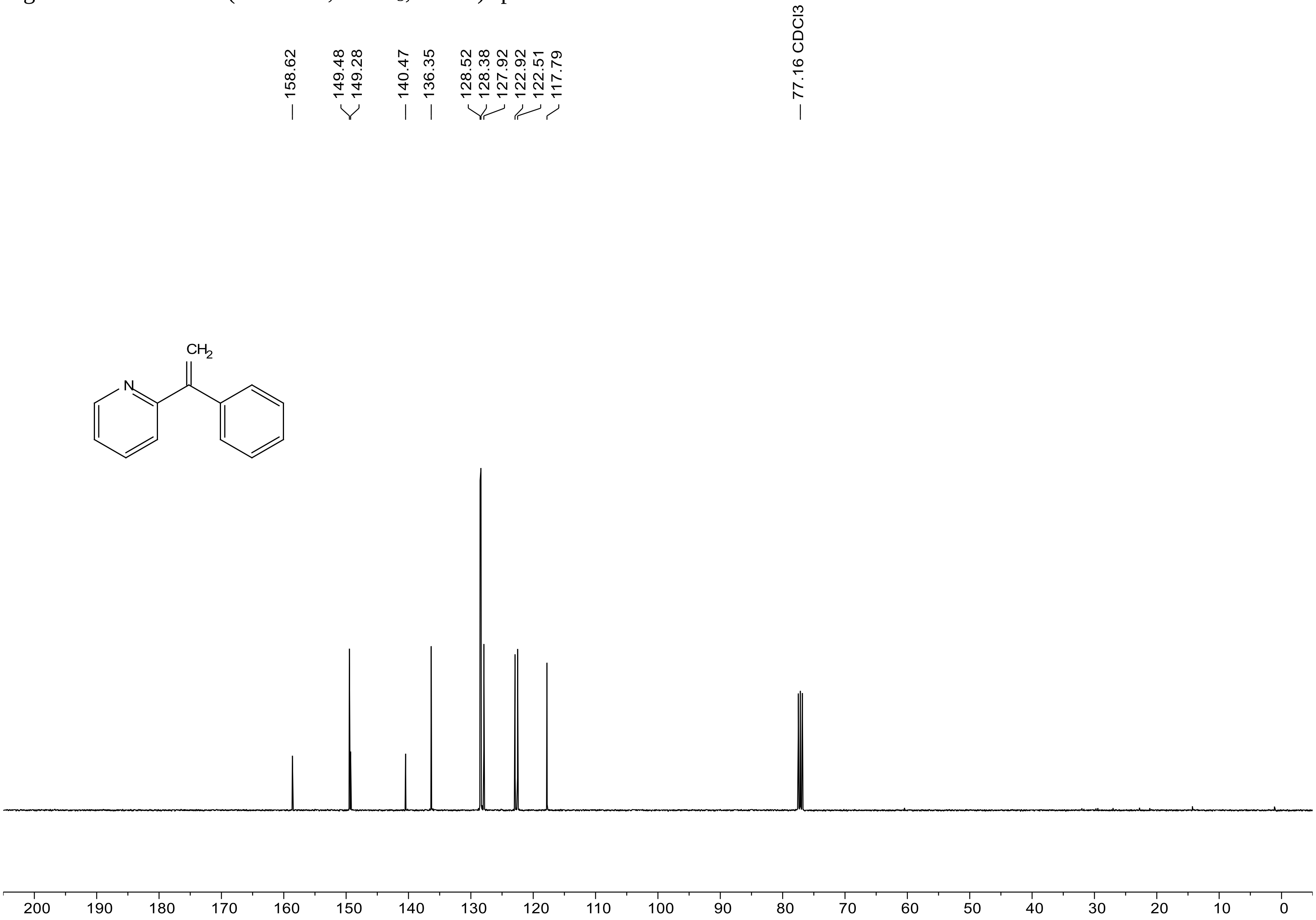

Figure S28: ^{1}**H NMR** (400 MHz, $CDCl_3$, 298 K) spectrum of **1n**.

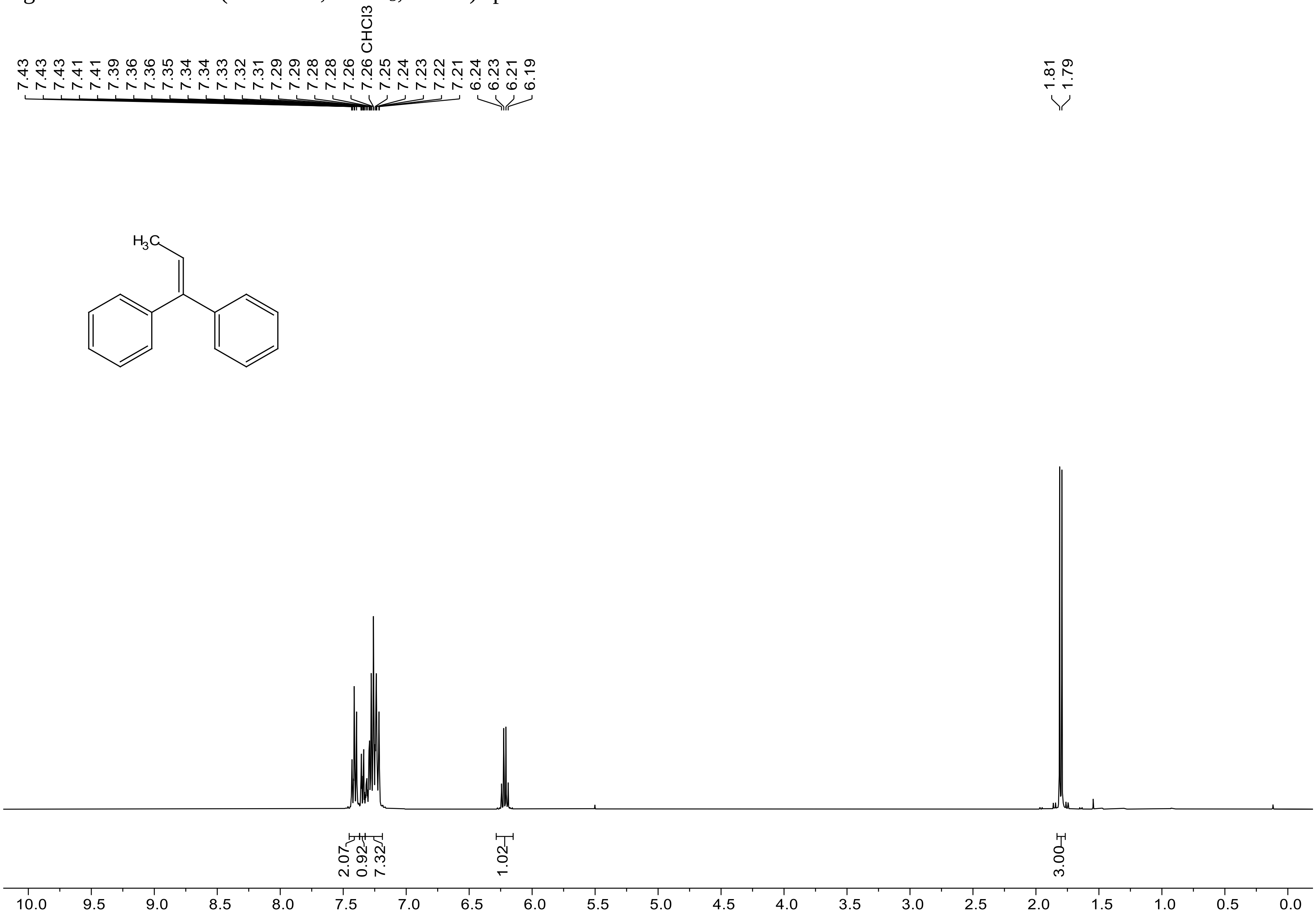

Figure S29: **^{13}C NMR** (101 MHz, $CDCl_3$, 298 K) spectrum of **1n**.

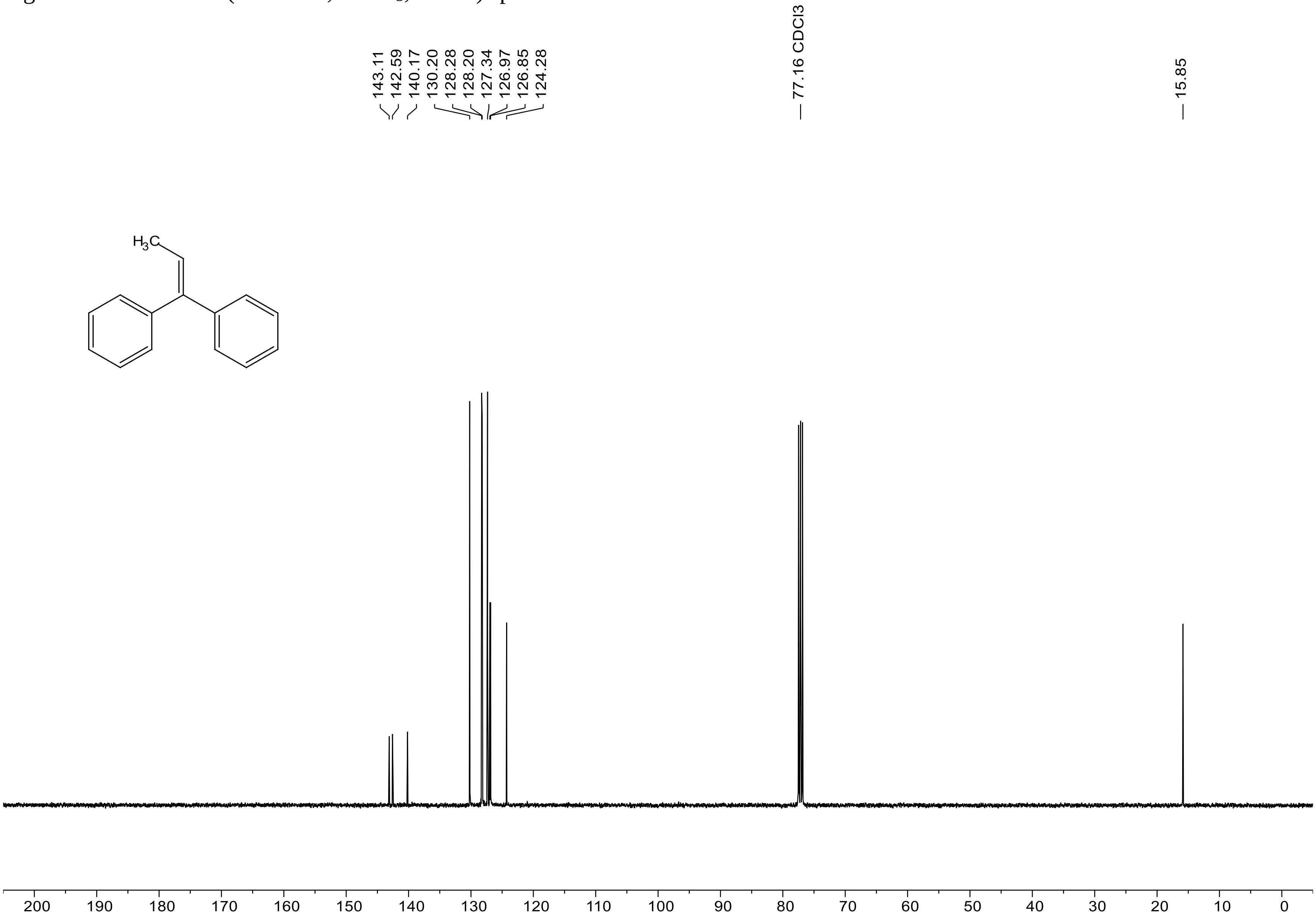

Figure S30: **^{1}H NMR** (400 MHz, $CDCl_3$, 298 K) spectrum of **1o**.

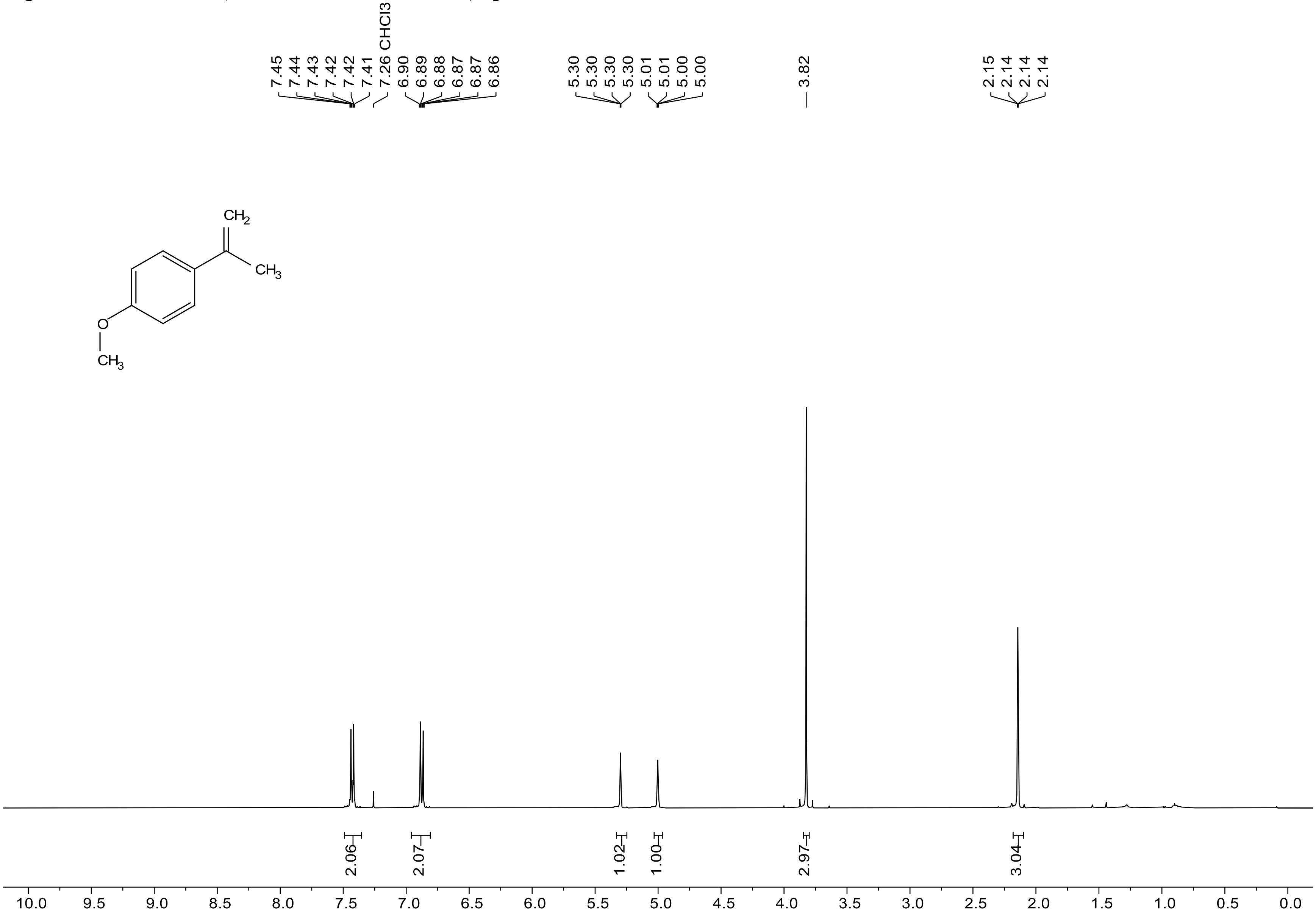

Figure S31: ^{13}**C NMR** (101 MHz, $CDCl_3$, 298 K) spectrum of **1o**.

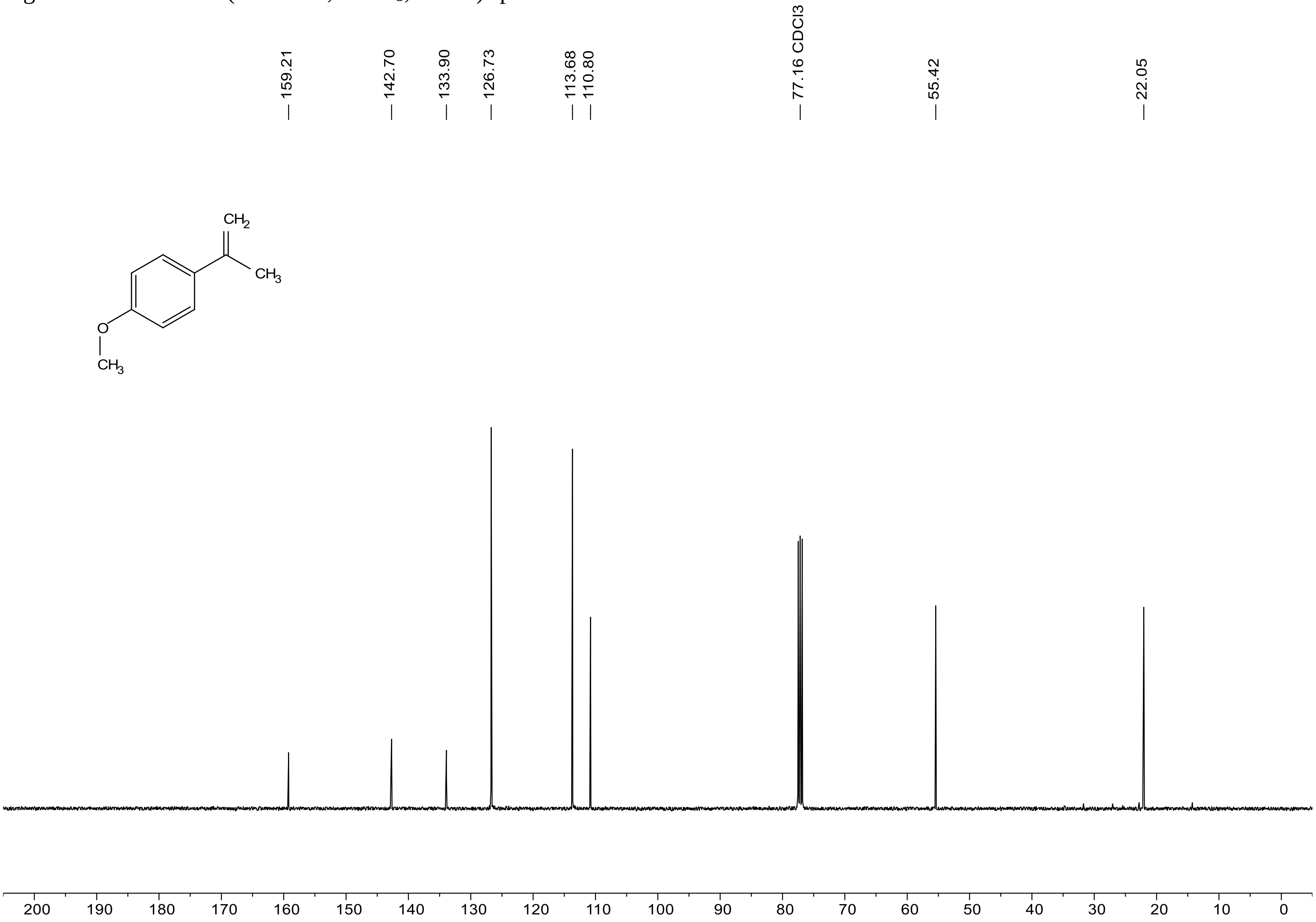

Figure S32: **^{1}H NMR** (400 MHz, $CDCl_3$, 298 K) spectrum of **1r**.

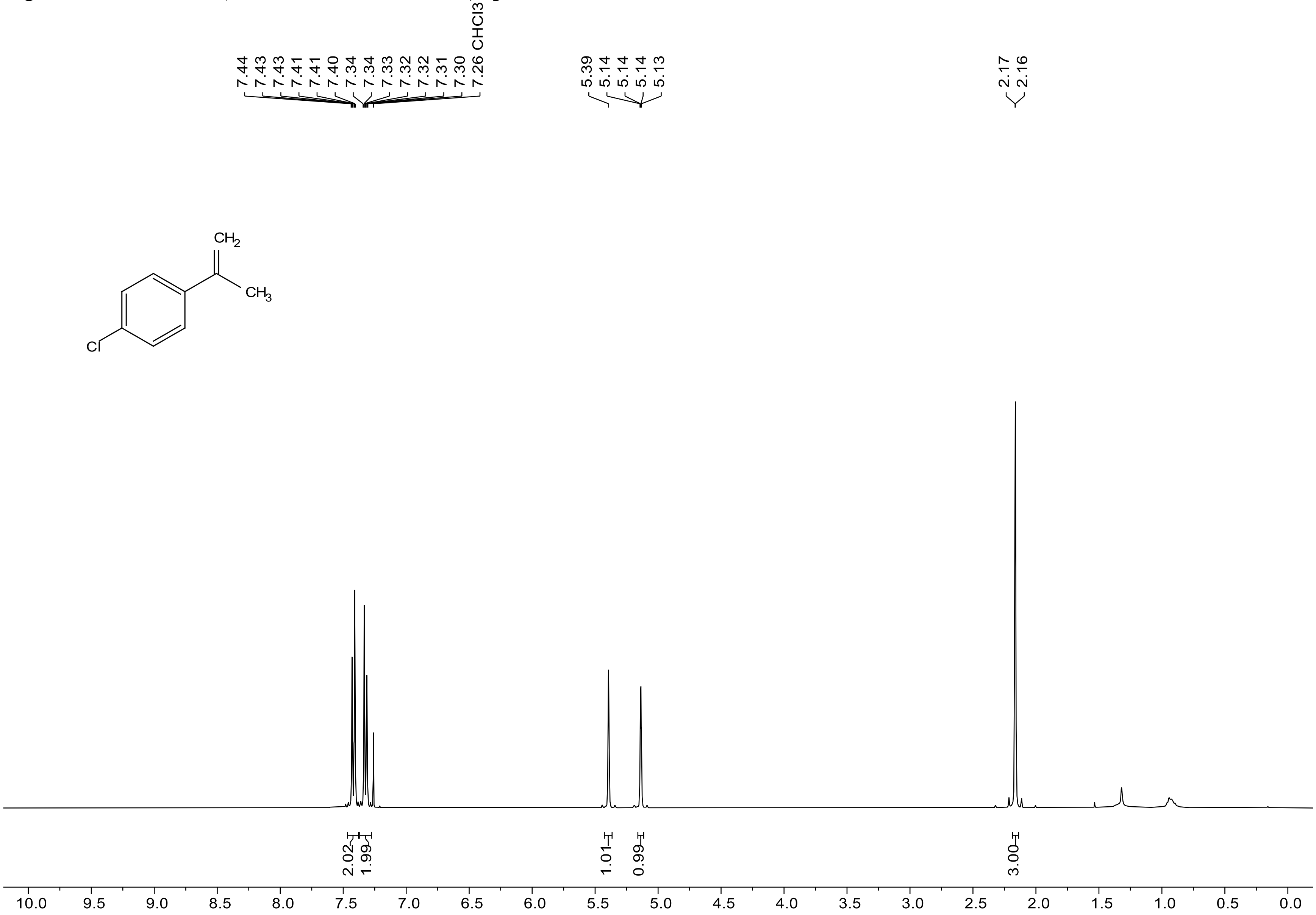

Figure S33: ^{13}**C NMR** (101 MHz, $CDCl_3$, 298 K) spectrum of **1r**.

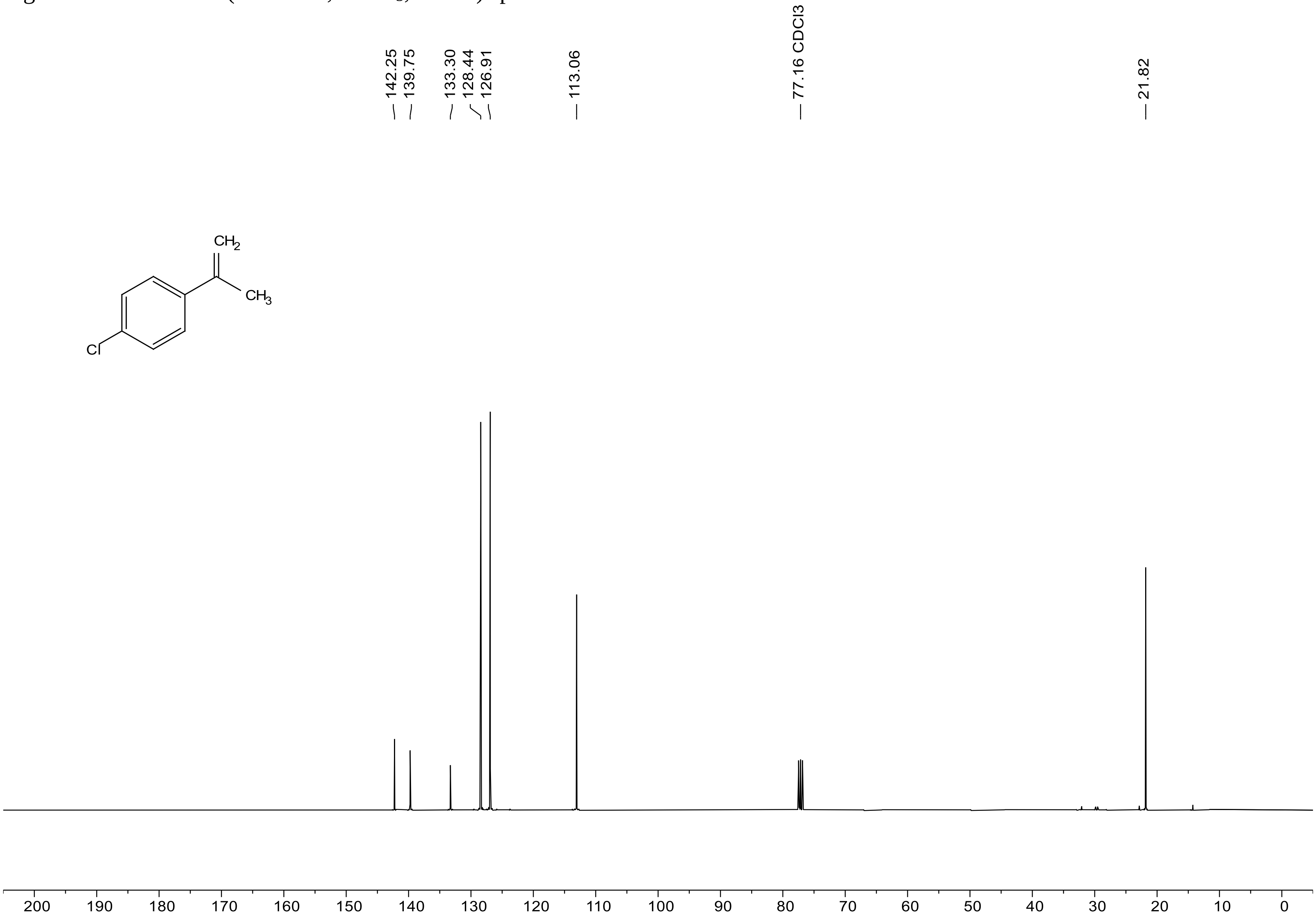

Figure S34: **^{1}H NMR** (400 MHz, $CDCl_3$, 298 K) spectrum of **1s**.

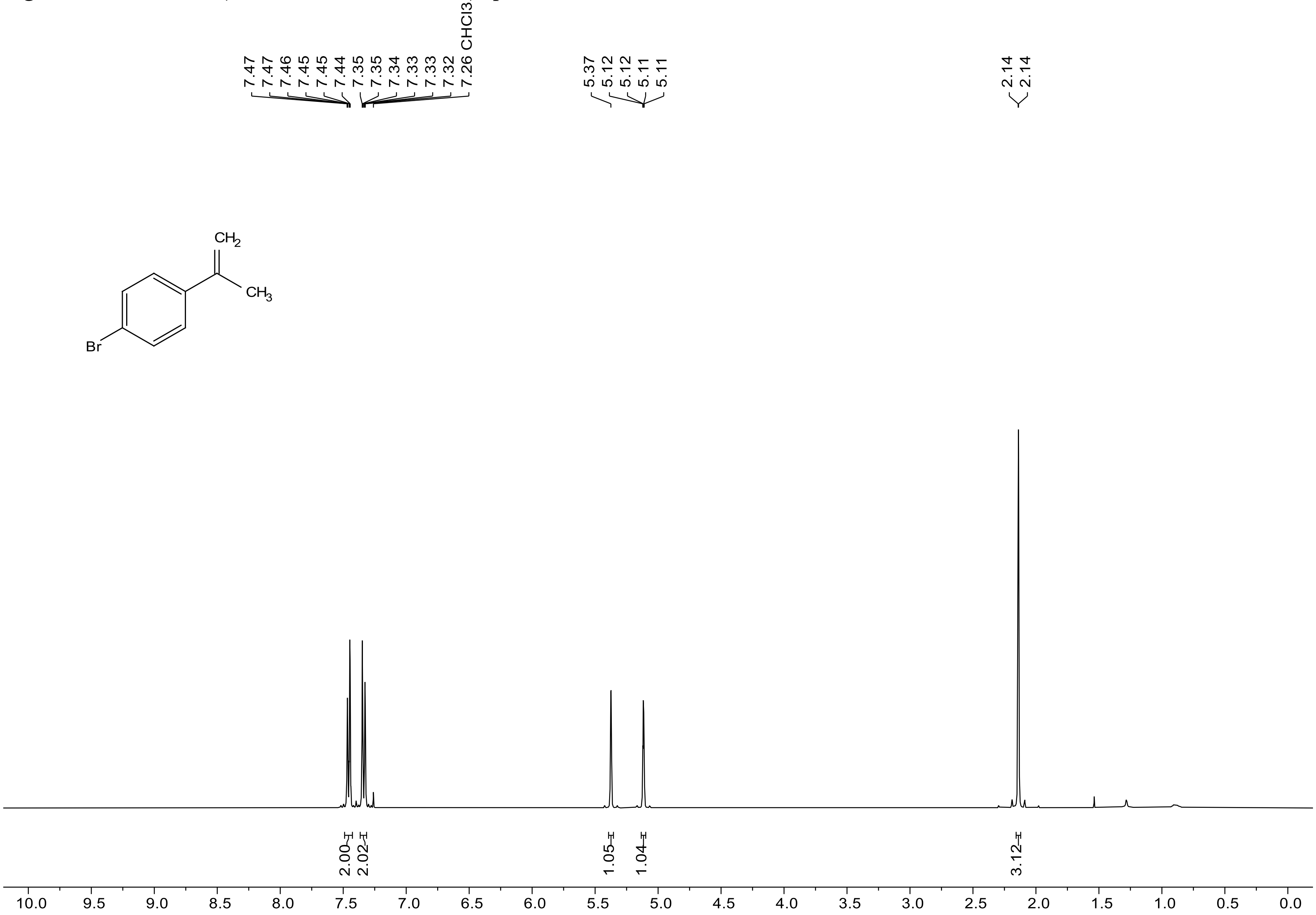

Figure S35: ^{13}**C NMR** (101 MHz, $CDCl_3$, 298 K) spectrum of **1s**.

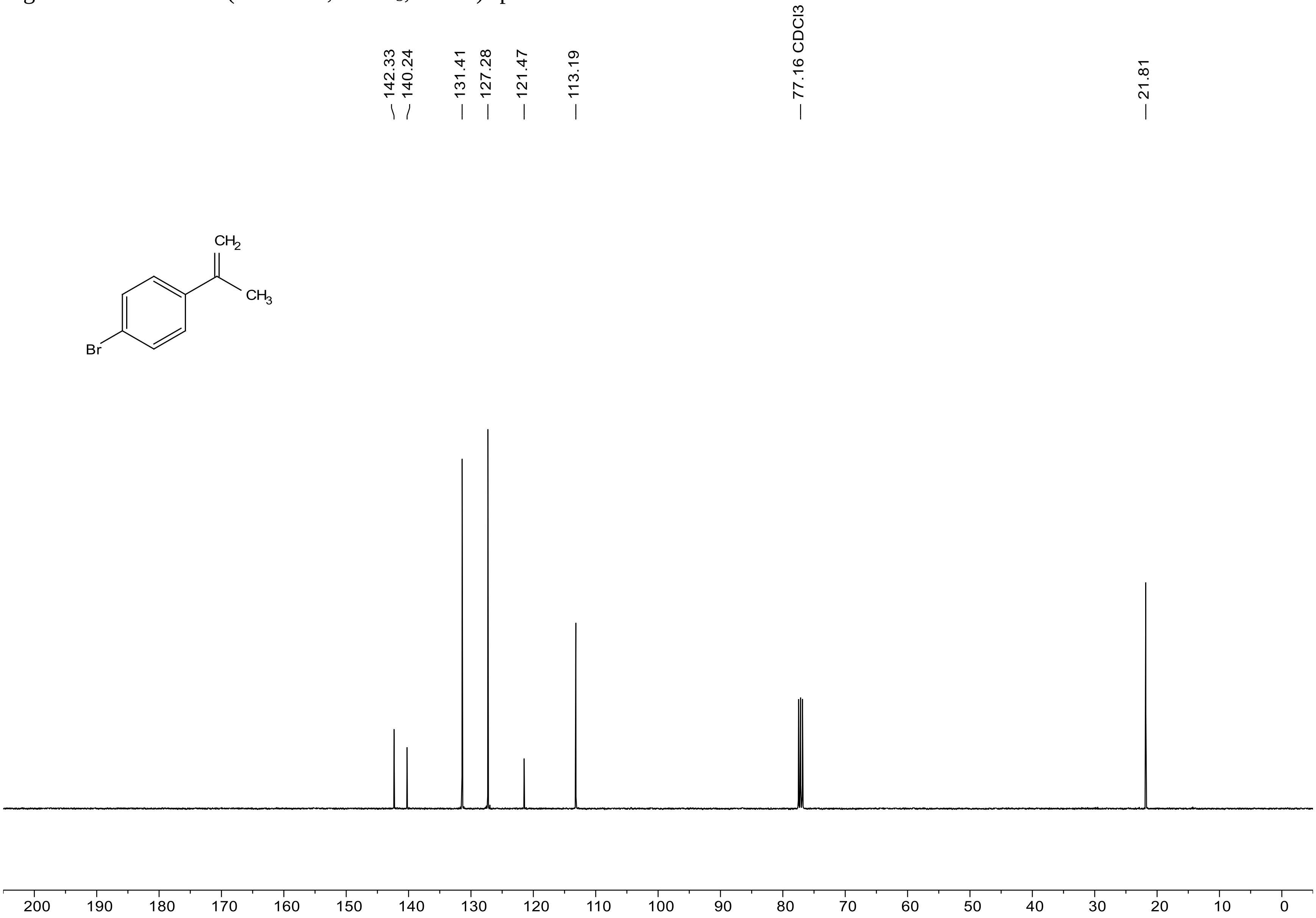

Figure S36: ^{1}**H NMR** (400 MHz, $CDCl_3$, 298 K) spectrum of **1t**.

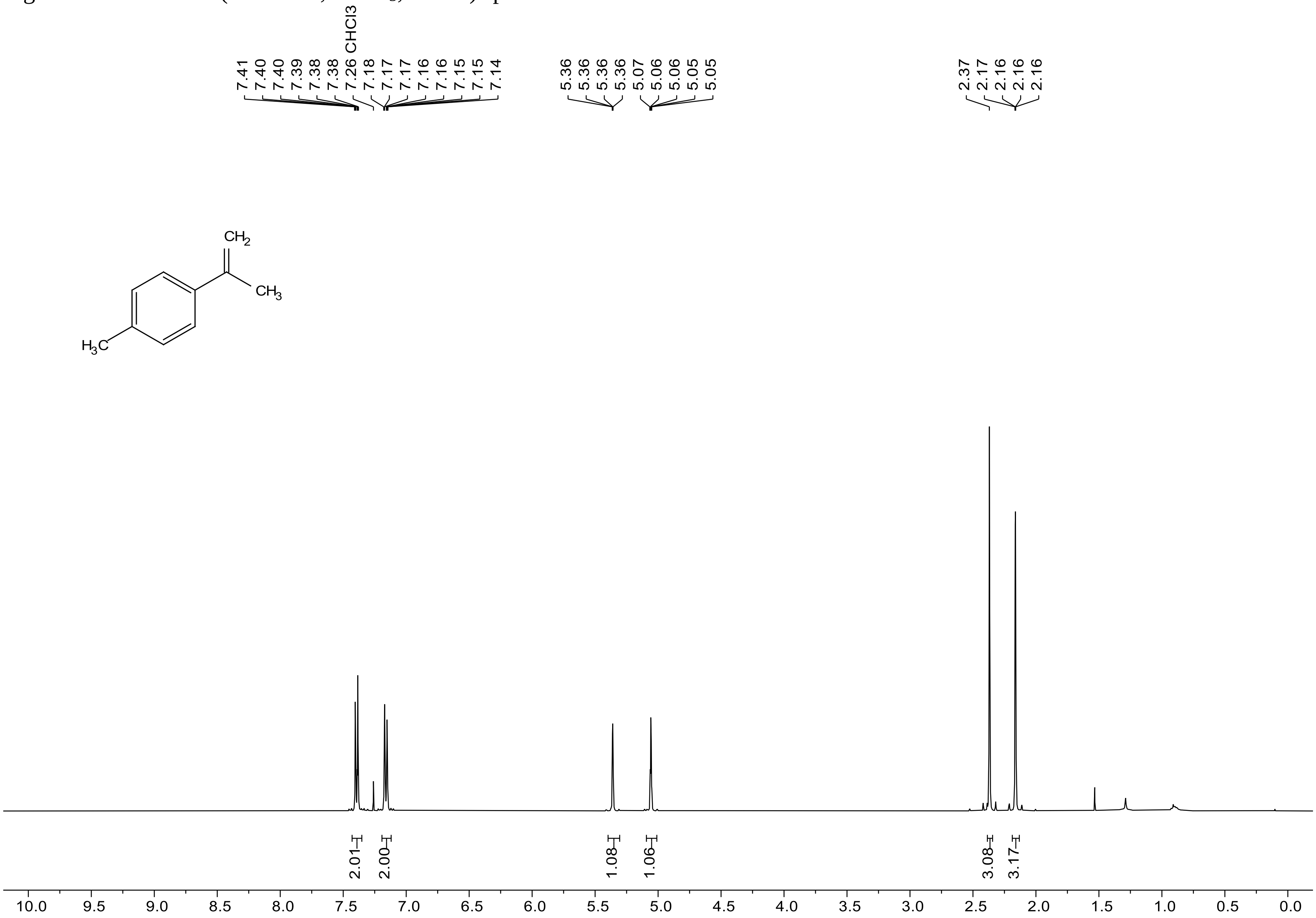

Figure S37: ^{13}**C NMR** (101 MHz, $CDCl_3$, 298 K) spectrum of **1t**.

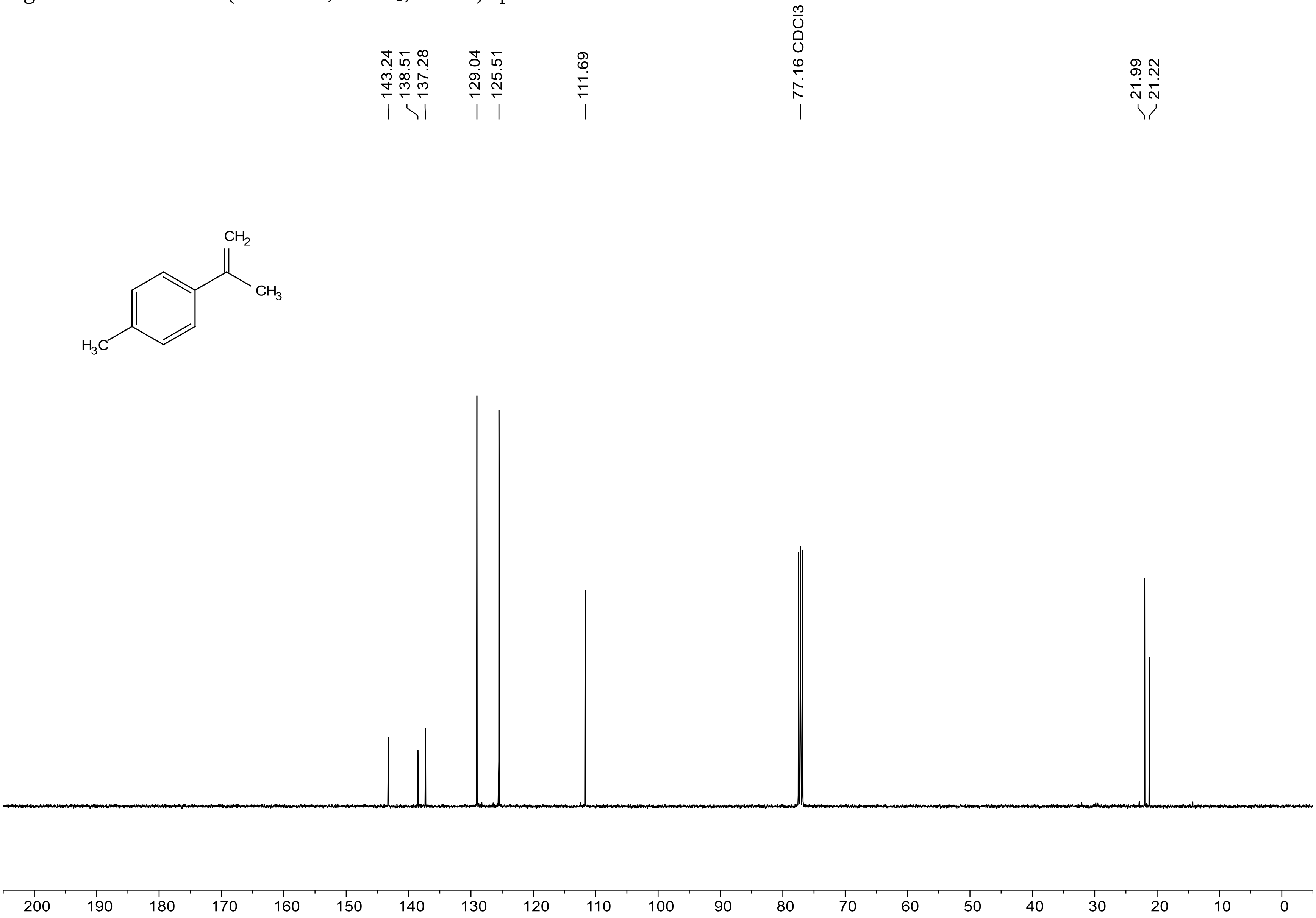

Figure S38: **^{1}H NMR** (400 MHz, $CDCl_3$, 298 K) spectrum of **1u**.

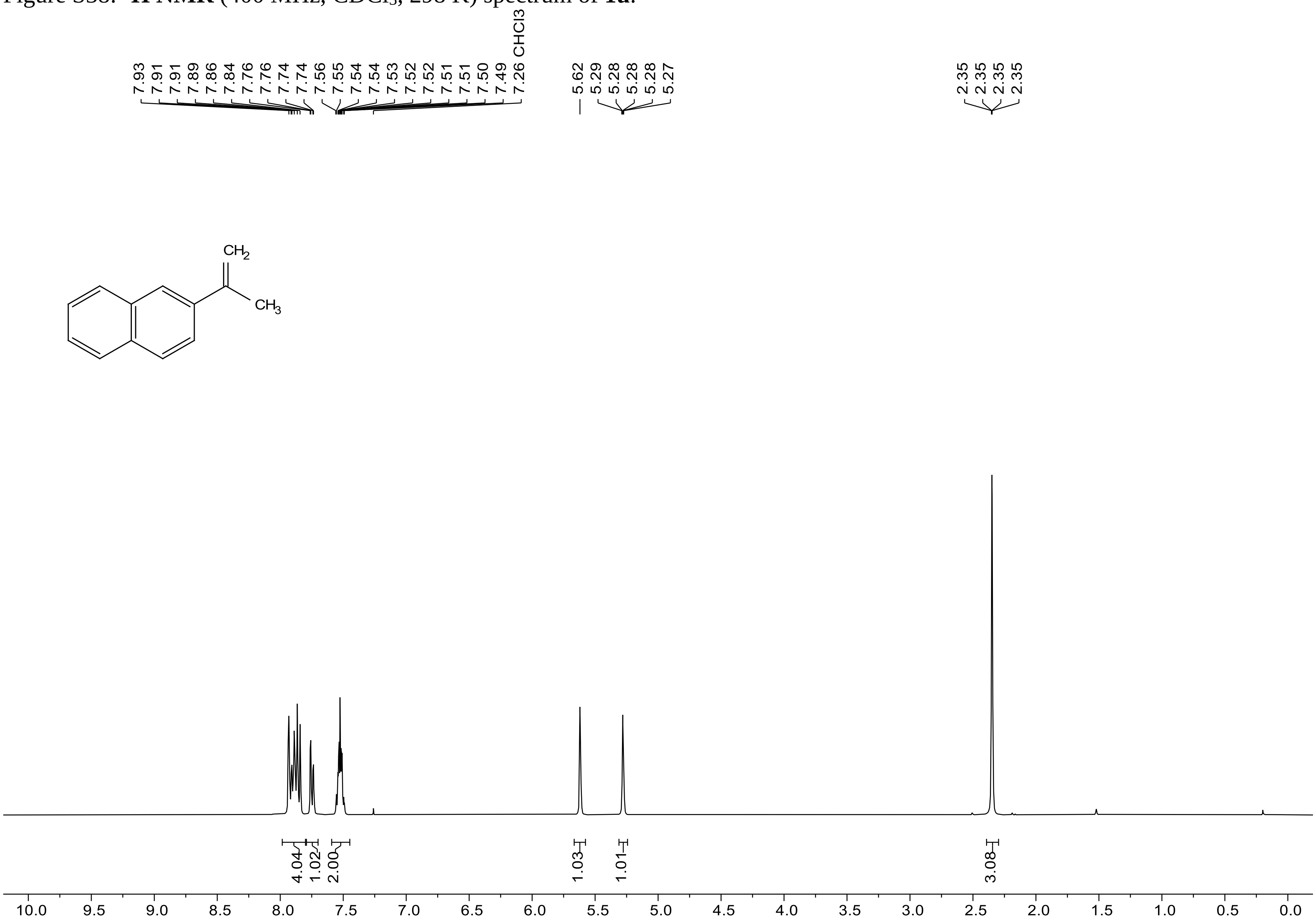

Figure S39: **^{13}C NMR** (101 MHz, $CDCl_3$, 298 K) spectrum of **1u**.

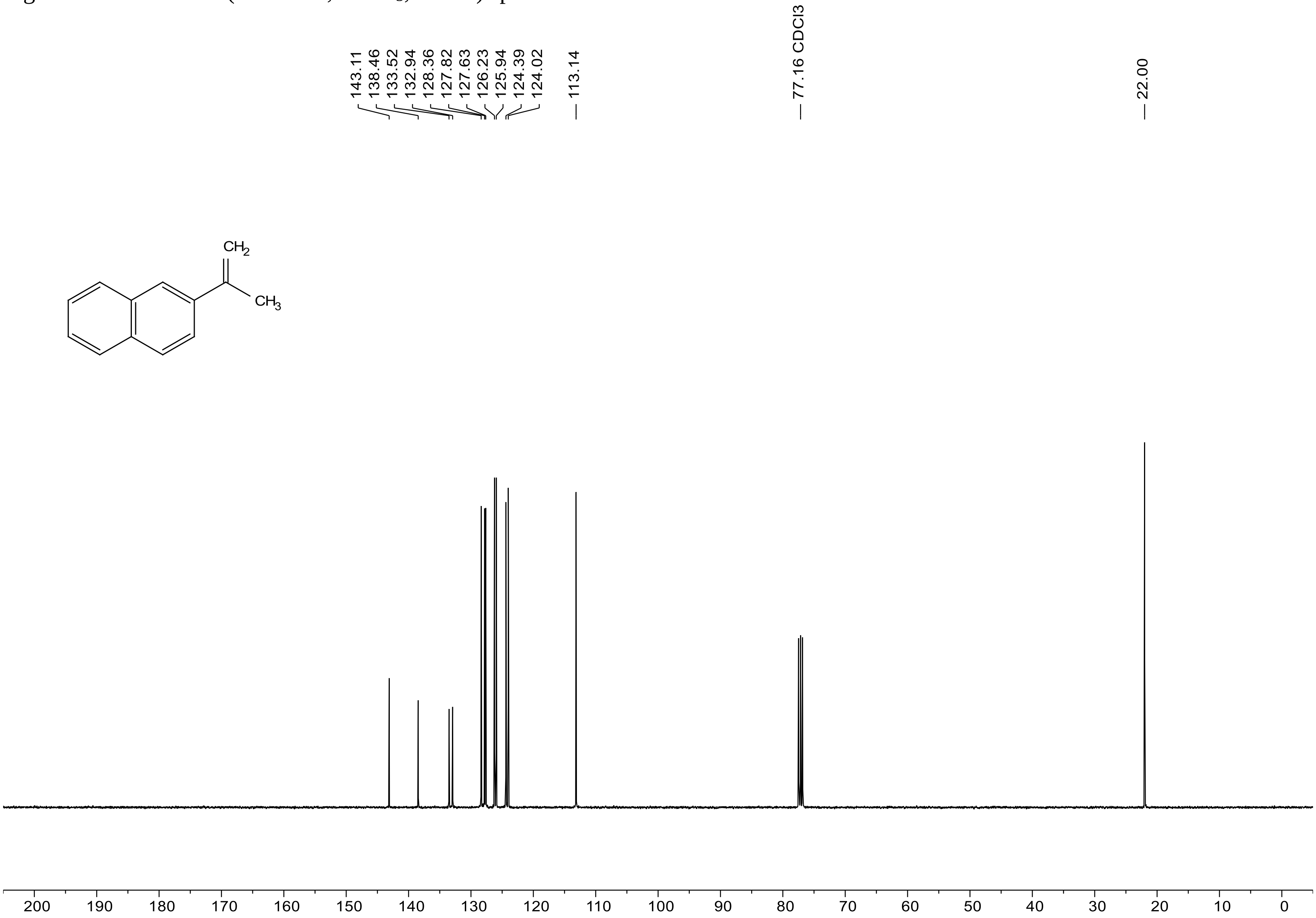

Figure S40: **^{1}H NMR** (400 MHz, $CDCl_3$, 298 K) spectrum of **1v**.

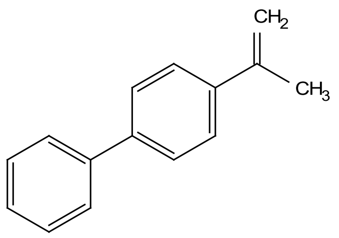

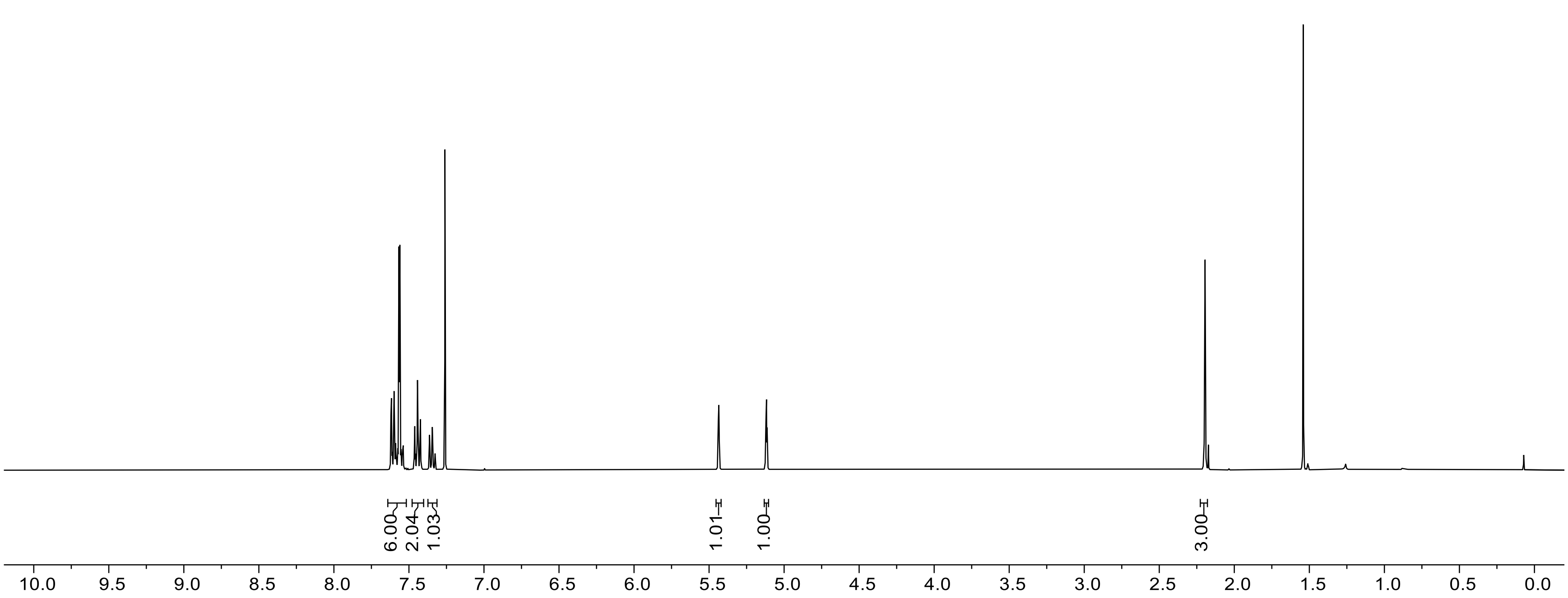

Figure S41: **^{13}C NMR** (101 MHz, $CDCl_3$, 298 K) spectrum of **1v**.

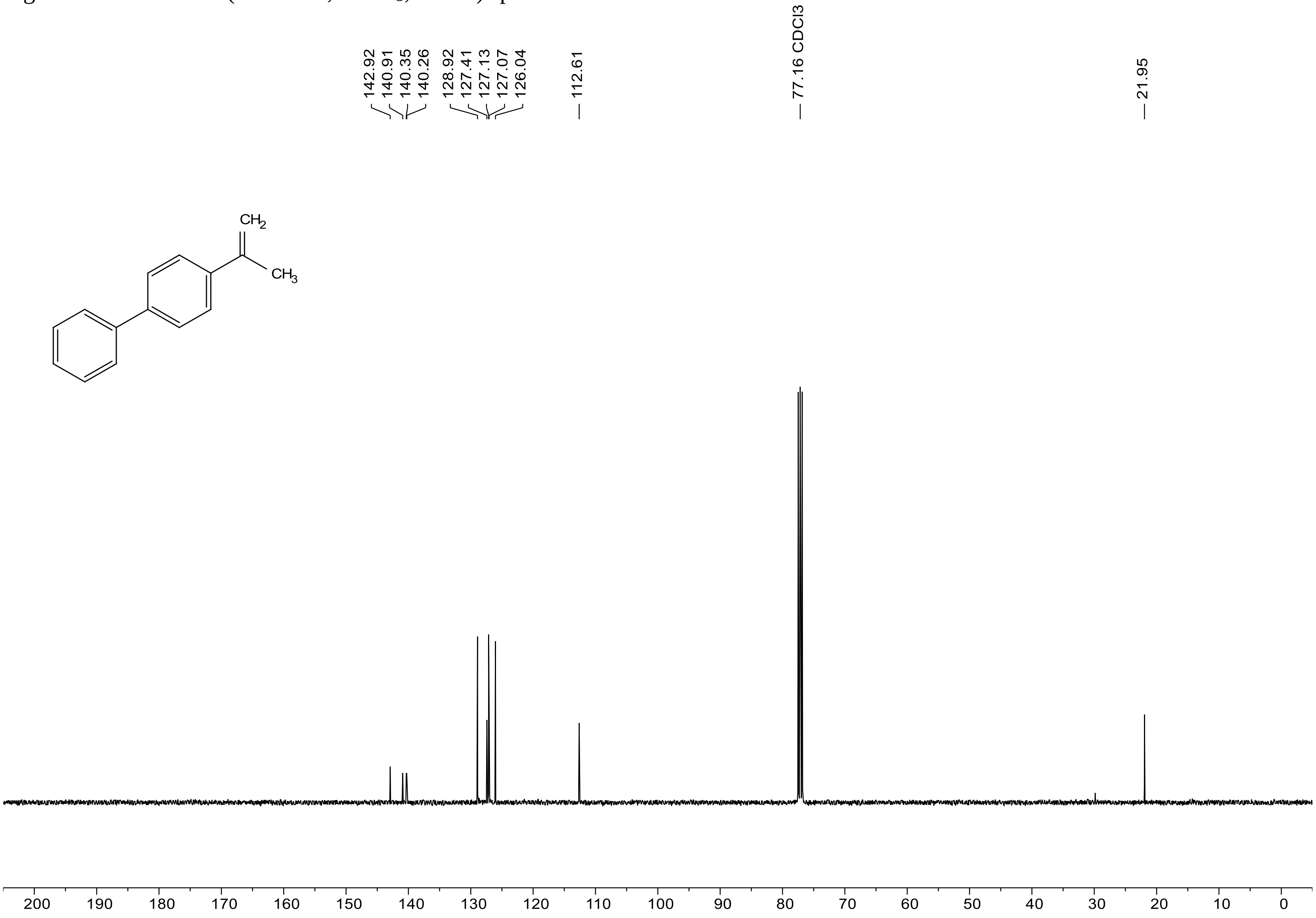

Figure S42: **^{1}H NMR** (400 MHz, $CDCl_3$, 298 K) spectrum of **1w**.

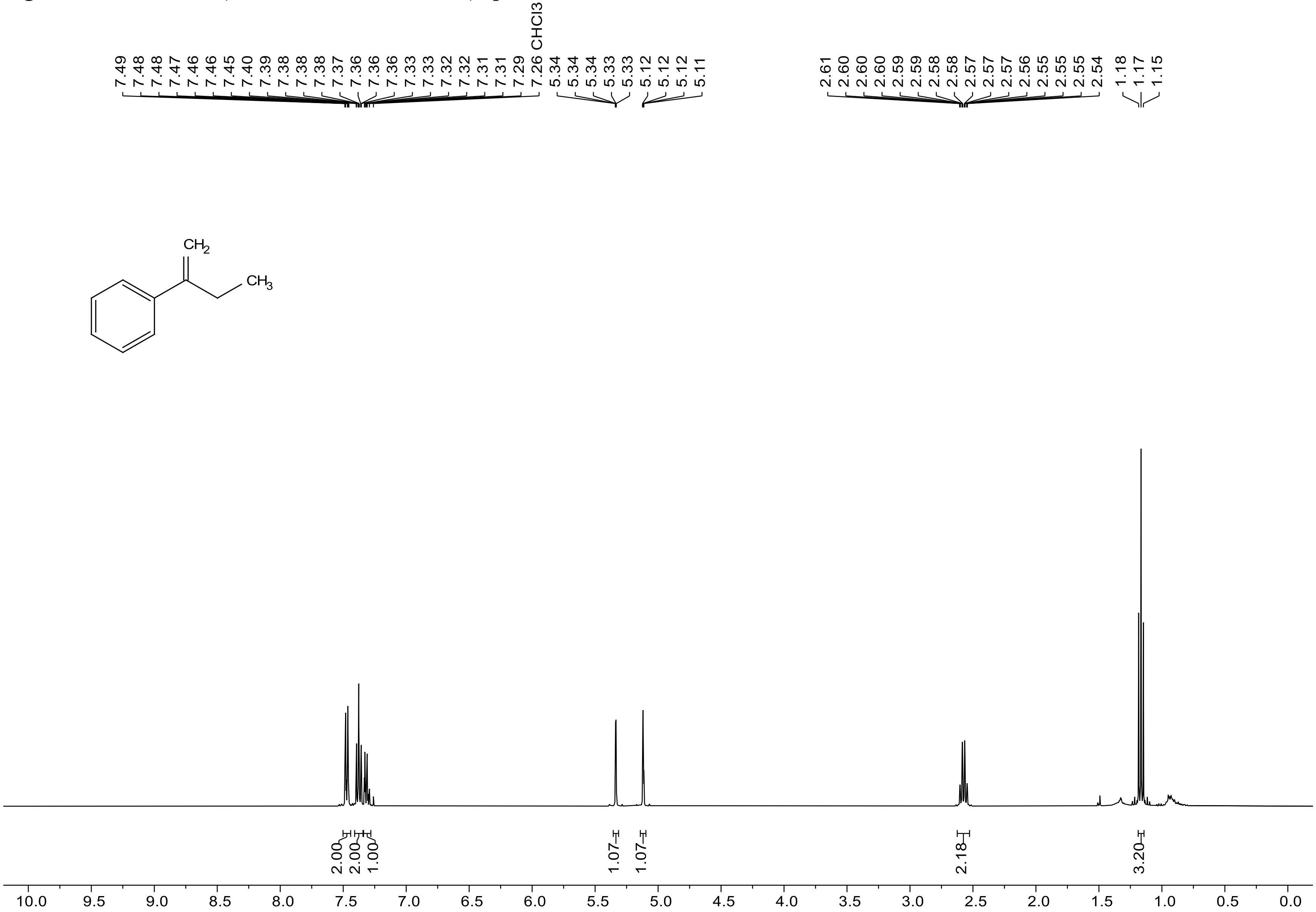

Figure S43: **^{13}C NMR** (101 MHz, $CDCl_3$, 298 K) spectrum of **1w**.

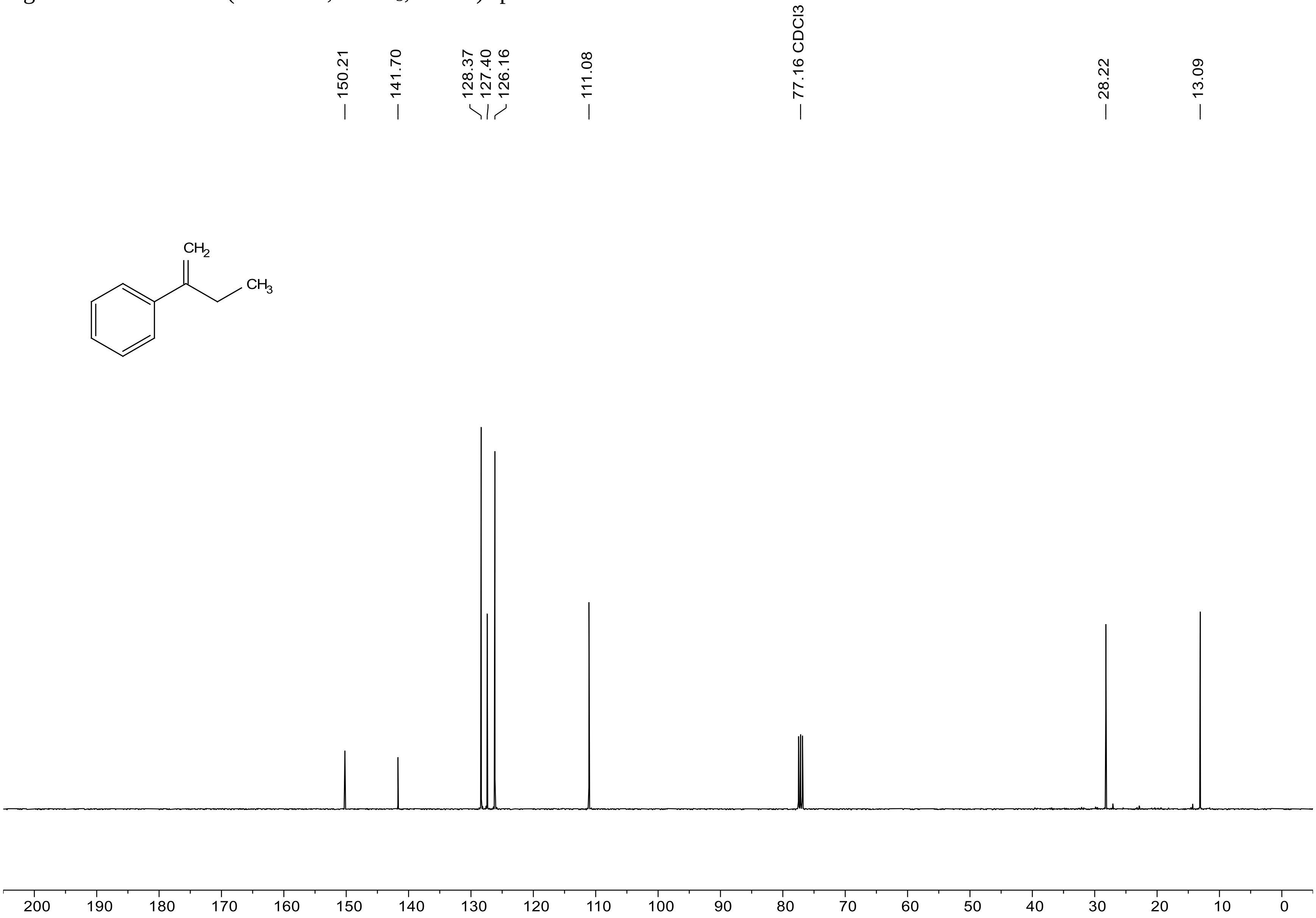

Figure S44: **^{1}H NMR** (400 MHz, $CDCl_3$, 298 K) spectrum of **1x**.

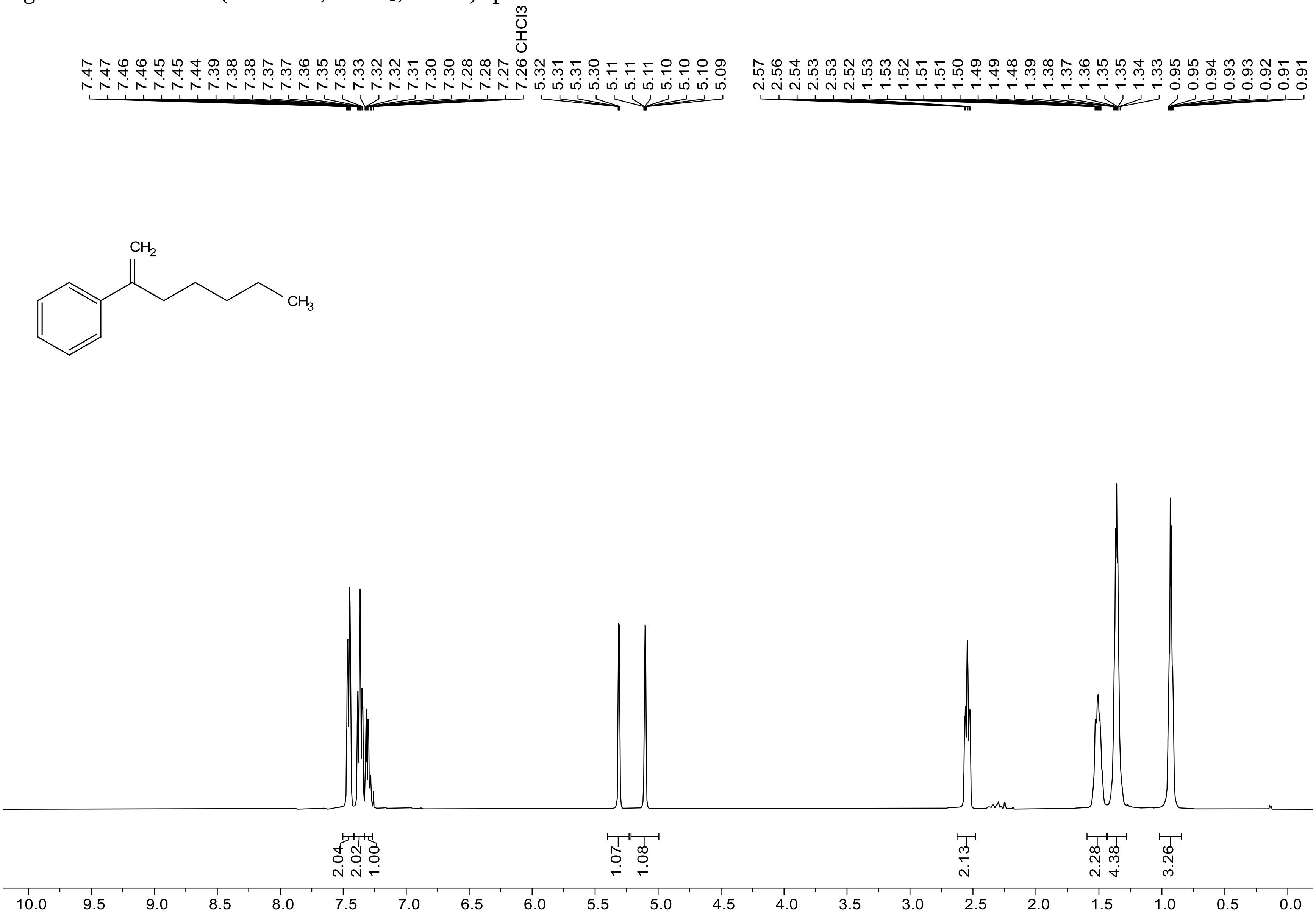

Figure S45: ^{13}**C NMR** (101 MHz, $CDCl_3$, 298 K) spectrum of **1x**.

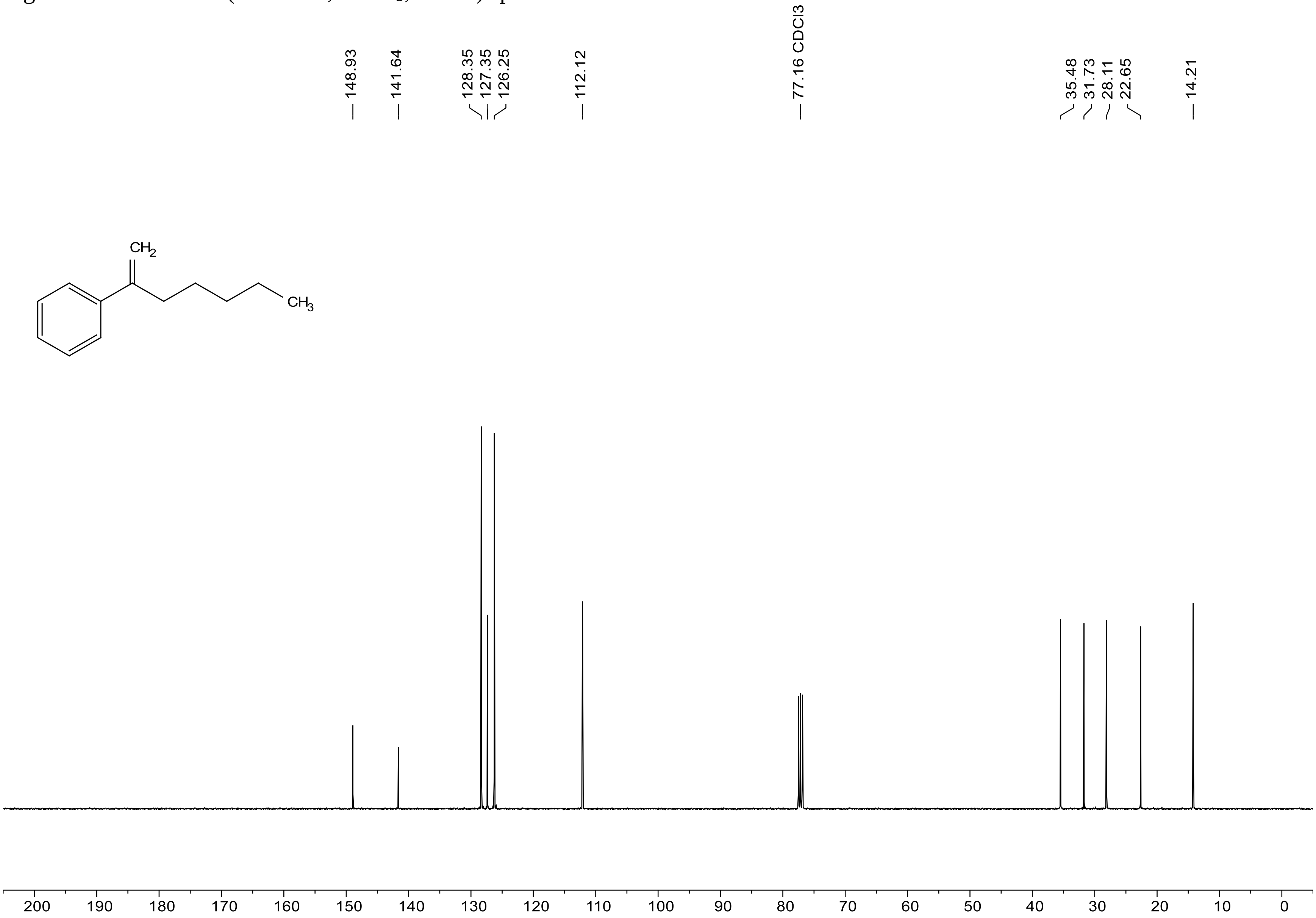

Figure S46: **^{1}H NMR** (400 MHz, $CDCl_3$, 298 K) spectrum of **3aa**.

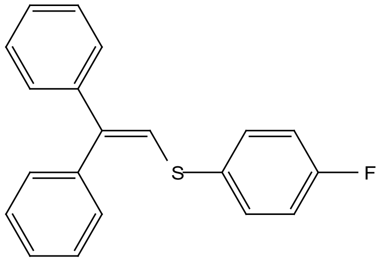

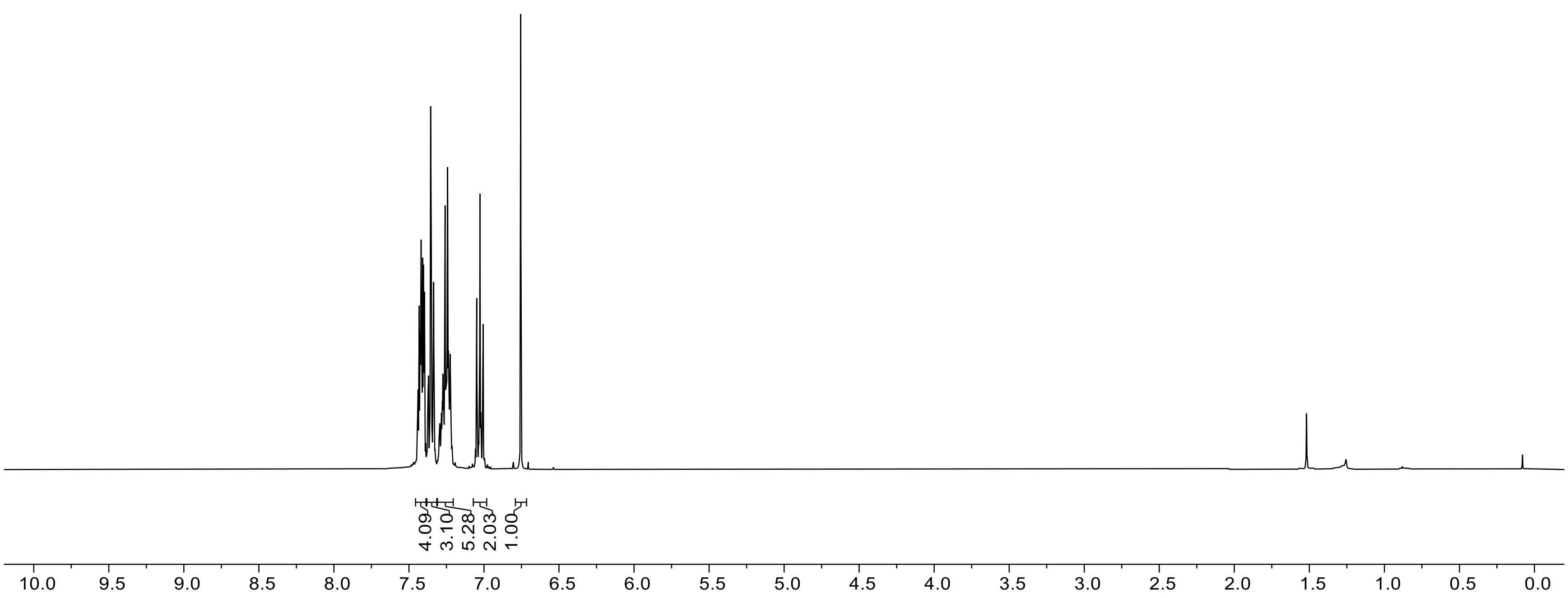

Figure S47: **^{13}C NMR** (101 MHz, $CDCl_3$, 298 K) spectrum of **3aa**.

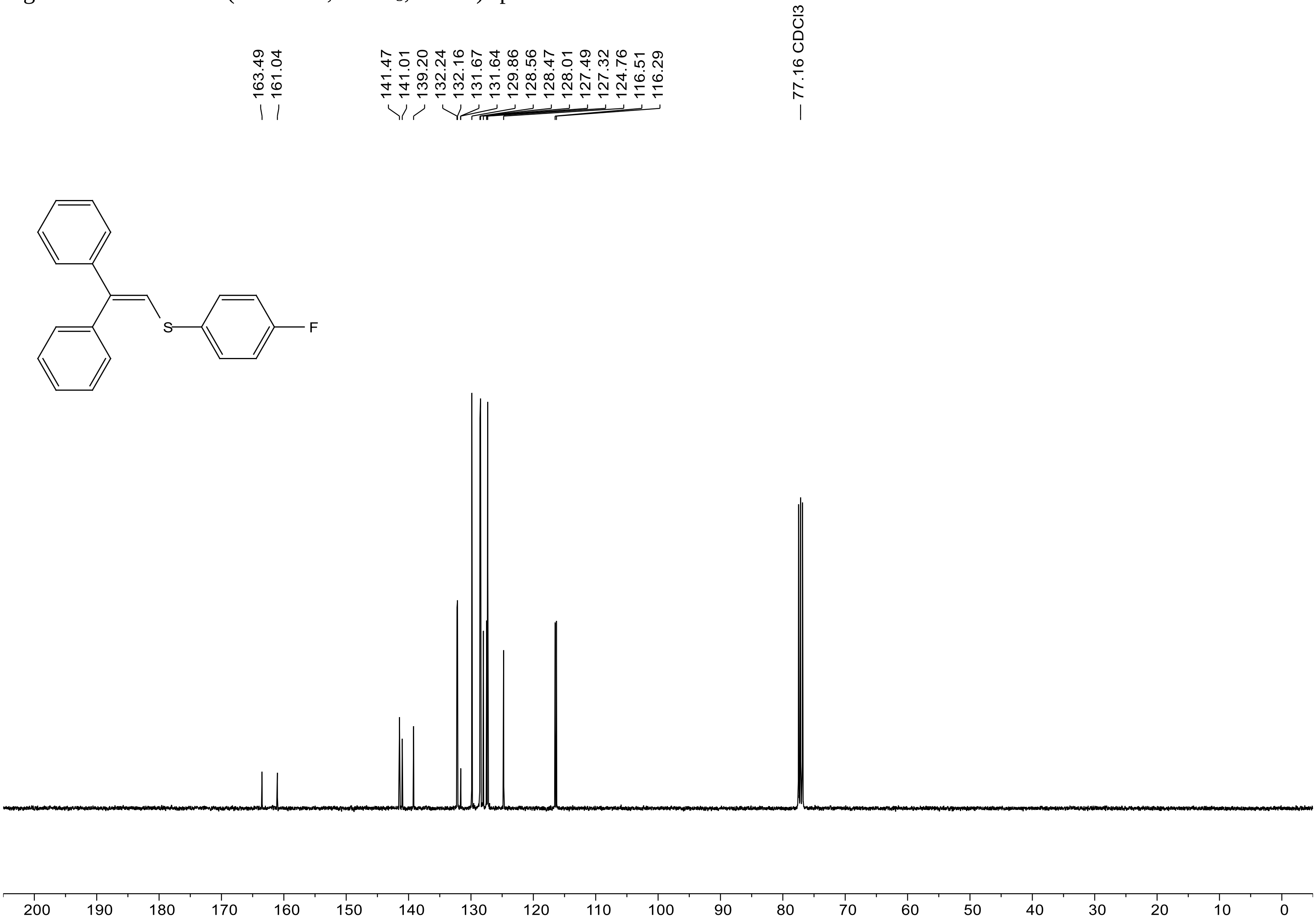

Figure S48: ^{19}**F NMR** (376 MHz, $CDCl_3$, 298 K) spectrum of **3aa**.

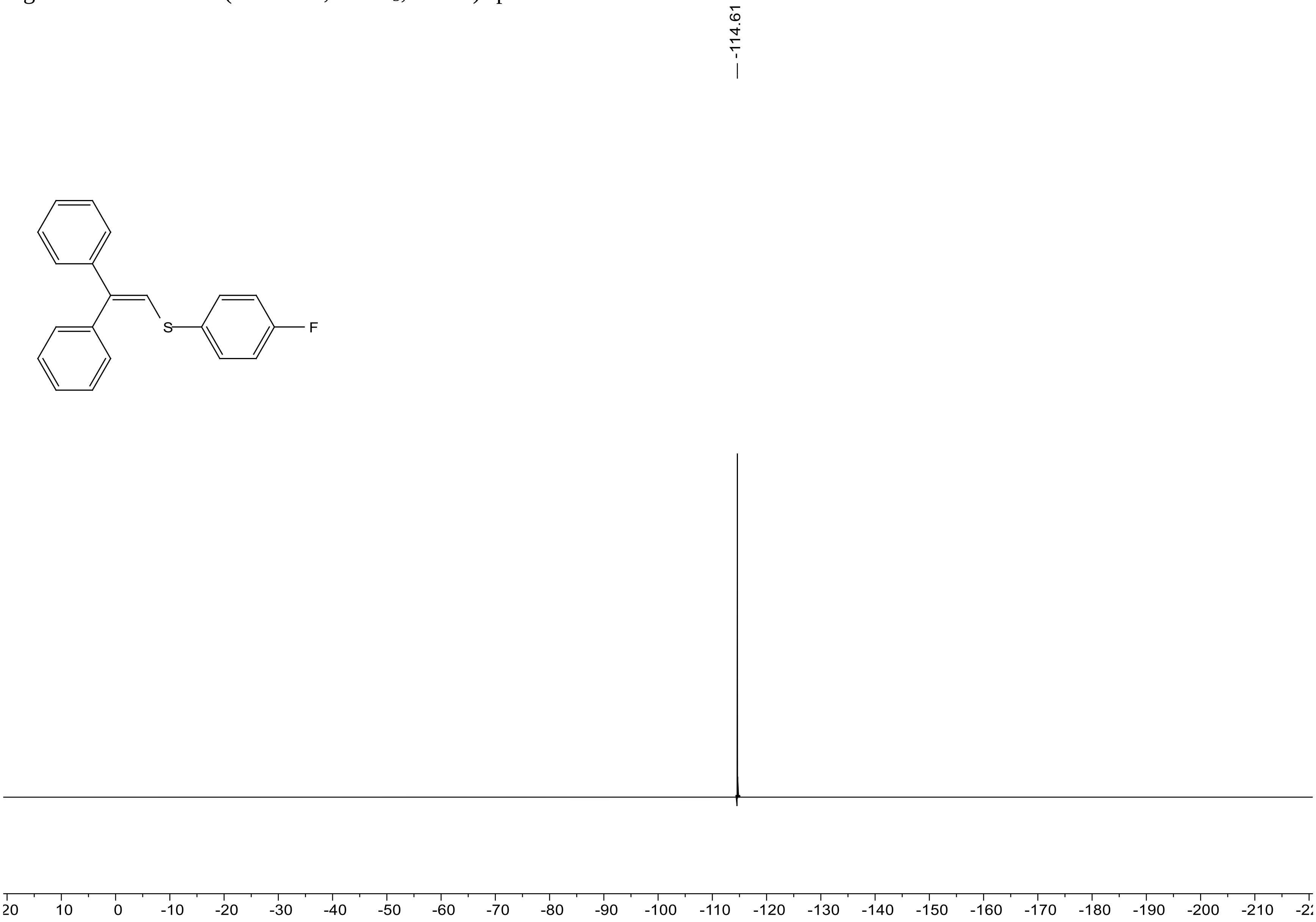

Figure S49: **^{1}H NMR** (400 MHz, $CDCl_3$, 298 K) spectrum of **3ba**.

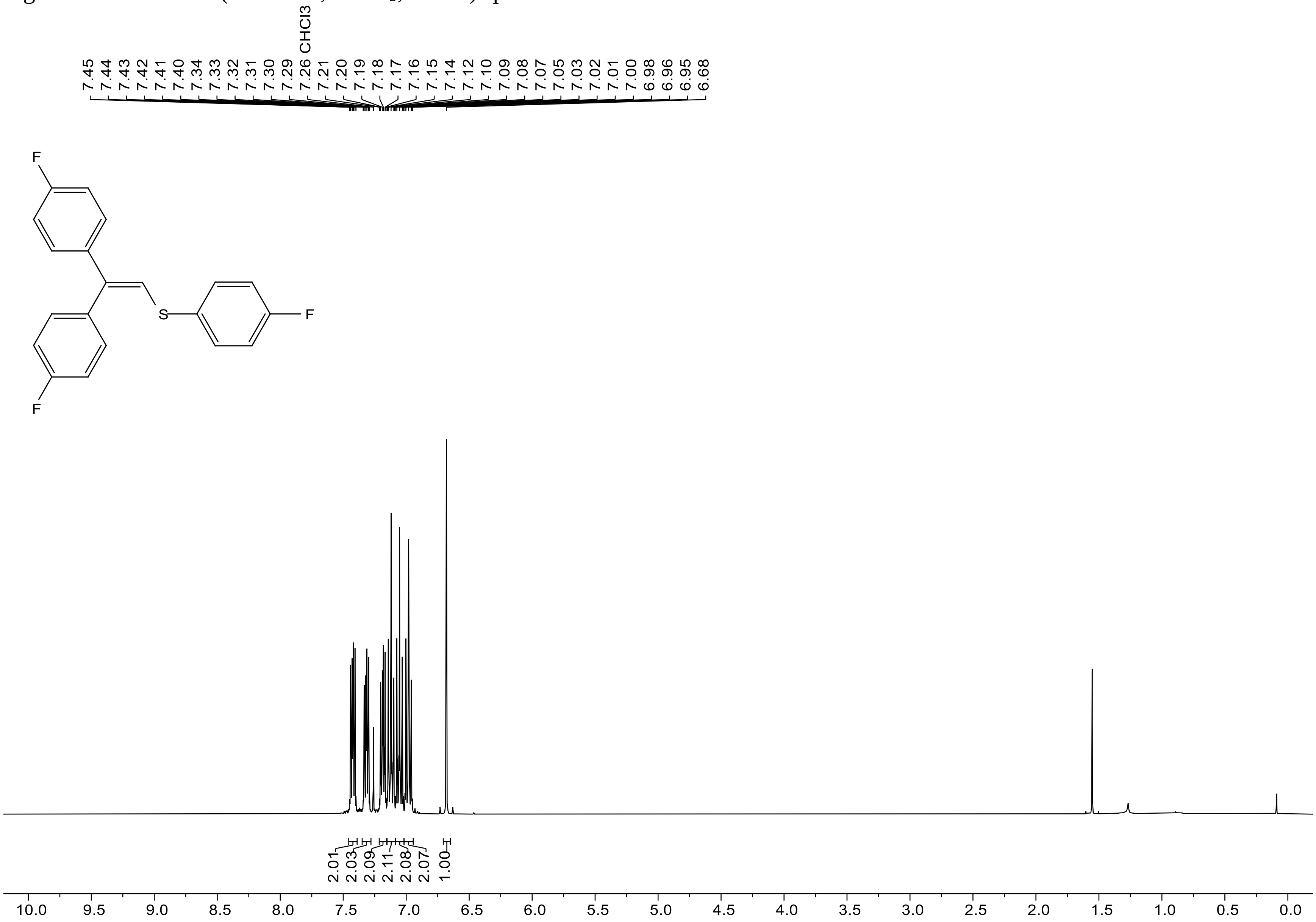

Figure S50: **^{13}C NMR** (101 MHz, $CDCl_3$, 298 K) spectrum of **3ba**.

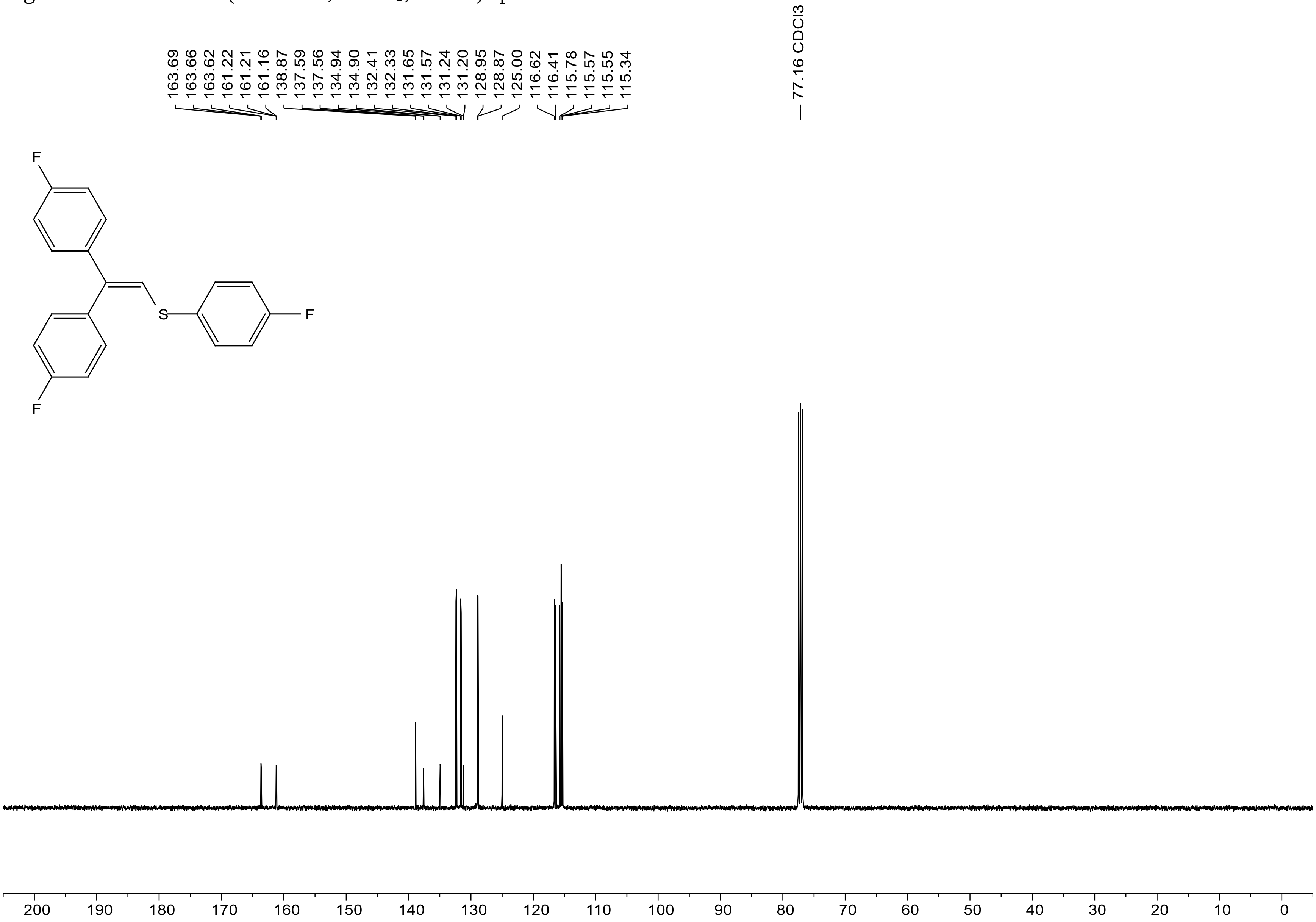

Figure S51: ^{19}**F NMR** (376 MHz, $CDCl_3$, 298 K) spectrum of **3ba**.

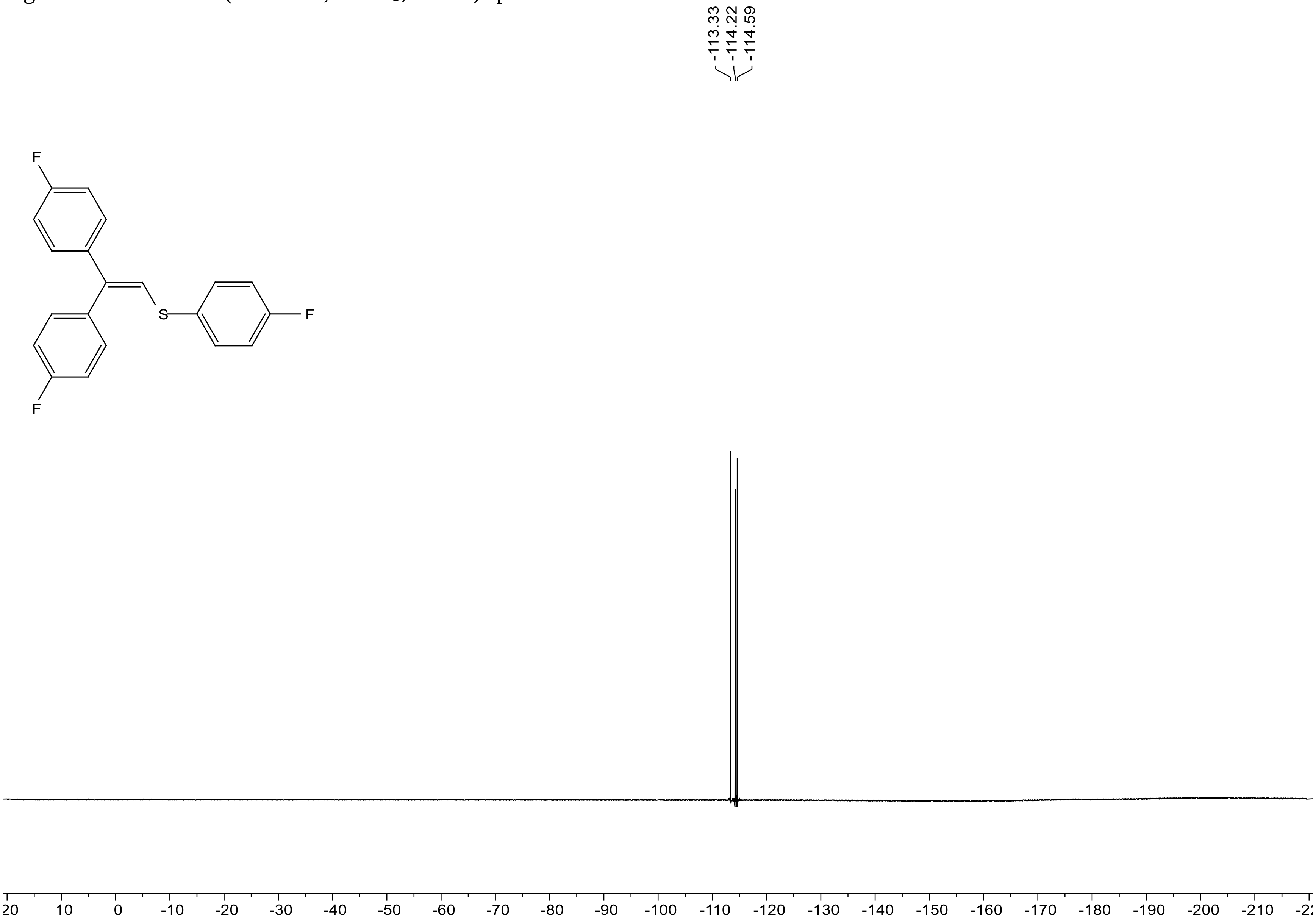

Figure S52: **^{1}H NMR** (400 MHz, $CDCl_3$, 298 K) spectrum of **3ca**.

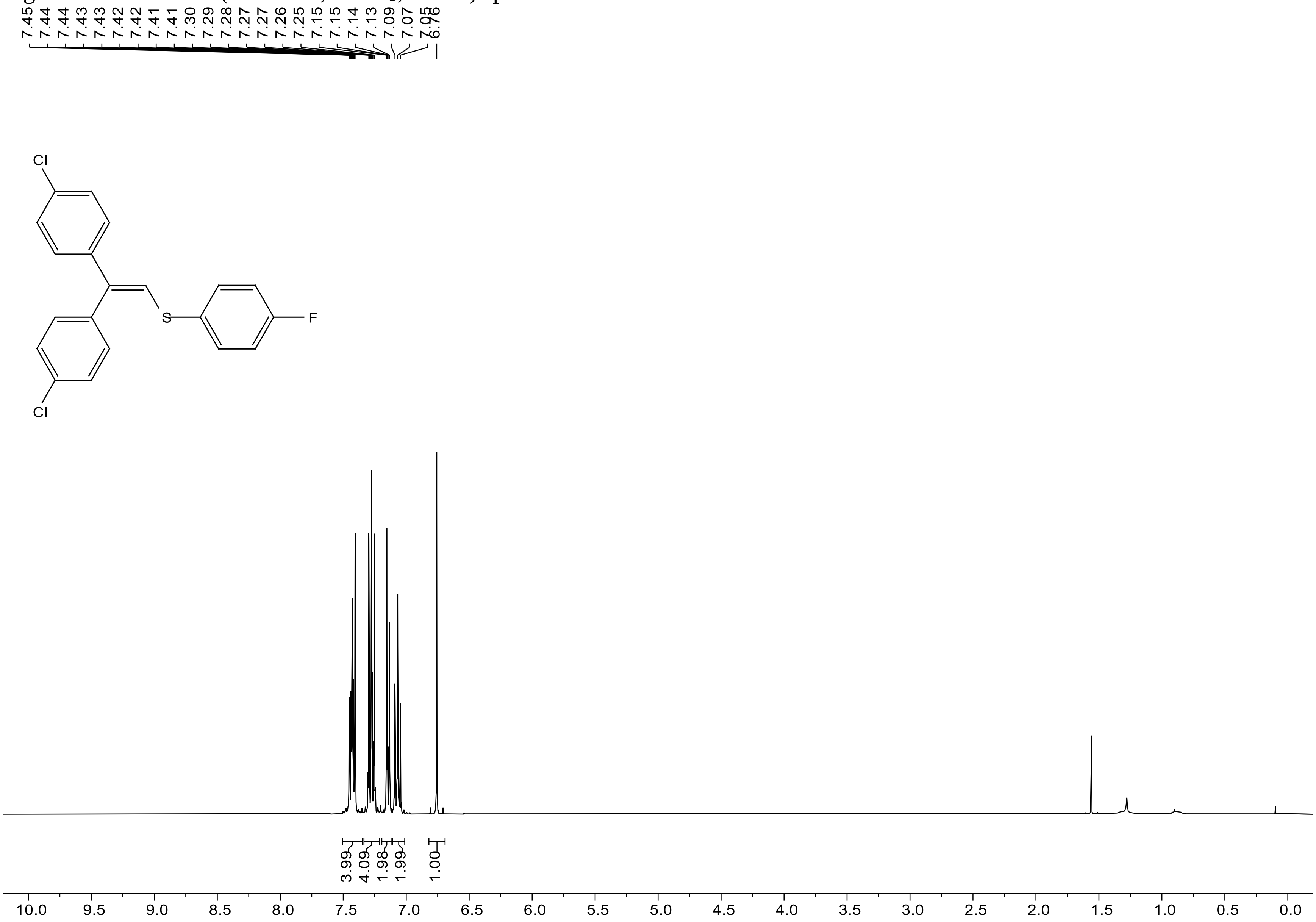

Figure S53: **^{13}C NMR** (101 MHz, $CDCl_3$, 298 K) spectrum of **3ca**.

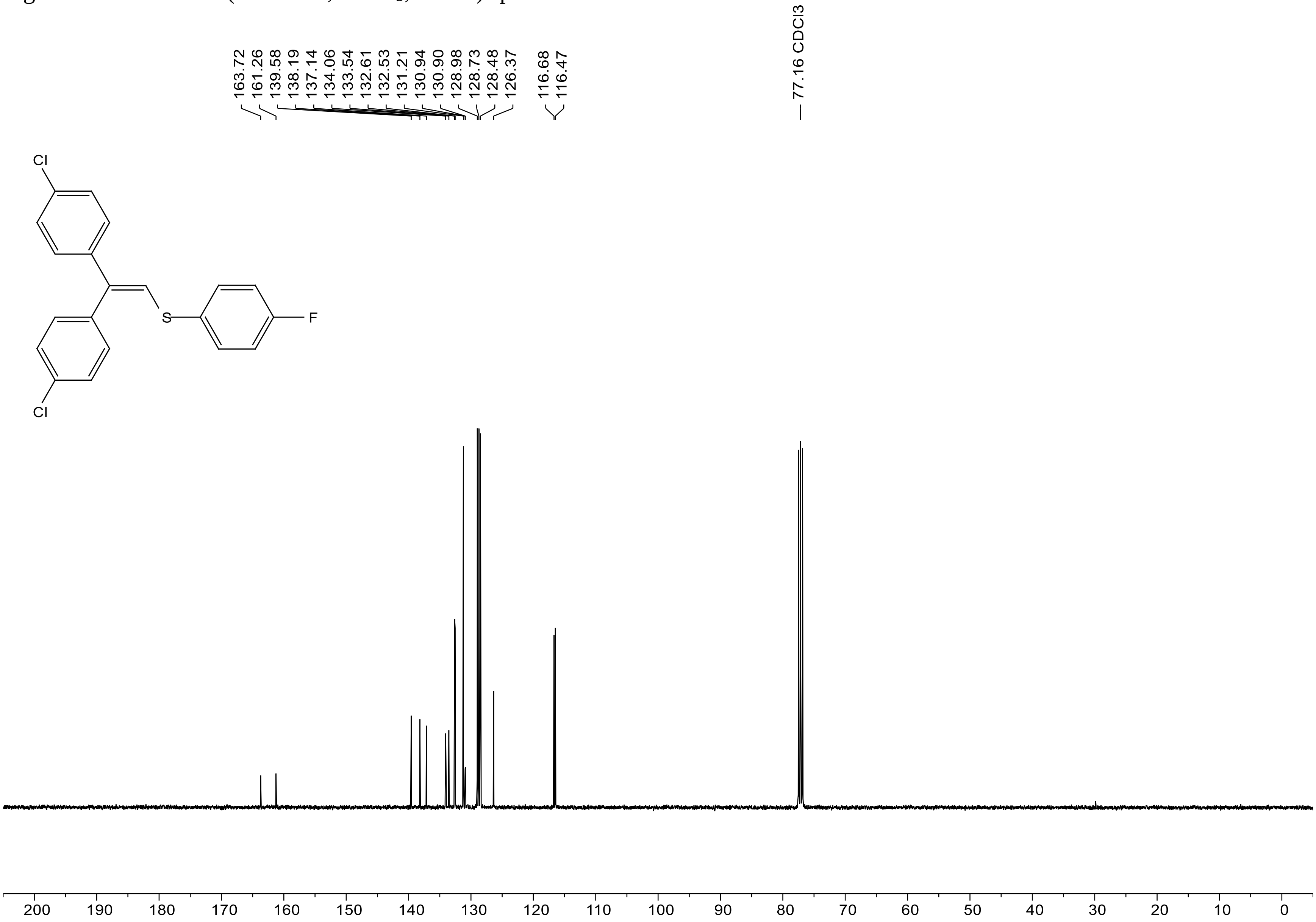

Figure S54: **^{19}F NMR** (376 MHz, $CDCl_3$, 298 K) spectrum of **3ca**.

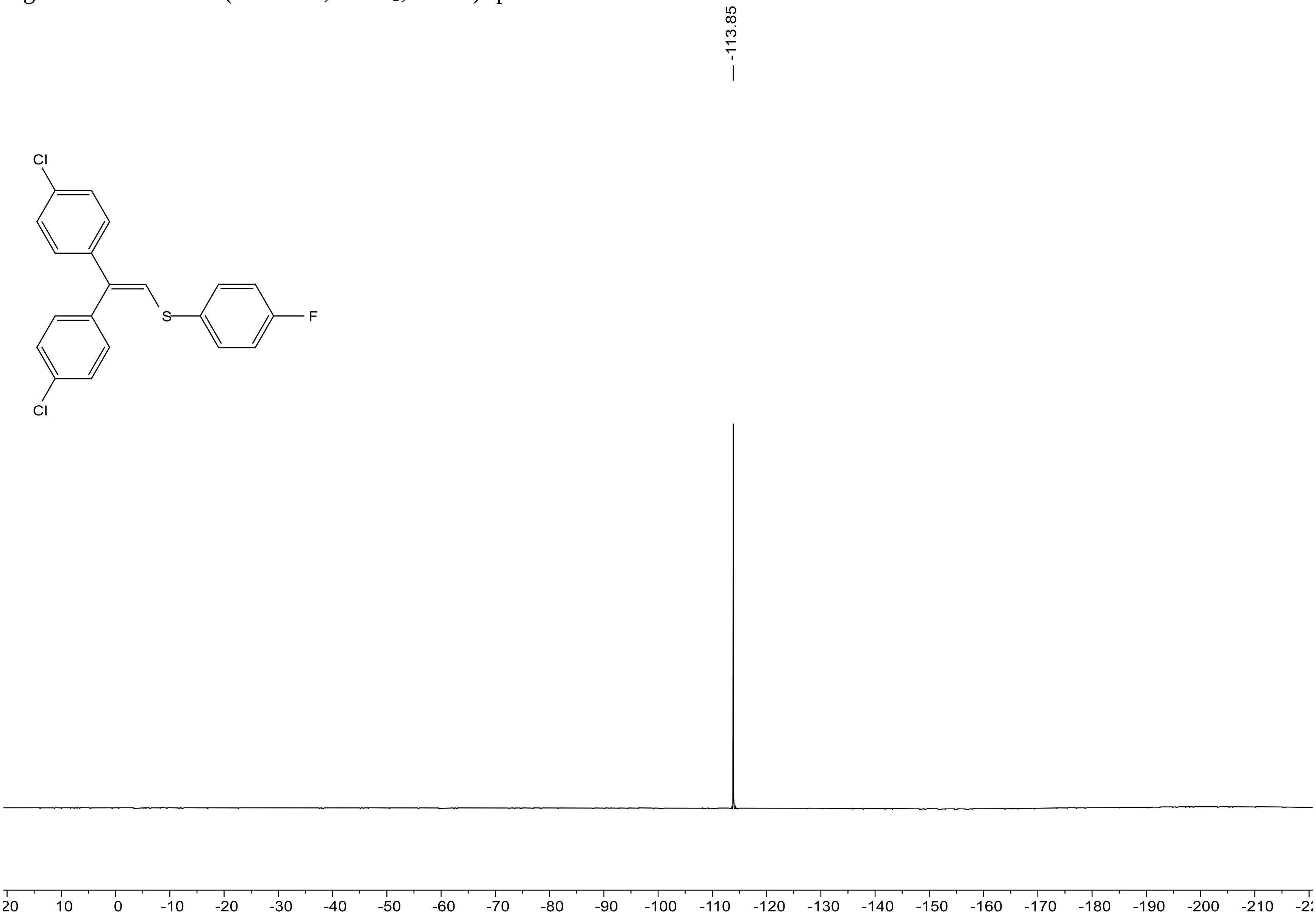

Figure S55: **^{1}H NMR** (400 MHz, $CDCl_3$, 298 K) spectrum of **3da**.

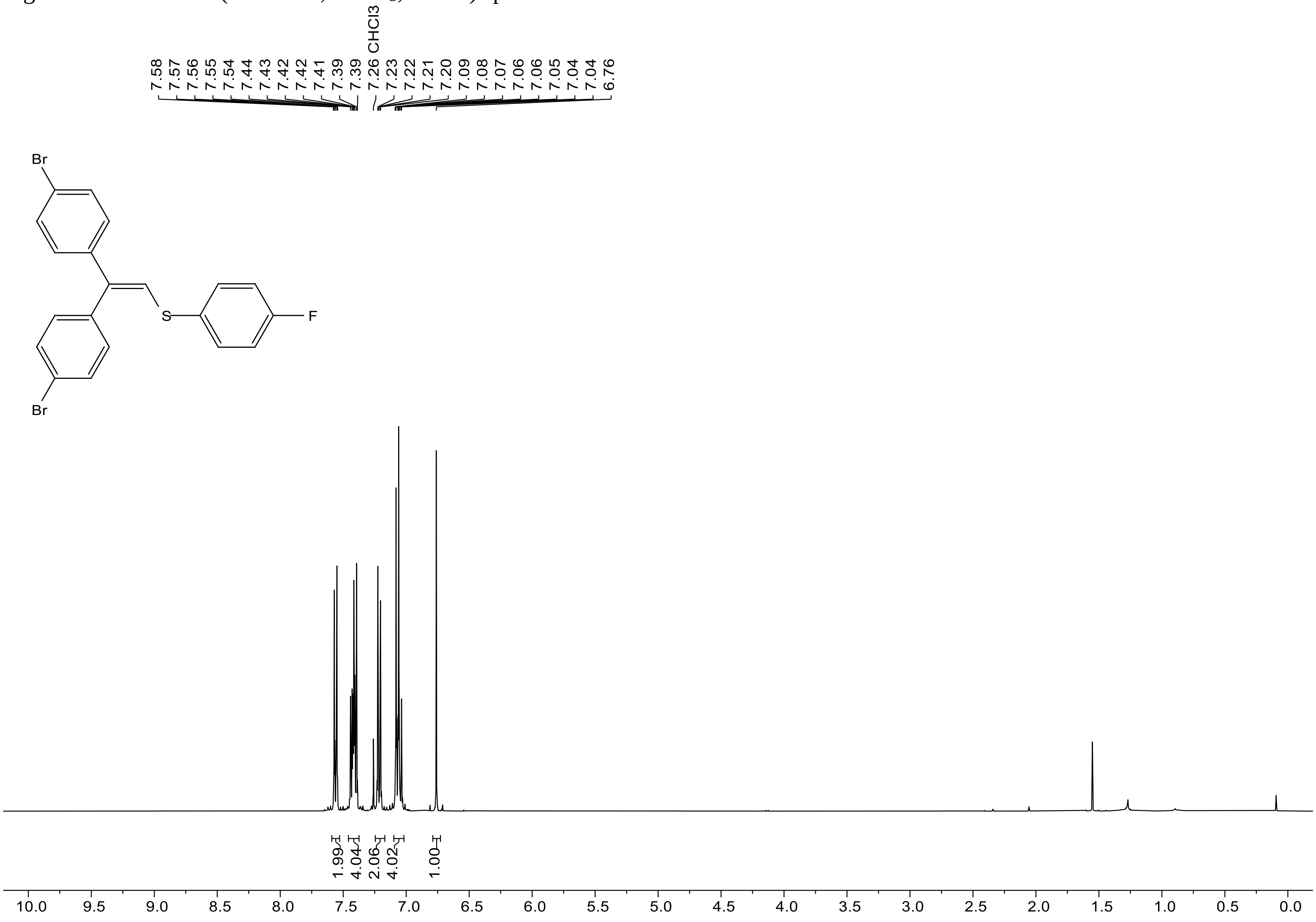

Figure S56: ^{13}**C NMR** (101 MHz, $CDCl_3$, 298 K) spectrum of **3da**.

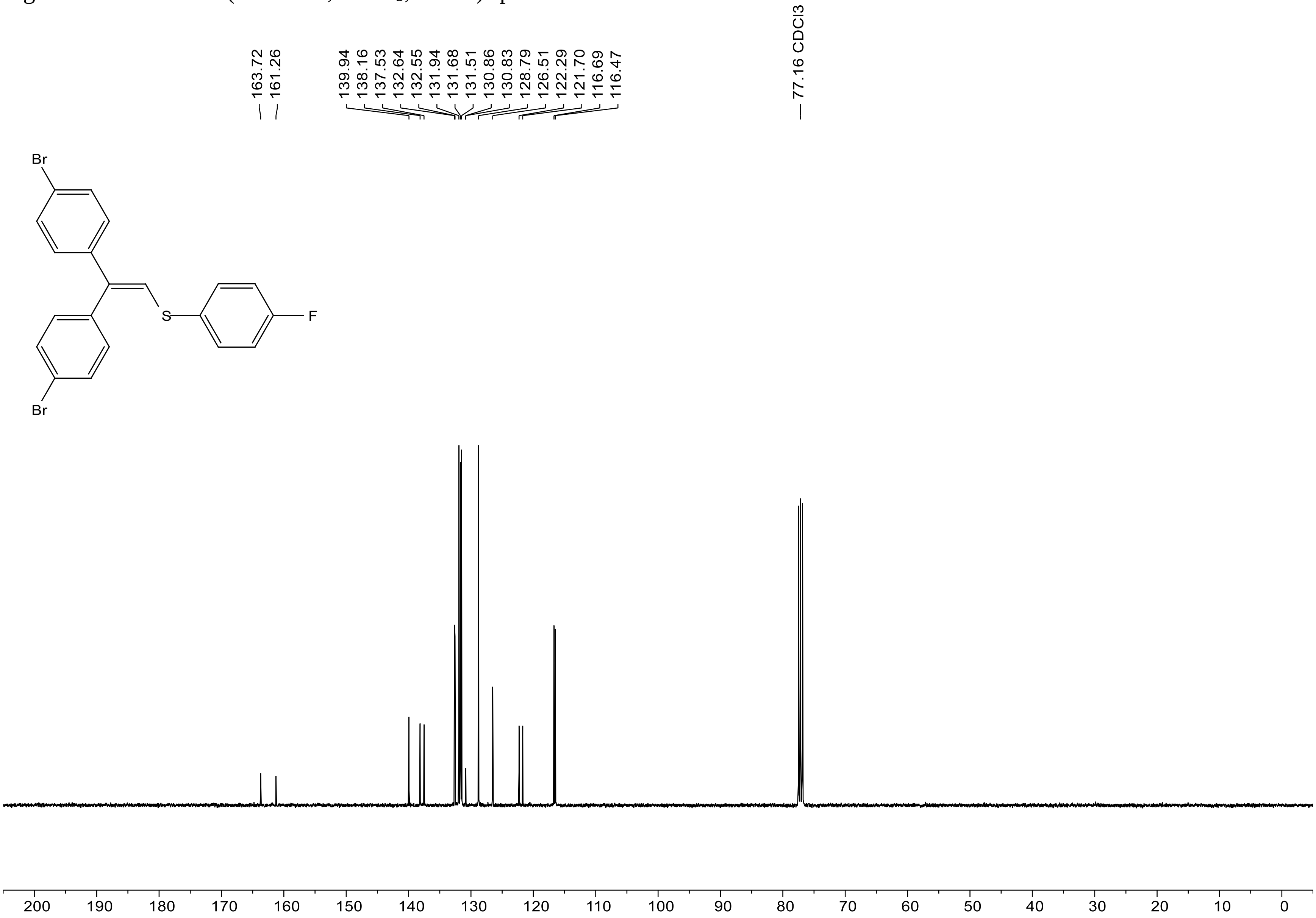

Figure S57: ^{19}**F NMR** (376 MHz, $CDCl_3$, 298 K) spectrum of **3da**.

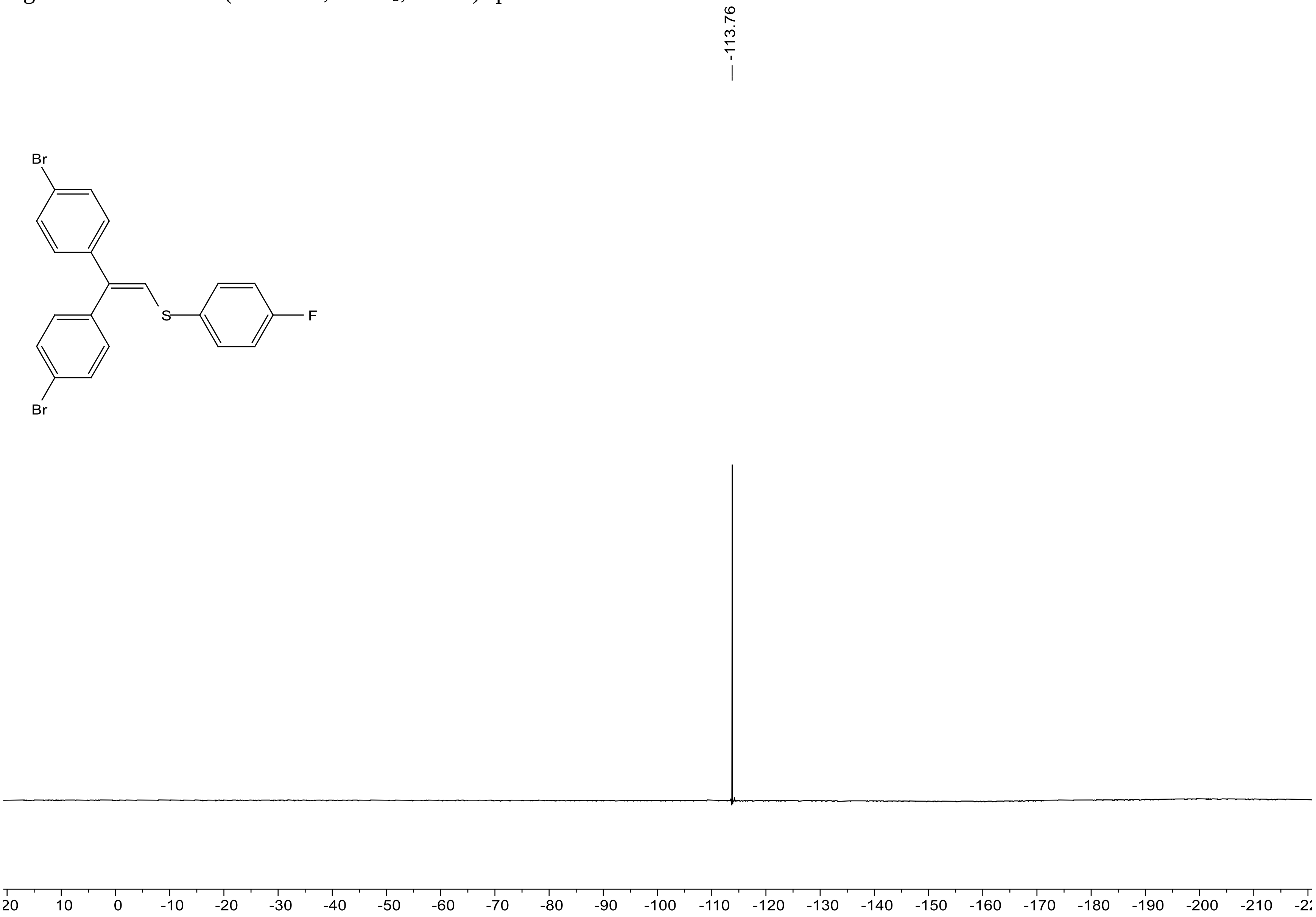

Figure S58: **^{1}H NMR** (400 MHz, $CDCl_3$, 298 K) spectrum of **3ea**.

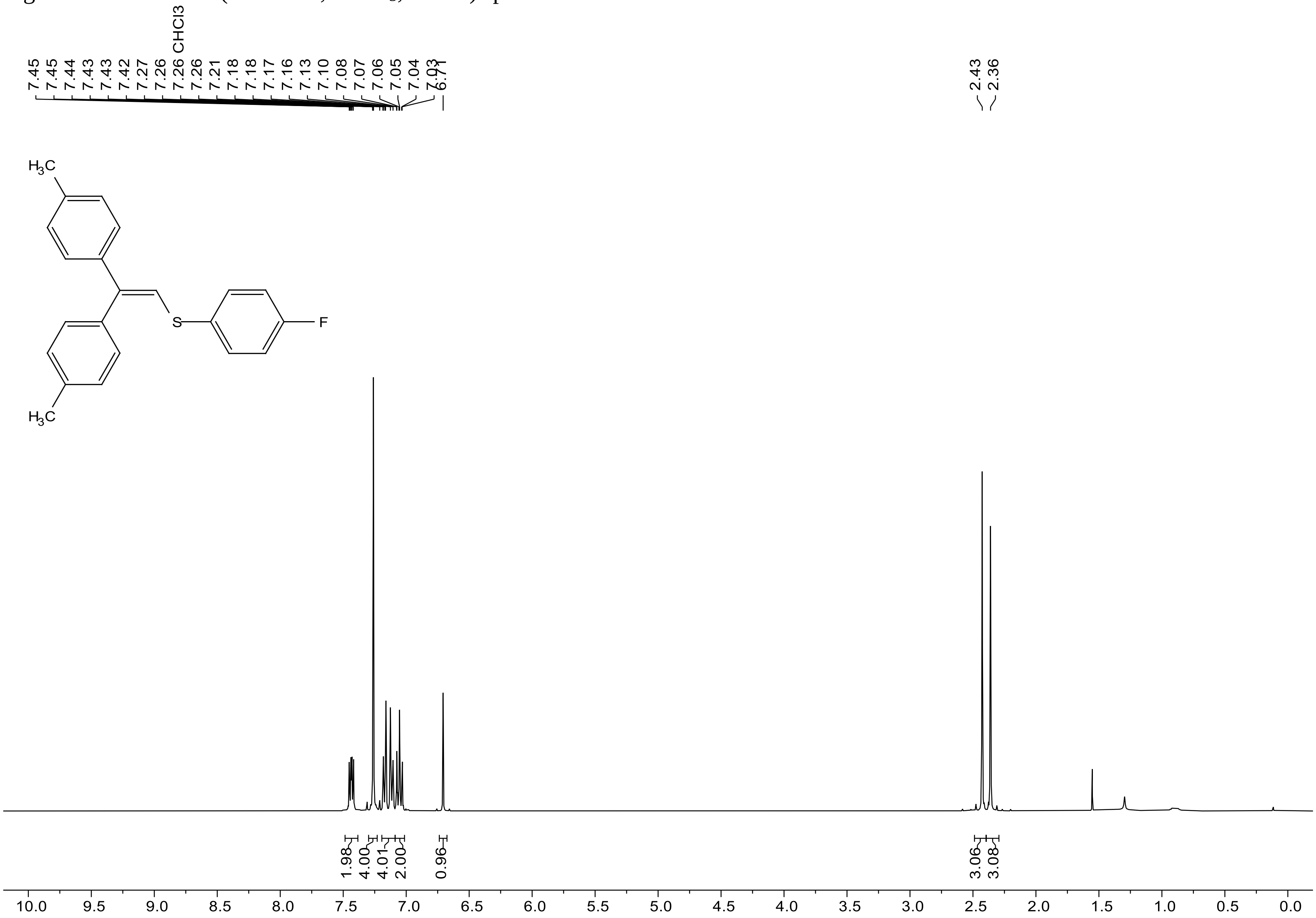

Figure S59: **^{13}C NMR** (101 MHz, $CDCl_3$, 298 K) spectrum of **3ea**.

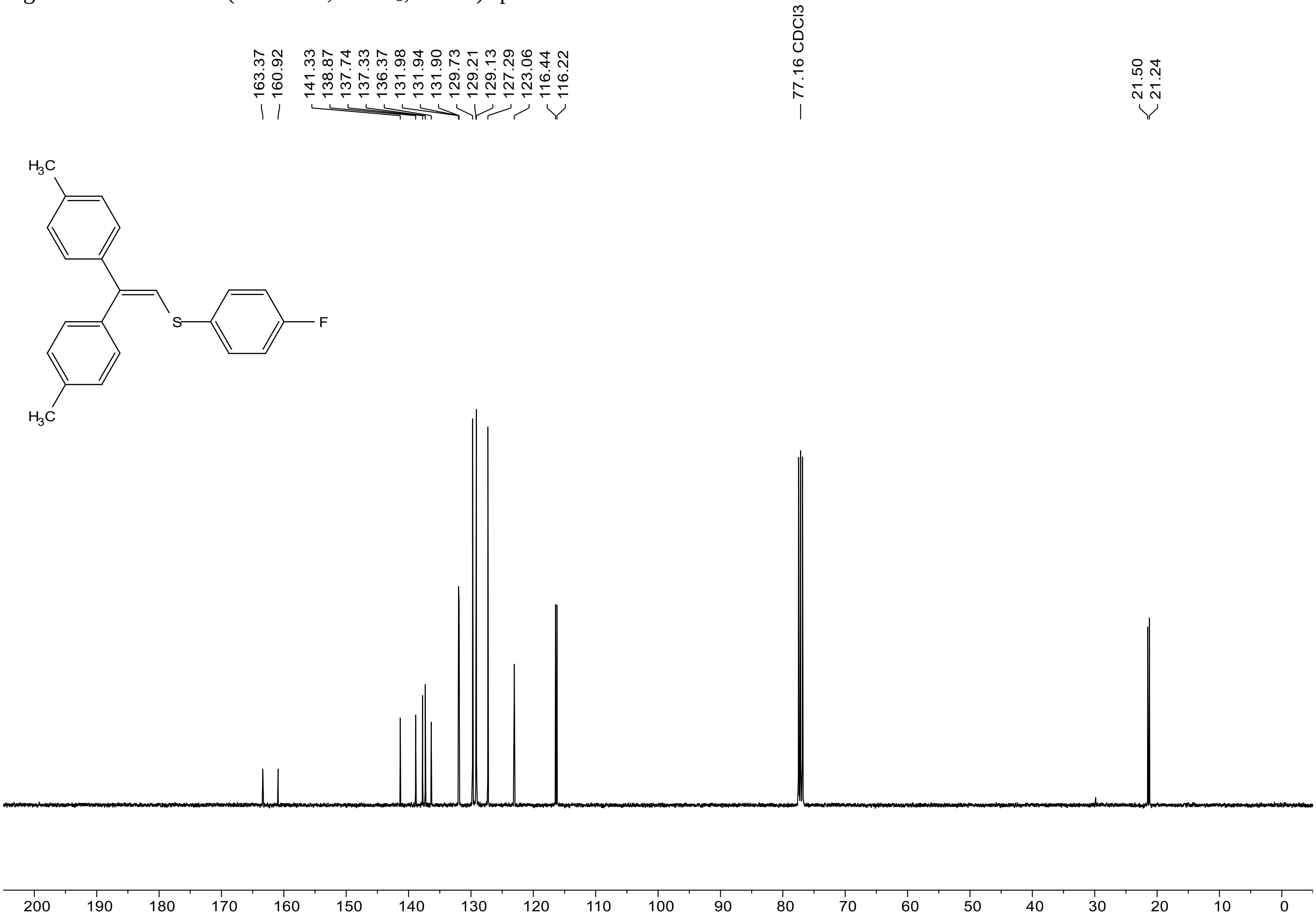

Figure S60: **^{19}F NMR** (376 MHz, $CDCl_3$, 298 K) spectrum of **3ea**.

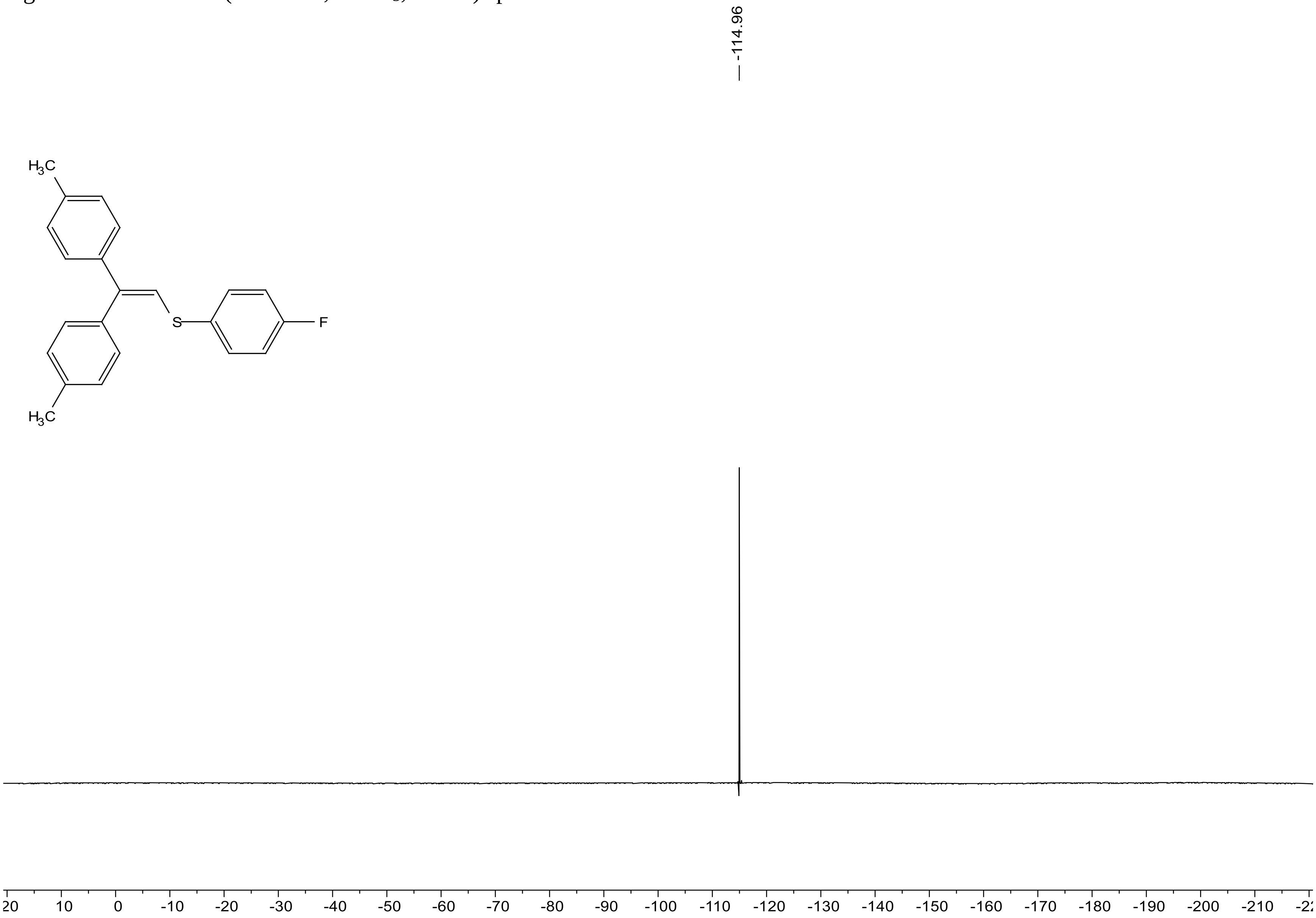

Figure S61: ^{1}**H NMR** (400 MHz, $CDCl_3$, 298 K) spectrum of **3fa**.

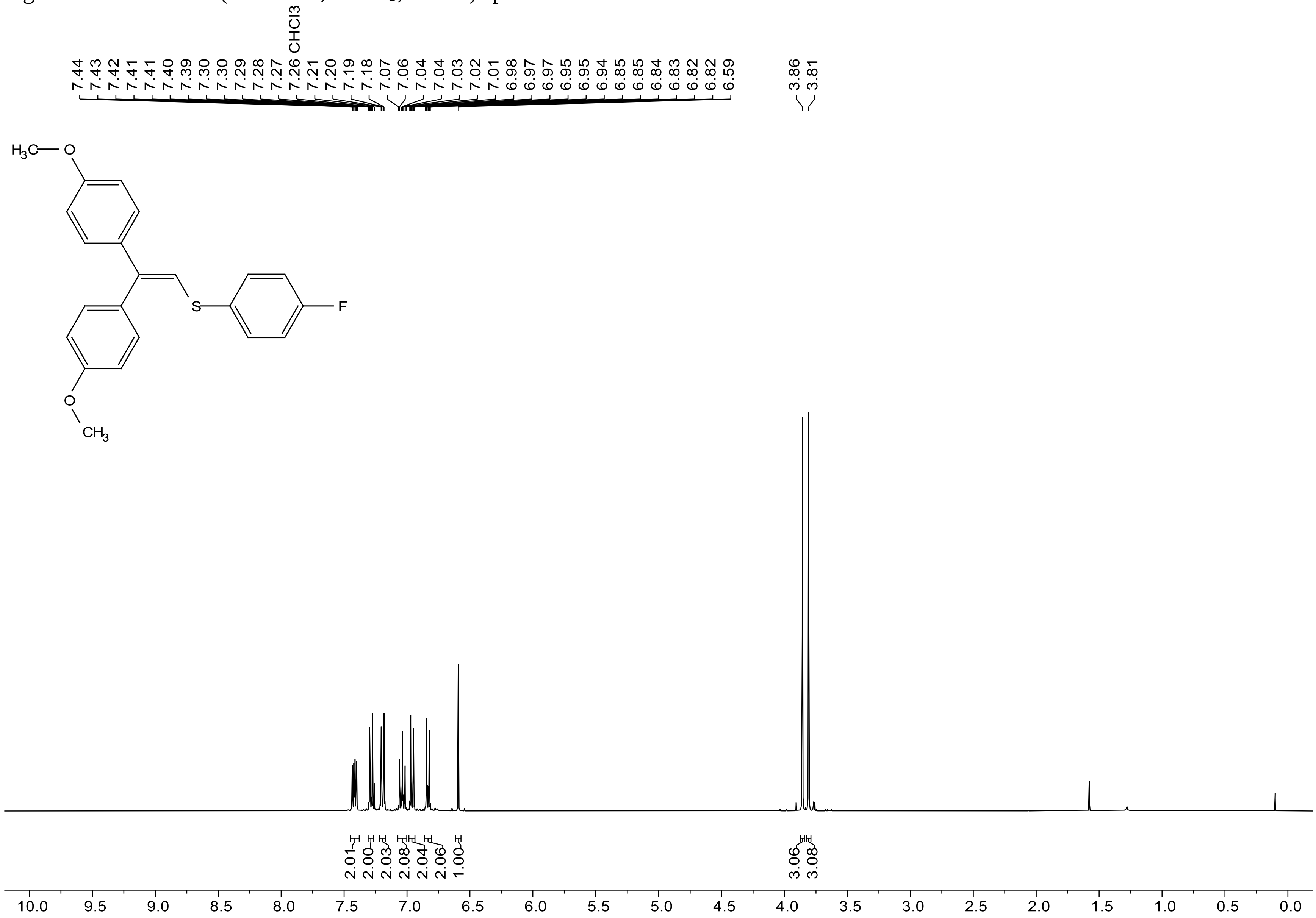

Figure S62: **^{13}C NMR** (101 MHz, $CDCl_3$, 298 K) spectrum of **3fa**.

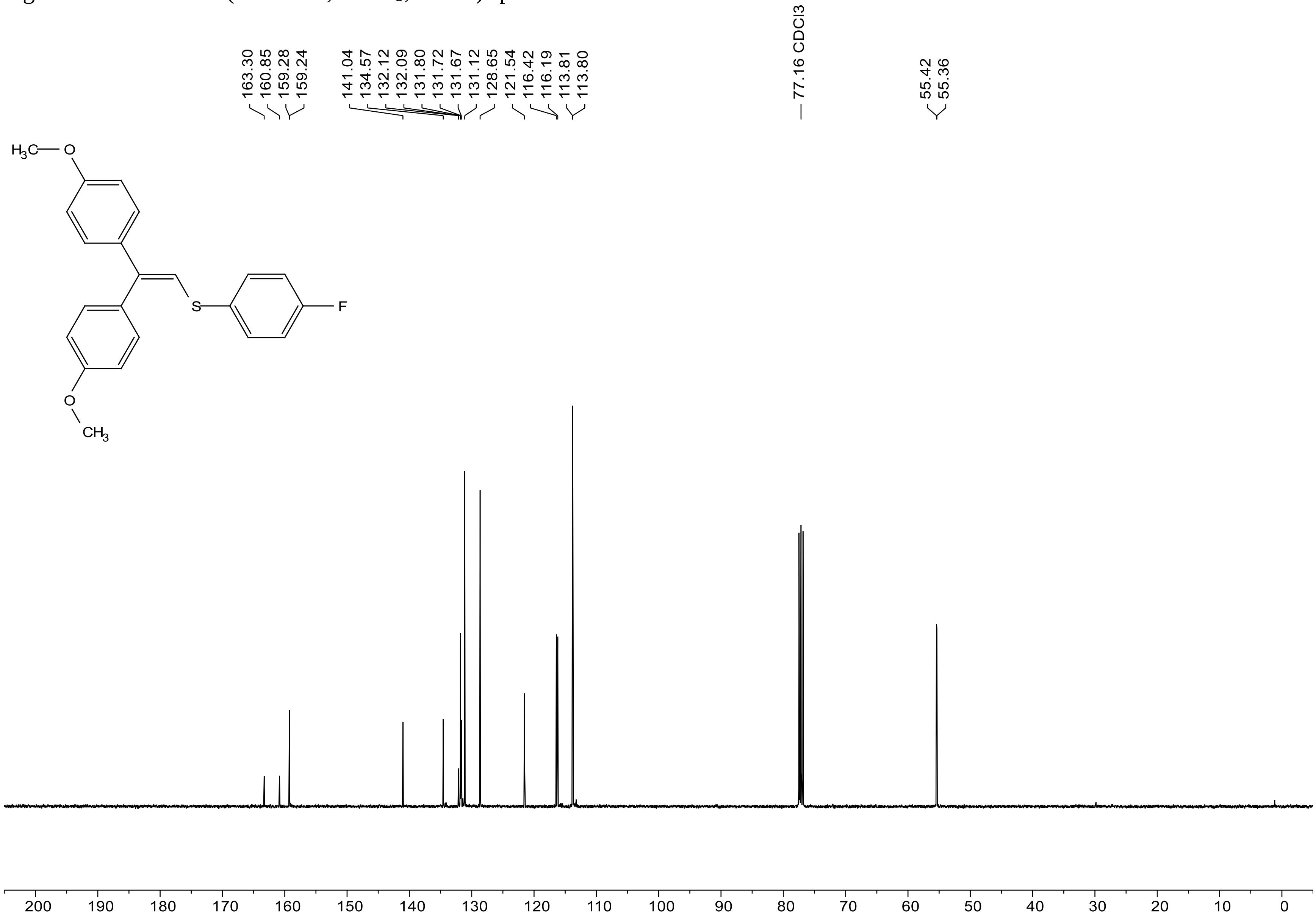

Figure S63: **^{19}F NMR** (376 MHz, $CDCl_3$, 298 K) spectrum of **3fa**.

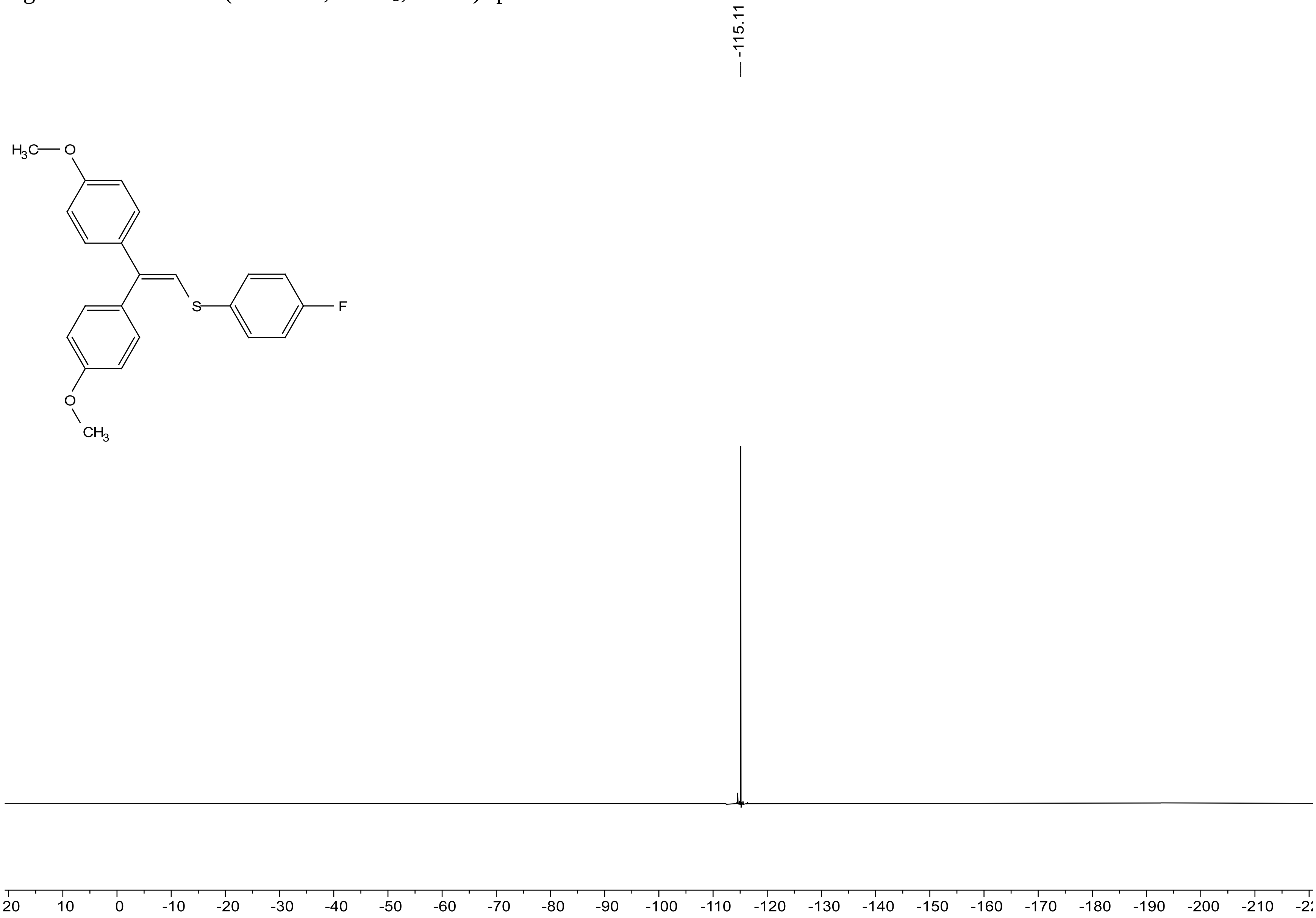

Figure S64: **^{1}H NMR** (400 MHz, $CDCl_3$, 298 K) spectrum of **3ga**.

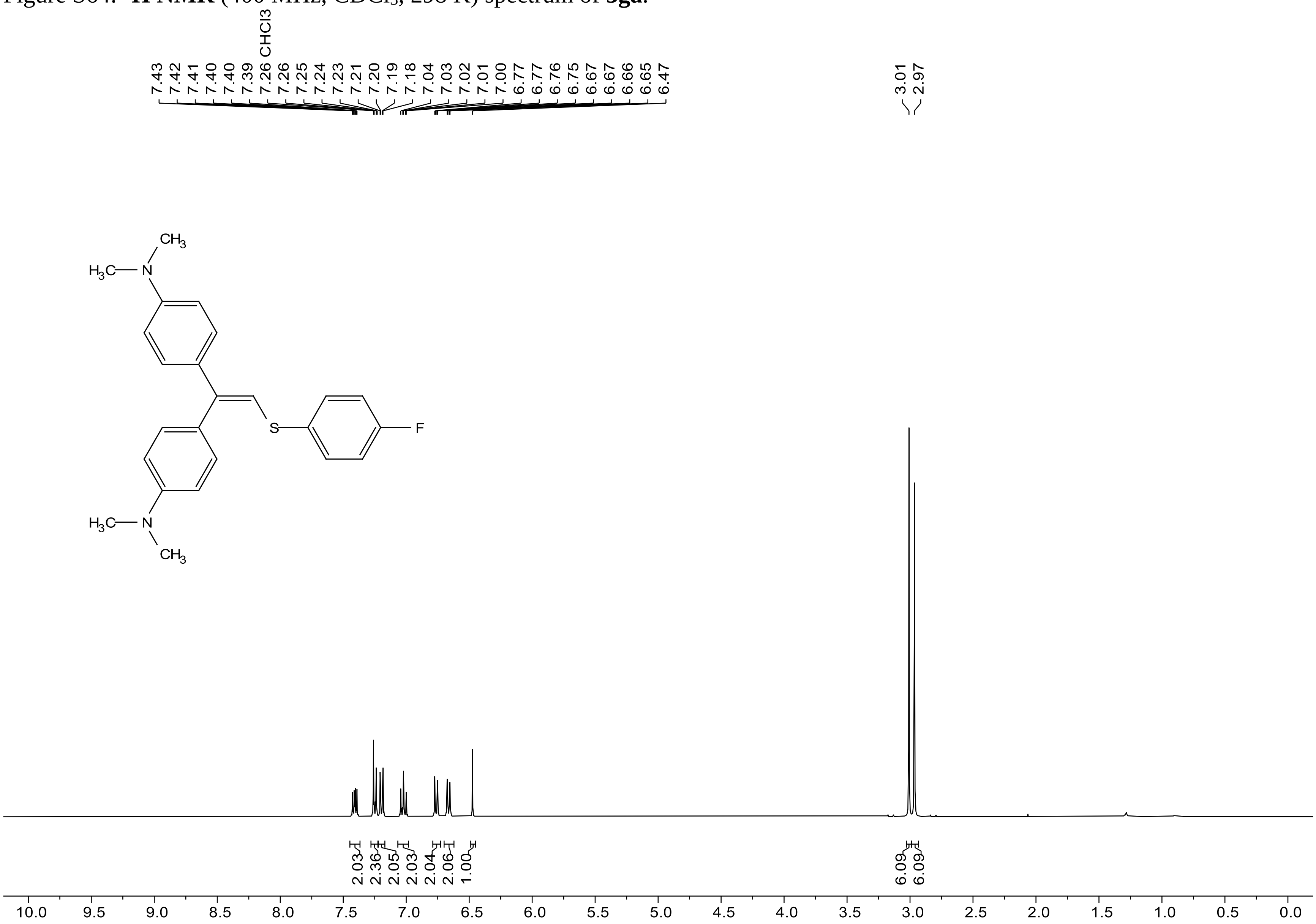

Figure S65: **^{13}C NMR** (101 MHz, $CDCl_3$, 298 K) spectrum of **3ga**.

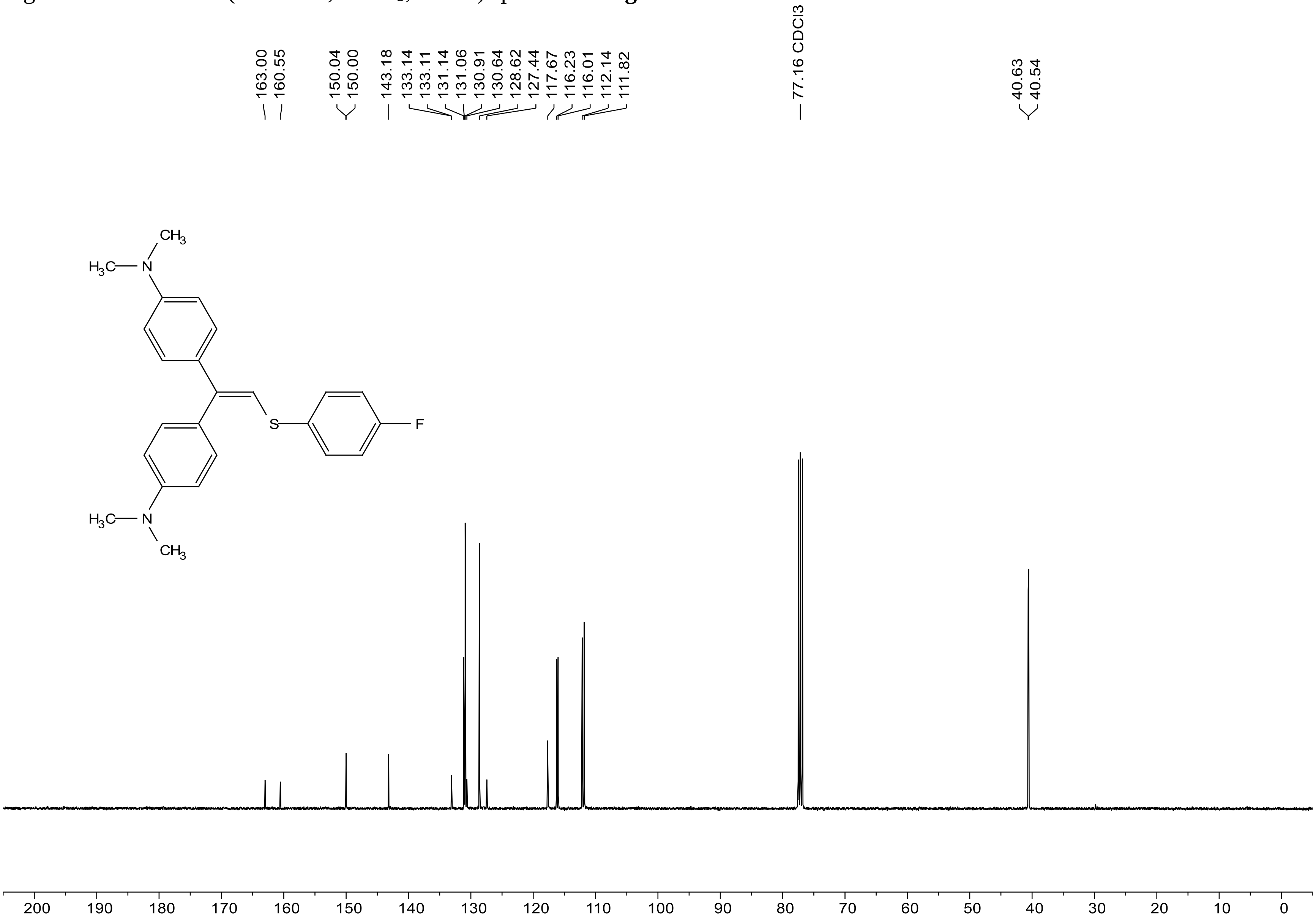

Figure S66: **^{19}F NMR** (376 MHz, $CDCl_3$, 298 K) spectrum of **3ga**.

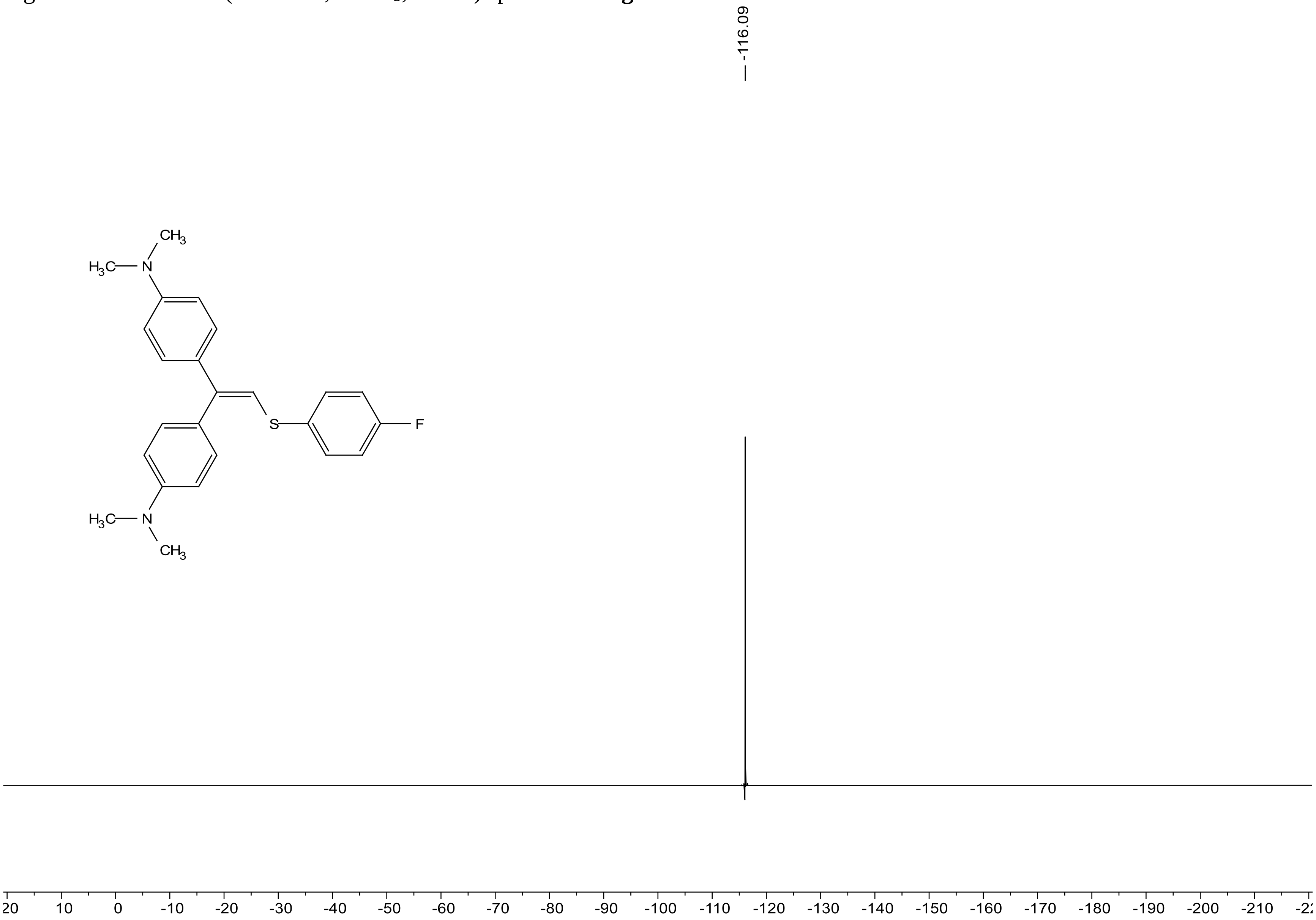

Figure S67: **^{1}H NMR** (400 MHz, $CDCl_3$, 298 K) spectrum of **3ha**.

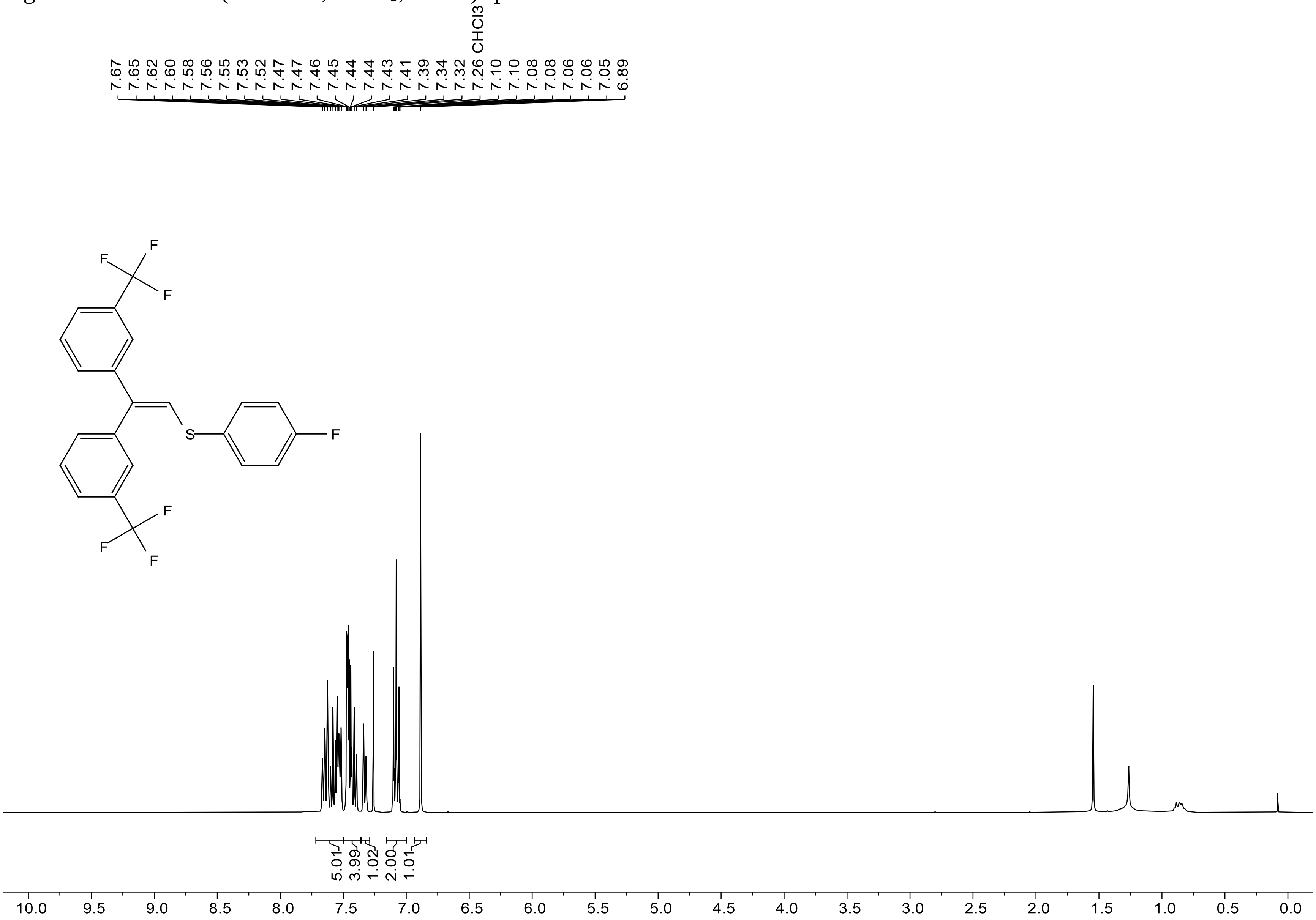

Figure S68: **^{13}C NMR** (101 MHz, $CDCl_3$, 298 K) spectrum of **3ha**.

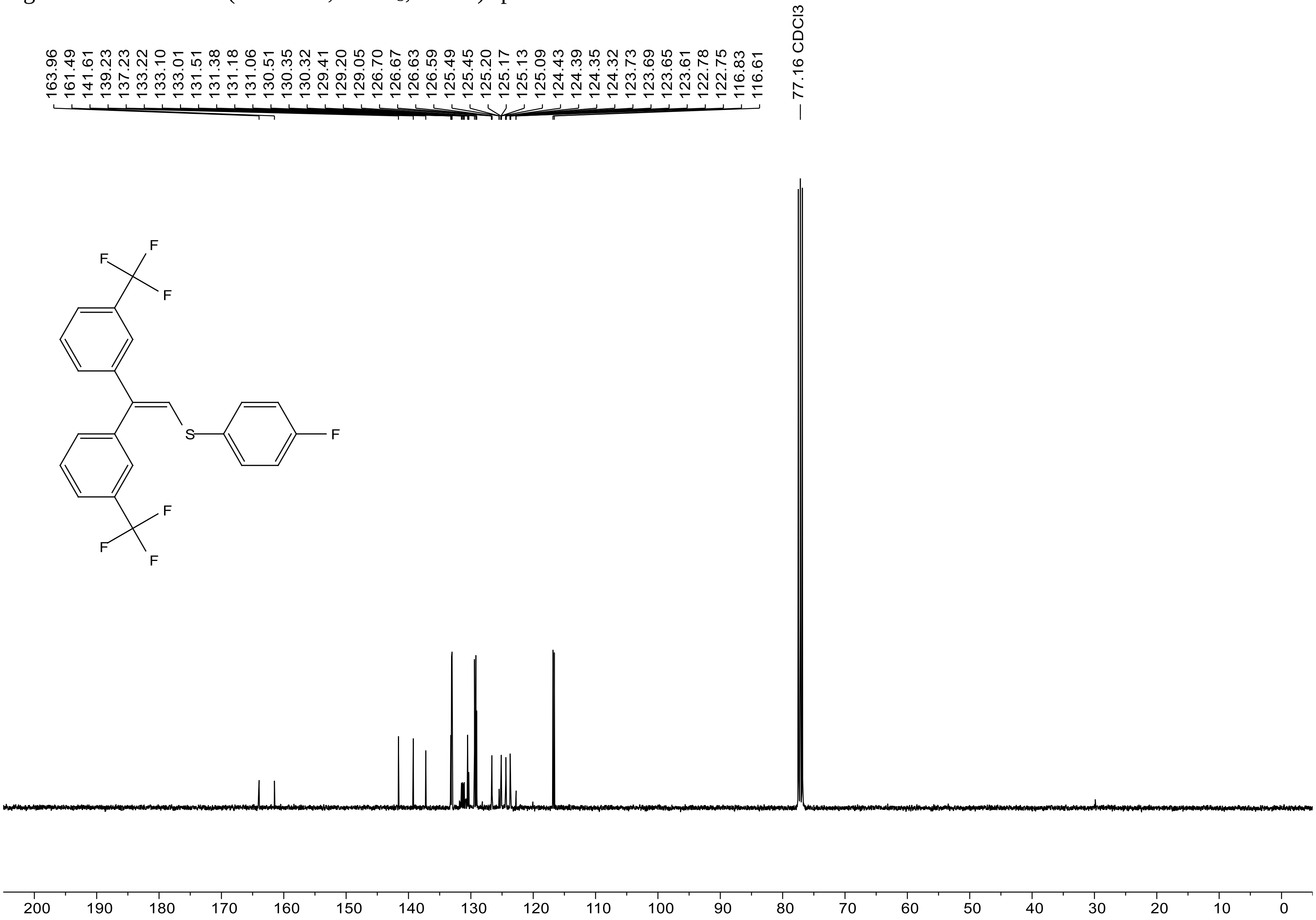

Figure S69: ^{19}**F NMR** (376 MHz, $CDCl_3$, 298 K) spectrum of **3ha**.

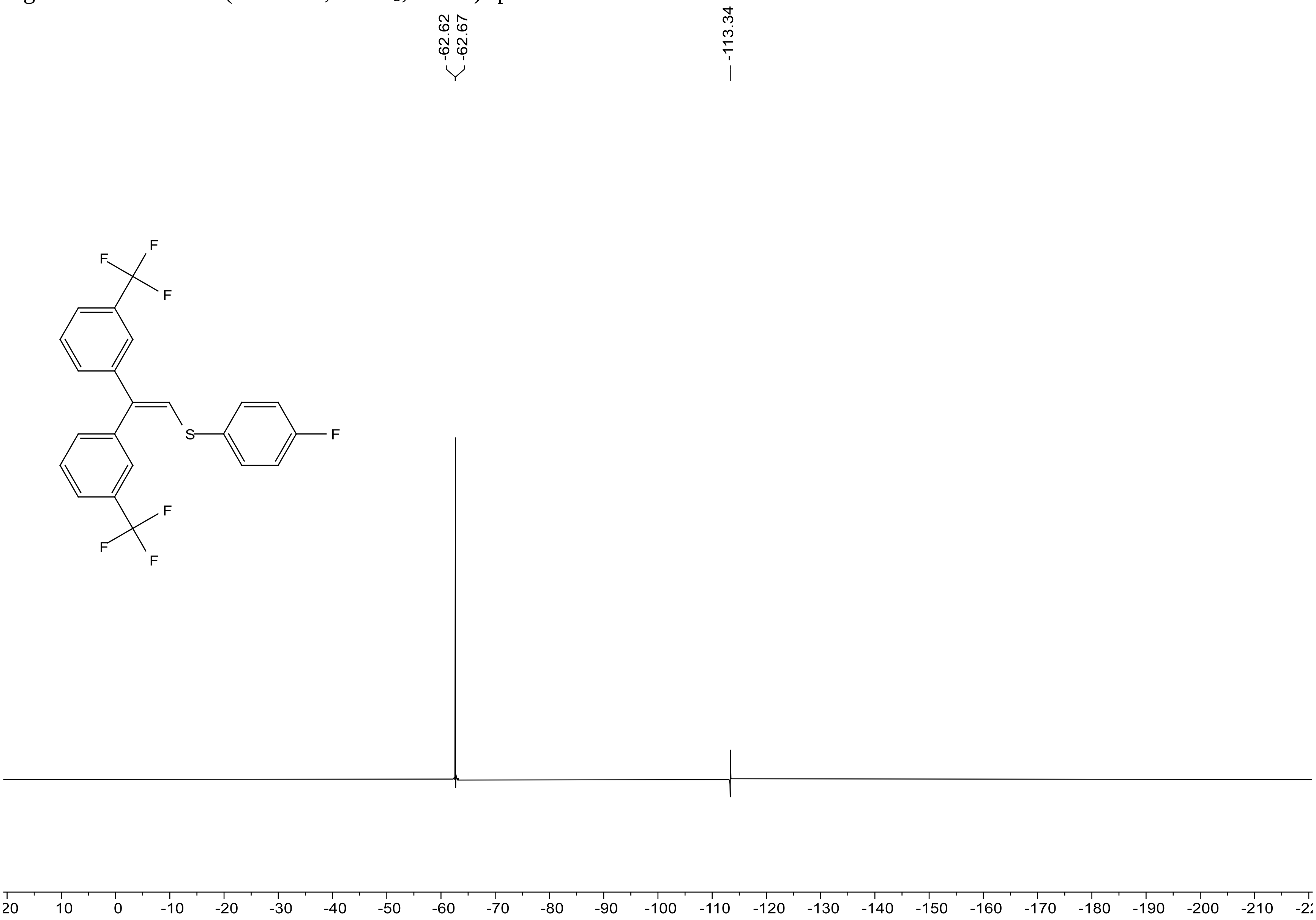

Figure S70: **^{1}H NMR** (400 MHz, $CDCl_3$, 298 K) spectrum of **3ia** (*E* and *Z*).

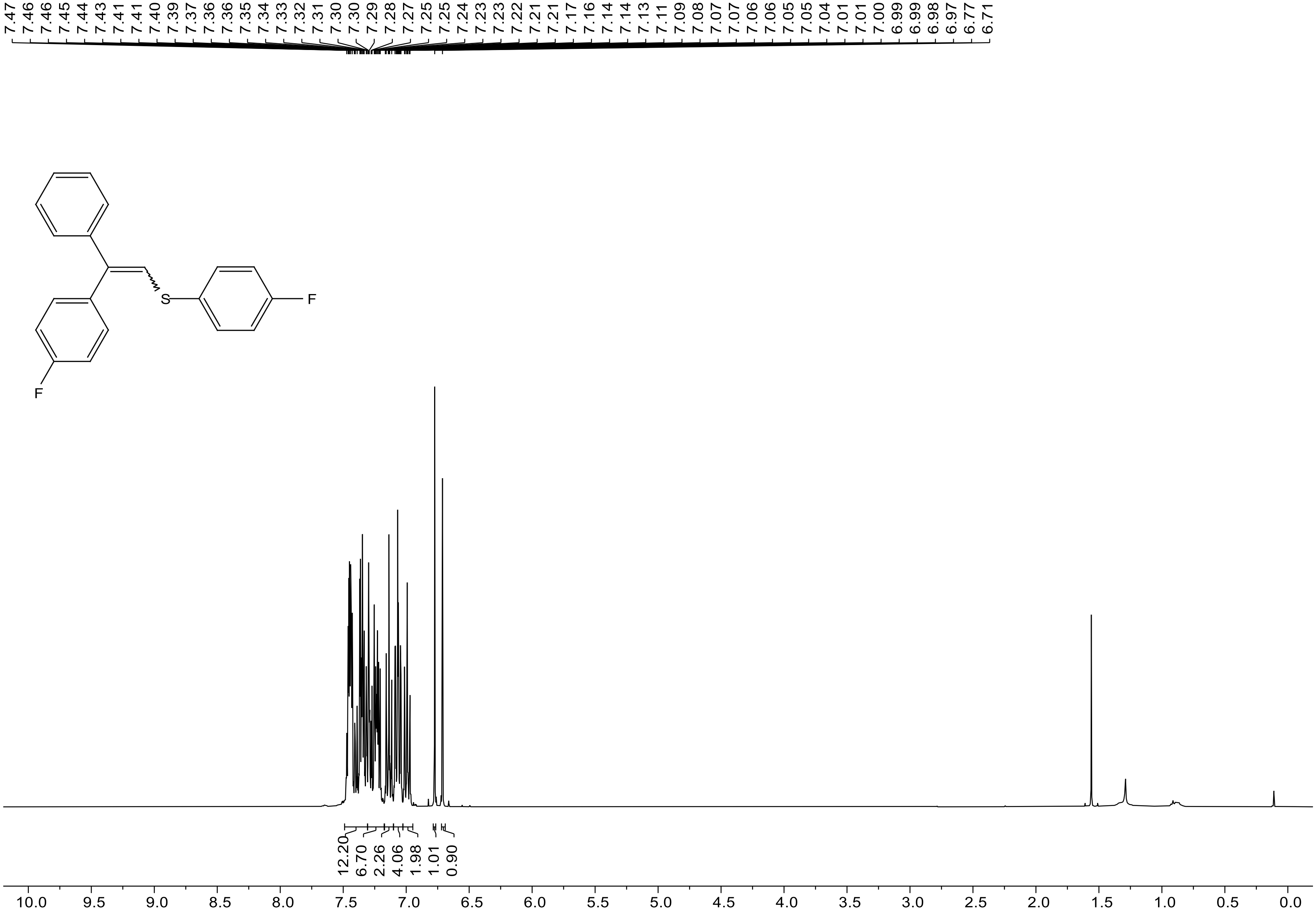

Figure S71: ^{13}**C NMR** (101 MHz, $CDCl_3$, 298 K) spectrum of **3ia** (*E* and *Z*).

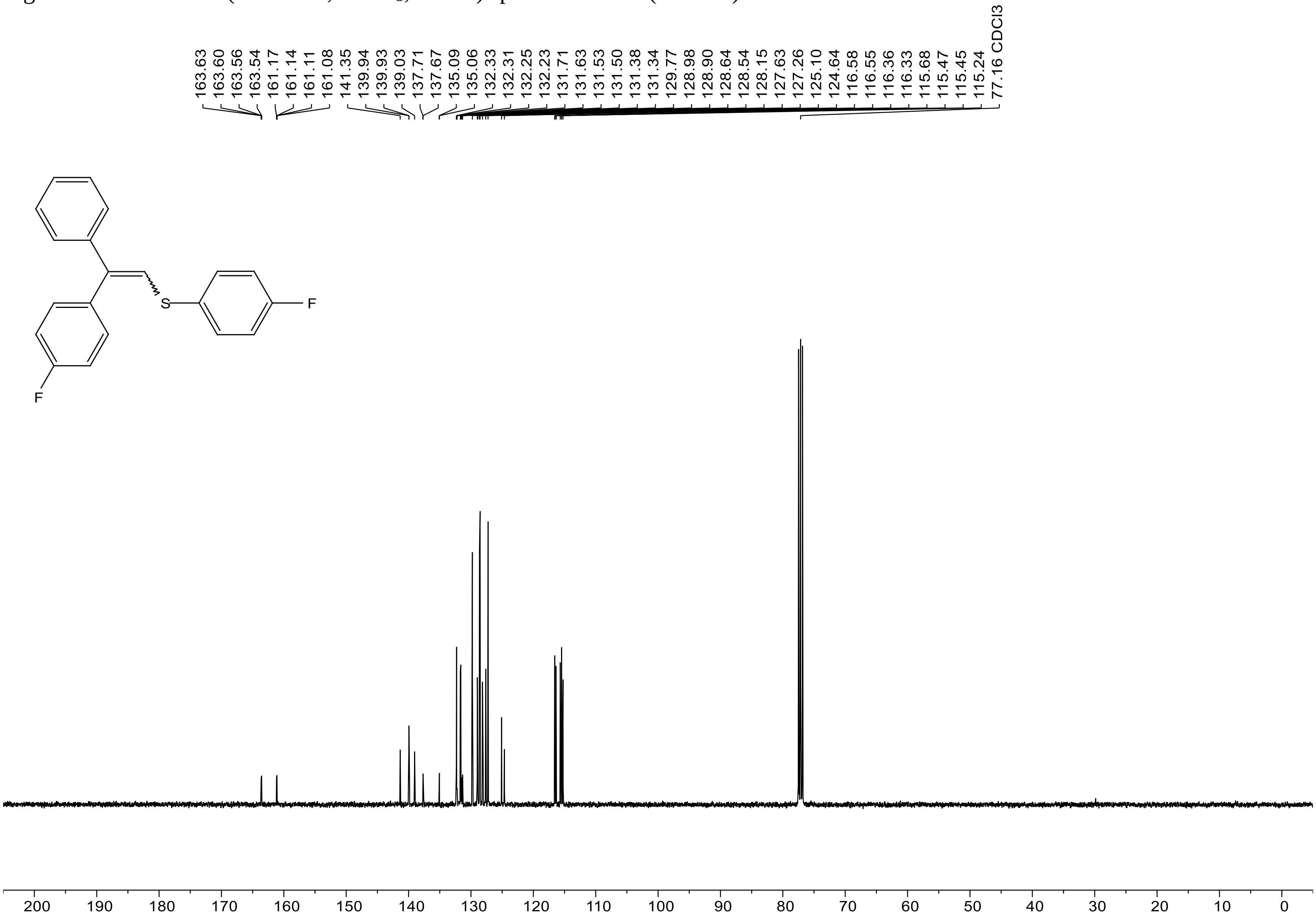

Figure S72: **^{19}F NMR** (376 MHz, $CDCl_3$, 298 K) spectrum of **3ia** (*E* and *Z*).

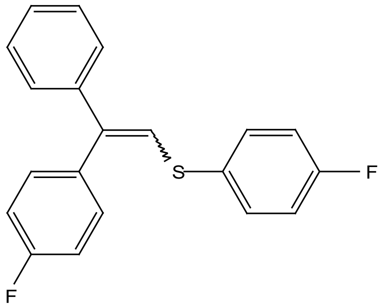

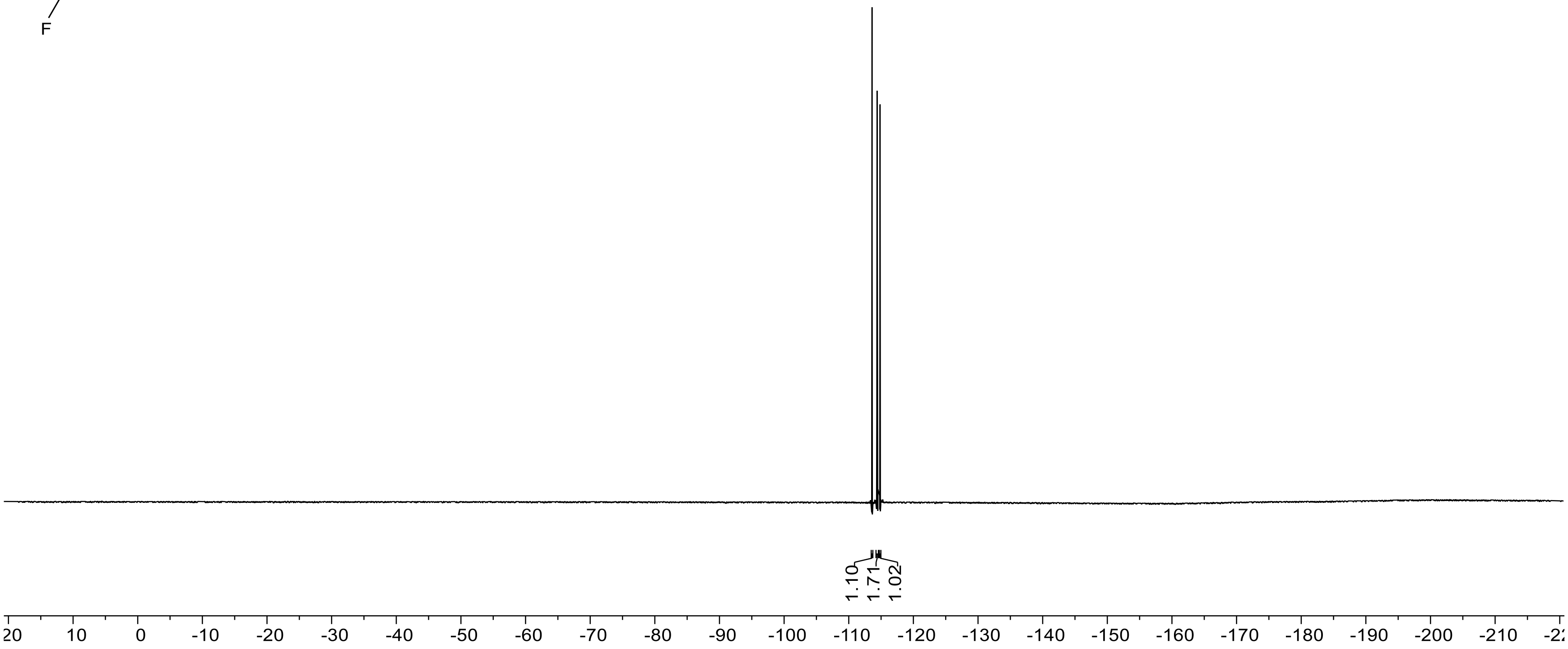

Figure S73: **^{1}H NMR** (400 MHz, $CDCl_3$, 298 K) spectrum of **3ja** (*E* and *Z*).

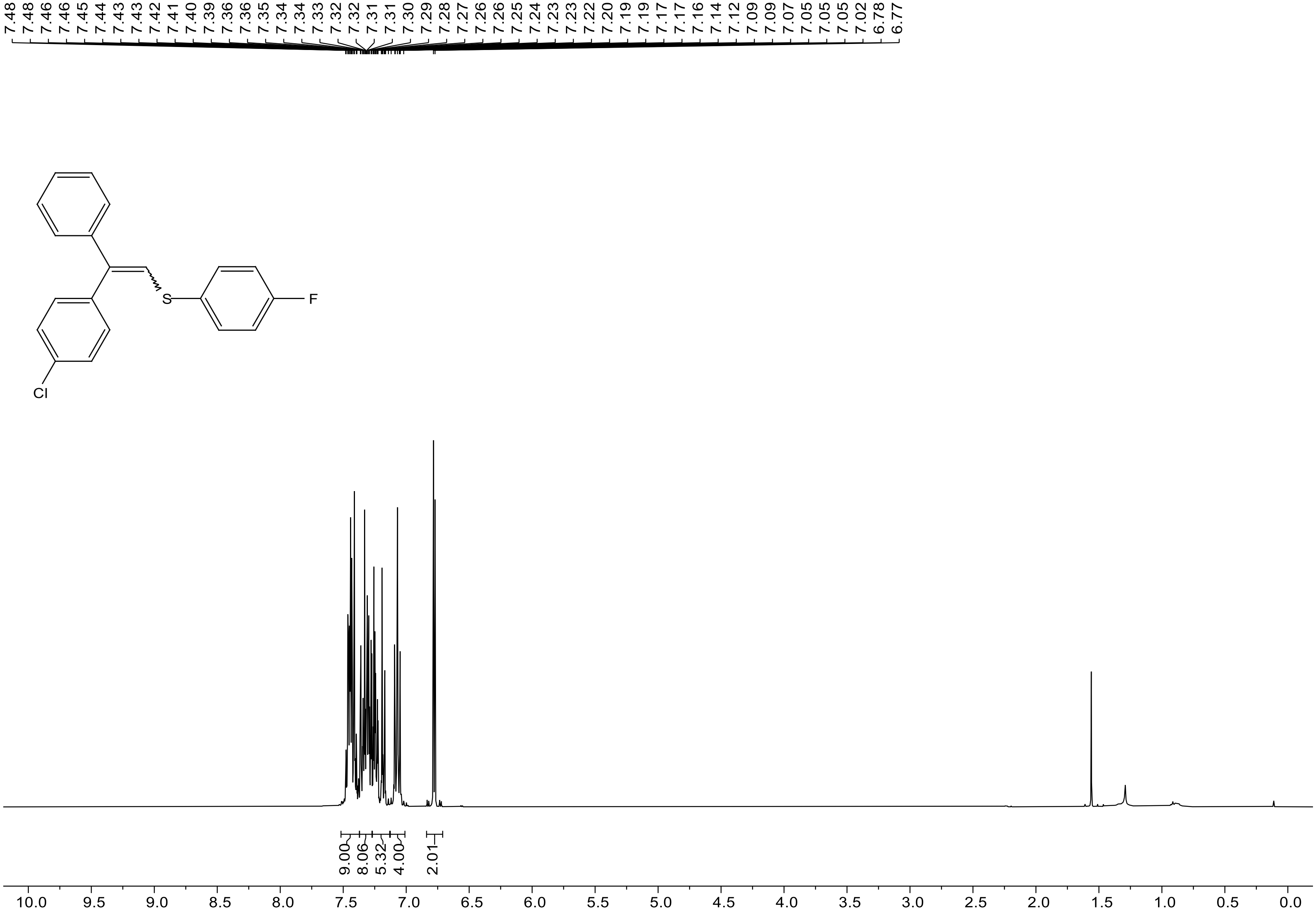

Figure S74: ^{13}**C NMR** (101 MHz, $CDCl_3$, 298 K) spectrum of **3ja** (*E* and *Z*).

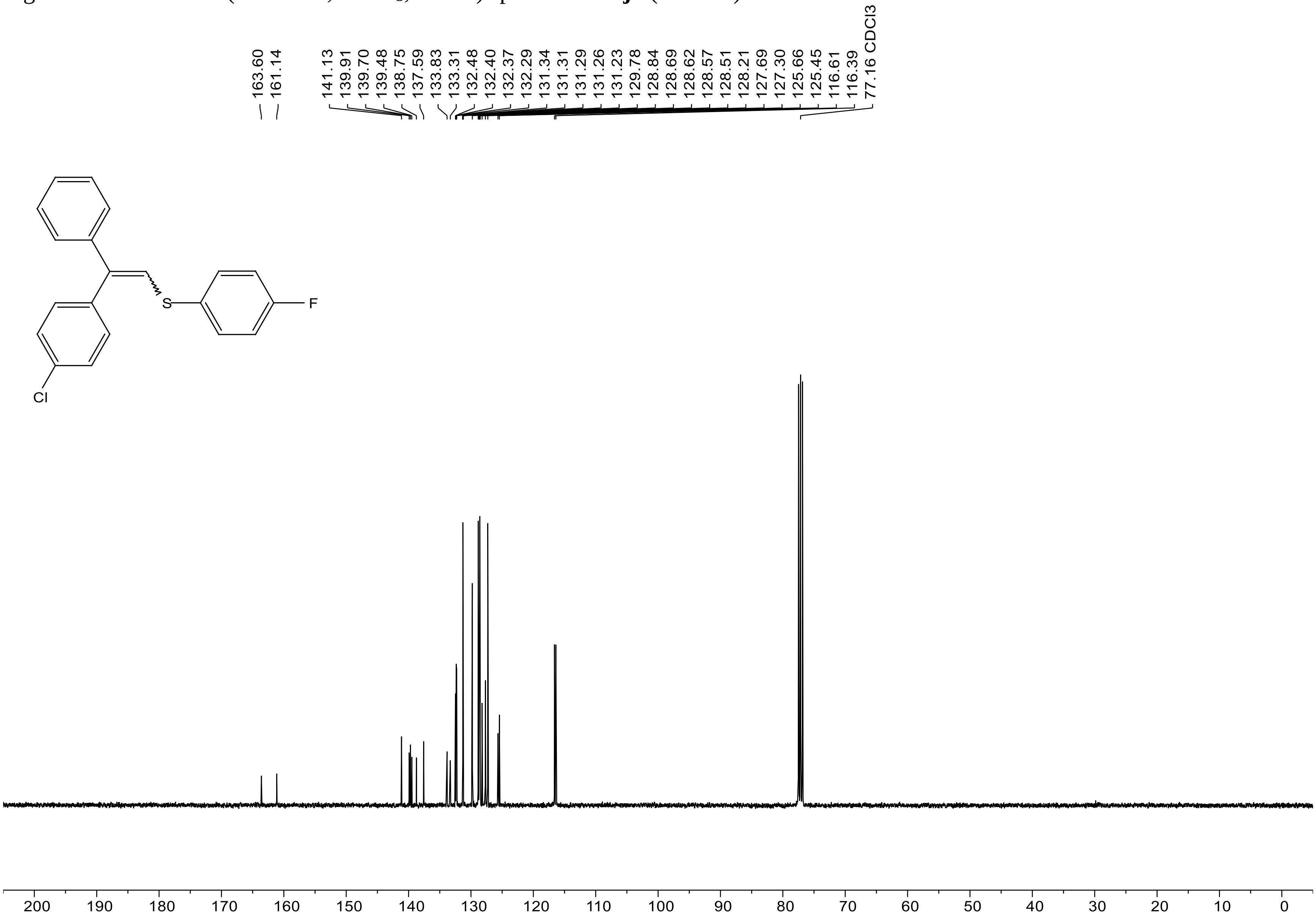

Figure S75: **^{19}F NMR** (376 MHz, $CDCl_3$, 298 K) spectrum of **3ja** (*E* and *Z*).

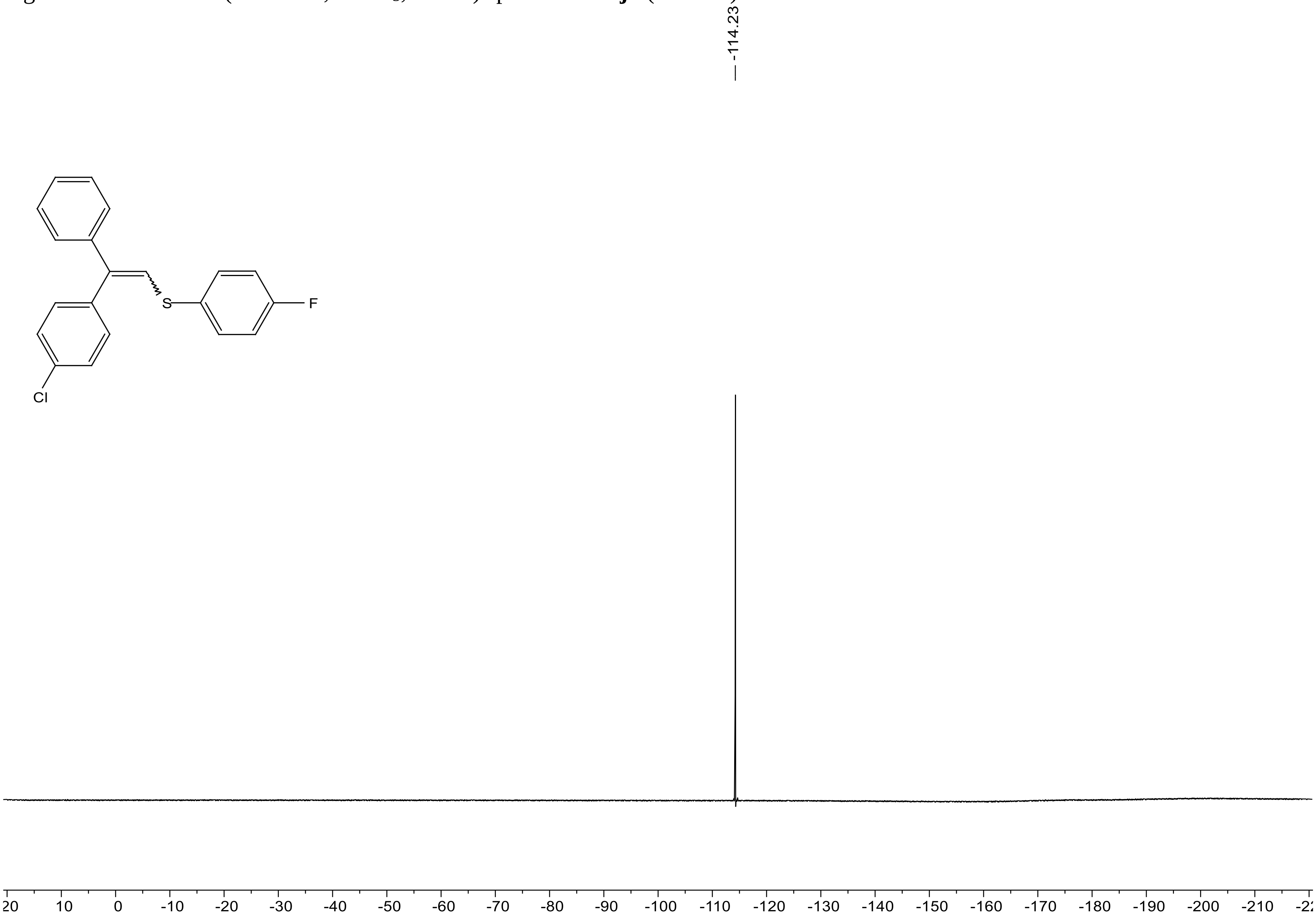

Figure S76: **^{1}H NMR** (400 MHz, $CDCl_3$, 298 K) spectrum of **3ka** (*E* and *Z*).

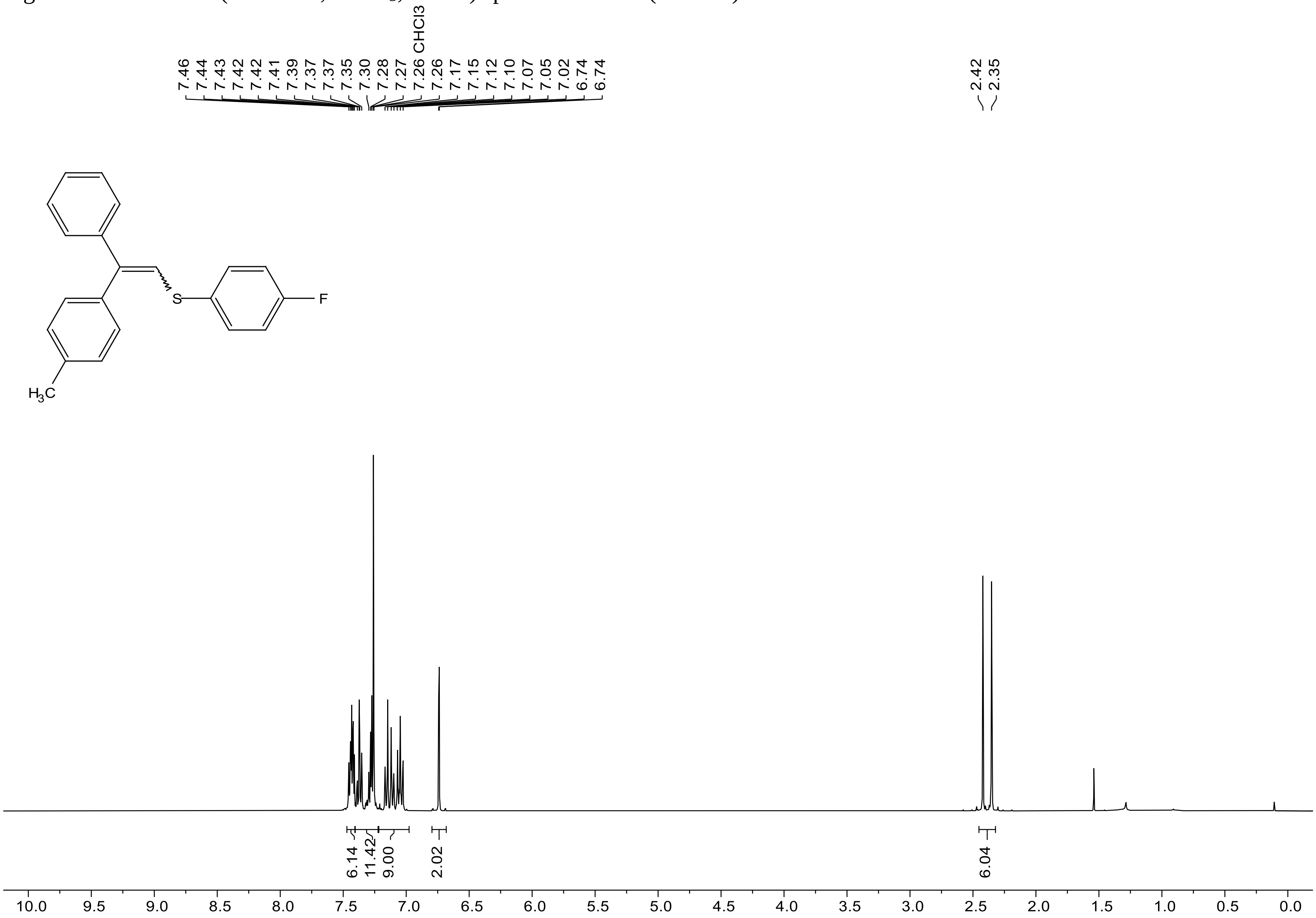

Figure S77: ^{13}**C NMR** (101 MHz, $CDCl_3$, 298 K) spectrum of **3ka** (*E* and *Z*).

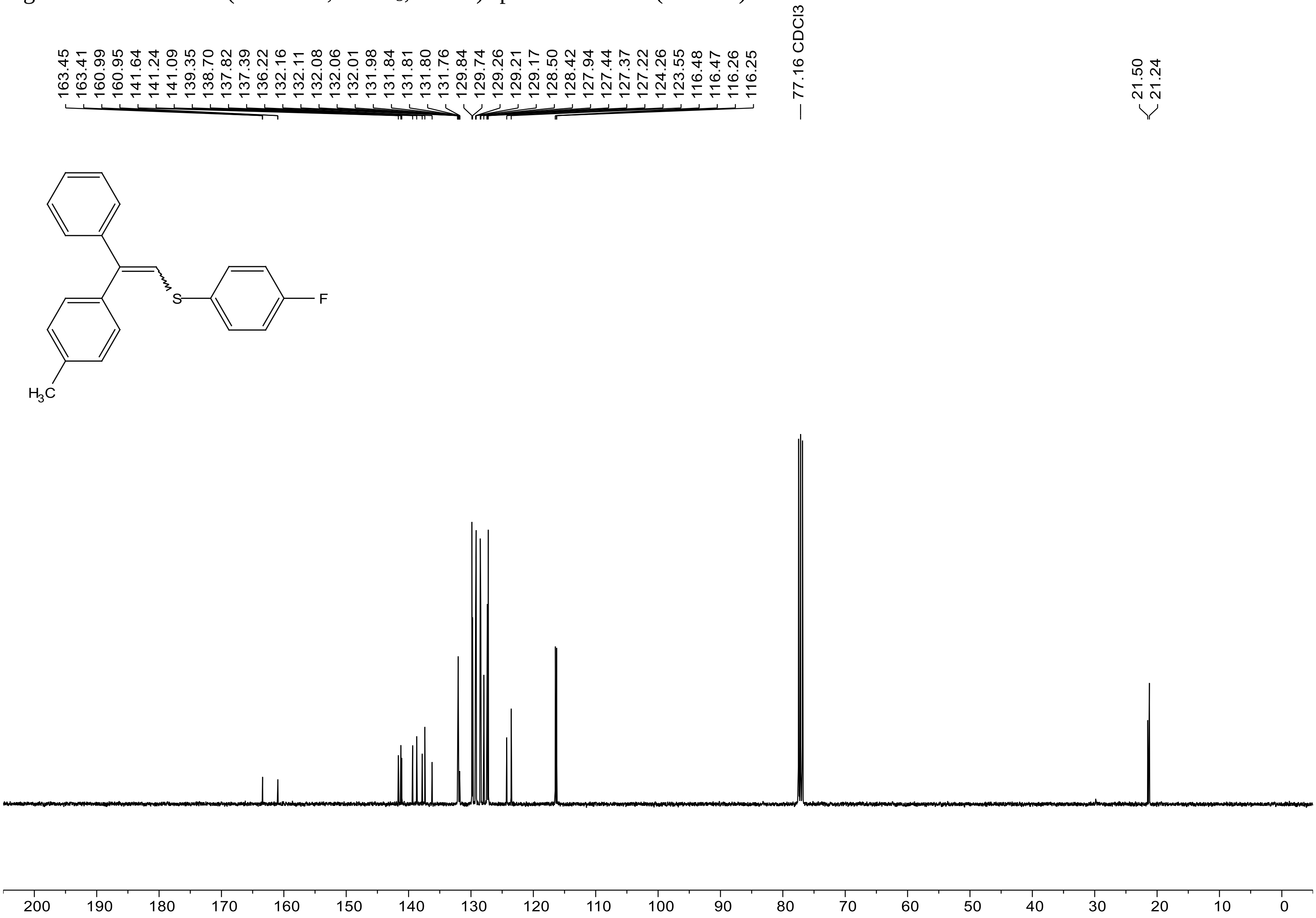

Figure S78: **^{19}F NMR** (376 MHz, $CDCl_3$, 298 K) spectrum of **3ka** (*E* and *Z*).

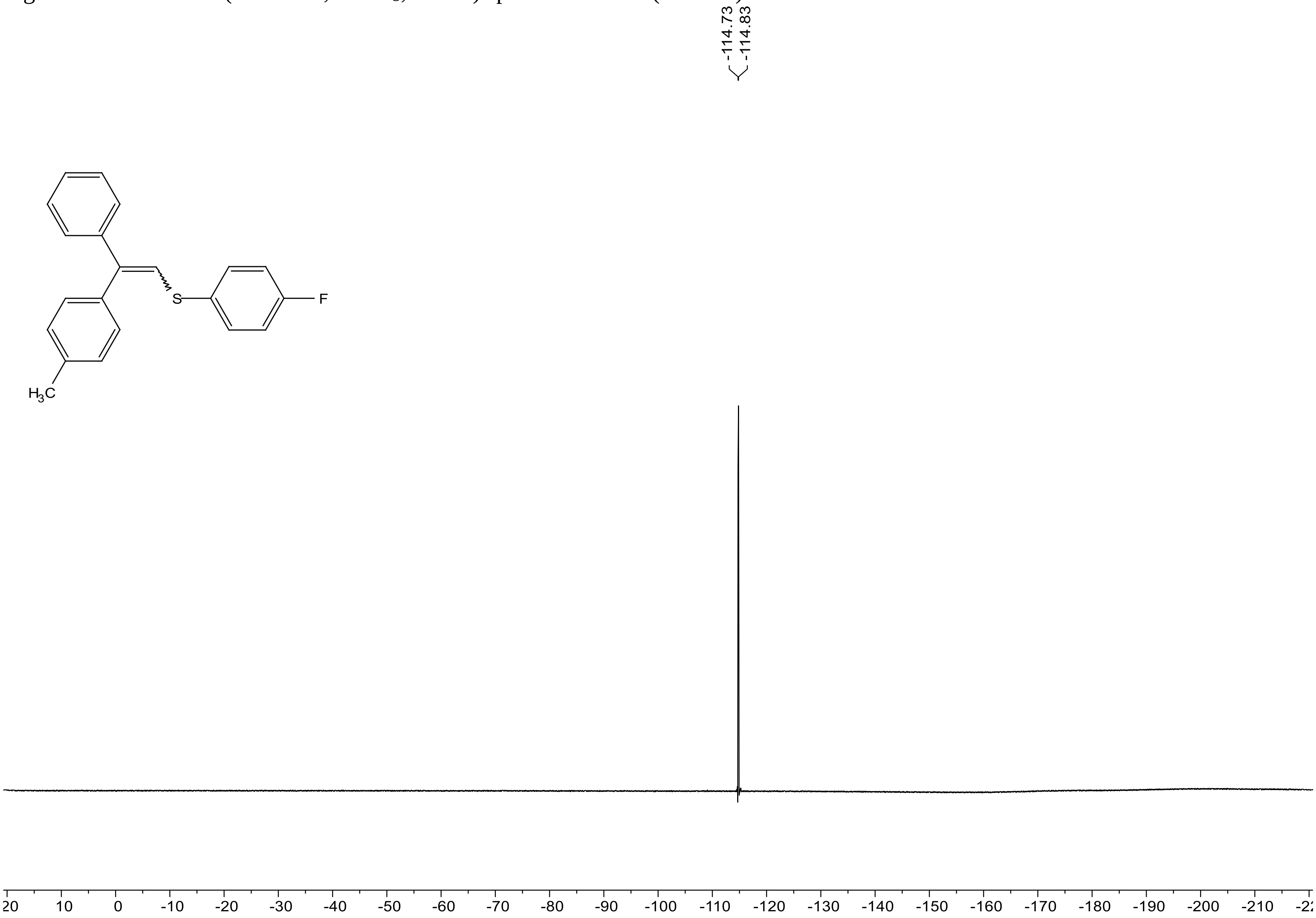

Figure S79: **^{1}H NMR** (400 MHz, $CDCl_3$, 298 K) spectrum of **3la** (*E* and *Z*).

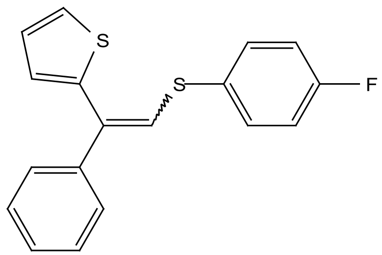

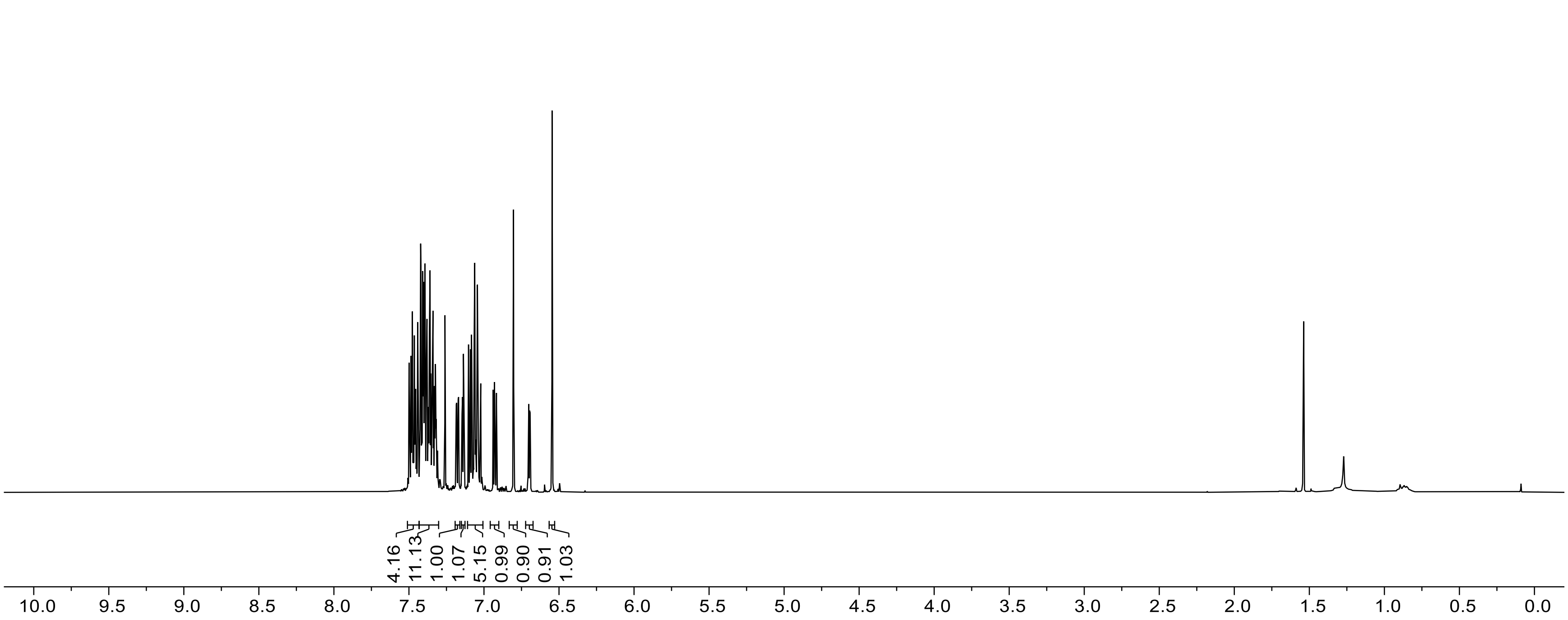

Figure S80: **^{13}C NMR** (101 MHz, $CDCl_3$, 298 K) spectrum of **3la** (*E* and *Z*).

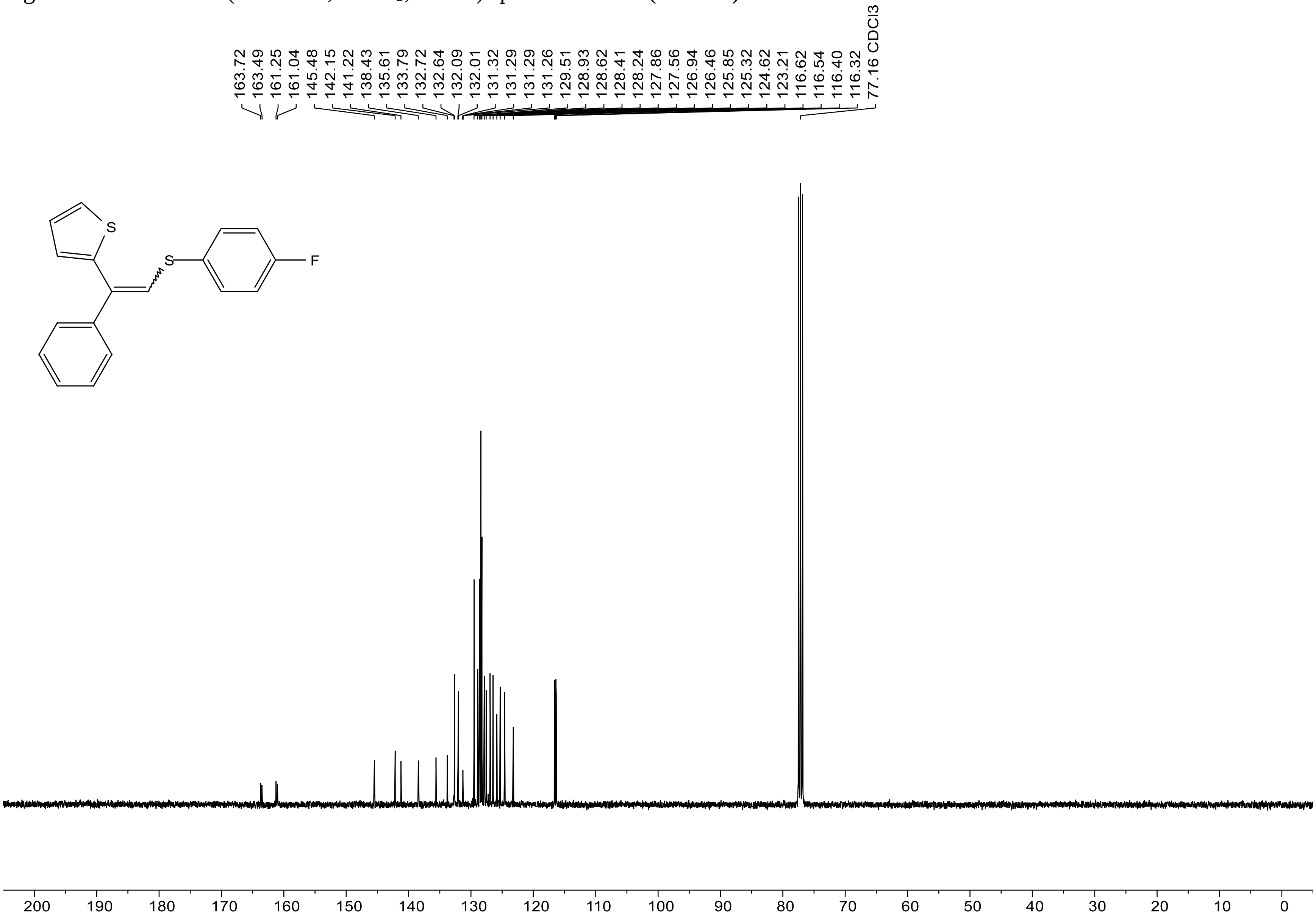

Figure S81: ^{19}**F NMR** (376 MHz, $CDCl_3$, 298 K) spectrum of **3la** (*E* and *Z*).

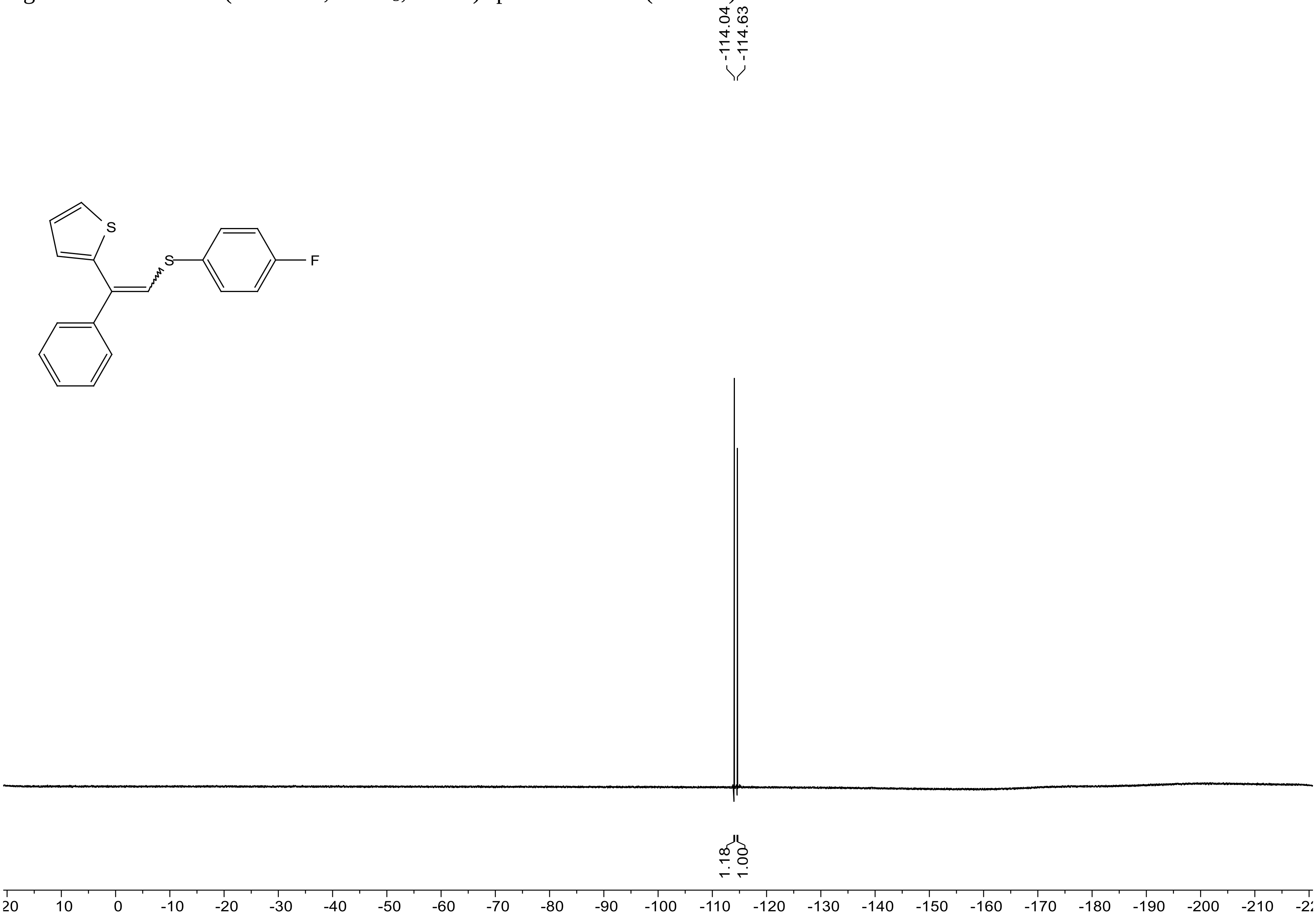

Figure S82: **^{1}H NMR** (400 MHz, $CDCl_3$, 298 K) spectrum of **3na**.

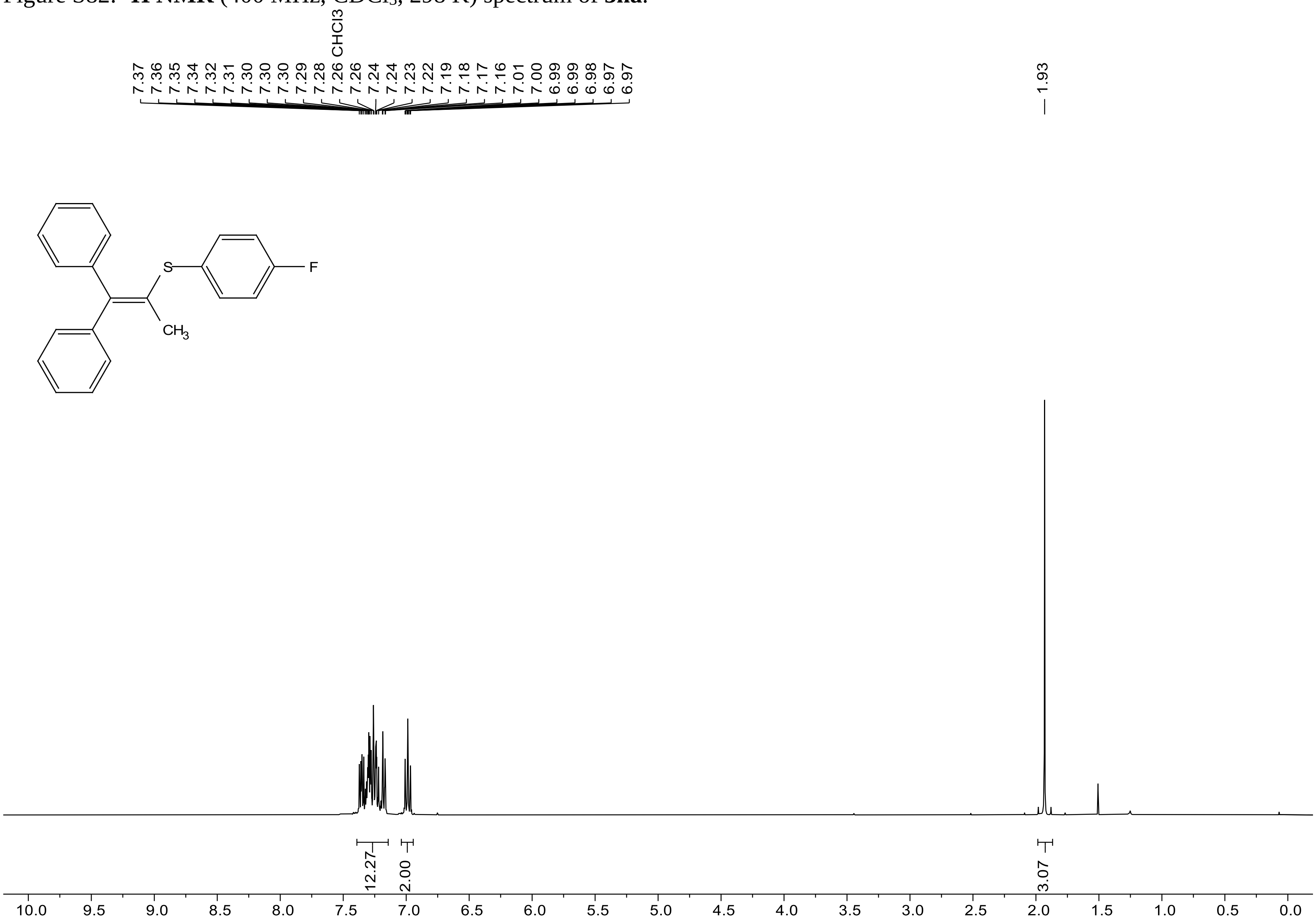

Figure S83: **^{13}C NMR** (101 MHz, $CDCl_3$, 298 K) spectrum of **3na**.

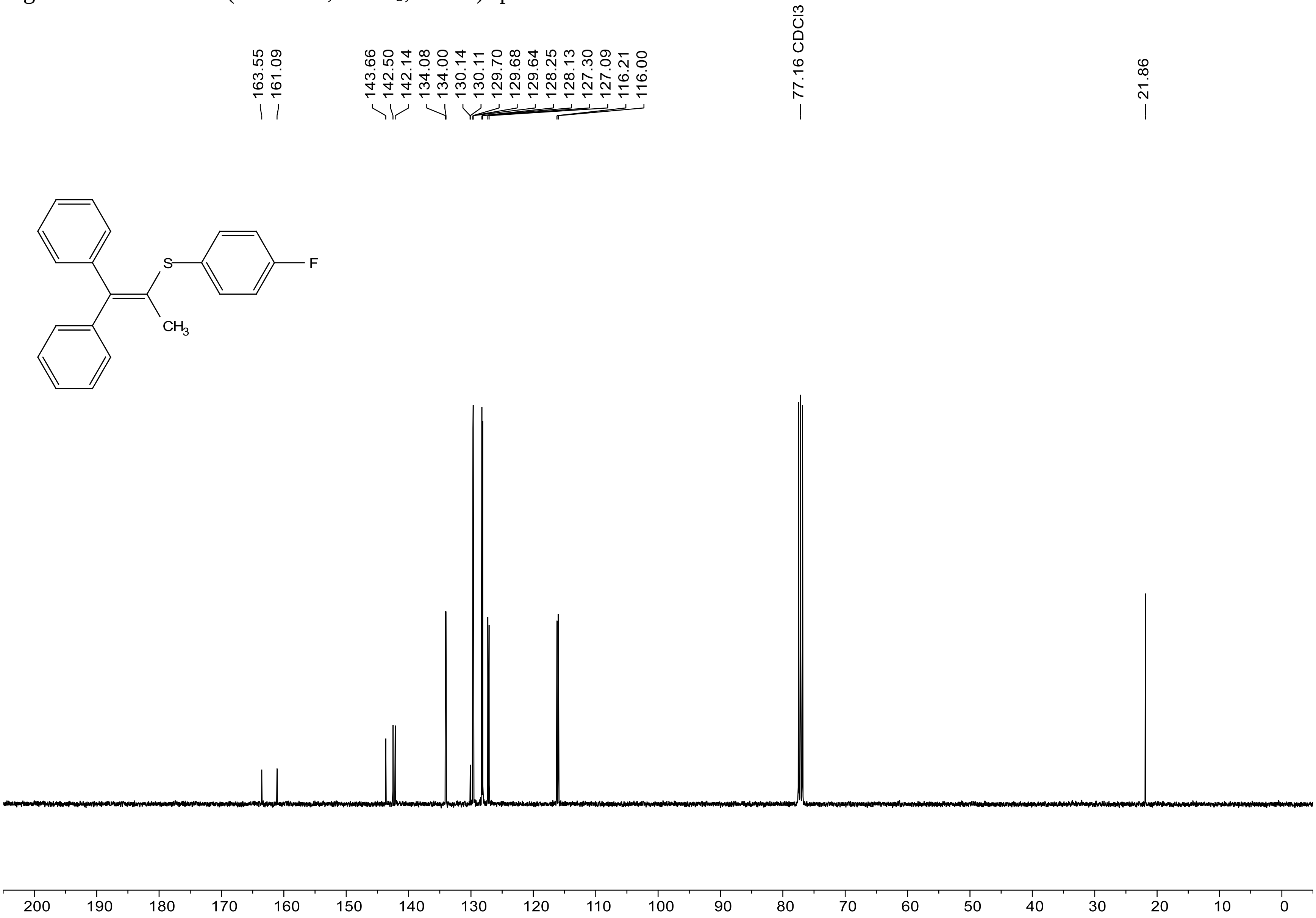

Figure S84: **^{19}F NMR** (376 MHz, $CDCl_3$, 298 K) spectrum of **3na**.

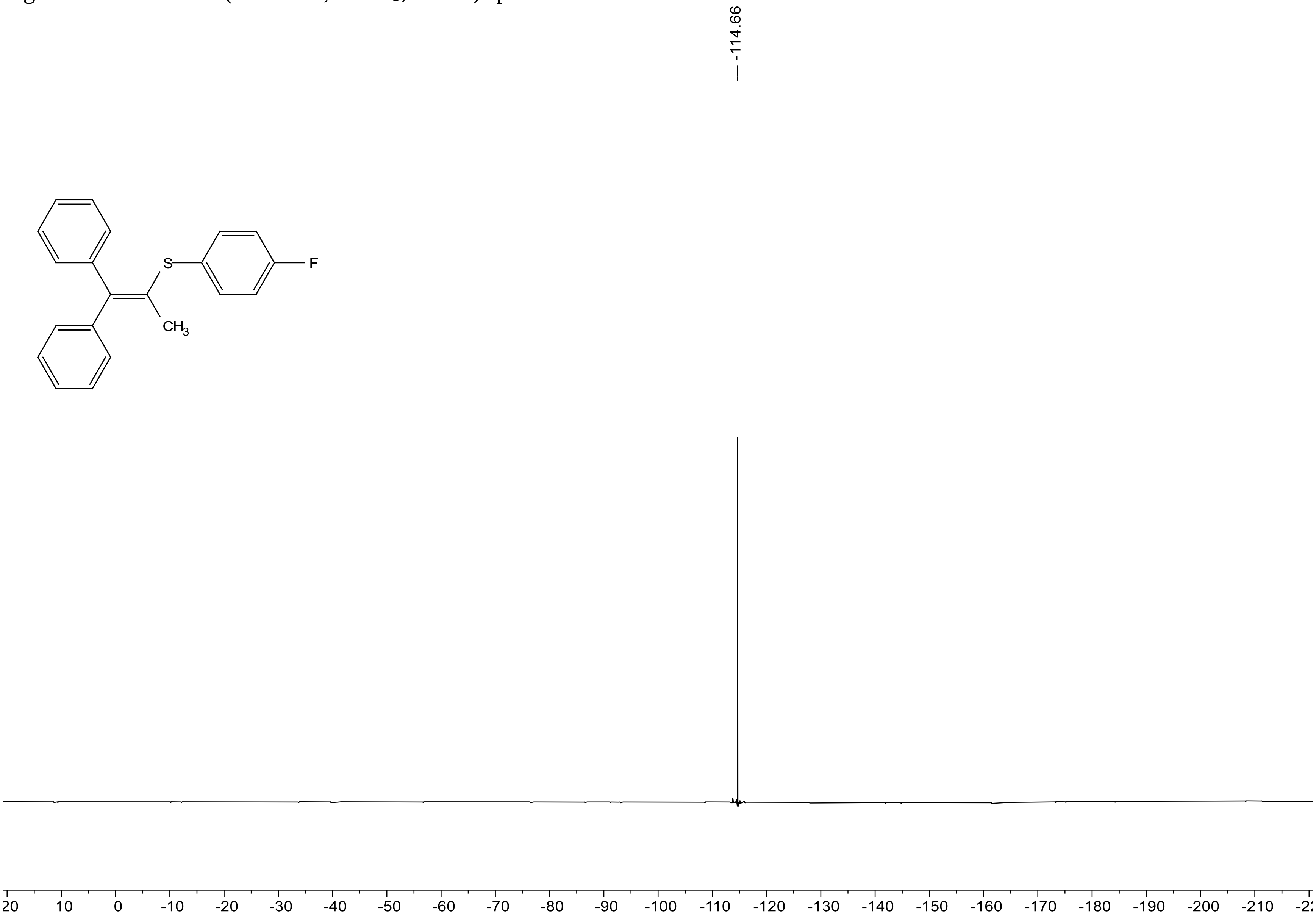

Figure S85: **^{1}H NMR** (400 MHz, $CDCl_3$, 298 K) spectrum of **3oa**.

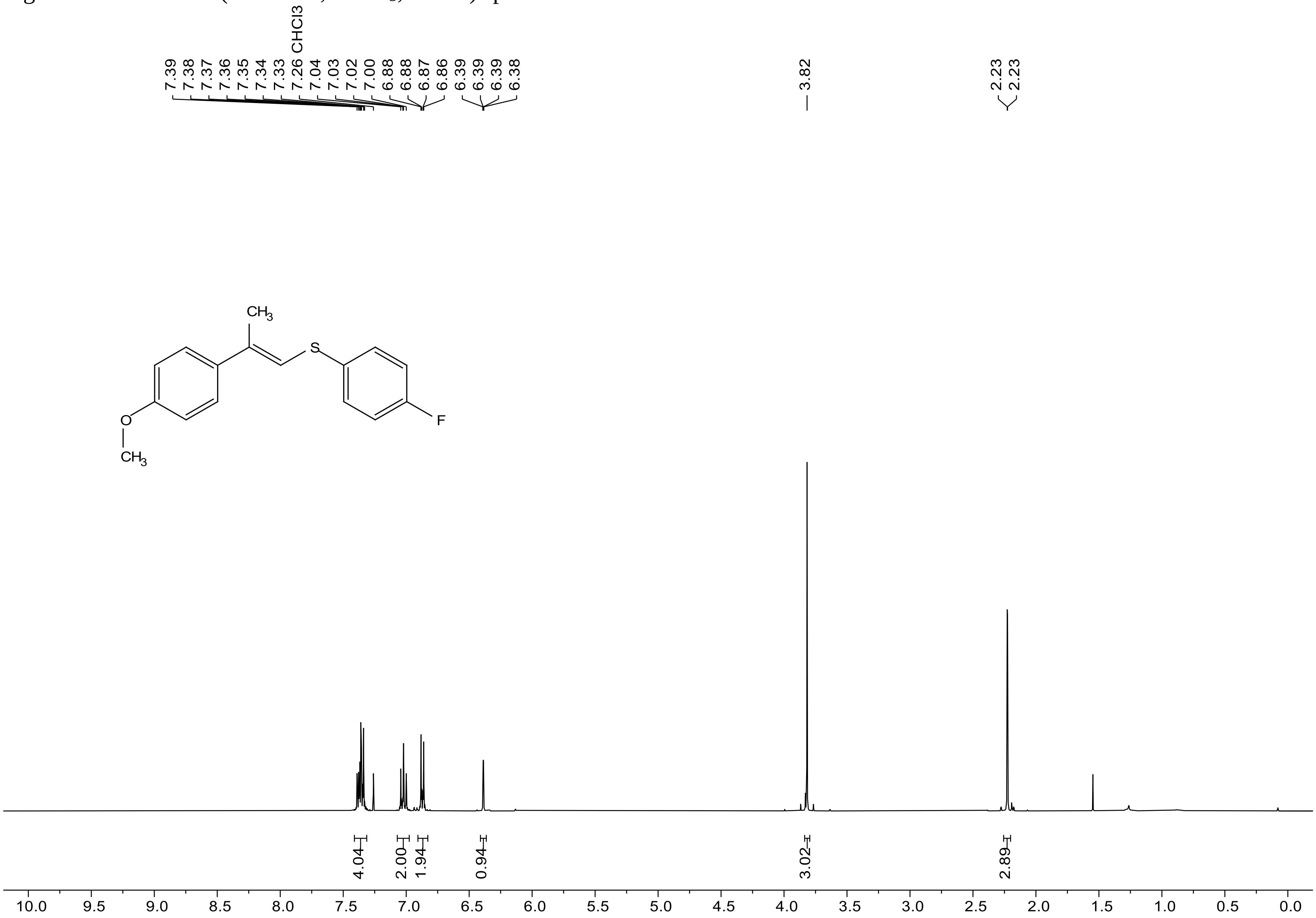

Figure S86: **^{13}C NMR** (101 MHz, $CDCl_3$, 298 K) spectrum of **3oa**.

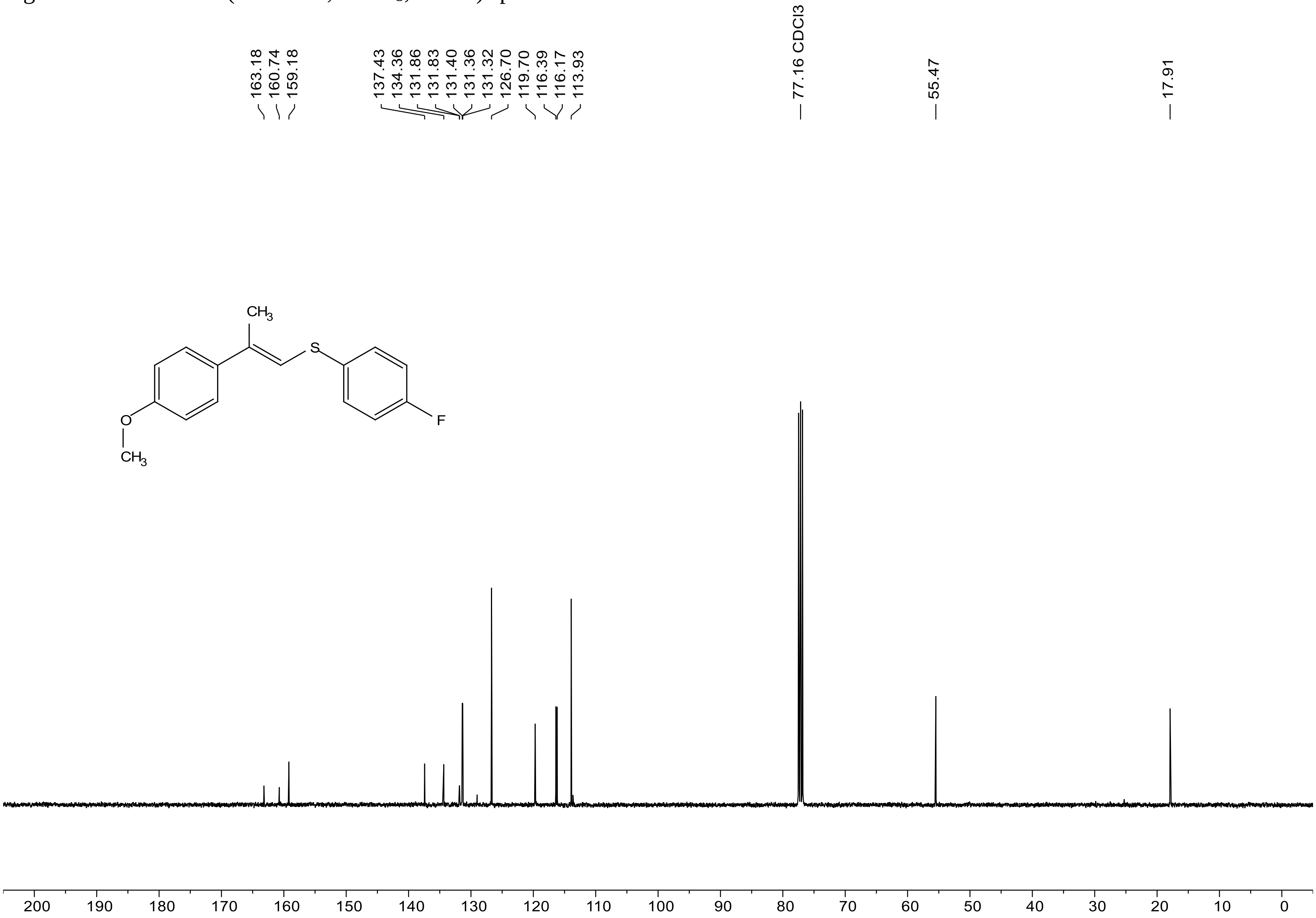

Figure S87: ^{19}**F NMR** (376 MHz, $CDCl_3$, 298 K) spectrum of **3oa**.

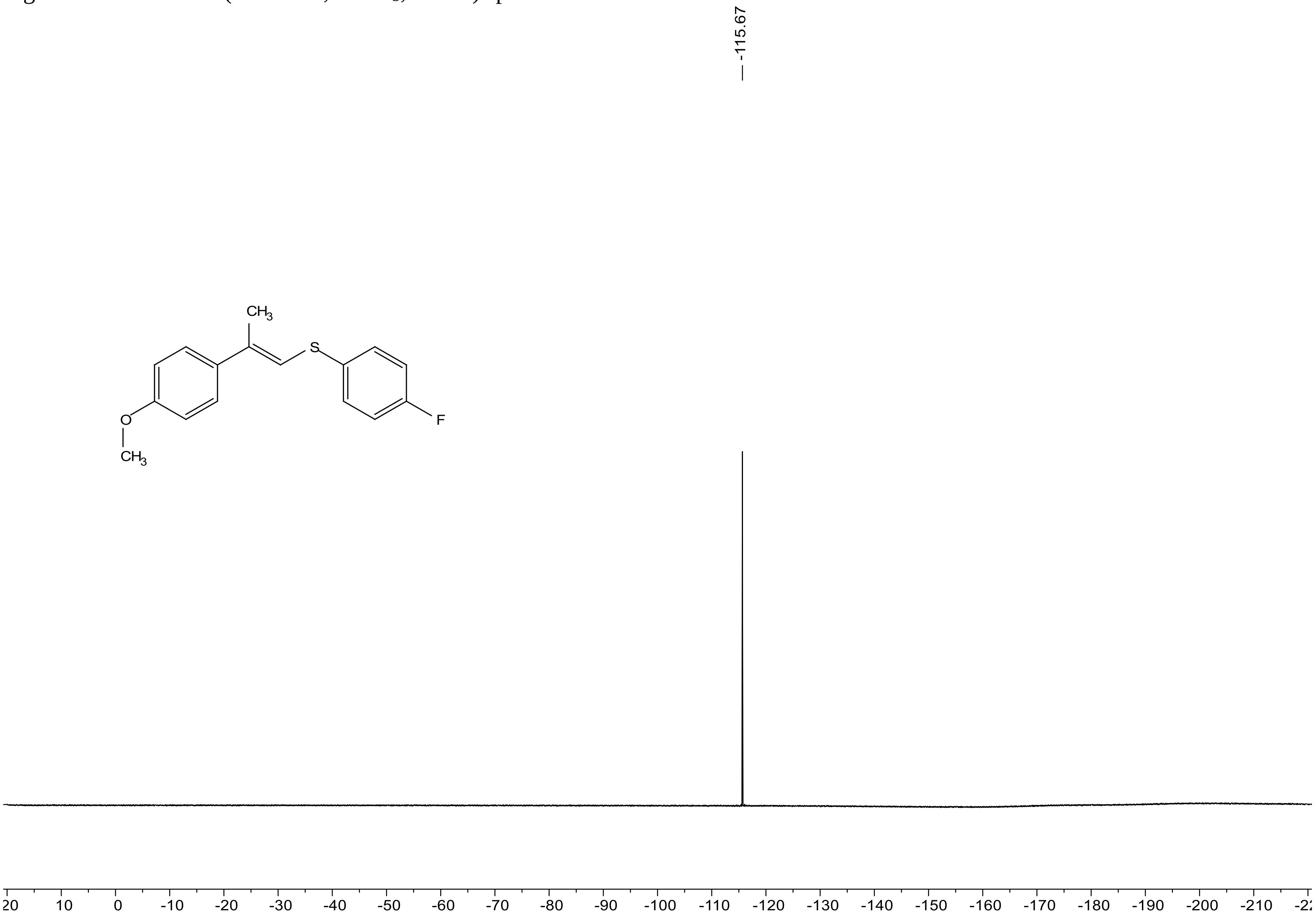

Figure S88: **^{1}H NMR** (400 MHz, $CDCl_3$, 298 K) spectrum of **3ob**.

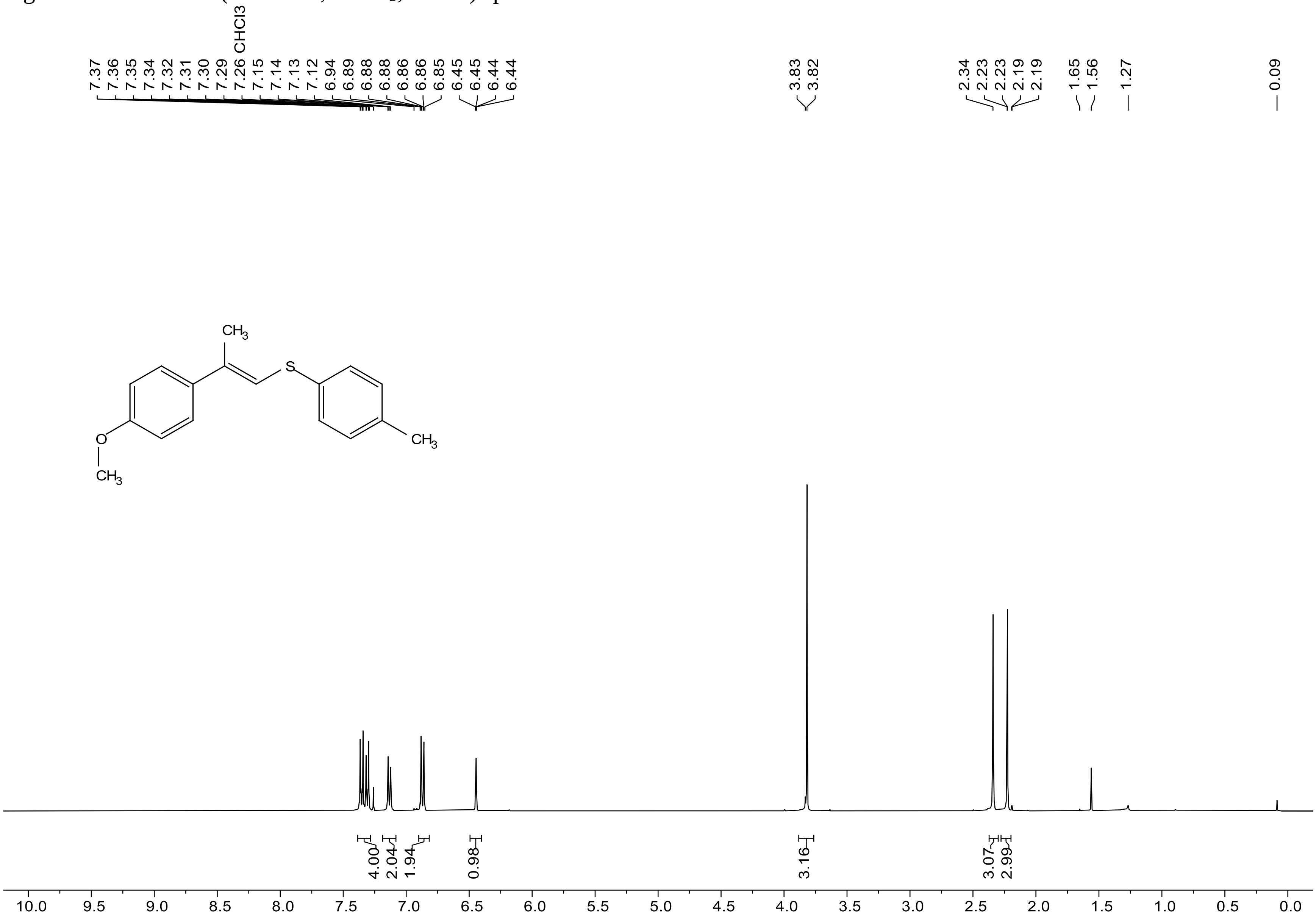

Figure S89: **^{13}C NMR** (101 MHz, $CDCl_3$, 298 K) spectrum of **3ob**.

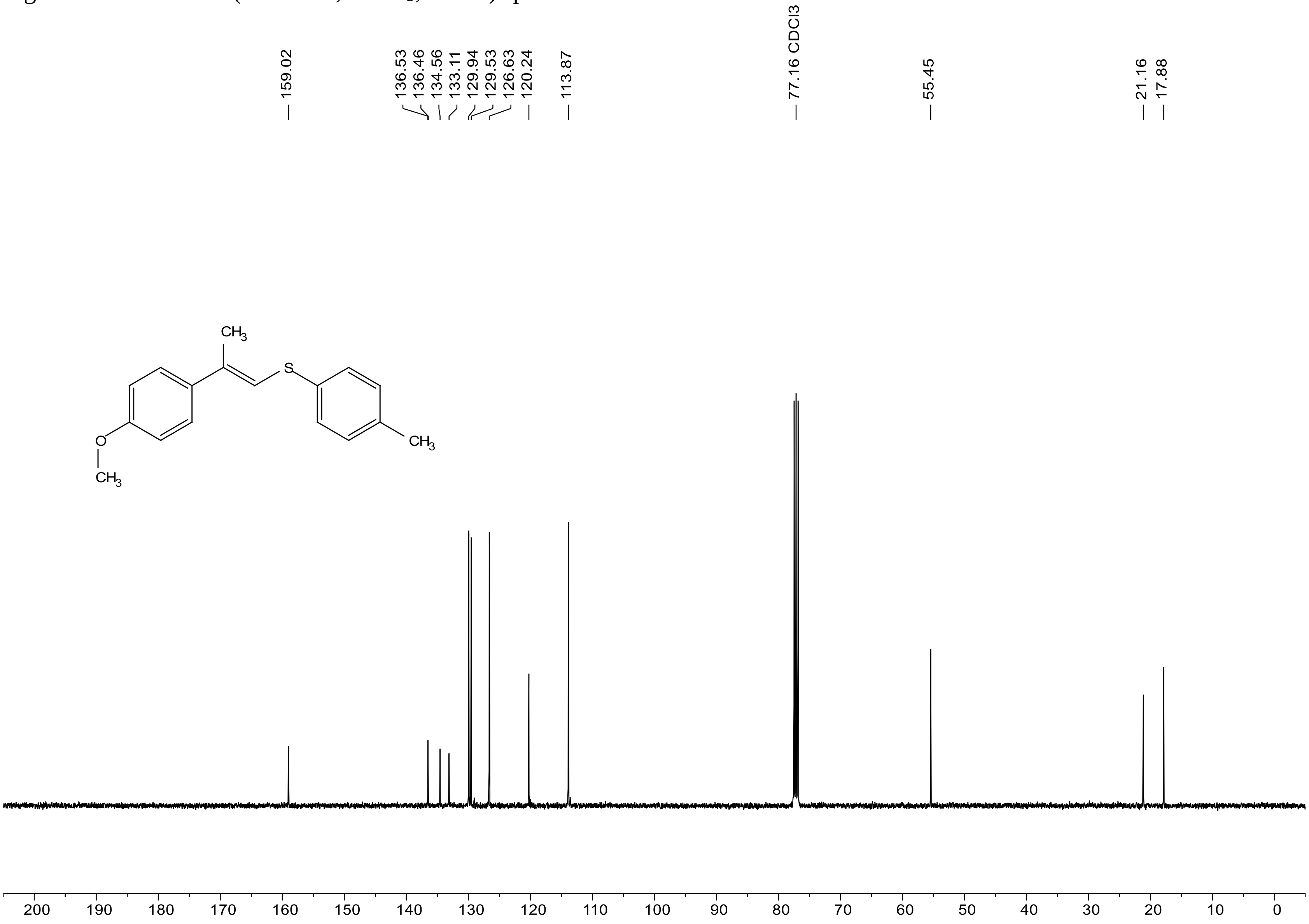

Figure S90: **^{1}H NMR** (400 MHz, $CDCl_3$, 298 K) spectrum of **3ac**.

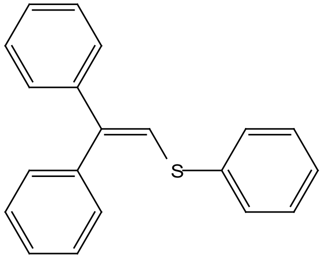

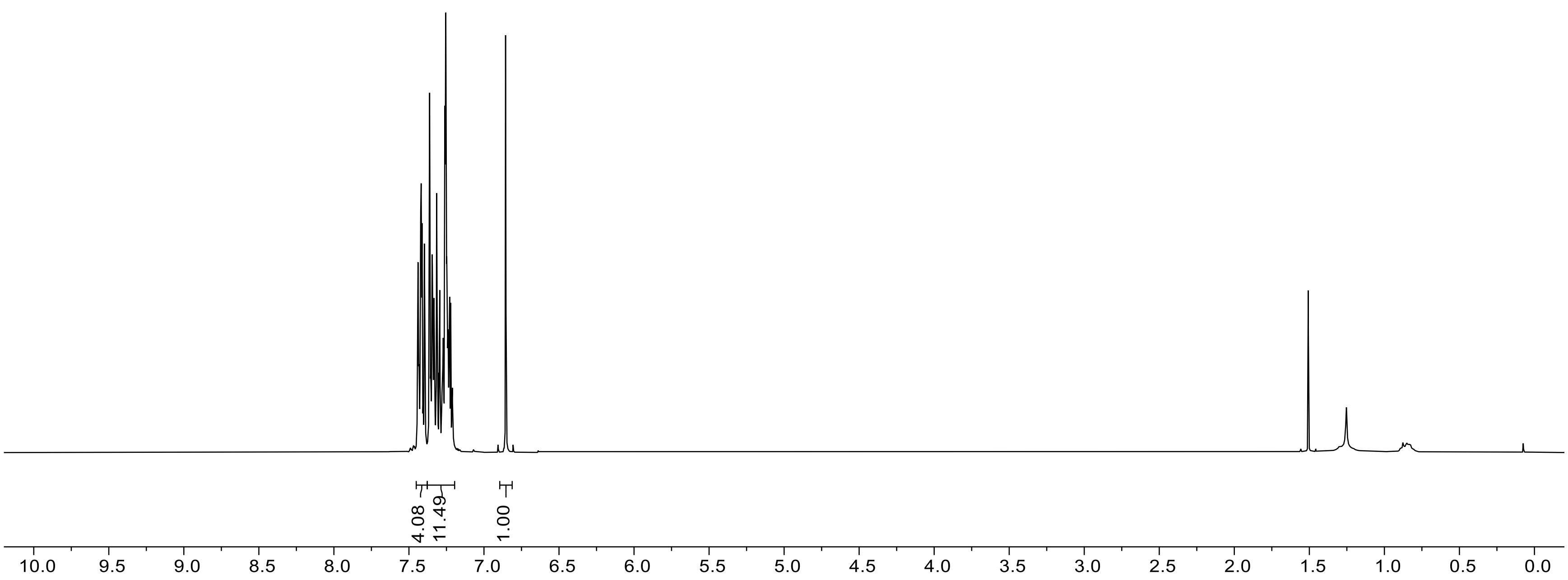

Figure S91: **^{13}C NMR** (101 MHz, $CDCl_3$, 298 K) spectrum of **3ac**.

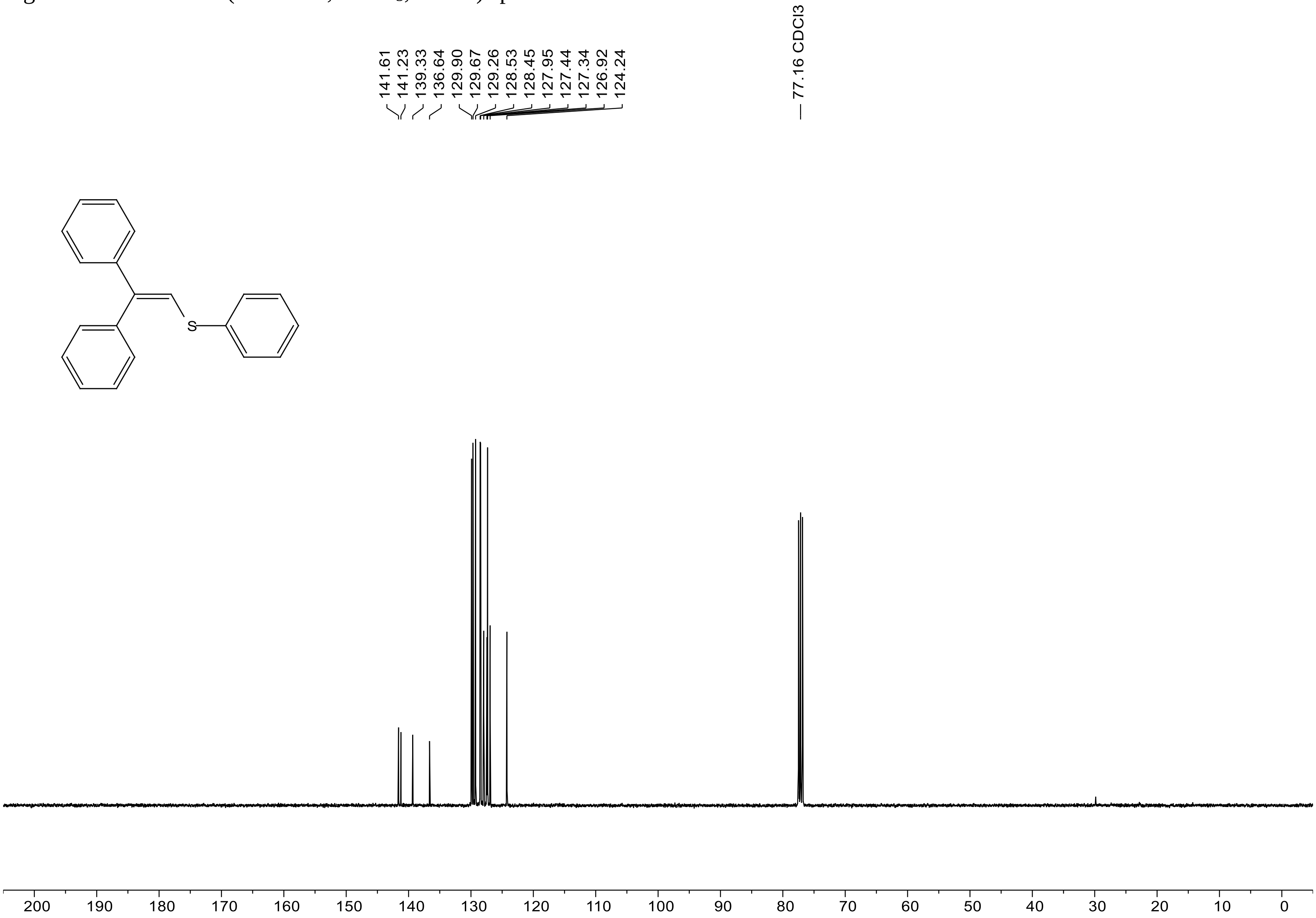

Figure S92: **^{1}H NMR** (400 MHz, $CDCl_3$, 298 K) spectrum of **3ab**.

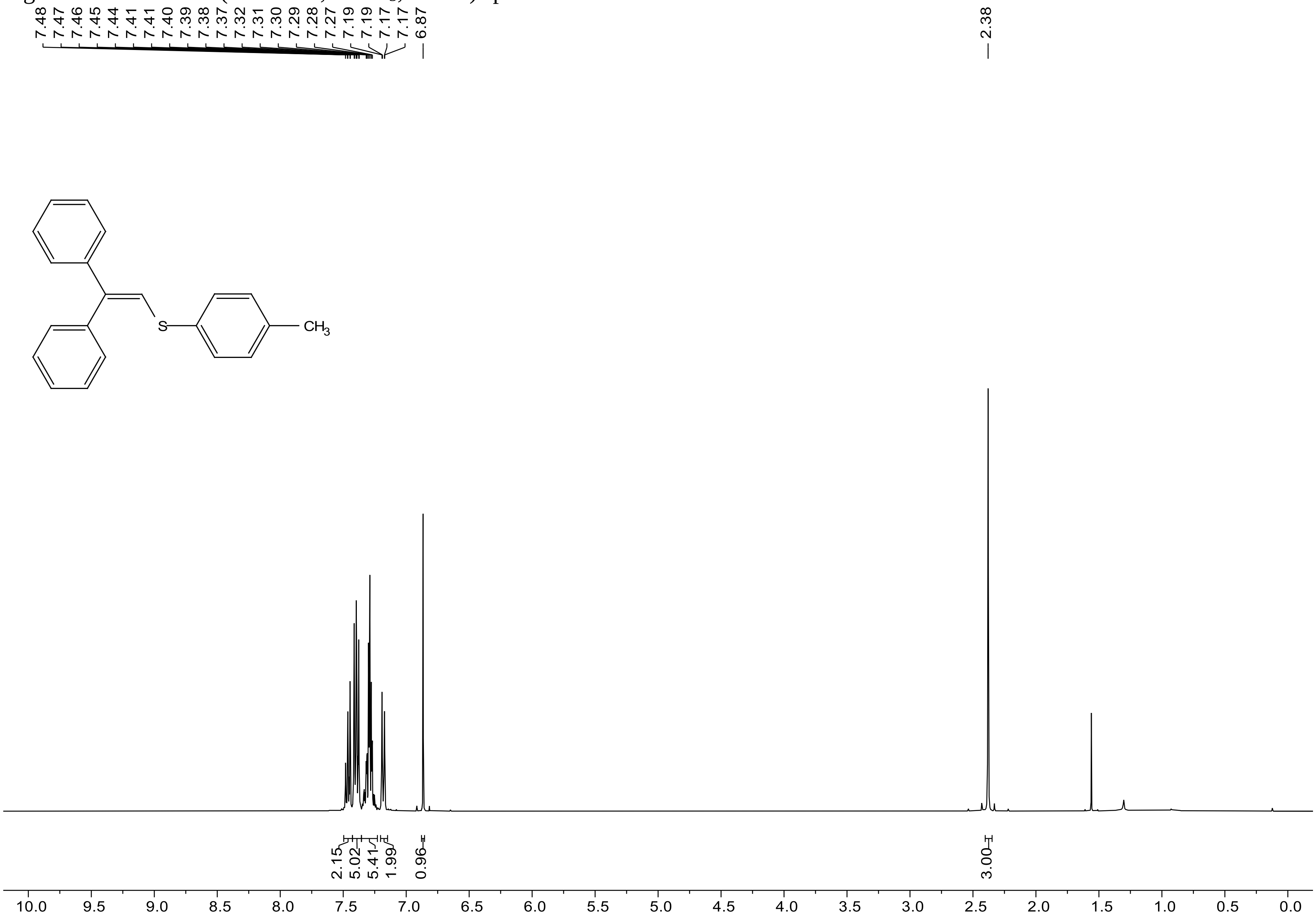

Figure S93: **^{13}C NMR** (101 MHz, $CDCl_3$, 298 K) spectrum of **3ab**.

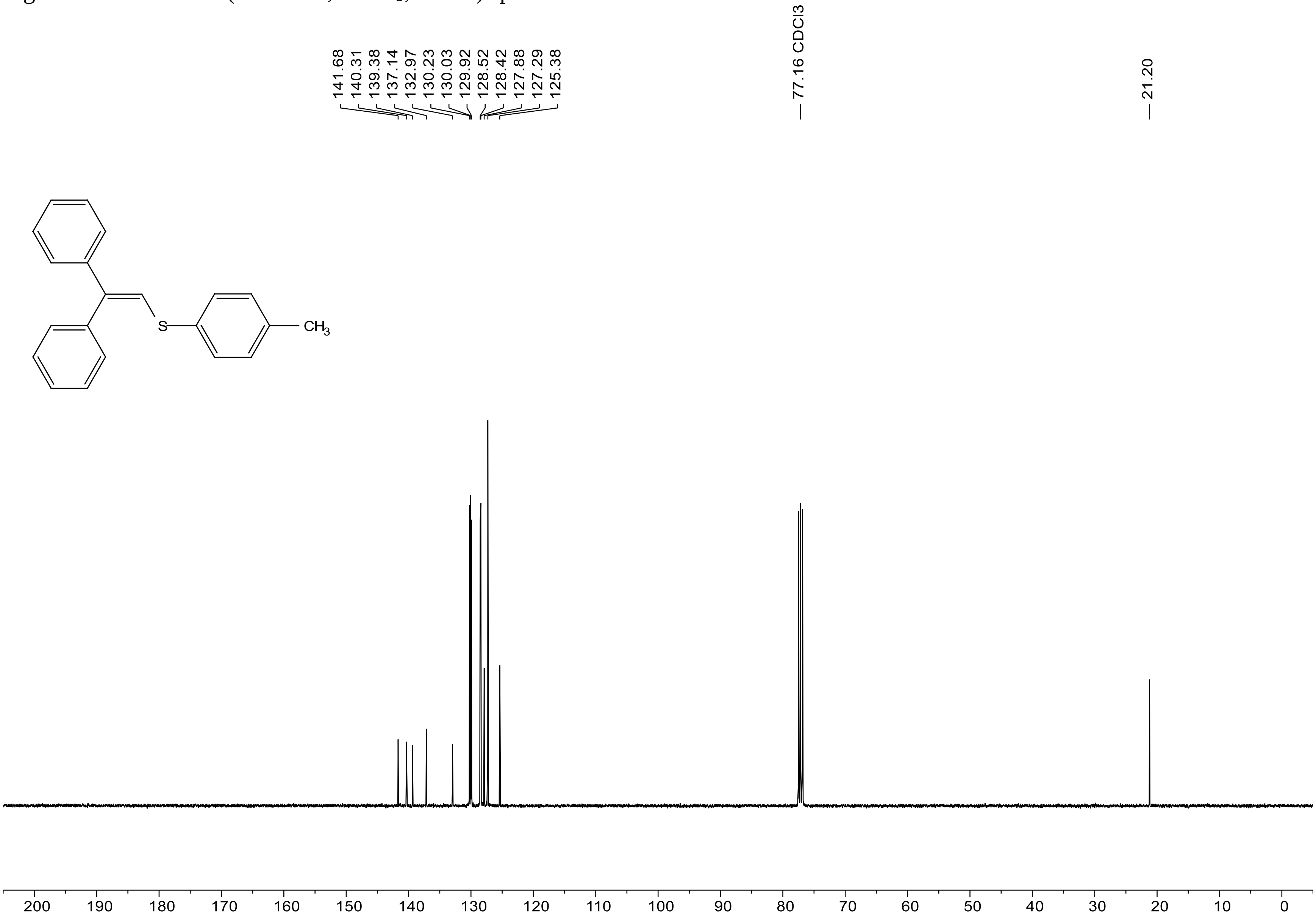

Figure S94: **^{1}H NMR** (400 MHz, $CDCl_3$, 298 K) spectrum of **3ad**.

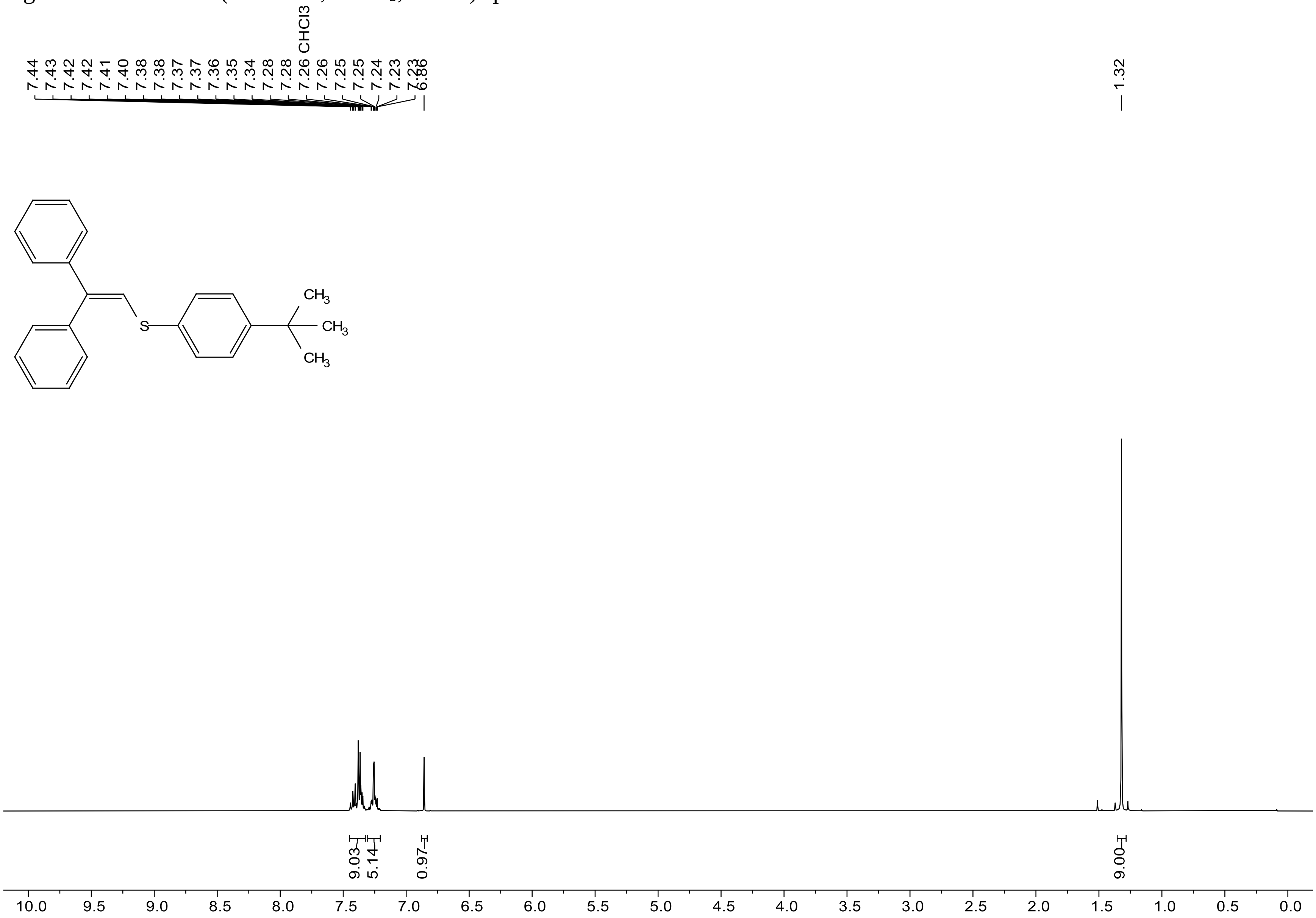

Figure S95: ^{13}**C NMR** (101 MHz, $CDCl_3$, 298 K) spectrum of **3ad**.

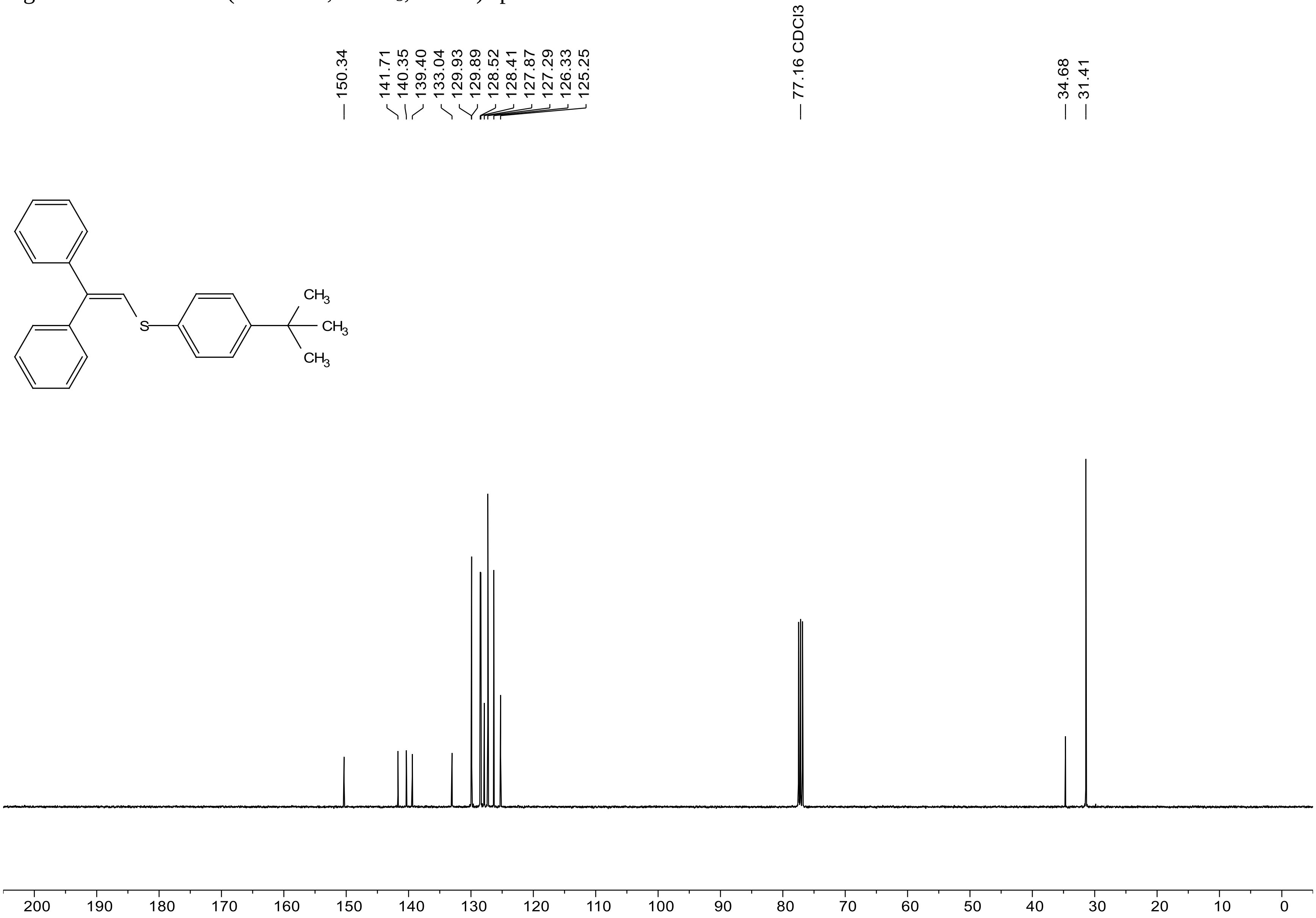

Figure S96: **^{1}H NMR** (400 MHz, $CDCl_3$, 298 K) spectrum of **3ae**.

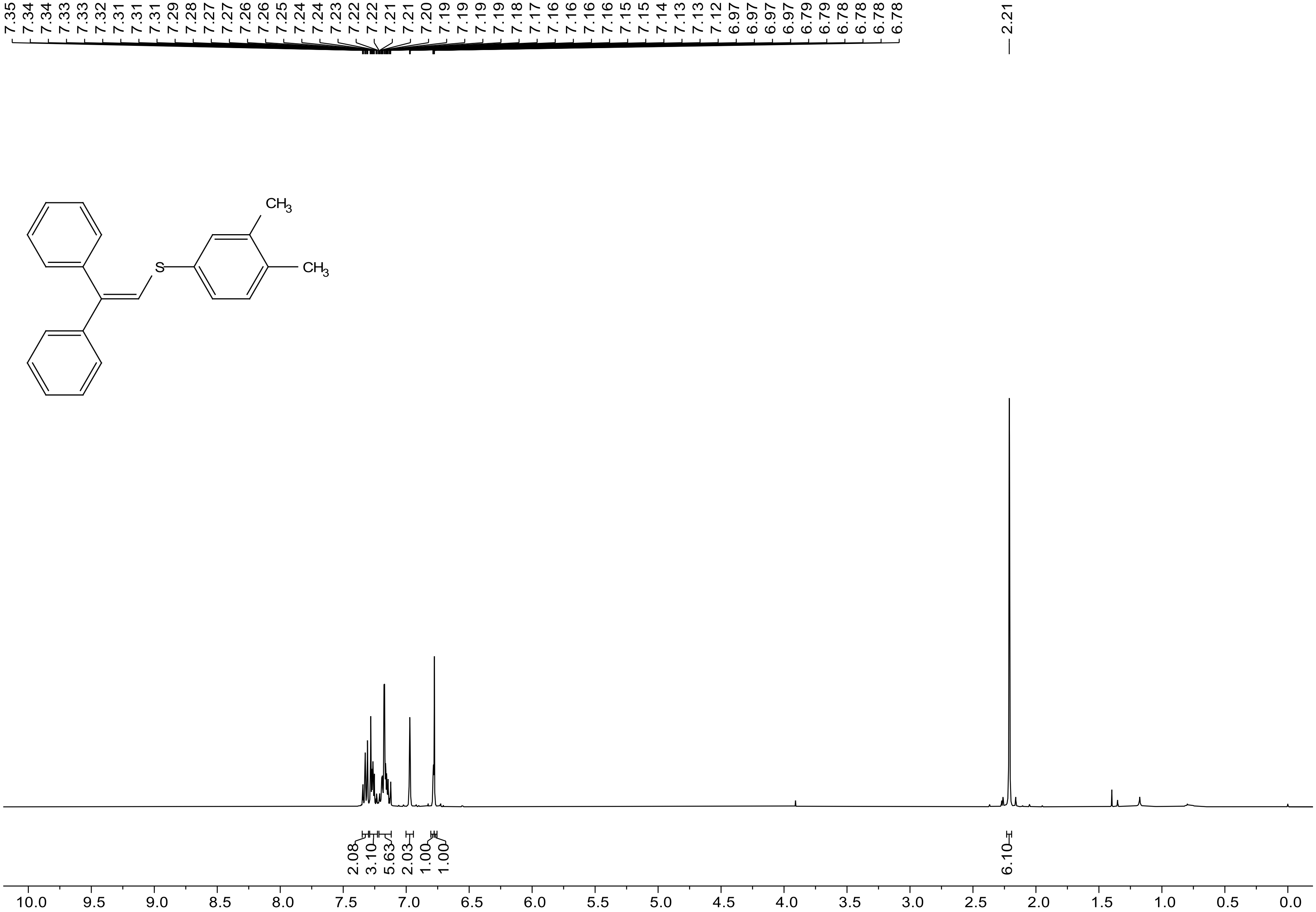

Figure S97: **^{13}C NMR** (101 MHz, $CDCl_3$, 298 K) spectrum of **3ae**.

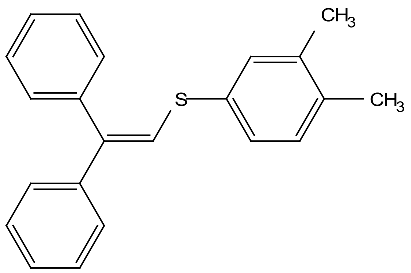

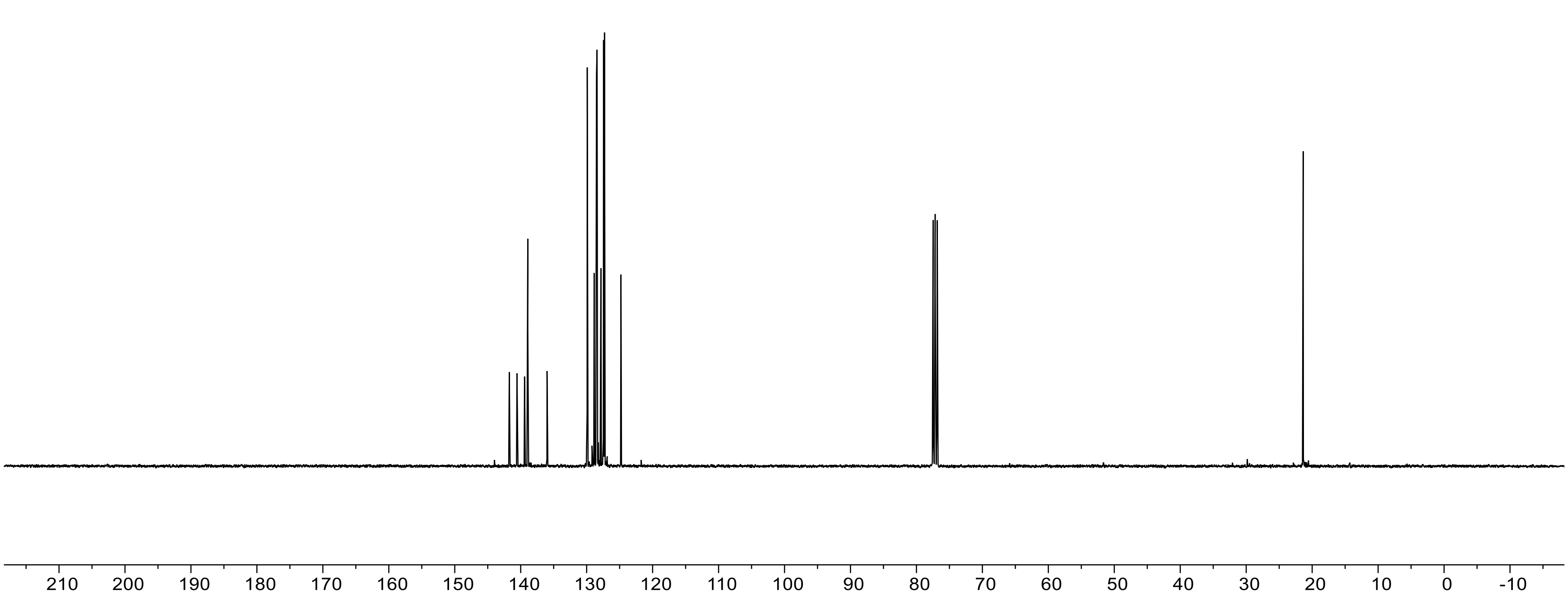

Figure S98: **^{1}H NMR** (400 MHz, $CDCl_3$, 298 K) spectrum of **3af**.

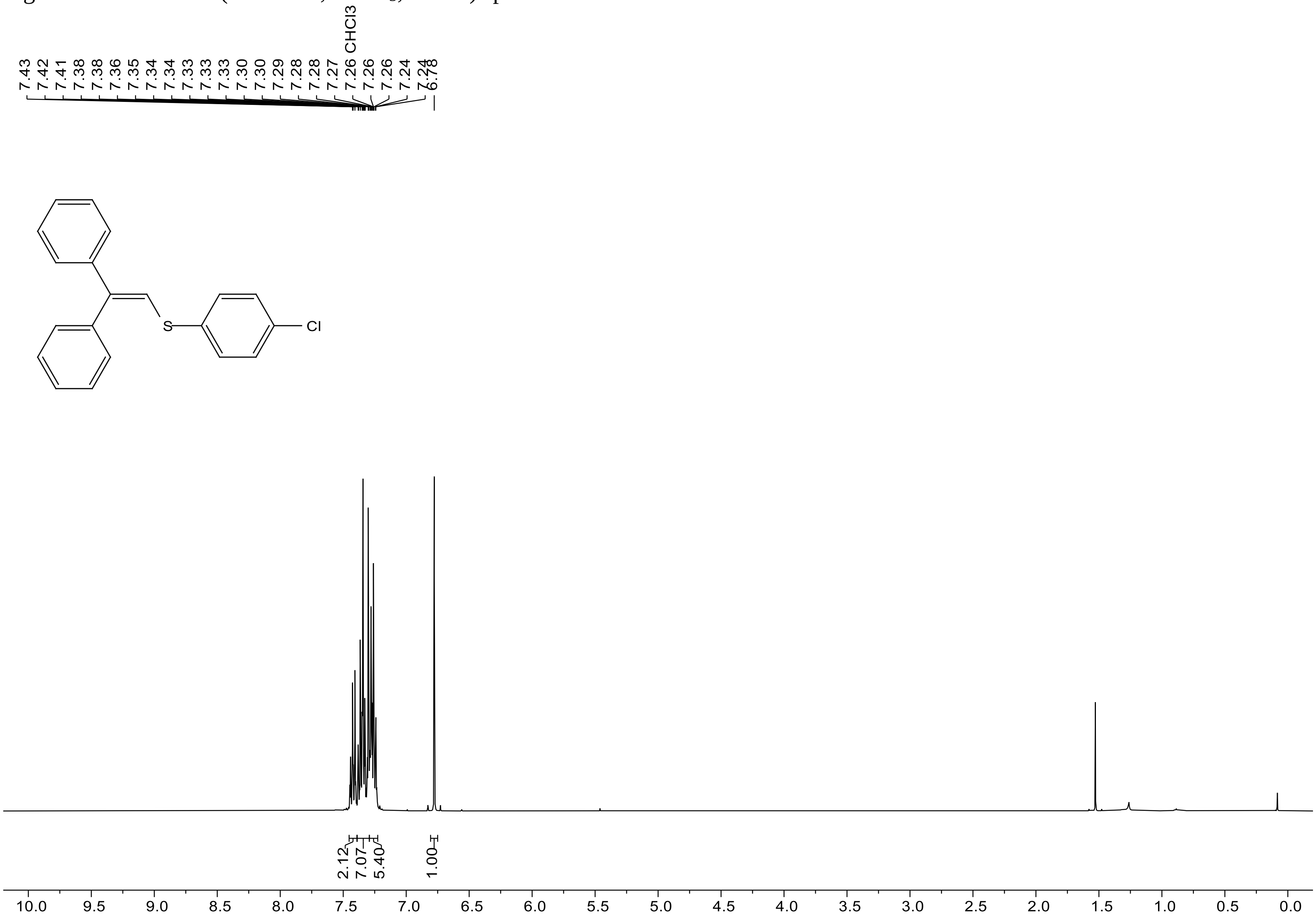

Figure S99: ^{13}**C NMR** (101 MHz, $CDCl_3$, 298 K) spectrum of **3af**.

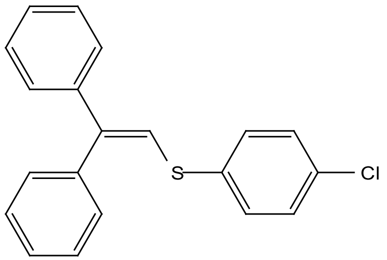

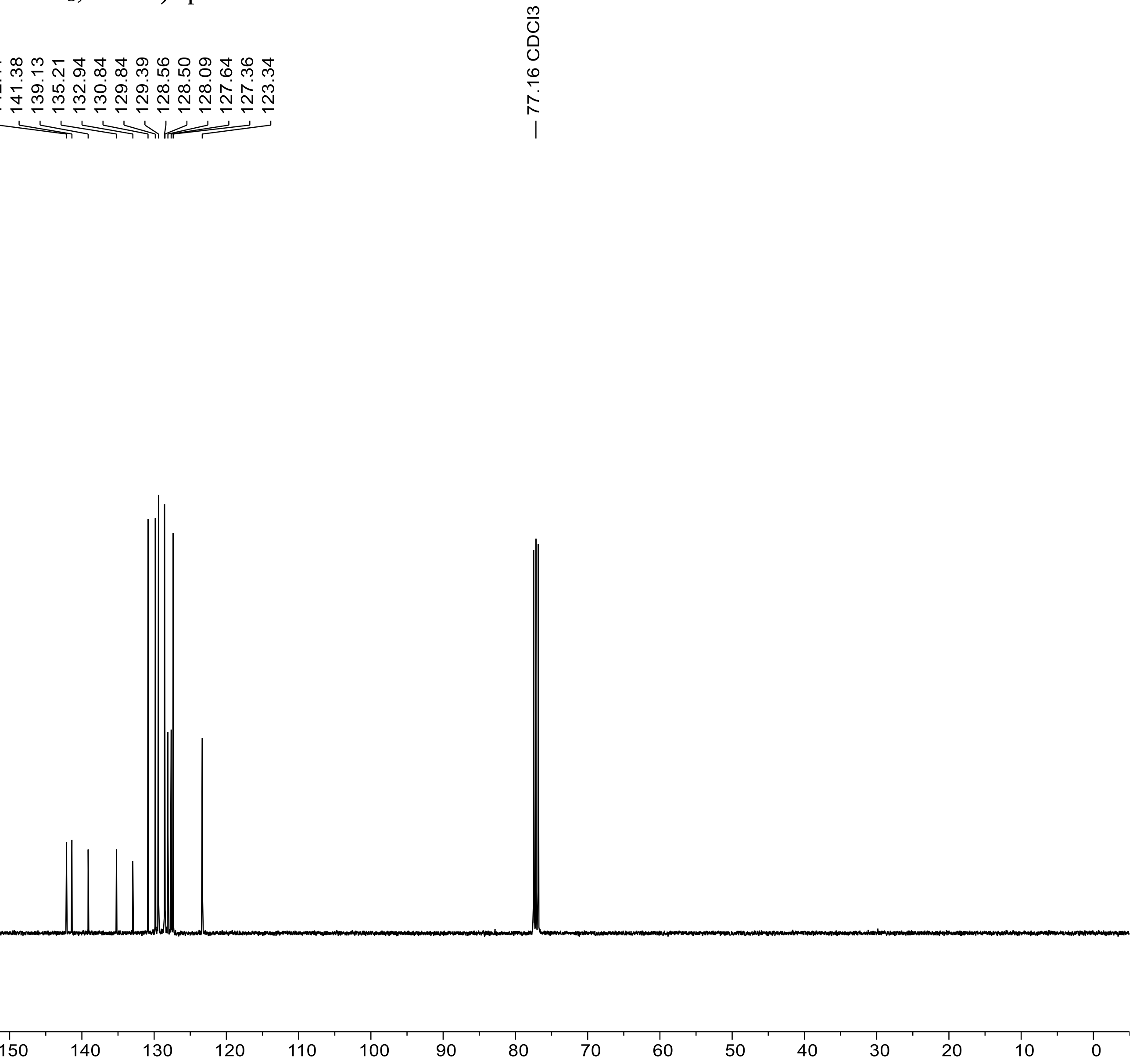

Figure S100: **^{1}H NMR** (400 MHz, $CDCl_3$, 298 K) spectrum of **3ag**.

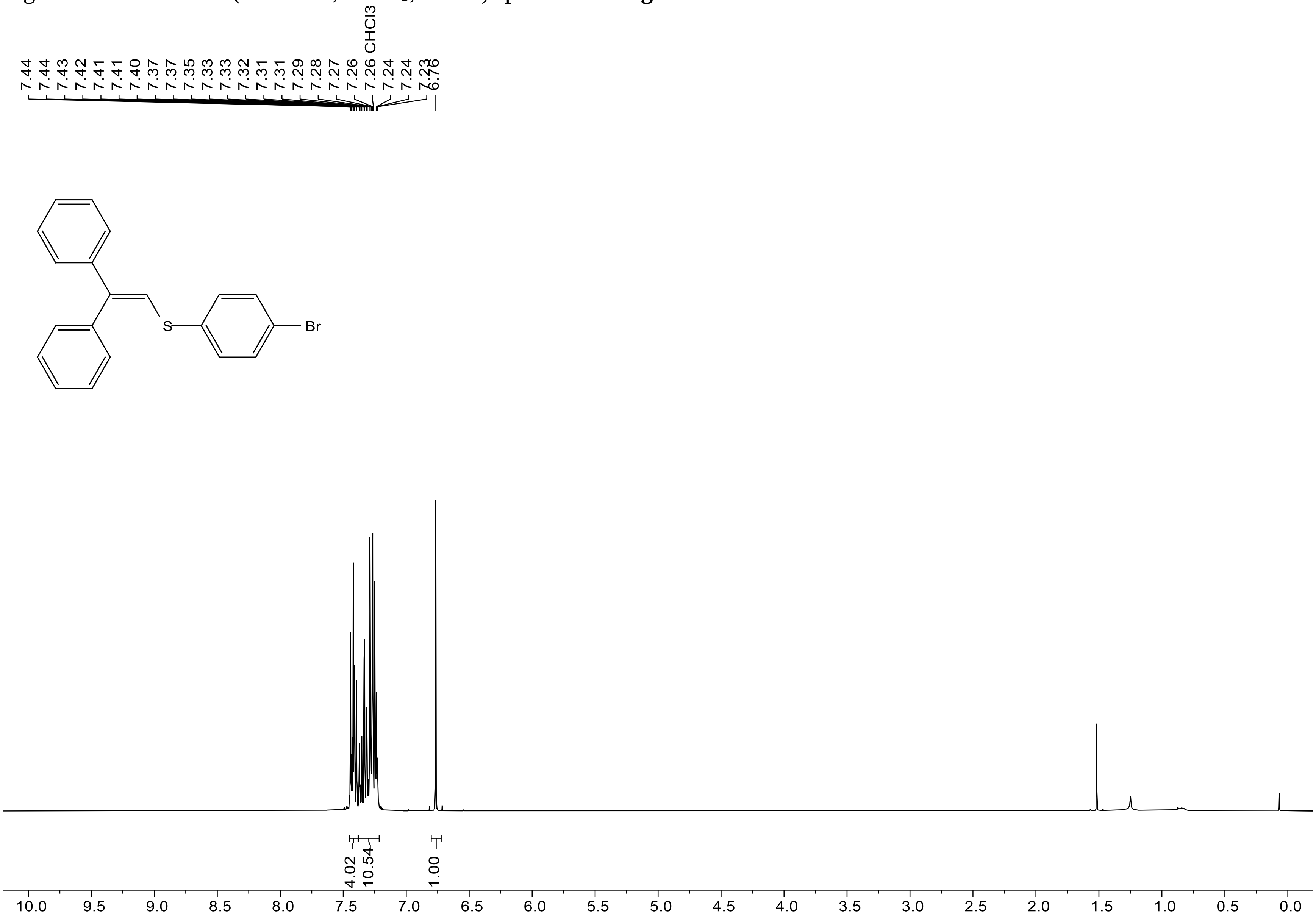

Figure S101: ^{13}**C NMR** (101 MHz, $CDCl_3$, 298 K) spectrum of **3ag**.

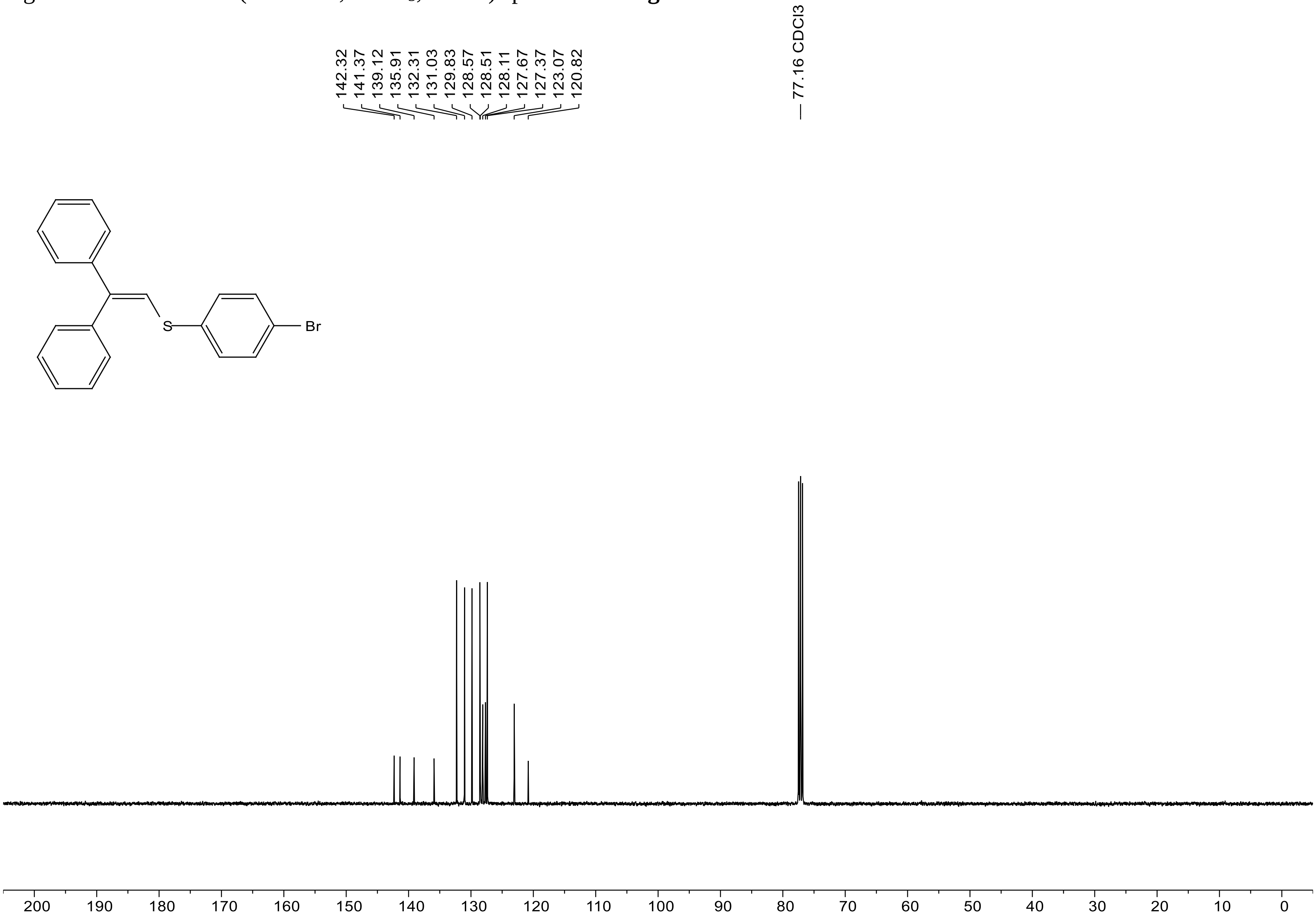

Figure S102: **^{1}H NMR** (400 MHz, $CDCl_3$, 298 K) spectrum of **3ah**.

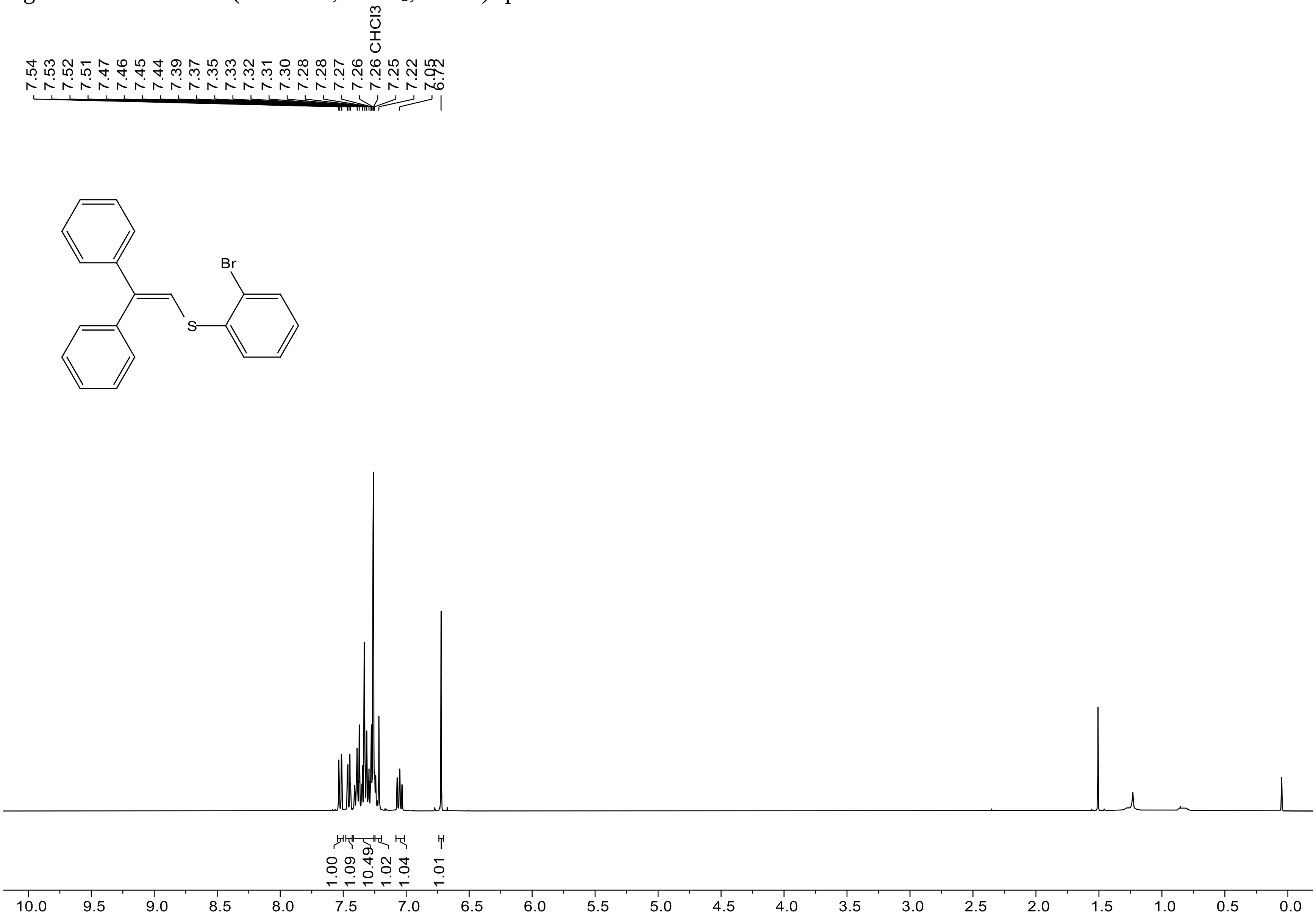

Figure S103: **^{13}C NMR** (101 MHz, $CDCl_3$, 298 K) spectrum of **3ah**.

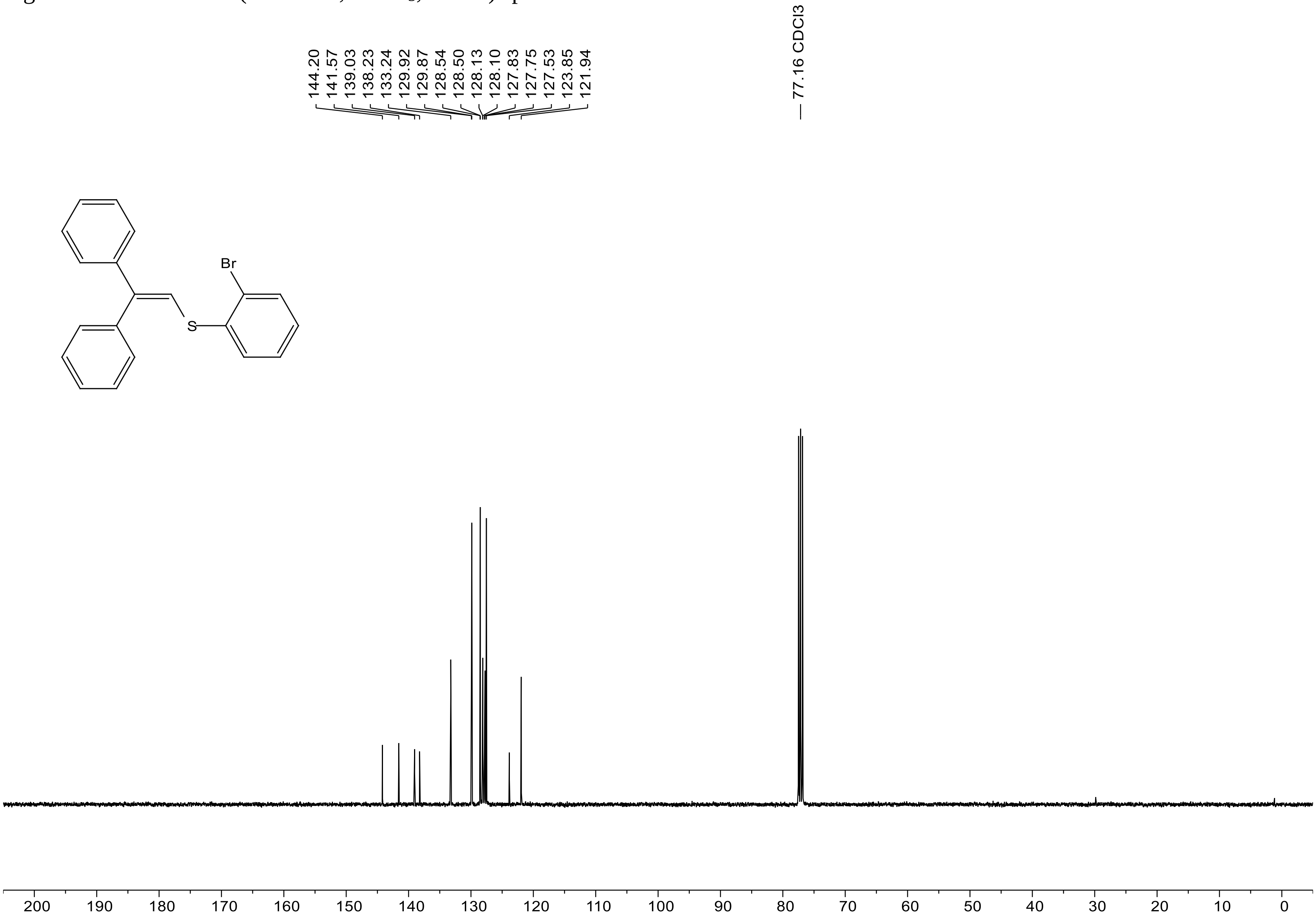

Figure S104: **^{1}H NMR** (400 MHz, $CDCl_3$, 298 K) spectrum of **3ai**.

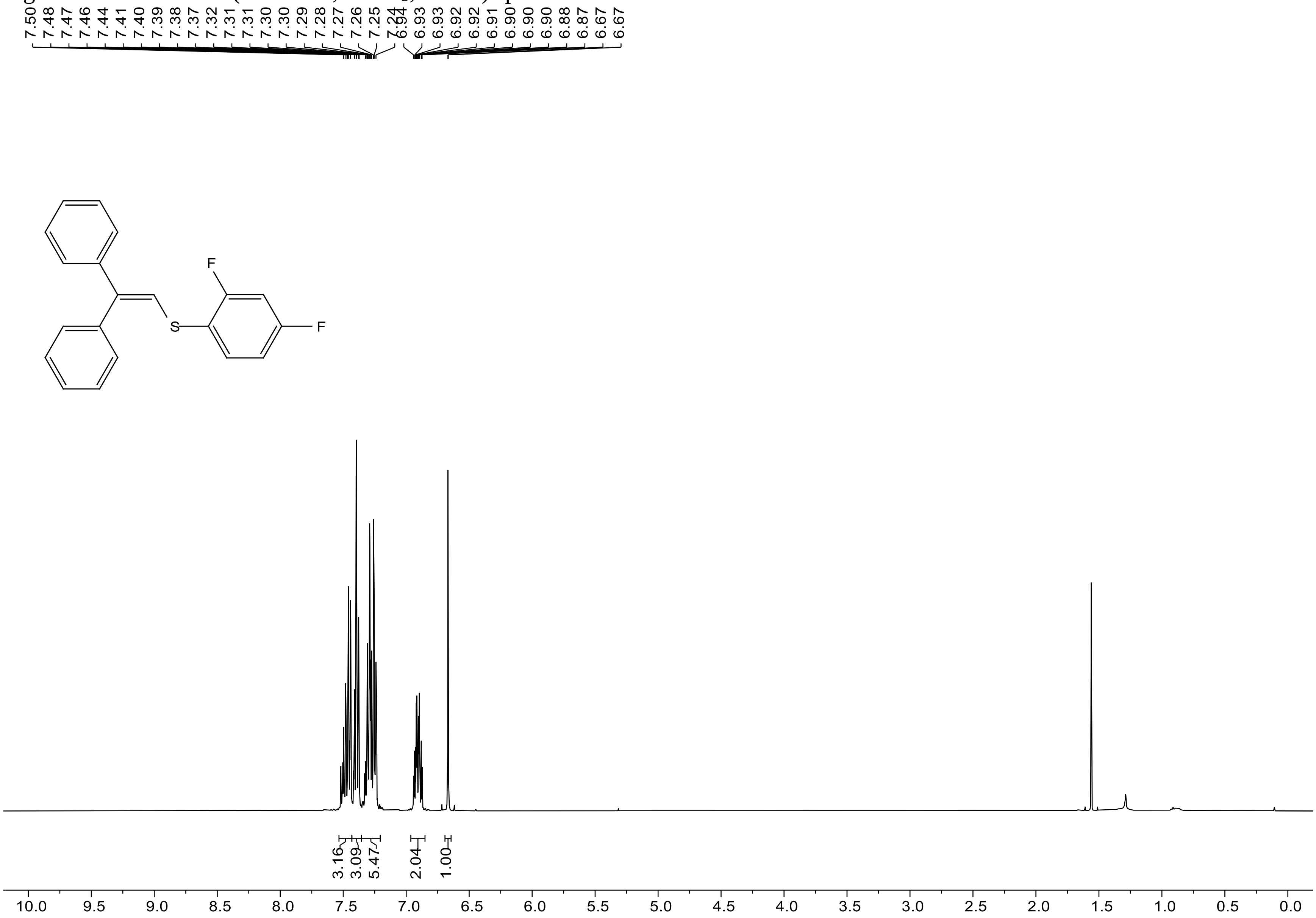

Figure S105: ^{13}**C NMR** (101 MHz, $CDCl_3$, 298 K) spectrum of **3ai**.

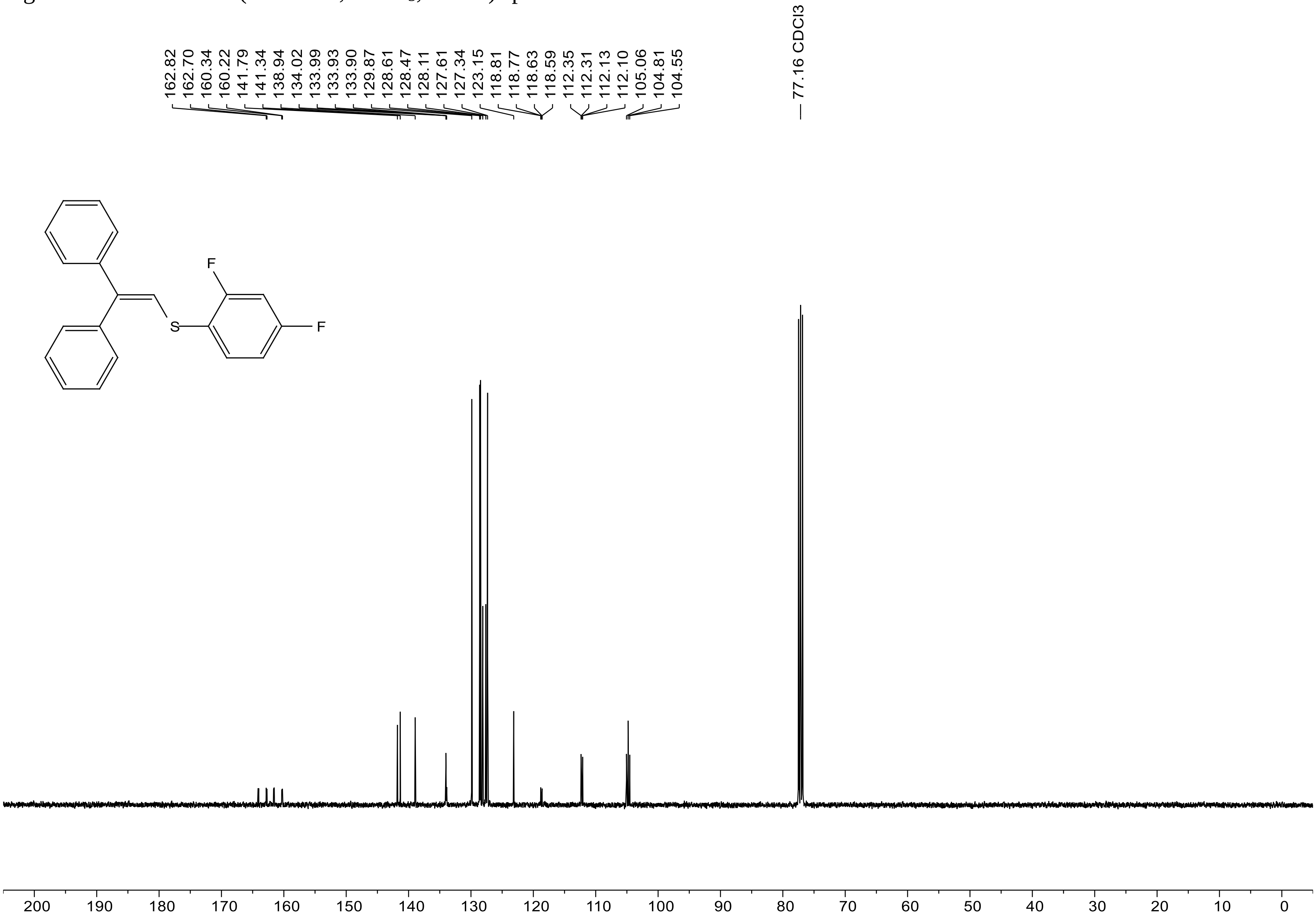

Figure S106: ^{19}**F NMR** (376 MHz, $CDCl_3$, 298 K) spectrum of **3ai**.

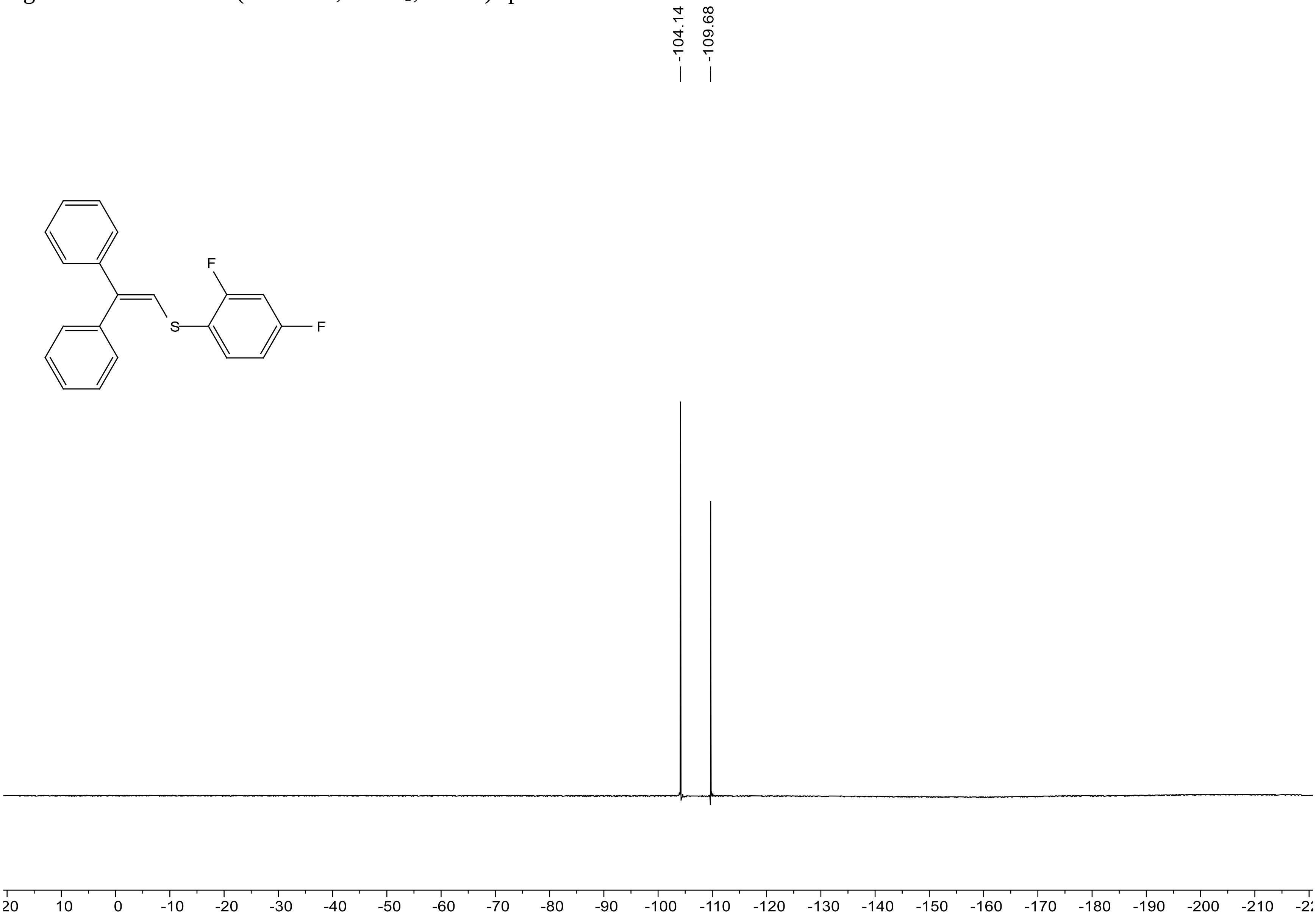

Figure S107: **^{1}H NMR** (400 MHz, $CDCl_3$, 298 K) spectrum of **3aj**.

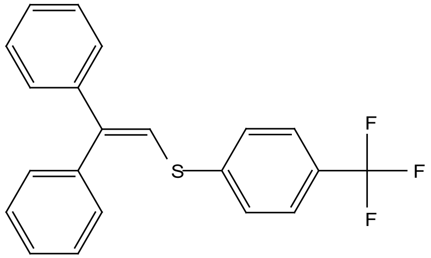

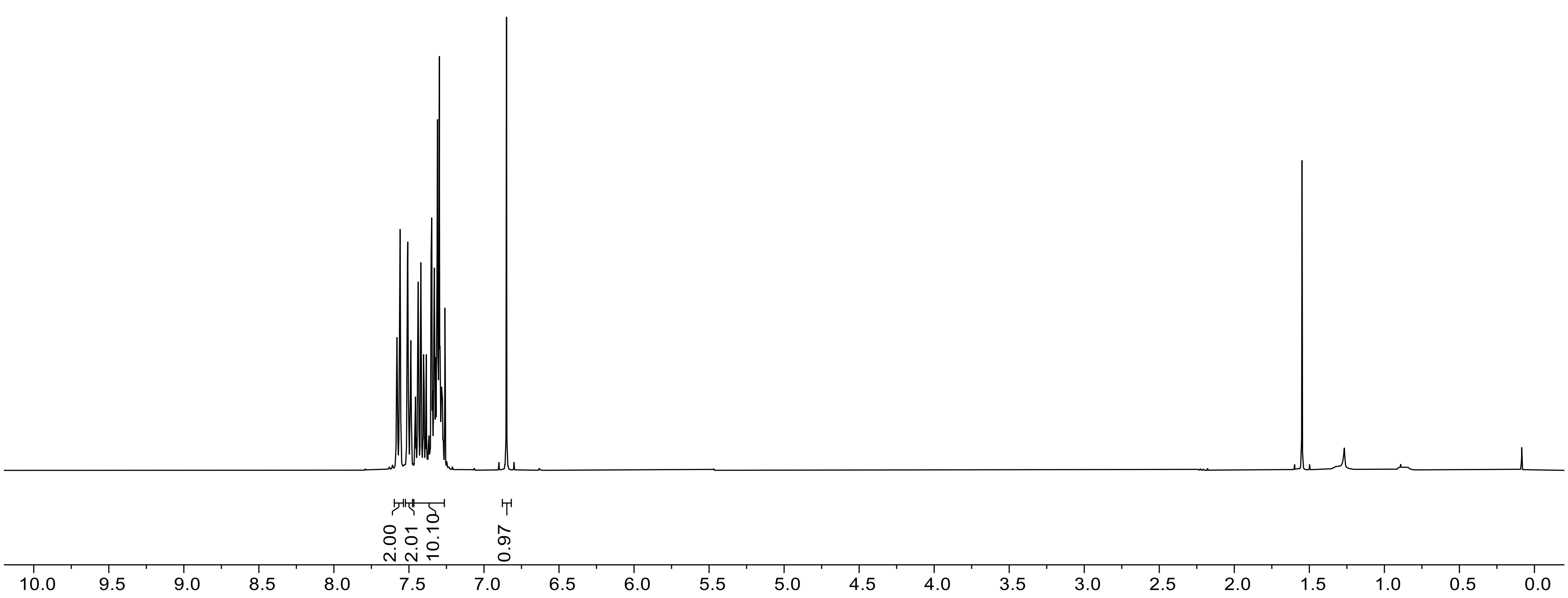

Figure S108: **^{13}C NMR** (101 MHz, $CDCl_3$, 298 K) spectrum of **3aj**.

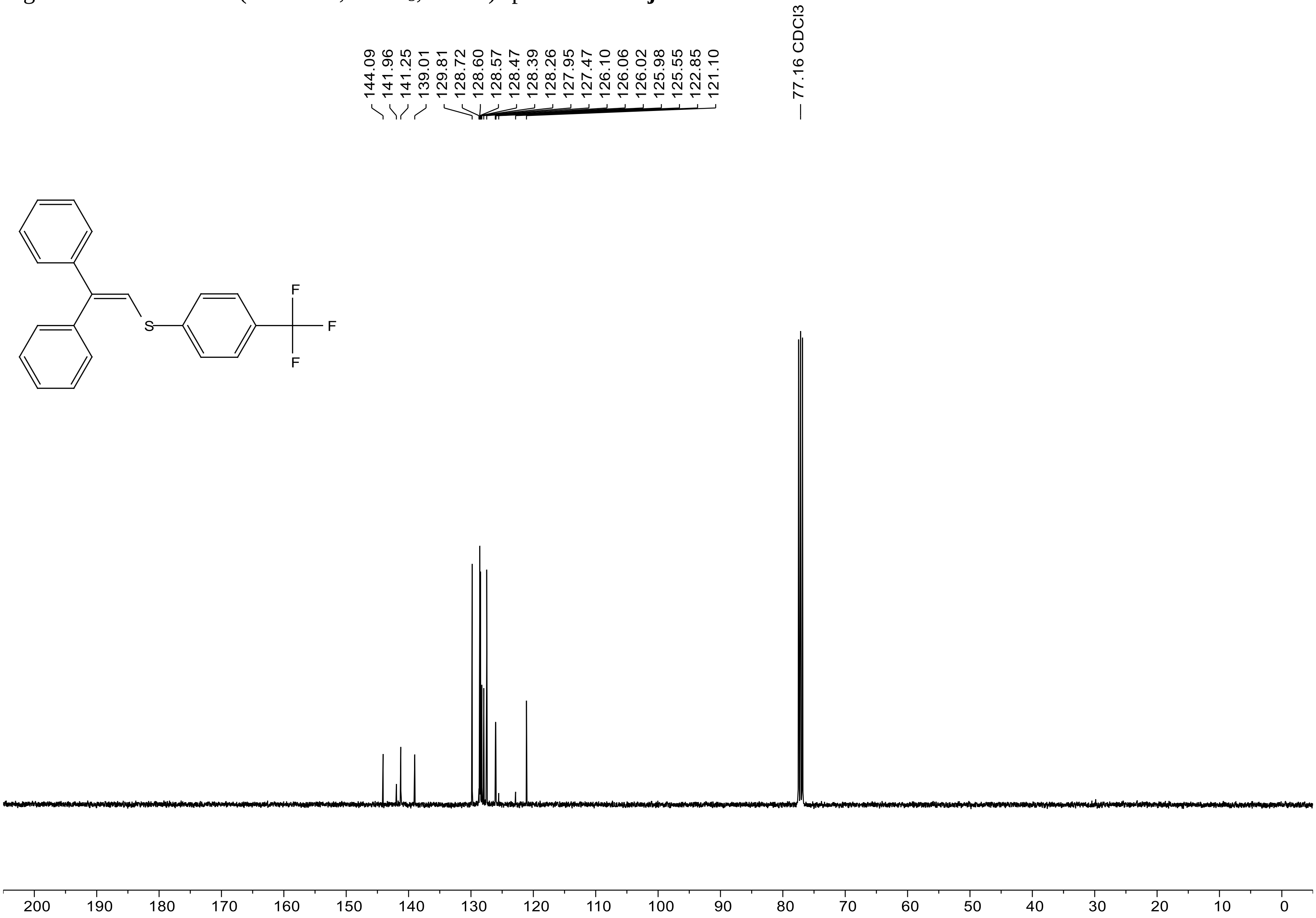

Figure S109: ^{19}**F NMR** (376 MHz, $CDCl_3$, 298 K) spectrum of **3aj**.

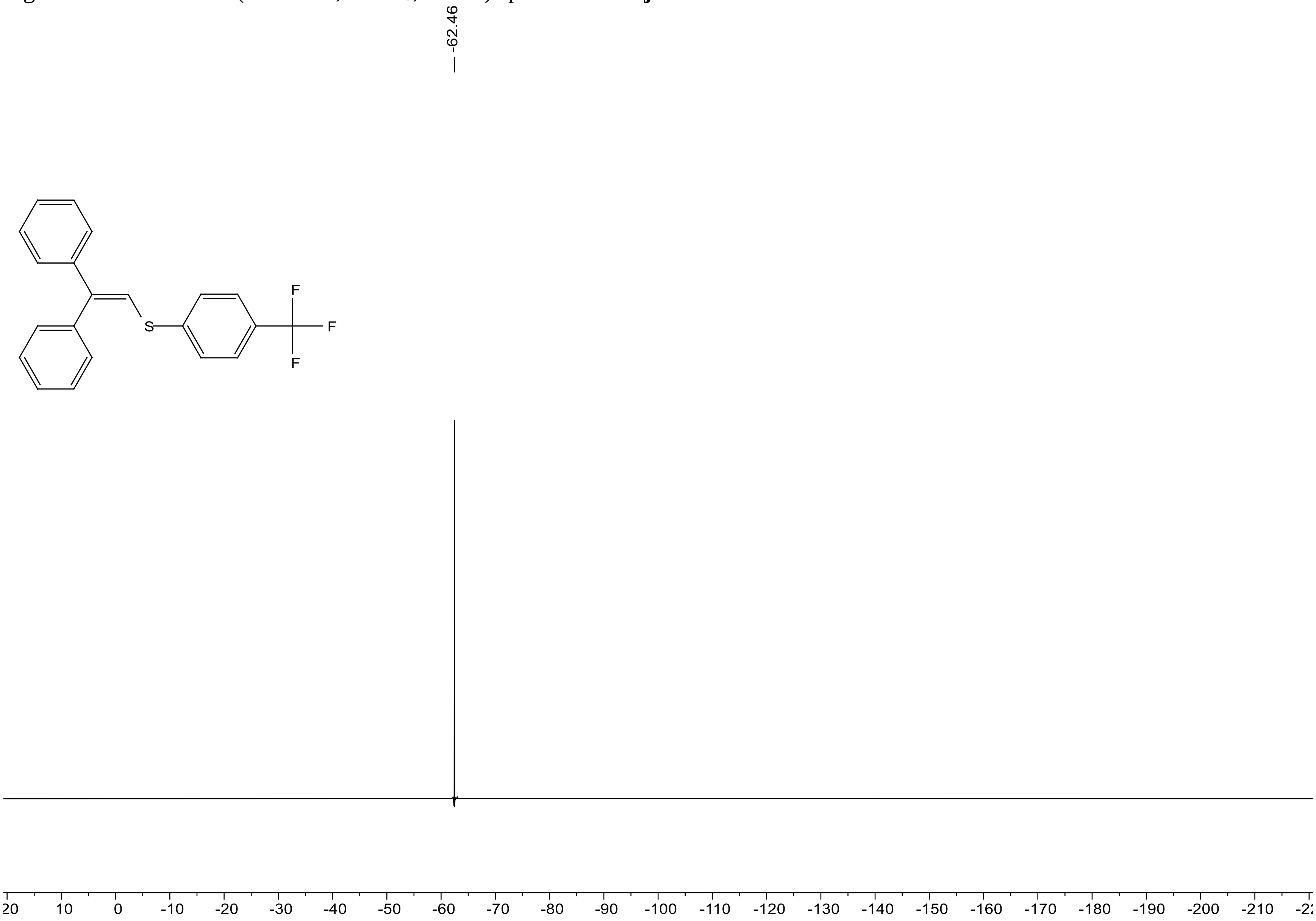

Figure S110: **^{1}H NMR** (400 MHz, $CDCl_3$, 298 K) spectrum of **3ak**.

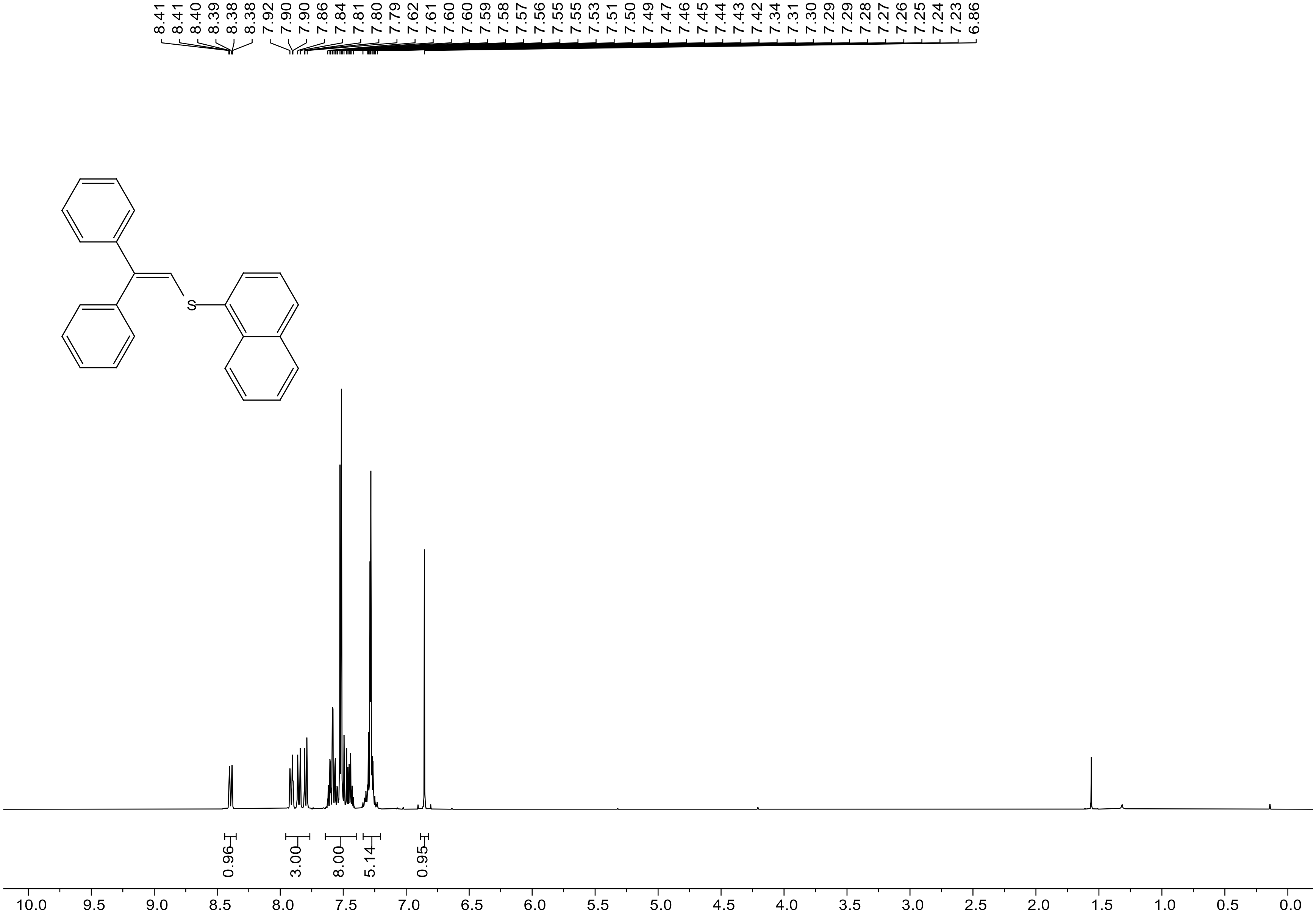

Figure S111: ^{13}**C NMR** (101 MHz, $CDCl_3$, 298 K) spectrum of **3ak**.

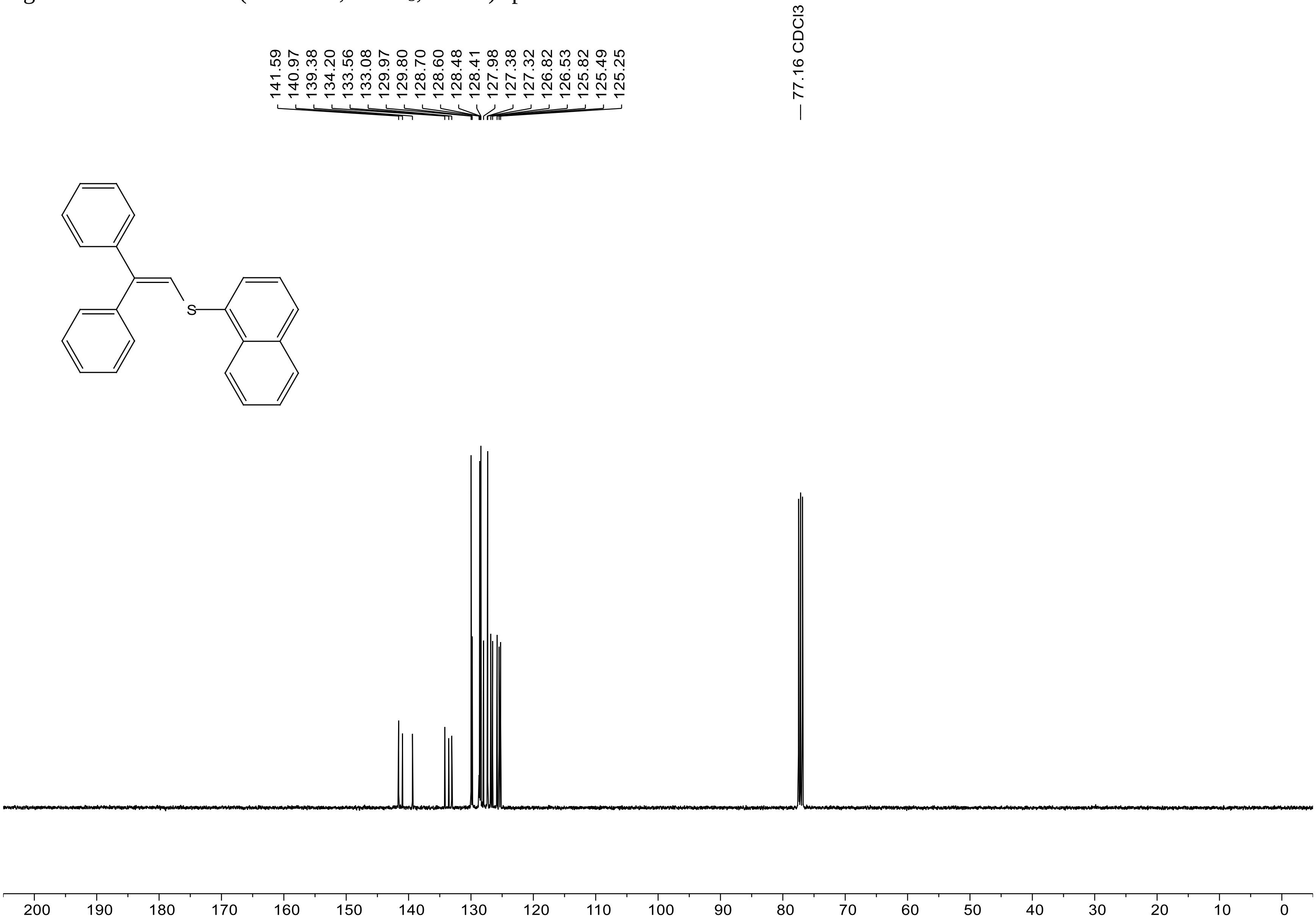

Figure S112: **^{1}H NMR** (400 MHz, $CDCl_3$, 298 K) spectrum of **3al**.

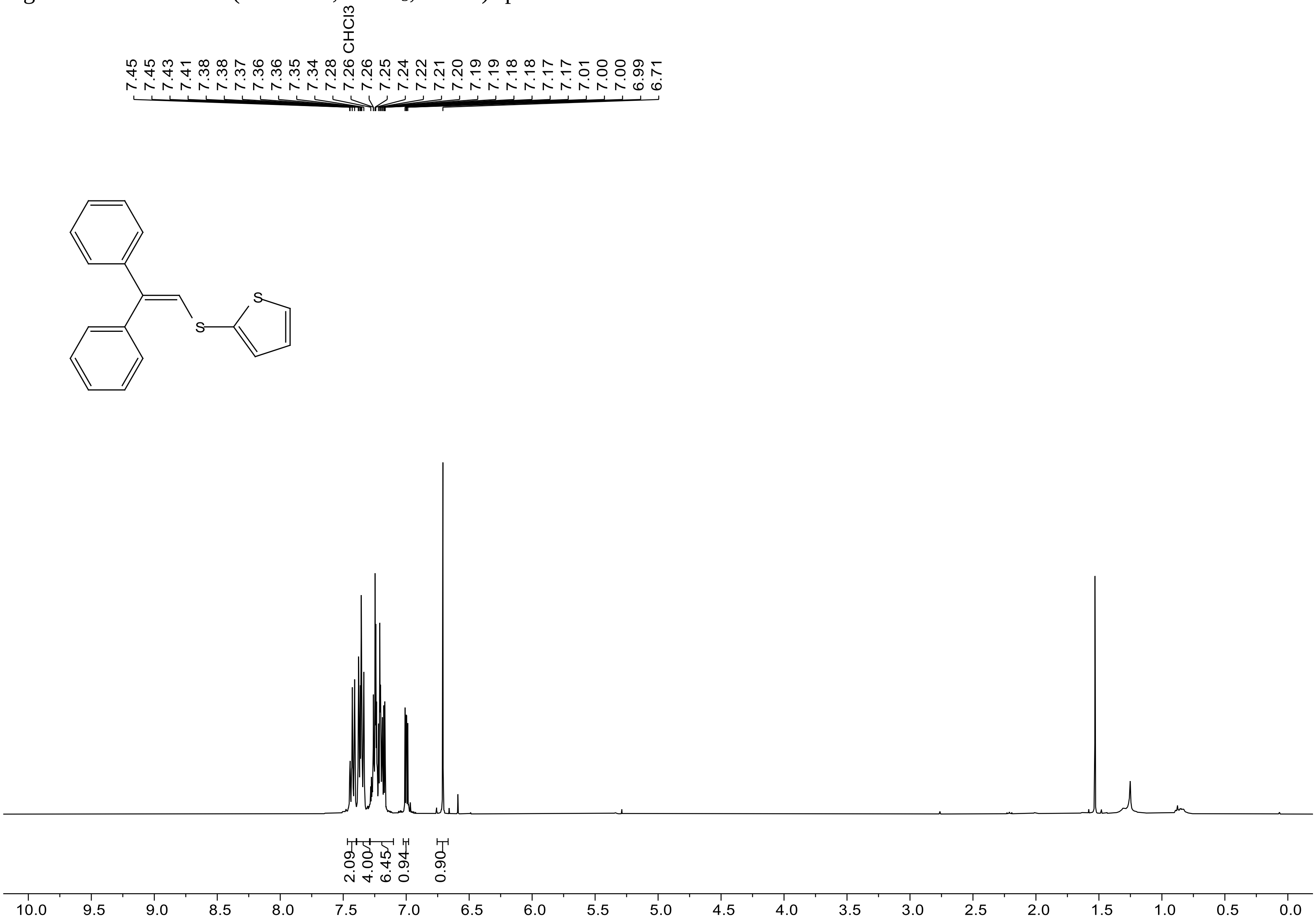

Figure S113: **^{13}C NMR** (101 MHz, $CDCl_3$, 298 K) spectrum of **3al**.

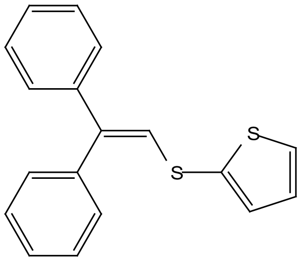

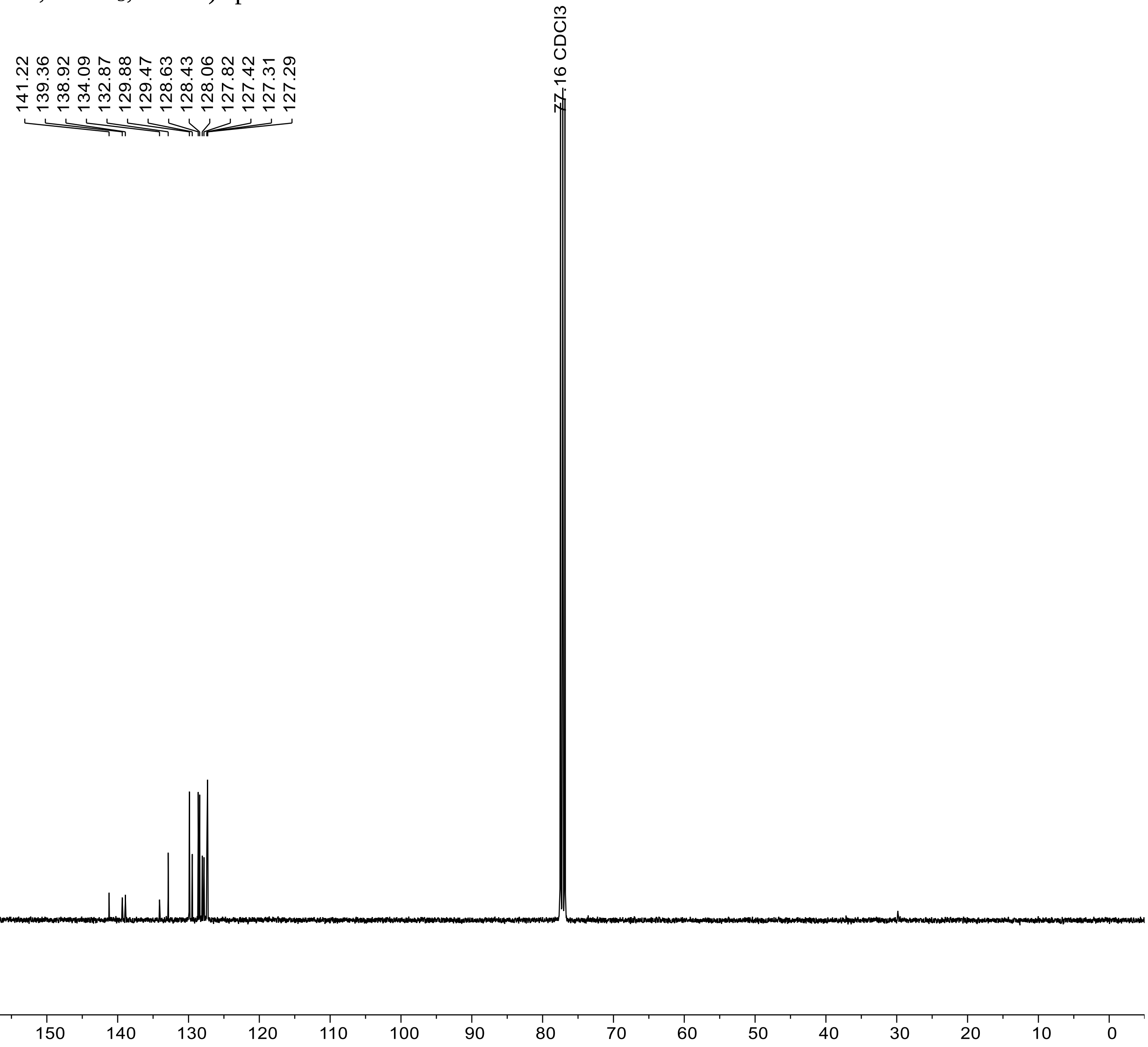

Figure S114: **^{1}H NMR** (400 MHz, $CDCl_3$, 298 K) spectrum of **3am**.

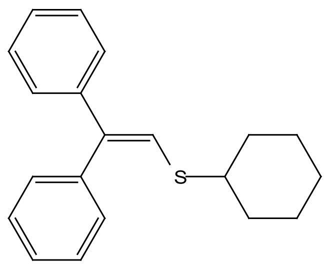

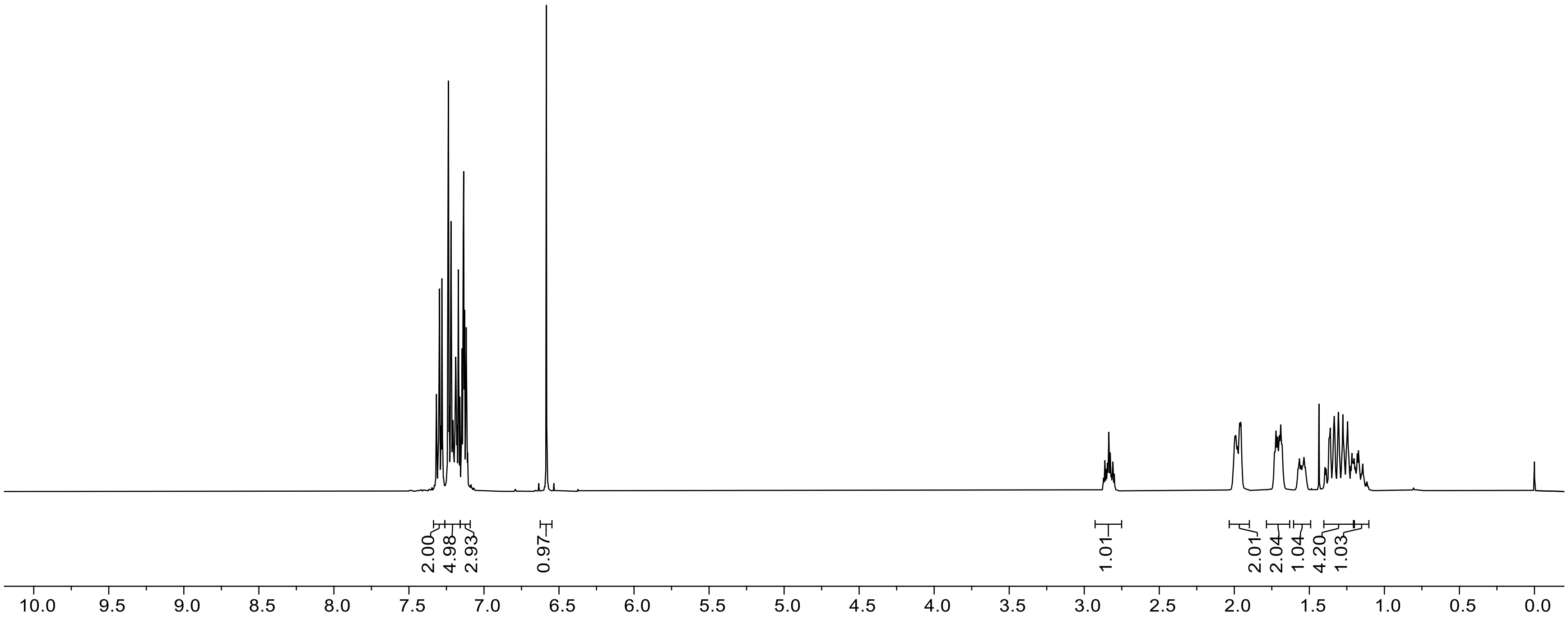

Figure S115: **^{13}C NMR** (101 MHz, $CDCl_3$, 298 K) spectrum of **3am**.

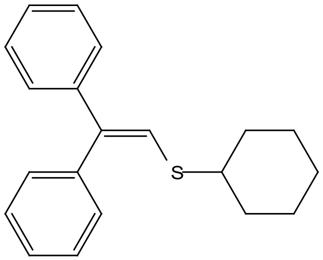

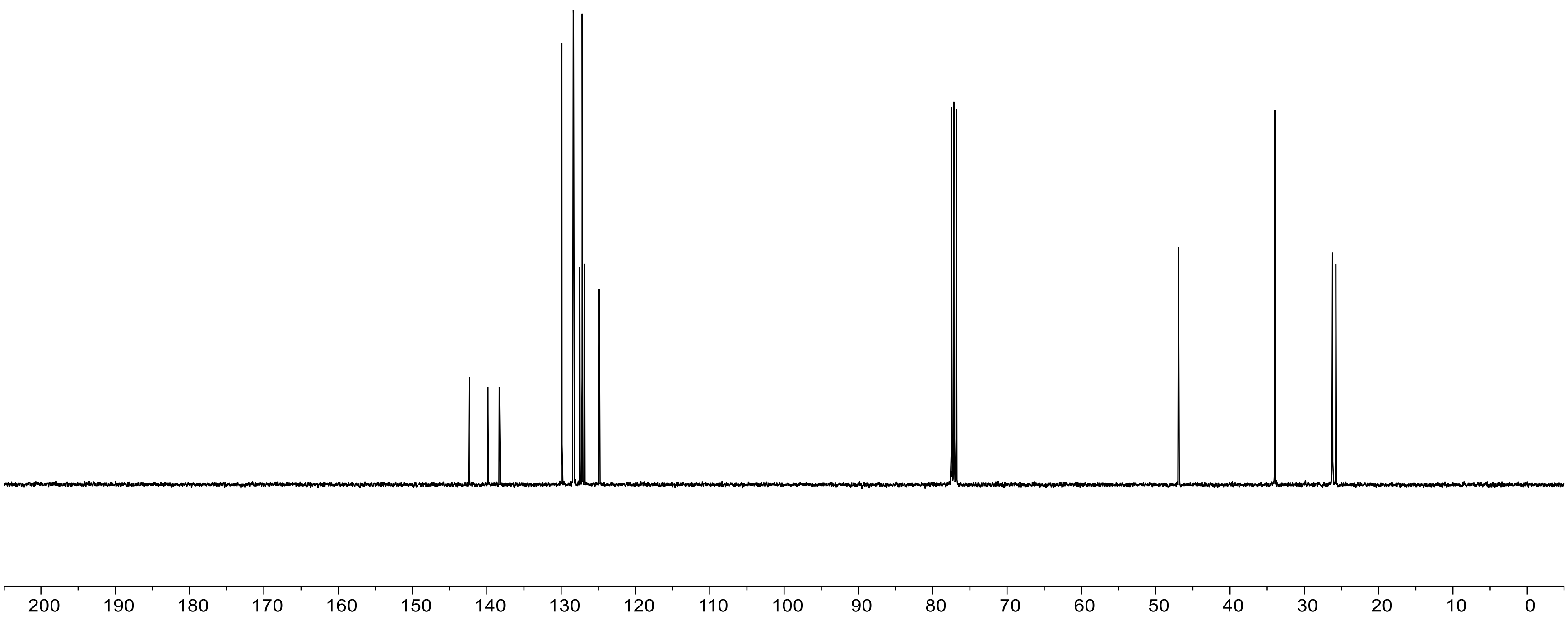

Figure S116: **^{1}H NMR** (400 MHz, $CDCl_3$, 298 K) spectrum of **4pa**.

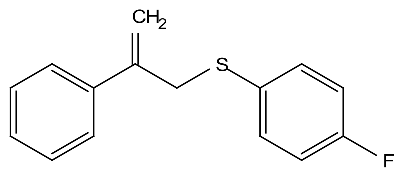

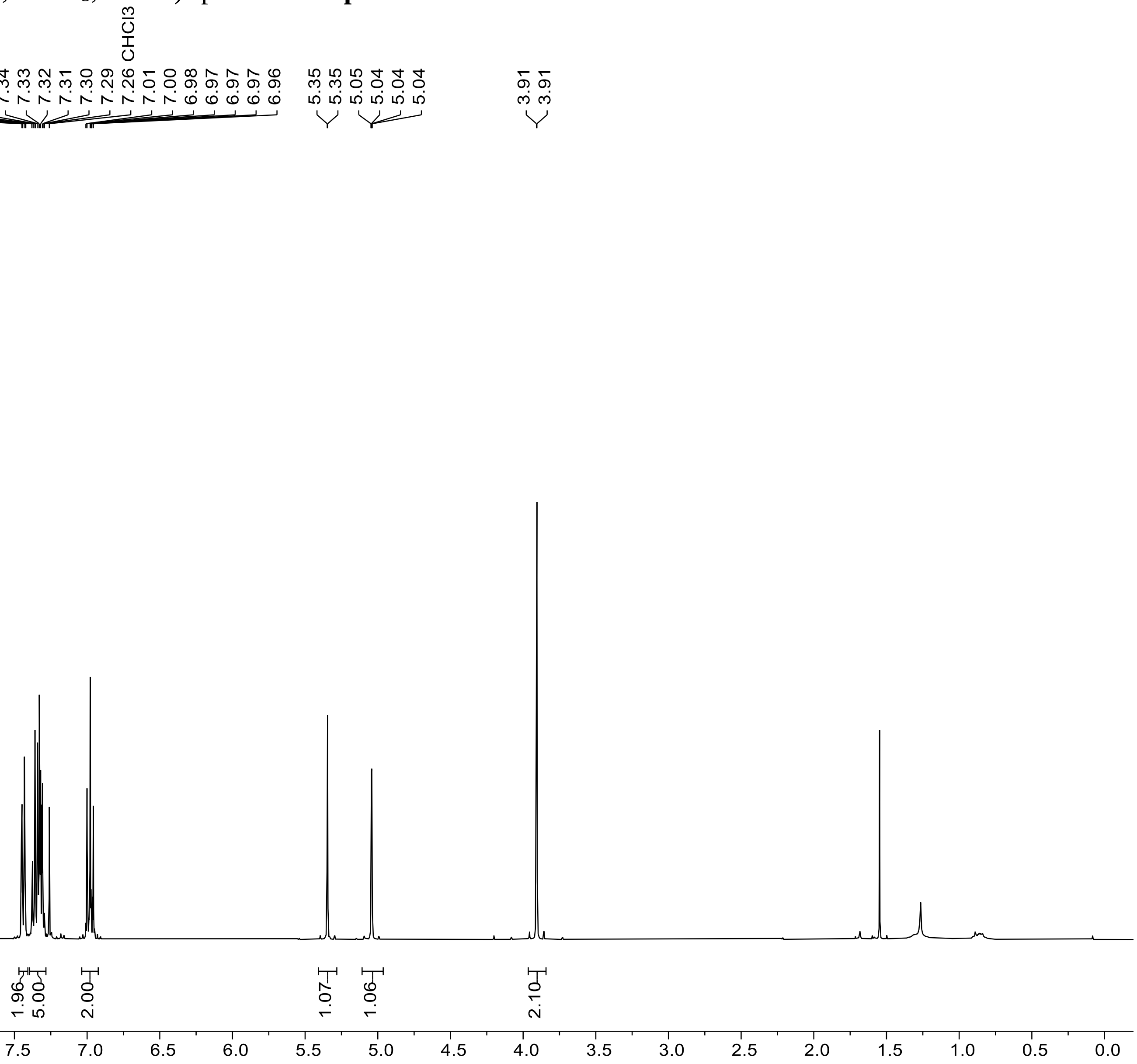

Figure S117: **^{13}C NMR** (101 MHz, $CDCl_3$, 298 K) spectrum of **4pa**.

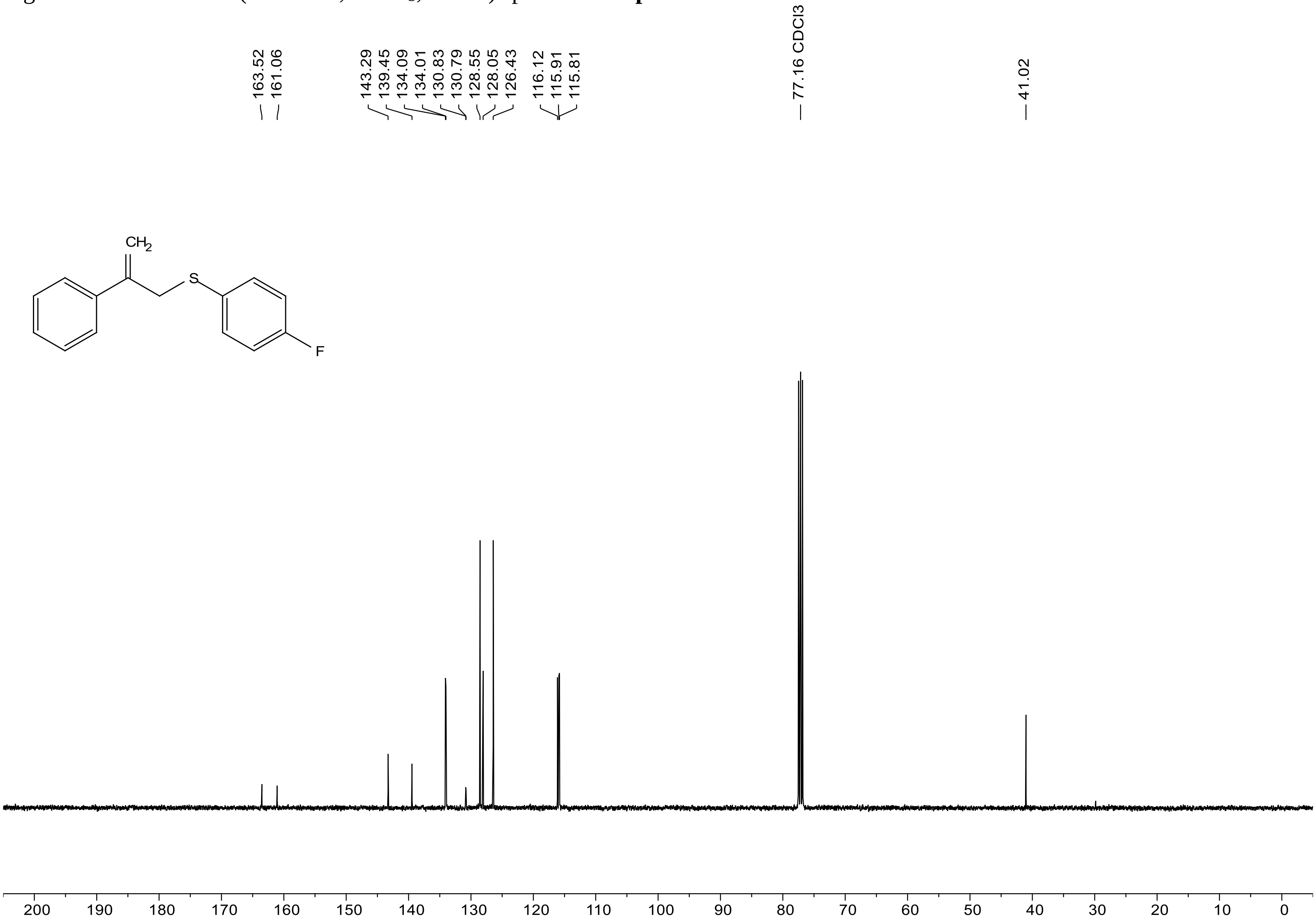

Figure S118: ^{19}**F NMR** (376 MHz, $CDCl_3$, 298 K) spectrum of **4pa**.

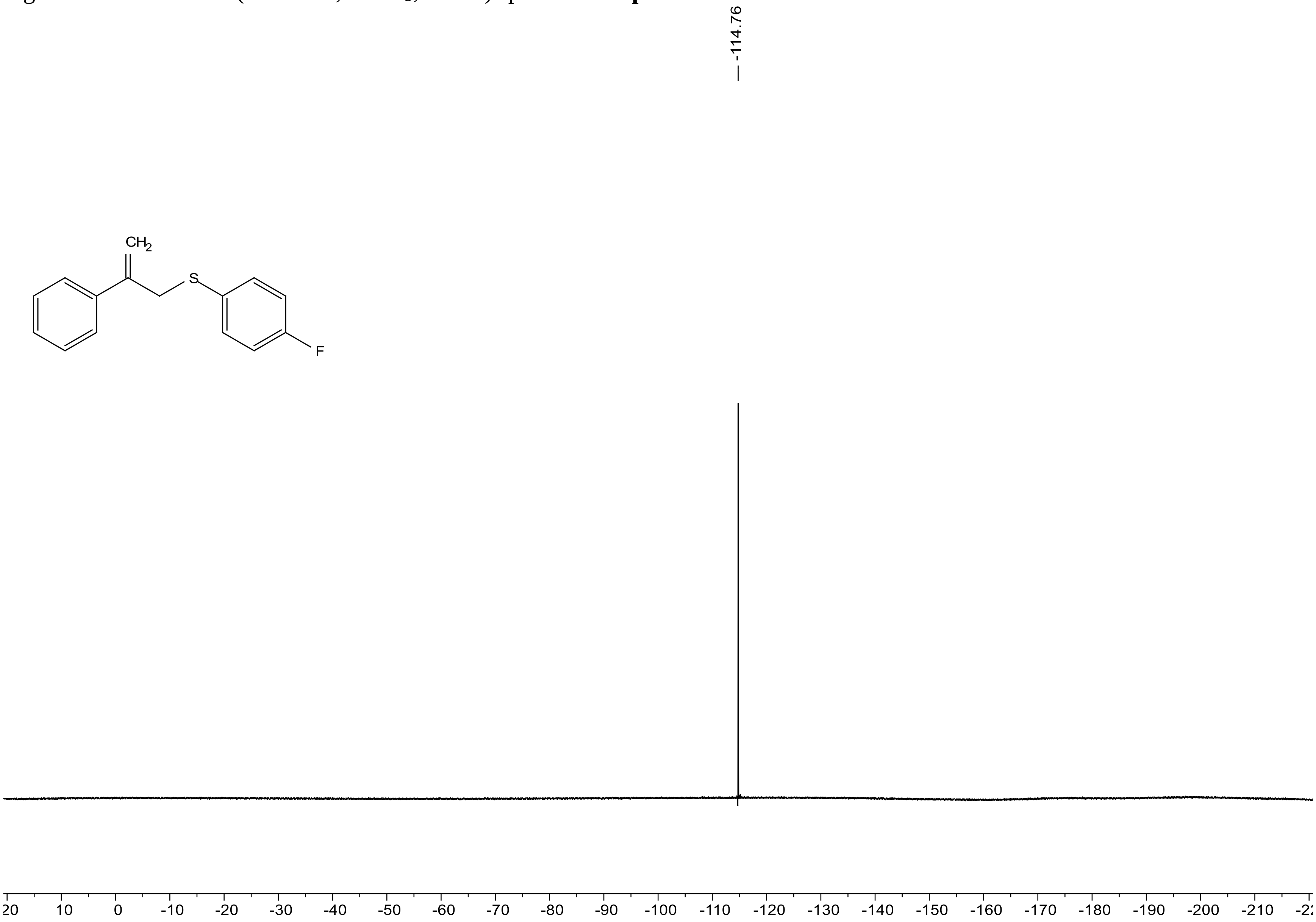

Figure S119: **^{1}H NMR** (400 MHz, $CDCl_3$, 298 K) spectrum of **4qa**.

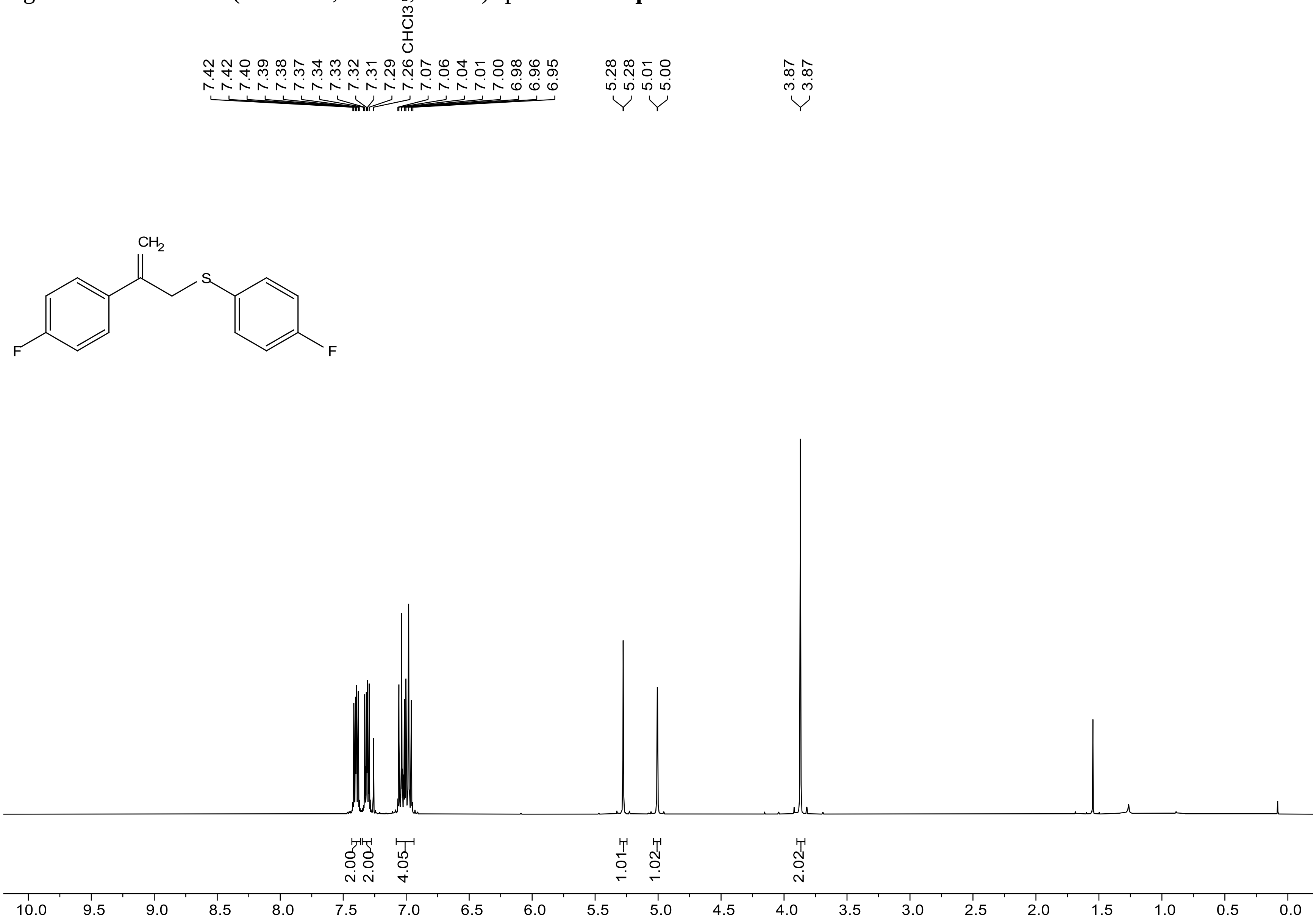

Figure S120: **^{13}C NMR** (101 MHz, $CDCl_3$, 298 K) spectrum of **4qa**.

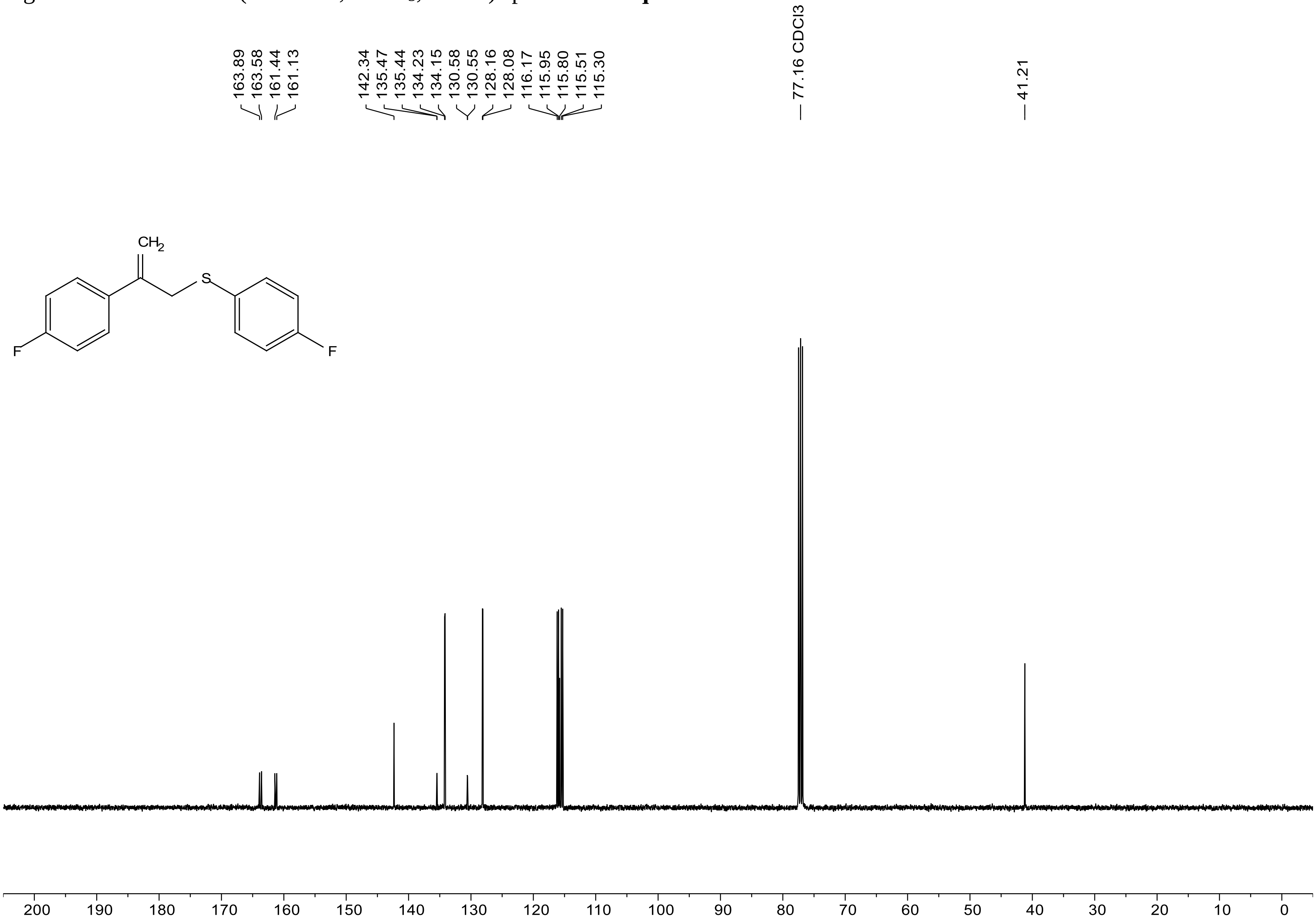

Figure S121: ^{19}**F NMR** (376 MHz, $CDCl_3$, 298 K) spectrum of **4qa**.

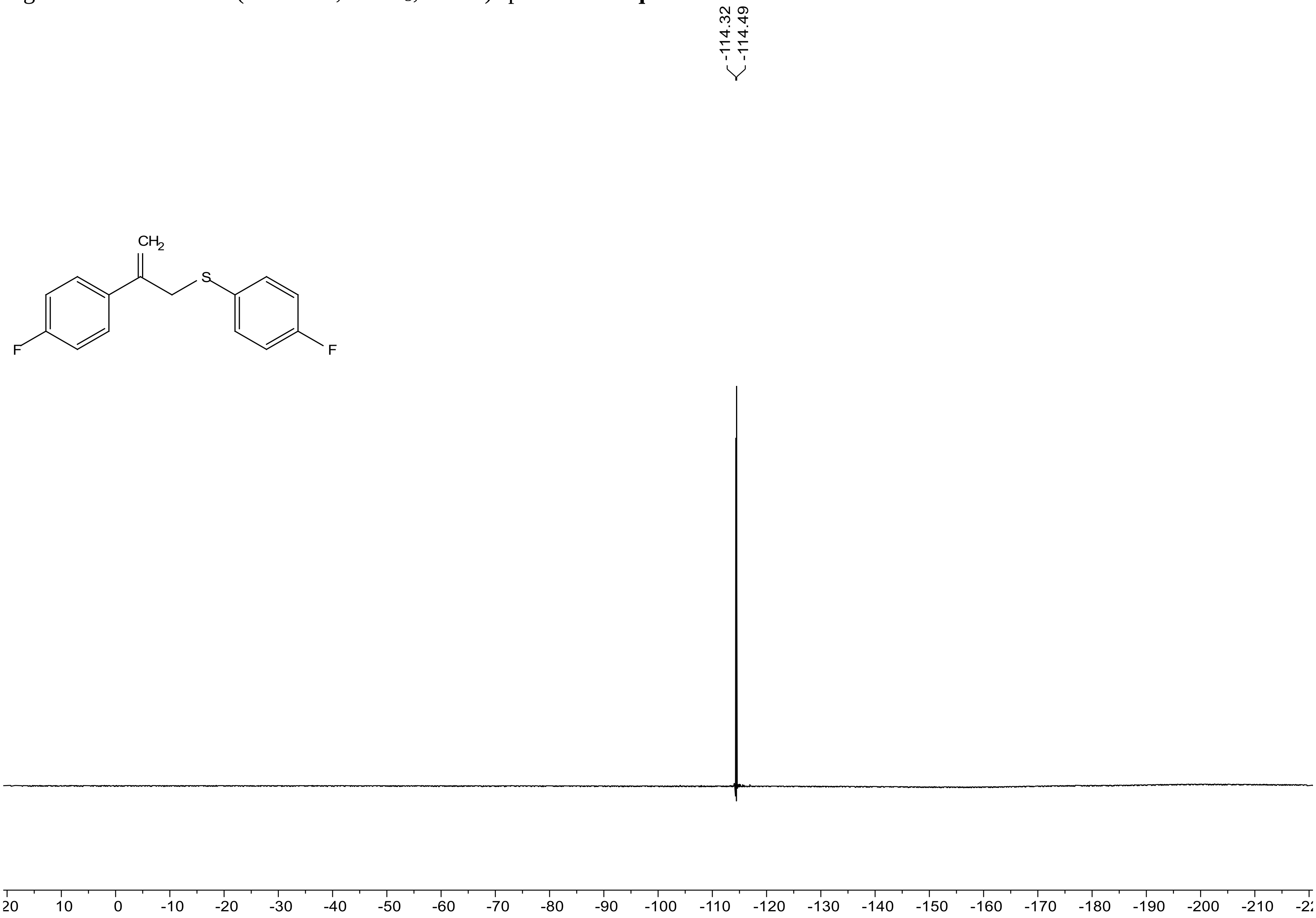

Figure S122: **^{1}H NMR** (400 MHz, $CDCl_3$, 298 K) spectrum of **4ra**.

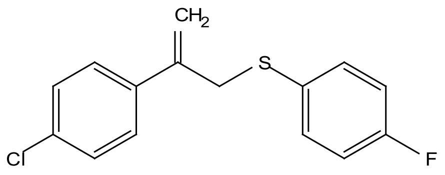

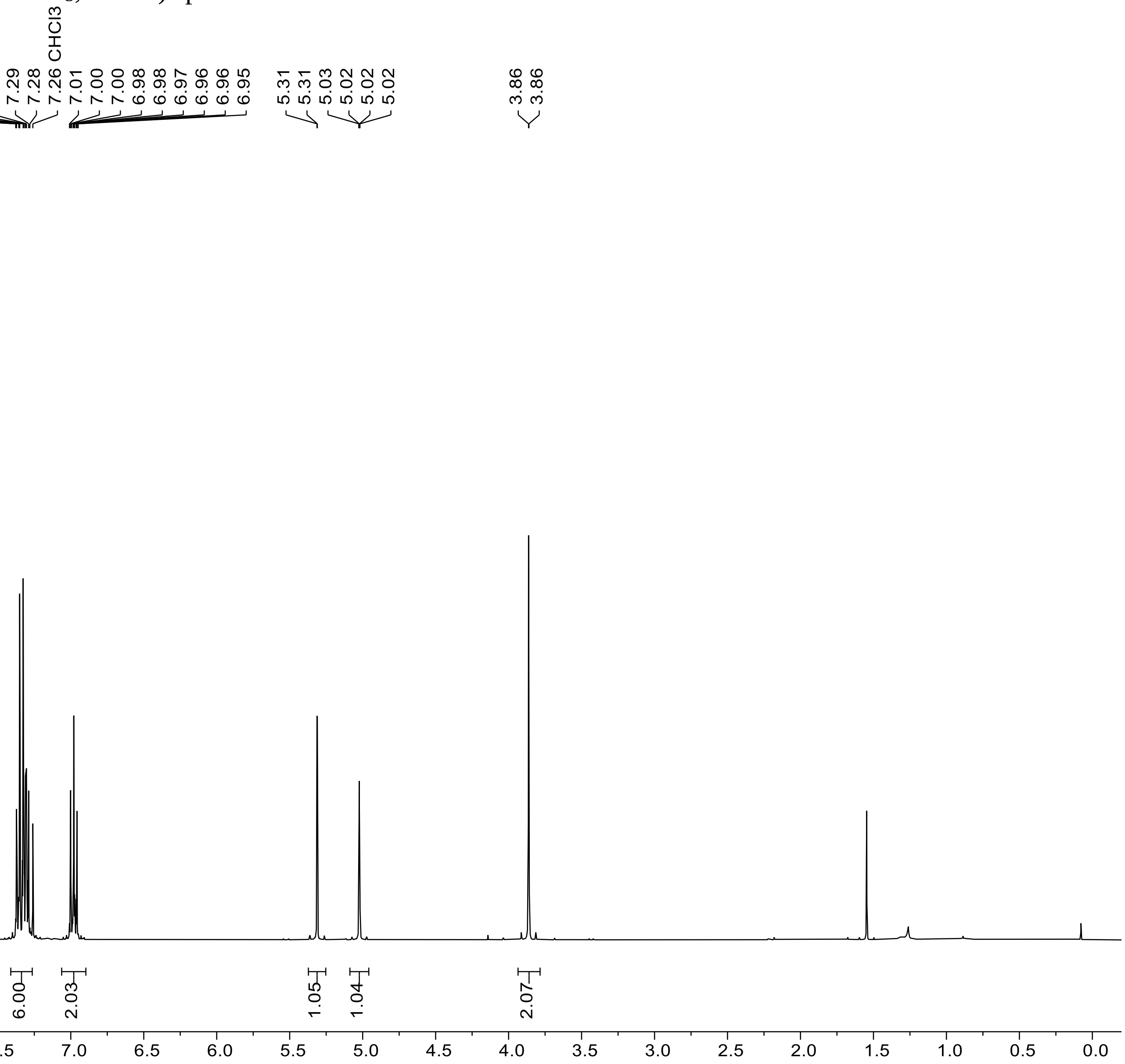

Figure S123: **^{13}C NMR** (101 MHz, $CDCl_3$, 298 K) spectrum of **4ra**.

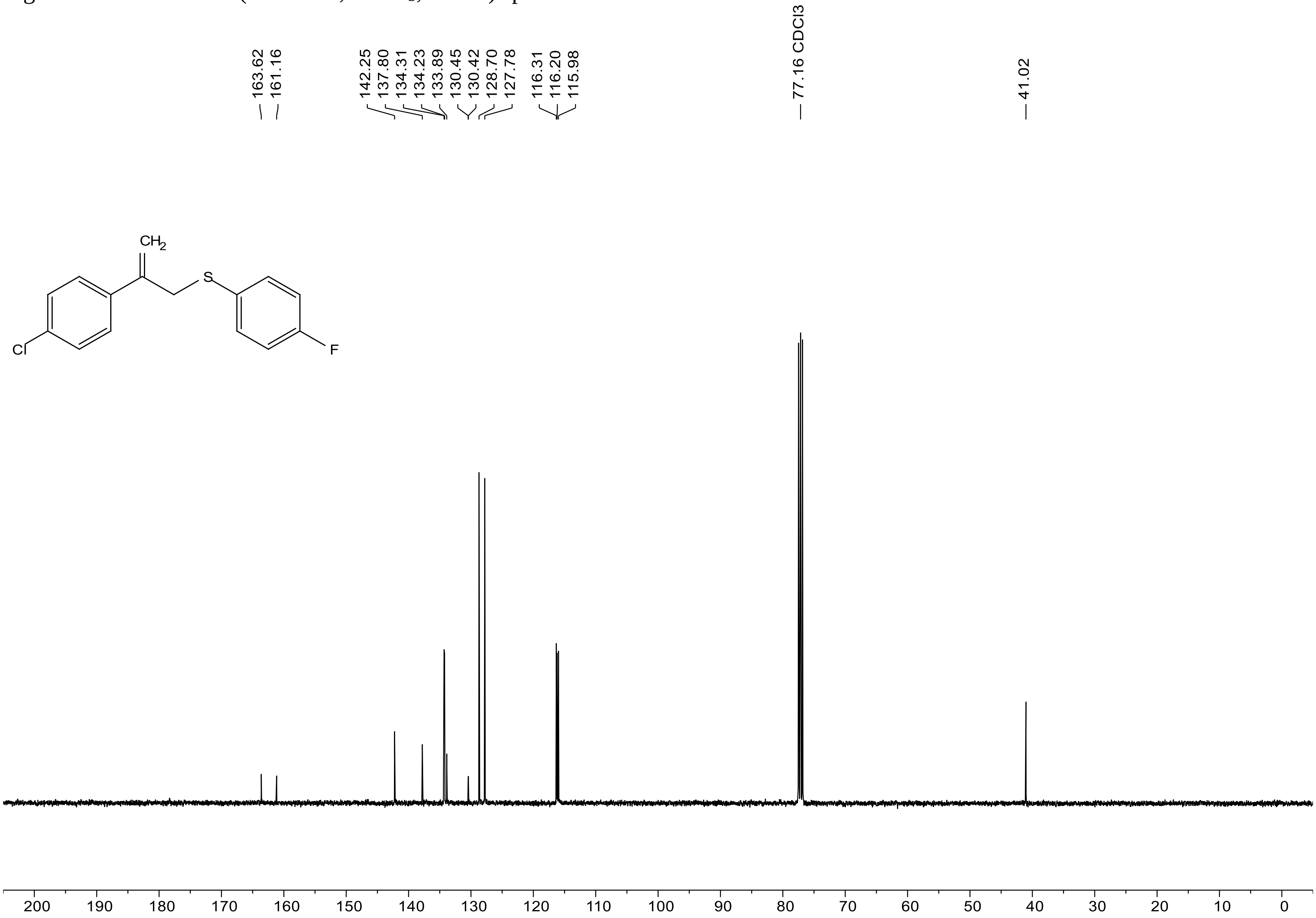

Figure S124: **^{19}F NMR** (376 MHz, $CDCl_3$, 298 K) spectrum of **4ra**.

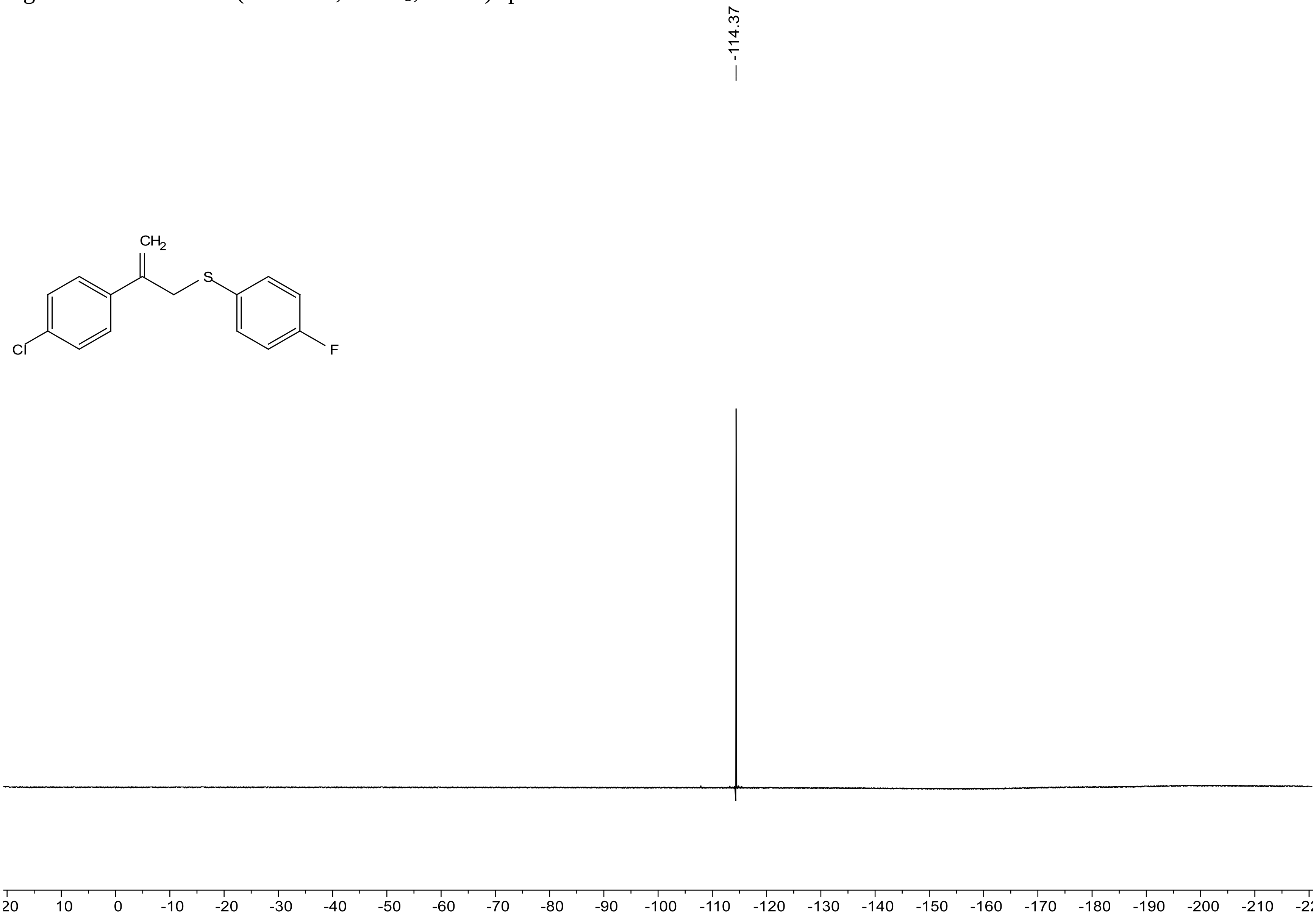

Figure S125: **^{1}H NMR** (400 MHz, $CDCl_3$, 298 K) spectrum of **4sa**.

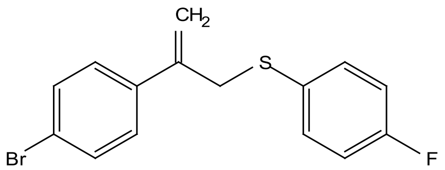

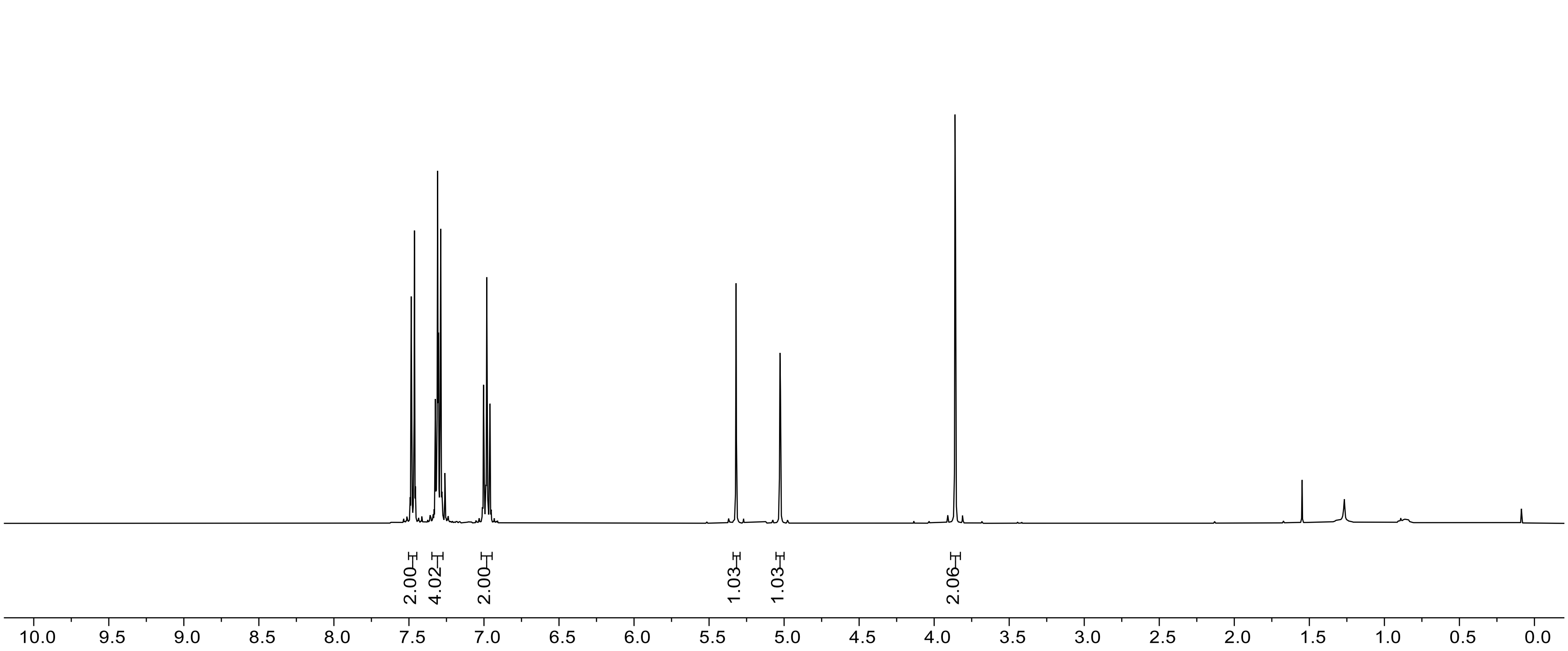

Figure S126: **^{13}C NMR** (101 MHz, $CDCl_3$, 298 K) spectrum of **4sa**.

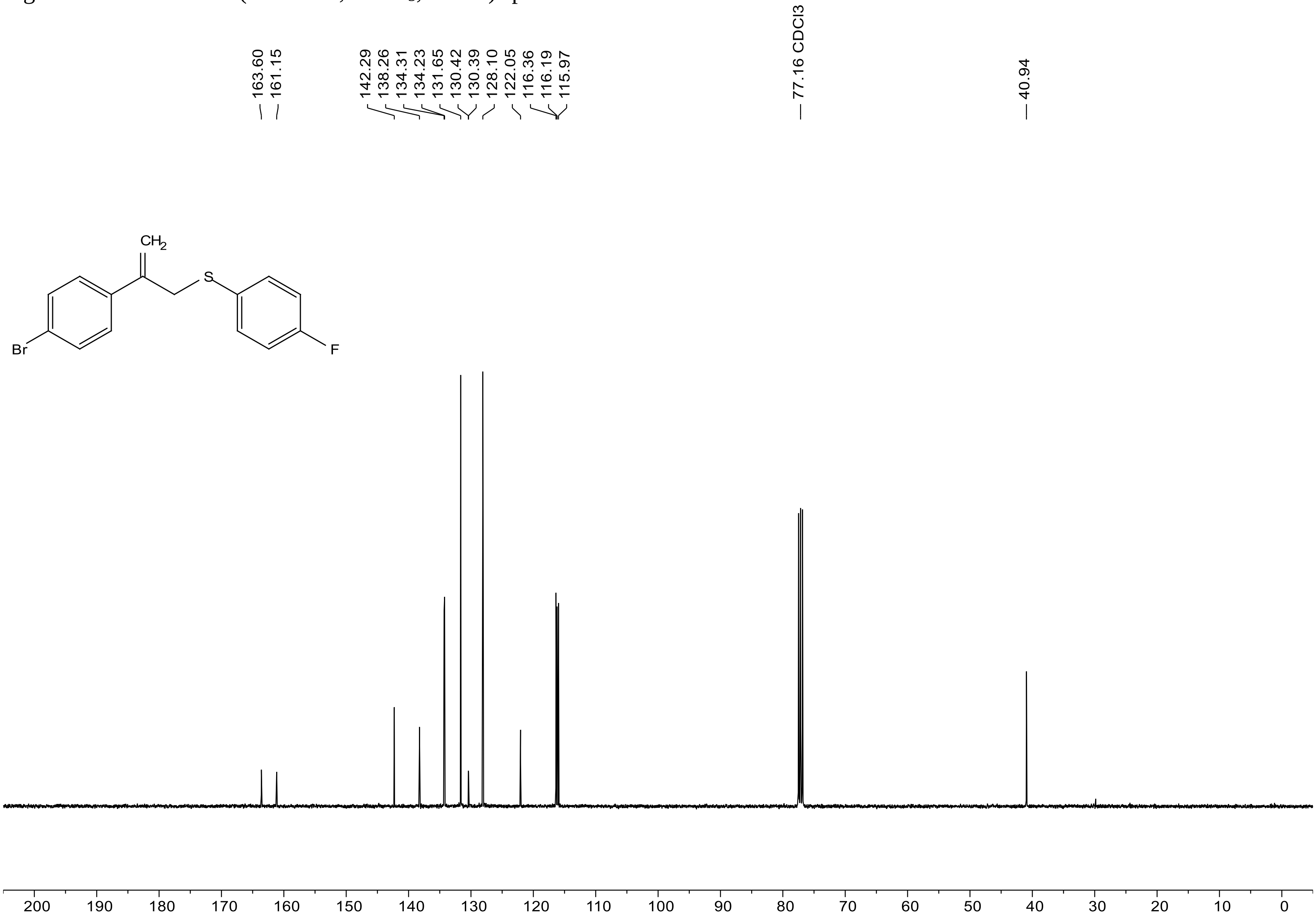

Figure S127: ^{19}**F NMR** (376 MHz, $CDCl_3$, 298 K) spectrum of **4sa**.

-114.32

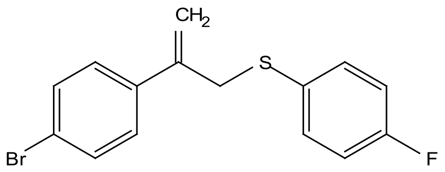

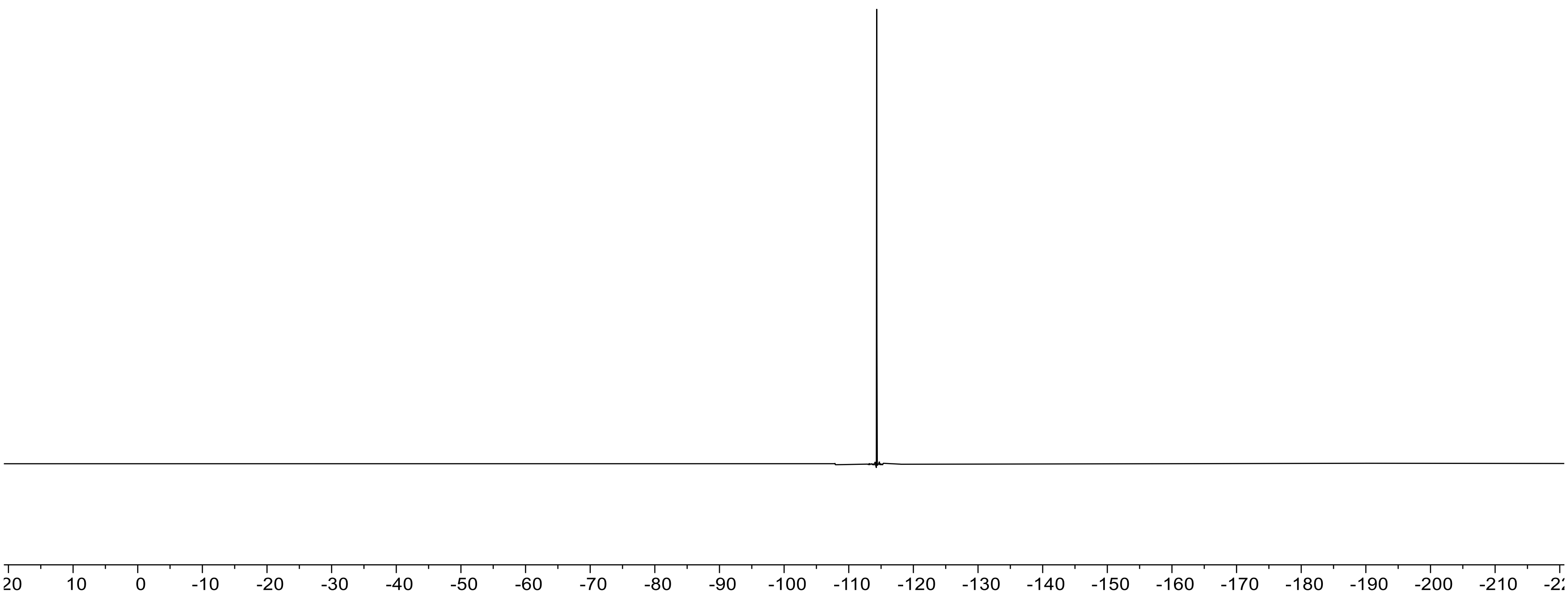

Figure S128: **^{1}H NMR** (400 MHz, $CDCl_3$, 298 K) spectrum of **4ta**.

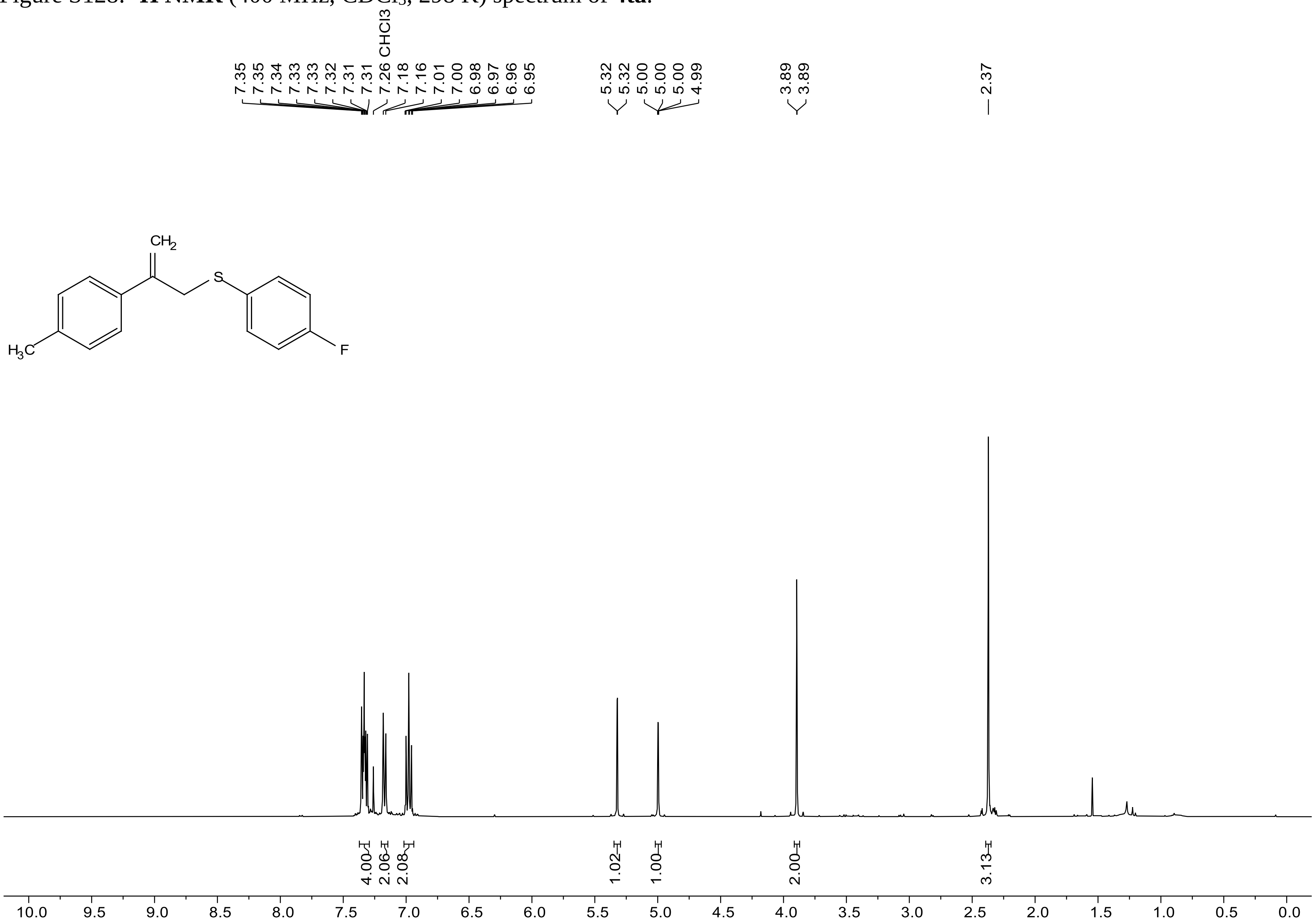

Figure S129: ^{13}**C NMR** (101 MHz, $CDCl_3$, 298 K) spectrum of **4ta**.

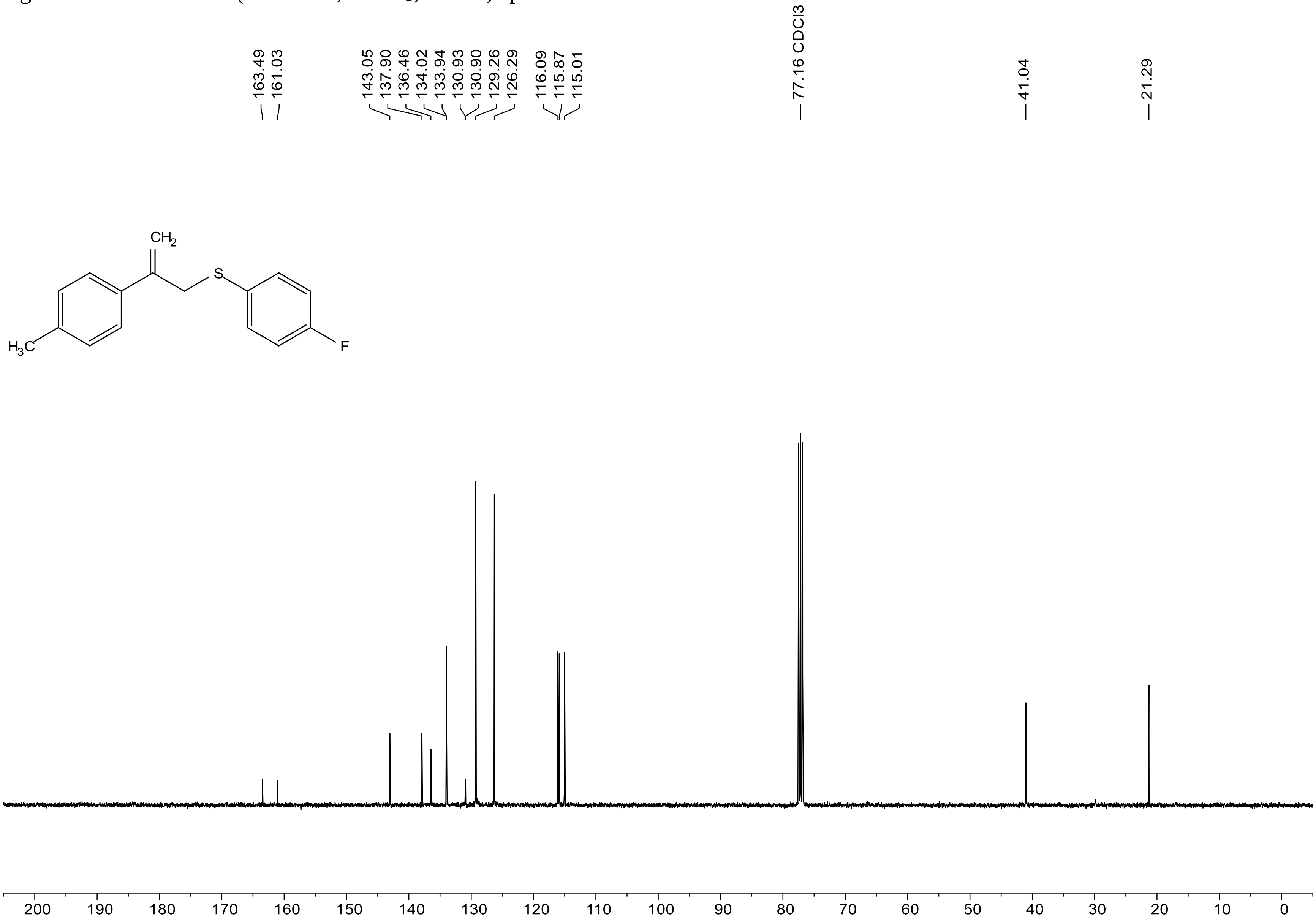

Figure S130: **^{19}F NMR** (376 MHz, $CDCl_3$, 298 K) spectrum of **4ta**.

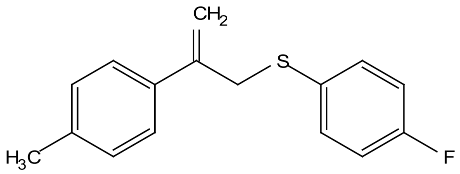

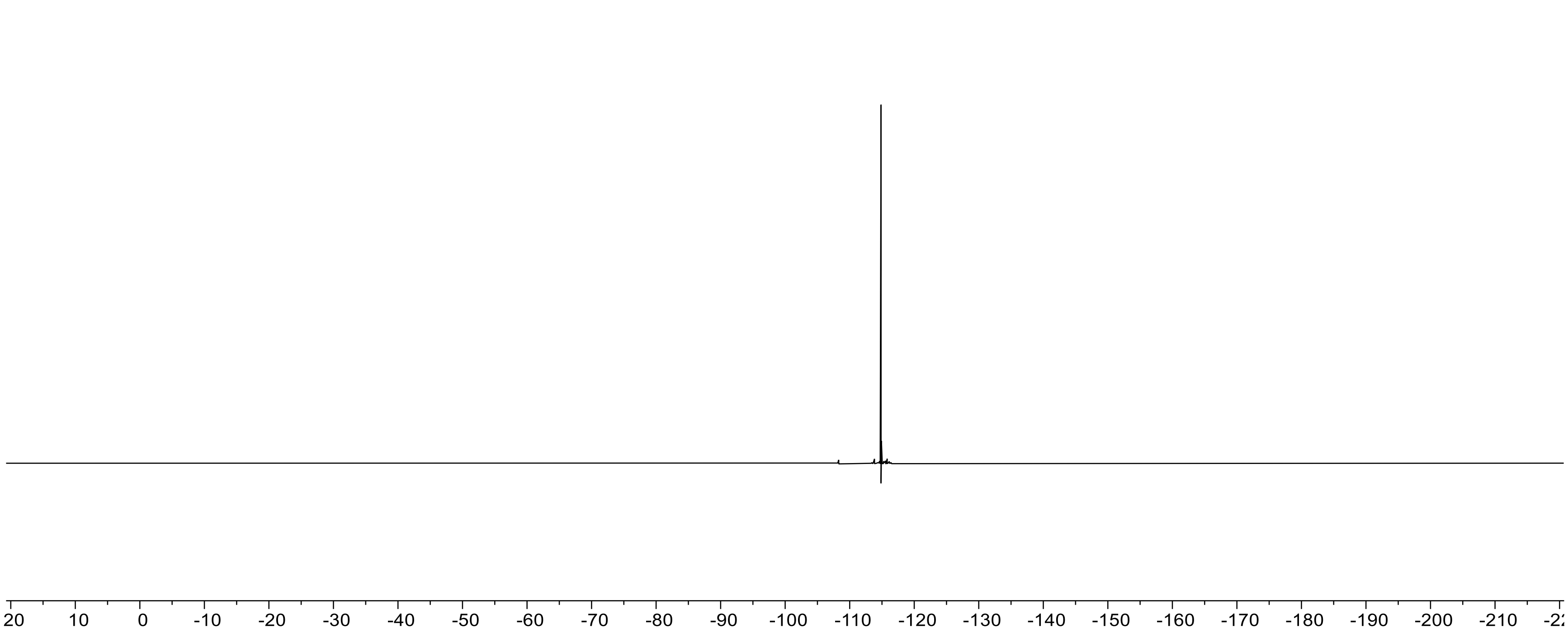

Figure S131: **^{1}H NMR** (400 MHz, $CDCl_3$, 298 K) spectrum of **4ua**.

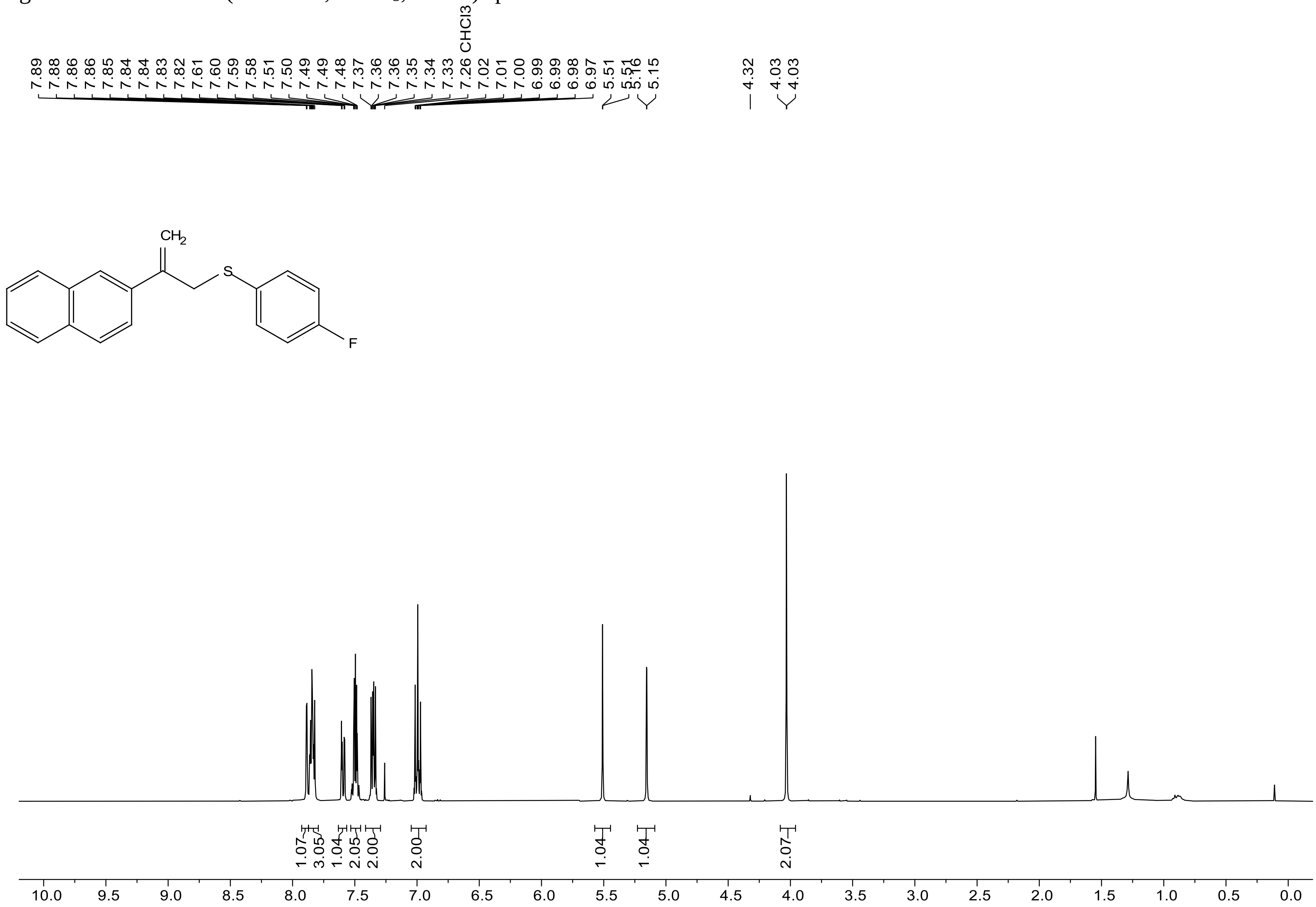

Figure S132: ^{13}**C NMR** (101 MHz, $CDCl_3$, 298 K) spectrum of **4ua**.

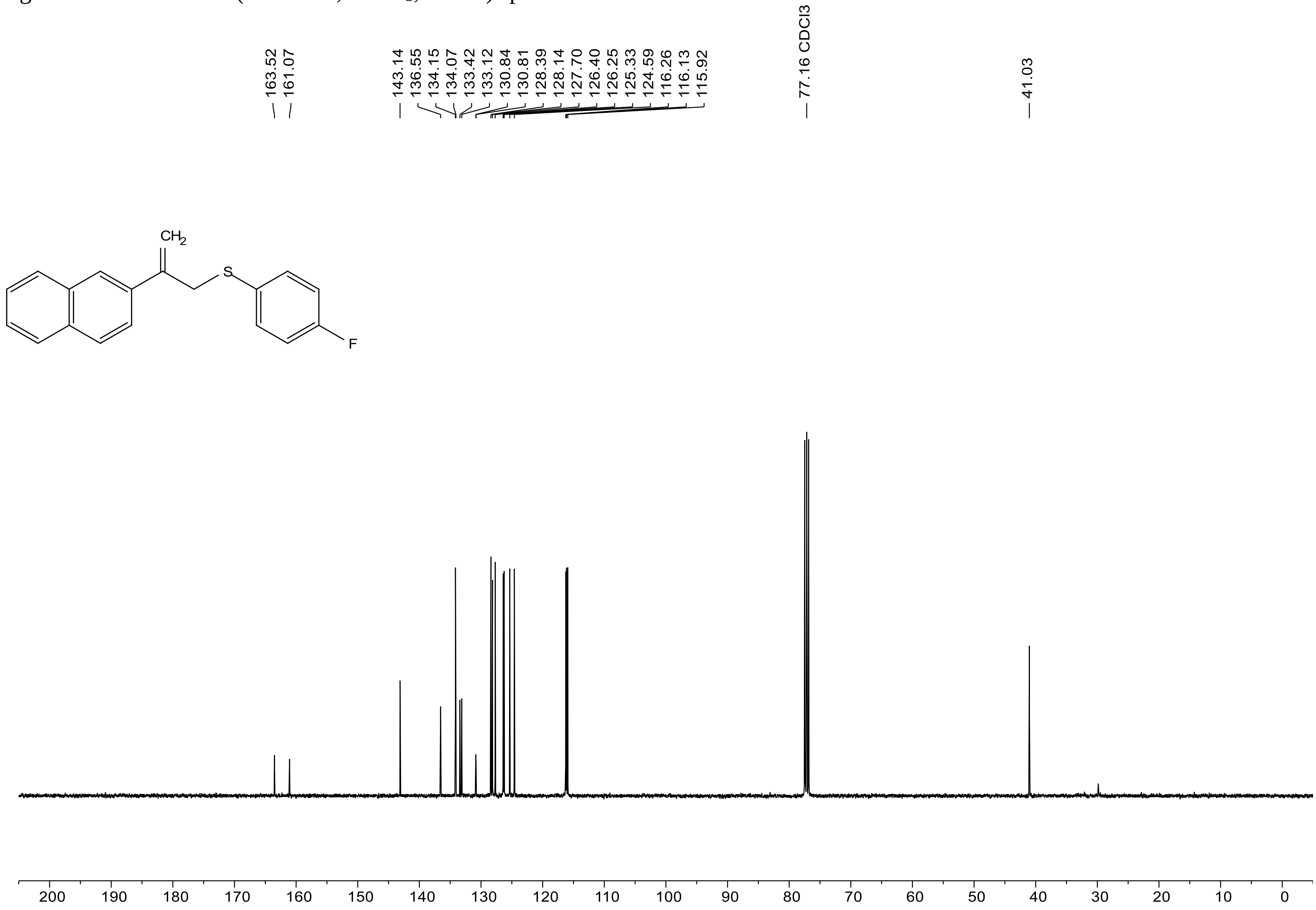

Figure S133: **^{19}F NMR** (376 MHz, $CDCl_3$, 298 K) spectrum of **4ua**.

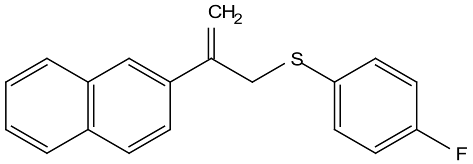

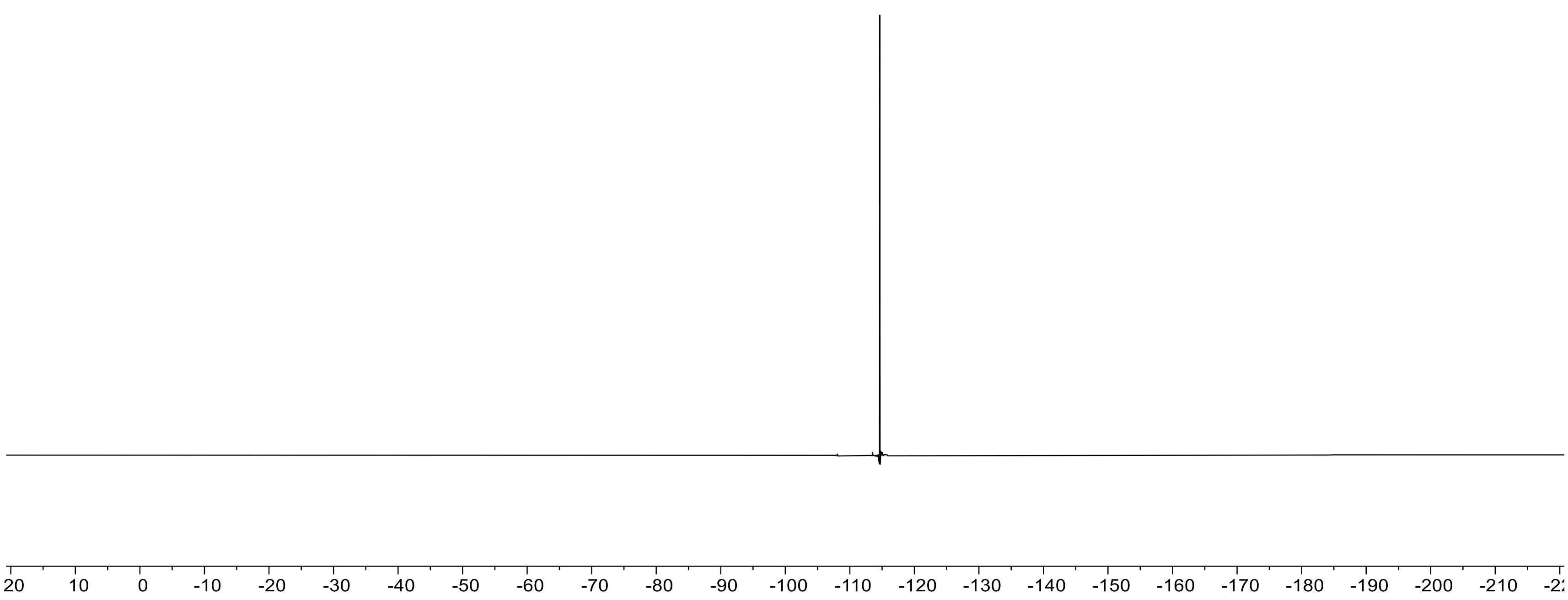

Figure S134: **¹H NMR** (400 MHz, $CDCl_3$, 298 K) spectrum of **4va**.

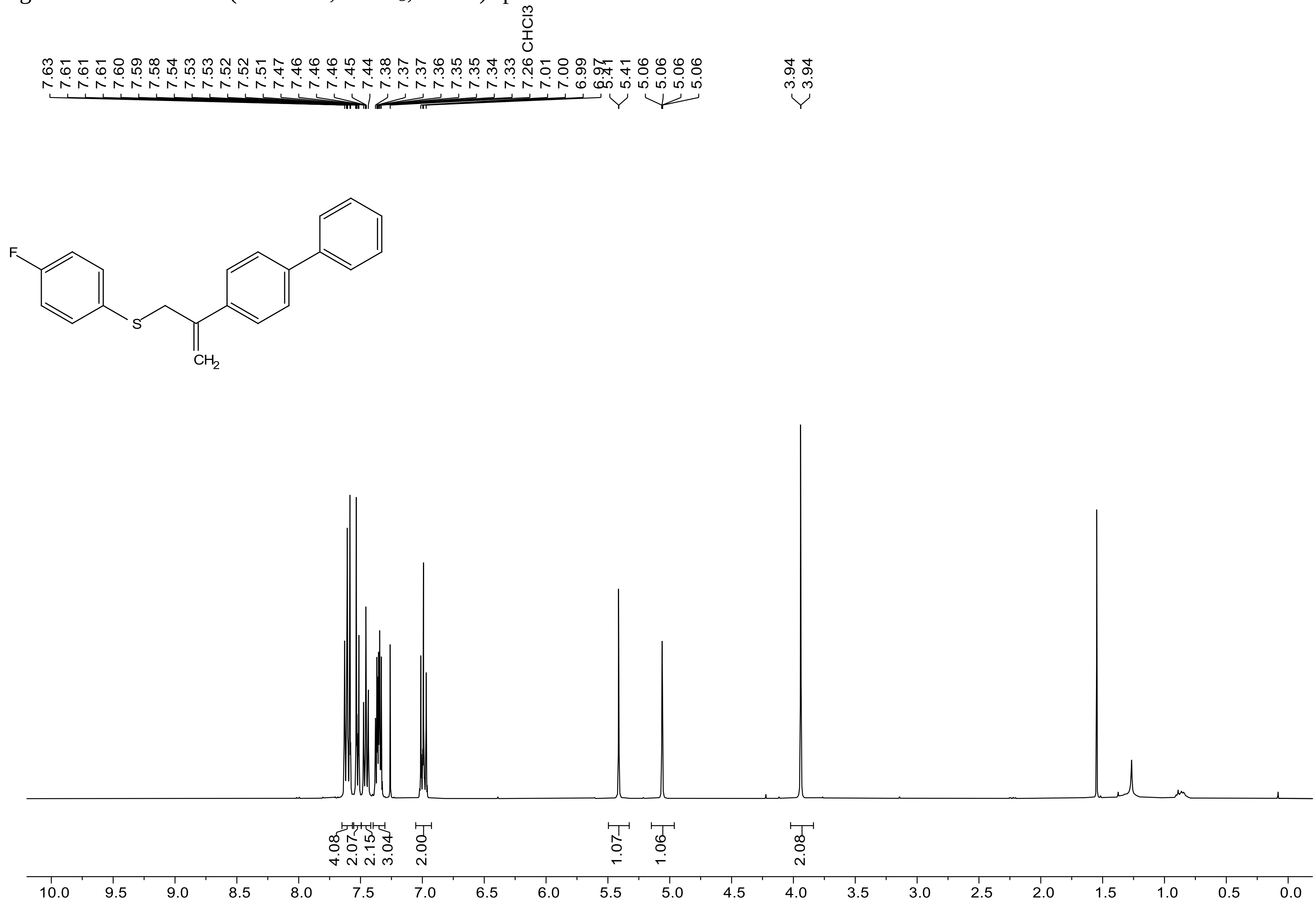

Figure S135: **^{13}C NMR** (101 MHz, $CDCl_3$, 298 K) spectrum of **4va**.

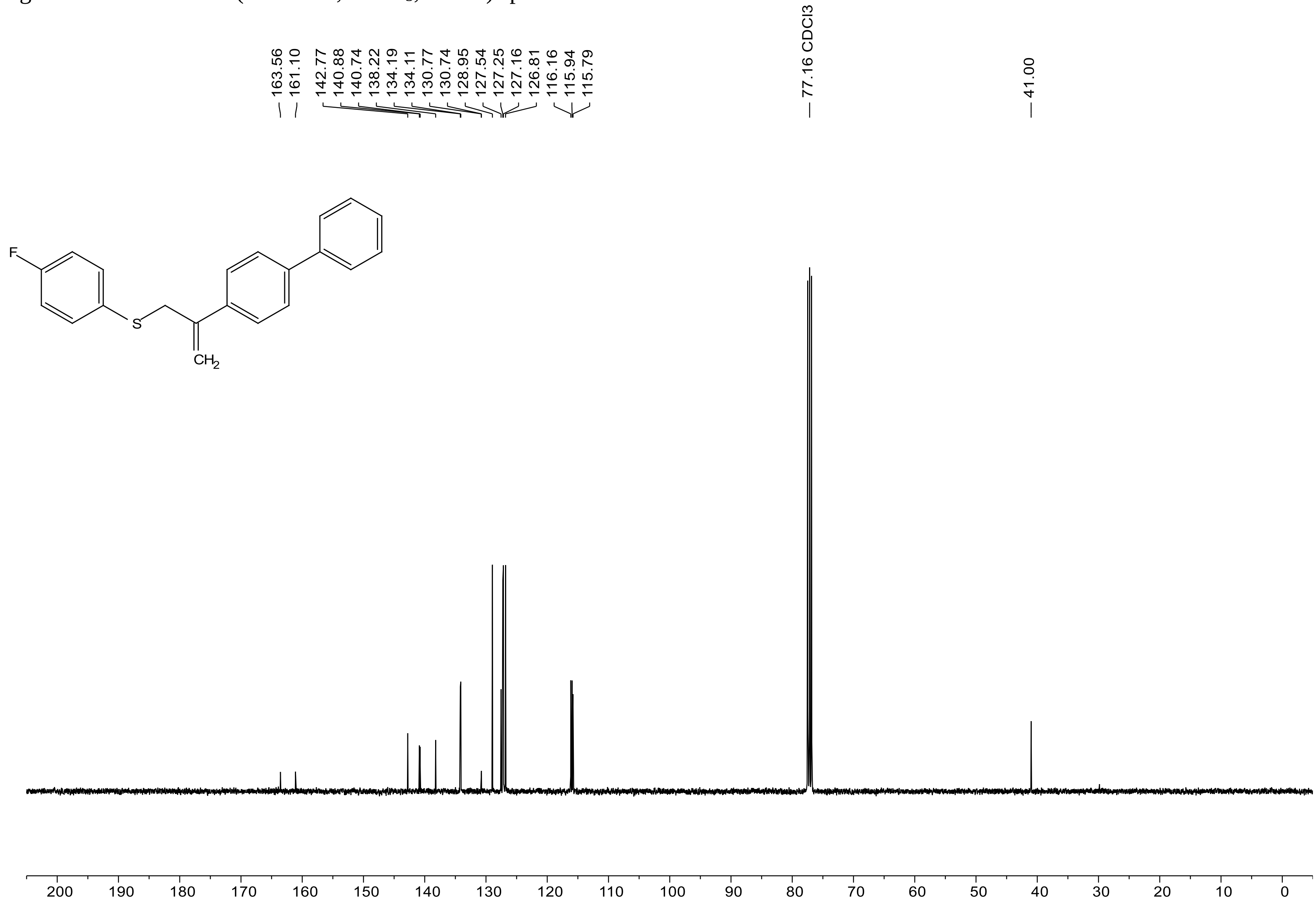

Figure S136: ^{19}**F NMR** (376 MHz, $CDCl_3$, 298 K) spectrum of **4va**.

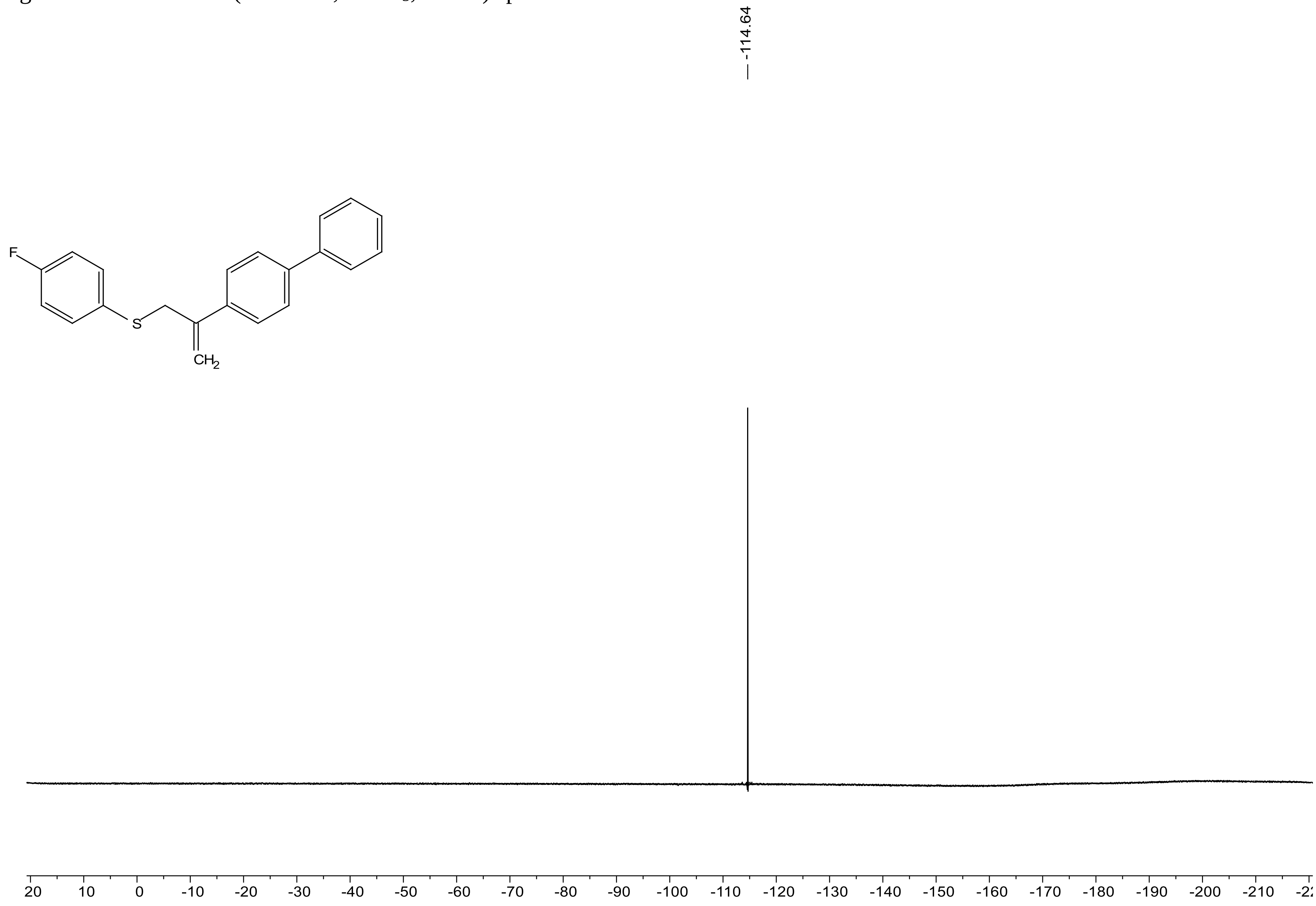

Figure S137: **^{1}H NMR** (400 MHz, $CDCl_3$, 298 K) spectrum of **4wa**.

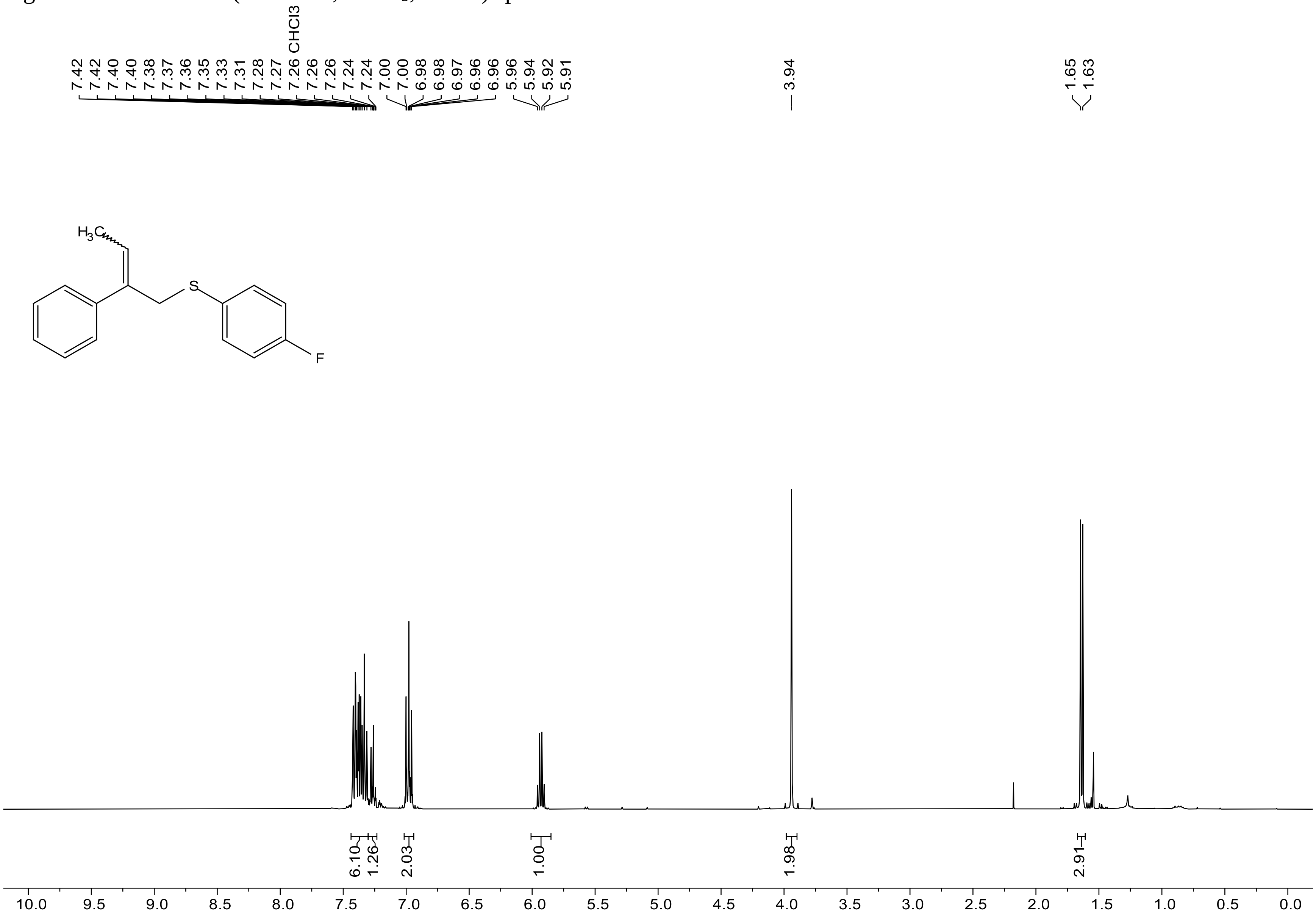

Figure S138: ^{13}**C NMR** (101 MHz, $CDCl_3$, 298 K) spectrum of **4wa**.

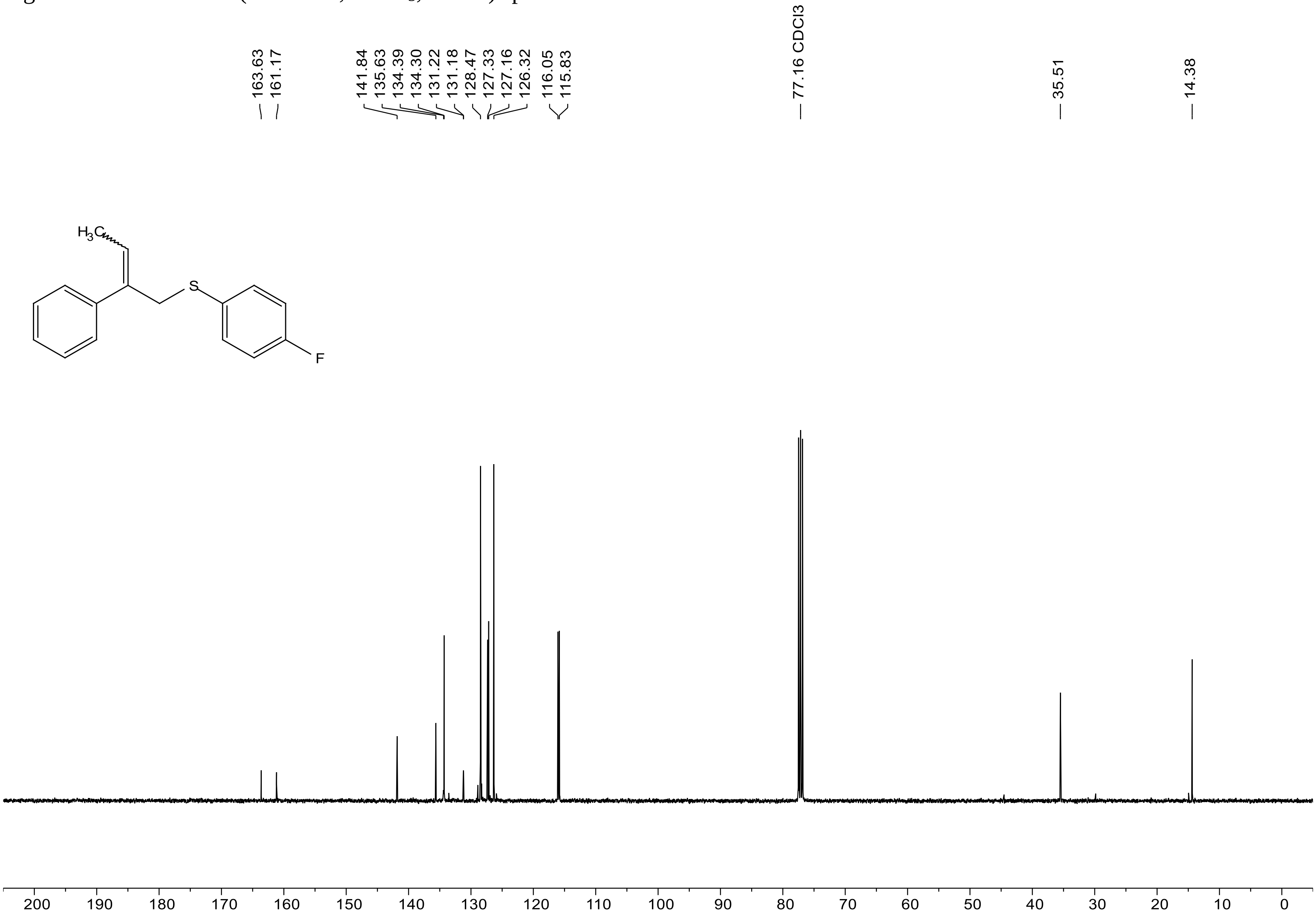

Figure S139: **^{19}F NMR** (376 MHz, $CDCl_3$, 298 K) spectrum of **4wa**.

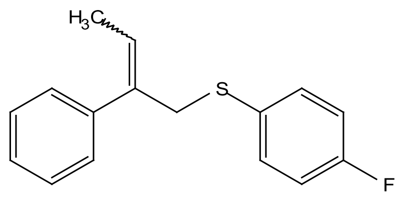

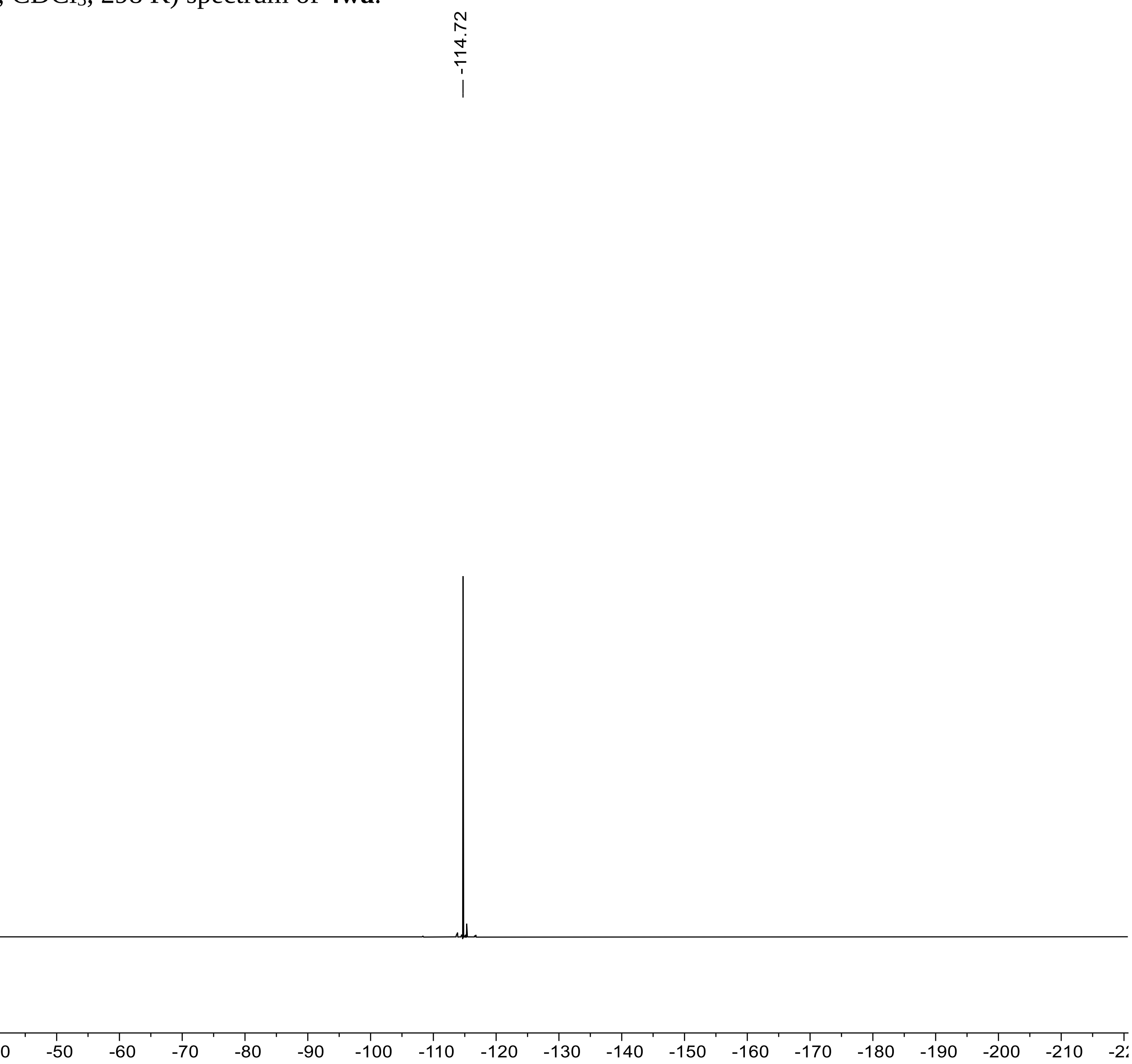

Figure S140: **^{1}H NMR** (400 MHz, $CDCl_3$, 298 K) spectrum of **4xa**.

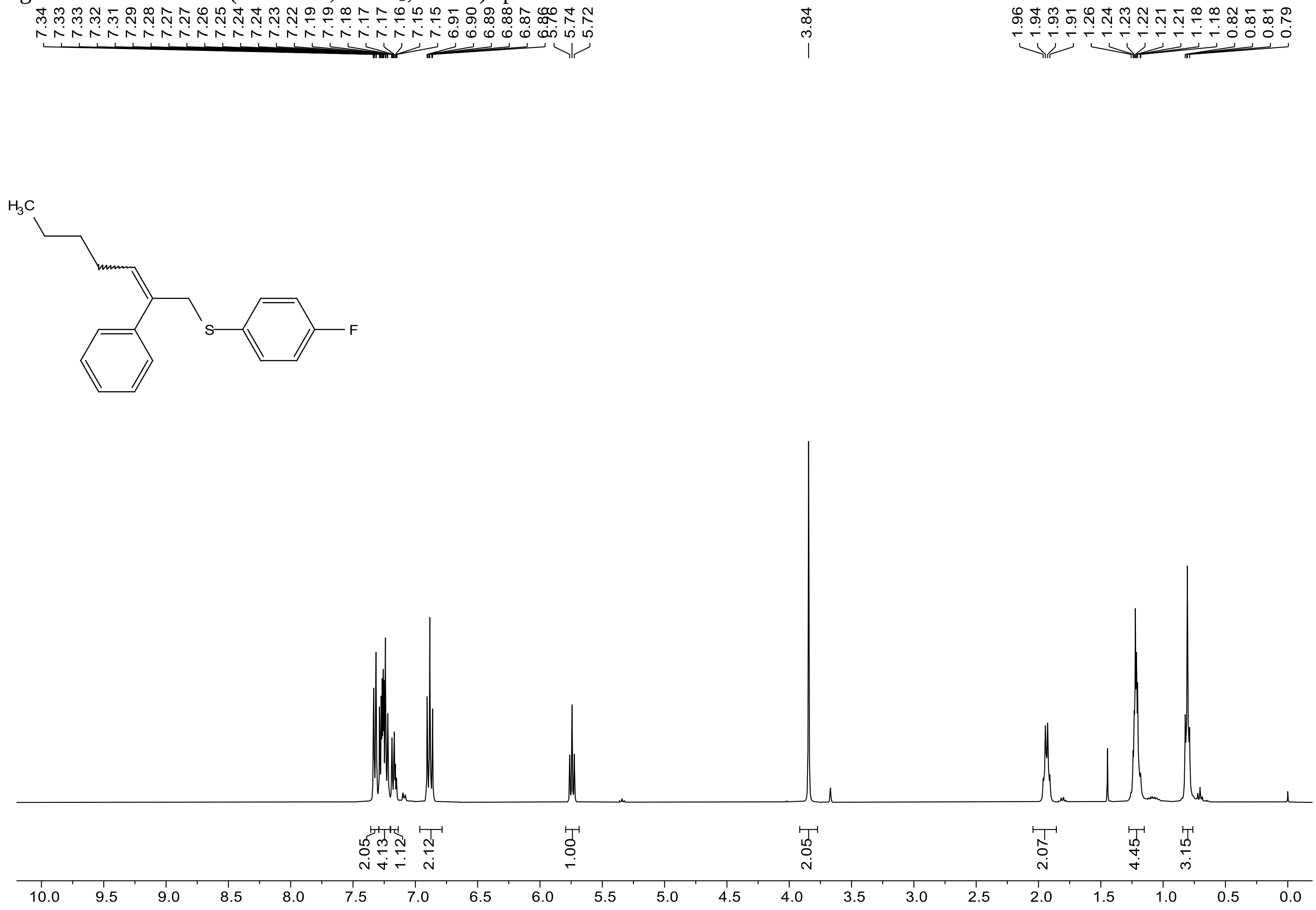

Figure S141: ^{13}**C NMR** (101 MHz, $CDCl_3$, 298 K) spectrum of **4xa**.

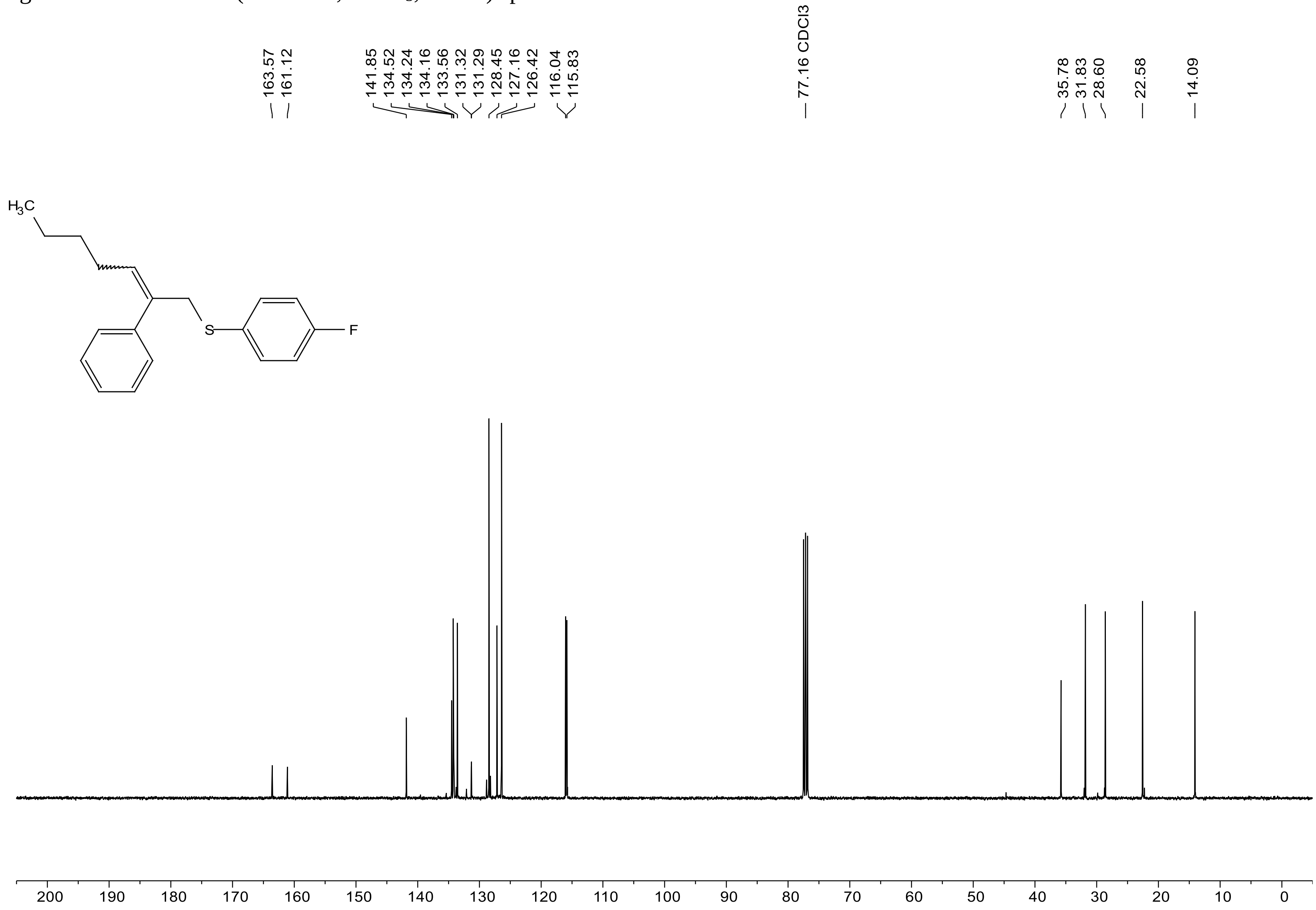

Figure S142: ^{19}**F NMR** (376 MHz, $CDCl_3$, 298 K) spectrum of **4xa**.

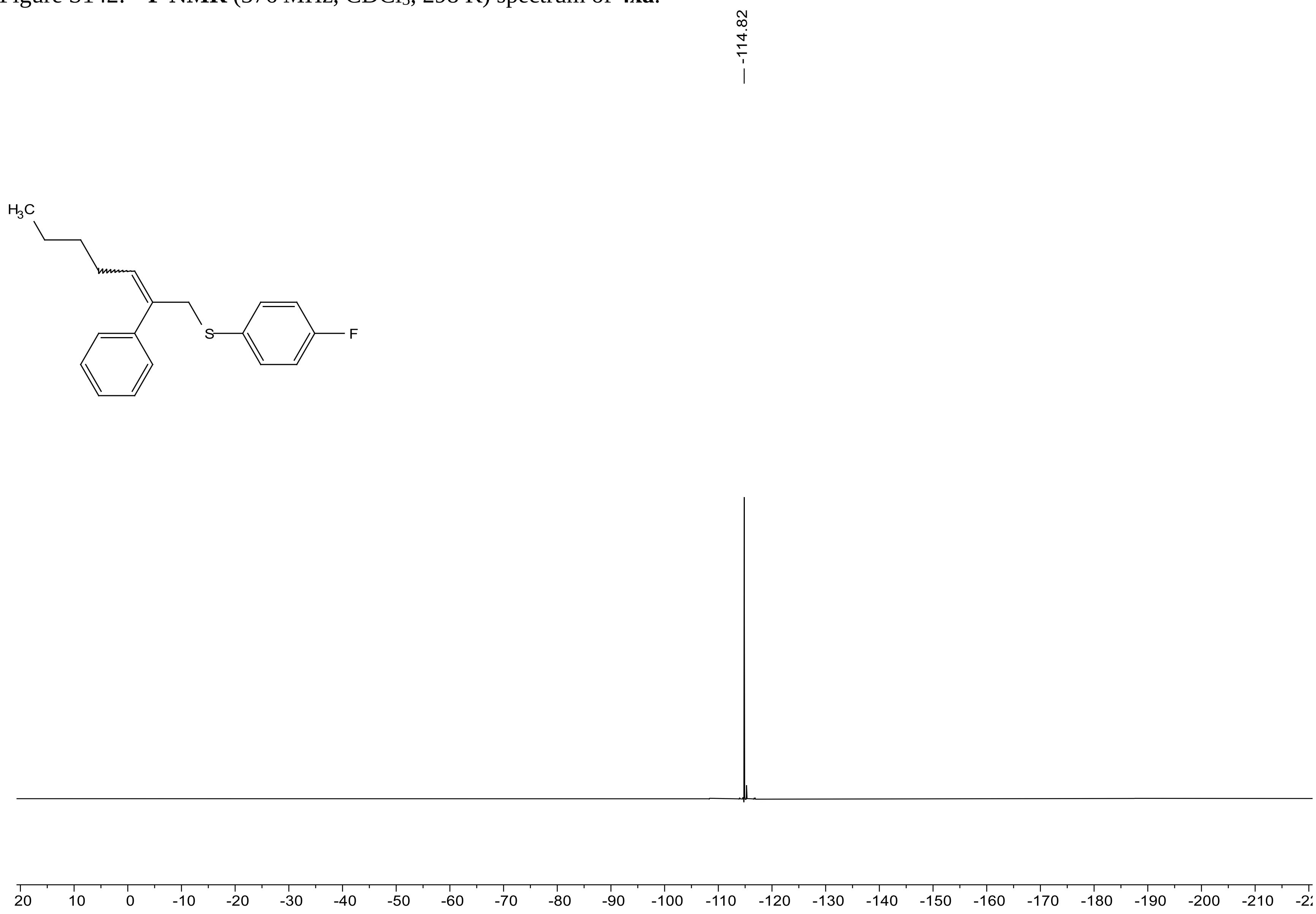

Figure S143: ^{1}**H NMR** (400 MHz, $CDCl_3$, 298 K) spectrum of **4pc**.

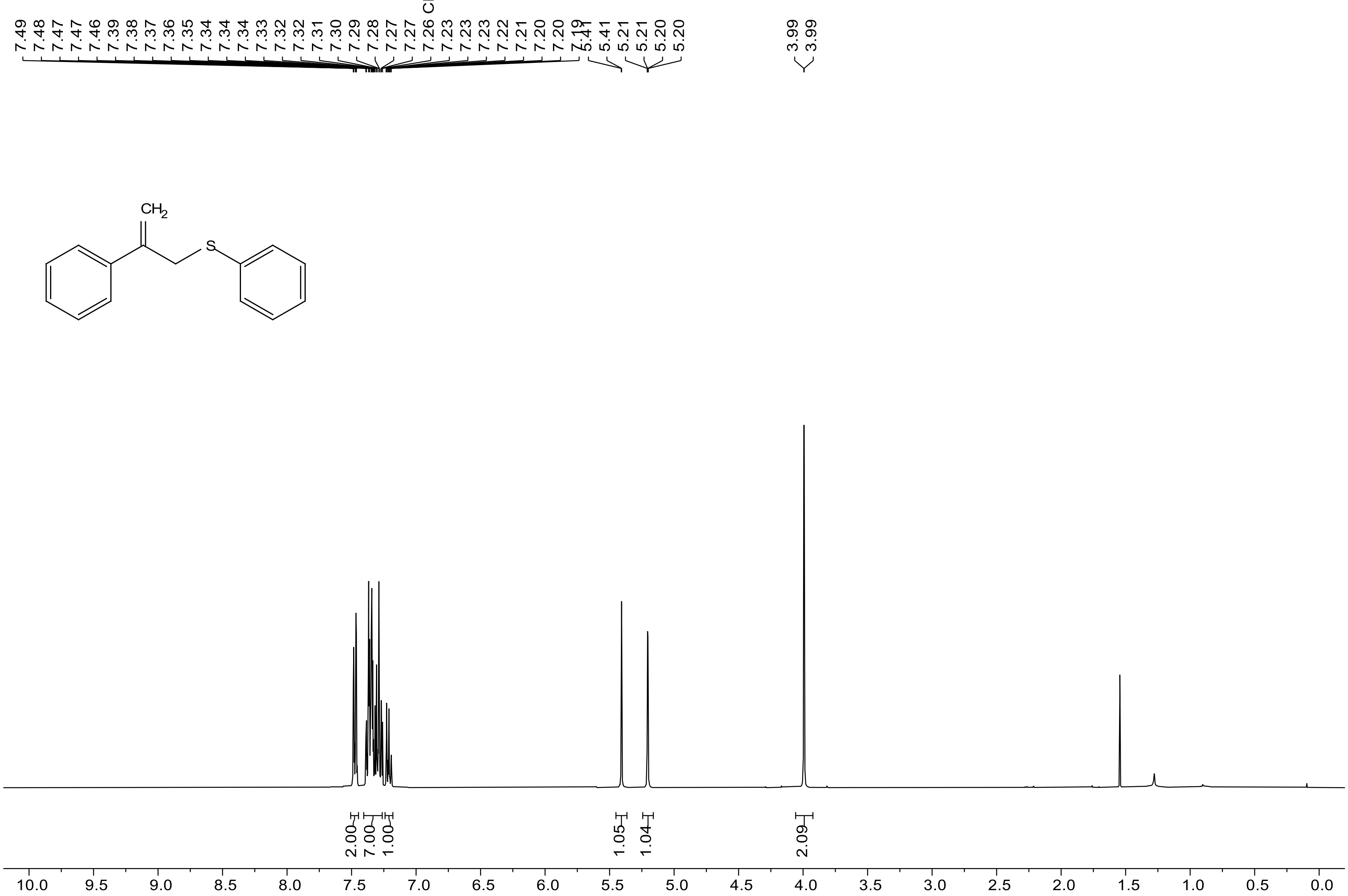

Figure S144: **^{13}C NMR** (101 MHz, $CDCl_3$, 298 K) spectrum of **4pc**.

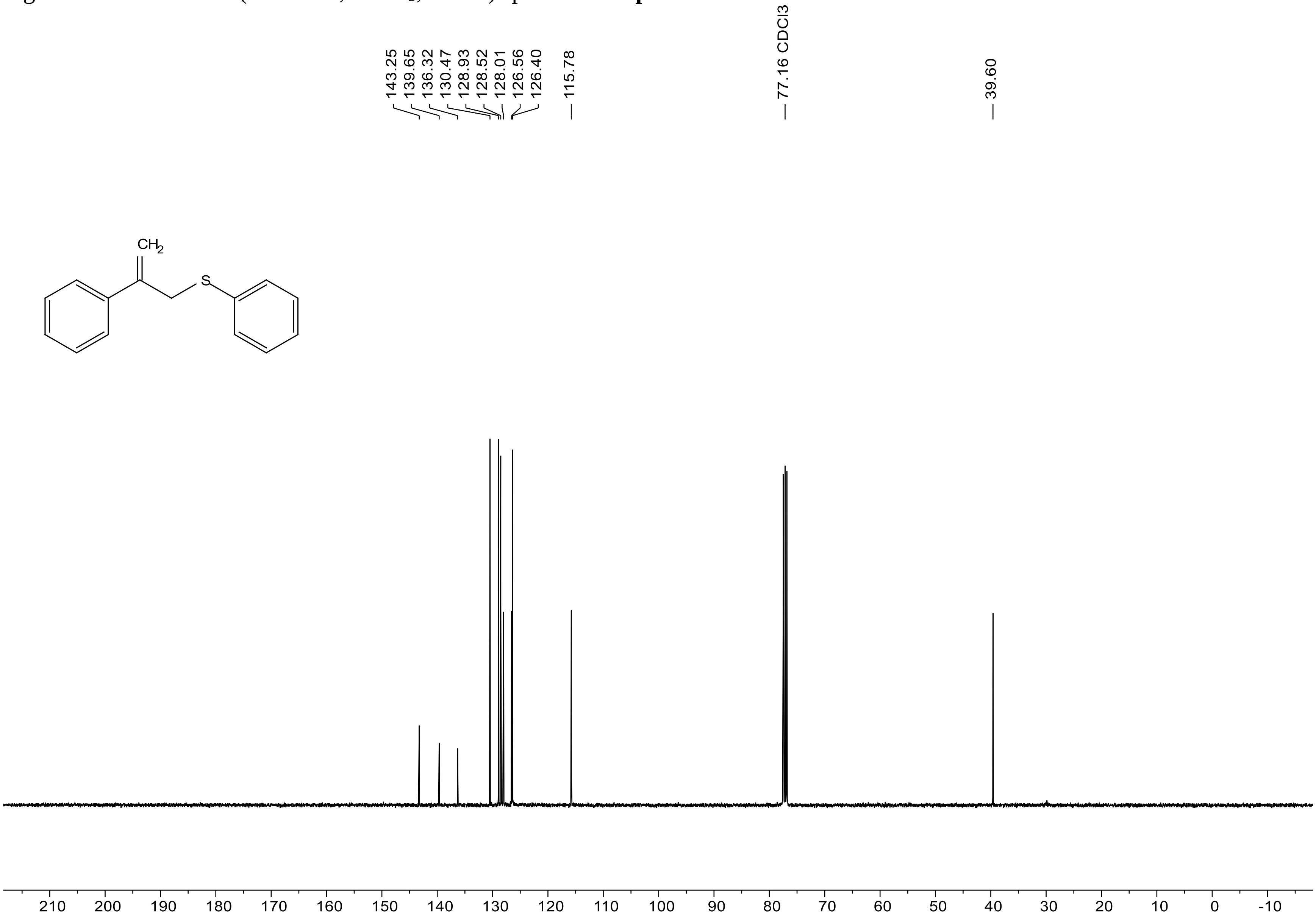

Figure S145: **^{1}H NMR** (400 MHz, $CDCl_3$, 298 K) spectrum of **4pb**.

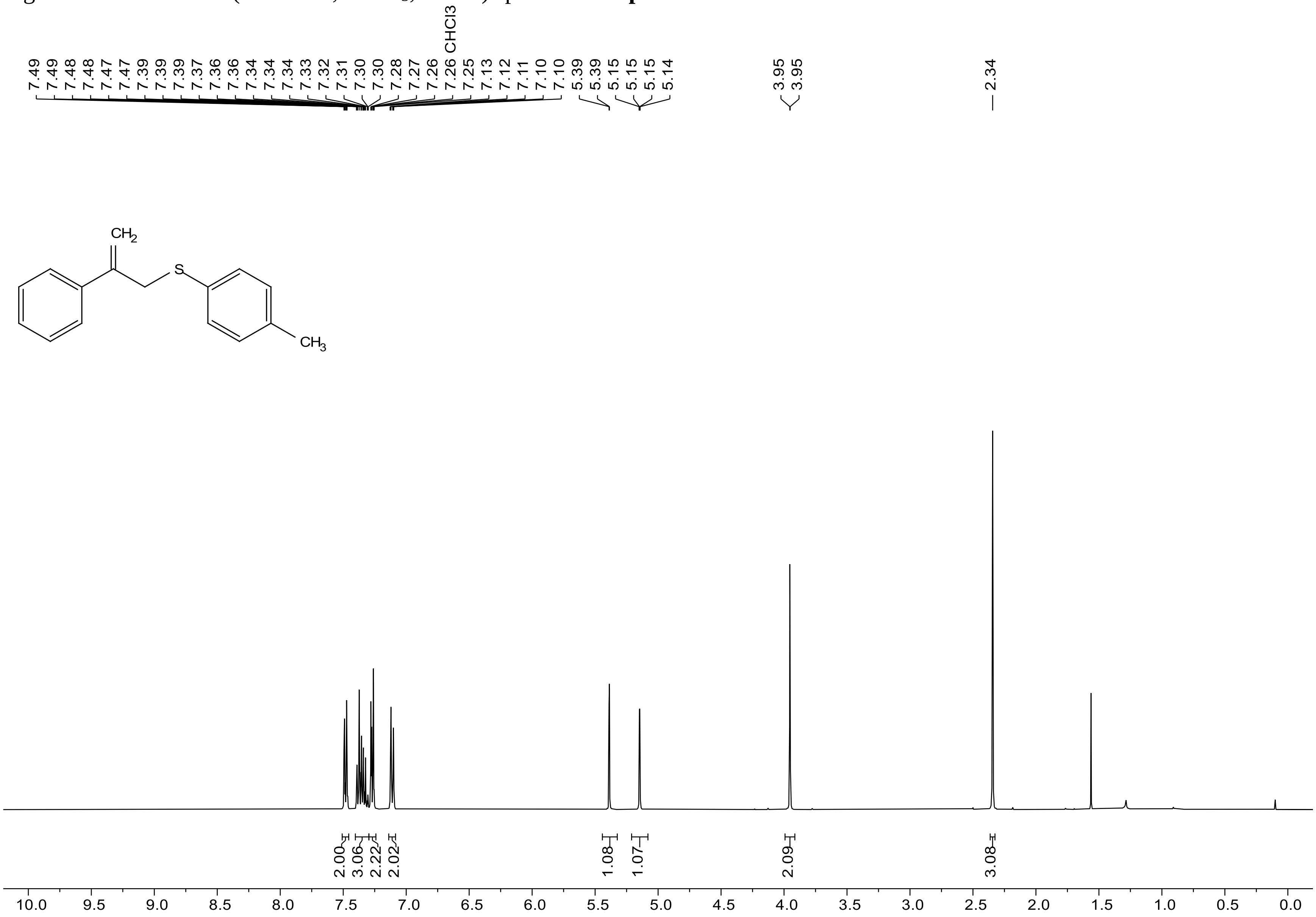

Figure S146: ^{13}**C NMR** (101 MHz, $CDCl_3$, 298 K) spectrum of **4pb**.

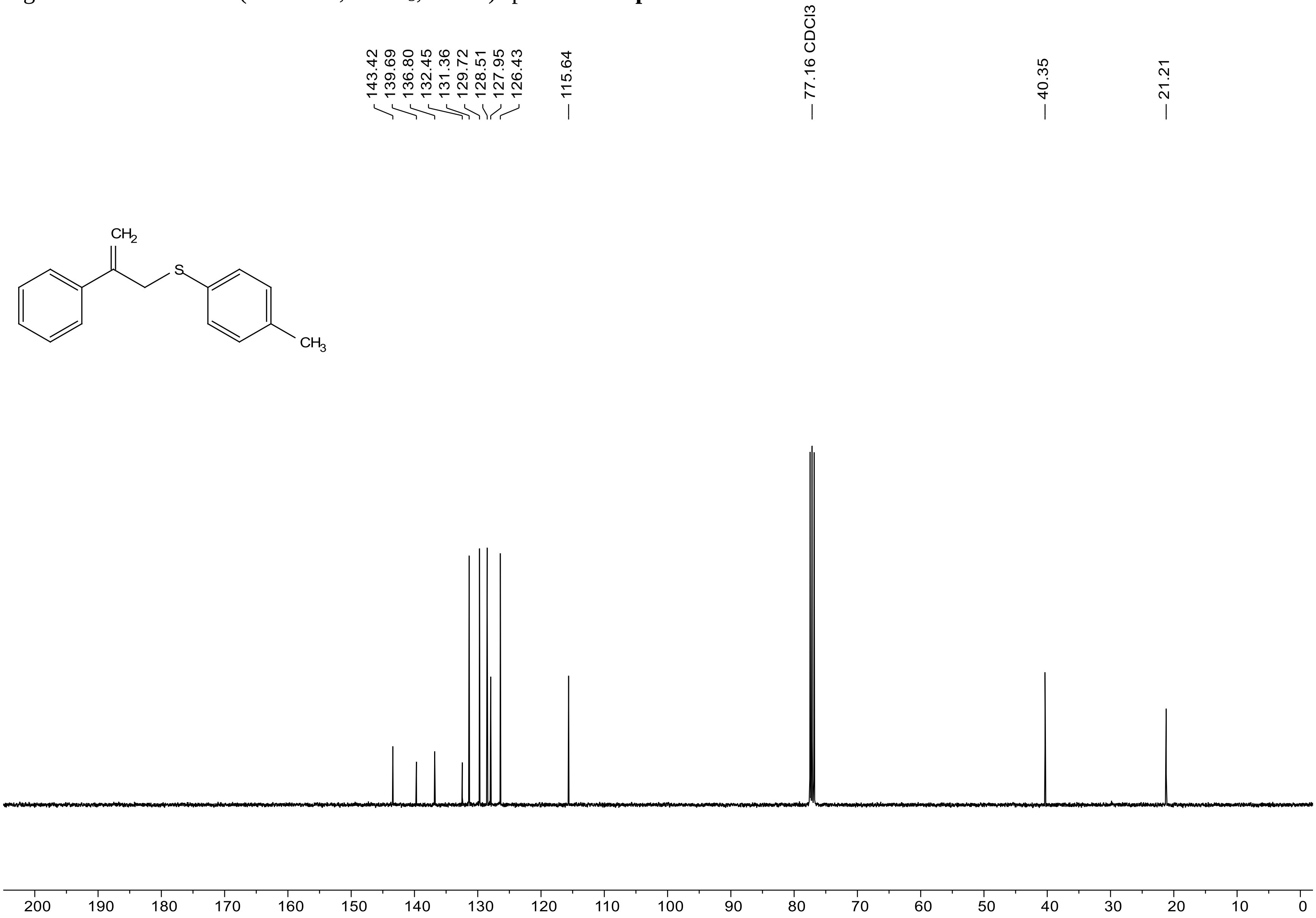

Figure S147: **^{1}H NMR** (400 MHz, $CDCl_3$, 298 K) spectrum of **4pd**.

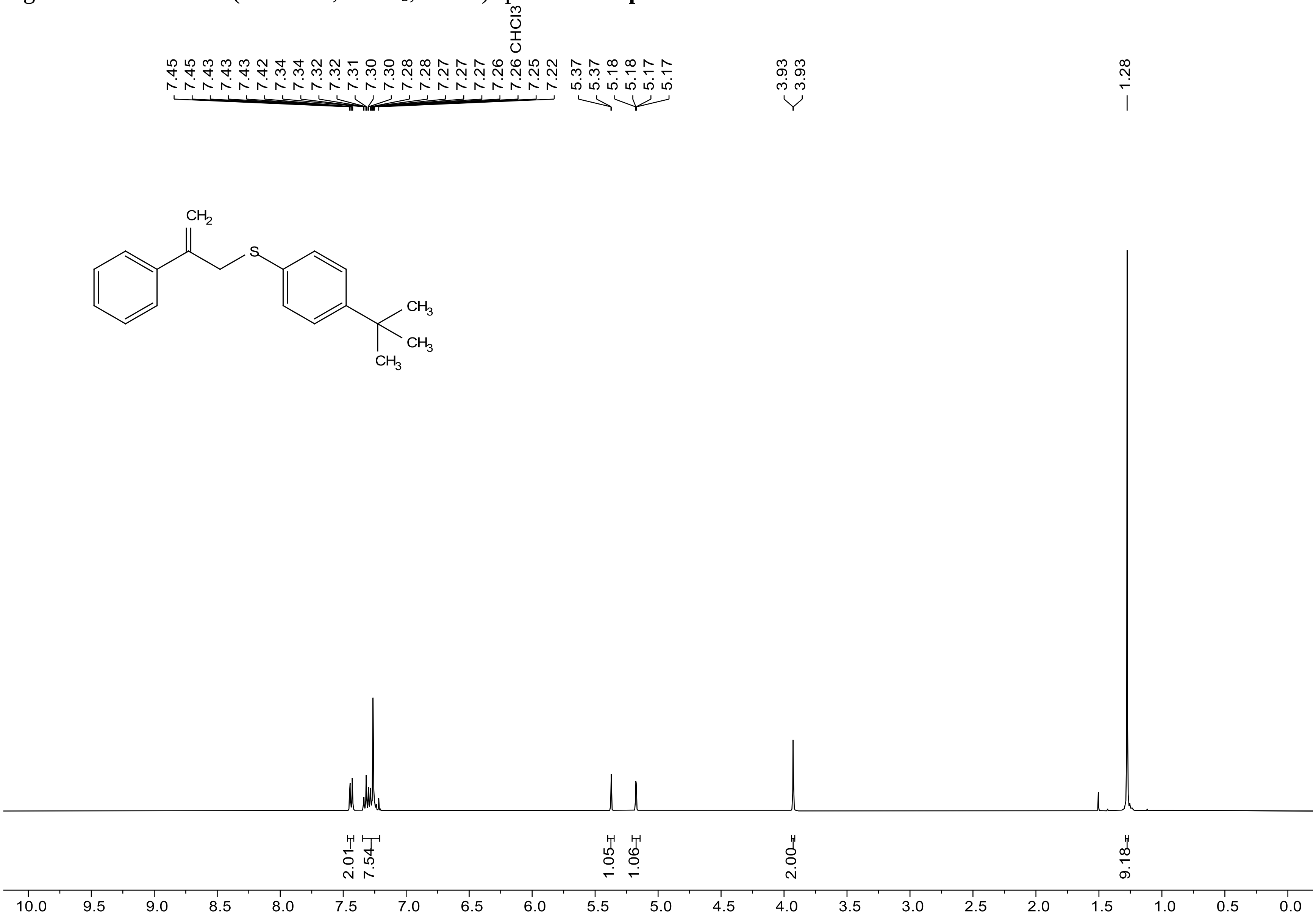

Figure S148: ^{13}**C NMR** (101 MHz, $CDCl_3$, 298 K) spectrum of **4pd**.

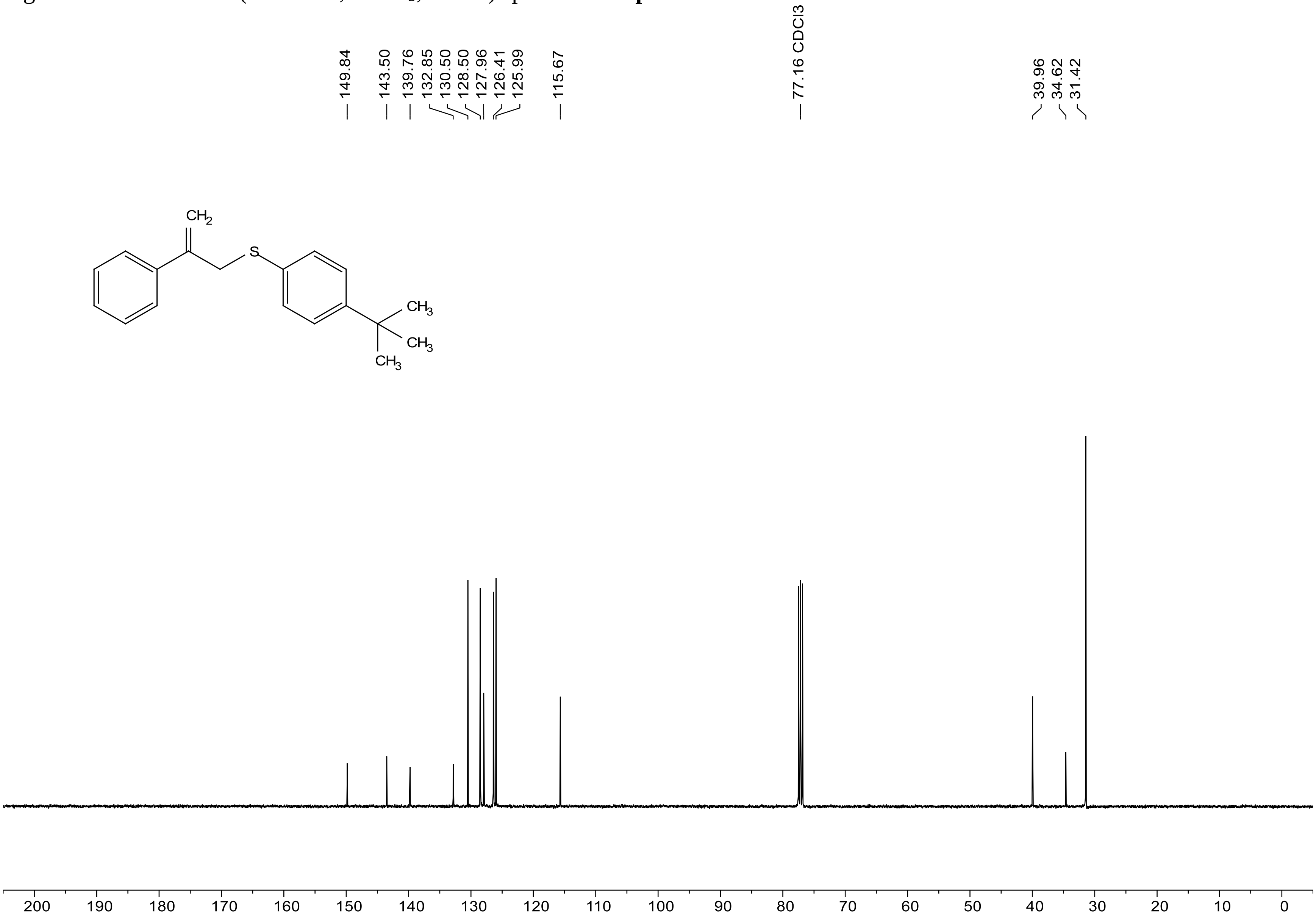

Figure S149: **^{1}H NMR** (400 MHz, $CDCl_3$, 298 K) spectrum of **4ph**.

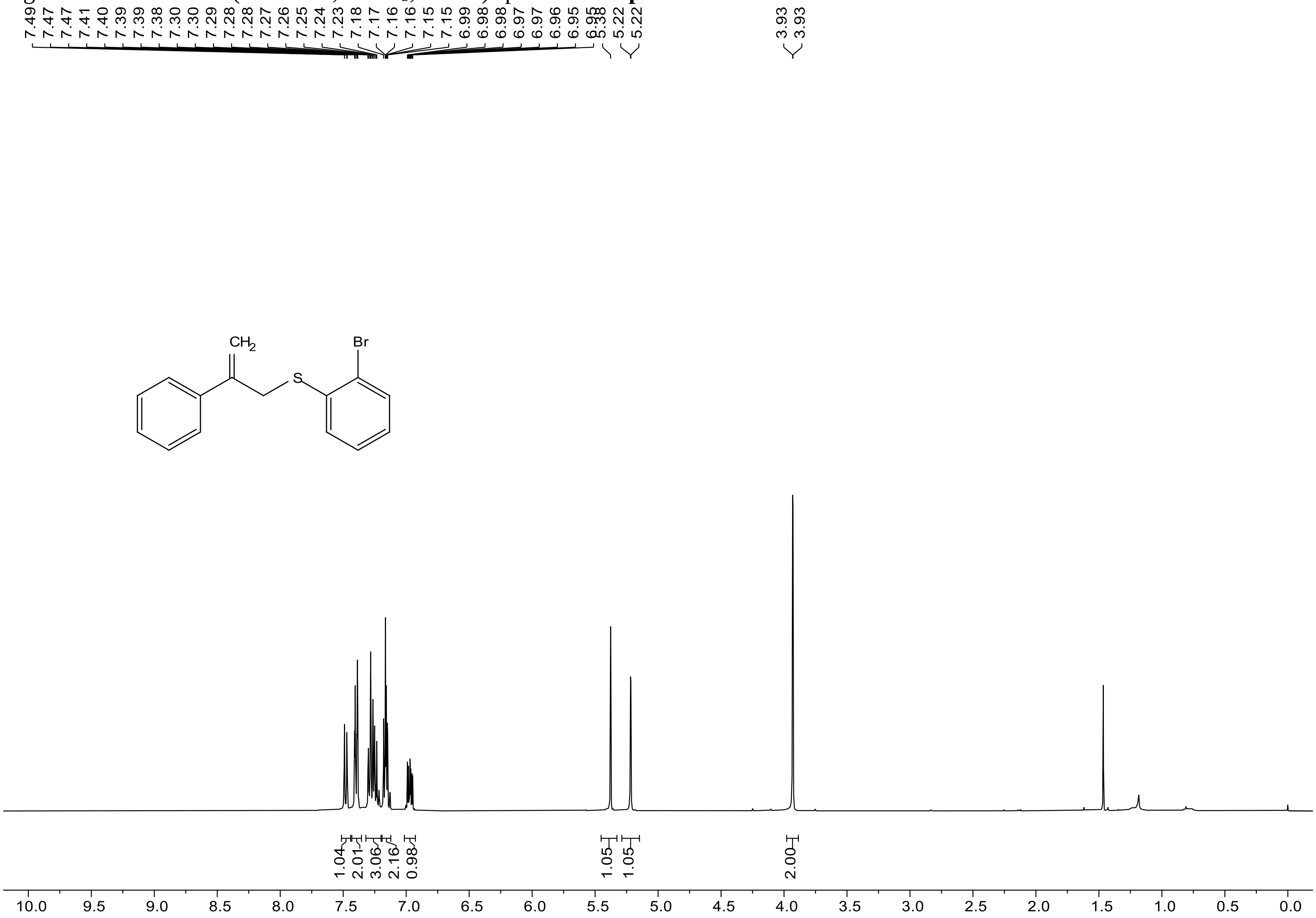

Figure S150: ^{13}**C NMR** (101 MHz, $CDCl_3$, 298 K) spectrum of **4ph**.

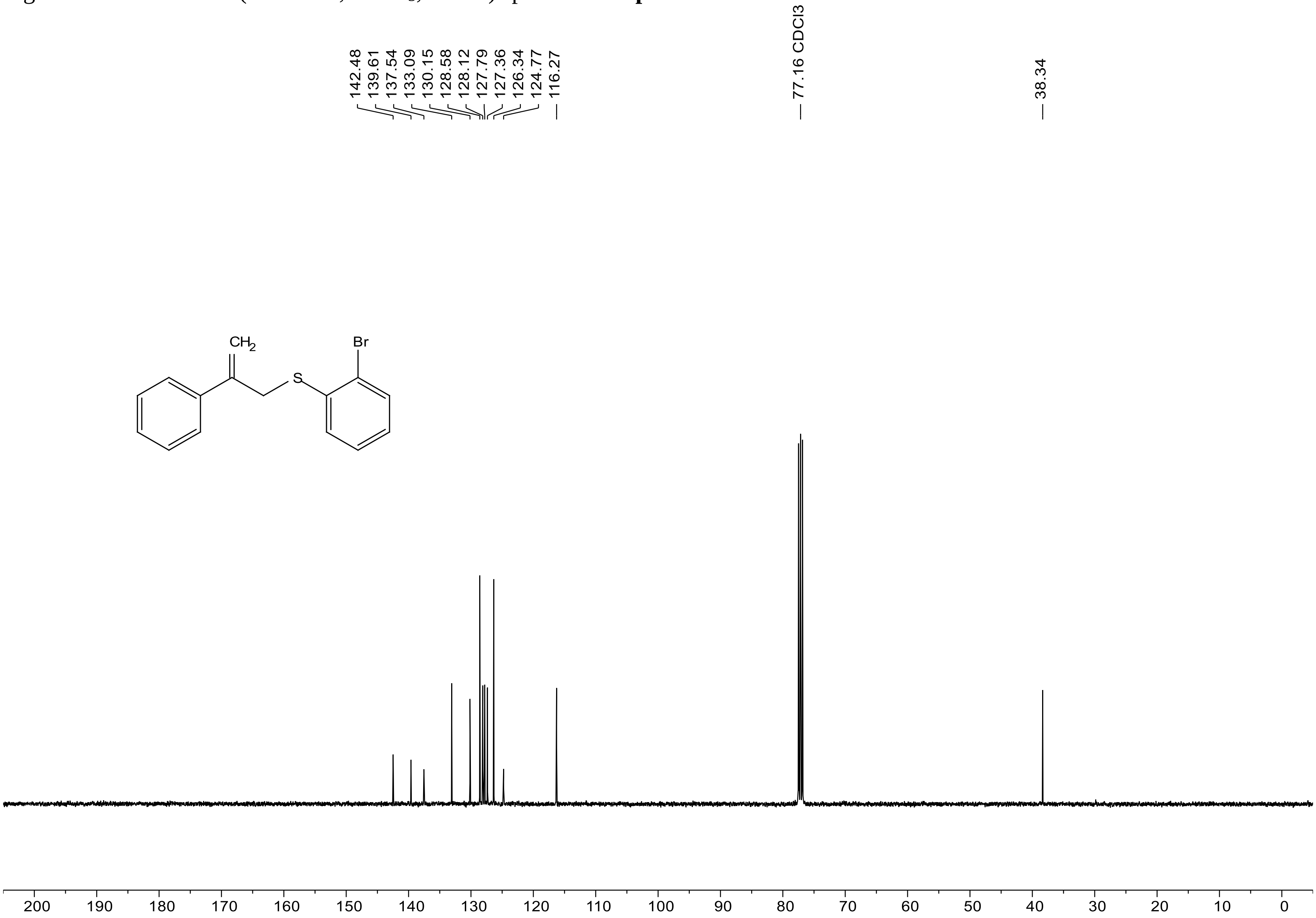

Figure S151: **^{1}H NMR** (400 MHz, $CDCl_3$, 298 K) spectrum of **4pm**.

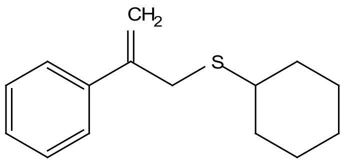

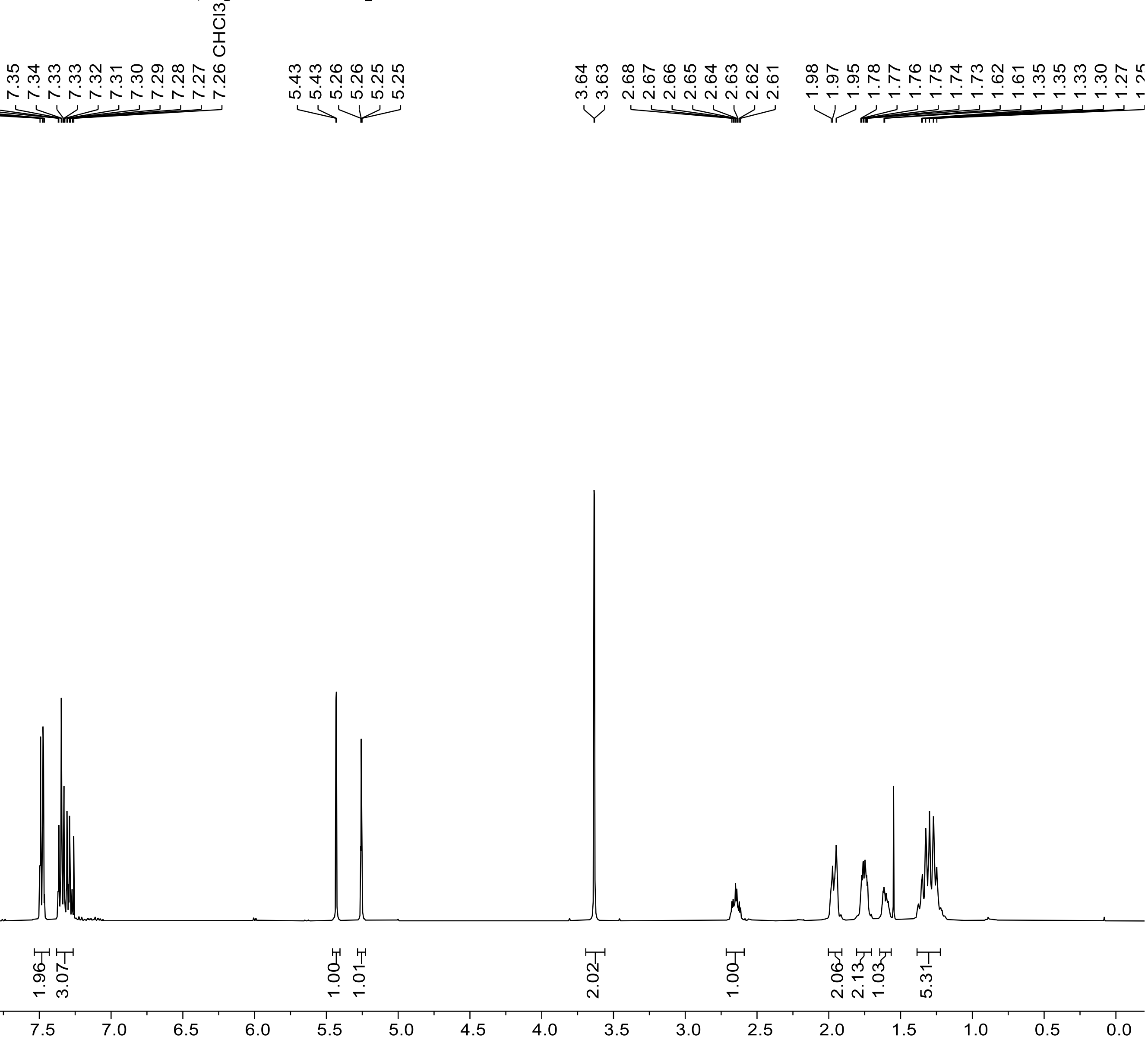

Figure S152: **^{13}C NMR** (101 MHz, $CDCl_3$, 298 K) spectrum of **4pm**.

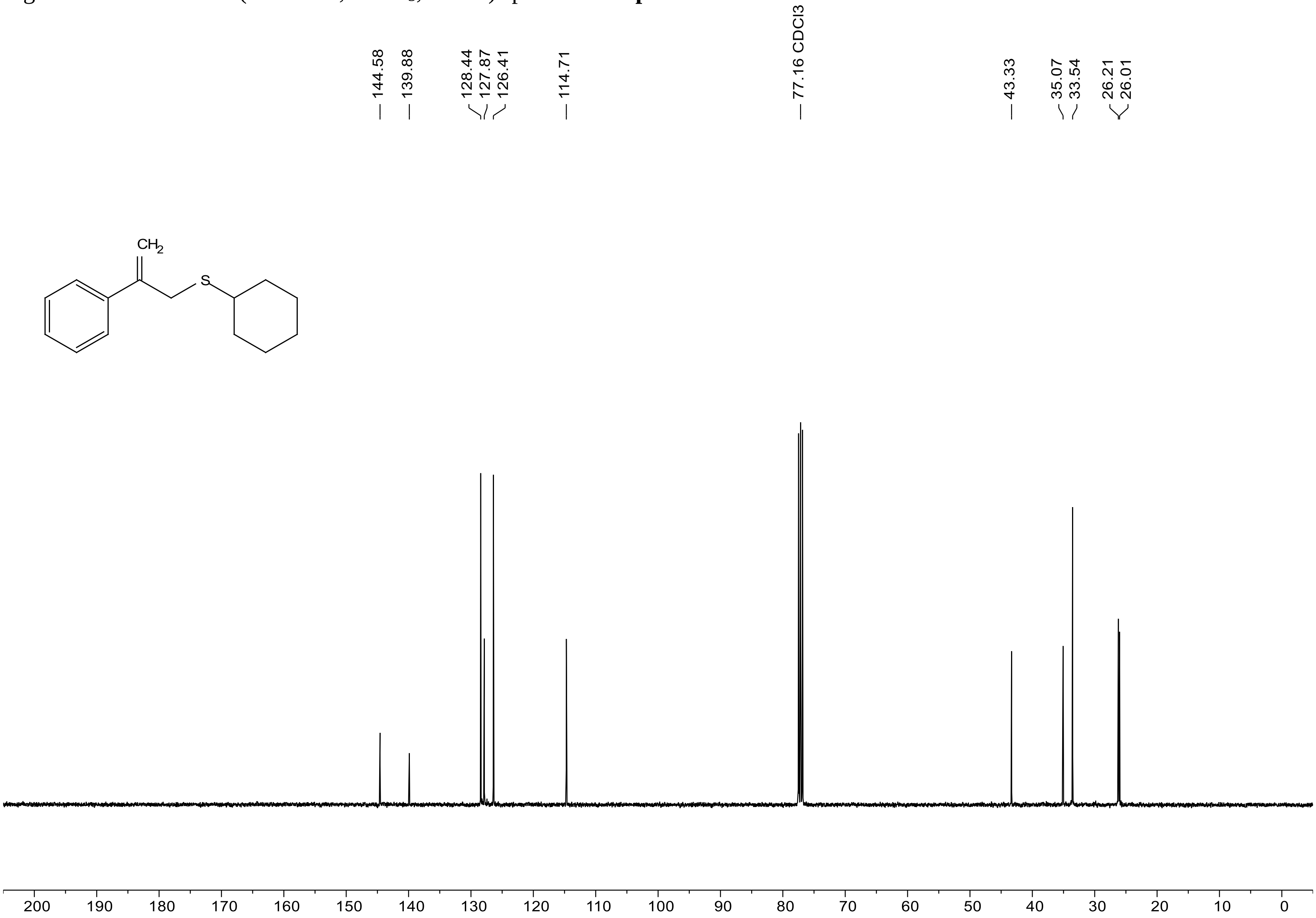

Figure S153: **^{1}H NMR** (400 MHz, $CDCl_3$, 298 K) spectrum of **5**.

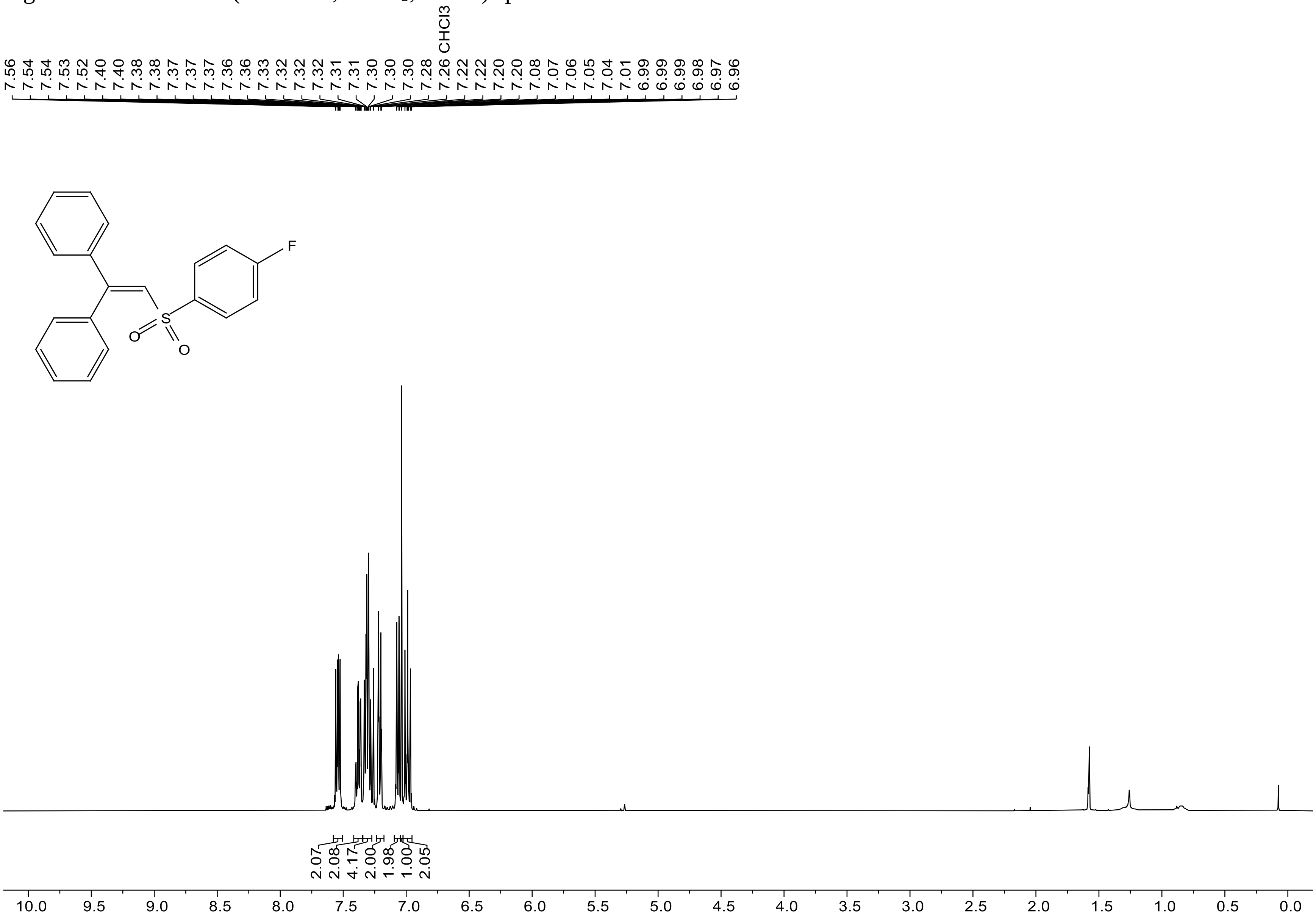

Figure S154: ^{13}**C NMR** (101 MHz, $CDCl_3$, 298 K) spectrum of **5**.

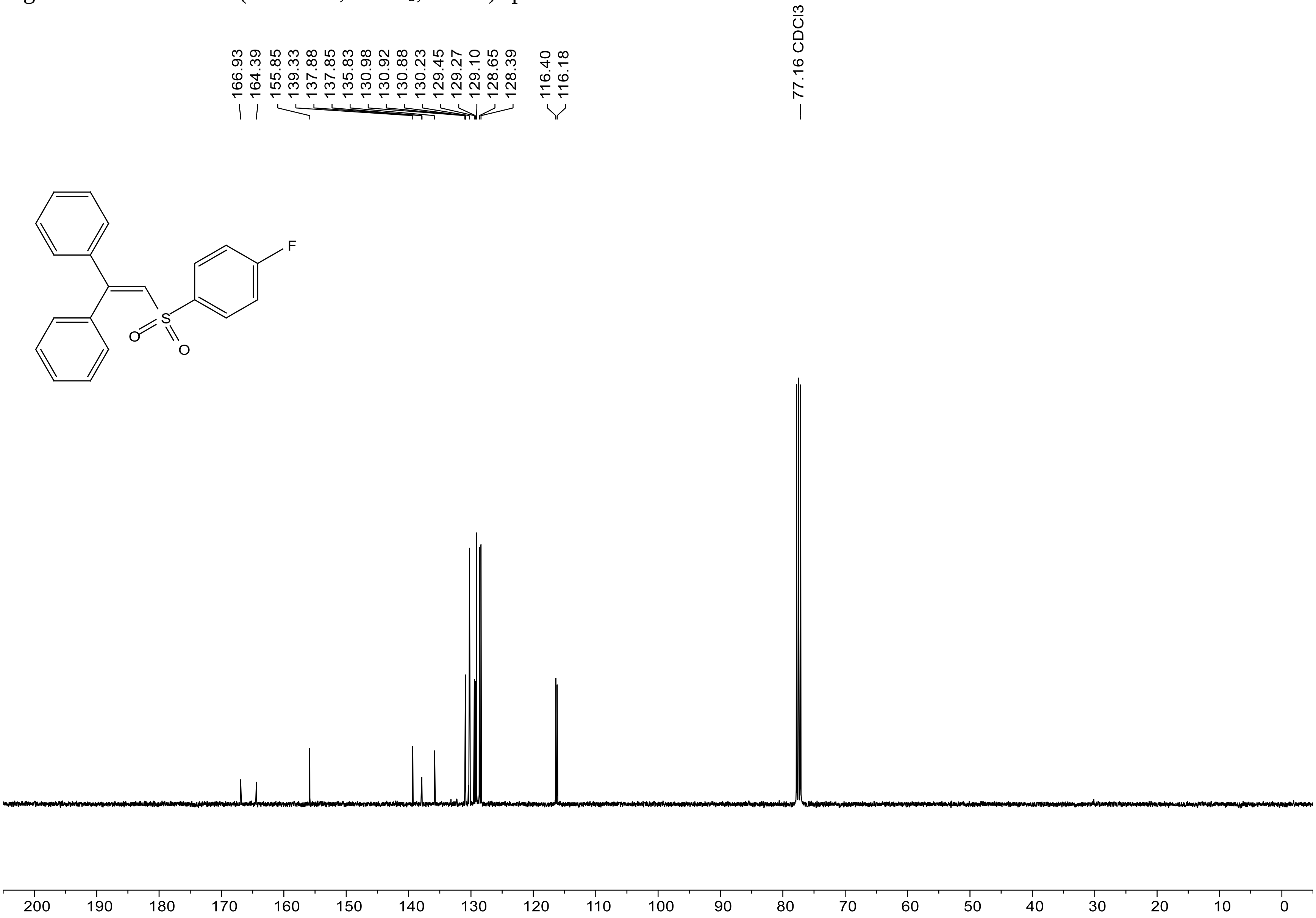

Figure S155: **^{19}F NMR** (376 MHz, $CDCl_3$, 298 K) spectrum of **5**.

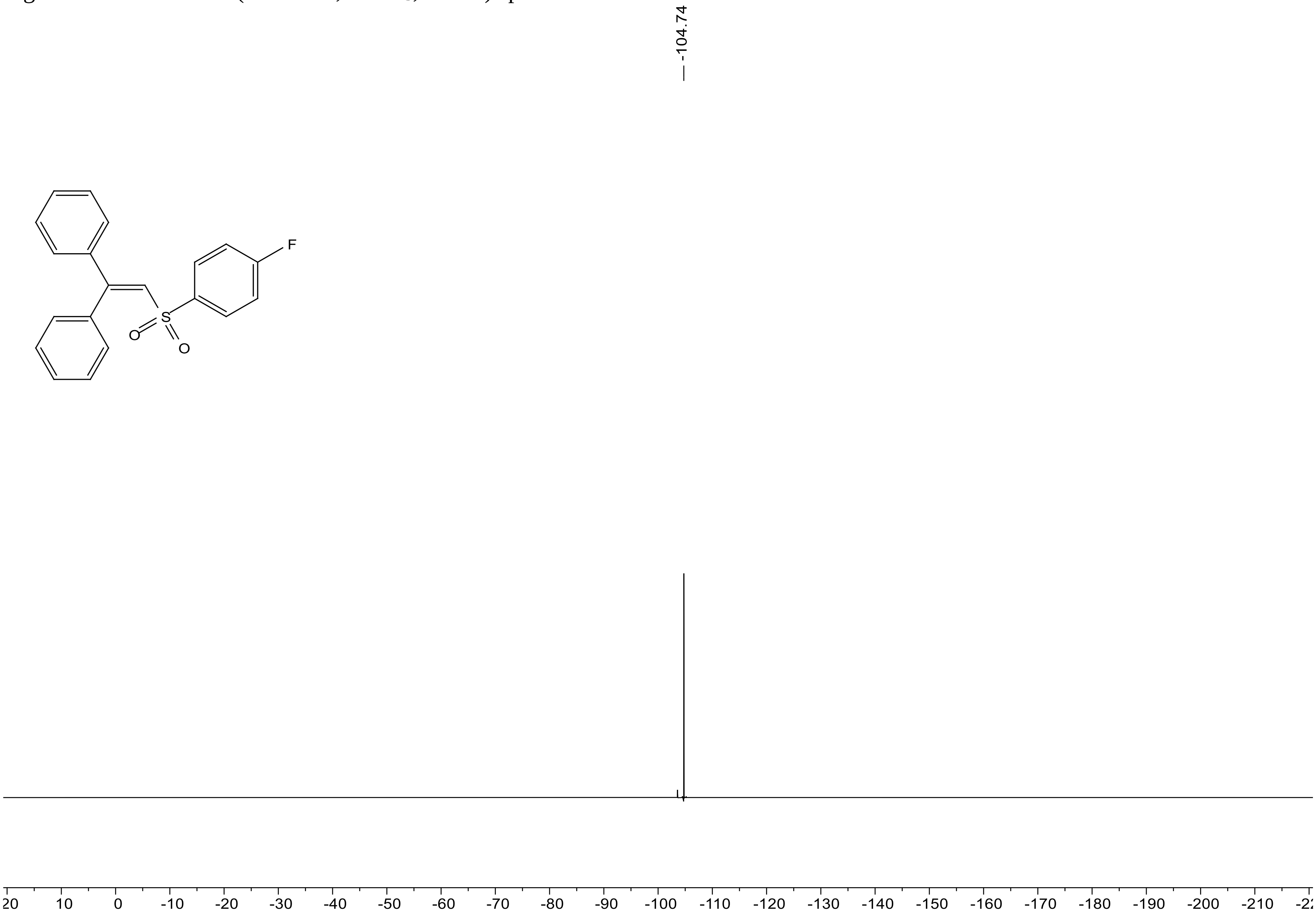

Figure S156: **^{1}H NMR** (400 MHz, $CDCl_3$, 298 K) spectrum of **6**.

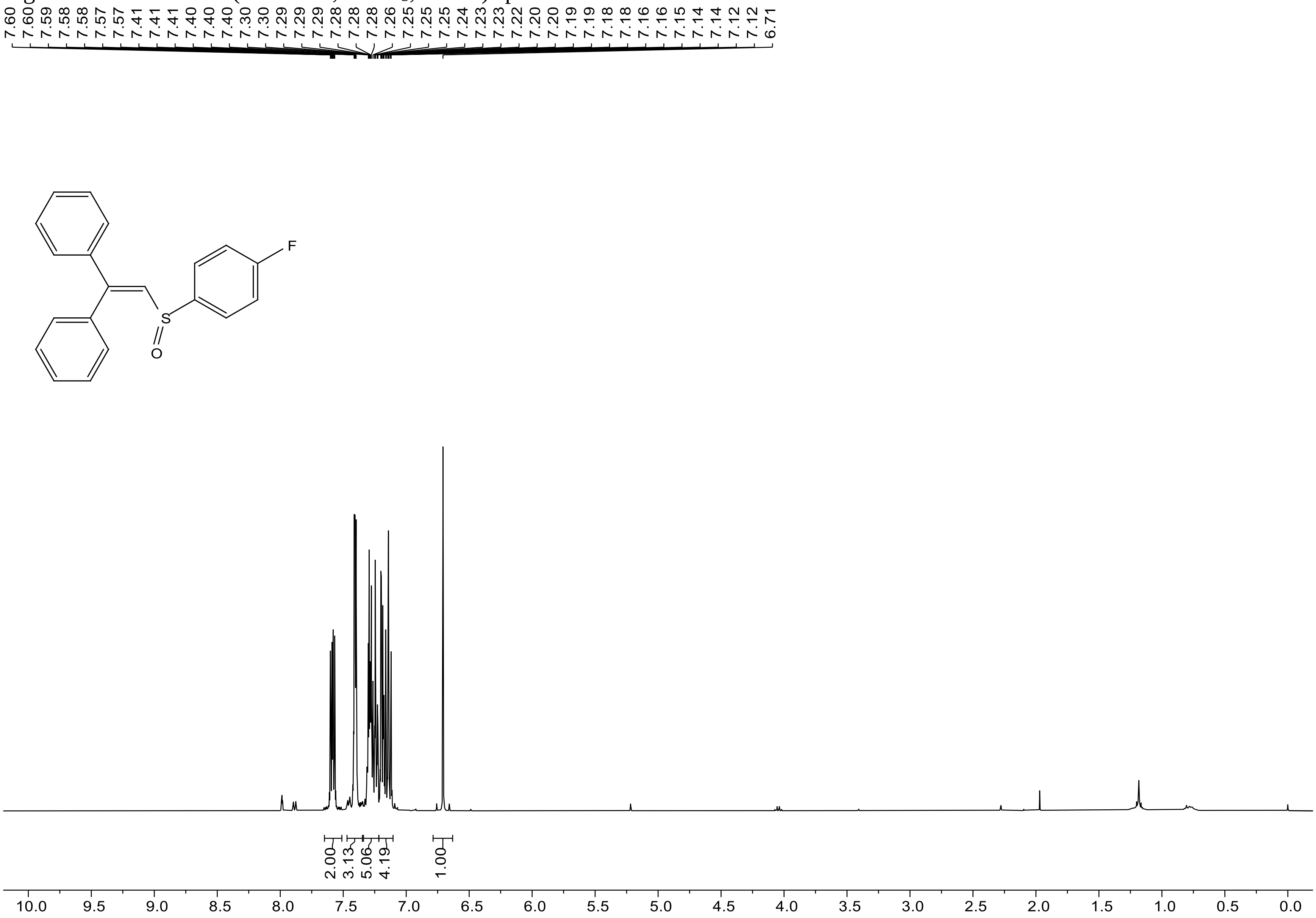

Figure S157: **^{13}C NMR** (101 MHz, $CDCl_3$, 298 K) spectrum of **6**.

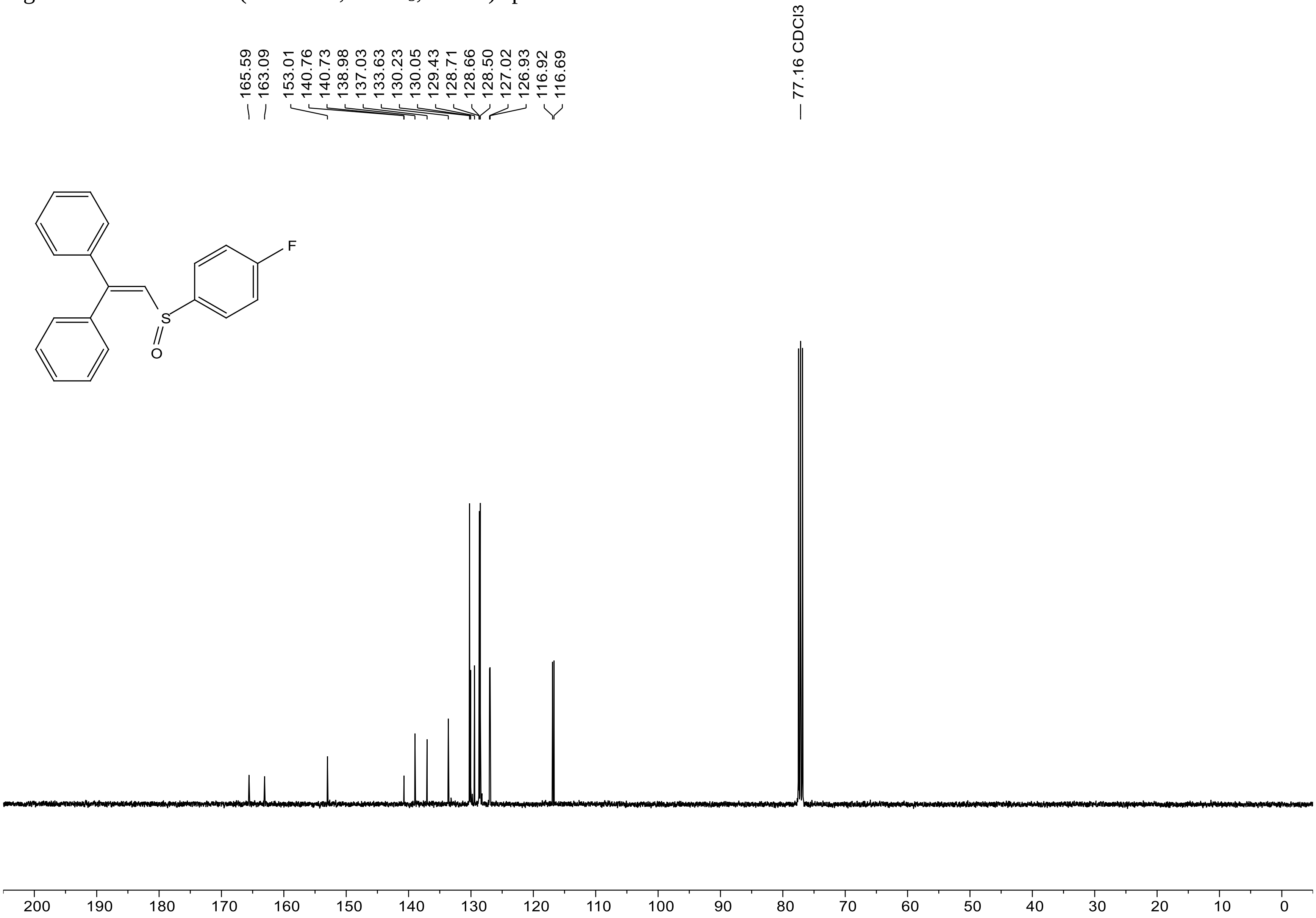

Figure S158: **^{19}F NMR** (376 MHz, $CDCl_3$, 298 K) spectrum of **6**.

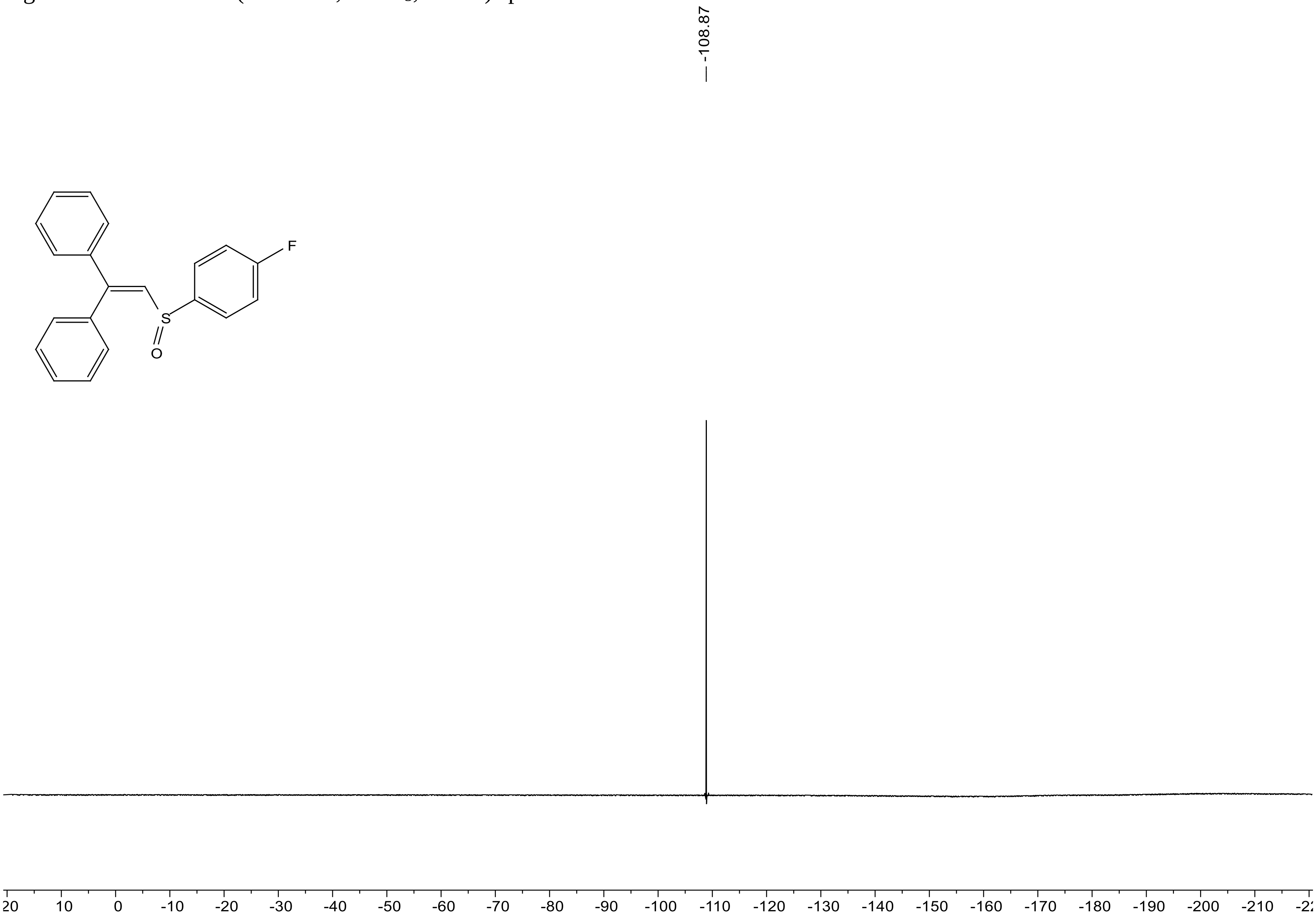

Figure S159: **^{1}H NMR** (400 MHz, $CDCl_3$, 298 K) spectrum of **7**.

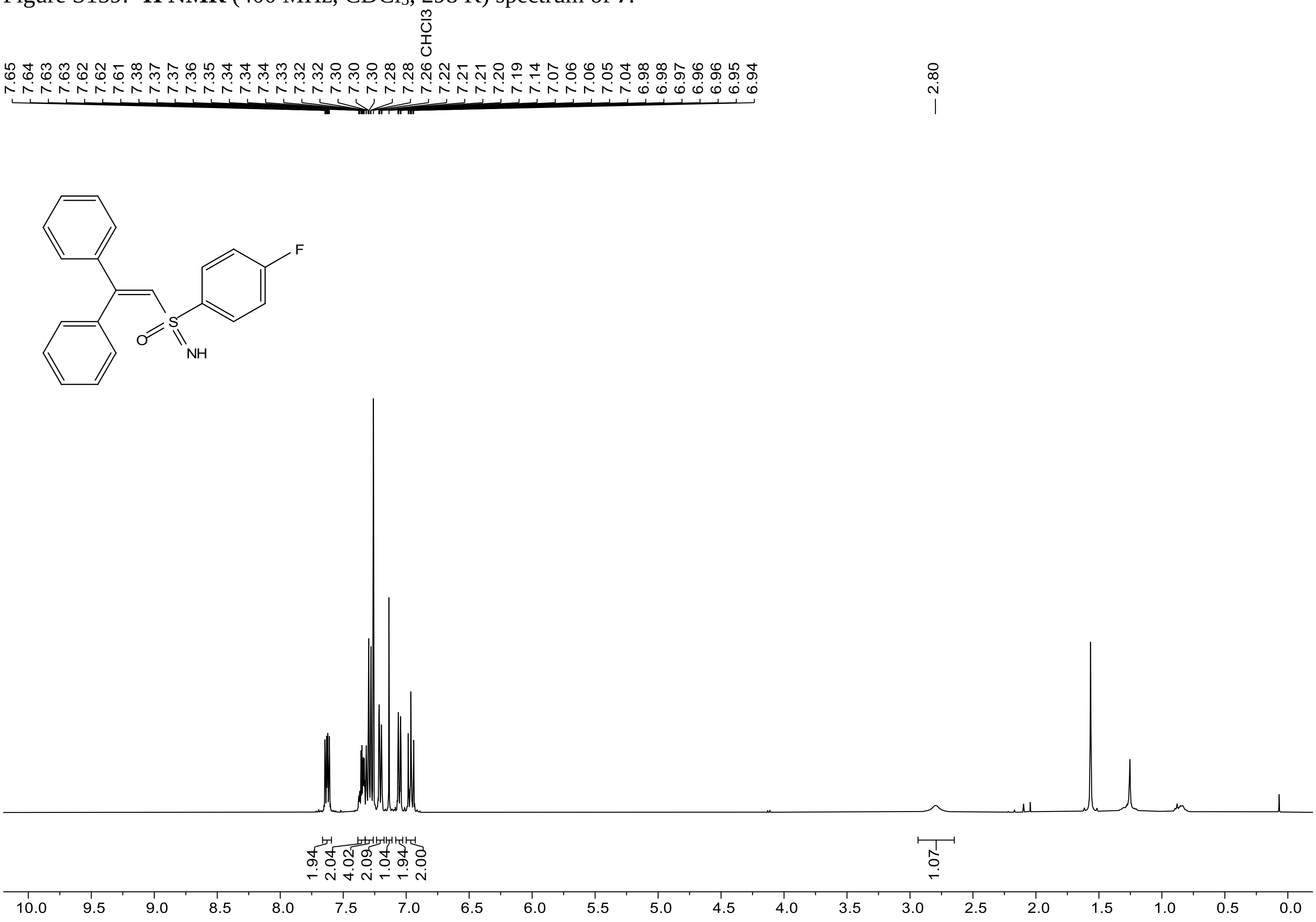

Figure S160: **^{13}C NMR** (101 MHz, $CDCl_3$, 298 K) spectrum of **7**.

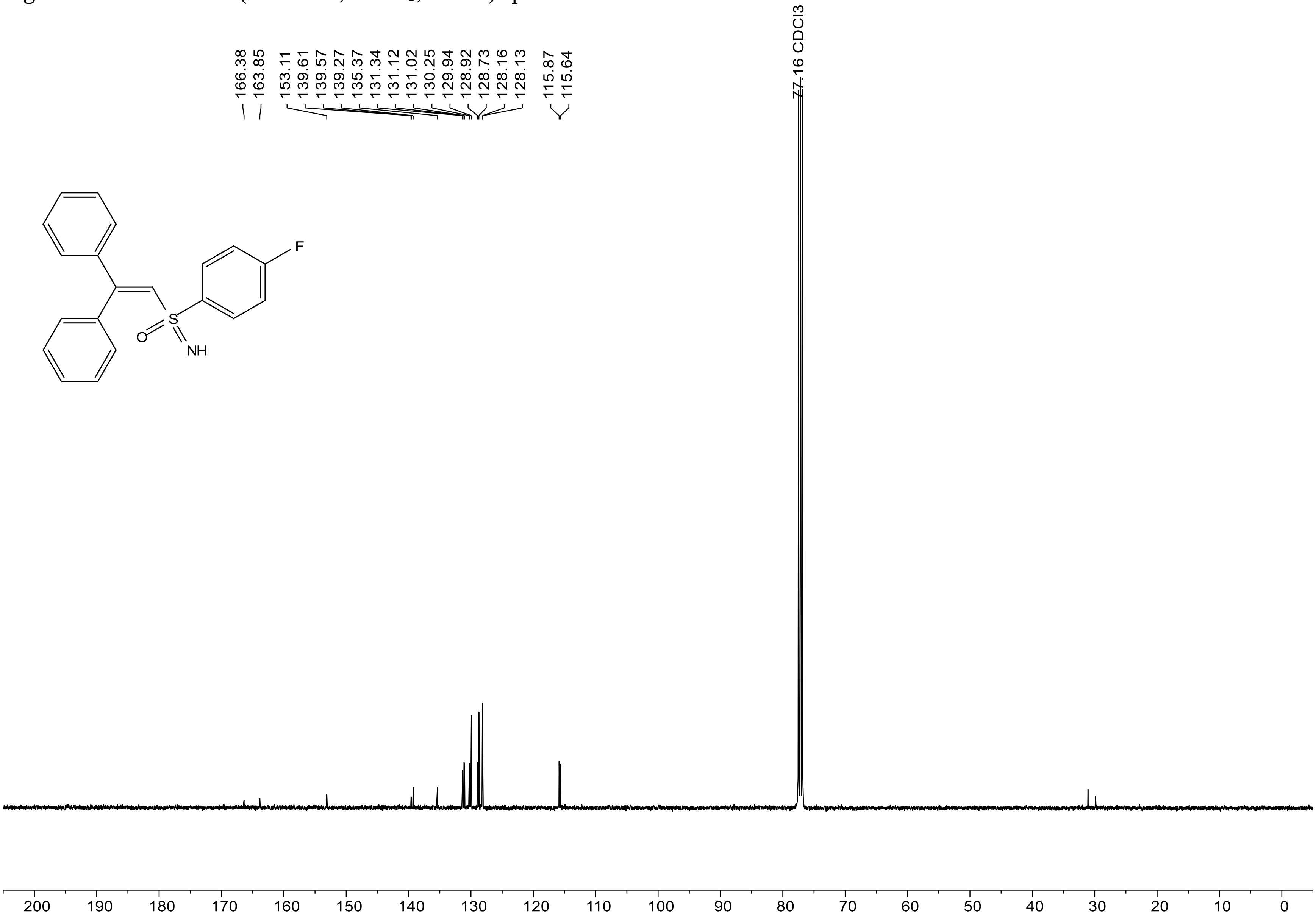

Figure S161: ^{19}**F NMR** (376 MHz, $CDCl_3$, 298 K) spectrum of **7**.

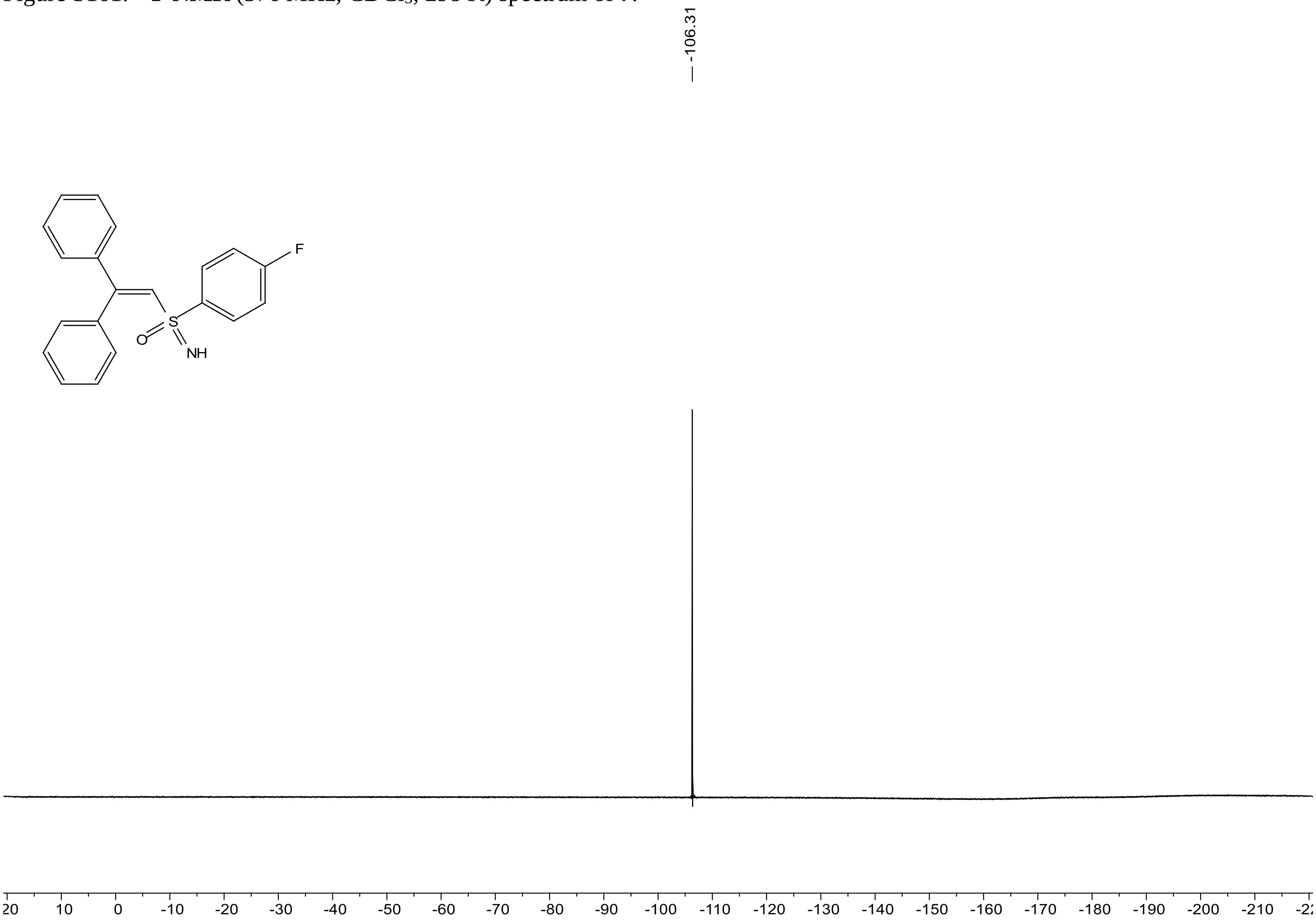

Figure S162: **^{1}H NMR** (400 MHz, $CDCl_3$, 298 K) spectrum of **8**.

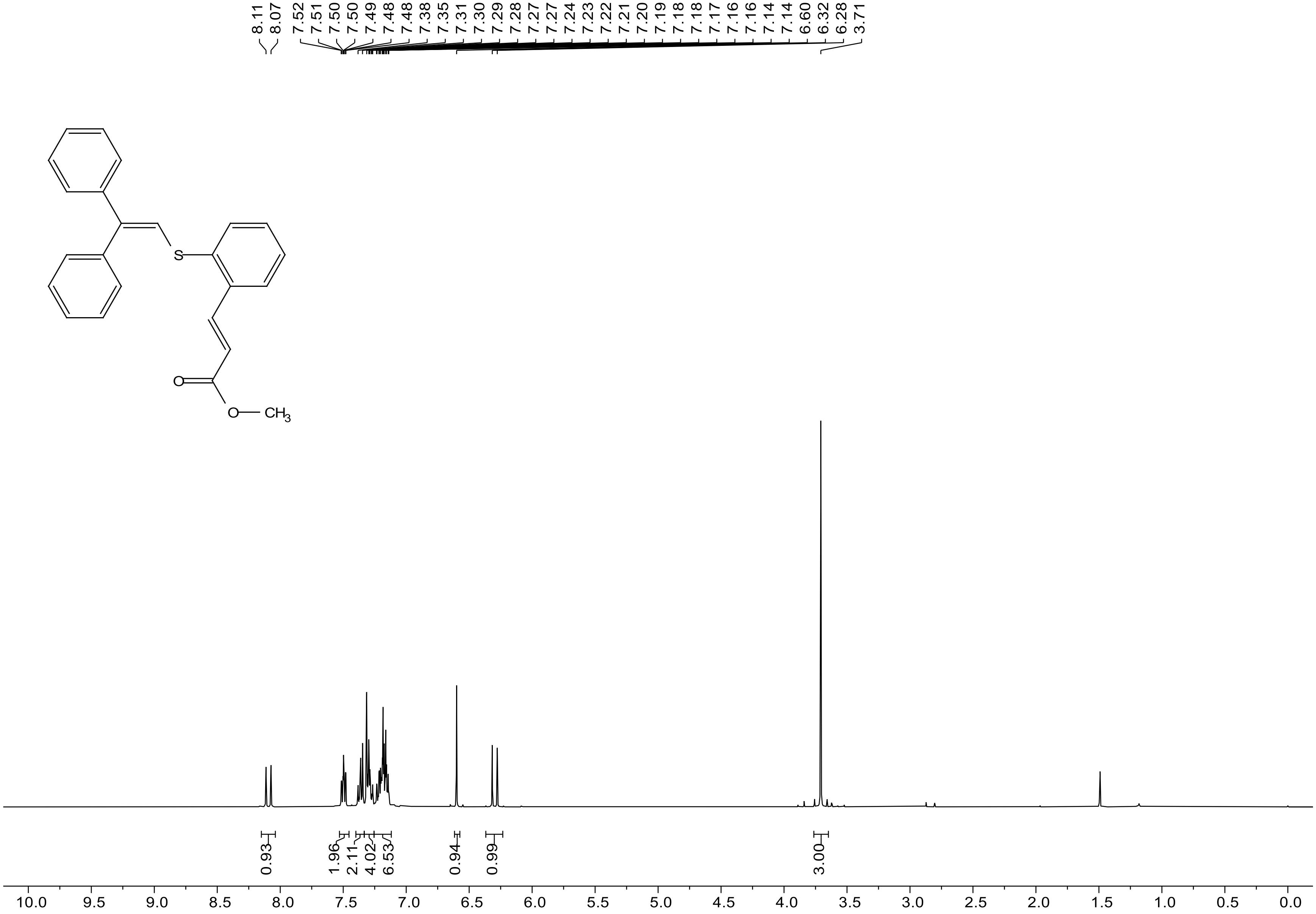

Figure S163: **^{13}C NMR** (101 MHz, $CDCl_3$, 298 K) spectrum of **8**.

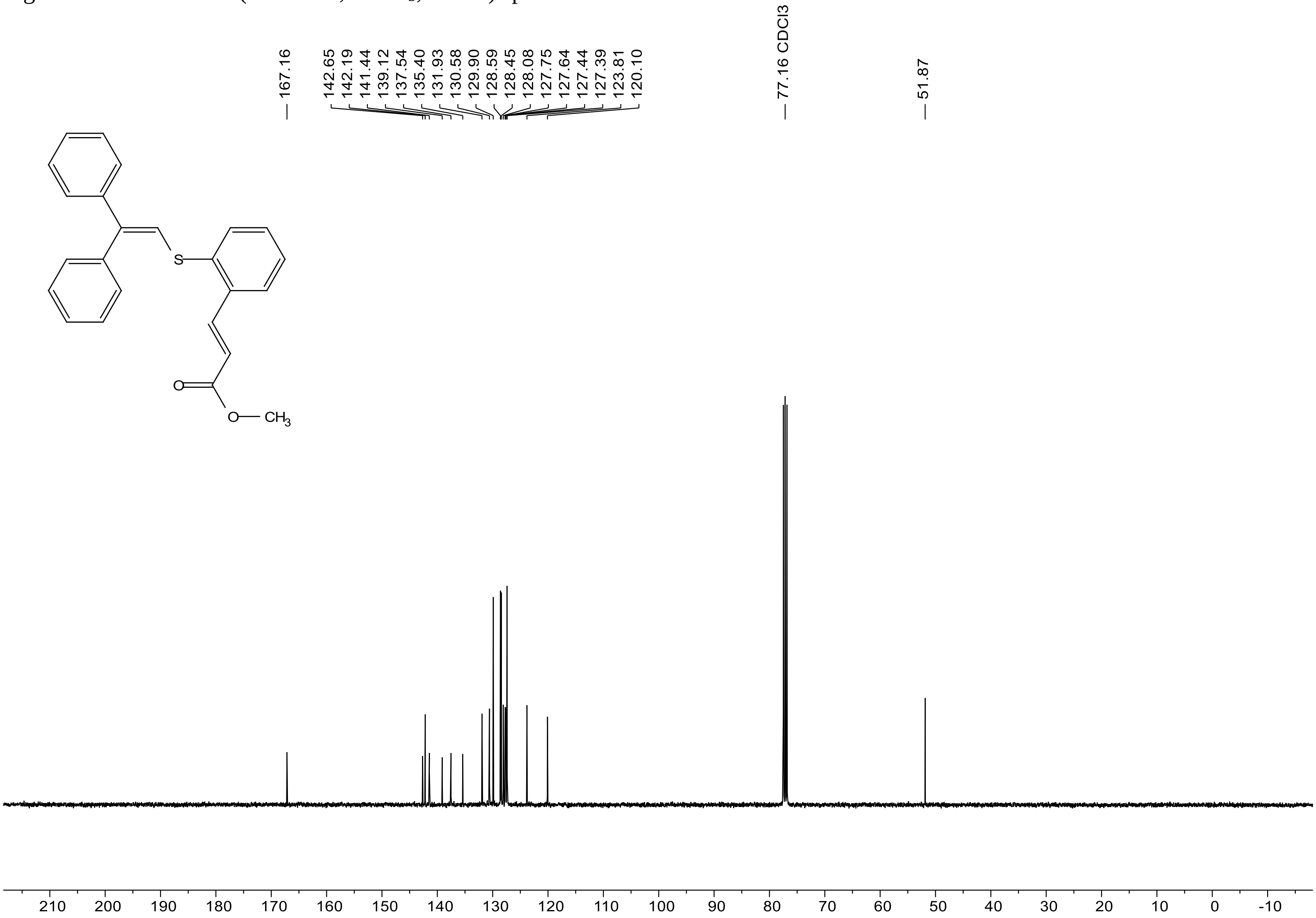

Figure S164: ^{1}**H NMR** (400 MHz, $CDCl_3$, 298 K) spectrum of **9**.

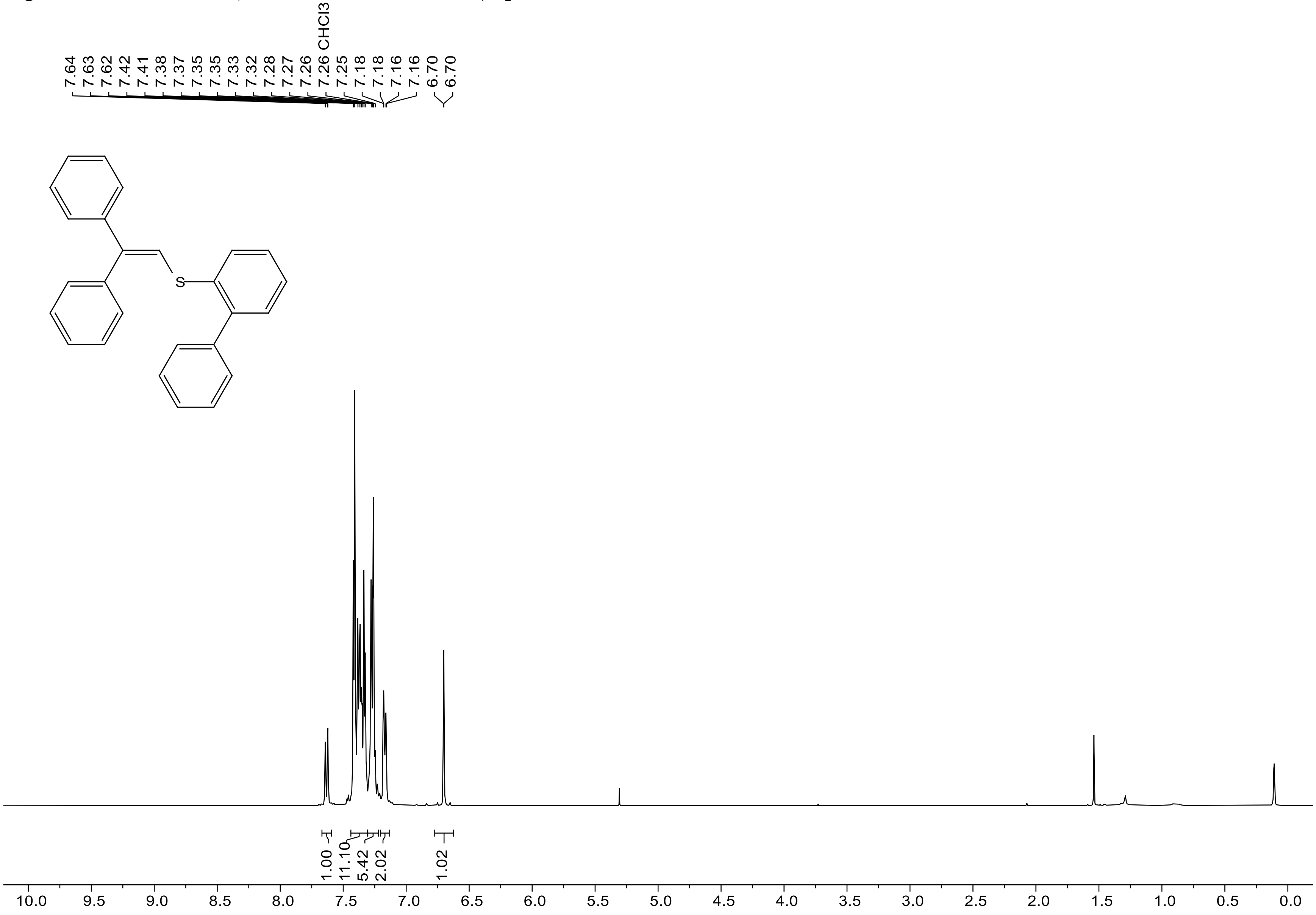

Figure S165: **^{13}C NMR** (101 MHz, $CDCl_3$, 298 K) spectrum of **9**.

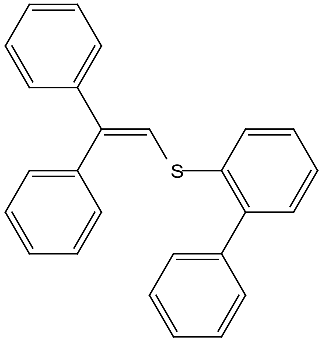

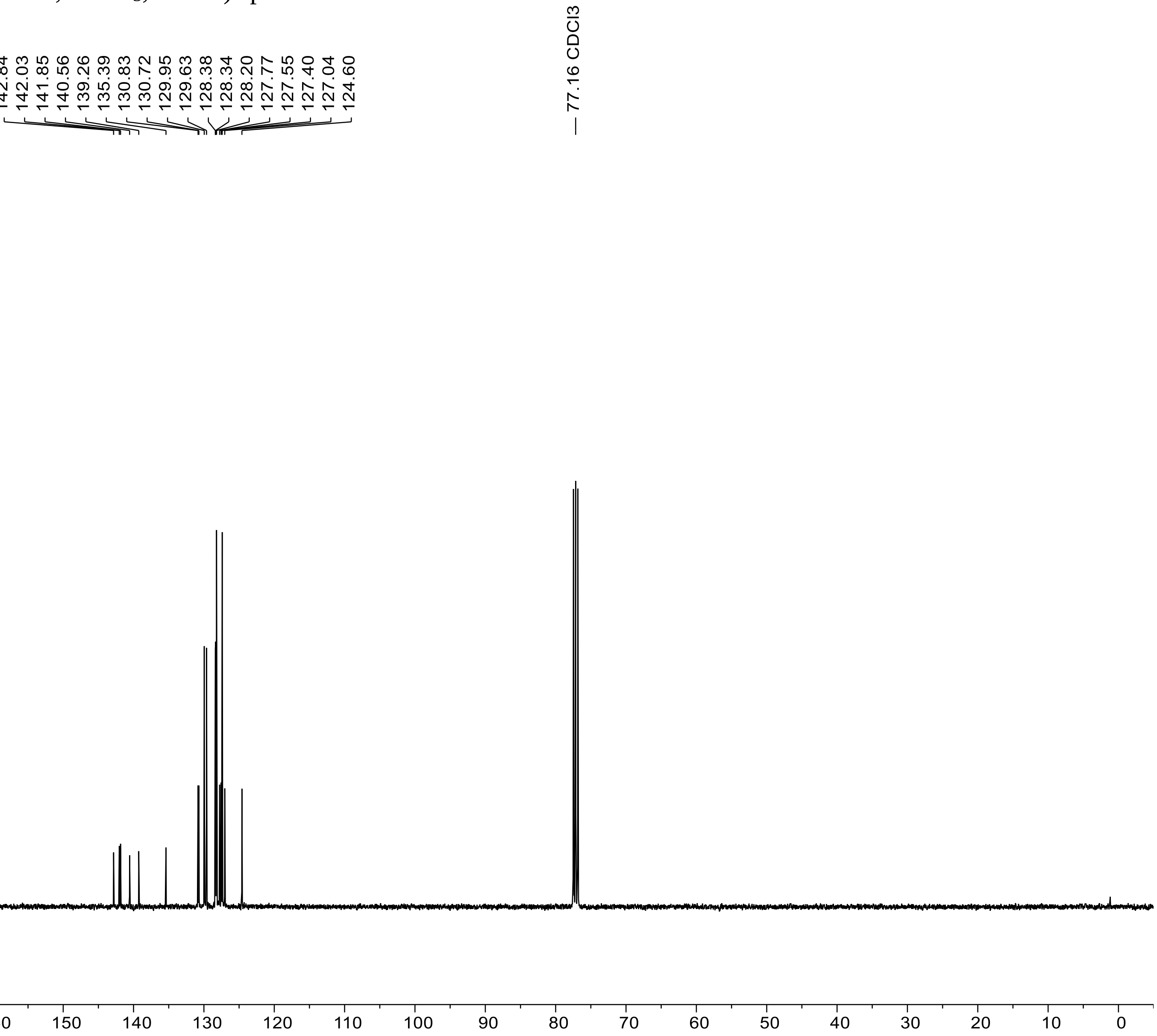

6. References

[1] Santi, M.; Ould, D. M.; Wenz, J.; Soltani, Y.; Melen, R. L.; Wirth, T. Metal-Free Tandem Rearrangement/Lactonization: Access to 3,3-Disubstituted Benzofuran-2-(3*H*)-ones. *Angew. Chem., Int. Ed.* **2019**, *58,* 7861−7865.

[2] Kesavan, A.; Sahu, A. K.; Anbarasan, P. Acid-Promoted Carbosulfenylation of 1, 6-Dienes: Selective Synthesis of Dehydropiperidines Scaffolds. *Org. Lett.* **2023**, *25*, 4765–4769.

[3] Pramanik, M.; Das, S.; Babaahmadi, R.; Pahar, S.; Wirth, T.; Richards, E.; Melen, R. L. B $(C_6F_5)_3$-catalyzed selective C–H chalcogenation of arenes and heteroarenes. *Chem* **2024**, DOI: org/10.1016/j.chempr.2024.05.025.

[4] Xia, D.; Wu, R.; Wang, J.; Han, X.; Li, Y.; Li, Q.; Luan, X.; Hong, X.; Zhang, Y.; Zhang, W.-D. Visible-Light-Mediated Energy Transfer Enables Cyclopropanes Bearing Contiguous All-Carbon Quaternary Centers. *ACS Catal.* **2023**, *13*, 9806–9816.

[5] Namai, H.; Ikeda, H.; Kato, N.; Mizuno, K. Substituent effects on the energies of the electronic transitions of geminally diphenyl-substituted trimethylenemethane (TMM) radical cations. Experimental and theoretical evidence for a twisted molecular and localized electronic structure. *J. Phys. Chem. A* **2007**, *111*, 4436–4442.

[6] Zhou, Y.; Zhao, L.; Hu, M.; Duan, X.-H.; Liu, L. Visible-Light Photoredox-Catalyzed Divergent 1, 2-Diacylation and Hydroacylation of Alkenes with Carboxylic Acid Anhydride. *Org. Lett.* **2023**, *25*, 5268–5272.

[7] K. Lienkamp, L. Noé, M.-H. Breniaux, I. Lieberwirth, F. Groehn and G. Wegner, *Macromolecules* **2007**, *40*, 2486–2502.

[8] Lei, C.; Yip, Y. J.; Zhou, J. S. Nickel-catalyzed direct synthesis of aryl olefins from ketones and organoboron reagents under neutral conditions. *J. Am.Chem. Soc.* **2017**, *139*, 6086–6089.

[9] H. Yang, E. Wang, P. Yang, H. Lv and X. Zhang, *Org. Lett.* **2017**, *19*, 5062–5065.

[10] Wei, W.; Dai, X.-J.; Wang, H.; Li, C.; Yang, X.; Li, C.-J. Ruthenium (II)-catalyzed olefination via carbonyl reductive cross-coupling. *Chem. Sci.* **2017**, *8*, 8193–8197.

[11] Li, J.; Li, J.; He, R.; Liu, J.; Liu, Y.; Chen, L.; Huang, Y.; Li, Y. Selective Synthesis of Substituted Pyridines and Pyrimidines through Cascade Annulation of Isopropene Derivatives. *Org. Lett.* **2022**, *24*, 1620–1625.

[12] Cussó, O.; Ribas, X.; Lloret-Fillol, J.; Costas, M. Synergistic Interplay of a Non-Heme Iron Catalyst and Amino Acid Coligands in H2O2 Activation for Asymmetric Epoxidation of α-Alkyl-Substituted Styrenes. *Angew. Chem., Int. Ed.* **2015**, *54*, 2729–2733.

[13] Zhang, X.; Liang, N.; Li, R.; Sun, Z. Application of Halogen-Bonding Catalysis for Markovnikov-Type Hydrothiolation of Alkenes. *Synlett* **2023**, *34*, 379–387.

[14] Walker, J. C.; Oestreich, M. Regioselective transfer hydrodeuteration of alkenes with a hydrogen deuteride surrogate using B (C_6F_5)$_3$ catalysis. *Org. Lett.* **2018**, *20*, 6411–6414.

[15] Jiang, M.; Wei, Y.; Shi, M. Palladium Acetate Catalyzed Oxidative Aromatization of Methylenecyclopropanes. *Eur. J. Org. Chem.* **2010**, 3307–3311.

[16] Movahhed, S.; Westphal, J.; Dindaroğlu, M.; Falk, A.; Schmalz, H. G. Low-Pressure Cobalt-Catalyzed Enantioselective Hydrovinylation of Vinylarenes. *Chem. Eur. J.* **2016**, *22*, 7381–7384.

[17] Zeng, H.; Hua, R. Palladium-catalyzed hydrophenylation of alkynes with sodium tetraphenylborate under mild conditions. *J. Org. Chem.* **2008**, *73*, 558–562.

[18] Wang, B. W.; Jiang, K.; Li, J. X.; Luo, S. H.; Wang, Z. Y.; Jiang, H. F. 1, 1-Diphenylvinylsulfide as a Functional AIEgen Derived from the Aggregation-Caused-Quenching Molecule 1, 1-Diphenylethene through Simple Thioetherification. *Angew. Chem., Int. Ed.* **2020**, *59*, 2338–2343.

[19] Zhang, J.; Wang, E.; Zhou, Y.; Zhang, L.; Chen, M.; Lin, X. A metal-free synthesis of 1, 1-diphenylvinylsulfides with thiols via thioetherification under continuous-flow conditions. *Org. Chem. Front.* **2020**, *7*, 1490–1494.

[20] Liu, C.; Xu, J.; Wu, G. Sodium iodide-mediated synthesis of vinyl sulfides and vinyl sulfones with solvent-controlled chemical selectivity. *RSC Adv.* **2021**, *11*, 35156–35160.

[21] Mukherjee, N.; Chatterjee, T. Recyclable iodine-catalyzed oxidative C–H chalcogenation of 1, 1-diarylethenes in water: green synthesis of trisubstituted vinyl sulfides and selenides. *Green Chem.* **2023**, *25*, 8798–8807.

[22] Li, C.; Li, J.; Tan, C.; Wu, W.; Jiang, H. DDQ-mediated regioselective C–S bond formation: efficient access to allylic sulfides. *Org. Chem. Front.* **2018**, *5*, 3158–3162.

[23] Liu, S.; Wang, L.; Ma, Z.; Zeng, X.; Xu, B. Pyridine hydrochloride-catalyzed thiolation of alkenes: divergent synthesis of allyl and vinyl sulfides. *Org. Chem. Front.* **2020**, *7*, 3474–3479.

[24] An, Y.; Wu, J. Synthesis of tetrahydropyridine derivatives through a reaction of 1, 6-enynes, sulfur dioxide, and aryldiazonium tetrafluoroborates. *Org. Lett.* **2017**, *19*, 6028–6031.

[25] Zhang, G.; Fu, J.-G.; Zhao, Q.; Zhang, G.-S.; Li, M.-Y.; Feng, C.-G.; Lin, G.-Q. Silver-promoted synthesis of vinyl sulfones from vinyl bromides and sulfonyl hydrazides in water. *Chem. Commun.* **2020**, *56*, 4688–4691.

[26] Frisch, M. J.; Trucks, G. W.; Schlegel, H. B.; Scuseria, G. E.; Robb, M. A.; Cheeseman, J. R.; Scalmani, G.; Barone, V.; Mennucci, B.; Petersson, G. A.; Nakatsuji, H.; Caricato, M.; Li, X.; Hratchian, H. P.; Izmaylov, A. F.; Bloino, J.; Zheng, G.; Sonnenberg, J. L.; Hada, M.; Ehara, M.; Toyota, K.; Fukuda, R.; Hasegawa, J.; Ishida, M.; Nakajima, T.; Honda, Y.; Kitao, O.; Nakai, H.; Vreven, T.; Montgomery, J. A., Jr.; Peralta, J. E.; Ogliaro, F.; Bearpark, M.; Heyd, J. J.; Brothers, E.; Kudin, K. N.; Staroverov, V. N.; Kobayashi, R.; Normand, J.; Raghavachari, K.; Rendell, A.; Burant, J. C.; Iyengar, S. S.; Tomasi, J.; Cossi, M.; Rega, N.; Millam, J. M.; Klene, M.; Knox, J. E.; Cross, J. B.; Bakken, V.; Adamo, C.; Jaramillo, J.; Gomperts, R.; Stratmann, R. E.; Yazyev, O.; Austin, A. J.; Cammi, R.; Pomelli, C.; Ochterski, J. W.; Martin, R. L.; Morokuma, K.; Zakrzewski, V. G.; Voth, G. A.; Salvador, P.; Dannenberg, J. J.; Dapprich, S.; Daniels, A. D.; Farkas, O.; Foresman, J. B.; Ortiz, J. V.; Cioslowski, J.; Fox, D. J. Gaussian 09, Revision B.01; Gaussian, Inc.: Wallingford, CT, **2009**.

[27] Zhao, Y., and Truhlar, D. G. Density Functionals with Broad Applicability in Chemistry. *Acc. Chem. Res.* **2008**, *41*, 157–167.

[28] Hariharan, P. C., and Pople, J. A. The influence of polarization functions on molecular orbital hydrogenation energies. *Theor. Chem. Acc.* **1973**, *28*, 213–222.

[29] Marenich, A. V., Cramer, C. J., Truhlar, D. G. Universal Solvation Model Based on Solute Electron Density and on a Continuum Model of the Solvent Defined by the Bulk Dielectric Constant and Atomic Surface Tensions. *J. Phys. Chem. B* **2009**, *113*, 6378–6396.

[30] Weigend, F., Furche, F., Ahlrichs, R. Gaussian basis sets of quadruple zeta valence quality for atoms H–Kr. *J. Chem. Phys.* **2003**, *119*, 12753–12762.

[31] Ochterski, J. W., Thermochemistry, Gaussian, Inc., Wallingford, CT, **2000**.

[32] Legault, C. Y. CYLview, 1.0b; Université de Sherbrooke: Québec, Canada, **2009**; available at http://www.cylview.org.
